# Identification of putative regulatory motifs in the upstream regions of co-expressed functional groups of genes in *Plasmodium falciparum*

**DOI:** 10.1186/1471-2164-10-18

**Published:** 2009-01-13

**Authors:** Prathima Iengar, NV Joshi

**Affiliations:** 1 Molecular Biophysics Unit, Indian Institute of Science, Bangalore, 560012, India; 2 Centre for Ecological Sciences, Indian Institute of Science, Bangalore, 560012, India

## Abstract

**Background:**

Regulation of gene expression in *Plasmodium falciparum (Pf) *remains poorly understood. While over half the genes are estimated to be regulated at the transcriptional level, few regulatory motifs and transcription regulators have been found.

**Results:**

The study seeks to identify putative regulatory motifs in the upstream regions of 13 functional groups of genes expressed in the intraerythrocytic developmental cycle of *Pf*. Three motif-discovery programs were used for the purpose, and motifs were searched for only on the gene coding strand. Four motifs – the 'G-rich', the 'C-rich', the 'TGTG' and the 'CACA' motifs – were identified, and zero to all four of these occur in the 13 sets of upstream regions. The 'CACA motif' was absent in functional groups expressed during the ring to early trophozoite transition. For functional groups expressed in each transition, the motifs tended to be similar. Upstream motifs in some functional groups showed 'positional conservation' by occurring at similar positions relative to the translational start site (TLS); this increases their significance as regulatory motifs. In the ribonucleotide synthesis, mitochondrial, proteasome and organellar translation machinery genes, G-rich, C-rich, CACA and TGTG motifs, respectively, occur with striking positional conservation. In the organellar translation machinery group, G-rich motifs occur close to the TLS. The same motifs were sometimes identified for multiple functional groups; differences in location and abundance of the motifs appear to ensure different modes of action.

**Conclusion:**

The identification of positionally conserved over-represented upstream motifs throws light on putative regulatory elements for transcription in *Pf*.

## Background

The life-cycle of the malarial parasite, *Plasmodium falciparum *(*Pf*), consists of several morphological forms which occur in the mosquito, liver and blood stages (e.g., gamete, salivary gland sporozoite, ring, trophozoite, schizont, merozoite, gametocyte). *Pf *generates these morphologies by regulating its gene expression; the morphological transformations are accompanied by changes in the RNA and protein repertoires of the cell [1,2]. The transcriptome of the *Pf *asexual intraerythrocytic developmental cycle (IDC) has been analysed and transcriptional regulation has been shown to orchestrate a continuous cascade of gene expression, with genes being induced once per cycle, in a "just-in-time" manner, only when the gene product is required by the cell [3]. The question then arises as to how such fine-tuned regulation of gene expression is achieved in this extreme parasite.

In functional genomics, it is believed to be likely that genes with similar mRNA expression profiles (co-expressed genes) are regulated via the same mechanism (co-regulated); similarly, it is thought to be likely that genes with similar functions are regulated by the same mechanism. Co-expressed genes which also have similar functions are more likely to be co-regulated [4]. They are likely to have conserved DNA sequence elements, called transcription factor binding motifs (TFBMs), in their promoter regions. TFBMs act as binding sites for transcription factors and coordinate the expression of the genes in whose promoter regions they appear. They are about 6–8 nucleotides (nt) in length and occur upstream of the gene transcription start site (TSS). In response to environmental and developmental cues, TFBMs are bound by their cognate transcription factors, and, as a result, co-expression of the associated set of genes takes place, by activation or inhibition of the transcription machinery [4-7].

Transcription of *Pf *genes appears to take place monocistronically, and *Pf *promoters have a bipartite structure consisting of a basal promoter regulated by upstream enhancer elements [8,9]. The number of transcription associated proteins (TAPs) encoded by the *Pf *genome is about one third the number of TAPs associated with the transcriptional process in free-living eukaryotes [10]. The basal transcriptional apparatus (RNA Polymerase II, TATA-box binding protein and other components) in *Pf *is similar to that in free-living eukaryotes. Chromatin remodeling complexes and histone acetylases and deacetylases, which modulate the accessibility of DNA to transcription factors, are also present. However, there are relatively few proteins in *Pf *which show homology to known transcriptional regulators and which contribute to gene-specific transcriptional regulation [10]. Thus, it would appear that a system of transcriptional regulators (activators and repressors) might not be the sole mechanism by which coordinated expression of genes is achieved during the *Pf *life-cycle.

Post-transcriptional mechanisms have been proposed to play an important role in the regulation of gene expression in *Pf *[11]. The CCCH-type zinc finger domain (CCCH stands for C-x8-C-x5-C-x3-H, where x is any amino acid) was found to be almost twice as abundant in the *Pf *genome as in free-living eukaryotes. This type of zinc finger functions in regulating mRNA stability and translation [10]. Further, the *Pf *genome encodes homologues of components of the CCR4-NOT complex, which has cytoplasmic mRNA deadenylase activity; deadenylation modifies the rates of translation initiation and is the rate-limiting step in mRNA decay. mRNA decay rates, on a genome-wide scale, during the IDC of *Pf *have been determined [12]. Globally, mRNA decay rates were found to increase during the IDC (from 9.5 min in the ring stage to 65 min in the schizont stage). The authors suggest that, in the late stage parasites, the morphological changes taking place (packaging multiple nuclei into merozoites) may complicate regulation of transcription, and, instead, global stabilization of mRNA may be regulating gene expression. All these observations provide evidence that post-transcriptional mRNA processing plays an important role in regulating protein levels during the *Pf *life cycle.

Translational repression has also been shown to be a means of regulating gene expression in *Pf *[13]. In gametocytes, there are transcripts which are abundant, but are in a state of translational repression. RNA binding proteins, called Puf proteins, which are upregulated in gametocytes, repress the translation of the mRNAs [14]; they do so by binding to a UUGU motif in the 3' untranslated region (UTR) of their respective target mRNAs. Multiple copies of this motif occur in the 3' UTRs. Another mRNA binding protein that has been characterized is *Pf*IRPa, an iron regulatory-like protein; it binds to an iron-responsive element, a stem-loop structure formed at 5' and 3' UTRs of mRNA, and functions to inhibit translation or to modulate the stability of mRNA [15].

The 4 stages of the *Pf *IDC have been considered and changes in mRNA and protein abundance over the 4 stage-transitions in the cycle – the merozoite/ring, ring/trophozoite, trophozoite/schizont and schizont/merozoite transitions – have been examined [16]. For 55% of the genes, mRNA and protein abundance changes followed similar trends over stage transitions; these were genes whose expression was regulated at the transcriptional level. For the remaining genes, response in protein levels was not complementary to changes in mRNA levels; for most of these genes, however, changes in mRNA and protein levels became complementary when the mRNA abundance changes observed over one transition were compared with protein abundance changes over the *succeeding *transition (for example, when mRNA abundance changes over the merozoite/ring transition were compared with protein abundance changes over the ring/trophozoite transition). These were genes whose expression was regulated at the post-transcriptional level. Thus, transcriptional and post-transcriptional regulation appear to be of equal importance in the *Pf *IDC. However, while transcriptional regulation is important, the *Pf *genome appears to encode fewer transcriptional regulators than free-living eukaryotes [10], and while post-transcriptional regulation is important, the mechanism by which it is achieved is not clear [12].

Given the reasonable importance of transcriptional regulation in *Pf*, in the present study, an attempt has been made to search for potential regulatory sequences in the upstream regions of co-expressed *Pf *genes, using *in silico *motif-discovery methods. Regulatory sites in *Pf *have previously been identified using such procedures. A G-rich regulatory element upstream of several *Pf *heat shock protein (*hsp*) genes has been identified using the AlignACE program [17]. The regulatory element, named as G-box, has the consensus sequence, (A/G)NGGGG(C/A), and has been shown to be required for reporter gene expression in transient transfection experiments. In another study, computational methods have first been used to identify modules of co-regulated *Pf *genes, and motif detection tools have then been used to identify potential TFBMs in these modules [18]. For the *hsp *genes, the G-box motif, and, in addition, a TG-box motif (a G-box preceded by a string of Ts), have been identified. For a set of 77 genes, a putative TFBM containing the core sequence "ACACA" has also been identified. In the upstream regions of 600 chloroquine responsive genes, a significantly over-represented GAGAGAA motif has been identified [19]. The motif formed specific RNA-protein, but not DNA-protein complexes, suggesting that it was regulating gene expression at the RNA level. Phylogenetic footprinting techniques have been integrated with techniques for detecting over-represented motifs, to identify TFBMs or *cis*-regulatory motifs in *Pf *[20]. Upstream regions of co-expressed *Pf *genes, together with the upstream regions of their orthologs in *P. y. yoelii*, have been considered, and AlignACE has been used to find over-represented motifs in the mixed set of sequences. The conservation of *cis*-regulatory motifs among different species has been exploited for motif detection. However, only 12 putative regulatory motifs have been identified. Most *Pf *genes were shown to have 4–5 of these regulatory elements in their upstream regions. The hypothesis has been made that each gene is regulated by multiple factors, with different combinations of factors being used to obtain the variety of expression profiles observed in the *Pf *life-cycle. Recently, a new algorithm called Gene Enrichment Motif Searching (GEMS) has been developed which has the ability to identify high-confidence, *cis*-regulatory elements in genomes such as those of *Pf*, which have a skewed nucleotide composition and are rich in repeat sequences [21]. The algorithm has been applied to the promoter regions of genes occurring in 21 functionally related, co-expressed clusters, generated from *Pf *life-cycle microarray data by semi-supervised clustering; 34 putative *cis*-regulatory elements have been identified.

The asexual IDC transcriptome of *Pf *has previously been analysed and 13 functional groups of co-expressed genes have been listed [3]. In the present study, putative control elements in the upstream regions of each of these 13 sets of genes have been searched for. Three motif searching programs have been used for the purpose: MEME [22], Weeder [23,24] and AlignACE [25]. These programs identify statistically over-represented motifs in a given set of upstream sequences. A short motif that occurs more frequently in a given set of sequences than expected, based on its frequency of occurrence in the genome, is said to be over-represented. The rationale behind looking for over-represented motifs is that, in eukaryotes, transcription-related regulatory motifs are repeated many times in the upstream regions, and multiple copies of the motif are correlated with transcriptional activity [26,27]. Thus, methods developed for the *de novo *identification of regulatory motifs are based on the premise that statistically over-represented motifs are likely to be of biological significance. Studies correlating over-represented upstream motifs with gene expression levels, in *Saccharomyces cerevisiae*, have given validity to this assumption [28,29]. The over-represented motifs identified in this study would be putative regulatory elements and their actual significance would have to be experimentally determined.

The present study differs from the study by Young et al. in [21] in several ways. While, in the present study, over-represented motifs identified by 3 motif-discovery programs have been compared in order to identify reliable motifs, in [21], a new method (called GEMS) has been developed to identify regulatory motifs. The dataset of co-expressed gene clusters also differs in the 2 studies (13 clusters restricted to the *Pf *IDC versus 21 clusters covering the entire *Pf *life-cycle). In the present study, as a result of examining the promoter regions of gene clusters expressed in the 48-hour *Pf *IDC, it has been possible to identify combinations of putative regulatory motifs that might be controlling gene co-expression in each stage transition in the cycle. Also, in the present study, putative regulatory elements have been identified for organellar genes (which are encoded in the nuclear genome but whose products are transported to organelles such as the mitochondrion and apicoplast). Regulatory elements identified for the organellar genes have also been compared with those identified for the cytoplasmic genes. Other differences between the 2 studies are discussed below.

## Results

In the asexual IDC transcriptome of *Pf*, it has been shown that cellular processes take place in an orderly, programmed and continuous cascade, and that functionally related genes along this cascade show similar expression profiles [3]. Table S2, in [3], lists 13 groups of nuclear encoded genes, each group consisting of functionally related genes that share a common expression profile; the expression data or profile for genes in each group is also given. Pearson correlation values were calculated for all pairs of gene expression profiles in each group; the average value for each group is given in Table 1; the values range from 0.66 to 0.88, indicating good correlation between the expression profiles of genes in each group. Each group may, therefore, be looked upon as a set of co-expressed genes. The groups of genes being functionally related *and *co-expressed, the chances are greater that they are co-regulated; this makes it meaningful to search for regulatory motifs in the upstream regions of the groups of genes.

**Table 1:** Strong motif groups identified in the upstream regions of 13 sets of functionally related genes taken from reference [3] (cf., Methods).

^a^Functional group	^b^No. of genes	^c^Upstream regions < 2000 nt	^d^Average Pearson value	^e^Strong motif groups
***Ring/early trophozoite***				
Transcription machinery	22	15	0.76	G-rich
Cytoplasmic Translation Machinery	132	60	0.83	G-rich, C-rich, TGTG
Glycolytic pathway	11	2	0.70	G-rich
Ribonucleotide synthesis	16	11	0.66	G-rich
				
***Trophozoite/early schizont***				
Deoxynucleotide synthesis	6	1	0.88	no strong motif group identified
DNA replication machinery	32	15	0.81	TGTG, G-rich, CACA, C-rich
TCA cycle	8	3	0.67	G-rich
Proteasome	28	17	0.70	G-rich, CACA, TGTATG
Mitochondrial genes	15	12	0.72	C-rich, TGTG, G-rich
Organellar Translation machinery	33	27	0.74	C-rich, G-rich, TGTG
				
***Schizont***				
Merozoite invasion	55	22	0.75	TGCACA, TGTATG, C-rich, G-rich
Actin myosin motility	10	5	0.83	TGCACA, TGTG, G-rich
				
***Early ring***				
Early ring transcripts	24	2	0.71	no strong motif group identified

^a ^The groups of functionally related genes have been arranged in the order in which they are expressed in the *Pf *IDC.

^b ^The number of genes (or the number of upstream regions) in each functional group.

^c ^The number of upstream regions in the group that are < 2000 nt in length; for example, in the transcription machinery group, 15 upstream regions out of 22 are < 2000 nt in length.

^d ^The expression profile for each gene in each group has been given in reference [3]. Pearson correlation values were calculated for all pairs of profiles in each group. The average of all pairwise Pearson values in the group is given in the table.

^e ^Strong motif groups (constituted, usually, by motif-sets that are identified by multiple programs and that are related) identified in the upstream regions of each functional group.

In Table 1, the 13 groups of functionally related genes have been arranged in the order in which they have been presented in [3], which is the order in which they appear in the *Pf *IDC. The 48 hour *Pf *IDC consists of the morphological stages of ring, trophozoite, schizont and merozoite. Induction of large numbers of genes occurs in the ring to early trophozoite transition, in the trophozoite to early schizont transition, during the mid- and late-schizont stages, and during the early ring stage. The functional groups induced during these 4 transitions are listed in Table 1. The results of the present study have been discussed keeping this order of appearance of the functional groups in mind.

The aim of the study was to search the upstream regions of each set of functionally related, co-expressed genes, in the *Pf *IDC, for conserved, regulatory motifs which might be responsible for the co-ordinate expression of the set of genes. Upstream regions have been considered on only one DNA strand, the strand on which each gene was encoded; ≤2000 nt upstream of the translational start site (TLS) of each gene have been considered for motif searching (for more details, see Methods). All sequences used and motifs presented in this study are in the 5'- to 3'-orientation. In the literature, many methods have become available for the task of motif discovery. A comparative assessment of 13 of them has been carried out [30] and it has been recommended that multiple tools, rather than a single one, be used to search for motifs, because different tools, based on different algorithms, predict slightly different motifs, and, therefore, cover over-represented motif space better. It has also been recommended that the top few predicted motifs be considered, rather than the top most one alone. In the present study, therefore, 3 motif discovery programs, MEME, AlignACE and Weeder, have been used to search for over-represented motifs, and several of the top-scoring motifs have been considered. MEME and AlignACE were selected because they have been used in previous studies aimed at identifying regulatory sequences in the *Pf *genome [17-20]. The Weeder program was selected because, in the study in [30], it outperformed other programs by most of the measures used.

In order to check the sequences being used, and the computations, an attempt was made to retrieve the G-box regulatory element that had previously been identified [17]. The upstream regions (2000 nt upstream of the TLS) of the 18 *hsp *genes considered in the study, were extracted, and the AlignACE program, with previously reported parameters, was used for motif searching. The G-box motif was retrieved, and a comparison of the results obtained with those given in the paper showed that the sequences being used in this study, and the computations, were right.

### Results of motif searching in the upstream regions of 13 groups of co-expressed Pf genes

The 3 motif-discovery programs, MEME, Weeder and AlignACE, were used to search for over-represented motifs in the upstream regions of each of the 13 sets of functionally related, co-expressed *Pf *genes. A total of 179 motif-sets were identified by the 3 programs in the 13 sets of upstream regions, and there were a total of 3847 motif occurrences in these motif-sets (for definitions, cf., Methods). The 179 motif-sets were grouped together (as described under Methods) to form 57 motif groups, out of which 27 were regarded as strong motif groups (an example is given in Table 2), because they included related motif-sets that had been identified by more than one motif-discovery program (there were exceptions; cf., below and Methods). These 27 strong motif groups were formed by grouping together 129 out of 179 motif-sets, and included 3208 motif occurrences. Additional files 1, 2, 3 and 4 show the 57 motif groups identified in the 13 sets of upstream regions; strong *and *weak motif groups, and all motif-sets, motif occurrences and sequence logos are shown. Additional file 1 shows the motif groups identified for the 4 sets of genes expressed during the ring to early trophozoite transition (cf., Table 1), Additional file 2 shows the motif groups identified for the 6 sets of genes expressed during the trophozoite to early schizont transition, and, similarly, Additional files 3 and 4 show motif groups identified for the 2 and 1 sets of genes expressed during the schizont and early ring stages, respectively. The 27 strong motif groups are labeled as 'strong motifs' in the Figures.

**Table 2:** G-rich motif-sets identified by AlignACE, Weeder and MEME in the upstream regions of the group of 22 transcription machinery genes.

AlignACE			AlignACE		
	GGG--AAAW-WAWA			KGWGGGS	
PFB0715w;	GGGAAAAAACAAAA	122	PFB0290c;	TGGGGAG	150*
PFC0155c;	GGGAAAAAAAAAAA	398	PFE0465c;	TGTGGGG	32*
PFC0155c;	GGGTAAAAAAAAAA	1458	PFE0465c;	AGAGGGG	634*
PFF1390w;	GGGAAAAAAAAAAA	163*	PFF1390w;	TGTGGAC	122
PF11_0264;	GGGGGAAAAATAAA	588*	PFF1390w;	TGTAGGC	981
PF11_0445;	GGGTCACATTTATA	6*	PF07_0027;	TGTGGGG	81*
PF11_0445;	GGGTGAAAATAAAA	626	PF07_0027;	AGAGGGC	914*
PF11_0445;	GGGAAAAAAAAAAA	1147*	PF11_0264;	GGAGGGG	583*
PF14_0207;	GGGAAAAAATAATA	774*	PF11_0445;	TGTGGGG	2*
			PF11_0445;	TGTGGAG	42*
AlignACE			PF13_0023;	GGTGGAG	1579*
	G-GGGG-AAAAAWAAAAWWAWA		PF14_0207;	GGTTGGG	770*
PFC0155c;	GCGGGTCTACAAGAAAAATGAA	678			
PFE0465c;	GGGGTGTAATGATAAAAAGGGA	35*	Weeder		
PFE0465c;	GAGGGGAAAATAAAATAATAAA	635		TGGGGAGT	
PFF1390w;	GTGGGAAAAAAAAAAAAAAAAA	161*	PFB0290c;	TGGGGAGT	151*
PF07_0027;	GAGGGCTCAAAAAAAAAAAAAA	915*	PFE0465c;	TGGGGTGT	35*
PF11_0264;	GGGGGGGAAAAATAAAATAATA	586*	PF07_0027;	TGGGGAGT	84*
PF11_0445;	GTGGGGTCACATTTATATTGAA	3*	PF13_0150;	TAGGGAGT	1488
PF11_0445;	AAGGGGAAAAAAAAAAATAAAA	1144*			
PF13_0023;	GAGGGGGAAAGTATTAATTATT	1583*	MEME		
					AGTGGAAAAAA
AlignACE			PF13_0023;	1583	GGAGGGGGAAA*
	G-GGGG-S--A		PF11_0264;	587	GGGGGGGAAAA*
PF11_0445;	GTGGGGTCACA	3*	PFF1390w;	161	GGTGGGAAAAA
PF11_0264;	GAGGGGGGGAA	584*	PFE0465c;	635	AGAGGGGAAAA*
PFE0465c;	GTGGGGTGTAA	33*	PF13_0150;	863	GGTGGAAAAAA
PF07_0027;	GTGGGGAGTGA	82*	PF07_0027;	85	GGGGAGTGAAA*
PF07_0027;	GAGGGCTCAAA	915*	PFL0330c;	1146	AGAGGAAGAAA
PF11_0445;	GTGGAGACTTA	43*	PF11_0445;	1146	AGGGGAAAAAA
PF13_0023;	GTGGAGGGGGA	1580*	PF14_0469;	646	AGTGAGAGAAA
PFF1390w;	GTGTGGACCAA	121*	PF14_0207;	771	GGTTGGGAAAA*
PFB0290c;	TTGGGGAGTTA	149*	PFC0805w;	254	AGTGGAAAAAA
			PFA0505c;	106	AGAGGAAAAAA
AlignACE			PF10_0269;	368	GGAGAAAAAAA
	GGGGRGKGR-A		PFB0715w;	1202	AGAGAAGAAAA
PF13_0023;	GGAGGGGGAAA	1582*	PF11_0358;	312	GAAGGAAGACA
PF11_0264;	GGAGGGGGGGA	583*	PFC0155c;	396	AATGGGAAAAA
PF07_0027;	GGGGAGTGAAA	84*	PFL0665c;	484	AGTGAAGACAA
PFE0465c;	GTGGGGTGTAA	33*	PF13_0341;	8	AGCGAAAAAAA
PFB0290c;	GGGGAGTTATA	151*	PF14_0241;	511	CATGGAAGAAA
PF11_0445;	GTGGGGTCACA	3*	PFI1130c;	767	GCTGAGTAAAA

The motif-sets are related and together constitute a strong motif group.

**Figure 1:**
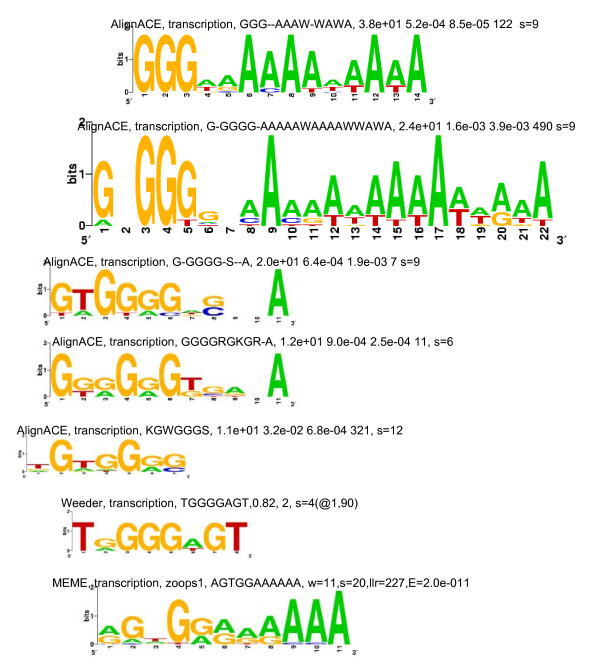
**Sequence logos for the G-rich motif-sets given in Table **2** and identified in the upstream regions of the set of 22 transcription machinery genes**. The logos have been manually aligned so that equivalent nucleotide positions, in the motif-sets used to generate them, lie approximately one below the other. The caption for each logo indicates the program that identified the motif-set, the group of genes whose upstream regions are being considered, the consensus motif for the motif-set and information about the motif-set output by the program. For the AlignACE logos, the caption gives the MAP (maximum *a priori *log likelihood [25]), group specificity and positional specificity scores, the preferred position at which the motifs occur and the number of motif occurrences in the set of upstream regions. For the Weeder logo, the caption gives a significance score, the number of 'redundant' consensus motifs related to the given one and the number of motif occurrences in the set of upstream regions when 1 mutation and a match percentage ≥ 90% are allowed. For the MEME logo, the caption gives the mode in which the program has been run, the width of the motif, the number of motif occurrences or sites in the set of upstream regions, the log likelihood ratio and the expectation value. Similar information is provided for the logos in Figures 2, 3 and 4.

Given, for each motif in a set, are: the gene name, the motif that was identified in the upstream region of the gene, the position of the motif in the upstream region, and, sometimes, an *, which indicates that the motif overlaps with motif occurrences in one or more of the other motif-sets; at the top of each motif-set, a consensus sequence for the set is given; each motif-set has been used to obtain a sequence logo which is shown in Figure 1; all motifs in the table occur on the forward strand.

**Figure 2:**
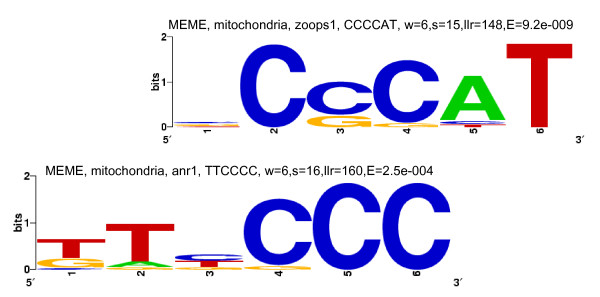
**Sequence logos for C-rich motif-sets identified by MEME in the upstream regions of the set of 15 mitochondrial genes**. Motif-sets are given in Additional file 2:37 and logo captions are explained in the legend to Figure 1.

**Figure 3:**
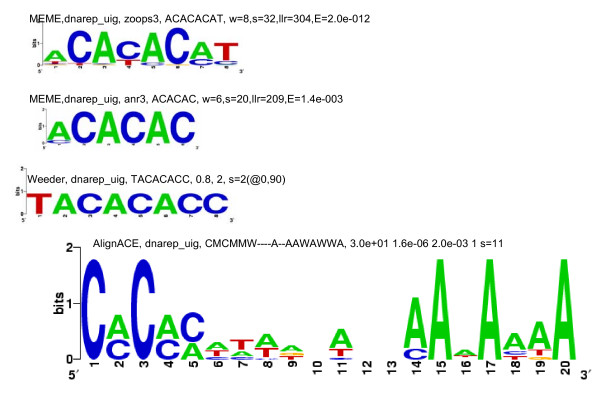
**Sequence logos for CACA motif-sets identified in the upstream regions of the DNA replication machinery set of genes**. Motif-sets are given in Additional file 2:12 and logo captions are explained in the legend to Figure 1.

**Figure 4:**
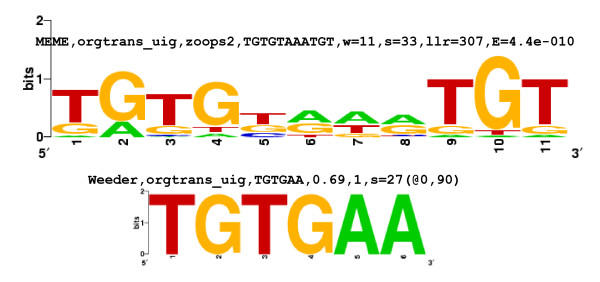
**Sequence logos for TGTG motif-sets identified in the upstream regions of the organellar translation machinery group of genes**. Motif-sets are given in Additional file 2:48 and logo captions are explained in the legend to Figure 1.

An examination of the 27 strong motif groups showed that there were only 4 unique motifs, and that none, some or all 4 of them appeared as over-represented motifs in the upstream regions of the 13 sets of genes. The 4 motifs have been referred to, in the present study, as the G-rich motif, the C-rich motif, the CACA motif and the TGTG motif; examples of these motifs are shown in Figures 1, 2, 3 and 4, respectively. The G-rich motif has a core sequence consisting of 3 to 4 Gs (GGG, GGGG), the C-rich motif has a core sequence consisting of 3 to 4 Cs (CCC, CCCC), the CACA motif has the CACA core sequence and the TGTG motif has a core sequence of more than one TG pair (TGTG, TGTATG). The motif groups (or, simply, motifs) occurring in the upstream regions of each of the 13 sets of genes are given in Table 1.

Three of the four motifs (all, except the C-rich motif) have previously been identified [20]. The C-rich motif has been identified as an over-represented motif by the MEME program alone, for the set of mitochondrial genes (Figure 2); it has, nevertheless, been regarded as a strong motif because it occurs in most of the genes in the set. The G-rich motif has previously been shown to be a regulatory element (called the G-box or the TG-box) for the *Pf *hsp genes [17,18]. In the present study, G-rich motifs have been identified in the upstream regions of several sets of genes, other than the *hsp *group, suggesting that they are not limited to the latter group. Interestingly, in eukaryotes, *cis*-regulatory elements, called G-strings, consisting of runs of only Gs on one strand and only Cs on the other, have been found to appear frequently in the upstream regions of genes that do not contain TATA boxes, initiator elements or CCAAT boxes [31]. *Pf *upstream regions, which often lack these latter elements, are thus ideally suited to host G-strings or G-rich motifs. The CACA motif has previously been identified in a putative TFBM [18]. In the present study, it is conspicuously absent in the upstream regions of functional groups expressed during the ring to early trophozoite transition (Table 1). The CACA and TGTG motifs identified, in this study, are not microsatellites. While in mammalian genomes, [CA]n repeats occur as microsatellites, in *Pf*, [CA]n and [TG]n repeats do not do so; instead, [TA]n, [T]n and [TAA]n repeats occur as microsatellites [32]. If at the start of the study, one had hoped to find one unique upstream motif, per co-expressed, functional group, that would explain why genes in the group were concomitantly turned on, one was certainly disappointed. Instead, a total of only 4 putative regulatory motifs were identified in the 13 sets, and 0 to all 4 of these motifs were found to appear in the upstream regions of each set. This is in consonance with the suggestion made previously [20] that, while *Pf *may have a small number of regulators and regulatory sequences, as compared to other eukaryotes, it uses them in a combinatorial way to bring about the required level of diversity in gene expression. It is also closer to the situation where a unique motif or a unique combination of motifs *per transition*, would explain why sets of genes specific to that transition were concomitantly turned on.

In order to check if the strong motifs identified in Table 1 were significant, statistical validation of the motifs was carried out, as described under Methods. The upstream sequences of each set of genes were shuffled 10 times to obtain 10 sets of shuffled sequences, and motif-searching by all 3 programs was carried out on all 10 sets. MEME, in 200 runs, did not identify any over-represented motif, in any set of shuffled sequences, obtained from any set of upstream sequences. Thus, over-represented motifs identified by MEME in the original sets of upstream sequences were significant because they were not picked in any of the randomized sequences. AlignACE identified no over-represented motifs in 128 out of 140 runs. In the remaining 12 runs, 13 motifs were identified. All motifs, however, were random ones because their MAP scores were poor (< 3.5; all motifs presented in the study have MAP scores > 10). Weeder runs (100) did not identify the motifs presented in this study. Considering that motifs presented in the study were each usually identified by multiple programs, and that they were not identified in the randomized sequences, one can conclude with confidence that the motifs are significant.

The *Pf *genome is the most AT-rich (or GC-poor) genome to have been sequenced to date; it has an overall GC-content of 19.4%, with the GC-content of exons being higher, at 23.7%, and that of intergenic regions being lower, at 13.6% [33]. Given the low overall GC-content, one would expect that the usage of GC in the genome would not be wasteful, and that G-C containing sequences would primarily be restricted to critical regions. Consistent with this, exons have a significantly higher GC-content than intergenic regions. In the upstream regions of genes (which are intergenic regions), if GC-containing motifs are over-represented, it would be reasonable to expect that such motifs are important and, perhaps, have a regulatory role, because GC is sparingly used in the genome. In the present study, therefore, greater significance has been attached to over-represented, GC-containing motifs. Over-represented AT-rich motifs (e.g., ATATAT, AAAAAA, TTTTTT, etc.) have largely been ignored because, owing to the high AT-content of the genome, these motifs were not likely to be unique to particular sets of co-expressed genes.

The CACA and TGTG motifs (and likewise, G-rich and C-rich motifs), identified in the present study, although they are partly complementary in sequence, have been regarded as 2 different motifs, and not as the same motif occurring on opposite strands. This is because, motif-searching was carried out on single-stranded upstream sequences (Methods), and the TGTG and CACA motifs that were picked were *not *from complementary strands. Further, when a regulatory motif is not palindromic (motifs in this study are not palindromic), the cognate protein is likely to bind in a different orientation (relative to the TSS) to, for example, a CACA motif on the coding strand, as compared to a CACA motif on the non-coding strand. Moreover, motif-searching programs, too, when run in single or double-stranded modes, report complementary motifs as separate motifs. Thus, TGTG and CACA motifs (and likewise, G-rich and C-rich motifs) deserved to be treated as distinct motifs because distinct TFs were likely to recognize them.

### Positional conservation of motif occurrences in the upstream regions

**Figure 5:**
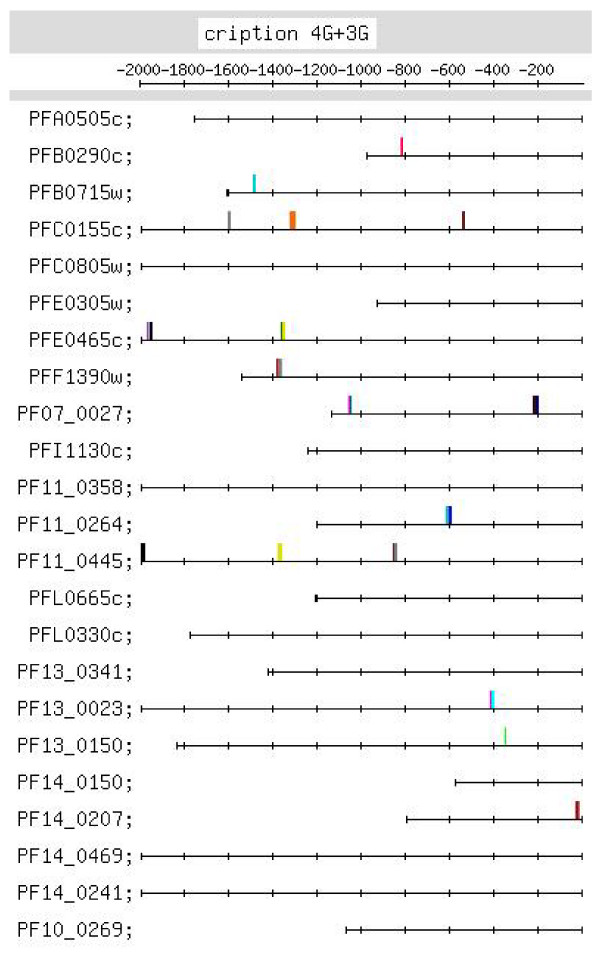
**Feature map marking the locations of 4G- and 3G-containing motif occurrences, given in Table **2**, in the upstream regions of the transcription machinery set of genes**. In Figures 5–11, gene names are indicated on the left, the upstream region of each gene is represented as a horizontal line marked from -1 to -2000 in steps of -200, where the TLS of the gene is located at 0 (not shown). The location of each motif in the upstream region is marked by a vertical line. The sequence and position of motifs in each figure are given in Additional file 6.

**Figure 6:**
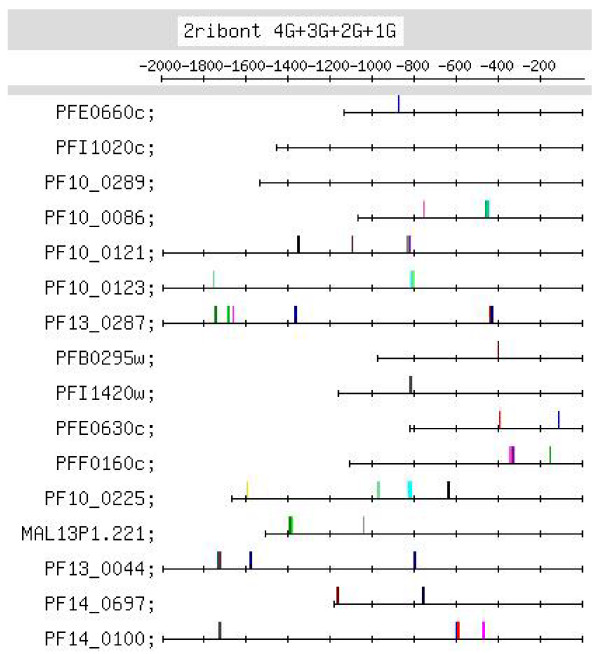
**Feature map marking the locations of 4G-, 3G-, 2G- and 1G-containing motif occurrences in the G-rich motif group identified in the upstream regions of the ribonucleotide synthesis set of genes (motif group shown in Additional file **1**:36)**.

**Figure 7:**
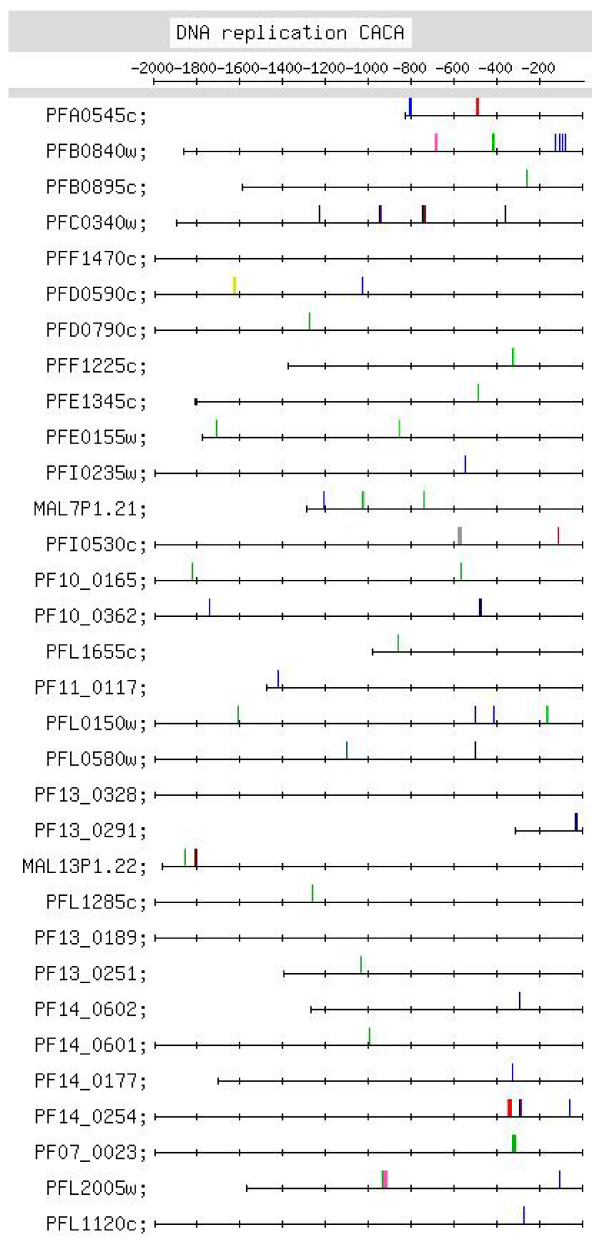
**Feature map marking the locations of CACA containing motif occurrences in the CACA motif group identified in the upstream regions of the DNA replication machinery set of genes (motif group shown in Additional file **2**:12)**.

**Figure 8:**
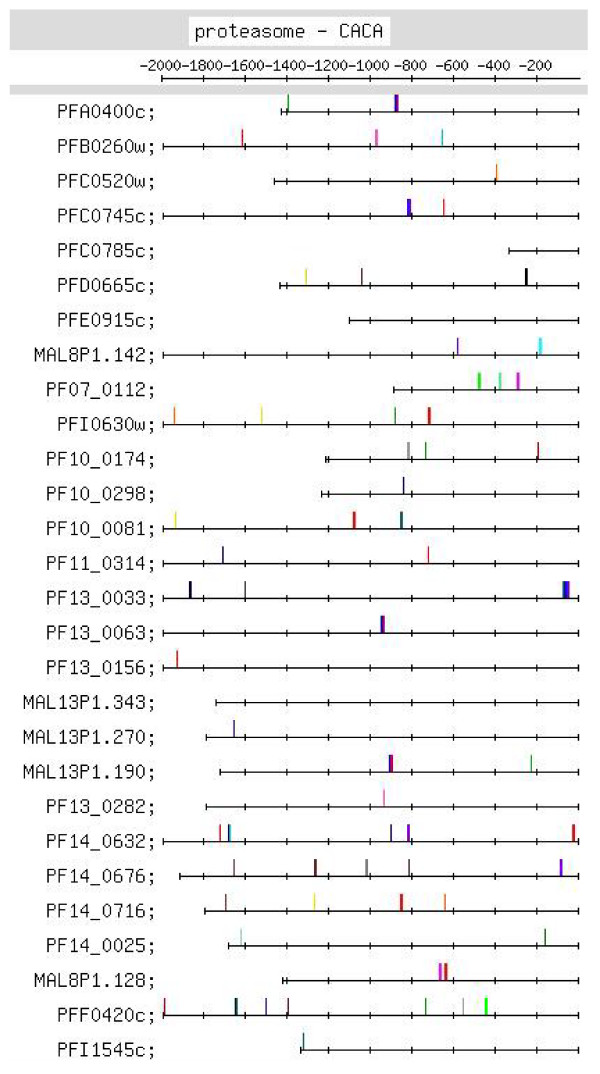
**Feature map marking the locations of CACA containing motif occurrences in the CACA motif group identified by MEME in the upstream regions of the proteasome set of genes (motif group shown in Additional file **2**:29)**.

**Figure 9:**
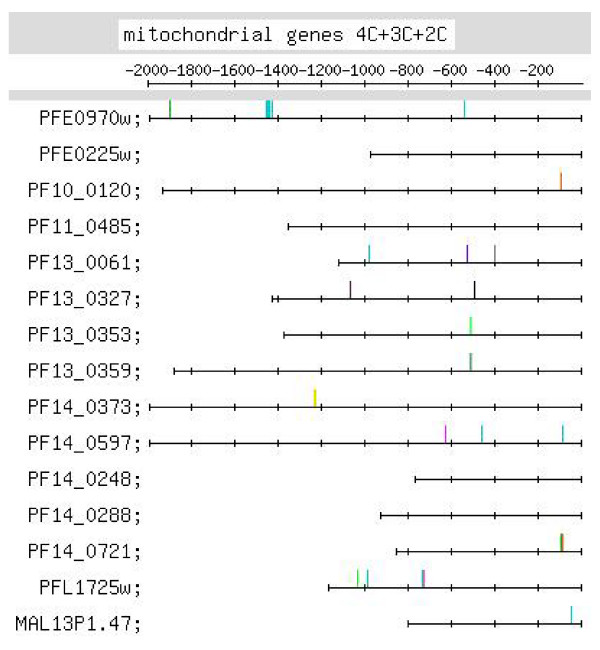
**Feature map marking the locations of 4C-, 3C- and 2C-containing motif occurrences in the C-rich motif group identified by MEME in the upstream regions of the set of mitochondrial genes (motif group shown in Additional file **2**:37)**.

**Figure 10:**
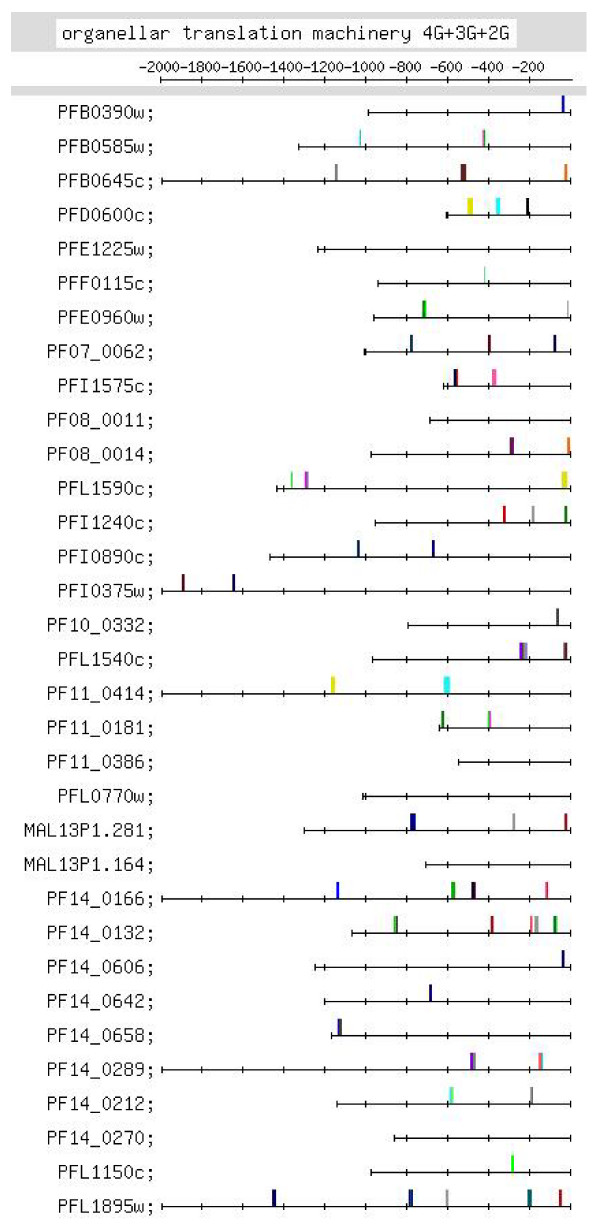
**Feature map marking the locations of 4G-, 3G- and 2G-containing motif occurrences in the G-rich motif group identified in the upstream regions of the organellar translation machinery set of genes (motif group shown in Additional file **2**:45, 46)**.

**Figure 11:**
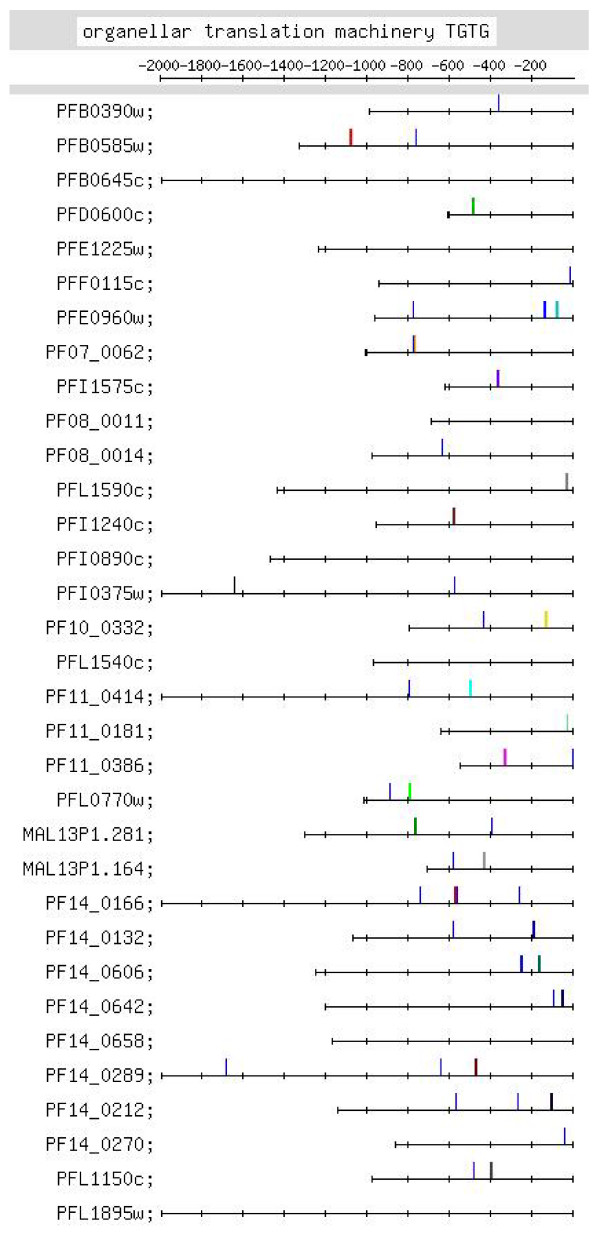
**Feature map marking the locations of TGTG containing motif occurrences in the TGTG motif group identified in the upstream regions of the organellar translation machinery set of genes (motif group shown in Additional file **2**:48)**.

The motif occurrences in the 27 strong motif groups, identified in the upstream regions of the 13 sets of genes, were sorted [see Additional files 5 and 6] and then visualized using feature maps (Additional file 7 and Figures 5, 6, 7, 8, 9, 10 and 11; cf., Methods). An interesting feature observed in some of the feature maps was that, in subsets of gene upstream regions, there was a preferential positioning of motifs relative to the TLS (examples discussed below); i.e., motifs show a preference to occur at a particular upstream position relative to the start of translation. In the literature, this has been referred to as 'positional bias', and TFBMs are known to display such a bias or preference [25]. In [21], 'positional enrichment' of motifs relative to the start codon has been noted for some clusters of *Pf *genes (sexual development, cytosolic ribosome and antigenic variation clusters), and the enrichment has been regarded as an indication that the motif is functionally important.

In the present study, positional bias or enrichment of a motif, relative to the TLS, has been referred to as 'positional conservation', and the latter has been regarded as an indication that the motif is important and, perhaps, plays a regulatory role. Putative regulatory motifs identified in the upstream sequences of each of the 13 sets of genes have been listed in Additional file 6. To identify sets of motifs which show positional conservation, the upstream region (extending from -2000 to -1) has been divided into windows of width 100 nt and the number of motifs occurring in each window, and also the number of sequences showing motifs in the window, have been counted from Additional file 6. Positional conservation seen in a window has been considered to be statistically significant if the probability of obtaining the observed (or higher) number of motifs, and the observed (or higher) number of sequences with motifs, in that window, by chance (as observed by 20,000 Monte Carlo simulations), was less than 0.05 (Methods). Thus, positionally conserved motifs presented in the study have been statistically validated. Further, instances of positional conservation have been discussed only when 4 or more motifs, and 4 or more sequences with motifs, occurred in a window. Details for the statistically validated, positionally conserved motifs are given in Additional file 8, and the motifs are highlighted in color in Additional file 6.

An attempt has also been made to examine if some of the positionally conserved motifs play a regulatory role at the transcriptional (DNA) or at the post-transcriptional (RNA) level. This has been done by retrieving the ESTs for the upstream sequences of the sets of genes, and searching for the putative regulatory motifs in the ESTs [details given in Additional file 9]. As ESTs contain extensions to the 5' end of the target mRNA, one can try to search for regulatory motifs in these additional sequence stretches. The presence of regulatory motifs occurring at or upstream of ~-300, in the ESTs of several upstream sequences of a set of genes, at the appropriate position with respect to the EST start codon, has been taken to suggest that the motifs may be playing a regulatory role at the RNA level (results discussed below). Further, regulatory motifs identified for some sets of *Pf *genes have also been searched for in the upstream sequences of orthologous genes from other *Plasmodium *species (results discussed below and details given in Additional file 9). The appearance of positionally conserved *Pf *motifs in the upstream sequences of 'in genera' orthologs gives considerable validity to the identified putative regulatory motifs.

Interesting observations made for the 13 sets of upstream regions are described below. The strong motifs identified for the 13 sets, and the positional conservation information for the motifs, are summarized in Additional file 10. This file also indicates the relevant Figures, Tables and Additional files that the reader may turn to, while following the discussion below. While referring to Additional files, the slide number has been mentioned; thus, AF-1:9 refers to the 9^th ^slide in Additional file 1. Negative numbers have been used to indicate motif positions; -400, for example, refers to a position 400 nt upstream from the TLS.

#### (i) Transcription machinery genes

A G-rich motif group has been identified as a strong motif for this set of genes [Additional file 10]. The majority of 4G and 3G motifs occur upstream of -400.

#### (ii) Cytoplasmic translation machinery genes

This is a large functional group consisting of 132 genes. G-rich, C-rich and TGTG motif groups have been identified as strong motif groups for this set of genes; all 3 motifs show positional conservation [Additional file 10]. The majority of 4G and 3G motifs occur upstream of -400, suggesting that these motifs tend to occur away from the TLS. 4C motifs also tend to occur upstream of -400. TGTG motif occurrences are small in number. In [21], an upstream CCCTTA motif was identified for a cytosolic ribosome cluster (89 genes), and the motif was found to be positionally enriched between -700 and -999. This motif has, however, been regarded as the approximate reverse complement of the G-box motif (ATGGGGC) [17]. 35 genes are common between the cluster in [21] and the present cluster. Here, C-rich *and *G-rich motifs have been reported; positionally conserved 4C motifs have been observed at ~-900.

#### (iii) Glycolytic pathway

A G-rich motif was identified as a strong motif for this set, but no positional conservation was observed [Additional file 10]. The latter was surprising because genes in the set are tightly coregulated [3]. This set of genes is thought to be *post*-transcriptionally regulated because a delay is observed between mRNA accumulation and protein expression [16]. Therefore, regulatory motifs at the transcriptional level may be less important.

#### (iv)Ribonucleotide synthesis genes

A G-rich motif appears as a strong motif in the upstream regions of this set (Figure 6). Distinct positional conservation is observed at ~-800, where 2 4G, 2 3G, 3 2G and 1 1G motifs occur [see Additional file 6]. Positional conservation is also observed at ~-1300, -1700 (4, 6 occurrences, respectively) and at ~-400 (6 occurrences; interesting but statistically weakly significant, p < 0.22). All 4G motifs, and all except 1 3G motifs occur upstream of -400. Thus, distinctly positionally conserved G-rich motifs are observed for this set of genes. The G-rich motifs were also searched for in the ESTs retrieved for the upstream sequences of this set of genes. Motifs occurring at -400 or -800, with respect to the EST start codon, were not observed in any of the ESTs [details given in Additional file 9]. This would suggest that the motif plays a regulatory role at the transcriptional (DNA) level, and not at the post-transcriptional (RNA) level. The G-rich motifs [listed in Additional file 6] were also searched for in the upstream sequences of orthologs of this set of genes in other *Plasmodium *species. Oddly, the G-rich motifs occurred infrequently in the upstream sequences of the orthologous genes [Additional file 9]. This would suggest that the motifs are unique to the upstream sequences of this set of genes in *Pf*.

#### (v) Deoxynucleotide synthesis

No strong upstream motif was identified for this set, even though it is a tightly co-expressed set (Pearson correlation value, 0.88; Table 1).

#### (vi) DNA replication machinery

4 strong motifs were identified for this set of genes – the CACA, the TGTG, the G-rich, and the (less important) C-rich motifs [see Additional file 10]. The CACA motif (Figure 3) shows positional conservation at ~-300 (8 occurrences) and at ~-500 and -1000 [5, 5 occurrences, respectively; Figure 7, Additional file 6]. The latter 2 sets of motifs are interesting but statistically weakly significant, p < 0.10 and p < 0.15, respectively. CACA motifs were searched for in the ESTs retrieved for the upstream sequences of this set of genes [details given in Additional file 9]. In the ESTs for 6 upstream sequences, upstream CACA motifs (occurring at -118, -34, -111, -264, ~-300 and -489) were identified. Thus, in the ESTs of 3 of these upstream sequences, positionally conserved upstream motifs (occurring at -264, ~-300 and -489) were observed. Owing to this latter observation, one has to admit the possibility that the CACA motif plays a regulatory role at the RNA or post-transcriptional level. The CACA motifs were also searched for in the upstream sequences of orthologs of this set of genes in other *Plasmodium *species. The motif is clearly observed in the upstream sequences, occurring, more frequently, between -1 and -600 [feature map in Additional file 9]. The occurrence of the motif in the upstream sequences of 'in genera' orthologs gives considerable validity to the motif being a regulatory motif. TGTG motifs occur between -1 and -800 and between -1000 and -1700. The G-rich motif includes a significant number of 2G and 1G (e.g., GAGAGA) motif occurrences [see Additional file 5]; in comparison, 4G and 3G occurrences are fewer, and all of them, except 1 4G and 1 3G motifs, occur upstream of -400. In [21], for the cluster of 100 DNA replication and chromosome cycle genes (only 12 of which are common to the present cluster of 32 genes), an upstream NTGTGTGA motif was identified. It has been pointed out that this motif is observed for a large group of genes expressed during the middle to later stages of the IDC and that it may play a role in the regulation of the group. In agreement with this, in the present study, the TGTG motif was observed in most groups of genes expressed during the trophozoite/early schizont and schizont stages (Table 1). In addition to the TGTG motif, an interesting CACA motif has been identified for this set of genes, which may be functioning at the RNA level.

#### (vii) TCA cycle

A G-rich motif was identified as a strong motif for this set of genes [Additional file 10]. 4G, 3G and 2G motifs did not show positional conservation.

#### (viii) Proteasome

The strong motifs identified for this set were the CACA, the G-rich and the TGTG motifs [Additional file 10]. The CACA motif showed distinct positional conservation between ~-800 and -1000 (15 occurrences) and between -1600 and -1700 (10 occurrences) [Figure 8, Additional file 6]. While the CACA motifs in the upstream regions of the DNA replication machinery genes occur closer to the TLS (-200 to -600 and -1000; Figure 7), the CACA motifs for this set of genes occur further upstream from the TLS (~-900 and -1700). CACA motifs were searched for in the ESTs retrieved for the upstream sequences of this set of genes [Additional file 9]. In the ESTs for 3 proteasome upstream sequences, motifs matching upstream CACA motifs (occurring at -190, -199 and -232) were observed. In none of the ESTs, however, were positionally conserved CACA motifs occurring at ~-900 identified. This would suggest a regulatory role for the motif at the transcriptional (DNA) level. The CACA motifs [listed in Additional file 6] were also searched for in the upstream sequences of 'in genera' orthologs of this set of genes. Motifs were observed between -1 and ~-1200 in many sequences, and further upstream in others; some positional conservation was also observed [Additional file 9]. The G-rich motifs – 4G+3G – show some positional conservation. The TGTG motif occurs as the variant, TGTATG. In [21], for the cluster of 75 proteasome complex genes (15 of which are common to the present cluster of 28 genes), no upstream motif was identified. In the present study, 3 motifs were identified.

#### (ix) Mitochondrial genes

This set of genes, encoded on the nuclear genome, generates protein products that have to travel to the mitochondrion of the parasite. The genes have to be expressed together, in order that their protein products may arrive together at the mitochondrion. One might, therefore, expect to find regulatory motifs which coordinate the temporal co-expression of this set of genes. 3 strong motifs have been identified for the set – the C-rich, the G-rich and the TGTG motifs [Additional file 10]. The C-rich motif (Figure 2) was identified by MEME, alone. Figure 9, which shows the locations of 4C+3C+2C motifs, shows that there is distinct positional conservation of motifs at ~-500 (7 occurrences – 1 4C, 5 3C and 1 2C) and at ~-100 (4 occurrences – 3 4C and 1 3C; statistically weakly significant, p < 0.08). The striking positional conservation, together with the fact that the number of motif occurrences is limited to at most 3 per gene, suggests that the motif may have a significant regulatory role. The C-rich motifs were further searched for in the 244 ESTs retrieved for the upstream sequences of this set of genes [Additional file 9]. In a single EST for one upstream sequence, a positionally conserved upstream motif (occurring at -497) was observed. More evidence than this would be needed to suggest that the C-rich motif plays a regulatory role at the post-transcriptional (RNA) rather than the transcriptional (DNA) level. The C-rich motifs, listed in Additional file 6, were searched for in the upstream sequences of 'in genera' orthologs of this set of genes. Impressively, motifs were observed between -400 and -600; they were also observed further upstream and between -200 and -400 [map in Additional file 9]. The occurrence of the motif in orthologous upstream sequences provides considerable support for the motif as a regulatory motif. The G-rich motif – 4G+3G+2G+1G – showed positional conservation between ~-200 and -300 (6 occurrences). Out of 3 4G motifs, one occurs at -107 and one occurs at -226. TGTG motifs occurred between -200 and -450 (8 occurrences). The 3 motifs, individually or together, appear to play a regulatory role, because most of the occurrences of all 3 motifs lie between -200 and -600 and are suitably positioned to function as TFBMs.

#### (x) Organellar translation machinery

This set of genes codes for components of the apicoplast and/or mitochondrial translation machinery. The genes are nuclear encoded but the proteins travel to the apicoplast or mitochondrion. Strong motifs identified for the set were the G-rich, C-rich and TGTG motifs [Additional file 10]. Figure 10 shows that G-rich motifs are quite numerous: there are 11 4G, 29 3G and 27 2G motifs. Positional conservation is observed close to the TLS, at ~-30 [13 occurrences – 2 4G, 6 3G, 5 2G; Additional file 6]. While in most other groups of genes, 4G and (to a lesser extent) 3G motifs usually occur upstream of -400 (e.g., Figure 6), in this group, 4G and 3G motifs frequently occur between -1 and -400. Thus, the number and location of G-rich motifs is uncharacteristic in this set of genes. TGTG motifs also show positional conservation [Figures 4, 11, Additional file 10]. C-rich motifs, in this set, in comparison with the mitochondrial genes, are more numerous (16 4C, 26 3C and 24 2C motifs), but do show positional conservation at ~-500. This set of genes shows homology to components of the cytoplasmic translation machinery [3]. Interestingly, the strong motifs identified for both sets of genes are the same (G-rich, C-rich and TGTG motifs); motif occurrences in the upstream regions of the 2 sets, however, show differences. In the organellar translation machinery set, G-rich motifs occur closer to the TLS, while in the cytoplasmic translation machinery set, they occur further upstream [Figure 10 and Additional file 7:5–7]; in the former, TGTG motifs are more numerous and show positional conservation, while in the latter, occurrences of the motif are far fewer [Figure 11 and Additional file 7:13]; TGTG and C-rich motifs tend to occur further upstream in the latter as compared to the former; finally, in the former, upstream regions are shorter as compared to the latter. The differences may be regulatory cues that ensure that the 2 sets of genes are turned on at different time points in the IDC. They may also be a reflection of the prokaryotic and eukaryotic origins of the 2 sets of genes.

G-rich and TGTG motifs were searched for in the 339 ESTs retrieved for the upstream sequences of this set of genes [Additional file 9]. Considering that many G-rich motifs occur close to the TLS, and that motifs occurring close to the TLS (rather than those occurring further upstream) are more likely to be transcribed into the ESTs, it was surprising to find only a small number of upstream motifs in the ESTs. For the upstream sequence, PF14_0132, 3 interesting ESTs were found; each EST had the start codon and 3 upstream G-rich motifs (occurring at -83, -176 and -200). Altogether, in the ESTs of only 3 and 5 upstream sequences, out of 33, respectively, were upstream G-rich and TGTG motifs found, respectively. Only upstream motifs occurring close to the TLS (between -200 and the TLS) were observed in the ESTs. More evidence would be needed to suggest that the G-rich and TGTG motifs observed for this set of genes play a regulatory role at the post-transcriptional level. The G-rich and TGTG motifs [listed in Additional file 6] were also searched for in the upstream sequences of 'in genera' orthologs of this set of genes. In contrast to the considerable number of G-rich motifs observed in the *Pf *upstream sequences, few motifs were observed in the orthologous upstream sequences; an indication, perhaps, that the G-rich motifs were unique to the *Pf *upstream sequences. TGTG motifs were observed in the orthologous upstream sequences. They occurred between -200 and -800 where positionally conserved *Pf *upstream motifs also occur [Additional file 9].

#### (xi) Merozoite invasion genes

4 strong motifs – TGCACA (CACA motif preceded by TG), TGTG, C-rich, G-rich – were identified for this set [Additional file 10]. The four motifs do not show striking patterns of positional conservation. G-rich motifs tend to occur upstream of -400.

#### (xii) Actin myosin motors

This is a tightly co-expressed group of genes (Pearson correlation value, 0.83; Table 1). Strong motifs identified for this group were the TGCACA motif, the G-rich motif and the TGTG motif [Additional file 10]. The motifs did not show distinct patterns of positional conservation. Most 4G and 3G motifs occurred upstream of -400; several TGTG motifs occurred between -100 and -400.

#### (xiii) Early ring transcript genes

Strong motifs were not identified for this set, perhaps, because the genes are post-transcriptionally regulated [16].

## Discussion

The study by Young et al. [21] and the present study have both used computational methods to identify putative regulatory motifs in the upstream regions of co-expressed genes in the *Pf *life-cycle. The co-expressed gene clusters for which regulatory motifs have been identified, however, *differ *in the 2 studies (Introduction). For the cytoplasmic translation machinery, DNA replication machinery and proteasome clusters, however, there is some overlap of genes in the 2 studies. In the present study, for each of the first 2 of these clusters, several putative regulatory motifs have been identified, including a motif similar to the one in [21]; for the proteasome cluster, 3 motifs were identified in the present study (Results), while none were identified in [21].

The search, in the present study, for over-represented motifs in the upstream regions of 13 functional groups of co-expressed genes, in the 48-hour *Pf *IDC, yielded several interesting results. 4 main motifs were identified by the methods used, and they have been referred to as the G-rich, the C-rich, the CACA and the TGTG motifs. Zero to all four of these motifs occur in the upstream regions of each of the 13 sets of genes. Motifs, similar to these 4, have previously been identified for different sets of genes [17-21]. In this study, *combinations *of putative regulatory motifs have been identified for co-expressed sets of genes in the stage transitions of the 48-hour *Pf *IDC. It has previously been suggested that, rather than a large variety of *cis*-regulatory motifs, different combinations of a small number of motifs regulate gene expression in *Pf *[20]. It is further suggested here that the combination of regulatory motifs tends to be more similar for functional groups of genes expressed in the same transition (Table 1). An examination of the occurrences of each motif in the upstream regions of the relevant group of genes showed that, for some motifs, the occurrences were positionally conserved in subsets of genes; i.e., in subsets of genes, motifs occurred at similar upstream positions with respect to the TLS, even when the genes in the subset were present on different chromosomes. The observed positional conservation enhances the significance of the over-represented motifs identified by the motif discovery programs, and suggests that the motifs may have a regulatory role. In [21], 'positional enrichment' of motifs relative to the start codon has been noted for some clusters of *Pf *genes. In the present study, the observed positional conservation has been shown to be statistically significant.

Additional file 6 lists putative regulatory motifs (including positionally conserved ones) discussed in the text. The table would be useful to experimentalists looking for transcription regulatory elements in the *Pf *genome. Some striking examples of positional conservation were observed. In the ribonucleotide synthesis group, distinct positional conservation of G-rich motifs was observed, with the motifs almost occurring in columns (Figure 6). In the set of mitochondrial genes, the C-rich motif occurs with striking positional conservation at -500 (Figure 9). In the proteasome set of genes, the CACA motif shows striking positional conservation at ~-900 and -1700 (Figure 8); on the other hand, in the DNA replication machinery genes, the CACA motif occurs closer to the TLS (-200 to -600 and at ~-1000; Figure 7). In all functional groups, except the organellar translation machinery group, 4G- and (to a lesser extent) 3G-containing G-rich motifs tended to occur upstream of -400; in the organellar translation machinery group, 4G and 3G motifs frequently occurred between -1 and -400 (Figure 10); thus, the position of G-rich motifs is unusual in this set of genes. The organellar translation machinery and mitochondrial genes are nuclear encoded but are of prokaryotic origin; their protein products are slated for and function in the mitochondrion and apicoplast, organelles of prokaryotic origin. The C-rich motif occurring upstream of the mitochondrial genes, and the uncharacteristically positioned G-rich motifs occurring upstream of the organellar translation machinery genes, perhaps, help to ensure that these genes are expressed in a timely fashion, in order that their protein products may travel to their destination, outside the nucleus.

As separate cytoplasmic and organellar translation machinery gene co-expression clusters, occurring at different time points in the *Pf *IDC, are observed (Table 1), it has been possible, in the present study, to make a comparison of the putative regulatory motifs in the upstream regions of these 2 sets of genes. The upstream regions tend to be longer in the former as compared to the latter. The G-rich, the TGTG and the C-rich motifs were identified as over-represented for both sets of genes (Table 1). The locations of the motifs, however, differ in the 2 sets of genes. G-rich motifs in the former usually occur upstream of -400, while in the latter they frequently occur between -1 and -400; TGTG motifs are not numerous in the former but are numerous in the latter; TGTG and C-rich motifs tend to occur further upstream in the former as compared to the latter. Thus, presumably, the control mechanisms differ for the 2 sets of genes, even though the latter set shows homology to the former [3]. This is necessary, too, because the 2 sets of genes are expressed at different time points in the *Pf *IDC (Table 1).

In [21], the G-box motif [17] has been regarded as the complement of the CCCCTTA motif and it has been suggested that the motif may be associated with highly expressed trophozoite-specific metabolic genes. Further, the NTGTGTGA motif has been observed for a large group of genes expressed during the middle to later stages of the IDC [21]. In the present study, too, TGTG, C-rich *and *G-rich motifs are observed for many clusters of genes in the IDC (G-rich and C-rich motifs have been regarded as separate motifs; cf., Results). However, the combination of motifs is more similar for clusters in a transition (Table 1). Further, the CACA motif appears *not *to be over-represented only in the upstream regions of genes expressed in the ring to early trophozoite transition. The presence of the motif may ensure that genes with the motif are *not *turned on during this transition. The motif is over-represented in the upstream regions of sets of genes expressed in the trophozoite to early schizont transition (except the mitochondrial and organellar translation machinery sets of genes, which are unique because their protein products are headed outside the nucleus), and also in the schizont stage, where modified CACA motifs (TGCACA motifs) are over-represented.

An attempt has been made to examine if some of the positionally conserved motifs might be playing a regulatory role at the post-transcriptional level. Putative regulatory motifs were searched for in the ESTs retrieved for the upstream sequences of sets of genes. Upstream G-rich motifs (at -400 and -800) and CACA motifs (at ~-900) identified for the ribonucleotide synthesis and proteasome sets of genes, respectively, were not found in the ESTs; this would suggest that these motifs play a regulatory role not at the RNA, but at the DNA level. Upstream CACA motifs (at -489, ~-300 and -264) identified for the DNA replication machinery set were observed in the ESTs, admitting the possibility that the motifs play a role at the post-transcriptional level. While some positionally conserved upstream motifs were observed in the ESTs for the mitochondrial and organellar translation machinery sets of upstream sequences, more evidence would be needed to suggest that the motifs play a role at the RNA level. In general, among the motifs observed in the ESTs, upstream motifs occurring close to the TLS were the most common while those occurring further upstream (at ~-400) were rare. Upstream motifs at ~-700 and above were never observed in the ESTs.

In order to validate some of the positionally conserved motifs identified for sets of *Pf *genes, the motifs have been searched for in the upstream sequences of orthologous genes from other *Plasmodium *species. Upstream CACA motifs for the DNA replication machinery and proteasome sets of genes, C-rich motifs for the mitochondrial genes and TGTG motifs for the organellar translation machinery genes were all observed in the orthologous upstream sequences, sometimes at positions at which positionally conserved motifs were observed in *Pf*. These observations lend considerable validity to these motifs being regulatory motifs. Oddly, G-rich motifs for the ribonucleotide synthesis and organellar translation machinery sets of genes were not observed in orthologous upstream sequences from other *Plasmodium *species. Since G-rich motifs for *two *sets of genes were not observed, it is possible that the occurrence of these motifs, in these sets of genes, is unique to *Pf*.

Some motif occurrences, in the present study, show positional conservation at positions ranging from -1 to -800 while others show conservation between -1000 and -2000. One might speculate that motifs occurring at the former positions constitute TFBMs [25], while those occurring at the latter positions probably constitute upstream enhancer elements. In the ribonucleotide synthesis, proteasome, mitochondrial, organellar translation machinery and DNA replication machinery sets of genes, positionally conserved upstream motifs were observed. Many of the upstream sequences in these sets are < 2000 nt in length (Table 1), ranging between 600 and 1800 nt. Given the positional conservation of motifs, one is led to speculate that these sets of genes are transcriptionally regulated. To conclude, it is felt that, by looking for positional conservation of motif occurrences, a way has been found to gain more insight into regulatory elements that might be responsible for transcriptional regulation in *Pf*. If experimental approaches were to be used to evaluate the positionally conserved motifs identified in the present study, the functional groups on which efforts could be focused would be the ribonucleotide synthesis, mitochondrial, organellar translation machinery, proteasome and DNA replication machinery groups.

## Conclusion

The study identifies positionally conserved, over-represented motifs in the upstream regions of functionally related, co-expressed *Pf *genes. Positional conservation with respect to the TLS, observed even for genes located on different chromosomes, increases the significance of some of the identified over-represented motifs. The positionally conserved arrangement of motifs in the upstream regions of some sets of genes (e.g., the ribonucleotide synthesis genes) suggests a regulatory role for the motifs which may include control of co-expression of the sets of genes. Further, for the different sets of genes expressed in a transition, the observed combination of upstream motifs tends to be similar; this may be a regulatory mechanism for co-ordinating the expression of sets of genes that are expressed in the transition. Thus, the study throws light on regulatory elements for transcription in the *Pf *IDC. The identified motifs may be regarded as useful hypotheses for experimentally probing transcriptional regulation of gene co-expression in *Pf*.

## Methods

### (1) Dataset

The asexual IDC transcriptome of *Pf *has previously been studied, and 13 groups of nuclear encoded, functionally related, co-expressed genes have been listed in Table S2 of reference [3]. The 13 groups of genes constituted the dataset for this study and the aim was to search for over-represented motifs in the upstream regions of each group. To begin with, it was necessary to extract the upstream regions. In order to do this, *Pf *chromosome, coding and protein sequence files (dated February 2005) were first downloaded from PlasmoDB [34]. A program was then written in Perl to use the genome annotation given in the coding sequence file, to extract, from the sequence of each *Pf *chromosome, the exons, introns, upstream and downstream regions of each gene. Upstream regions were considered on only one DNA strand; this was the strand on which the gene was encoded. All sequences used and presented in this study are in the 5'- to 3'-orientation. For the purpose of motif searching, only 2000 nt upstream of the TLS of each gene were considered; the remaining upstream sequence of the gene (extending up to the nearest upstream gene) was left out. If the gene of interest was separated from its upstream gene by < 2000 nt, then nt belonging to the upstream gene were left out. The choice of 2000 nt was guided by a previous study in which 2000 nt upstream of the TLS of *hsp *genes have been considered for motif-discovery [17]. In [21], 1000 nt upstream of the TLS of each gene have been considered for motif-searching, after determining that *Pf *intergenic regions were on average 1694 nt in length. The TLS has been used as a proxy for the TSS because the latter has not been determined for most *Pf *genes.

### (2) Definitions

In the present study, the terms – motif, motif occurrence, motif-set, consensus motif and motif-group – have been used and need to be explained. In a set of upstream regions, a particular short stretch of sequence (approximately 6–12 nt in length) may be over-represented; the short stretch of sequence has been referred to as a 'motif'. All occurrences of this and closely related motifs, in the set of upstream sequences, have been referred to as 'motif occurrences'. The collective set of motif occurrences, in the set of upstream sequences, has been referred to as a 'motif-set'. The motif occurrences may be aligned and used to obtain a 'consensus motif'. A group of related motif-sets (explained below), identified by the same or different programs, has been referred to as a 'motif group'. In Table 2, for example, each block of sequences is a motif-set, and each sequence or motif in the motif-set is a motif occurrence; the sequences constituting each motif-set are aligned and used to obtain a 'consensus motif' which is given at the top of the motif-set; all motif-sets in Table 2 are related (explained below) and constitute a 'motif-group'. While discussing results, however, for convenience, 'motif-group' has also been referred to, merely, as 'motif'.

### (3) Motif-discovery programs used

Three motif-discovery programs have been used to search for over-represented motifs in the upstream regions of each of the above-mentioned 13 sets of genes. The programs were downloaded over the web and used as standalones. A description of how the 3 programs have been used follows. Parameters not specified below were left at their default values.

*(i) MEME *uses an Expectation Maximization algorithm to discover over-represented motifs [22]. The program was used to search for motifs of length 6–12 nt, with E-values ≤ 1, only on the forward strands of the input sequences. For each set of upstream regions, motif searching was carried out using both 'zoops' (zero or one occurrence per sequence) and 'anr' (any number of repetitions) options. These options determine the distribution of motifs in the upstream sequences. Information obtained from both the 'zoops' and 'anr' runs was found to be useful. From the output of both runs, motif-sets with E-values ≥ 0.1 were discarded. Motif-sets consisting of ATATATAT, TTTTTTTT, AAAAAAA, etc., motifs were ignored because they were not likely to be unique to any particular set of genes (Results). For the remaining, significant motif-sets, sequence logos [35,36] were generated, using the motif occurrences in each set (Figures 1, 2, 3 and 4).

*(ii) Weeder *uses an enumerative algorithm and a suffix tree representation of input sequences to find over-represented motifs [23,24]. The program was run in the 'large' mode which reports motifs of lengths 6, 8, 10 and 12 nt, and, as a degree of approximation, allows 1, 2, 3 and 4 mutations, respectively, to occur in the instances of the motifs. A single-stranded analysis was carried out, and 'PF' (which stands for *Plasmodium falciparum*) was selected as the organism to be used for modeling the background frequencies. The motifs were assumed to appear in 'some' sequences of the set, and they were expected to occur once per input sequence. The 15 highest-scoring motifs of each length were saved. The "advice" provided in the Weeder output file was used to select significant motifs for each set of genes. Motifs regarded as "interesting (highest-ranking)" in the output were considered; if only AT-rich motifs were picked in this list, motifs regarded in the output as "interesting (not highest-ranking)" were also considered. From both lists, motifs consisting entirely of A and T were ignored and the remaining motifs were selected. The occurrences of each selected motif in the input set of sequences were picked using the 'locator' program. Occurrences with 0 or 1 mutations and with a match percentage ≥ 90% (between the motif and its occurrence) were accepted. Motifs with too many occurrences in the set of upstream sequences were rejected because they were not likely to be unique. For the remaining motifs, sequence logos were created, using the motif occurrences.

*(iii) AlignACE *relies on a Gibbs sampling algorithm to find over-represented motifs [25]. While running the program, the GC-content parameter ('gcback') was set to 0.13 (the value used in [17]), which is the fractional GC-content of *Pf *intergenic regions. The 'numcols' parameter, which specifies the number of nt columns to align, was assigned values from 6 to 10, and for each 'numcols' value, AlignACE was run twice. Thus, for each set of upstream regions, AlignACE was run 10 times. This was done in order to obtain a greater variety of consensus motifs. The 'expect' value, which is the number of motif occurrences to expect in the set of sequences, was set to 3; the oversample parameter was also set to 3. At the end of the AlignACE runs, only consensus motifs with MAP (maximum *a priori *log likelihood) scores > 10 were accepted, as this was the score previously used to identify yeast TFBMs [25]. Next, the ScanACE program was used to scan the upstream regions of all *Pf *genes for the presence of the accepted AlignACE consensus motifs. For ScanACE, the GC-content parameter was set to 0.13, and the maximum number of motif occurrences to be returned was set to 1000. The MotifStats program was next run; it uses the output from ScanACE to calculate the group specificity and positional specificity scores for each consensus motif. Motifs with the smallest group and positional specificity scores were regarded as being significant. It has been pointed out that TFBMs in yeast show high group specificity and positional bias ([25]; both scores are < 10^-10^). In the present *Pf *study, however, both scores were always found to be > 10^-10^. The CompareACE program was next used to perform a pairwise comparison between all consensus motifs. The motifs were then clustered (c > 0.6) using the Tree and list_clusters.pl programs. From the final list of consensus motif clusters, motifs with the best group specificity scores and with good positional bias scores were manually selected. For each of these consensus motifs, a sequence logo was created, using motif occurrences, identified by AlignACE, on the forward strand alone.

### (4) Grouping motif-sets

After the 3 motif-discovery programs had been run for a set of upstream regions, there would be motif-sets identified by MEME, motif-sets identified by Weeder and motif-sets identified by AlignACE; each motif-set would also have its sequence logo. The motif-sets were then compared among themselves to see if they could be grouped. For this, the sequence logos of the motif-sets were first visually compared, and, if they appeared similar, the motif-sets were tentatively grouped. Motif-sets within each tentative group were then compared. No 2 motif-sets were ever identical; the motif occurrences and their lengths tended to differ. Therefore, in the present study, 2 motif-sets have been regarded as being strongly related, if over 2 motif occurrences in one overlapped with motif occurrences in the other. (2 motif-sets have been regarded as being weakly related, if only 1 or 2 motif occurrences in one, overlapped with motif occurrences in the other). Strongly related motif-sets were then grouped together to form a motif group. A motif group has been regarded as a strong motif group if it included motif-sets identified by more than one program (exception discussed below). The motif-sets in Table 2, for example, are strongly related; in the different motif-sets, motif occurrences which overlap have been marked by asterisks. The motif-sets in the table, and the corresponding sequence logos in Figure 1, constitute a strong motif-group because the group includes motif-sets identified by more than one program. Thus, motif-sets were grouped together, based on a manual comparison of their motif occurrences. Each strong motif group was regarded as a potential regulatory motif.

Greater confidence was reposed in strongly related motif-sets identified by more than one motif discovery program. If 2 programs, using different algorithms to find statistically over-represented motifs, regard nearly the same set of sequences as being over-represented, then the chances are greater that the set of sequences are significant and, perhaps, biologically important. However, over-represented motifs identified by a single program have also been considered as putative regulatory motifs if they consisted primarily of G and/or C and if they occurred frequently in a set of upstream sequences (e.g., the C-rich motif identified by MEME, for the mitochondrial genes; Figure 2). There were also instances, in a motif group, when closely related motif-sets had been identified several times by the same program (in Table 2, for example, AlignACE has identified 5 related motif-sets). However, once the motif-sets had been grouped, all motif occurrences in the group (all motif occurrences in Table 2, for example) were regarded as occurrences of the same motif. All strong and weak motif groups identified for each of the 13 sets of genes are given in Additional files 1, 2, 3, and 4.

In order to independently check if the motif-sets grouped together to form each strong motif group were indeed similar, the web tool, STAMP [37], was used. The motif-sets in each motif group were input to the program, which tries to build a tree showing the similarity between the motif-sets. Similarity tree building was attempted for all 27 strong motif groups identified in the study. For 14 strong motif groups, the *worst *pairwise similarity between motif-sets was < 0.1, indicating that there was a high degree of similarity between motif-sets in each group. This justified the grouping of the motif-sets into their respective motif groups. For 9 more strong motif groups, no tree was generated because there were only 2 motif-sets to be compared, in each group; for 3 of the remaining 4, the worst pairwise similarity between motif-sets was < 0.2 and for 1, it was < 0.33 (this was the C-rich motif group identified for the large set of 132 cytoplasmic translation machinery genes; Additional file 1:13). Thus, overall, the results of STAMP corroborated the manual grouping of motif-sets.

### (5) Sorting and visualizing motif occurrences

After the motif-sets had been grouped, the next step was to sort and visualize the motif occurrences in each motif group. By sorting the motif occurrences, the best subset of occurrences in each motif group was being considered. As discussed under Results, 4 kinds of motif groups were identified for the 13 sets of genes. They were the G-rich, the C-rich, the CACA and the TGTG motif groups (Figures 1, 2, 3 and 4). The strong motif groups identified for each set of genes were considered one at a time, and the motif occurrences in the group were sorted. The Linux 'grep' filter was used to do all the sorting. Considering, first, a G-rich motif group (e.g., Table 2), all motif occurrences in the group were pooled together and were then sorted by separating motif occurrences with a maximum of 4 (or more) Gs, 3Gs and 2Gs. Likewise, for each C-rich motif group (Figure 2 and Additional file 2:37), motif occurrences with a maximum of 4 (or more) Cs, 3Cs and 2Cs were separated. In each CACA motif group (Figure 3, Additional file 2:12, 29), in order to retain only the most significant motifs, motifs containing the sequence ACACAC and also those where at most 2As were replaced by Gs and at most the corresponding 2 Cs were replaced by Ts were considered; motifs containing the sequence ACAC or CACA and also those where one or both As were replaced by Gs were considered. Likewise, in each TGTG motif group (Figure 4 and Additional file 2:48), motifs containing the sequence TGTG or GTGT and also those where one or both Ts were replaced by Cs were considered; motifs containing the sequence TGTATG and also those where the second T was replaced by C were considered, and motifs containing the sequence GTATGT and also those where the first and/or third Ts were replaced by Cs were considered. These substitutions were selected because they were observed among the motifs picked by the motif-discovery programs. In order to allow the majority of motifs picked by the programs to be included among the sorted motifs, these substitutions were permitted, during sorting. Motifs such as CATATA or TGTATA were left out because they would be virtually indistinguishable from the TATATA sequences that occur in plenty in the *Pf *genome. Motif occurrences in each strong motif group, after sorting, are listed in Additional file 5. For example, 4G-, 3G-, 2G- and 1G-containing motifs, in the strong motif group in Table 2, are listed in Additional file 5, after sorting.

The next step was to visualize the positions at which the sorted motifs occurred in the upstream regions of the appropriate set of genes. The dna-pattern and feature map programs were used for this [27,38,39]. The sorted motif occurrences and the relevant set of upstream regions were input to the dna-pattern program. For example, sorted 4G- and 3G-containing motifs, in Additional file 5, for the transcription machinery genes, together with the relevant set of upstream sequences, were input to the program. The program locates all occurrences of each input motif in the set of upstream sequences. These occurrences are input to the feature map program which generates a graphical feature map marking the positions of the occurrences in the upstream regions (Figures 5, 6, 7, 8, 9, 10 and 11). For each feature map discussed in the text, the sequence of each motif occurring in the map, and the position at which it occurs, with respect to the TLS, is given in Additional file 6. For example, the sequence and position of each upstream motif in the feature map for the transcription machinery genes (Figure 5), is shown, in order, in Additional file 6. Feature maps for sorted motif occurrences are shown in Figures 5, 6, 7, 8, 9, 10 and 11 and in Additional file 7, and for unsorted motif occurrences in Additional files 1, 2, 3, and 4.

### (6) Statistical validations of putative regulatory motifs

#### (i) Testing the significance of the motifs identified by the motif-discovery programs

The upstream sequences of 10 sets of genes in Table 1, for which strong motif groups have been identified, were used for testing. (The large set of 132 cytoplasmic translation machinery genes was left out because it would take too much time to get through the tests). For testing, the above 10 sets of upstream sequences were shuffled using the 'shuffleseq' program in the EMBOSS software suite [40] (the program shuffles individual sequences in each set, while maintaining composition). From each set of upstream sequences, 10 shuffled sets of sequences were obtained. The 3 motif-discovery programs were then used to search for over-represented motifs in each of the 10 shuffled sets. MEME was run 200 times – once each in 'zoops' and 'anr' modes, for each of the 10 shuffled sets, for each of the 10 sets of upstream sequences. AlignACE was run 140 times: 14 times for each of the 10 sets of upstream sequences; 14 times for an upstream set, because it was run once for each of the 10 shuffled sets with numcols set to 6, and 4 more times with any 4 of the shuffled sets with 'numcols' set to 7, 8, 9 and 10. Weeder was run 100 times: once for each of the 10 shuffled sets, of each of the 10 sets of upstream sequences. The motifs identified in the shuffled sets (if any), and their scores (e.g., MAP score for AlignACE motifs), were compared with the motifs and scores obtained with the original, unshuffled set of upstream sequences. A motif identified in the original upstream set was regarded as significant: (i) if it was not identified in the shuffled sets and (ii) if its scores were better than the scores obtained for the random motifs.

#### (ii) Statistical validation of positionally conserved motifs

The upstream region (extending from -2000 to -1) was divided into 39 overlapping windows of width 100 nt. The starting point of consecutive windows differed by 50 nt; that is, windows were considered in the following way: -2000 to -1901, -1950 to -1851, -1900 to -1801, ..., -250 to -151, -200 to -101, -150 to -51, -100 to -1. Positional conservation of motifs, in sets of upstream sequences, was examined in these windows of width 100 nt. For each set of motifs in a set of upstream sequences, the number of motifs occurring in each window, and the total number of sequences with one or motifs, occurring in each window, was tabulated using the data presented in Additional file 6. The total number of motifs in a set of upstream sequences, and the number of motifs occurring in each upstream sequence, was also tabulated using Additional file 6.

The statistical significance of positionally conserved motifs was examined by carrying out Monte Carlo simulations on each set of motifs occurring in a set of upstream sequences. Each simulation consisted of shuffling the positions of the motifs in the upstream sequences, and then examining the occupancy of the motifs in each of the above windows. The null hypothesis that was to be tested was that there were no favored regions for the motifs; i.e., motifs could occur anywhere in the upstream sequences with equal probability. In each simulation, while randomly distributing motifs in the upstream sequences, care was taken to ensure that: (i) the number of motifs occurring in each upstream sequence was the same as the observed number of motifs in the sequence, and (ii) that there were no overlapping motifs in any sequence. The occupancy of motifs in the upstream windows was then examined in the following way. Let Nmio denote the *o*bserved number of *m*otifs in the *i*th window and let Nsio denote the *o*bserved number of *s*equences with one or more motifs, in the *i*th window (tabulated as described above, from Additional file 6). Analogously, let Nmi' and Nsi' denote the corresponding values observed in each Monte Carlo simulation. These latter values would describe the occupancy of motifs in the upstream windows, in each simulation. A total of 20,000 simulations were carried out for each set of motifs occurring in a set of upstream sequences, and Nmi' and Nsi' were counted for each simulation. The positional conservation of motifs *observed *in a window (from Additional file 6) was considered to be statistically significant if the number of simulations (out of 20,000), in which Nmi' ≥ Nmio and Nsi' ≥ Nsio, was less than 1000 (this would correspond to p < 0.05; i.e., the probability of observing Nmio or more motifs in the window, and the probability of observing Nsio or more sequences, each with one or more motifs, in the window, by chance, was < 0.05). Since positional enrichment (positional conservation) has been reported previously for similar datasets [21], the objective of the above analysis was to examine statistical evidence in support of a similar pattern in the present dataset. We would therefore prefer to describe our results as suggestive (and not necessarily conclusive) of positional conservation of motifs. Details for the statistically validated, positionally conserved motifs are given in Additional file 8, and the motifs are highlighted in Additional file 6.

## Abbreviations

TLS: translational start site; *Pf*: *Plasmodium falciparum*; TF: transcription factor; TFBM: transcription factor binding motif; IDC: intraerythrocytic developmental cycle; TAP: transcription associated protein; TSS: transcription start site; nt: nucleotide; UTR: untranslated region; *hsp*: heat shock protein; MAP: maximum *a priori *log likelihood; EST: expressed sequence tag.

## Authors' contributions

PI conceived of the study, carried out motif detection and drafted the manuscript. NVJ carried out the statistical validation of motifs. All authors read and approved the final manuscript.

## Supplementary Material

Additional file 1 **Over-represented upstream motifs identified for the 4 functional groups of genes expressed during the ring to early trophozoite transition.** Sets of over-represented motifs identified for each functional group by 3 motif-discovery programs, sequence logos for the sets, grouping of the sets into strong and weak motif groups, and feature maps for each group, are given. Click here for file

Additional file 2 **Over-represented upstream motifs identified for the 6 functional groups of genes expressed during the trophozoite to early schizont transition.** Strong and weak motif groups identified for each functional group are given. Click here for file

Additional file 3 **Over-represented upstream motifs identified for the 2 functional groups of genes expressed during the mid- and late-schizont stages.** Strong and weak motif groups identified for each functional group are given. Click here for file

Additional file 4 **Over-represented upstream motifs identified for the single functional group of genes expressed during the early ring stage.** Over-represented motif sets identified for the functional group are given. Click here for file

Additional file 5 **Motif occurrences in the strong motif groups, identified for each of the 13 functional groups of genes, after sorting.** These motif occurrences were used to obtain feature maps. Click here for file

Additional file 6 **The list of putative regulatory motifs occurring in the upstream regions of the 13 functional groups of genes.** The sequence and upstream position of each putative regulatory motif is given. Click here for file

Additional file 7 **Feature maps for the strong motif groups identified in the upstream regions of the 13 functional groups of genes.** The maps have been obtained after sorting the motifs in each strong motif group. Click here for file

Additional file 8 **Statistically validated positionally conserved motifs.** Positionally conserved upstream motifs which have been statistically validated by Monte Carlo simulations are listed. Click here for file

Additional file 9 **Feature maps showing the occurrence of putative *Pf *regulatory motifs in ESTs and orthologous sequences.** The maps show the distribution of putative *Pf *regulatory motifs (a) in ESTs retrieved for the gene upstream sequences and (b) in the upstream sequences of orthologous genes from other *Plasmodium *species. Click here for file

Additional file 10 **Positional conservation information for the strong motif groups identified for the 13 functional groups of genes.** The table summarizes the positional conservation information for motifs in all functional groups and helps the reader navigate through the Additional files. Click here for file
