# Supplementary material for: Identification of putative regulatory motifs in the upstream regions of co-expressed functional groups of genes in Plasmodium falciparum
Source: BMC Genomics. 2009 Jan 13;10:18. doi: 10.1186/1471-2164-10-18 (PMC2662883; doi:10.1186/1471-2164-10-18)
Supplement: Additional file 1 — Over-represented upstream motifs identified for the 4 functional groups of genes expressed during the ring to early trophozoite transition. Sets of over-represented motifs identified for each functional group by 3 motif-discovery programs, sequence logos for the sets, grouping of the sets into strong and weak motif groups, and feature maps for each group, are given. [file 1471-2164-10-18-S1.ppt]

## Slide 1
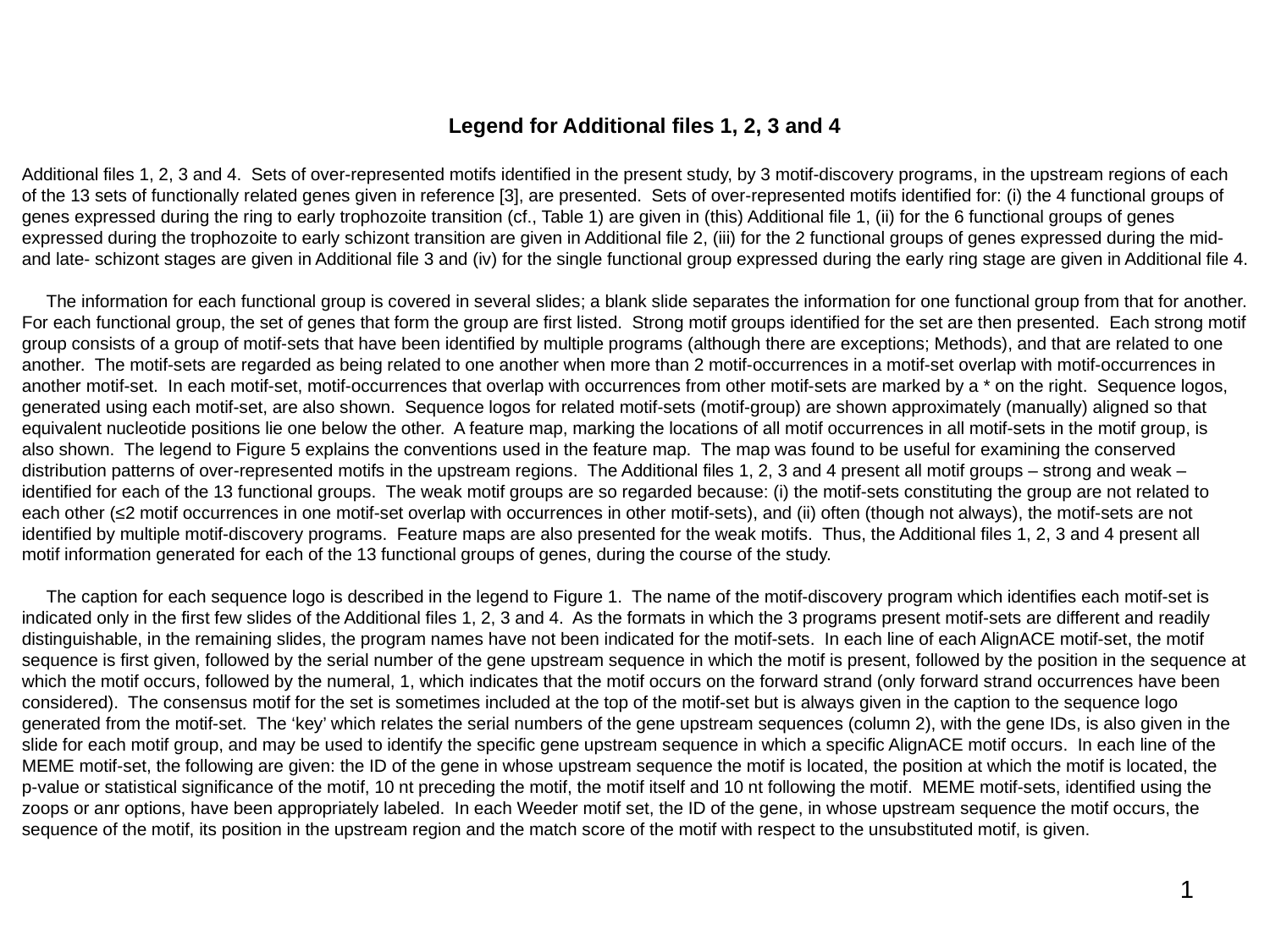

Legend for Additional files 1, 2, 3 and 4
Additional files 1, 2, 3 and 4. Sets of over-represented motifs identified in the present study, by 3 motif-discovery programs, in the upstream regions of each
of the 13 sets of functionally related genes given in reference [3], are presented. Sets of over-represented motifs identified for: (i) the 4 functional groups of
genes expressed during the ring to early trophozoite transition (cf., Table 1) are given in (this) Additional file 1, (ii) for the 6 functional groups of genes
expressed during the trophozoite to early schizont transition are given in Additional file 2, (iii) for the 2 functional groups of genes expressed during the mid-
and late- schizont stages are given in Additional file 3 and (iv) for the single functional group expressed during the early ring stage are given in Additional file 4.
 The information for each functional group is covered in several slides; a blank slide separates the information for one functional group from that for another.
For each functional group, the set of genes that form the group are first listed. Strong motif groups identified for the set are then presented. Each strong motif
group consists of a group of motif-sets that have been identified by multiple programs (although there are exceptions; Methods), and that are related to one
another. The motif-sets are regarded as being related to one another when more than 2 motif-occurrences in a motif-set overlap with motif-occurrences in
another motif-set. In each motif-set, motif-occurrences that overlap with occurrences from other motif-sets are marked by a * on the right. Sequence logos,
generated using each motif-set, are also shown. Sequence logos for related motif-sets (motif-group) are shown approximately (manually) aligned so that
equivalent nucleotide positions lie one below the other. A feature map, marking the locations of all motif occurrences in all motif-sets in the motif group, is
also shown. The legend to Figure 5 explains the conventions used in the feature map. The map was found to be useful for examining the conserved
distribution patterns of over-represented motifs in the upstream regions. The Additional files 1, 2, 3 and 4 present all motif groups – strong and weak –
identified for each of the 13 functional groups. The weak motif groups are so regarded because: (i) the motif-sets constituting the group are not related to
each other (≤2 motif occurrences in one motif-set overlap with occurrences in other motif-sets), and (ii) often (though not always), the motif-sets are not
identified by multiple motif-discovery programs. Feature maps are also presented for the weak motifs. Thus, the Additional files 1, 2, 3 and 4 present all
motif information generated for each of the 13 functional groups of genes, during the course of the study.
 The caption for each sequence logo is described in the legend to Figure 1. The name of the motif-discovery program which identifies each motif-set is
indicated only in the first few slides of the Additional files 1, 2, 3 and 4. As the formats in which the 3 programs present motif-sets are different and readily
distinguishable, in the remaining slides, the program names have not been indicated for the motif-sets. In each line of each AlignACE motif-set, the motif
sequence is first given, followed by the serial number of the gene upstream sequence in which the motif is present, followed by the position in the sequence at
which the motif occurs, followed by the numeral, 1, which indicates that the motif occurs on the forward strand (only forward strand occurrences have been
considered). The consensus motif for the set is sometimes included at the top of the motif-set but is always given in the caption to the sequence logo
generated from the motif-set. The ‘key’ which relates the serial numbers of the gene upstream sequences (column 2), with the gene IDs, is also given in the
slide for each motif group, and may be used to identify the specific gene upstream sequence in which a specific AlignACE motif occurs. In each line of the
MEME motif-set, the following are given: the ID of the gene in whose upstream sequence the motif is located, the position at which the motif is located, the
p-value or statistical significance of the motif, 10 nt preceding the motif, the motif itself and 10 nt following the motif. MEME motif-sets, identified using the
zoops or anr options, have been appropriately labeled. In each Weeder motif set, the ID of the gene, in whose upstream sequence the motif occurs, the
sequence of the motif, its position in the upstream region and the match score of the motif with respect to the unsubstituted motif, is given.
1

## Slide 2
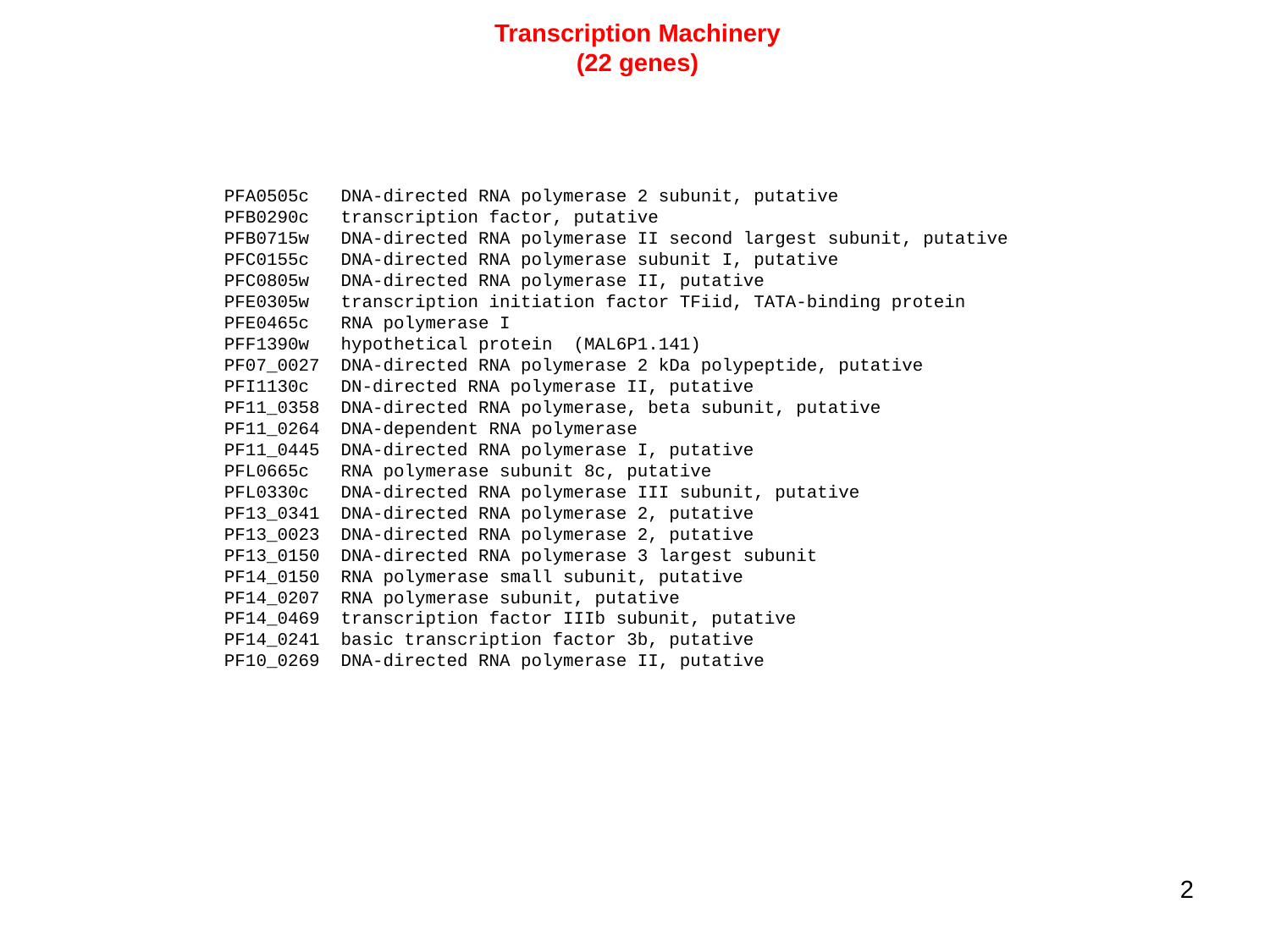

Transcription Machinery
(22 genes)
PFA0505c DNA-directed RNA polymerase 2 subunit, putative
PFB0290c transcription factor, putative
PFB0715w DNA-directed RNA polymerase II second largest subunit, putative
PFC0155c DNA-directed RNA polymerase subunit I, putative
PFC0805w DNA-directed RNA polymerase II, putative
PFE0305w transcription initiation factor TFiid, TATA-binding protein
PFE0465c RNA polymerase I
PFF1390w hypothetical protein (MAL6P1.141)
PF07_0027 DNA-directed RNA polymerase 2 kDa polypeptide, putative
PFI1130c DN-directed RNA polymerase II, putative
PF11_0358 DNA-directed RNA polymerase, beta subunit, putative
PF11_0264 DNA-dependent RNA polymerase
PF11_0445 DNA-directed RNA polymerase I, putative
PFL0665c RNA polymerase subunit 8c, putative
PFL0330c DNA-directed RNA polymerase III subunit, putative
PF13_0341 DNA-directed RNA polymerase 2, putative
PF13_0023 DNA-directed RNA polymerase 2, putative
PF13_0150 DNA-directed RNA polymerase 3 largest subunit
PF14_0150 RNA polymerase small subunit, putative
PF14_0207 RNA polymerase subunit, putative
PF14_0469 transcription factor IIIb subunit, putative
PF14_0241 basic transcription factor 3b, putative
PF10_0269 DNA-directed RNA polymerase II, putative
2

## Slide 3
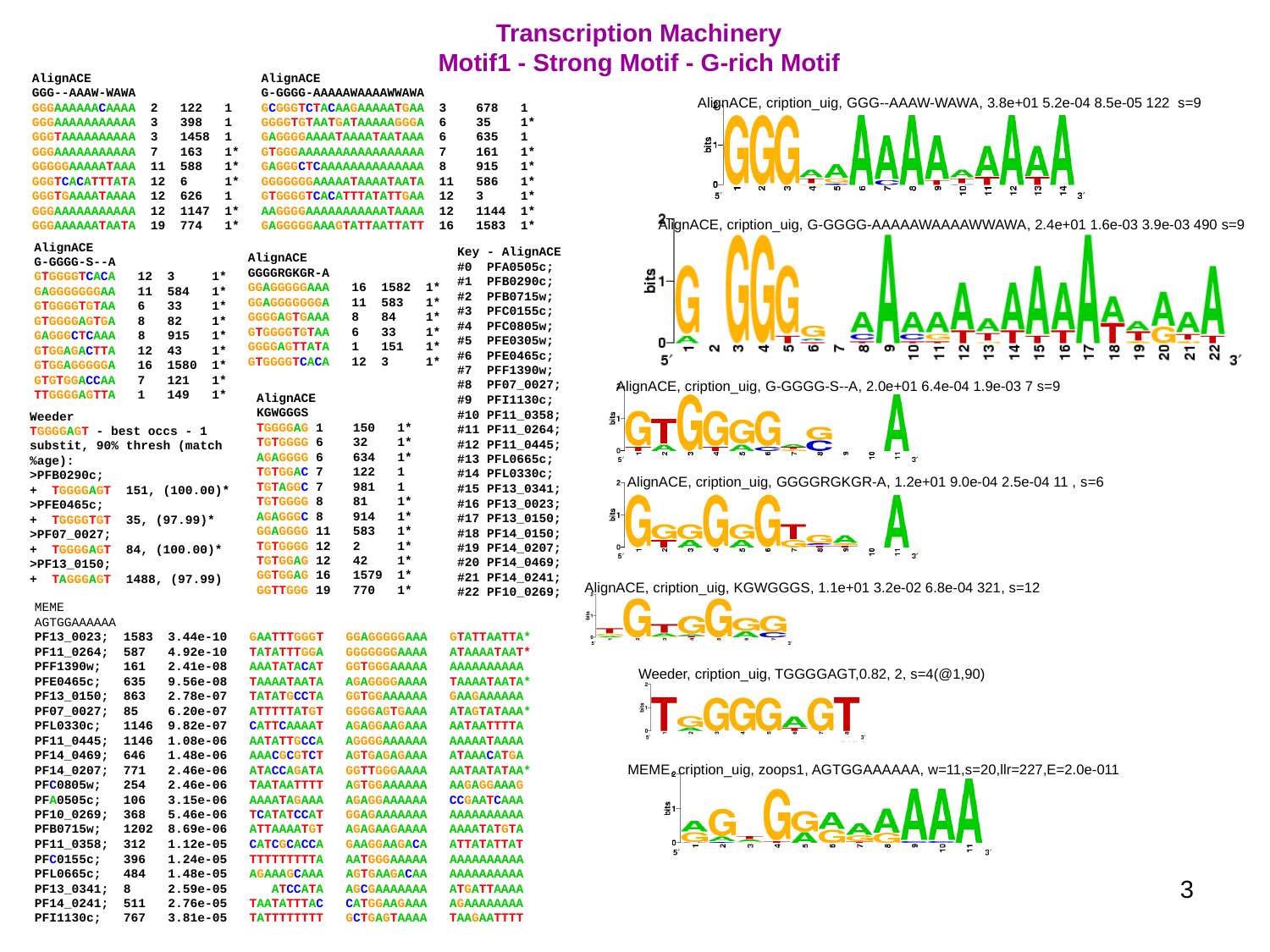

Transcription Machinery
Motif1 - Strong Motif - G-rich Motif
AlignACE
GGG--AAAW-WAWA
GGGAAAAAACAAAA 2 122 1
GGGAAAAAAAAAAA 3 398 1
GGGTAAAAAAAAAA 3 1458 1
GGGAAAAAAAAAAA 7 163 1*
GGGGGAAAAATAAA 11 588 1*
GGGTCACATTTATA 12 6 1*
GGGTGAAAATAAAA 12 626 1
GGGAAAAAAAAAAA 12 1147 1*
GGGAAAAAATAATA 19 774 1*
AlignACE
G-GGGG-AAAAAWAAAAWWAWA
GCGGGTCTACAAGAAAAATGAA 3 678 1
GGGGTGTAATGATAAAAAGGGA 6 35 1*
GAGGGGAAAATAAAATAATAAA 6 635 1
GTGGGAAAAAAAAAAAAAAAAA 7 161 1*
GAGGGCTCAAAAAAAAAAAAAA 8 915 1*
GGGGGGGAAAAATAAAATAATA 11 586 1*
GTGGGGTCACATTTATATTGAA 12 3 1*
AAGGGGAAAAAAAAAAATAAAA 12 1144 1*
GAGGGGGAAAGTATTAATTATT 16 1583 1*
AlignACE, cription_uig, GGG--AAAW-WAWA, 3.8e+01 5.2e-04 8.5e-05 122 s=9
AlignACE, cription_uig, G-GGGG-AAAAAWAAAAWWAWA, 2.4e+01 1.6e-03 3.9e-03 490 s=9
AlignACE, cription_uig, G-GGGG-S--A, 2.0e+01 6.4e-04 1.9e-03 7 s=9
AlignACE, cription_uig, GGGGRGKGR-A, 1.2e+01 9.0e-04 2.5e-04 11 , s=6
AlignACE, cription_uig, KGWGGGS, 1.1e+01 3.2e-02 6.8e-04 321, s=12
Weeder, cription_uig, TGGGGAGT,0.82, 2, s=4(@1,90)
MEME, cription_uig, zoops1, AGTGGAAAAAA, w=11,s=20,llr=227,E=2.0e-011
AlignACE
G-GGGG-S--A
GTGGGGTCACA 12 3 1*
GAGGGGGGGAA 11 584 1*
GTGGGGTGTAA 6 33 1*
GTGGGGAGTGA 8 82 1*
GAGGGCTCAAA 8 915 1*
GTGGAGACTTA 12 43 1*
GTGGAGGGGGA 16 1580 1*
GTGTGGACCAA 7 121 1*
TTGGGGAGTTA 1 149 1*
Key - AlignACE
#0 PFA0505c;
#1 PFB0290c;
#2 PFB0715w;
#3 PFC0155c;
#4 PFC0805w;
#5 PFE0305w;
#6 PFE0465c;
#7 PFF1390w;
#8 PF07_0027;
#9 PFI1130c;
#10 PF11_0358;
#11 PF11_0264;
#12 PF11_0445;
#13 PFL0665c;
#14 PFL0330c;
#15 PF13_0341;
#16 PF13_0023;
#17 PF13_0150;
#18 PF14_0150;
#19 PF14_0207;
#20 PF14_0469;
#21 PF14_0241;
#22 PF10_0269;
AlignACE
GGGGRGKGR-A
GGAGGGGGAAA 16 1582 1*
GGAGGGGGGGA 11 583 1*
GGGGAGTGAAA 8 84 1*
GTGGGGTGTAA 6 33 1*
GGGGAGTTATA 1 151 1*
GTGGGGTCACA 12 3 1*
AlignACE
KGWGGGS
TGGGGAG 1 150 1*
TGTGGGG 6 32 1*
AGAGGGG 6 634 1*
TGTGGAC 7 122 1
TGTAGGC 7 981 1
TGTGGGG 8 81 1*
AGAGGGC 8 914 1*
GGAGGGG 11 583 1*
TGTGGGG 12 2 1*
TGTGGAG 12 42 1*
GGTGGAG 16 1579 1*
GGTTGGG 19 770 1*
Weeder
TGGGGAGT - best occs - 1 substit, 90% thresh (match %age):
>PFB0290c;
+ TGGGGAGT 151, (100.00)*
>PFE0465c;
+ TGGGGTGT 35, (97.99)*
>PF07_0027;
+ TGGGGAGT 84, (100.00)*
>PF13_0150;
+ TAGGGAGT 1488, (97.99)
MEME
AGTGGAAAAAA
PF13_0023; 1583 3.44e-10 GAATTTGGGT GGAGGGGGAAA GTATTAATTA*
PF11_0264; 587 4.92e-10 TATATTTGGA GGGGGGGAAAA ATAAAATAAT*
PFF1390w; 161 2.41e-08 AAATATACAT GGTGGGAAAAA AAAAAAAAAA
PFE0465c; 635 9.56e-08 TAAAATAATA AGAGGGGAAAA TAAAATAATA*
PF13_0150; 863 2.78e-07 TATATGCCTA GGTGGAAAAAA GAAGAAAAAA
PF07_0027; 85 6.20e-07 ATTTTTATGT GGGGAGTGAAA ATAGTATAAA*
PFL0330c; 1146 9.82e-07 CATTCAAAAT AGAGGAAGAAA AATAATTTTA
PF11_0445; 1146 1.08e-06 AATATTGCCA AGGGGAAAAAA AAAAATAAAA
PF14_0469; 646 1.48e-06 AAACGCGTCT AGTGAGAGAAA ATAAACATGA
PF14_0207; 771 2.46e-06 ATACCAGATA GGTTGGGAAAA AATAATATAA*
PFC0805w; 254 2.46e-06 TAATAATTTT AGTGGAAAAAA AAGAGGAAAG
PFA0505c; 106 3.15e-06 AAAATAGAAA AGAGGAAAAAA CCGAATCAAA
PF10_0269; 368 5.46e-06 TCATATCCAT GGAGAAAAAAA AAAAAAAAAA
PFB0715w; 1202 8.69e-06 ATTAAAATGT AGAGAAGAAAA AAAATATGTA
PF11_0358; 312 1.12e-05 CATCGCACCA GAAGGAAGACA ATTATATTAT
PFC0155c; 396 1.24e-05 TTTTTTTTTA AATGGGAAAAA AAAAAAAAAA
PFL0665c; 484 1.48e-05 AGAAAGCAAA AGTGAAGACAA AAAAAAAAAA
PF13_0341; 8 2.59e-05 ATCCATA AGCGAAAAAAA ATGATTAAAA
PF14_0241; 511 2.76e-05 TAATATTTAC CATGGAAGAAA AGAAAAAAAA
PFI1130c; 767 3.81e-05 TATTTTTTTT GCTGAGTAAAA TAAGAATTTT
3

## Slide 4
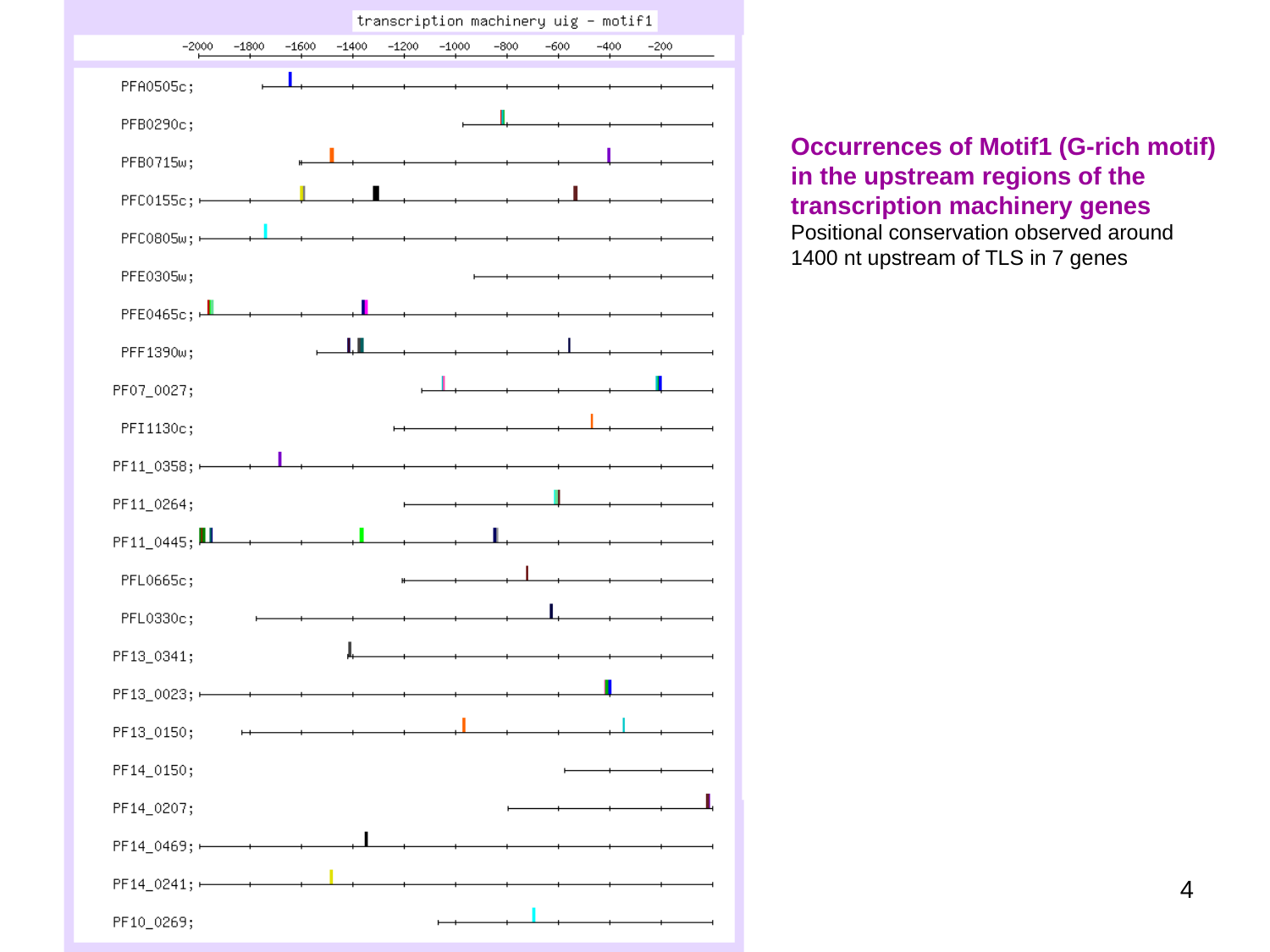

Occurrences of Motif1 (G-rich motif)
in the upstream regions of the
transcription machinery genes
Positional conservation observed around
1400 nt upstream of TLS in 7 genes
4

## Slide 5
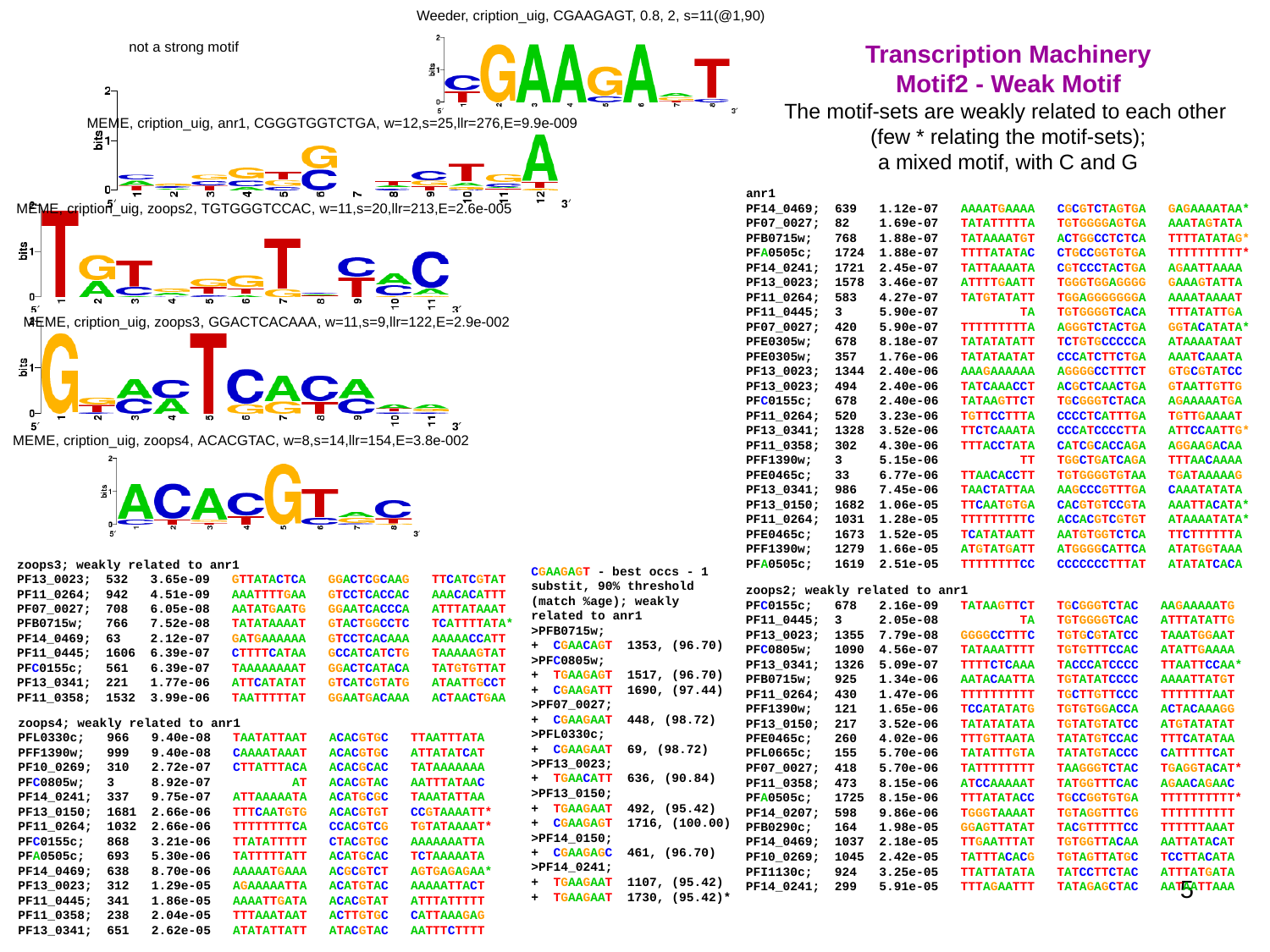

Weeder, cription_uig, CGAAGAGT, 0.8, 2, s=11(@1,90)
MEME, cription_uig, anr1, CGGGTGGTCTGA, w=12,s=25,llr=276,E=9.9e-009
MEME, cription_uig, zoops2, TGTGGGTCCAC, w=11,s=20,llr=213,E=2.6e-005
MEME, cription_uig, zoops3, GGACTCACAAA, w=11,s=9,llr=122,E=2.9e-002
MEME, cription_uig, zoops4, ACACGTAC, w=8,s=14,llr=154,E=3.8e-002
not a strong motif
Transcription Machinery
Motif2 - Weak Motif
The motif-sets are weakly related to each other
(few * relating the motif-sets);
a mixed motif, with C and G
anr1
PF14_0469; 639 1.12e-07 AAAATGAAAA CGCGTCTAGTGA GAGAAAATAA*
PF07_0027; 82 1.69e-07 TATATTTTTA TGTGGGGAGTGA AAATAGTATA
PFB0715w; 768 1.88e-07 TATAAAATGT ACTGGCCTCTCA TTTTATATAG*
PFA0505c; 1724 1.88e-07 TTTTATATAC CTGCCGGTGTGA TTTTTTTTTT*
PF14_0241; 1721 2.45e-07 TATTAAAATA CGTCCCTACTGA AGAATTAAAA
PF13_0023; 1578 3.46e-07 ATTTTGAATT TGGGTGGAGGGG GAAAGTATTA
PF11_0264; 583 4.27e-07 TATGTATATT TGGAGGGGGGGA AAAATAAAAT
PF11_0445; 3 5.90e-07 TA TGTGGGGTCACA TTTATATTGA
PF07_0027; 420 5.90e-07 TTTTTTTTTA AGGGTCTACTGA GGTACATATA*
PFE0305w; 678 8.18e-07 TATATATATT TCTGTGCCCCCA ATAAAATAAT
PFE0305w; 357 1.76e-06 TATATAATAT CCCATCTTCTGA AAATCAAATA
PF13_0023; 1344 2.40e-06 AAAGAAAAAA AGGGGCCTTTCT GTGCGTATCC
PF13_0023; 494 2.40e-06 TATCAAACCT ACGCTCAACTGA GTAATTGTTG
PFC0155c; 678 2.40e-06 TATAAGTTCT TGCGGGTCTACA AGAAAAATGA
PF11_0264; 520 3.23e-06 TGTTCCTTTA CCCCTCATTTGA TGTTGAAAAT
PF13_0341; 1328 3.52e-06 TTCTCAAATA CCCATCCCCTTA ATTCCAATTG*
PF11_0358; 302 4.30e-06 TTTACCTATA CATCGCACCAGA AGGAAGACAA
PFF1390w; 3 5.15e-06 TT TGGCTGATCAGA TTTAACAAAA
PFE0465c; 33 6.77e-06 TTAACACCTT TGTGGGGTGTAA TGATAAAAAG
PF13_0341; 986 7.45e-06 TAACTATTAA AAGCCCGTTTGA CAAATATATA
PF13_0150; 1682 1.06e-05 TTCAATGTGA CACGTGTCCGTA AAATTACATA*
PF11_0264; 1031 1.28e-05 TTTTTTTTTC ACCACGTCGTGT ATAAAATATA*
PFE0465c; 1673 1.52e-05 TCATATAATT AATGTGGTCTCA TTCTTTTTTA
PFF1390w; 1279 1.66e-05 ATGTATGATT ATGGGGCATTCA ATATGGTAAA
PFA0505c; 1619 2.51e-05 TTTTTTTTCC CCCCCCCTTTAT ATATATCACA
zoops3; weakly related to anr1
PF13_0023; 532 3.65e-09 GTTATACTCA GGACTCGCAAG TTCATCGTAT
PF11_0264; 942 4.51e-09 AAATTTTGAA GTCCTCACCAC AAACACATTT
PF07_0027; 708 6.05e-08 AATATGAATG GGAATCACCCA ATTTATAAAT
PFB0715w; 766 7.52e-08 TATATAAAAT GTACTGGCCTC TCATTTTATA*
PF14_0469; 63 2.12e-07 GATGAAAAAA GTCCTCACAAA AAAAACCATT
PF11_0445; 1606 6.39e-07 CTTTTCATAA GCCATCATCTG TAAAAAGTAT
PFC0155c; 561 6.39e-07 TAAAAAAAAT GGACTCATACA TATGTGTTAT
PF13_0341; 221 1.77e-06 ATTCATATAT GTCATCGTATG ATAATTGCCT
PF11_0358; 1532 3.99e-06 TAATTTTTAT GGAATGACAAA ACTAACTGAA
CGAAGAGT - best occs - 1
substit, 90% threshold
(match %age); weakly
related to anr1
>PFB0715w;
+ CGAACAGT 1353, (96.70)
>PFC0805w;
+ TGAAGAGT 1517, (96.70)
+ CGAAGATT 1690, (97.44)
>PF07_0027;
+ CGAAGAAT 448, (98.72)
>PFL0330c;
+ CGAAGAAT 69, (98.72)
>PF13_0023;
+ TGAACATT 636, (90.84)
>PF13_0150;
+ TGAAGAAT 492, (95.42)
+ CGAAGAGT 1716, (100.00)
>PF14_0150;
+ CGAAGAGC 461, (96.70)
>PF14_0241;
+ TGAAGAAT 1107, (95.42)
+ TGAAGAAT 1730, (95.42)*
zoops2; weakly related to anr1
PFC0155c; 678 2.16e-09 TATAAGTTCT TGCGGGTCTAC AAGAAAAATG
PF11_0445; 3 2.05e-08 TA TGTGGGGTCAC ATTTATATTG
PF13_0023; 1355 7.79e-08 GGGGCCTTTC TGTGCGTATCC TAAATGGAAT
PFC0805w; 1090 4.56e-07 TATAAATTTT TGTGTTTCCAC ATATTGAAAA
PF13_0341; 1326 5.09e-07 TTTTCTCAAA TACCCATCCCC TTAATTCCAA*
PFB0715w; 925 1.34e-06 AATACAATTA TGTATATCCCC AAAATTATGT
PF11_0264; 430 1.47e-06 TTTTTTTTTT TGCTTGTTCCC TTTTTTTAAT
PFF1390w; 121 1.65e-06 TCCATATATG TGTGTGGACCA ACTACAAAGG
PF13_0150; 217 3.52e-06 TATATATATA TGTATGTATCC ATGTATATAT
PFE0465c; 260 4.02e-06 TTTGTTAATA TATATGTCCAC TTTCATATAA
PFL0665c; 155 5.70e-06 TATATTTGTA TATATGTACCC CATTTTTCAT
PF07_0027; 418 5.70e-06 TATTTTTTTT TAAGGGTCTAC TGAGGTACAT*
PF11_0358; 473 8.15e-06 ATCCAAAAAT TATGGTTTCAC AGAACAGAAC
PFA0505c; 1725 8.15e-06 TTTATATACC TGCCGGTGTGA TTTTTTTTTT*
PF14_0207; 598 9.86e-06 TGGGTAAAAT TGTAGGTTTCG TTTTTTTTTT
PFB0290c; 164 1.98e-05 GGAGTTATAT TACGTTTTTCC TTTTTTAAAT
PF14_0469; 1037 2.18e-05 TTGAATTTAT TGTGGTTACAA AATTATACAT
PF10_0269; 1045 2.42e-05 TATTTACACG TGTAGTTATGC TCCTTACATA
PFI1130c; 924 3.25e-05 TTATTATATA TATCCTTCTAC ATTTATGATA
PF14_0241; 299 5.91e-05 TTTAGAATTT TATAGAGCTAC AATAATTAAA
zoops4; weakly related to anr1
PFL0330c; 966 9.40e-08 TAATATTAAT ACACGTGC TTAATTTATA
PFF1390w; 999 9.40e-08 CAAAATAAAT ACACGTGC ATTATATCAT
PF10_0269; 310 2.72e-07 CTTATTTACA ACACGCAC TATAAAAAAA
PFC0805w; 3 8.92e-07 AT ACACGTAC AATTTATAAC
PF14_0241; 337 9.75e-07 ATTAAAAATA ACATGCGC TAAATATTAA
PF13_0150; 1681 2.66e-06 TTTCAATGTG ACACGTGT CCGTAAAATT*
PF11_0264; 1032 2.66e-06 TTTTTTTTCA CCACGTCG TGTATAAAAT*
PFC0155c; 868 3.21e-06 TTATATTTTT CTACGTGC AAAAAAATTA
PFA0505c; 693 5.30e-06 TATTTTTATT ACATGCAC TCTAAAAATA
PF14_0469; 638 8.70e-06 AAAAATGAAA ACGCGTCT AGTGAGAGAA*
PF13_0023; 312 1.29e-05 AGAAAAATTA ACATGTAC AAAAATTACT
PF11_0445; 341 1.86e-05 AAAATTGATA ACACGTAT ATTTATTTTT
PF11_0358; 238 2.04e-05 TTTAAATAAT ACTTGTGC CATTAAAGAG
PF13_0341; 651 2.62e-05 ATATATTATT ATACGTAC AATTTCTTTT
5

## Slide 6
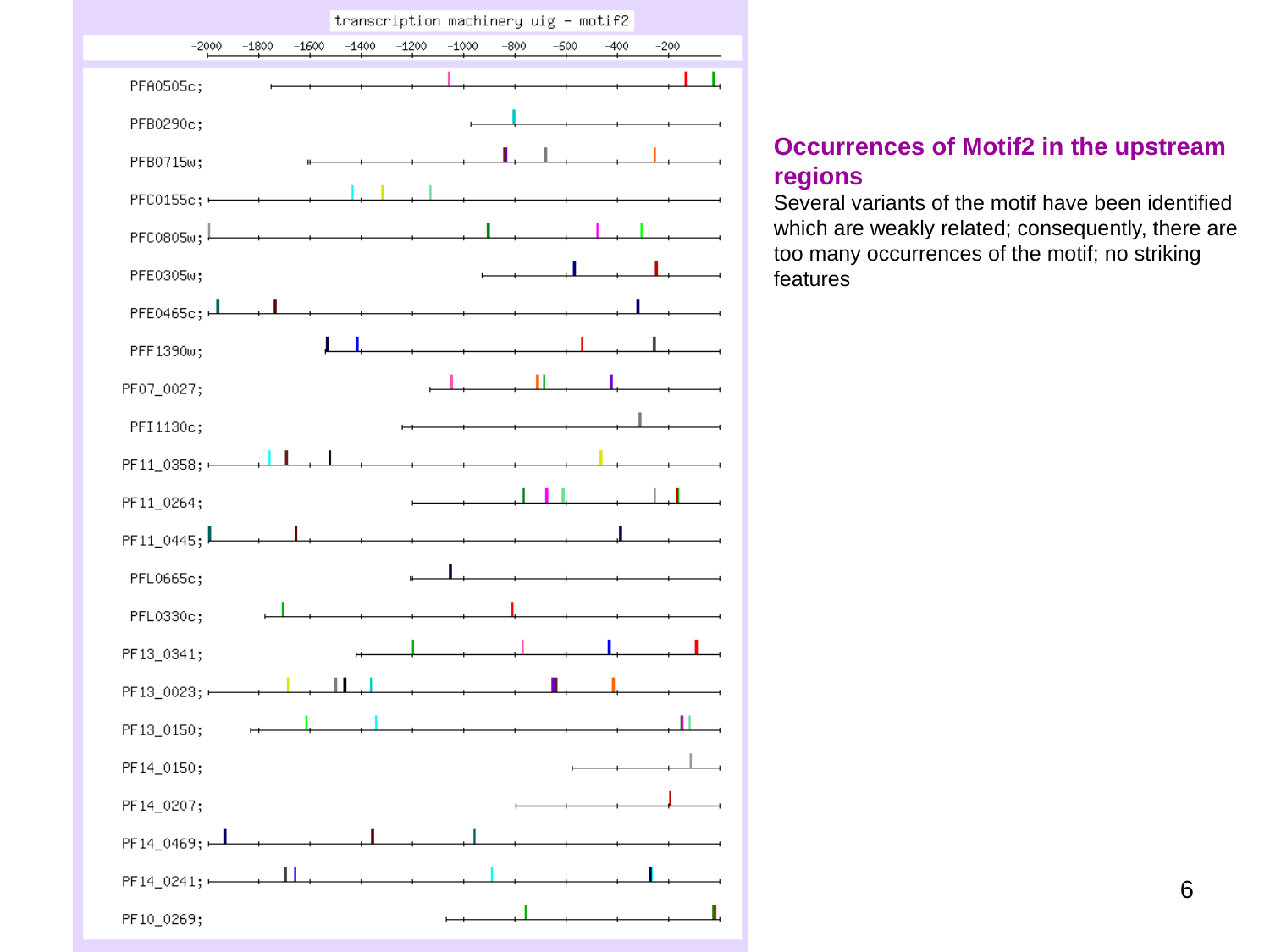

Occurrences of Motif2 in the upstream
regions
Several variants of the motif have been identified
which are weakly related; consequently, there are
too many occurrences of the motif; no striking
features
6

## Slide 7
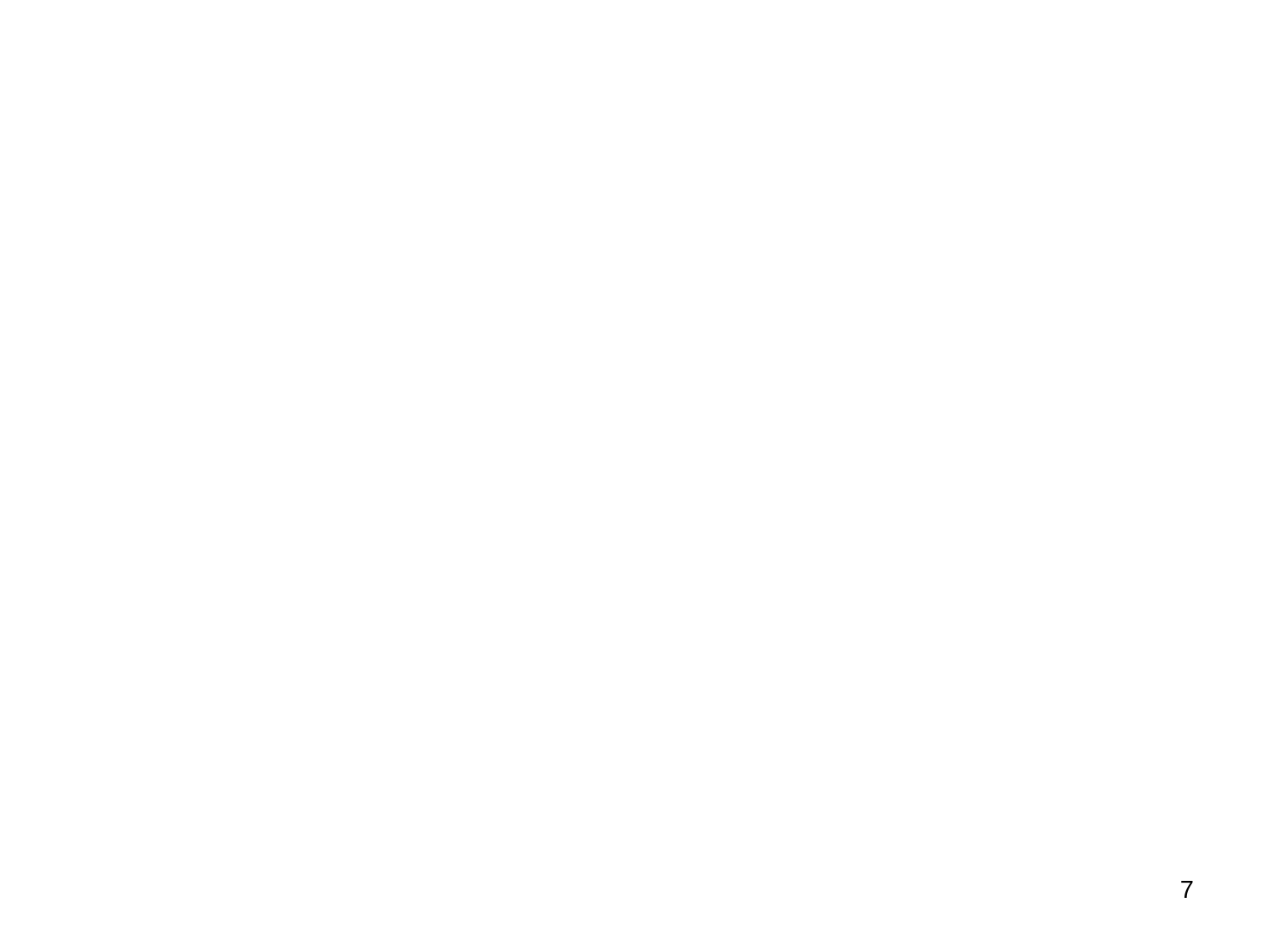

7

## Slide 8
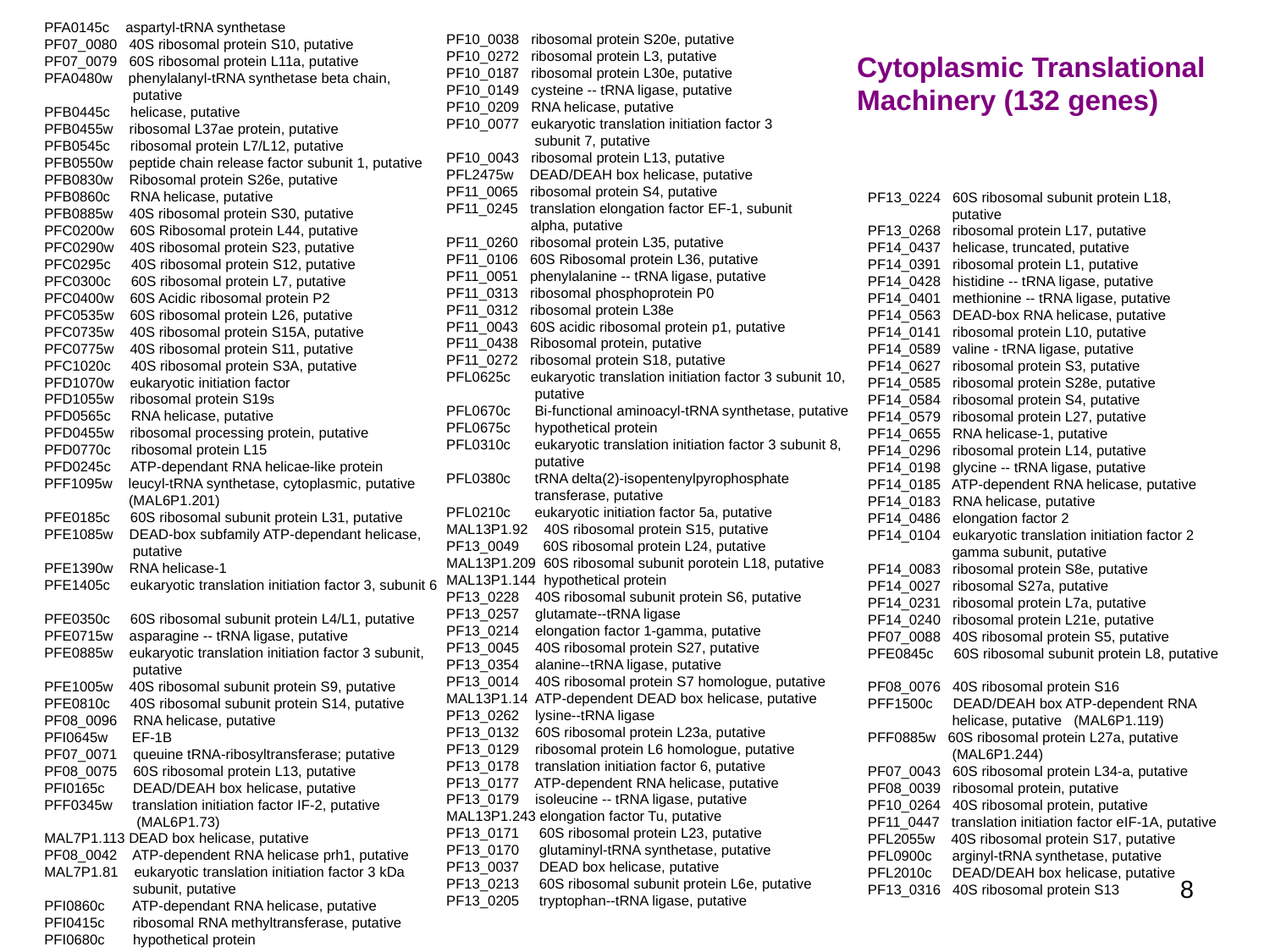

PFA0145c aspartyl-tRNA synthetase
PF07_0080 40S ribosomal protein S10, putative
PF07_0079 60S ribosomal protein L11a, putative
PFA0480w phenylalanyl-tRNA synthetase beta chain,
 putative
PFB0445c helicase, putative
PFB0455w ribosomal L37ae protein, putative
PFB0545c ribosomal protein L7/L12, putative
PFB0550w peptide chain release factor subunit 1, putative
PFB0830w Ribosomal protein S26e, putative
PFB0860c RNA helicase, putative
PFB0885w 40S ribosomal protein S30, putative
PFC0200w 60S Ribosomal protein L44, putative
PFC0290w 40S ribosomal protein S23, putative
PFC0295c 40S ribosomal protein S12, putative
PFC0300c 60S ribosomal protein L7, putative
PFC0400w 60S Acidic ribosomal protein P2
PFC0535w 60S ribosomal protein L26, putative
PFC0735w 40S ribosomal protein S15A, putative
PFC0775w 40S ribosomal protein S11, putative
PFC1020c 40S ribosomal protein S3A, putative
PFD1070w eukaryotic initiation factor
PFD1055w ribosomal protein S19s
PFD0565c RNA helicase, putative
PFD0455w ribosomal processing protein, putative
PFD0770c ribosomal protein L15
PFD0245c ATP-dependant RNA helicae-like protein
PFF1095w leucyl-tRNA synthetase, cytoplasmic, putative
 (MAL6P1.201)
PFE0185c 60S ribosomal subunit protein L31, putative
PFE1085w DEAD-box subfamily ATP-dependant helicase,
 putative
PFE1390w RNA helicase-1
PFE1405c eukaryotic translation initiation factor 3, subunit 6
PFE0350c 60S ribosomal subunit protein L4/L1, putative
PFE0715w asparagine -- tRNA ligase, putative
PFE0885w eukaryotic translation initiation factor 3 subunit,
 putative
PFE1005w 40S ribosomal subunit protein S9, putative
PFE0810c 40S ribosomal subunit protein S14, putative
PF08_0096 RNA helicase, putative
PFI0645w EF-1B
PF07_0071 queuine tRNA-ribosyltransferase; putative
PF08_0075 60S ribosomal protein L13, putative
PFI0165c DEAD/DEAH box helicase, putative
PFF0345w translation initiation factor IF-2, putative
 (MAL6P1.73)
MAL7P1.113 DEAD box helicase, putative
PF08_0042 ATP-dependent RNA helicase prh1, putative
MAL7P1.81 eukaryotic translation initiation factor 3 kDa
 subunit, putative
PFI0860c ATP-dependant RNA helicase, putative
PFI0415c ribosomal RNA methyltransferase, putative
PFI0680c hypothetical protein
PF10_0038 ribosomal protein S20e, putative
PF10_0272 ribosomal protein L3, putative
PF10_0187 ribosomal protein L30e, putative
PF10_0149 cysteine -- tRNA ligase, putative
PF10_0209 RNA helicase, putative
PF10_0077 eukaryotic translation initiation factor 3
 subunit 7, putative
PF10_0043 ribosomal protein L13, putative
PFL2475w DEAD/DEAH box helicase, putative
PF11_0065 ribosomal protein S4, putative
PF11_0245 translation elongation factor EF-1, subunit
 alpha, putative
PF11_0260 ribosomal protein L35, putative
PF11_0106 60S Ribosomal protein L36, putative
PF11_0051 phenylalanine -- tRNA ligase, putative
PF11_0313 ribosomal phosphoprotein P0
PF11_0312 ribosomal protein L38e
PF11_0043 60S acidic ribosomal protein p1, putative
PF11_0438 Ribosomal protein, putative
PF11_0272 ribosomal protein S18, putative
PFL0625c eukaryotic translation initiation factor 3 subunit 10,
 putative
PFL0670c Bi-functional aminoacyl-tRNA synthetase, putative
PFL0675c hypothetical protein
PFL0310c eukaryotic translation initiation factor 3 subunit 8,
 putative
PFL0380c tRNA delta(2)-isopentenylpyrophosphate
 transferase, putative
PFL0210c eukaryotic initiation factor 5a, putative
MAL13P1.92 40S ribosomal protein S15, putative
PF13_0049 60S ribosomal protein L24, putative
MAL13P1.209 60S ribosomal subunit porotein L18, putative
MAL13P1.144 hypothetical protein
PF13_0228 40S ribosomal subunit protein S6, putative
PF13_0257 glutamate--tRNA ligase
PF13_0214 elongation factor 1-gamma, putative
PF13_0045 40S ribosomal protein S27, putative
PF13_0354 alanine--tRNA ligase, putative
PF13_0014 40S ribosomal protein S7 homologue, putative
MAL13P1.14 ATP-dependent DEAD box helicase, putative
PF13_0262 lysine--tRNA ligase
PF13_0132 60S ribosomal protein L23a, putative
PF13_0129 ribosomal protein L6 homologue, putative
PF13_0178 translation initiation factor 6, putative
PF13_0177 ATP-dependent RNA helicase, putative
PF13_0179 isoleucine -- tRNA ligase, putative
MAL13P1.243 elongation factor Tu, putative
PF13_0171 60S ribosomal protein L23, putative
PF13_0170 glutaminyl-tRNA synthetase, putative
PF13_0037 DEAD box helicase, putative
PF13_0213 60S ribosomal subunit protein L6e, putative
PF13_0205 tryptophan--tRNA ligase, putative
Cytoplasmic Translational
Machinery (132 genes)
PF13_0224 60S ribosomal subunit protein L18,
 putative
PF13_0268 ribosomal protein L17, putative
PF14_0437 helicase, truncated, putative
PF14_0391 ribosomal protein L1, putative
PF14_0428 histidine -- tRNA ligase, putative
PF14_0401 methionine -- tRNA ligase, putative
PF14_0563 DEAD-box RNA helicase, putative
PF14_0141 ribosomal protein L10, putative
PF14_0589 valine - tRNA ligase, putative
PF14_0627 ribosomal protein S3, putative
PF14_0585 ribosomal protein S28e, putative
PF14_0584 ribosomal protein S4, putative
PF14_0579 ribosomal protein L27, putative
PF14_0655 RNA helicase-1, putative
PF14_0296 ribosomal protein L14, putative
PF14_0198 glycine -- tRNA ligase, putative
PF14_0185 ATP-dependent RNA helicase, putative
PF14_0183 RNA helicase, putative
PF14_0486 elongation factor 2
PF14_0104 eukaryotic translation initiation factor 2
 gamma subunit, putative
PF14_0083 ribosomal protein S8e, putative
PF14_0027 ribosomal S27a, putative
PF14_0231 ribosomal protein L7a, putative
PF14_0240 ribosomal protein L21e, putative
PF07_0088 40S ribosomal protein S5, putative
PFE0845c 60S ribosomal subunit protein L8, putative
PF08_0076 40S ribosomal protein S16
PFF1500c DEAD/DEAH box ATP-dependent RNA
 helicase, putative (MAL6P1.119)
PFF0885w 60S ribosomal protein L27a, putative
 (MAL6P1.244)
PF07_0043 60S ribosomal protein L34-a, putative
PF08_0039 ribosomal protein, putative
PF10_0264 40S ribosomal protein, putative
PF11_0447 translation initiation factor eIF-1A, putative
PFL2055w 40S ribosomal protein S17, putative
PFL0900c arginyl-tRNA synthetase, putative
PFL2010c DEAD/DEAH box helicase, putative
PF13_0316 40S ribosomal protein S13
8

## Slide 9
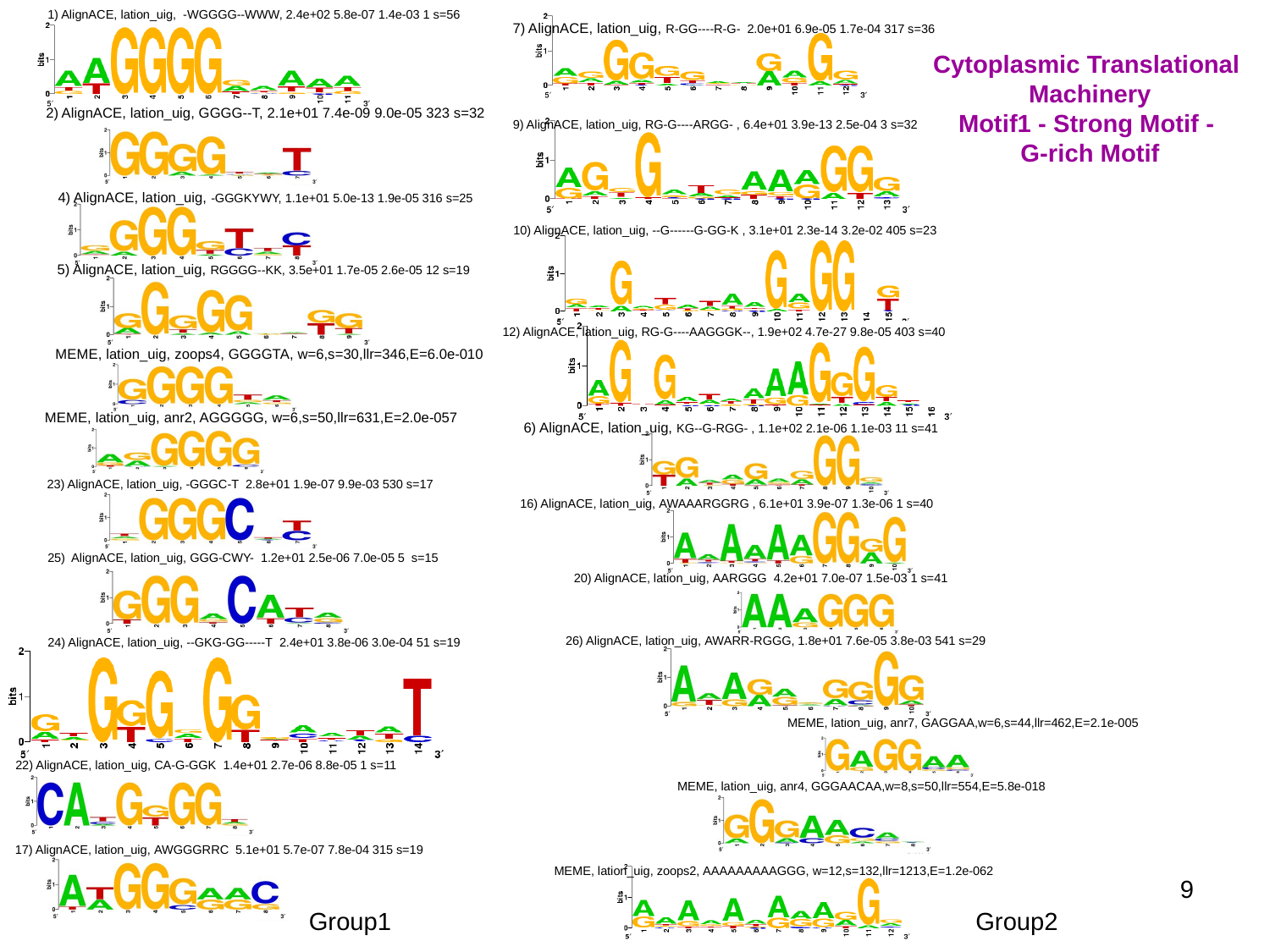

1) AlignACE, lation_uig, -WGGGG--WWW, 2.4e+02 5.8e-07 1.4e-03 1 s=56
2) AlignACE, lation_uig, GGGG--T, 2.1e+01 7.4e-09 9.0e-05 323 s=32
4) AlignACE, lation_uig, -GGGKYWY, 1.1e+01 5.0e-13 1.9e-05 316 s=25
5) AlignACE, lation_uig, RGGGG--KK, 3.5e+01 1.7e-05 2.6e-05 12 s=19
MEME, lation_uig, zoops4, GGGGTA, w=6,s=30,llr=346,E=6.0e-010
MEME, lation_uig, anr2, AGGGGG, w=6,s=50,llr=631,E=2.0e-057
23) AlignACE, lation_uig, -GGGC-T 2.8e+01 1.9e-07 9.9e-03 530 s=17
25) AlignACE, lation_uig, GGG-CWY- 1.2e+01 2.5e-06 7.0e-05 5 s=15
24) AlignACE, lation_uig, --GKG-GG-----T 2.4e+01 3.8e-06 3.0e-04 51 s=19
22) AlignACE, lation_uig, CA-G-GGK 1.4e+01 2.7e-06 8.8e-05 1 s=11
17) AlignACE, lation_uig, AWGGGRRC 5.1e+01 5.7e-07 7.8e-04 315 s=19
7) AlignACE, lation_uig, R-GG----R-G- 2.0e+01 6.9e-05 1.7e-04 317 s=36
9) AlignACE, lation_uig, RG-G----ARGG- , 6.4e+01 3.9e-13 2.5e-04 3 s=32
10) AlignACE, lation_uig, --G------G-GG-K , 3.1e+01 2.3e-14 3.2e-02 405 s=23
12) AlignACE, lation_uig, RG-G----AAGGGK--, 1.9e+02 4.7e-27 9.8e-05 403 s=40
6) AlignACE, lation_uig, KG--G-RGG- , 1.1e+02 2.1e-06 1.1e-03 11 s=41
16) AlignACE, lation_uig, AWAAARGGRG , 6.1e+01 3.9e-07 1.3e-06 1 s=40
20) AlignACE, lation_uig, AARGGG 4.2e+01 7.0e-07 1.5e-03 1 s=41
26) AlignACE, lation_uig, AWARR-RGGG, 1.8e+01 7.6e-05 3.8e-03 541 s=29
MEME, lation_uig, anr7, GAGGAA,w=6,s=44,llr=462,E=2.1e-005
MEME, lation_uig, anr4, GGGAACAA,w=8,s=50,llr=554,E=5.8e-018
MEME, lation_uig, zoops2, AAAAAAAAAGGG, w=12,s=132,llr=1213,E=1.2e-062
Cytoplasmic Translational
Machinery
Motif1 - Strong Motif -
G-rich Motif
9
Group1
Group2

## Slide 10
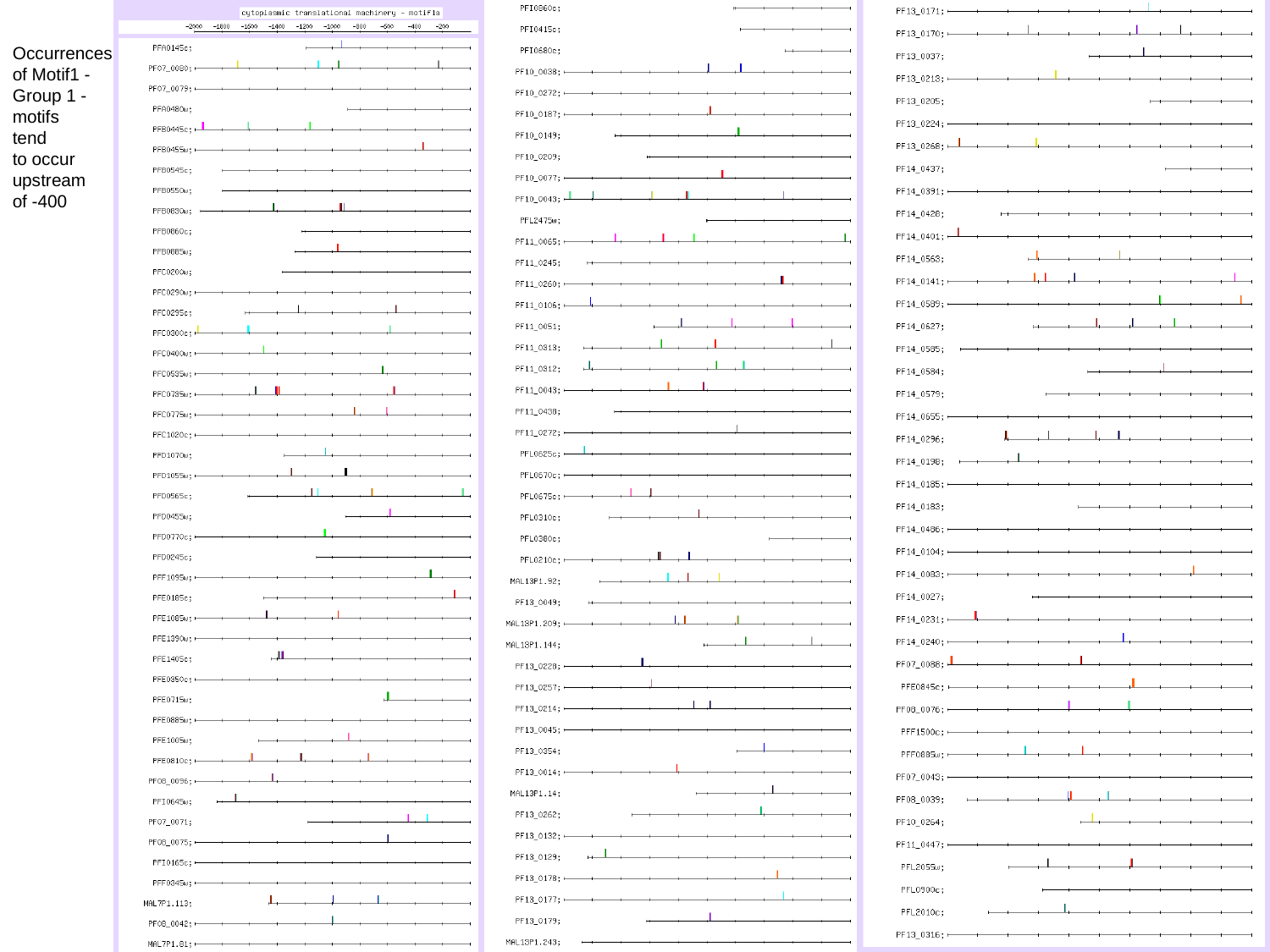

Occurrences
of Motif1 -
Group 1 -
motifs
tend
to occur
upstream
of -400
10

## Slide 11
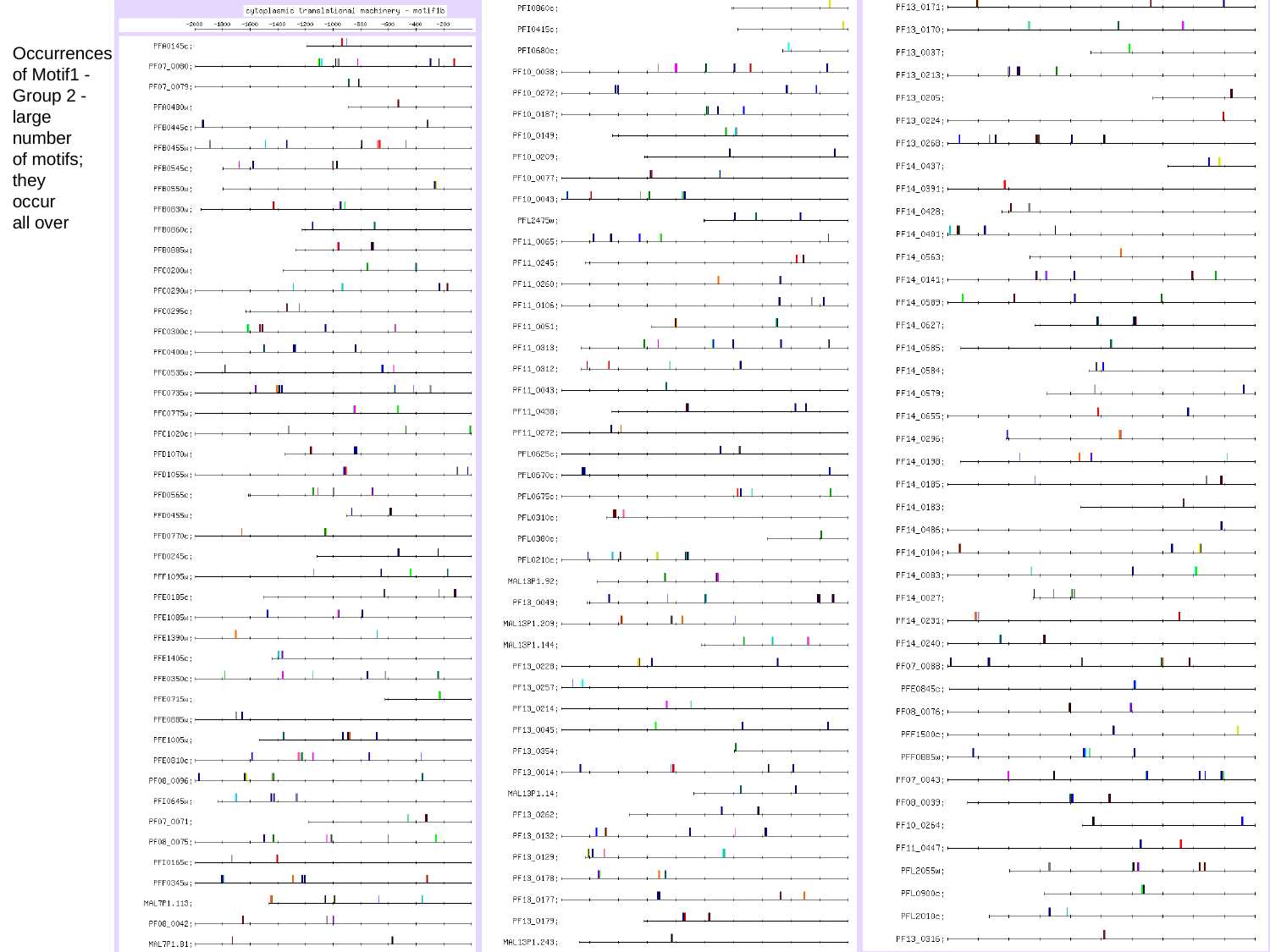

Occurrences
of Motif1 -
Group 2 -
large
number
of motifs;
they
occur
all over
11

## Slide 12
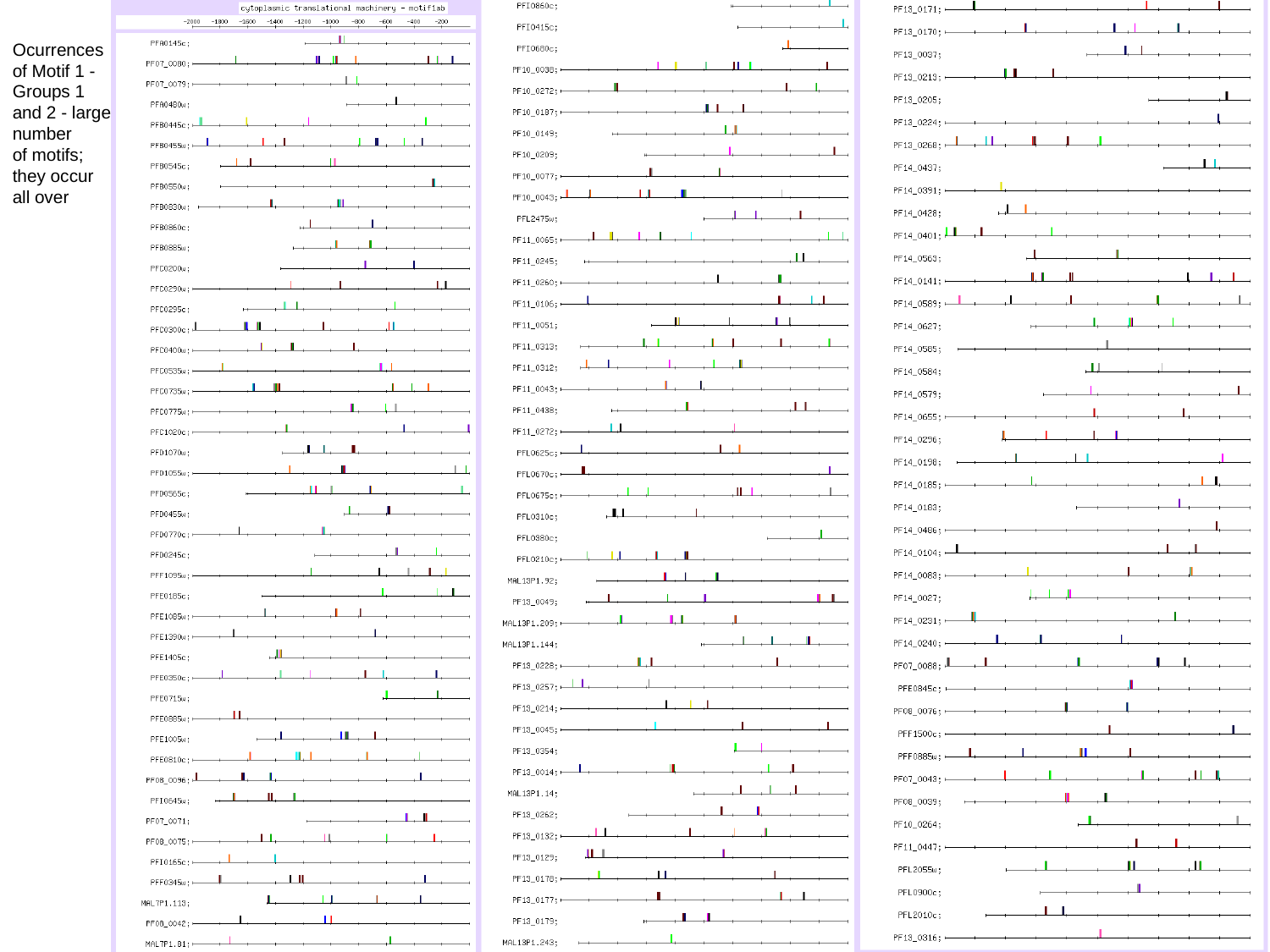

Ocurrences of Motif 1 -
Groups 1 and 2 - large
number
of motifs;
they occur
all over
12

## Slide 13
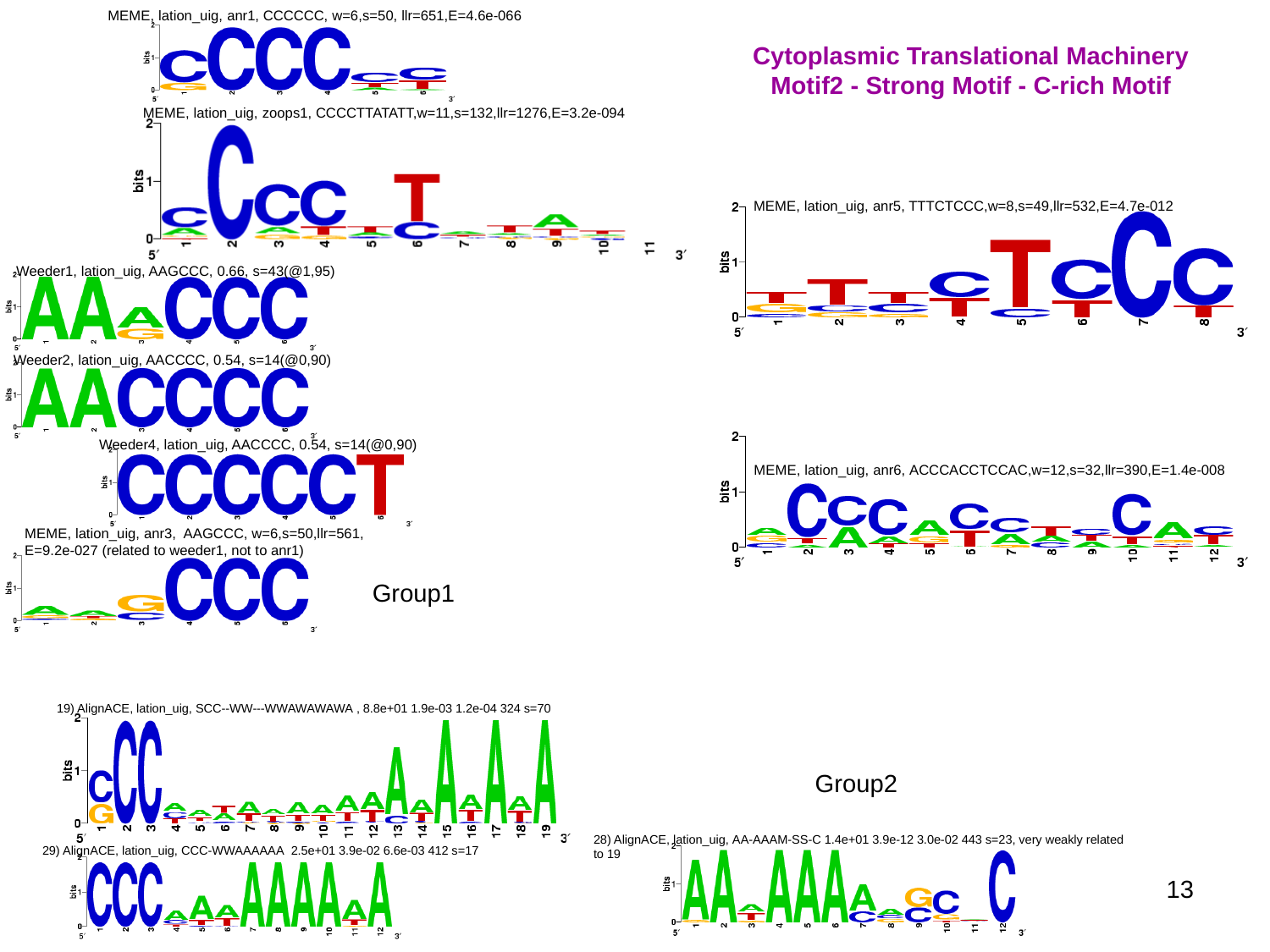

MEME, lation_uig, anr1, CCCCCC, w=6,s=50, llr=651,E=4.6e-066
MEME, lation_uig, zoops1, CCCCTTATATT,w=11,s=132,llr=1276,E=3.2e-094
Weeder1, lation_uig, AAGCCC, 0.66, s=43(@1,95)
Weeder2, lation_uig, AACCCC, 0.54, s=14(@0,90)
Weeder4, lation_uig, AACCCC, 0.54, s=14(@0,90)
MEME, lation_uig, anr3, AAGCCC, w=6,s=50,llr=561,
E=9.2e-027 (related to weeder1, not to anr1)
MEME, lation_uig, anr5, TTTCTCCC,w=8,s=49,llr=532,E=4.7e-012
MEME, lation_uig, anr6, ACCCACCTCCAC,w=12,s=32,llr=390,E=1.4e-008
19) AlignACE, lation_uig, SCC--WW---WWAWAWAWA , 8.8e+01 1.9e-03 1.2e-04 324 s=70
29) AlignACE, lation_uig, CCC-WWAAAAAA 2.5e+01 3.9e-02 6.6e-03 412 s=17
28) AlignACE, lation_uig, AA-AAAM-SS-C 1.4e+01 3.9e-12 3.0e-02 443 s=23, very weakly related
to 19
Cytoplasmic Translational Machinery
Motif2 - Strong Motif - C-rich Motif
Group1
Group2
13

## Slide 14
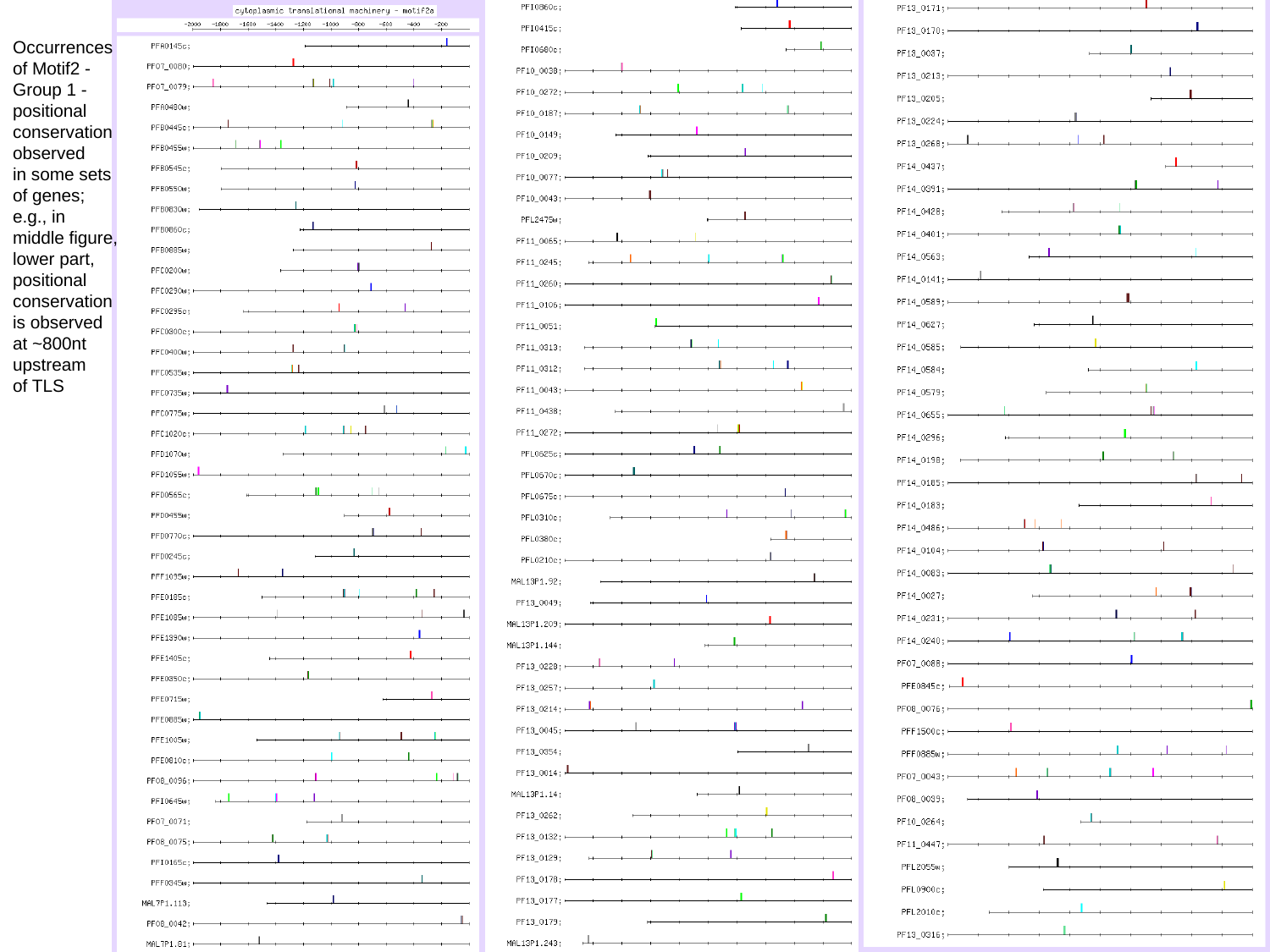

Occurrences
of Motif2 -
Group 1 -
positional
conservation
observed
in some sets
of genes;
e.g., in
middle figure,
lower part,
positional
conservation
is observed
at ~800nt
upstream
of TLS
14

## Slide 15
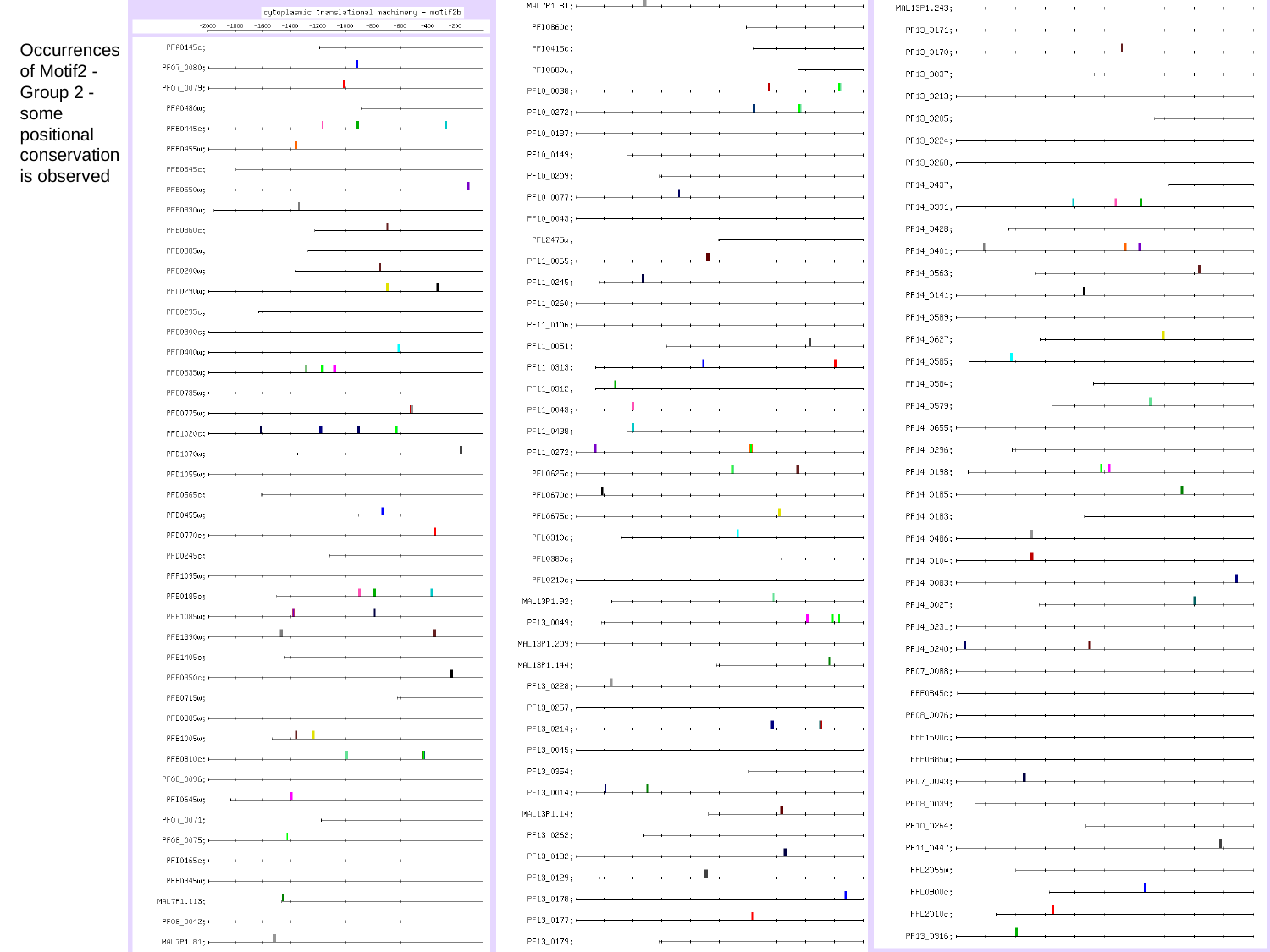

Occurrences
of Motif2 -
Group 2 -
some
positional
conservation
is observed
15

## Slide 16
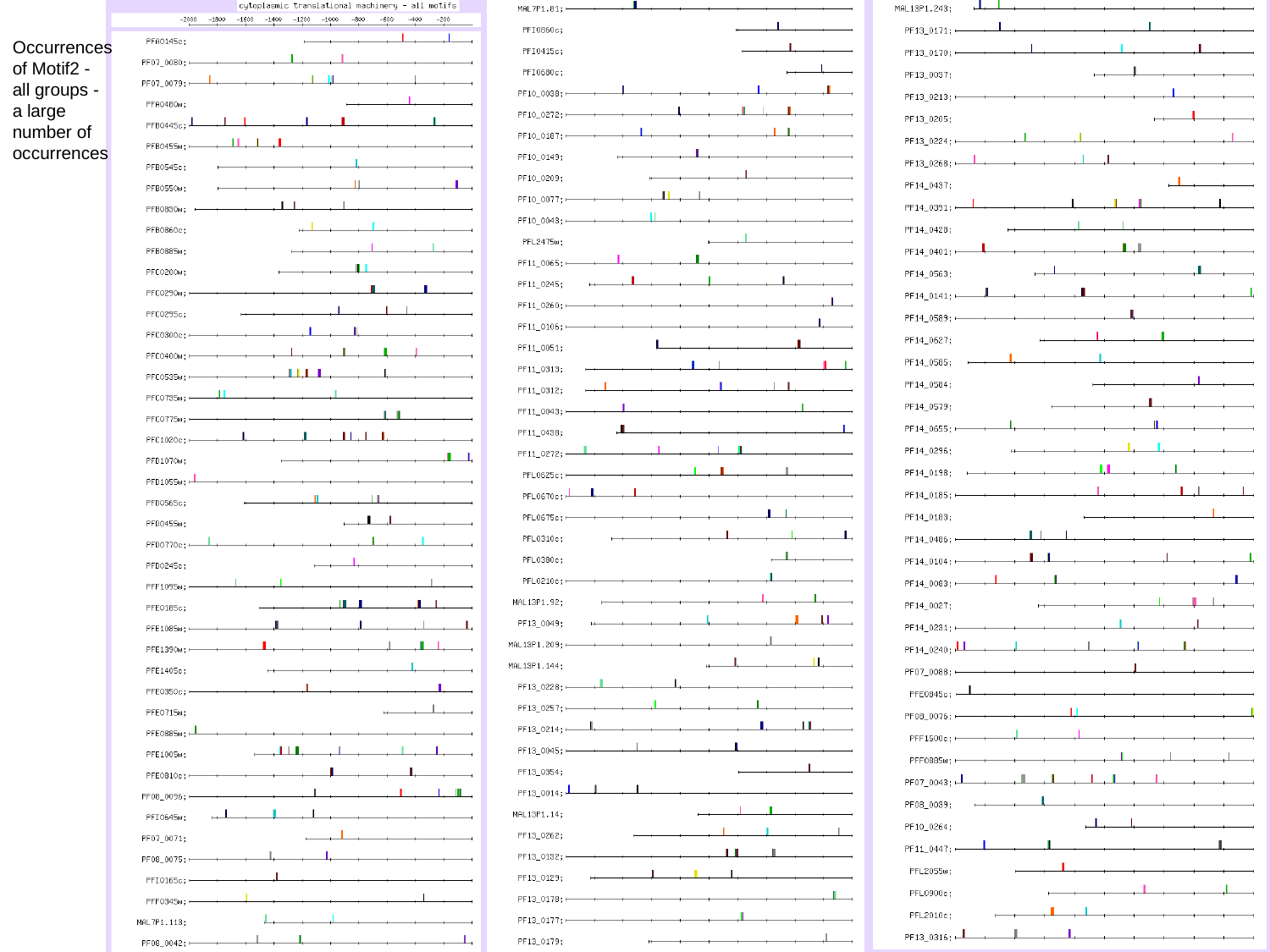

Occurrences
of Motif2 -
all groups -
a large
number of
occurrences
16

## Slide 17
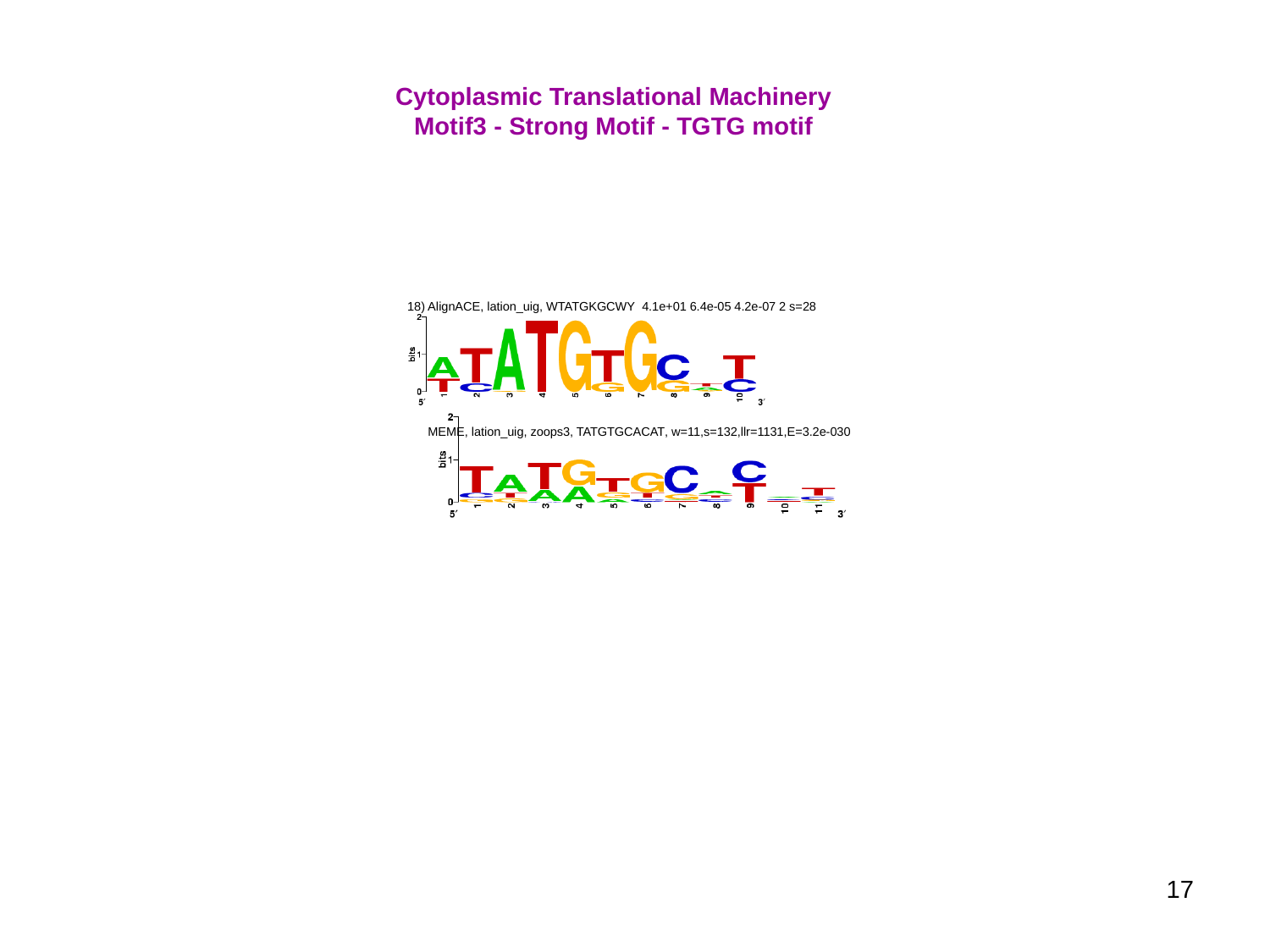

Cytoplasmic Translational Machinery
Motif3 - Strong Motif - TGTG motif
18) AlignACE, lation_uig, WTATGKGCWY 4.1e+01 6.4e-05 4.2e-07 2 s=28
MEME, lation_uig, zoops3, TATGTGCACAT, w=11,s=132,llr=1131,E=3.2e-030
17

## Slide 18
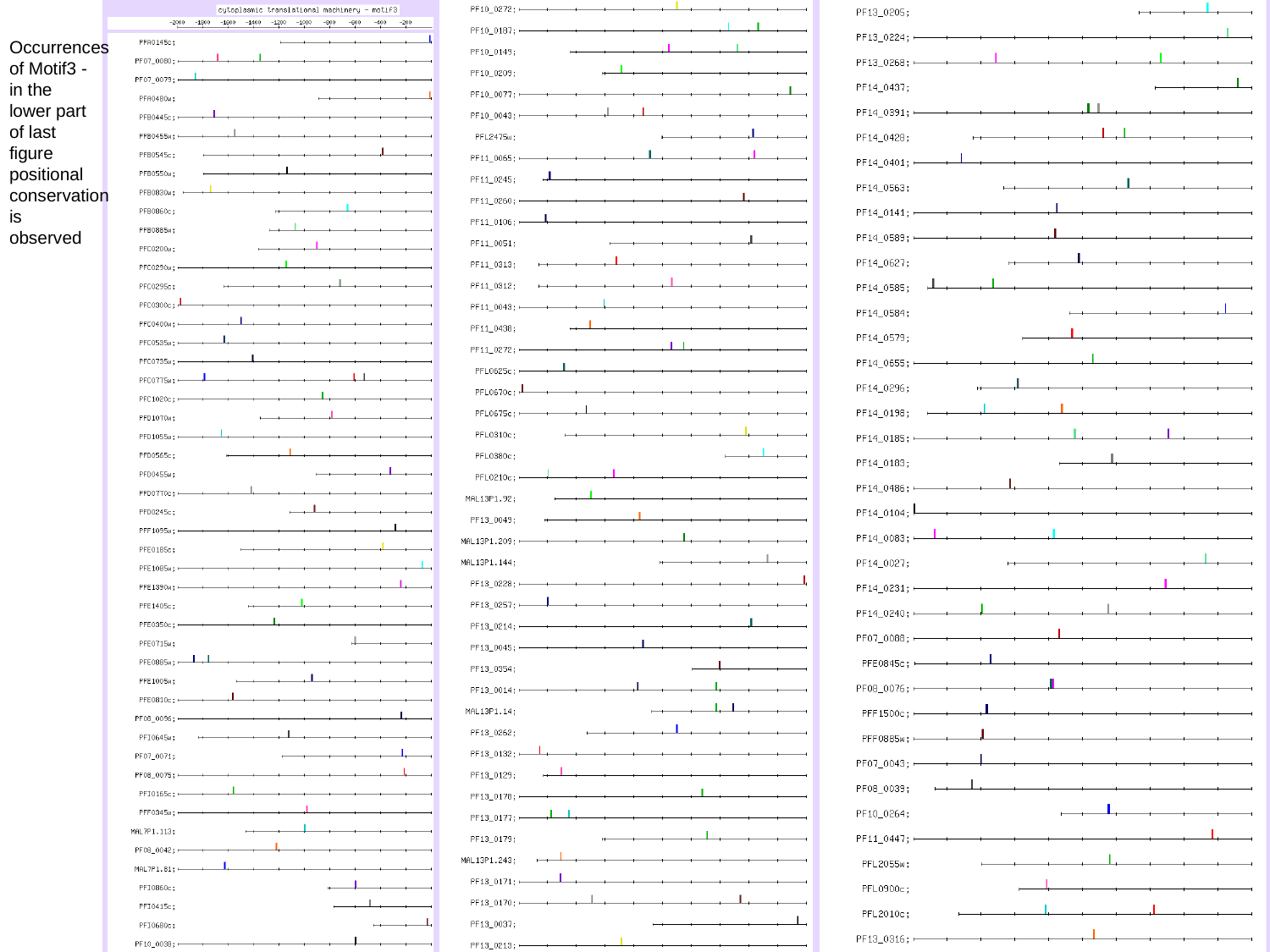

Occurrences
of Motif3 -
in the
lower part
of last
figure
positional
conservation
is
observed
18

## Slide 19
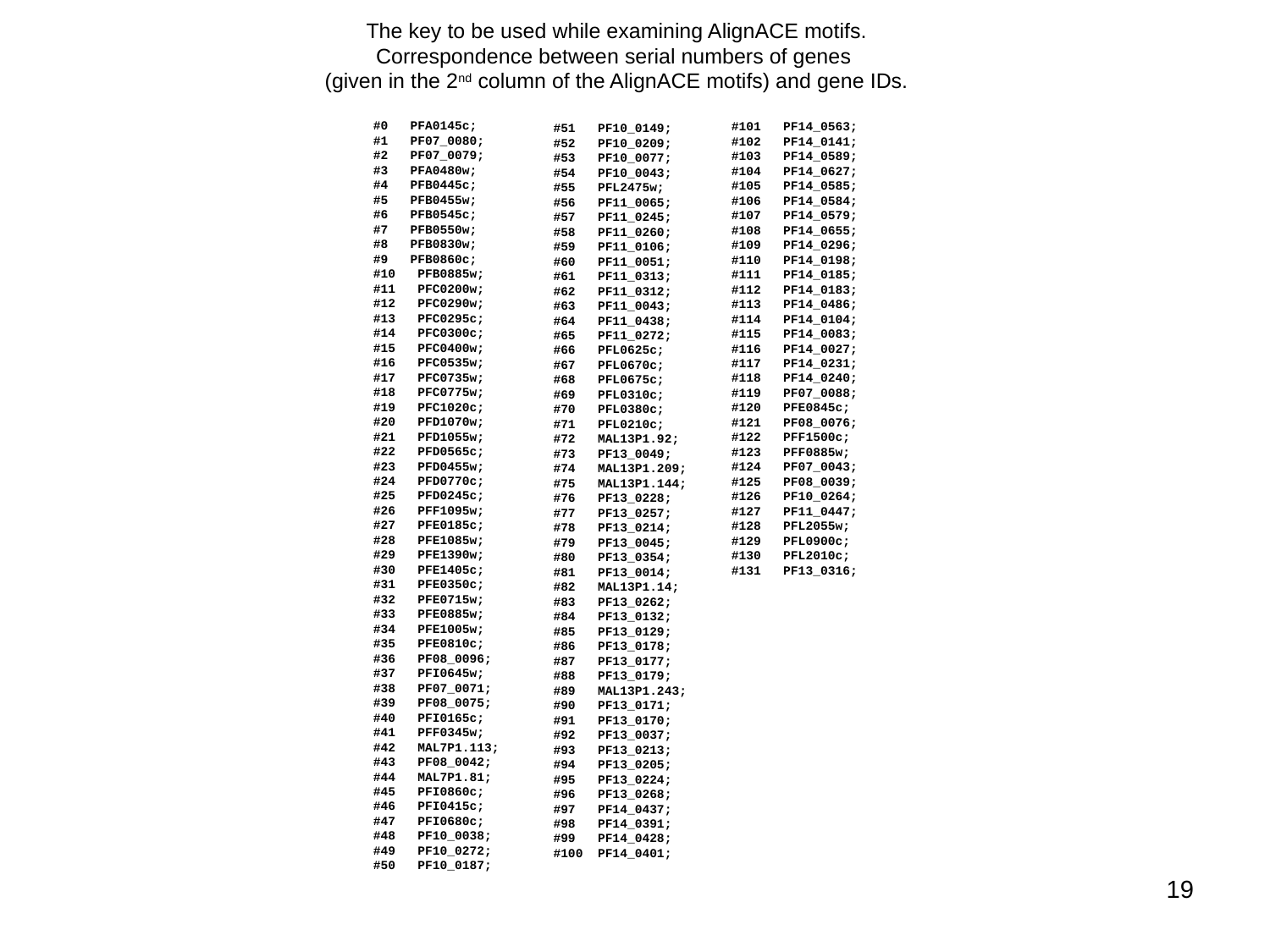

The key to be used while examining AlignACE motifs.
Correspondence between serial numbers of genes
(given in the 2nd column of the AlignACE motifs) and gene IDs.
#0 PFA0145c;
#1 PF07_0080;
#2 PF07_0079;
#3 PFA0480w;
#4 PFB0445c;
#5 PFB0455w;
#6 PFB0545c;
#7 PFB0550w;
#8 PFB0830w;
#9 PFB0860c;
#10 PFB0885w;
#11 PFC0200w;
#12 PFC0290w;
#13 PFC0295c;
#14 PFC0300c;
#15 PFC0400w;
#16 PFC0535w;
#17 PFC0735w;
#18 PFC0775w;
#19 PFC1020c;
#20 PFD1070w;
#21 PFD1055w;
#22 PFD0565c;
#23 PFD0455w;
#24 PFD0770c;
#25 PFD0245c;
#26 PFF1095w;
#27 PFE0185c;
#28 PFE1085w;
#29 PFE1390w;
#30 PFE1405c;
#31 PFE0350c;
#32 PFE0715w;
#33 PFE0885w;
#34 PFE1005w;
#35 PFE0810c;
#36 PF08_0096;
#37 PFI0645w;
#38 PF07_0071;
#39 PF08_0075;
#40 PFI0165c;
#41 PFF0345w;
#42 MAL7P1.113;
#43 PF08_0042;
#44 MAL7P1.81;
#45 PFI0860c;
#46 PFI0415c;
#47 PFI0680c;
#48 PF10_0038;
#49 PF10_0272;
#50 PF10_0187;
#101 PF14_0563;
#102 PF14_0141;
#103 PF14_0589;
#104 PF14_0627;
#105 PF14_0585;
#106 PF14_0584;
#107 PF14_0579;
#108 PF14_0655;
#109 PF14_0296;
#110 PF14_0198;
#111 PF14_0185;
#112 PF14_0183;
#113 PF14_0486;
#114 PF14_0104;
#115 PF14_0083;
#116 PF14_0027;
#117 PF14_0231;
#118 PF14_0240;
#119 PF07_0088;
#120 PFE0845c;
#121 PF08_0076;
#122 PFF1500c;
#123 PFF0885w;
#124 PF07_0043;
#125 PF08_0039;
#126 PF10_0264;
#127 PF11_0447;
#128 PFL2055w;
#129 PFL0900c;
#130 PFL2010c;
#131 PF13_0316;
#51 PF10_0149;
#52 PF10_0209;
#53 PF10_0077;
#54 PF10_0043;
#55 PFL2475w;
#56 PF11_0065;
#57 PF11_0245;
#58 PF11_0260;
#59 PF11_0106;
#60 PF11_0051;
#61 PF11_0313;
#62 PF11_0312;
#63 PF11_0043;
#64 PF11_0438;
#65 PF11_0272;
#66 PFL0625c;
#67 PFL0670c;
#68 PFL0675c;
#69 PFL0310c;
#70 PFL0380c;
#71 PFL0210c;
#72 MAL13P1.92;
#73 PF13_0049;
#74 MAL13P1.209;
#75 MAL13P1.144;
#76 PF13_0228;
#77 PF13_0257;
#78 PF13_0214;
#79 PF13_0045;
#80 PF13_0354;
#81 PF13_0014;
#82 MAL13P1.14;
#83 PF13_0262;
#84 PF13_0132;
#85 PF13_0129;
#86 PF13_0178;
#87 PF13_0177;
#88 PF13_0179;
#89 MAL13P1.243;
#90 PF13_0171;
#91 PF13_0170;
#92 PF13_0037;
#93 PF13_0213;
#94 PF13_0205;
#95 PF13_0224;
#96 PF13_0268;
#97 PF14_0437;
#98 PF14_0391;
#99 PF14_0428;
#100 PF14_0401;
19

## Slide 20
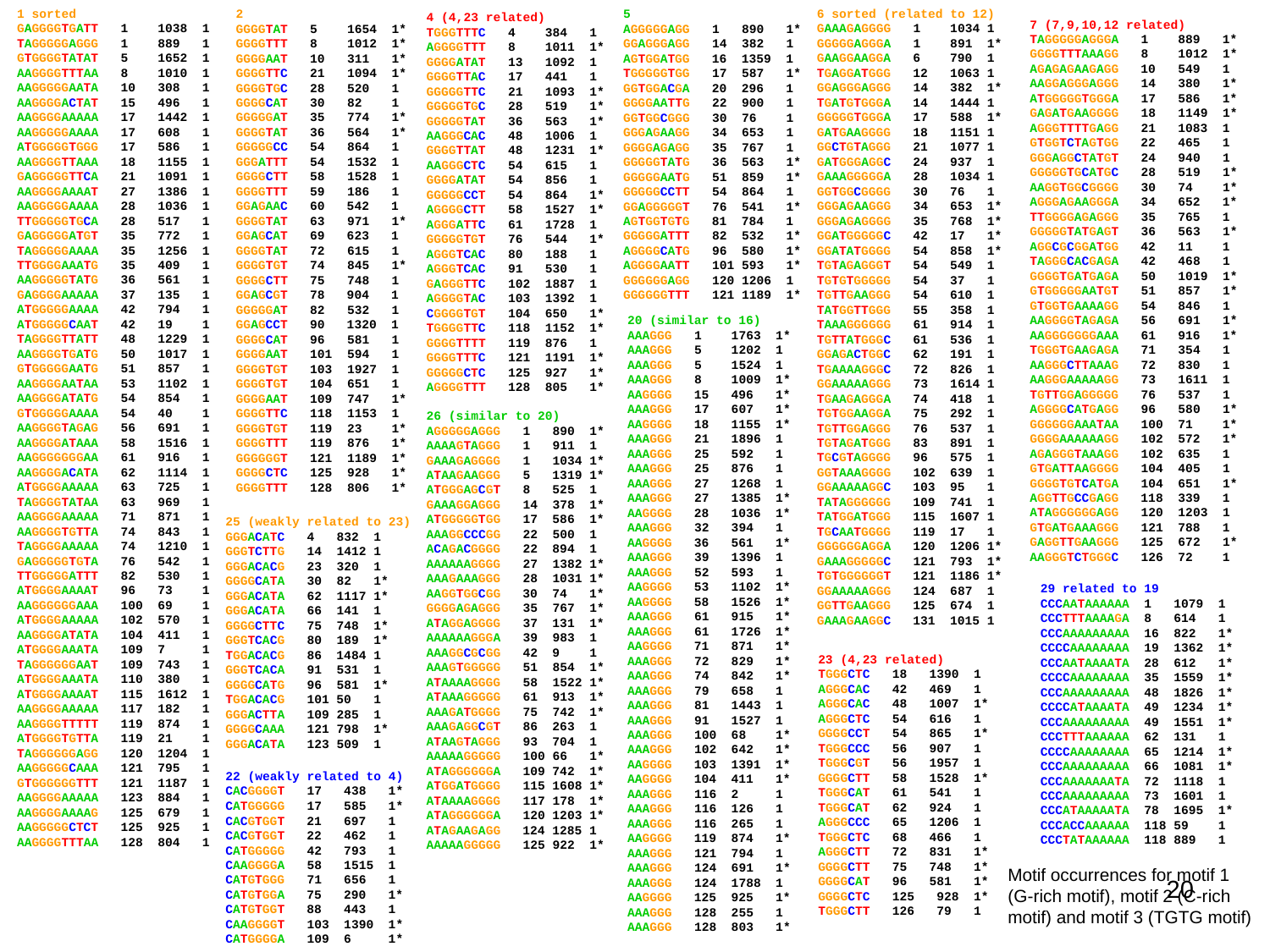

1 sorted
GAGGGGTGATT 1 1038 1
TAGGGGGAGGG 1 889 1
GTGGGGTATAT 5 1652 1
AAGGGGTTTAA 8 1010 1
AAGGGGGAATA 10 308 1
AAGGGGACTAT 15 496 1
AAGGGGAAAAA 17 1442 1
AAGGGGGAAAA 17 608 1
ATGGGGGTGGG 17 586 1
AAGGGGTTAAA 18 1155 1
GAGGGGGTTCA 21 1091 1
AAGGGGAAAAT 27 1386 1
AAGGGGGAAAA 28 1036 1
TTGGGGGTGCA 28 517 1
GAGGGGGATGT 35 772 1
TAGGGGGAAAA 35 1256 1
TTGGGGAAATG 35 409 1
AAGGGGGTATG 36 561 1
GAGGGGAAAAA 37 135 1
ATGGGGGAAAA 42 794 1
ATGGGGGCAAT 42 19 1
TAGGGGTTATT 48 1229 1
AAGGGGTGATG 50 1017 1
GTGGGGGAATG 51 857 1
AAGGGGAATAA 53 1102 1
AAGGGGATATG 54 854 1
GTGGGGGAAAA 54 40 1
AAGGGGTAGAG 56 691 1
AAGGGGATAAA 58 1516 1
AAGGGGGGGAA 61 916 1
AAGGGGACATA 62 1114 1
ATGGGGAAAAA 63 725 1
TAGGGGTATAA 63 969 1
AAGGGGAAAAA 71 871 1
AAGGGGTGTTA 74 843 1
TAGGGGAAAAA 74 1210 1
GAGGGGGTGTA 76 542 1
TTGGGGGATTT 82 530 1
ATGGGGAAAAT 96 73 1
AAGGGGGGAAA 100 69 1
ATGGGGAAAAA 102 570 1
AAGGGGATATA 104 411 1
ATGGGGAAATA 109 7 1
TAGGGGGGAAT 109 743 1
ATGGGGAAATA 110 380 1
ATGGGGAAAAT 115 1612 1
AAGGGGAAAAA 117 182 1
AAGGGGTTTTT 119 874 1
ATGGGGTGTTA 119 21 1
TAGGGGGGAGG 120 1204 1
AAGGGGGCAAA 121 795 1
GTGGGGGGTTT 121 1187 1
AAGGGGAAAAA 123 884 1
AAGGGGAAAAG 125 679 1
AAGGGGGCTCT 125 925 1
AAGGGGTTTAA 128 804 1
6 sorted (related to 12)
GAAAGAGGGG 1 1034 1
GGGGGAGGGA 1 891 1*
GAAGGAAGGA 6 790 1
TGAGGATGGG 12 1063 1
GGAGGGAGGG 14 382 1*
TGATGTGGGA 14 1444 1
GGGGGTGGGA 17 588 1*
GATGAAGGGG 18 1151 1
GGCTGTAGGG 21 1077 1
GATGGGAGGC 24 937 1
GAAAGGGGGA 28 1034 1
GGTGGCGGGG 30 76 1
GGGAGAAGGG 34 653 1*
GGGAGAGGGG 35 768 1*
GGATGGGGGC 42 17 1*
GGATATGGGG 54 858 1*
TGTAGAGGGT 54 549 1
TGTGTGGGGG 54 37 1
TGTTGAAGGG 54 610 1
TATGGTTGGG 55 358 1
TAAAGGGGGG 61 914 1
TGTTATGGGC 61 536 1
GGAGACTGGC 62 191 1
TGAAAAGGGC 72 826 1
GGAAAAAGGG 73 1614 1
TGAAGAGGGA 74 418 1
TGTGGAAGGA 75 292 1
TGTTGGAGGG 76 537 1
TGTAGATGGG 83 891 1
TGCGTAGGGG 96 575 1
GGTAAAGGGG 102 639 1
GGAAAAAGGC 103 95 1
TATAGGGGGG 109 741 1
TATGGATGGG 115 1607 1
TGCAATGGGG 119 17 1
GGGGGGAGGA 120 1206 1*
GAAAGGGGGC 121 793 1*
TGTGGGGGGT 121 1186 1*
GGAAAAAGGG 124 687 1
GGTTGAAGGG 125 674 1
GAAAGAAGGC 131 1015 1
2
GGGGTAT 5 1654 1*
GGGGTTT 8 1012 1*
GGGGAAT 10 311 1*
GGGGTTC 21 1094 1*
GGGGTGC 28 520 1
GGGGCAT 30 82 1
GGGGGAT 35 774 1*
GGGGTAT 36 564 1*
GGGGGCC 54 864 1
GGGATTT 54 1532 1
GGGGCTT 58 1528 1
GGGGTTT 59 186 1
GGAGAAC 60 542 1
GGGGTAT 63 971 1*
GGAGCAT 69 623 1
GGGGTAT 72 615 1
GGGGTGT 74 845 1*
GGGGCTT 75 748 1
GGAGCGT 78 904 1
GGGGGAT 82 532 1
GGAGCCT 90 1320 1
GGGGCAT 96 581 1
GGGGAAT 101 594 1
GGGGTGT 103 1927 1
GGGGTGT 104 651 1
GGGGAAT 109 747 1*
GGGGTTC 118 1153 1
GGGGTGT 119 23 1*
GGGGTTT 119 876 1*
GGGGGGT 121 1189 1*
GGGGCTC 125 928 1*
GGGGTTT 128 806 1*
5
AGGGGGAGG 1 890 1*
GGAGGGAGG 14 382 1
AGTGGATGG 16 1359 1
TGGGGGTGG 17 587 1*
GGTGGACGA 20 296 1
GGGGAATTG 22 900 1
GGTGGCGGG 30 76 1
GGGAGAAGG 34 653 1
GGGGAGAGG 35 767 1
GGGGGTATG 36 563 1*
GGGGGAATG 51 859 1*
GGGGGCCTT 54 864 1
GGAGGGGGT 76 541 1*
AGTGGTGTG 81 784 1
GGGGGATTT 82 532 1*
AGGGGCATG 96 580 1*
AGGGGAATT 101 593 1*
GGGGGGAGG 120 1206 1
GGGGGGTTT 121 1189 1*
4 (4,23 related)
TGGGTTTC 4 384 1
AGGGGTTT 8 1011 1*
GGGGATAT 13 1092 1
GGGGTTAC 17 441 1
GGGGGTTC 21 1093 1*
GGGGGTGC 28 519 1*
GGGGGTAT 36 563 1*
AAGGGCAC 48 1006 1
GGGGTTAT 48 1231 1*
AAGGGCTC 54 615 1
GGGGATAT 54 856 1
GGGGGCCT 54 864 1*
AGGGGCTT 58 1527 1*
AGGGATTC 61 1728 1
GGGGGTGT 76 544 1*
AGGGTCAC 80 188 1
AGGGTCAC 91 530 1
GAGGGTTC 102 1887 1
AGGGGTAC 103 1392 1
CGGGGTGT 104 650 1*
TGGGGTTC 118 1152 1*
GGGGTTTT 119 876 1
GGGGTTTC 121 1191 1*
GGGGGCTC 125 927 1*
AGGGGTTT 128 805 1*
7 (7,9,10,12 related)
TAGGGGGAGGGA 1 889 1*
GGGGTTTAAAGG 8 1012 1*
AGAGAGAAGAGG 10 549 1
AAGGAGGGAGGG 14 380 1*
ATGGGGGTGGGA 17 586 1*
GAGATGAAGGGG 18 1149 1*
AGGGTTTTGAGG 21 1083 1
GTGGTCTAGTGG 22 465 1
GGGAGGCTATGT 24 940 1
GGGGGTGCATGC 28 519 1*
AAGGTGGCGGGG 30 74 1*
AGGGAGAAGGGA 34 652 1*
TTGGGGAGAGGG 35 765 1
GGGGGTATGAGT 36 563 1*
AGGCGCGGATGG 42 11 1
TAGGGCACGAGA 42 468 1
GGGGTGATGAGA 50 1019 1*
GTGGGGGAATGT 51 857 1*
GTGGTGAAAAGG 54 846 1
AAGGGGTAGAGA 56 691 1*
AAGGGGGGGAAA 61 916 1*
TGGGTGAAGAGA 71 354 1
AAGGGCTTAAAG 72 830 1
AAGGGAAAAAGG 73 1611 1
TGTTGGAGGGGG 76 537 1
AGGGGCATGAGG 96 580 1*
GGGGGGAAATAA 100 71 1*
GGGGAAAAAAGG 102 572 1*
AGAGGGTAAAGG 102 635 1
GTGATTAAGGGG 104 405 1
GGGGTGTCATGA 104 651 1*
AGGTTGCCGAGG 118 339 1
ATAGGGGGGAGG 120 1203 1
GTGATGAAAGGG 121 788 1
GAGGTTGAAGGG 125 672 1*
AAGGGTCTGGGC 126 72 1
20 (similar to 16)
AAAGGG 1 1763 1*
AAAGGG 5 1202 1
AAAGGG 5 1524 1
AAAGGG 8 1009 1*
AAGGGG 15 496 1*
AAAGGG 17 607 1*
AAGGGG 18 1155 1*
AAAGGG 21 1896 1
AAAGGG 25 592 1
AAAGGG 25 876 1
AAAGGG 27 1268 1
AAAGGG 27 1385 1*
AAGGGG 28 1036 1*
AAAGGG 32 394 1
AAGGGG 36 561 1*
AAAGGG 39 1396 1
AAAGGG 52 593 1
AAGGGG 53 1102 1*
AAGGGG 58 1526 1*
AAAGGG 61 915 1*
AAAGGG 61 1726 1*
AAGGGG 71 871 1*
AAAGGG 72 829 1*
AAAGGG 74 842 1*
AAAGGG 79 658 1
AAAGGG 81 1443 1
AAAGGG 91 1527 1
AAAGGG 100 68 1*
AAAGGG 102 642 1*
AAGGGG 103 1391 1*
AAGGGG 104 411 1*
AAAGGG 116 2 1
AAAGGG 116 126 1
AAAGGG 116 265 1
AAGGGG 119 874 1*
AAAGGG 121 794 1
AAAGGG 124 691 1*
AAAGGG 124 1788 1
AAGGGG 125 925 1*
AAAGGG 128 255 1
AAAGGG 128 803 1*
26 (similar to 20)
AGGGGGAGGG 1 890 1*
AAAAGTAGGG 1 911 1
GAAAGAGGGG 1 1034 1*
ATAAGAAGGG 5 1319 1*
ATGGGAGCGT 8 525 1
GAAAGGAGGG 14 378 1*
ATGGGGGTGG 17 586 1*
AAAGGCCCGG 22 500 1
ACAGACGGGG 22 894 1
AAAAAAGGGG 27 1382 1*
AAAGAAAGGG 28 1031 1*
AAGGTGGCGG 30 74 1*
GGGGAGAGGG 35 767 1*
ATAGGAGGGG 37 131 1*
AAAAAAGGGA 39 983 1
AAAGGCGCGG 42 9 1
AAAGTGGGGG 51 854 1*
ATAAAAGGGG 58 1522 1*
ATAAAGGGGG 61 913 1*
AAAGATGGGG 75 742 1*
AAAGAGGCGT 86 263 1
ATAAGTAGGG 93 704 1
AAAAAGGGGG 100 66 1*
ATAGGGGGGA 109 742 1*
ATGGATGGGG 115 1608 1*
ATAAAAGGGG 117 178 1*
ATAGGGGGGA 120 1203 1*
ATAGAAGAGG 124 1285 1
AAAAAGGGGG 125 922 1*
25 (weakly related to 23)
GGGACATC 4 832 1
GGGTCTTG 14 1412 1
GGGACACG 23 320 1
GGGGCATA 30 82 1*
GGGACATA 62 1117 1*
GGGACATA 66 141 1
GGGGCTTC 75 748 1*
GGGTCACG 80 189 1*
TGGACACG 86 1484 1
GGGTCACA 91 531 1
GGGGCATG 96 581 1*
TGGACACG 101 50 1
GGGACTTA 109 285 1
GGGGCAAA 121 798 1*
GGGACATA 123 509 1
29 related to 19
CCCAATAAAAAA 1 1079 1
CCCTTTAAAAGA 8 614 1
CCCAAAAAAAAA 16 822 1*
CCCCAAAAAAAA 19 1362 1*
CCCAATAAAATA 28 612 1*
CCCCAAAAAAAA 35 1559 1*
CCCAAAAAAAAA 48 1826 1*
CCCCATAAAATA 49 1234 1*
CCCAAAAAAAAA 49 1551 1*
CCCTTTAAAAAA 62 131 1
CCCCAAAAAAAA 65 1214 1*
CCCAAAAAAAAA 66 1081 1*
CCCAAAAAAATA 72 1118 1
CCCAAAAAAAAA 73 1601 1
CCCATAAAAATA 78 1695 1*
CCCACCAAAAAA 118 59 1
CCCTATAAAAAA 118 889 1
23 (4,23 related)
TGGGCTC 18 1390 1
AGGGCAC 42 469 1
AGGGCAC 48 1007 1*
AGGGCTC 54 616 1
GGGGCCT 54 865 1*
TGGGCCC 56 907 1
TGGGCGT 56 1957 1
GGGGCTT 58 1528 1*
TGGGCAT 61 541 1
TGGGCAT 62 924 1
AGGGCCC 65 1206 1
TGGGCTC 68 466 1
AGGGCTT 72 831 1*
GGGGCTT 75 748 1*
GGGGCAT 96 581 1*
GGGGCTC 125 928 1*
TGGGCTT 126 79 1
22 (weakly related to 4)
CACGGGGT 17 438 1*
CATGGGGG 17 585 1*
CACGTGGT 21 697 1
CACGTGGT 22 462 1
CATGGGGG 42 793 1
CAAGGGGA 58 1515 1
CATGTGGG 71 656 1
CATGTGGA 75 290 1*
CATGTGGT 88 443 1
CAAGGGGT 103 1390 1*
CATGGGGA 109 6 1*
Motif occurrences for motif 1
(G-rich motif), motif 2 (C-rich
motif) and motif 3 (TGTG motif)
20

## Slide 21
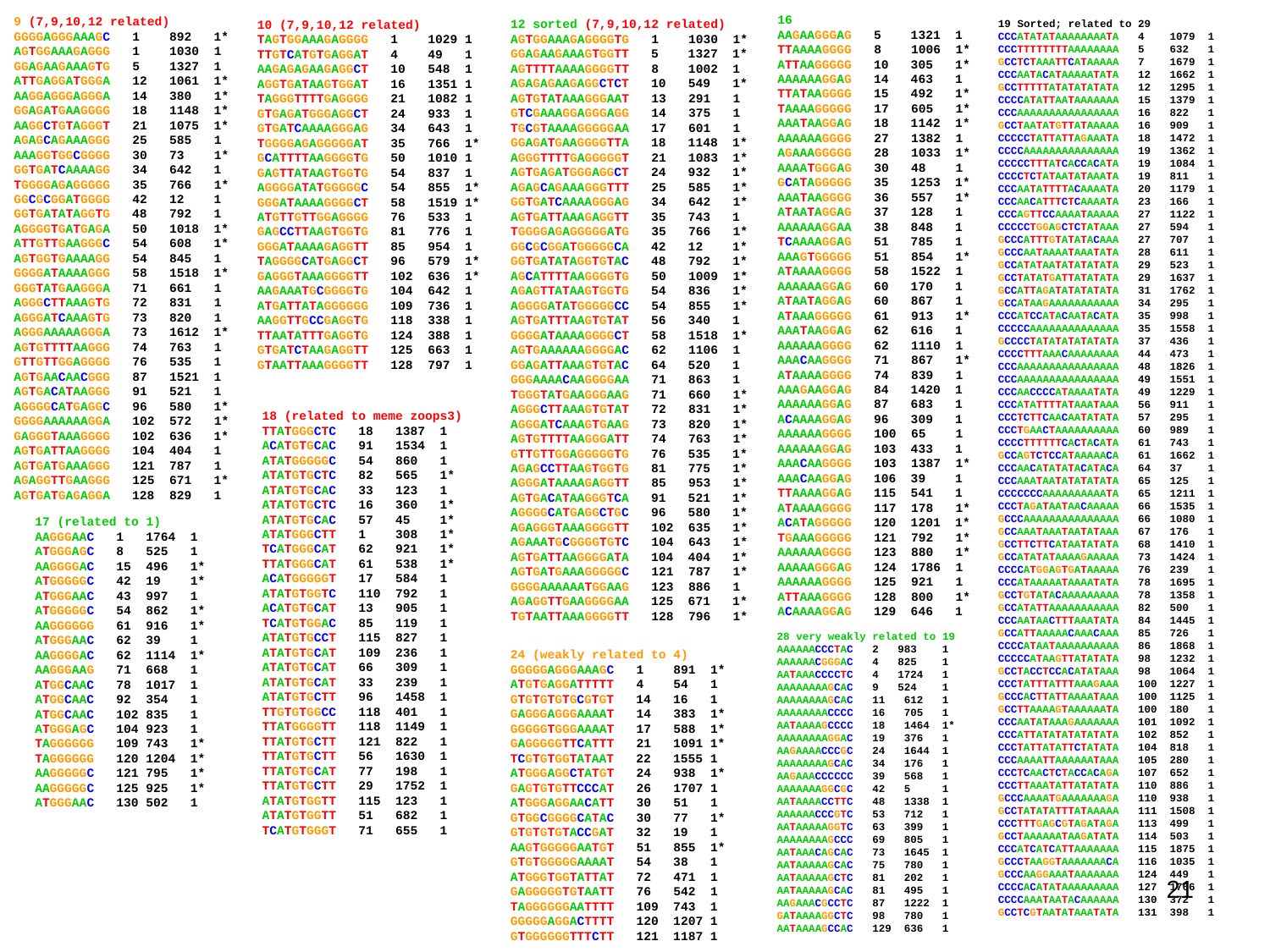

16
AAGAAGGGAG 5 1321 1
TTAAAAGGGG 8 1006 1*
ATTAAGGGGG 10 305 1*
AAAAAAGGAG 14 463 1
TTATAAGGGG 15 492 1*
TAAAAGGGGG 17 605 1*
AAATAAGGAG 18 1142 1*
AAAAAAGGGG 27 1382 1
AGAAAGGGGG 28 1033 1*
AAAATGGGAG 30 48 1
GCATAGGGGG 35 1253 1*
AAATAAGGGG 36 557 1*
ATAATAGGAG 37 128 1
AAAAAAGGAA 38 848 1
TCAAAAGGAG 51 785 1
AAAGTGGGGG 51 854 1*
ATAAAAGGGG 58 1522 1
AAAAAAGGAG 60 170 1
ATAATAGGAG 60 867 1
ATAAAGGGGG 61 913 1*
AAATAAGGAG 62 616 1
AAAAAAGGGG 62 1110 1
AAACAAGGGG 71 867 1*
ATAAAAGGGG 74 839 1
AAAGAAGGAG 84 1420 1
AAAAAAGGAG 87 683 1
ACAAAAGGAG 96 309 1
AAAAAAGGGG 100 65 1
AAAAAAGGAG 103 433 1
AAACAAGGGG 103 1387 1*
AAACAAGGAG 106 39 1
TTAAAAGGAG 115 541 1
ATAAAAGGGG 117 178 1*
ACATAGGGGG 120 1201 1*
TGAAAGGGGG 121 792 1*
AAAAAAGGGG 123 880 1*
AAAAAGGGAG 124 1786 1
AAAAAAGGGG 125 921 1
ATTAAAGGGG 128 800 1*
ACAAAAGGAG 129 646 1
9 (7,9,10,12 related)
GGGGAGGGAAAGC 1 892 1*
AGTGGAAAGAGGG 1 1030 1
GGAGAAGAAAGTG 5 1327 1
ATTGAGGATGGGA 12 1061 1*
AAGGAGGGAGGGA 14 380 1*
GGAGATGAAGGGG 18 1148 1*
AAGGCTGTAGGGT 21 1075 1*
AGAGCAGAAAGGG 25 585 1
AAAGGTGGCGGGG 30 73 1*
GGTGATCAAAAGG 34 642 1
TGGGGAGAGGGGG 35 766 1*
GGCGCGGATGGGG 42 12 1
GGTGATATAGGTG 48 792 1
AGGGGTGATGAGA 50 1018 1*
ATTGTTGAAGGGC 54 608 1*
AGTGGTGAAAAGG 54 845 1
GGGGATAAAAGGG 58 1518 1*
GGGTATGAAGGGA 71 661 1
AGGGCTTAAAGTG 72 831 1
AGGGATCAAAGTG 73 820 1
AGGGAAAAAGGGA 73 1612 1*
AGTGTTTTAAGGG 74 763 1
GTTGTTGGAGGGG 76 535 1
AGTGAACAACGGG 87 1521 1
AGTGACATAAGGG 91 521 1
AGGGGCATGAGGC 96 580 1*
GGGGAAAAAAGGA 102 572 1*
GAGGGTAAAGGGG 102 636 1*
AGTGATTAAGGGG 104 404 1
AGTGATGAAAGGG 121 787 1
AGAGGTTGAAGGG 125 671 1*
AGTGATGAGAGGA 128 829 1
12 sorted (7,9,10,12 related)
AGTGGAAAGAGGGGTG 1 1030 1*
GGAGAAGAAAGTGGTT 5 1327 1*
AGTTTTAAAAGGGGTT 8 1002 1
AGAGAGAAGAGGCTCT 10 549 1*
AGTGTATAAAGGGAAT 13 291 1
GTCGAAAGGAGGGAGG 14 375 1
TGCGTAAAAGGGGGAA 17 601 1
GGAGATGAAGGGGTTA 18 1148 1*
AGGGTTTTGAGGGGGT 21 1083 1*
AGTGAGATGGGAGGCT 24 932 1*
AGAGCAGAAAGGGTTT 25 585 1*
GGTGATCAAAAGGGAG 34 642 1*
AGTGATTAAAGAGGTT 35 743 1
TGGGGAGAGGGGGATG 35 766 1*
GGCGCGGATGGGGGCA 42 12 1*
GGTGATATAGGTGTAC 48 792 1*
AGCATTTTAAGGGGTG 50 1009 1*
AGAGTTATAAGTGGTG 54 836 1*
AGGGGATATGGGGGCC 54 855 1*
AGTGATTTAAGTGTAT 56 340 1
GGGGATAAAAGGGGCT 58 1518 1*
AGTGAAAAAAGGGGAC 62 1106 1
GGAGATTAAAGTGTAC 64 520 1
GGGAAAACAAGGGGAA 71 863 1
TGGGTATGAAGGGAAG 71 660 1*
AGGGCTTAAAGTGTAT 72 831 1*
AGGGATCAAAGTGAAG 73 820 1*
AGTGTTTTAAGGGATT 74 763 1*
GTTGTTGGAGGGGGTG 76 535 1*
AGAGCCTTAAGTGGTG 81 775 1*
AGGGATAAAAGAGGTT 85 953 1*
AGTGACATAAGGGTCA 91 521 1*
AGGGGCATGAGGCTGC 96 580 1*
AGAGGGTAAAGGGGTT 102 635 1*
AGAAATGCGGGGTGTC 104 643 1*
AGTGATTAAGGGGATA 104 404 1*
AGTGATGAAAGGGGGC 121 787 1*
GGGGAAAAAATGGAAG 123 886 1
AGAGGTTGAAGGGGAA 125 671 1*
TGTAATTAAAGGGGTT 128 796 1*
10 (7,9,10,12 related)
TAGTGGAAAGAGGGG 1 1029 1
TTGTCATGTGAGGAT 4 49 1
AAGAGAGAAGAGGCT 10 548 1
AGGTGATAAGTGGAT 16 1351 1
TAGGGTTTTGAGGGG 21 1082 1
GTGAGATGGGAGGCT 24 933 1
GTGATCAAAAGGGAG 34 643 1
TGGGGAGAGGGGGAT 35 766 1*
GCATTTTAAGGGGTG 50 1010 1
GAGTTATAAGTGGTG 54 837 1
AGGGGATATGGGGGC 54 855 1*
GGGATAAAAGGGGCT 58 1519 1*
ATGTTGTTGGAGGGG 76 533 1
GAGCCTTAAGTGGTG 81 776 1
GGGATAAAAGAGGTT 85 954 1
TAGGGGCATGAGGCT 96 579 1*
GAGGGTAAAGGGGTT 102 636 1*
AAGAAATGCGGGGTG 104 642 1
ATGATTATAGGGGGG 109 736 1
AAGGTTGCCGAGGTG 118 338 1
TTAATATTTGAGGTG 124 388 1
GTGATCTAAGAGGTT 125 663 1
GTAATTAAAGGGGTT 128 797 1
19 Sorted; related to 29
CCCATATATAAAAAAAATA 4 1079 1
CCCTTTTTTTTAAAAAAAA 5 632 1
GCCTCTAAATTCATAAAAA 7 1679 1
CCCAATACATAAAAATATA 12 1662 1
GCCTTTTTATATATATATA 12 1295 1
CCCCATATTAATAAAAAAA 15 1379 1
CCCAAAAAAAAAAAAAAAA 16 822 1
GCCTAATATGTTATAAAAA 16 909 1
CCCCCTATTATTAGAAATA 18 1472 1
CCCCAAAAAAAAAAAAAAA 19 1362 1
CCCCCTTTATCACCACATA 19 1084 1
CCCCTCTATAATATAAATA 19 811 1
CCCAATATTTTACAAAATA 20 1179 1
CCCAACATTTCTCAAAATA 23 166 1
CCCAGTTCCAAAATAAAAA 27 1122 1
CCCCCTGGAGCTCTATAAA 27 594 1
GCCCATTTGTATATACAAA 27 707 1
GCCCAATAAAATAAATATA 28 611 1
GCCATATAATATATATATA 29 523 1
GCCTATATGATTATATATA 29 1637 1
GCCATTAGATATATATATA 31 1762 1
GCCATAAGAAAAAAAAAAA 34 295 1
CCCATCCATACAATACATA 35 998 1
CCCCCAAAAAAAAAAAAAA 35 1558 1
GCCCCTATATATATATATA 37 436 1
CCCCTTTAAACAAAAAAAA 44 473 1
CCCAAAAAAAAAAAAAAAA 48 1826 1
CCCAAAAAAAAAAAAAAAA 49 1551 1
CCCAACCCCATAAAATATA 49 1229 1
CCCATATTTTATAAATAAA 56 911 1
CCCTCTTCAACAATATATA 57 295 1
CCCTGAACTAAAAAAAAAA 60 989 1
CCCCTTTTTTCACTACATA 61 743 1
GCCAGTCTCCATAAAAACA 61 1662 1
CCCAACATATATACATACA 64 37 1
CCCAAATAATATATATATA 65 125 1
CCCCCCCAAAAAAAAAATA 65 1211 1
CCCTAGATAATAACAAAAA 66 1535 1
GCCCAAAAAAAAAAAAAAA 66 1080 1
GCCAAATAAATAATATAAA 67 176 1
GCCTTCTTCATAATATATA 68 1410 1
GCCATATATAAAAGAAAAA 73 1424 1
CCCCATGGAGTGATAAAAA 76 239 1
CCCATAAAAATAAAATATA 78 1695 1
GCCTGTATACAAAAAAAAA 78 1358 1
GCCATATTAAAAAAAAAAA 82 500 1
CCCAATAACTTTAAATATA 84 1445 1
GCCATTAAAAACAAACAAA 85 726 1
CCCCATAATAAAAAAAAAA 86 1868 1
CCCCCATAAGTTATATATA 98 1232 1
GCCTACCTCCACATATAAA 98 1064 1
CCCTATTTATTTAAAGAAA 100 1227 1
GCCCACTTATTAAAATAAA 100 1125 1
GCCTTAAAAGTAAAAAATA 100 180 1
CCCAATATAAAGAAAAAAA 101 1092 1
CCCATTATATATATATATA 102 852 1
CCCTATTATATTCTATATA 104 818 1
CCCAAAATTAAAAAATAAA 105 280 1
CCCTCAACTCTACCACAGA 107 652 1
CCCTTAAATATTATATATA 110 886 1
GCCCAAAATGAAAAAAAGA 110 938 1
GCCTATATATTTATAAAAA 111 1508 1
CCCTTTGAGCGTAGATAGA 113 499 1
GCCTAAAAAATAAGATATA 114 503 1
CCCATCATCATTAAAAAAA 115 1875 1
GCCCTAAGGTAAAAAAACA 116 1035 1
GCCCAAGGAAATAAAAAAA 124 449 1
CCCCACATATAAAAAAAAA 127 1766 1
CCCCAAATAATACAAAAAA 130 372 1
GCCTCGTAATATAAATATA 131 398 1
18 (related to meme zoops3)
TTATGGGCTC 18 1387 1
ACATGTGCAC 91 1534 1
ATATGGGGGC 54 860 1
ATATGTGCTC 82 565 1*
ATATGTGCAC 33 123 1
ATATGTGCTC 16 360 1*
ATATGTGCAC 57 45 1*
ATATGGGCTT 1 308 1*
TCATGGGCAT 62 921 1*
TTATGGGCAT 61 538 1*
ACATGGGGGT 17 584 1
ATATGTGGTC 110 792 1
ACATGTGCAT 13 905 1
TCATGTGGAC 85 119 1
ATATGTGCCT 115 827 1
ATATGTGCAT 109 236 1
ATATGTGCAT 66 309 1
ATATGTGCAT 33 239 1
ATATGTGCTT 96 1458 1
TTGTGTGGCC 118 401 1
TTATGGGGTT 118 1149 1
TTATGTGCTT 121 822 1
TTATGTGCTT 56 1630 1
TTATGTGCAT 77 198 1
TTATGTGCTT 29 1752 1
ATATGTGGTT 115 123 1
ATATGTGGTT 51 682 1
TCATGTGGGT 71 655 1
17 (related to 1)
AAGGGAAC 1 1764 1
ATGGGAGC 8 525 1
AAGGGGAC 15 496 1*
ATGGGGGC 42 19 1*
ATGGGAAC 43 997 1
ATGGGGGC 54 862 1*
AAGGGGGG 61 916 1*
ATGGGAAC 62 39 1
AAGGGGAC 62 1114 1*
AAGGGAAG 71 668 1
ATGGCAAC 78 1017 1
ATGGCAAC 92 354 1
ATGGCAAC 102 835 1
ATGGGAGC 104 923 1
TAGGGGGG 109 743 1*
TAGGGGGG 120 1204 1*
AAGGGGGC 121 795 1*
AAGGGGGC 125 925 1*
ATGGGAAC 130 502 1
28 very weakly related to 19
AAAAAACCCTAC 2 983 1
AAAAAACGGGAC 4 825 1
AATAAACCCCTC 4 1724 1
AAAAAAAAGCAC 9 524 1
AAAAAAAAGCAC 11 612 1
AAAAAAAACCCC 16 705 1
AATAAAAGCCCC 18 1464 1*
AAAAAAAAGGAC 19 376 1
AAGAAAACCCGC 24 1644 1
AAAAAAAAGCAC 34 176 1
AAGAAACCCCCC 39 568 1
AAAAAAAGGCGC 42 5 1
AATAAAACCTTC 48 1338 1
AAAAAACCCGTC 53 712 1
AATAAAAAGGTC 63 399 1
AAAAAAAAGCCC 69 805 1
AATAAACAGCAC 73 1645 1
AATAAAAAGCAC 75 780 1
AATAAAAAGCTC 81 202 1
AATAAAAAGCAC 81 495 1
AAGAAACGCCTC 87 1222 1
GATAAAAGGCTC 98 780 1
AATAAAAGCCAC 129 636 1
24 (weakly related to 4)
GGGGGAGGGAAAGC 1 891 1*
ATGTGAGGATTTTT 4 54 1
GTGTGTGTGCGTGT 14 16 1
GAGGGAGGGAAAAT 14 383 1*
GGGGGTGGGAAAAT 17 588 1*
GAGGGGGTTCATTT 21 1091 1*
TCGTGTGGTATAAT 22 1555 1
ATGGGAGGCTATGT 24 938 1*
GAGTGTGTTCCCAT 26 1707 1
ATGGGAGGAACATT 30 51 1
GTGGCGGGGCATAC 30 77 1*
GTGTGTGTACCGAT 32 19 1
AAGTGGGGGAATGT 51 855 1*
GTGTGGGGGAAAAT 54 38 1
ATGGGTGGTATTAT 72 471 1
GAGGGGGTGTAATT 76 542 1
TAGGGGGGAATTTT 109 743 1
GGGGGAGGACTTTT 120 1207 1
GTGGGGGGTTTCTT 121 1187 1
21

## Slide 22
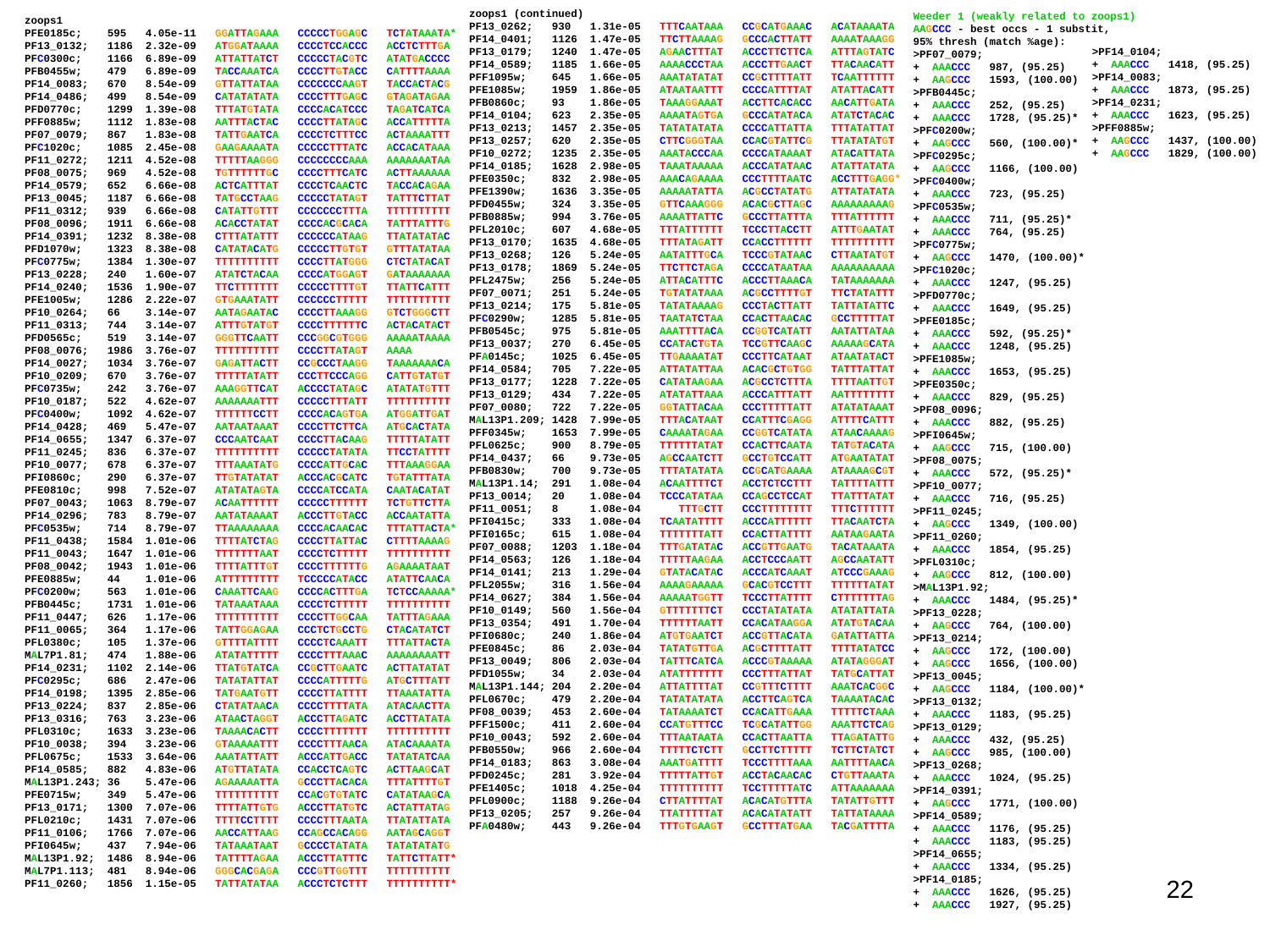

zoops1 (continued)
PF13_0262; 930 1.31e-05 TTTCAATAAA CCGCATGAAAC ACATAAAATA
PF14_0401; 1126 1.47e-05 TTCTTAAAAG GCCCACTTATT AAAATAAAGG
PF13_0179; 1240 1.47e-05 AGAACTTTAT ACCCTTCTTCA ATTTAGTATC
PF14_0589; 1185 1.66e-05 AAAACCCTAA ACCCTTGAACT TTACAACATT
PFF1095w; 645 1.66e-05 AAATATATAT CCGCTTTTATT TCAATTTTTT
PFE1085w; 1959 1.86e-05 ATAATAATTT CCCCATTTTAT ATATTACATT
PFB0860c; 93 1.86e-05 TAAAGGAAAT ACCTTCACACC AACATTGATA
PF14_0104; 623 2.35e-05 AAAATAGTGA GCCCATATACA ATATCTACAC
PF13_0213; 1457 2.35e-05 TATATATATA CCCCATTATTA TTTATATTAT
PF13_0257; 620 2.35e-05 CTTCGGGTAA CCACGTATTCG TTATATATGT
PF10_0272; 1235 2.35e-05 AAATACCCAA CCCCATAAAAT ATACATTATA
PF14_0185; 1628 2.98e-05 TAAATAAAAA ACCCATATAAC ATATTATATA
PFE0350c; 832 2.98e-05 AAACAGAAAA CCCTTTTAATC ACCTTTGAGG*
PFE1390w; 1636 3.35e-05 AAAAATATTA ACGCCTATATG ATTATATATA
PFD0455w; 324 3.35e-05 GTTCAAAGGG ACACGCTTAGC AAAAAAAAAG
PFB0885w; 994 3.76e-05 AAAATTATTC GCCCTTATTTA TTTATTTTTT
PFL2010c; 607 4.68e-05 TTTATTTTTT TCCCTTACCTT ATTTGAATAT
PF13_0170; 1635 4.68e-05 TTTATAGATT CCACCTTTTTT TTTTTTTTTT
PF13_0268; 126 5.24e-05 AATATTTGCA TCCCGTATAAC CTTAATATGT
PF13_0178; 1869 5.24e-05 TTCTTCTAGA CCCCATAATAA AAAAAAAAAA
PFL2475w; 256 5.24e-05 ATTACATTTC ACCCTTAAACA TATAAAAAAA
PF07_0071; 251 5.24e-05 TGTATATAAA ACGCCTTTTGT TTCTATATTT
PF13_0214; 175 5.81e-05 TATATAAAAG CCCTACTTATT TATTATATTC
PFC0290w; 1285 5.81e-05 TAATATCTAA CCACTTAACAC GCCTTTTTAT
PFB0545c; 975 5.81e-05 AAATTTTACA CCGGTCATATT AATATTATAA
PF13_0037; 270 6.45e-05 CCATACTGTA TCCGTTCAAGC AAAAAGCATA
PFA0145c; 1025 6.45e-05 TTGAAAATAT CCCTTCATAAT ATAATATACT
PF14_0584; 705 7.22e-05 ATTATATTAA ACACGCTGTGG TATTTATTAT
PF13_0177; 1228 7.22e-05 CATATAAGAA ACGCCTCTTTA TTTTAATTGT
PF13_0129; 434 7.22e-05 ATATATTAAA ACCCATTTATT AATTTTTTTT
PF07_0080; 722 7.22e-05 GGTATTACAA CCCTTTTTATT ATATATAAAT
MAL13P1.209; 1428 7.99e-05 TTTACATAAT CCATTTCGAGG ATTTTCATTT
PFF0345w; 1653 7.99e-05 CAAAATAGAA CCGGTCATATA ATAACAAAAG
PFL0625c; 900 8.79e-05 TTTTTTATAT CCACTTCAATA TATGTACATA
PF14_0437; 66 9.73e-05 AGCCAATCTT GCCTGTCCATT ATGAATATAT
PFB0830w; 700 9.73e-05 TTTATATATA CCGCATGAAAA ATAAAAGCGT
MAL13P1.14; 291 1.08e-04 ACAATTTTCT ACCTCTCCTTT TATTTTATTT
PF13_0014; 20 1.08e-04 TCCCATATAA CCAGCCTCCAT TTATTTATAT
PF11_0051; 8 1.08e-04 TTTGCTT CCCTTTTTTTT TTTCTTTTTT
PFI0415c; 333 1.08e-04 TCAATATTTT ACCCATTTTTT TTACAATCTA
PFI0165c; 615 1.08e-04 TTTTTTTATT CCACTTATTTT AATAAGAATA
PF07_0088; 1203 1.18e-04 TTTGATATAC ACCGTTGAATG TACATAAATA
PF14_0563; 126 1.18e-04 TTTTTAAGAA ACCTCCCAATT AGCCAATATT
PF14_0141; 213 1.29e-04 GTATACATAC ACCCATCAAAT ATCCCGAAAG
PFL2055w; 316 1.56e-04 AAAAGAAAAA GCACGTCCTTT TTTTTTATAT
PF14_0627; 384 1.56e-04 AAAAATGGTT TCCCTTATTTT CTTTTTTTAG
PF10_0149; 560 1.56e-04 GTTTTTTTCT CCCTATATATA ATATATTATA
PF13_0354; 491 1.70e-04 TTTTTTAATT CCACATAAGGA ATATGTACAA
PFI0680c; 240 1.86e-04 ATGTGAATCT ACCGTTACATA GATATTATTA
PFE0845c; 86 2.03e-04 TATATGTTGA ACGCTTTTATT TTTTATATCC
PF13_0049; 806 2.03e-04 TATTTCATCA ACCCGTAAAAA ATATAGGGAT
PFD1055w; 34 2.03e-04 ATATTTTTTT CCCTTTATTAT TATGCATTAT
MAL13P1.144; 204 2.20e-04 ATTATTTTAT CCGTTTCTTTT AAATCACGGC
PFL0670c; 479 2.20e-04 TATATATATA ACCTTCAGTCA TAAAATACAC
PF08_0039; 453 2.60e-04 TATAAAATCT CCACATTGAAA TTTTTCTAAA
PFF1500c; 411 2.60e-04 CCATGTTTCC TCGCATATTGG AAATTCTCAG
PF10_0043; 592 2.60e-04 TTTAATAATA CCACTTAATTA TTAGATATTG
PFB0550w; 966 2.60e-04 TTTTTCTCTT GCCTTCTTTTT TCTTCTATCT
PF14_0183; 863 3.08e-04 AAATGATTTT TCCCTTTTAAA AATTTTAACA
PFD0245c; 281 3.92e-04 TTTTTATTGT ACCTACAACAC CTGTTAAATA
PFE1405c; 1018 4.25e-04 TTTTTTTTTT TCCTTTTTATC ATTAAAAAAA
PFL0900c; 1188 9.26e-04 CTTATTTTAT ACACATGTTTA TATATTGTTT
PF13_0205; 257 9.26e-04 TTATTTTTAT ACACATATATT TATTATAAAA
PFA0480w; 443 9.26e-04 TTTGTGAAGT GCCTTTATGAA TACGATTTTA
Weeder 1 (weakly related to zoops1)
AAGCCC - best occs - 1 substit,
95% thresh (match %age):
>PF07_0079;
+ AAACCC 987, (95.25)
+ AAGCCC 1593, (100.00)
>PFB0445c;
+ AAACCC 252, (95.25)
+ AAACCC 1728, (95.25)*
>PFC0200w;
+ AAGCCC 560, (100.00)*
>PFC0295c;
+ AAGCCC 1166, (100.00)
>PFC0400w;
+ AAACCC 723, (95.25)
>PFC0535w;
+ AAACCC 711, (95.25)*
+ AAACCC 764, (95.25)
>PFC0775w;
+ AAGCCC 1470, (100.00)*
>PFC1020c;
+ AAACCC 1247, (95.25)
>PFD0770c;
+ AAACCC 1649, (95.25)
>PFE0185c;
+ AAACCC 592, (95.25)*
+ AAACCC 1248, (95.25)
>PFE1085w;
+ AAACCC 1653, (95.25)
>PFE0350c;
+ AAACCC 829, (95.25)
>PF08_0096;
+ AAACCC 882, (95.25)
>PFI0645w;
+ AAGCCC 715, (100.00)
>PF08_0075;
+ AAACCC 572, (95.25)*
>PF10_0077;
+ AAACCC 716, (95.25)
>PF11_0245;
+ AAGCCC 1349, (100.00)
>PF11_0260;
+ AAACCC 1854, (95.25)
>PFL0310c;
+ AAGCCC 812, (100.00)
>MAL13P1.92;
+ AAACCC 1484, (95.25)*
>PF13_0228;
+ AAGCCC 764, (100.00)
>PF13_0214;
+ AAGCCC 172, (100.00)
+ AAGCCC 1656, (100.00)
>PF13_0045;
+ AAGCCC 1184, (100.00)*
>PF13_0132;
+ AAACCC 1183, (95.25)
>PF13_0129;
+ AAACCC 432, (95.25)
+ AAGCCC 985, (100.00)
>PF13_0268;
+ AAACCC 1024, (95.25)
>PF14_0391;
+ AAGCCC 1771, (100.00)
>PF14_0589;
+ AAACCC 1176, (95.25)
+ AAACCC 1183, (95.25)
>PF14_0655;
+ AAACCC 1334, (95.25)
>PF14_0185;
+ AAACCC 1626, (95.25)
+ AAACCC 1927, (95.25)
>PF14_0104;
+ AAACCC 1418, (95.25)
>PF14_0083;
+ AAACCC 1873, (95.25)
>PF14_0231;
+ AAACCC 1623, (95.25)
>PFF0885w;
+ AAGCCC 1437, (100.00)
+ AAGCCC 1829, (100.00)
zoops1
PFE0185c; 595 4.05e-11 GGATTAGAAA CCCCCTGGAGC TCTATAAATA*
PF13_0132; 1186 2.32e-09 ATGGATAAAA CCCCTCCACCC ACCTCTTTGA
PFC0300c; 1166 6.89e-09 ATTATTATCT CCCCCTACGTC ATATGACCCC
PFB0455w; 479 6.89e-09 TACCAAATCA CCCCTTGTACC CATTTTAAAA
PF14_0083; 670 8.54e-09 GTTATTATAA CCCCCCCAAGT TACCACTACG
PF14_0486; 499 8.54e-09 CATATATATA CCCCTTTGAGC GTAGATAGAA
PFD0770c; 1299 1.39e-08 TTTATGTATA CCCCACATCCC TAGATCATCA
PFF0885w; 1112 1.83e-08 AATTTACTAC CCCCTTATAGC ACCATTTTTA
PF07_0079; 867 1.83e-08 TATTGAATCA CCCCTCTTTCC ACTAAAATTT
PFC1020c; 1085 2.45e-08 GAAGAAAATA CCCCCTTTATC ACCACATAAA
PF11_0272; 1211 4.52e-08 TTTTTAAGGG CCCCCCCCAAA AAAAAAATAA
PF08_0075; 969 4.52e-08 TGTTTTTTGC CCCCTTTCATC ACTTAAAAAA
PF14_0579; 652 6.66e-08 ACTCATTTAT CCCCTCAACTC TACCACAGAA
PF13_0045; 1187 6.66e-08 TATGCCTAAG CCCCCTATAGT TATTTCTTAT
PF11_0312; 939 6.66e-08 CATATTGTTT CCCCCCCTTTA TTTTTTTTTT
PF08_0096; 1911 6.66e-08 ACACCTATAT CCCCACGCACA TATTTATTTG
PF14_0391; 1232 8.38e-08 CTTTATATTT CCCCCCATAAG TTATATATAC
PFD1070w; 1323 8.38e-08 CATATACATG CCCCCTTGTGT GTTTATATAA
PFC0775w; 1384 1.30e-07 TTTTTTTTTT CCCCTTATGGG CTCTATACAT
PF13_0228; 240 1.60e-07 ATATCTACAA CCCCATGGAGT GATAAAAAAA
PF14_0240; 1536 1.90e-07 TTCTTTTTTT CCCCCTTTTGT TTATTCATTT
PFE1005w; 1286 2.22e-07 GTGAAATATT CCCCCCTTTTT TTTTTTTTTT
PF10_0264; 66 3.14e-07 AATAGAATAC CCCCTTAAAGG GTCTGGGCTT
PF11_0313; 744 3.14e-07 ATTTGTATGT CCCCTTTTTTC ACTACATACT
PFD0565c; 519 3.14e-07 GGGTTCAATT CCCGGCGTGGG AAAAATAAAA
PF08_0076; 1986 3.76e-07 TTTTTTTTTT CCCCTTATAGT AAAA
PF14_0027; 1034 3.76e-07 GAGATTACTT CCGCCCTAAGG TAAAAAAACA
PF10_0209; 670 3.76e-07 TTTTTATATT CCCTTCCCAGG CATTGTATGT
PFC0735w; 242 3.76e-07 AAAGGTTCAT ACCCCTATAGC ATATATGTTT
PF10_0187; 522 4.62e-07 AAAAAAATTT CCCCCTTTATT TTTTTTTTTT
PFC0400w; 1092 4.62e-07 TTTTTTCCTT CCCCACAGTGA ATGGATTGAT
PF14_0428; 469 5.47e-07 AATAATAAAT CCCCTTCTTCA ATGCACTATA
PF14_0655; 1347 6.37e-07 CCCAATCAAT CCCCTTACAAG TTTTTATATT
PF11_0245; 836 6.37e-07 TTTTTTTTTT CCCCCTATATA TTCCTATTTT
PF10_0077; 678 6.37e-07 TTTAAATATG CCCCATTGCAC TTTAAAGGAA
PFI0860c; 290 6.37e-07 TTGTATATAT ACCCACGCATC TGTATTTATA
PFE0810c; 998 7.52e-07 ATATATAGTA CCCCATCCATA CAATACATAT
PF07_0043; 1063 8.79e-07 ACAATTTTTT CCCCCTTTTTT TCTGTTCTTA
PF14_0296; 783 8.79e-07 AATATAAAAT ACCCTTGTACC ACCAATATTA
PFC0535w; 714 8.79e-07 TTAAAAAAAA CCCCACAACAC TTTATTACTA*
PF11_0438; 1584 1.01e-06 TTTTATCTAG CCCCTTATTAC CTTTTAAAAG
PF11_0043; 1647 1.01e-06 TTTTTTTAAT CCCCTCTTTTT TTTTTTTTTT
PF08_0042; 1943 1.01e-06 TTTTATTTGT CCCCTTTTTTG AGAAAATAAT
PFE0885w; 44 1.01e-06 ATTTTTTTTT TCCCCCATACC ATATTCAACA
PFC0200w; 563 1.01e-06 CAAATTCAAG CCCCACTTTGA TCTCCAAAAA*
PFB0445c; 1731 1.01e-06 TATAAATAAA CCCCTCTTTTT TTTTTTTTTT
PF11_0447; 626 1.17e-06 TTTTTTTTTT CCCCTTGGCAA TATTTAGAAA
PF11_0065; 364 1.17e-06 TATTGGAGAA CCCTCTGCCTG CTACATATCT
PFL0380c; 105 1.37e-06 GTTTTATTTT CCCCTCAAATT TTTATTACTA
MAL7P1.81; 474 1.88e-06 ATATATTTTT CCCCTTTAAAC AAAAAAAATT
PF14_0231; 1102 2.14e-06 TTATGTATCA CCGCTTGAATC ACTTATATAT
PFC0295c; 686 2.47e-06 TATATATTAT CCCCATTTTTG ATGCTTTATT
PF14_0198; 1395 2.85e-06 TATGAATGTT CCCCTTATTTT TTAAATATTA
PF13_0224; 837 2.85e-06 CTATATAACA CCCCTTTTATA ATACAACTTA
PF13_0316; 763 3.23e-06 ATAACTAGGT ACCCTTAGATC ACCTTATATA
PFL0310c; 1633 3.23e-06 TAAAACACTT CCCCTTTTTTT TTTTTTTTTT
PF10_0038; 394 3.23e-06 GTAAAAATTT CCCCTTTAACA ATACAAAATA
PFL0675c; 1533 3.64e-06 AAATATTATT ACCCATTGACC TATATATCAA
PF14_0585; 882 4.83e-06 ATGTTATATA CCACCTCAGTC ACTTAAGCAT
MAL13P1.243; 36 5.47e-06 AGAAAAATTA GCCCTTACACA TTTATTTTGT
PFE0715w; 349 5.47e-06 TTTTTTTTTT CCACGTGTATC CATATAAGCA
PF13_0171; 1300 7.07e-06 TTTTATTGTG ACCCTTATGTC ACTATTATAG
PFL0210c; 1431 7.07e-06 TTTTCCTTTT CCCCTTTAATA TTATATTATA
PF11_0106; 1766 7.07e-06 AACCATTAAG CCAGCCACAGG AATAGCAGGT
PFI0645w; 437 7.94e-06 TATAAATAAT GCCCCTATATA TATATATATG
MAL13P1.92; 1486 8.94e-06 TATTTTAGAA ACCCTTATTTC TATTCTTATT*
MAL7P1.113; 481 8.94e-06 GGGCACGAGA CCCGTTGGTTT TTTTTTTTTT
PF11_0260; 1856 1.15e-05 TATTATATAA ACCCTCTCTTT TTTTTTTTTT*
22

## Slide 23
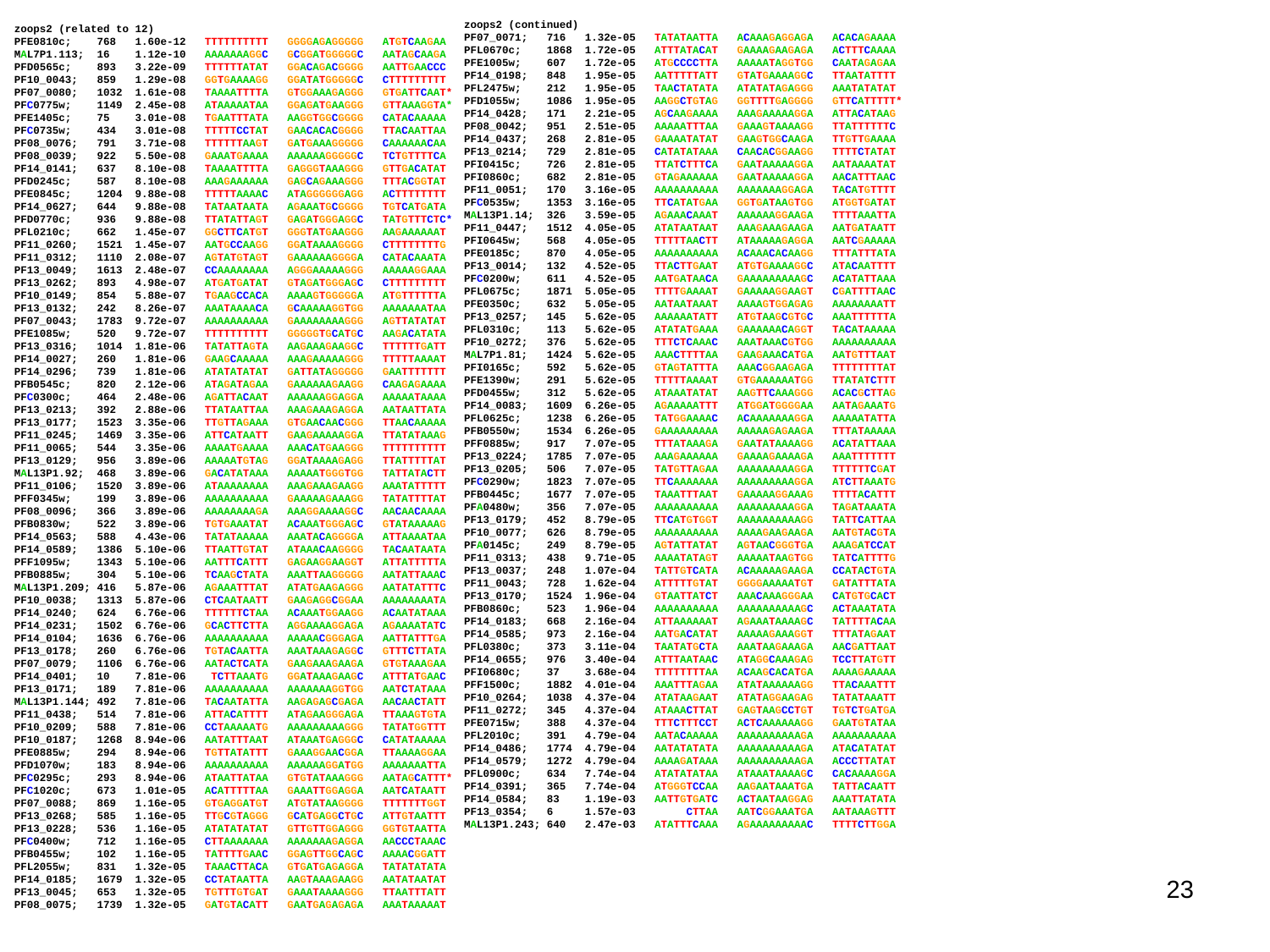

zoops2 (continued)
PF07_0071; 716 1.32e-05 TATATAATTA ACAAAGAGGAGA ACACAGAAAA
PFL0670c; 1868 1.72e-05 ATTTATACAT GAAAAGAAGAGA ACTTTCAAAA
PFE1005w; 607 1.72e-05 ATGCCCCTTA AAAAATAGGTGG CAATAGAGAA
PF14_0198; 848 1.95e-05 AATTTTTATT GTATGAAAAGGC TTAATATTTT
PFL2475w; 212 1.95e-05 TAACTATATA ATATATAGAGGG AAATATATAT
PFD1055w; 1086 1.95e-05 AAGGCTGTAG GGTTTTGAGGGG GTTCATTTTT*
PF14_0428; 171 2.21e-05 AGCAAGAAAA AAAGAAAAAGGA ATTACATAAG
PF08_0042; 951 2.51e-05 AAAAATTTAA GAAAGTAAAAGG TTATTTTTTC
PF14_0437; 268 2.81e-05 GAAAATATAT GAAGTGGCAAGA TTGTTGAAAA
PF13_0214; 729 2.81e-05 CATATATAAA CAACACGGAAGG TTTTCTATAT
PFI0415c; 726 2.81e-05 TTATCTTTCA GAATAAAAAGGA AATAAAATAT
PFI0860c; 682 2.81e-05 GTAGAAAAAA GAATAAAAAGGA AACATTTAAC
PF11_0051; 170 3.16e-05 AAAAAAAAAA AAAAAAAGGAGA TACATGTTTT
PFC0535w; 1353 3.16e-05 TTCATATGAA GGTGATAAGTGG ATGGTGATAT
MAL13P1.14; 326 3.59e-05 AGAAACAAAT AAAAAAGGAAGA TTTTAAATTA
PF11_0447; 1512 4.05e-05 ATATAATAAT AAAGAAAGAAGA AATGATAATT
PFI0645w; 568 4.05e-05 TTTTTAACTT ATAAAAAGAGGA AATCGAAAAA
PFE0185c; 870 4.05e-05 AAAAAAAAAA ACAAACACAAGG TTTATTTATA
PF13_0014; 132 4.52e-05 TTACTTGAAT ATGTGAAAAGGC ATACAATTTT
PFC0200w; 611 4.52e-05 AATGATAACA GAAAAAAAAAGC ACATATTAAA
PFL0675c; 1871 5.05e-05 TTTTGAAAAT GAAAAAGGAAGT CGATTTTAAC
PFE0350c; 632 5.05e-05 AATAATAAAT AAAAGTGGAGAG AAAAAAAATT
PF13_0257; 145 5.62e-05 AAAAAATATT ATGTAAGCGTGC AAATTTTTTA
PFL0310c; 113 5.62e-05 ATATATGAAA GAAAAAACAGGT TACATAAAAA
PF10_0272; 376 5.62e-05 TTTCTCAAAC AAATAAACGTGG AAAAAAAAAA
MAL7P1.81; 1424 5.62e-05 AAACTTTTAA GAAGAAACATGA AATGTTTAAT
PFI0165c; 592 5.62e-05 GTAGTATTTA AAACGGAAGAGA TTTTTTTTAT
PFE1390w; 291 5.62e-05 TTTTTAAAAT GTGAAAAAATGG TTATATCTTT
PFD0455w; 312 5.62e-05 ATAAATATAT AAGTTCAAAGGG ACACGCTTAG
PF14_0083; 1609 6.26e-05 AGAAAAATTT ATGGATGGGGAA AATAGAAATG
PFL0625c; 1238 6.26e-05 TATGGAAAAC ACAAAAAAAGGA AAAAATATTA
PFB0550w; 1534 6.26e-05 GAAAAAAAAA AAAAAGAGAAGA TTTATAAAAA
PFF0885w; 917 7.07e-05 TTTATAAAGA GAATATAAAAGG ACATATTAAA
PF13_0224; 1785 7.07e-05 AAAGAAAAAA GAAAAGAAAAGA AAATTTTTTT
PF13_0205; 506 7.07e-05 TATGTTAGAA AAAAAAAAAGGA TTTTTTCGAT
PFC0290w; 1823 7.07e-05 TTCAAAAAAA AAAAAAAAAGGA ATCTTAAATG
PFB0445c; 1677 7.07e-05 TAAATTTAAT GAAAAAGGAAAG TTTTACATTT
PFA0480w; 356 7.07e-05 AAAAAAAAAA AAAAAAAAAGGA TAGATAAATA
PF13_0179; 452 8.79e-05 TTCATGTGGT AAAAAAAAAAGG TATTCATTAA
PF10_0077; 626 8.79e-05 AAAAAAAAAA AAAAGAAGAAGA AATGTACGTA
PFA0145c; 249 8.79e-05 AGTATTATAT AGTAACGGGTGA AAAGATCCAT
PF11_0313; 438 9.71e-05 AAAATATAGT AAAAATAAGTGG TATCATTTTG
PF13_0037; 248 1.07e-04 TATTGTCATA ACAAAAAGAAGA CCATACTGTA
PF11_0043; 728 1.62e-04 ATTTTTGTAT GGGGAAAAATGT GATATTTATA
PF13_0170; 1524 1.96e-04 GTAATTATCT AAACAAAGGGAA CATGTGCACT
PFB0860c; 523 1.96e-04 AAAAAAAAAA AAAAAAAAAAGC ACTAAATATA
PF14_0183; 668 2.16e-04 ATTAAAAAAT AGAAATAAAAGC TATTTTACAA
PF14_0585; 973 2.16e-04 AATGACATAT AAAAAGAAAGGT TTTATAGAAT
PFL0380c; 373 3.11e-04 TAATATGCTA AAATAAGAAAGA AACGATTAAT
PF14_0655; 976 3.40e-04 ATTTAATAAC ATAGGCAAAGAG TCCTTATGTT
PFI0680c; 37 3.68e-04 TTTTTTTTAA ACAAGCACATGA AAAAGAAAAA
PFF1500c; 1882 4.01e-04 AAATTTAGAA ATATAAAAAAGG TTACAAATTT
PF10_0264; 1038 4.37e-04 ATATAAGAAT ATATAGGAAGAG TATATAAATT
PF11_0272; 345 4.37e-04 ATAAACTTAT GAGTAAGCCTGT TGTCTGATGA
PFE0715w; 388 4.37e-04 TTTCTTTCCT ACTCAAAAAAGG GAATGTATAA
PFL2010c; 391 4.79e-04 AATACAAAAA AAAAAAAAAAGA AAAAAAAAAA
PF14_0486; 1774 4.79e-04 AATATATATA AAAAAAAAAAGA ATACATATAT
PF14_0579; 1272 4.79e-04 AAAAGATAAA AAAAAAAAAAGA ACCCTTATAT
PFL0900c; 634 7.74e-04 ATATATATAA ATAAATAAAAGC CACAAAAGGA
PF14_0391; 365 7.74e-04 ATGGGTCCAA AAGAATAAATGA TATTACAATT
PF14_0584; 83 1.19e-03 AATTGTGATC ACTAATAAGGAG AAATTATATA
PF13_0354; 6 1.57e-03 CTTAA AATCGGAAATGA AATAAAGTTT
MAL13P1.243; 640 2.47e-03 ATATTTCAAA AGAAAAAAAAAC TTTTCTTGGA
zoops2 (related to 12)
PFE0810c; 768 1.60e-12 TTTTTTTTTT GGGGAGAGGGGG ATGTCAAGAA
MAL7P1.113; 16 1.12e-10 AAAAAAAGGC GCGGATGGGGGC AATAGCAAGA
PFD0565c; 893 3.22e-09 TTTTTTATAT GGACAGACGGGG AATTGAACCC
PF10_0043; 859 1.29e-08 GGTGAAAAGG GGATATGGGGGC CTTTTTTTTT
PF07_0080; 1032 1.61e-08 TAAAATTTTA GTGGAAAGAGGG GTGATTCAAT*
PFC0775w; 1149 2.45e-08 ATAAAAATAA GGAGATGAAGGG GTTAAAGGTA*
PFE1405c; 75 3.01e-08 TGAATTTATA AAGGTGGCGGGG CATACAAAAA
PFC0735w; 434 3.01e-08 TTTTTCCTAT GAACACACGGGG TTACAATTAA
PF08_0076; 791 3.71e-08 TTTTTTAAGT GATGAAAGGGGG CAAAAAACAA
PF08_0039; 922 5.50e-08 GAAATGAAAA AAAAAAGGGGGC TCTGTTTTCA
PF14_0141; 637 8.10e-08 TAAAATTTTA GAGGGTAAAGGG GTTGACATAT
PFD0245c; 587 8.10e-08 AAAGAAAAAA GAGCAGAAAGGG TTTACGGTAT
PFE0845c; 1204 9.88e-08 TTTTTAAAAC ATAGGGGGGAGG ACTTTTTTTT
PF14_0627; 644 9.88e-08 TATAATAATA AGAAATGCGGGG TGTCATGATA
PFD0770c; 936 9.88e-08 TTATATTAGT GAGATGGGAGGC TATGTTTCTC*
PFL0210c; 662 1.45e-07 GGCTTCATGT GGGTATGAAGGG AAGAAAAAAT
PF11_0260; 1521 1.45e-07 AATGCCAAGG GGATAAAAGGGG CTTTTTTTTG
PF11_0312; 1110 2.08e-07 AGTATGTAGT GAAAAAAGGGGA CATACAAATA
PF13_0049; 1613 2.48e-07 CCAAAAAAAA AGGGAAAAAGGG AAAAAGGAAA
PF13_0262; 893 4.98e-07 ATGATGATAT GTAGATGGGAGC CTTTTTTTTT
PF10_0149; 854 5.88e-07 TGAAGCCACA AAAAGTGGGGGA ATGTTTTTTA
PF13_0132; 242 8.26e-07 AAATAAAACA GCAAAAAGGTGG AAAAAAATAA
PF07_0043; 1783 9.72e-07 AAAAAAAAAA GAAAAAAAAGGG AGTTATATAT
PFE1085w; 520 9.72e-07 TTTTTTTTTT GGGGGTGCATGC AAGACATATA
PF13_0316; 1014 1.81e-06 TATATTAGTA AAGAAAGAAGGC TTTTTTGATT
PF14_0027; 260 1.81e-06 GAAGCAAAAA AAAGAAAAAGGG TTTTTAAAAT
PF14_0296; 739 1.81e-06 ATATATATAT GATTATAGGGGG GAATTTTTTT
PFB0545c; 820 2.12e-06 ATAGATAGAA GAAAAAAGAAGG CAAGAGAAAA
PFC0300c; 464 2.48e-06 AGATTACAAT AAAAAAGGAGGA AAAAATAAAA
PF13_0213; 392 2.88e-06 TTATAATTAA AAAGAAAGAGGA AATAATTATA
PF13_0177; 1523 3.35e-06 TTGTTAGAAA GTGAACAACGGG TTAACAAAAA
PF11_0245; 1469 3.35e-06 ATTCATAATT GAAGAAAAAGGA TTATATAAAG
PF11_0065; 544 3.35e-06 AAAATGAAAA AAACATGAAGGG TTTTTTTTTT
PF13_0129; 956 3.89e-06 AAAAATGTAG GGATAAAAGAGG TTATTTTTAT
MAL13P1.92; 468 3.89e-06 GACATATAAA AAAAATGGGTGG TATTATACTT
PF11_0106; 1520 3.89e-06 ATAAAAAAAA AAAGAAAGAAGG AAATATTTTT
PFF0345w; 199 3.89e-06 AAAAAAAAAA GAAAAAGAAAGG TATATTTTAT
PF08_0096; 366 3.89e-06 AAAAAAAAGA AAAGGAAAAGGC AACAACAAAA
PFB0830w; 522 3.89e-06 TGTGAAATAT ACAAATGGGAGC GTATAAAAAG
PF14_0563; 588 4.43e-06 TATATAAAAA AAATACAGGGGA ATTAAAATAA
PF14_0589; 1386 5.10e-06 TTAATTGTAT ATAAACAAGGGG TACAATAATA
PFF1095w; 1343 5.10e-06 AATTTCATTT GAGAAGGAAGGT ATTATTTTTA
PFB0885w; 304 5.10e-06 TCAAGCTATA AAATTAAGGGGG AATATTAAAC
MAL13P1.209; 416 5.87e-06 AGAAATTTAT ATATGAAGAGGG AATATATTTC
PF10_0038; 1313 5.87e-06 CTCAATAATT GAAGAGGCGGAA AAAAAAAATA
PF14_0240; 624 6.76e-06 TTTTTTCTAA ACAAATGGAAGG ACAATATAAA
PF14_0231; 1502 6.76e-06 GCACTTCTTA AGGAAAAGGAGA AGAAAATATC
PF14_0104; 1636 6.76e-06 AAAAAAAAAA AAAAACGGGAGA AATTATTTGA
PF13_0178; 260 6.76e-06 TGTACAATTA AAATAAAGAGGC GTTTCTTATA
PF07_0079; 1106 6.76e-06 AATACTCATA GAAGAAAGAAGA GTGTAAAGAA
PF14_0401; 10 7.81e-06 TCTTAAATG GGATAAAGAAGC ATTTATGAAC
PF13_0171; 189 7.81e-06 AAAAAAAAAA AAAAAAAGGTGG AATCTATAAA
MAL13P1.144; 492 7.81e-06 TACAATATTA AAGAGAGCGAGA AACAACTATT
PF11_0438; 514 7.81e-06 ATTACATTTT ATAGAAGGGAGA TTAAAGTGTA
PF10_0209; 588 7.81e-06 CCTAAAAATG AAAAAAAAAGGG TATATGGTTT
PF10_0187; 1268 8.94e-06 AATATTTAAT ATAAATGAGGGC CATATAAAAA
PFE0885w; 294 8.94e-06 TGTTATATTT GAAAGGAACGGA TTAAAAGGAA
PFD1070w; 183 8.94e-06 AAAAAAAAAA AAAAAAGGATGG AAAAAAATTA
PFC0295c; 293 8.94e-06 ATAATTATAA GTGTATAAAGGG AATAGCATTT*
PFC1020c; 673 1.01e-05 ACATTTTTAA GAAATTGGAGGA AATCATAATT
PF07_0088; 869 1.16e-05 GTGAGGATGT ATGTATAAGGGG TTTTTTTGGT
PF13_0268; 585 1.16e-05 TTGCGTAGGG GCATGAGGCTGC ATTGTAATTT
PF13_0228; 536 1.16e-05 ATATATATAT GTTGTTGGAGGG GGTGTAATTA
PFC0400w; 712 1.16e-05 CTTAAAAAAA AAAAAAAGAGGA AACCCTAAAC
PFB0455w; 102 1.16e-05 TATTTTGAAC GGAGTTGGCAGC AAAACGGATT
PFL2055w; 831 1.32e-05 TAAACTTACA GTGATGAGAGGA TATATATATA
PF14_0185; 1679 1.32e-05 CCTATAATTA AAGTAAAGAAGG AATATAATAT
PF13_0045; 653 1.32e-05 TGTTTGTGAT GAAATAAAAGGG TTAATTTATT
PF08_0075; 1739 1.32e-05 GATGTACATT GAATGAGAGAGA AAATAAAAAT
23

## Slide 24
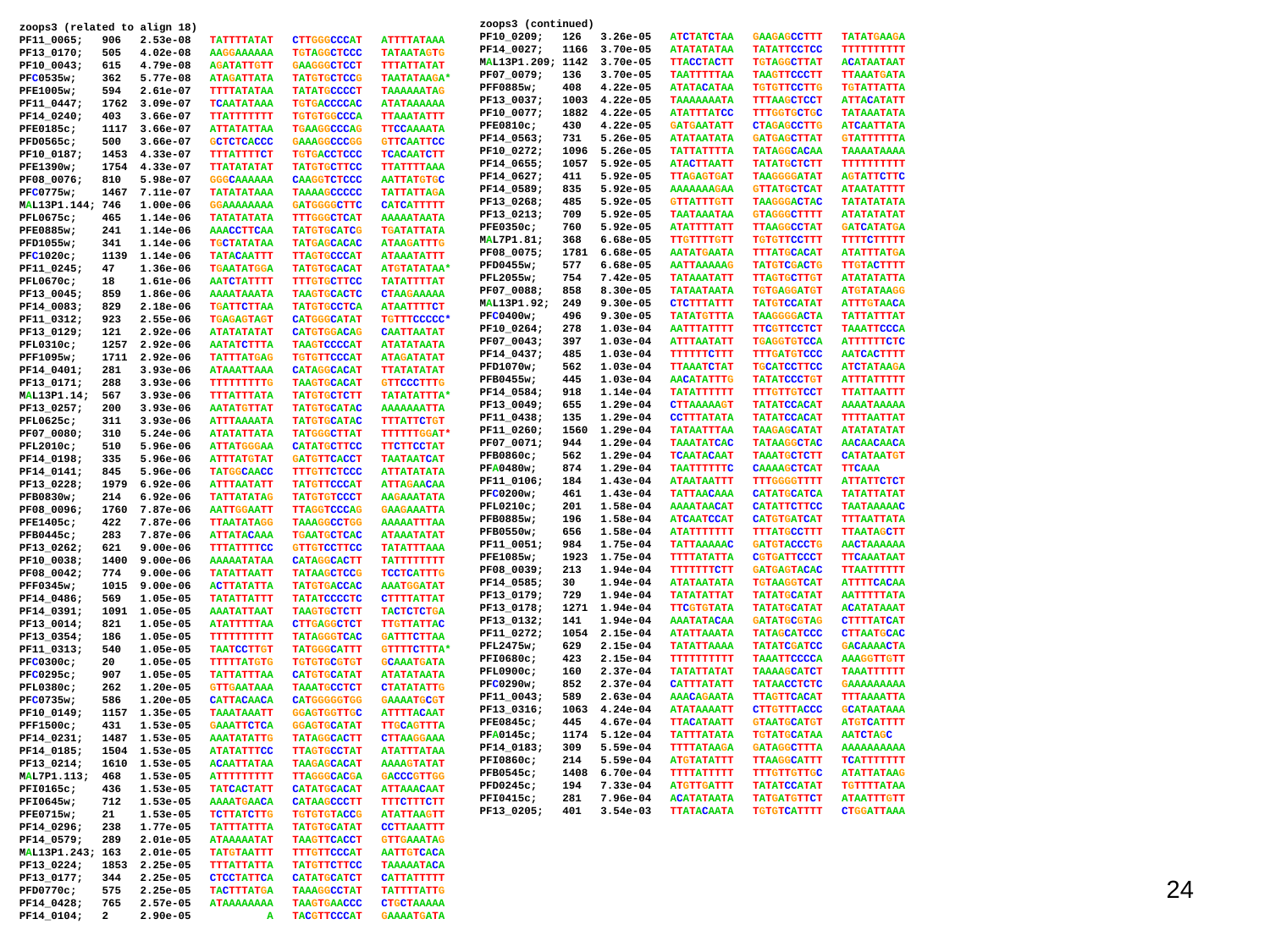

zoops3 (continued)
PF10_0209; 126 3.26e-05 ATCTATCTAA GAAGAGCCTTT TATATGAAGA
PF14_0027; 1166 3.70e-05 ATATATATAA TATATTCCTCC TTTTTTTTTT
MAL13P1.209; 1142 3.70e-05 TTACCTACTT TGTAGGCTTAT ACATAATAAT
PF07_0079; 136 3.70e-05 TAATTTTTAA TAAGTTCCCTT TTAAATGATA
PFF0885w; 408 4.22e-05 ATATACATAA TGTGTTCCTTG TGTATTATTA
PF13_0037; 1003 4.22e-05 TAAAAAAATA TTTAAGCTCCT ATTACATATT
PF10_0077; 1882 4.22e-05 ATATTTATCC TTTGGTGCTGC TATAAATATA
PFE0810c; 430 4.22e-05 GATGAATATT CTAGAGCCTTG ATCAATTATA
PF14_0563; 731 5.26e-05 ATATAATATA GATGAGCTTAT GTATTTTTTA
PF10_0272; 1096 5.26e-05 TATTATTTTA TATAGGCACAA TAAAATAAAA
PF14_0655; 1057 5.92e-05 ATACTTAATT TATATGCTCTT TTTTTTTTTT
PF14_0627; 411 5.92e-05 TTAGAGTGAT TAAGGGGATAT AGTATTCTTC
PF14_0589; 835 5.92e-05 AAAAAAAGAA GTTATGCTCAT ATAATATTTT
PF13_0268; 485 5.92e-05 GTTATTTGTT TAAGGGACTAC TATATATATA
PF13_0213; 709 5.92e-05 TAATAAATAA GTAGGGCTTTT ATATATATAT
PFE0350c; 760 5.92e-05 ATATTTTATT TTAAGGCCTAT GATCATATGA
MAL7P1.81; 368 6.68e-05 TTGTTTTGTT TGTGTTCCTTT TTTTCTTTTT
PF08_0075; 1781 6.68e-05 AATATGAATA TTTATGCACAT ATATTTATGA
PFD0455w; 577 6.68e-05 AATTAAAAAG TATGTCGACTG TTGTACTTTT
PFL2055w; 754 7.42e-05 TATAAATATT TTAGTGCTTGT ATATATATTA
PF07_0088; 858 8.30e-05 TATAATAATA TGTGAGGATGT ATGTATAAGG
MAL13P1.92; 249 9.30e-05 CTCTTTATTT TATGTCCATAT ATTTGTAACA
PFC0400w; 496 9.30e-05 TATATGTTTA TAAGGGGACTA TATTATTTAT
PF10_0264; 278 1.03e-04 AATTTATTTT TTCGTTCCTCT TAAATTCCCA
PF07_0043; 397 1.03e-04 ATTTAATATT TGAGGTGTCCA ATTTTTTCTC
PF14_0437; 485 1.03e-04 TTTTTTCTTT TTTGATGTCCC AATCACTTTT
PFD1070w; 562 1.03e-04 TTAAATCTAT TGCATCCTTCC ATCTATAAGA
PFB0455w; 445 1.03e-04 AACATATTTG TATATCCCTGT ATTTATTTTT
PF14_0584; 918 1.14e-04 TATATTTTTT TTTGTTGTCCT TTATTAATTT
PF13_0049; 655 1.29e-04 CTTAAAAAGT TATATCCACAT AAAATAAAAA
PF11_0438; 135 1.29e-04 CCTTTATATA TATATCCACAT TTTTAATTAT
PF11_0260; 1560 1.29e-04 TATAATTTAA TAAGAGCATAT ATATATATAT
PF07_0071; 944 1.29e-04 TAAATATCAC TATAAGGCTAC AACAACAACA
PFB0860c; 562 1.29e-04 TCAATACAAT TAAATGCTCTT CATATAATGT
PFA0480w; 874 1.29e-04 TAATTTTTTC CAAAAGCTCAT TTCAAA
PF11_0106; 184 1.43e-04 ATAATAATTT TTTGGGGTTTT ATTATTCTCT
PFC0200w; 461 1.43e-04 TATTAACAAA CATATGCATCA TATATTATAT
PFL0210c; 201 1.58e-04 AAAATAACAT CATATTCTTCC TAATAAAAAC
PFB0885w; 196 1.58e-04 ATCAATCCAT CATGTGATCAT TTTAATTATA
PFB0550w; 656 1.58e-04 ATATTTTTTT TTTATGCCTTT TTAATAGCTT
PF11_0051; 984 1.75e-04 TATTAAAAAC GATGTACCCTG AACTAAAAAA
PFE1085w; 1923 1.75e-04 TTTTATATTA CGTGATTCCCT TTCAAATAAT
PF08_0039; 213 1.94e-04 TTTTTTTCTT GATGAGTACAC TTAATTTTTT
PF14_0585; 30 1.94e-04 ATATAATATA TGTAAGGTCAT ATTTTCACAA
PF13_0179; 729 1.94e-04 TATATATTAT TATATGCATAT AATTTTTATA
PF13_0178; 1271 1.94e-04 TTCGTGTATA TATATGCATAT ACATATAAAT
PF13_0132; 141 1.94e-04 AAATATACAA GATATGCGTAG CTTTTATCAT
PF11_0272; 1054 2.15e-04 ATATTAAATA TATAGCATCCC CTTAATGCAC
PFL2475w; 629 2.15e-04 TATATTAAAA TATATCGATCC GACAAAACTA
PFI0680c; 423 2.15e-04 TTTTTTTTTT TAAATTCCCCA AAAGGTTGTT
PFL0900c; 160 2.37e-04 TATATTATAT TAAAAGCATCT TAAATTTTTT
PFC0290w; 852 2.37e-04 CATTTATATT TATAACCTCTC GAAAAAAAAA
PF11_0043; 589 2.63e-04 AAACAGAATA TTAGTTCACAT TTTAAAATTA
PF13_0316; 1063 4.24e-04 ATATAAAATT CTTGTTTACCC GCATAATAAA
PFE0845c; 445 4.67e-04 TTACATAATT GTAATGCATGT ATGTCATTTT
PFA0145c; 1174 5.12e-04 TATTTATATA TGTATGCATAA AATCTAGC
PF14_0183; 309 5.59e-04 TTTTATAAGA GATAGGCTTTA AAAAAAAAAA
PFI0860c; 214 5.59e-04 ATGTATATTT TTAAGGCATTT TCATTTTTTT
PFB0545c; 1408 6.70e-04 TTTTATTTTT TTTGTTGTTGC ATATTATAAG
PFD0245c; 194 7.33e-04 ATGTTGATTT TATATCCATAT TGTTTTATAA
PFI0415c; 281 7.96e-04 ACATATAATA TATGATGTTCT ATAATTTGTT
PF13_0205; 401 3.54e-03 TTATACAATA TGTGTCATTTT CTGGATTAAA
zoops3 (related to align 18)
PF11_0065; 906 2.53e-08 TATTTTATAT CTTGGGCCCAT ATTTTATAAA
PF13_0170; 505 4.02e-08 AAGGAAAAAA TGTAGGCTCCC TATAATAGTG
PF10_0043; 615 4.79e-08 AGATATTGTT GAAGGGCTCCT TTTATTATAT
PFC0535w; 362 5.77e-08 ATAGATTATA TATGTGCTCCG TAATATAAGA*
PFE1005w; 594 2.61e-07 TTTTATATAA TATATGCCCCT TAAAAAATAG
PF11_0447; 1762 3.09e-07 TCAATATAAA TGTGACCCCAC ATATAAAAAA
PF14_0240; 403 3.66e-07 TTATTTTTTT TGTGTGGCCCA TTAAATATTT
PFE0185c; 1117 3.66e-07 ATTATATTAA TGAAGGCCCAG TTCCAAAATA
PFD0565c; 500 3.66e-07 GCTCTCACCC GAAAGGCCCGG GTTCAATTCC
PF10_0187; 1453 4.33e-07 TTTATTTTCT TGTGACCTCCC TCACAATCTT
PFE1390w; 1754 4.33e-07 TTATATATAT TATGTGCTTCC TTATTTTAAA
PF08_0076; 810 5.98e-07 GGGCAAAAAA CAAGGTCTCCC AATTATGTGC
PFC0775w; 1467 7.11e-07 TATATATAAA TAAAAGCCCCC TATTATTAGA
MAL13P1.144; 746 1.00e-06 GGAAAAAAAA GATGGGGCTTC CATCATTTTT
PFL0675c; 465 1.14e-06 TATATATATA TTTGGGCTCAT AAAAATAATA
PFE0885w; 241 1.14e-06 AAACCTTCAA TATGTGCATCG TGATATTATA
PFD1055w; 341 1.14e-06 TGCTATATAA TATGAGCACAC ATAAGATTTG
PFC1020c; 1139 1.14e-06 TATACAATTT TTAGTGCCCAT ATAAATATTT
PF11_0245; 47 1.36e-06 TGAATATGGA TATGTGCACAT ATGTATATAA*
PFL0670c; 18 1.61e-06 AATCTATTTT TTTGTGCTTCC TATATTTTAT
PF13_0045; 859 1.86e-06 AAAATAAATA TAAGTGCACTC CTAAGAAAAA
PF14_0083; 829 2.18e-06 TGATTCTTAA TATGTGCCTCA ATAATTTTCT
PF11_0312; 923 2.55e-06 TGAGAGTAGT CATGGGCATAT TGTTTCCCCC*
PF13_0129; 121 2.92e-06 ATATATATAT CATGTGGACAG CAATTAATAT
PFL0310c; 1257 2.92e-06 AATATCTTTA TAAGTCCCCAT ATATATAATA
PFF1095w; 1711 2.92e-06 TATTTATGAG TGTGTTCCCAT ATAGATATAT
PF14_0401; 281 3.93e-06 ATAAATTAAA CATAGGCACAT TTATATATAT
PF13_0171; 288 3.93e-06 TTTTTTTTTG TAAGTGCACAT GTTCCCTTTG
MAL13P1.14; 567 3.93e-06 TTTATTTATA TATGTGCTCTT TATATATTTA*
PF13_0257; 200 3.93e-06 AATATGTTAT TATGTGCATAC AAAAAAATTA
PFL0625c; 311 3.93e-06 ATTTAAAATA TATGTGCATAC TTTATTCTGT
PF07_0080; 310 5.24e-06 ATATATTATA TATGGGCTTAT TTTTTTGGAT*
PFL2010c; 510 5.96e-06 ATTATGGGAA CATATGCTTCC TTCTTCCTAT
PF14_0198; 335 5.96e-06 ATTTATGTAT GATGTTCACCT TAATAATCAT
PF14_0141; 845 5.96e-06 TATGGCAACC TTTGTTCTCCC ATTATATATA
PF13_0228; 1979 6.92e-06 ATTTAATATT TATGTTCCCAT ATTAGAACAA
PFB0830w; 214 6.92e-06 TATTATATAG TATGTGTCCCT AAGAAATATA
PF08_0096; 1760 7.87e-06 AATTGGAATT TTAGGTCCCAG GAAGAAATTA
PFE1405c; 422 7.87e-06 TTAATATAGG TAAAGGCCTGG AAAAATTTAA
PFB0445c; 283 7.87e-06 ATTATACAAA TGAATGCTCAC ATAAATATAT
PF13_0262; 621 9.00e-06 TTTATTTTCC GTTGTCCTTCC TATATTTAAA
PF10_0038; 1400 9.00e-06 AAAAATATAA CATAGGCACTT TATTTTTTTT
PF08_0042; 774 9.00e-06 TATATTAATT TATAAGCTCCG TCCTCATTTG
PFF0345w; 1015 9.00e-06 ACTTATATTA TATGTGACCAC AAATGGATAT
PF14_0486; 569 1.05e-05 TATATTATTT TATATCCCCTC CTTTTATTAT
PF14_0391; 1091 1.05e-05 AAATATTAAT TAAGTGCTCTT TACTCTCTGA
PF13_0014; 821 1.05e-05 ATATTTTTAA CTTGAGGCTCT TTGTTATTAC
PF13_0354; 186 1.05e-05 TTTTTTTTTT TATAGGGTCAC GATTTCTTAA
PF11_0313; 540 1.05e-05 TAATCCTTGT TATGGGCATTT GTTTTCTTTA*
PFC0300c; 20 1.05e-05 TTTTTATGTG TGTGTGCGTGT GCAAATGATA
PFC0295c; 907 1.05e-05 TATTATTTAA CATGTGCATAT ATATATAATA
PFL0380c; 262 1.20e-05 GTTGAATAAA TAAATGCCTCT CTATATATTG
PFC0735w; 586 1.20e-05 CATTACAACA CATGGGGGTGG GAAAATGCGT
PF10_0149; 1157 1.35e-05 TAAATAAATT GGAGTGGTTGC ATTTTACAAT
PFF1500c; 431 1.53e-05 GAAATTCTCA GGAGTGCATAT TTGCAGTTTA
PF14_0231; 1487 1.53e-05 AAATATATTG TATAGGCACTT CTTAAGGAAA
PF14_0185; 1504 1.53e-05 ATATATTTCC TTAGTGCCTAT ATATTTATAA
PF13_0214; 1610 1.53e-05 ACAATTATAA TAAGAGCACAT AAAAGTATAT
MAL7P1.113; 468 1.53e-05 ATTTTTTTTT TTAGGGCACGA GACCCGTTGG
PFI0165c; 436 1.53e-05 TATCACTATT CATATGCACAT ATTAAACAAT
PFI0645w; 712 1.53e-05 AAAATGAACA CATAAGCCCTT TTTCTTTCTT
PFE0715w; 21 1.53e-05 TCTTATCTTG TGTGTGTACCG ATATTAAGTT
PF14_0296; 238 1.77e-05 TATTTATTTA TATGTGCATAT CCTTAAATTT
PF14_0579; 289 2.01e-05 ATAAAAATAT TAAGTTCACCT GTTGAAATAG
MAL13P1.243; 163 2.01e-05 TATGTAATTT TTTGTTCCCAT AATTGTCACA
PF13_0224; 1853 2.25e-05 TTTATTATTA TATGTTCTTCC TAAAAATACA
PF13_0177; 344 2.25e-05 CTCCTATTCA CATATGCATCT CATTATTTTT
PFD0770c; 575 2.25e-05 TACTTTATGA TAAAGGCCTAT TATTTTATTG
PF14_0428; 765 2.57e-05 ATAAAAAAAA TAAGTGAACCC CTGCTAAAAA
PF14_0104; 2 2.90e-05 A TACGTTCCCAT GAAAATGATA
24

## Slide 25
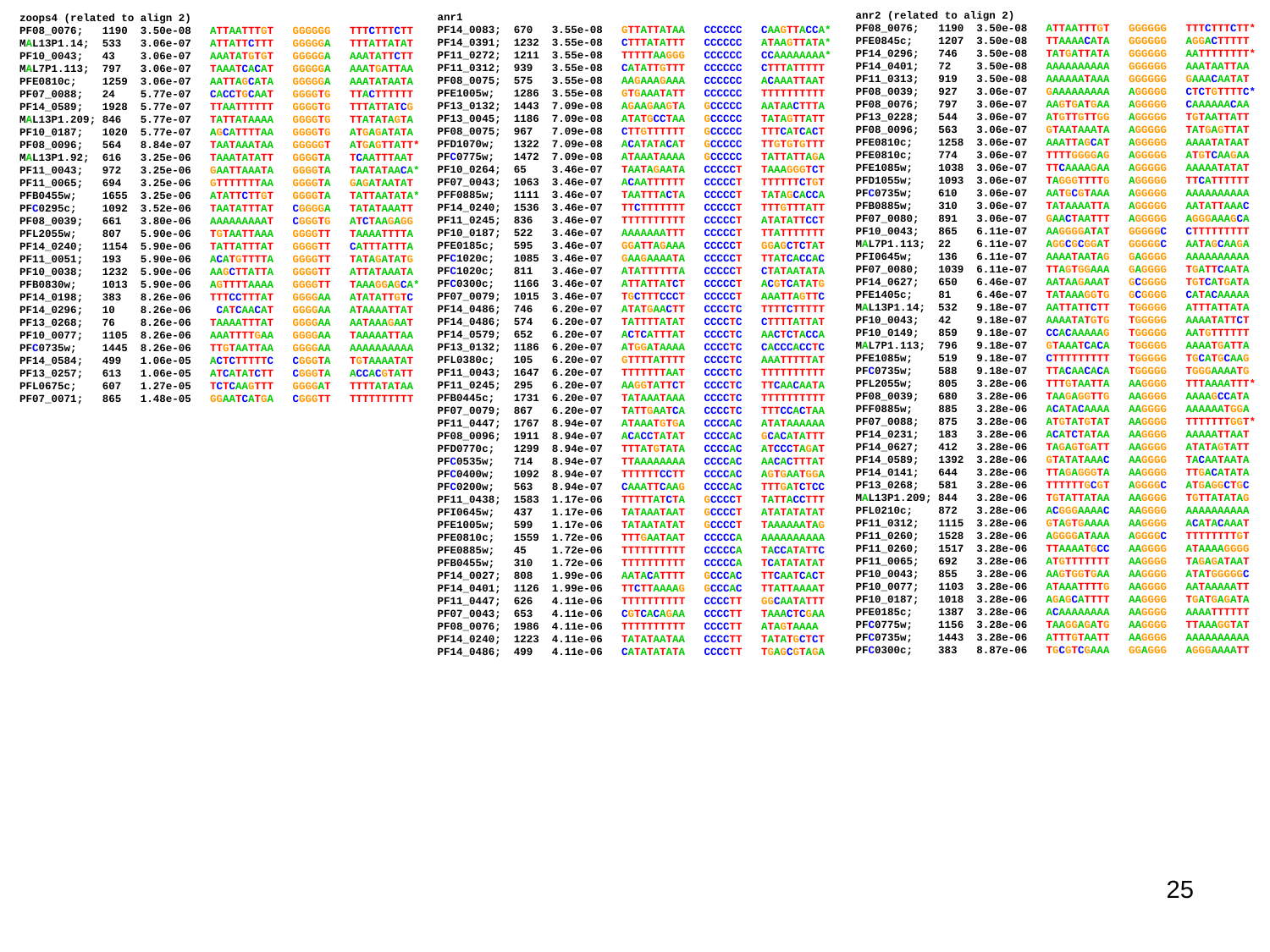

anr2 (related to align 2)
PF08_0076; 1190 3.50e-08 ATTAATTTGT GGGGGG TTTCTTTCTT*
PFE0845c; 1207 3.50e-08 TTAAAACATA GGGGGG AGGACTTTTT
PF14_0296; 746 3.50e-08 TATGATTATA GGGGGG AATTTTTTTT*
PF14_0401; 72 3.50e-08 AAAAAAAAAA GGGGGG AAATAATTAA
PF11_0313; 919 3.50e-08 AAAAAATAAA GGGGGG GAAACAATAT
PF08_0039; 927 3.06e-07 GAAAAAAAAA AGGGGG CTCTGTTTTC*
PF08_0076; 797 3.06e-07 AAGTGATGAA AGGGGG CAAAAAACAA
PF13_0228; 544 3.06e-07 ATGTTGTTGG AGGGGG TGTAATTATT
PF08_0096; 563 3.06e-07 GTAATAAATA AGGGGG TATGAGTTAT
PFE0810c; 1258 3.06e-07 AAATTAGCAT AGGGGG AAAATATAAT
PFE0810c; 774 3.06e-07 TTTTGGGGAG AGGGGG ATGTCAAGAA
PFE1085w; 1038 3.06e-07 TTCAAAAGAA AGGGGG AAAAATATAT
PFD1055w; 1093 3.06e-07 TAGGGTTTTG AGGGGG TTCATTTTTT
PFC0735w; 610 3.06e-07 AATGCGTAAA AGGGGG AAAAAAAAAA
PFB0885w; 310 3.06e-07 TATAAAATTA AGGGGG AATATTAAAC
PF07_0080; 891 3.06e-07 GAACTAATTT AGGGGG AGGGAAAGCA
PF10_0043; 865 6.11e-07 AAGGGGATAT GGGGGC CTTTTTTTTT
MAL7P1.113; 22 6.11e-07 AGGCGCGGAT GGGGGC AATAGCAAGA
PFI0645w; 136 6.11e-07 AAAATAATAG GAGGGG AAAAAAAAAA
PF07_0080; 1039 6.11e-07 TTAGTGGAAA GAGGGG TGATTCAATA
PF14_0627; 650 6.46e-07 AATAAGAAAT GCGGGG TGTCATGATA
PFE1405c; 81 6.46e-07 TATAAAGGTG GCGGGG CATACAAAAA
MAL13P1.14; 532 9.18e-07 AATTATTCTT TGGGGG ATTTATTATA
PF10_0043; 42 9.18e-07 AAAATATGTG TGGGGG AAAATATTCT
PF10_0149; 859 9.18e-07 CCACAAAAAG TGGGGG AATGTTTTTT
MAL7P1.113; 796 9.18e-07 GTAAATCACA TGGGGG AAAATGATTA
PFE1085w; 519 9.18e-07 CTTTTTTTTT TGGGGG TGCATGCAAG
PFC0735w; 588 9.18e-07 TTACAACACA TGGGGG TGGGAAAATG
PFL2055w; 805 3.28e-06 TTTGTAATTA AAGGGG TTTAAAATTT*
PF08_0039; 680 3.28e-06 TAAGAGGTTG AAGGGG AAAAGCCATA
PFF0885w; 885 3.28e-06 ACATACAAAA AAGGGG AAAAAATGGA
PF07_0088; 875 3.28e-06 ATGTATGTAT AAGGGG TTTTTTTGGT*
PF14_0231; 183 3.28e-06 ACATCTATAA AAGGGG AAAAATTAAT
PF14_0627; 412 3.28e-06 TAGAGTGATT AAGGGG ATATAGTATT
PF14_0589; 1392 3.28e-06 GTATATAAAC AAGGGG TACAATAATA
PF14_0141; 644 3.28e-06 TTAGAGGGTA AAGGGG TTGACATATA
PF13_0268; 581 3.28e-06 TTTTTTGCGT AGGGGC ATGAGGCTGC
MAL13P1.209; 844 3.28e-06 TGTATTATAA AAGGGG TGTTATATAG
PFL0210c; 872 3.28e-06 ACGGGAAAAC AAGGGG AAAAAAAAAA
PF11_0312; 1115 3.28e-06 GTAGTGAAAA AAGGGG ACATACAAAT
PF11_0260; 1528 3.28e-06 AGGGGATAAA AGGGGC TTTTTTTTGT
PF11_0260; 1517 3.28e-06 TTAAAATGCC AAGGGG ATAAAAGGGG
PF11_0065; 692 3.28e-06 ATGTTTTTTT AAGGGG TAGAGATAAT
PF10_0043; 855 3.28e-06 AAGTGGTGAA AAGGGG ATATGGGGGC
PF10_0077; 1103 3.28e-06 ATAAATTTTG AAGGGG AATAAAAATT
PF10_0187; 1018 3.28e-06 AGAGCATTTT AAGGGG TGATGAGATA
PFE0185c; 1387 3.28e-06 ACAAAAAAAA AAGGGG AAAATTTTTT
PFC0775w; 1156 3.28e-06 TAAGGAGATG AAGGGG TTAAAGGTAT
PFC0735w; 1443 3.28e-06 ATTTGTAATT AAGGGG AAAAAAAAAA
PFC0300c; 383 8.87e-06 TGCGTCGAAA GGAGGG AGGGAAAATT
anr1
PF14_0083; 670 3.55e-08 GTTATTATAA CCCCCC CAAGTTACCA*
PF14_0391; 1232 3.55e-08 CTTTATATTT CCCCCC ATAAGTTATA*
PF11_0272; 1211 3.55e-08 TTTTTAAGGG CCCCCC CCAAAAAAAA*
PF11_0312; 939 3.55e-08 CATATTGTTT CCCCCC CTTTATTTTT
PF08_0075; 575 3.55e-08 AAGAAAGAAA CCCCCC ACAAATTAAT
PFE1005w; 1286 3.55e-08 GTGAAATATT CCCCCC TTTTTTTTTT
PF13_0132; 1443 7.09e-08 AGAAGAAGTA GCCCCC AATAACTTTA
PF13_0045; 1186 7.09e-08 ATATGCCTAA GCCCCC TATAGTTATT
PF08_0075; 967 7.09e-08 CTTGTTTTTT GCCCCC TTTCATCACT
PFD1070w; 1322 7.09e-08 ACATATACAT GCCCCC TTGTGTGTTT
PFC0775w; 1472 7.09e-08 ATAAATAAAA GCCCCC TATTATTAGA
PF10_0264; 65 3.46e-07 TAATAGAATA CCCCCT TAAAGGGTCT
PF07_0043; 1063 3.46e-07 ACAATTTTTT CCCCCT TTTTTTCTGT
PFF0885w; 1111 3.46e-07 TAATTTACTA CCCCCT TATAGCACCA
PF14_0240; 1536 3.46e-07 TTCTTTTTTT CCCCCT TTTGTTTATT
PF11_0245; 836 3.46e-07 TTTTTTTTTT CCCCCT ATATATTCCT
PF10_0187; 522 3.46e-07 AAAAAAATTT CCCCCT TTATTTTTTT
PFE0185c; 595 3.46e-07 GGATTAGAAA CCCCCT GGAGCTCTAT
PFC1020c; 1085 3.46e-07 GAAGAAAATA CCCCCT TTATCACCAC
PFC1020c; 811 3.46e-07 ATATTTTTTA CCCCCT CTATAATATA
PFC0300c; 1166 3.46e-07 ATTATTATCT CCCCCT ACGTCATATG
PF07_0079; 1015 3.46e-07 TGCTTTCCCT CCCCCT AAATTAGTTC
PF14_0486; 746 6.20e-07 ATATGAACTT CCCCTC TTTTCTTTTT
PF14_0486; 574 6.20e-07 TATTTTATAT CCCCTC CTTTTATTAT
PF14_0579; 652 6.20e-07 ACTCATTTAT CCCCTC AACTCTACCA
PF13_0132; 1186 6.20e-07 ATGGATAAAA CCCCTC CACCCACCTC
PFL0380c; 105 6.20e-07 GTTTTATTTT CCCCTC AAATTTTTAT
PF11_0043; 1647 6.20e-07 TTTTTTTAAT CCCCTC TTTTTTTTTT
PF11_0245; 295 6.20e-07 AAGGTATTCT CCCCTC TTCAACAATA
PFB0445c; 1731 6.20e-07 TATAAATAAA CCCCTC TTTTTTTTTT
PF07_0079; 867 6.20e-07 TATTGAATCA CCCCTC TTTCCACTAA
PF11_0447; 1767 8.94e-07 ATAAATGTGA CCCCAC ATATAAAAAA
PF08_0096; 1911 8.94e-07 ACACCTATAT CCCCAC GCACATATTT
PFD0770c; 1299 8.94e-07 TTTATGTATA CCCCAC ATCCCTAGAT
PFC0535w; 714 8.94e-07 TTAAAAAAAA CCCCAC AACACTTTAT
PFC0400w; 1092 8.94e-07 TTTTTTCCTT CCCCAC AGTGAATGGA
PFC0200w; 563 8.94e-07 CAAATTCAAG CCCCAC TTTGATCTCC
PF11_0438; 1583 1.17e-06 TTTTTATCTA GCCCCT TATTACCTTT
PFI0645w; 437 1.17e-06 TATAAATAAT GCCCCT ATATATATAT
PFE1005w; 599 1.17e-06 TATAATATAT GCCCCT TAAAAAATAG
PFE0810c; 1559 1.72e-06 TTTGAATAAT CCCCCA AAAAAAAAAA
PFE0885w; 45 1.72e-06 TTTTTTTTTT CCCCCA TACCATATTC
PFB0455w; 310 1.72e-06 TTTTTTTTTT CCCCCA TCATATATAT
PF14_0027; 808 1.99e-06 AATACATTTT GCCCAC TTCAATCACT
PF14_0401; 1126 1.99e-06 TTCTTAAAAG GCCCAC TTATTAAAAT
PF11_0447; 626 4.11e-06 TTTTTTTTTT CCCCTT GGCAATATTT
PF07_0043; 653 4.11e-06 CGTCACAGAA CCCCTT TAAACTCGAA
PF08_0076; 1986 4.11e-06 TTTTTTTTTT CCCCTT ATAGTAAAA
PF14_0240; 1223 4.11e-06 TATATAATAA CCCCTT TATATGCTCT
PF14_0486; 499 4.11e-06 CATATATATA CCCCTT TGAGCGTAGA
zoops4 (related to align 2)
PF08_0076; 1190 3.50e-08 ATTAATTTGT GGGGGG TTTCTTTCTT
MAL13P1.14; 533 3.06e-07 ATTATTCTTT GGGGGA TTTATTATAT
PF10_0043; 43 3.06e-07 AAATATGTGT GGGGGA AAATATTCTT
MAL7P1.113; 797 3.06e-07 TAAATCACAT GGGGGA AAATGATTAA
PFE0810c; 1259 3.06e-07 AATTAGCATA GGGGGA AAATATAATA
PF07_0088; 24 5.77e-07 CACCTGCAAT GGGGTG TTACTTTTTT
PF14_0589; 1928 5.77e-07 TTAATTTTTT GGGGTG TTTATTATCG
MAL13P1.209; 846 5.77e-07 TATTATAAAA GGGGTG TTATATAGTA
PF10_0187; 1020 5.77e-07 AGCATTTTAA GGGGTG ATGAGATATA
PF08_0096; 564 8.84e-07 TAATAAATAA GGGGGT ATGAGTTATT*
MAL13P1.92; 616 3.25e-06 TAAATATATT GGGGTA TCAATTTAAT
PF11_0043; 972 3.25e-06 GAATTAAATA GGGGTA TAATATAACA*
PF11_0065; 694 3.25e-06 GTTTTTTTAA GGGGTA GAGATAATAT
PFB0455w; 1655 3.25e-06 ATATTCTTGT GGGGTA TATTAATATA*
PFC0295c; 1092 3.52e-06 TAATATTTAT CGGGGA TATATAAATT
PF08_0039; 661 3.80e-06 AAAAAAAAAT CGGGTG ATCTAAGAGG
PFL2055w; 807 5.90e-06 TGTAATTAAA GGGGTT TAAAATTTTA
PF14_0240; 1154 5.90e-06 TATTATTTAT GGGGTT CATTTATTTA
PF11_0051; 193 5.90e-06 ACATGTTTTA GGGGTT TATAGATATG
PF10_0038; 1232 5.90e-06 AAGCTTATTA GGGGTT ATTATAAATA
PFB0830w; 1013 5.90e-06 AGTTTTAAAA GGGGTT TAAAGGAGCA*
PF14_0198; 383 8.26e-06 TTTCCTTTAT GGGGAA ATATATTGTC
PF14_0296; 10 8.26e-06 CATCAACAT GGGGAA ATAAAATTAT
PF13_0268; 76 8.26e-06 TAAAATTTAT GGGGAA AATAAAGAAT
PF10_0077; 1105 8.26e-06 AAATTTTGAA GGGGAA TAAAAATTAA
PFC0735w; 1445 8.26e-06 TTGTAATTAA GGGGAA AAAAAAAAAA
PF14_0584; 499 1.06e-05 ACTCTTTTTC CGGGTA TGTAAAATAT
PF13_0257; 613 1.06e-05 ATCATATCTT CGGGTA ACCACGTATT
PFL0675c; 607 1.27e-05 TCTCAAGTTT GGGGAT TTTTATATAA
PF07_0071; 865 1.48e-05 GGAATCATGA CGGGTT TTTTTTTTTT
25

## Slide 26
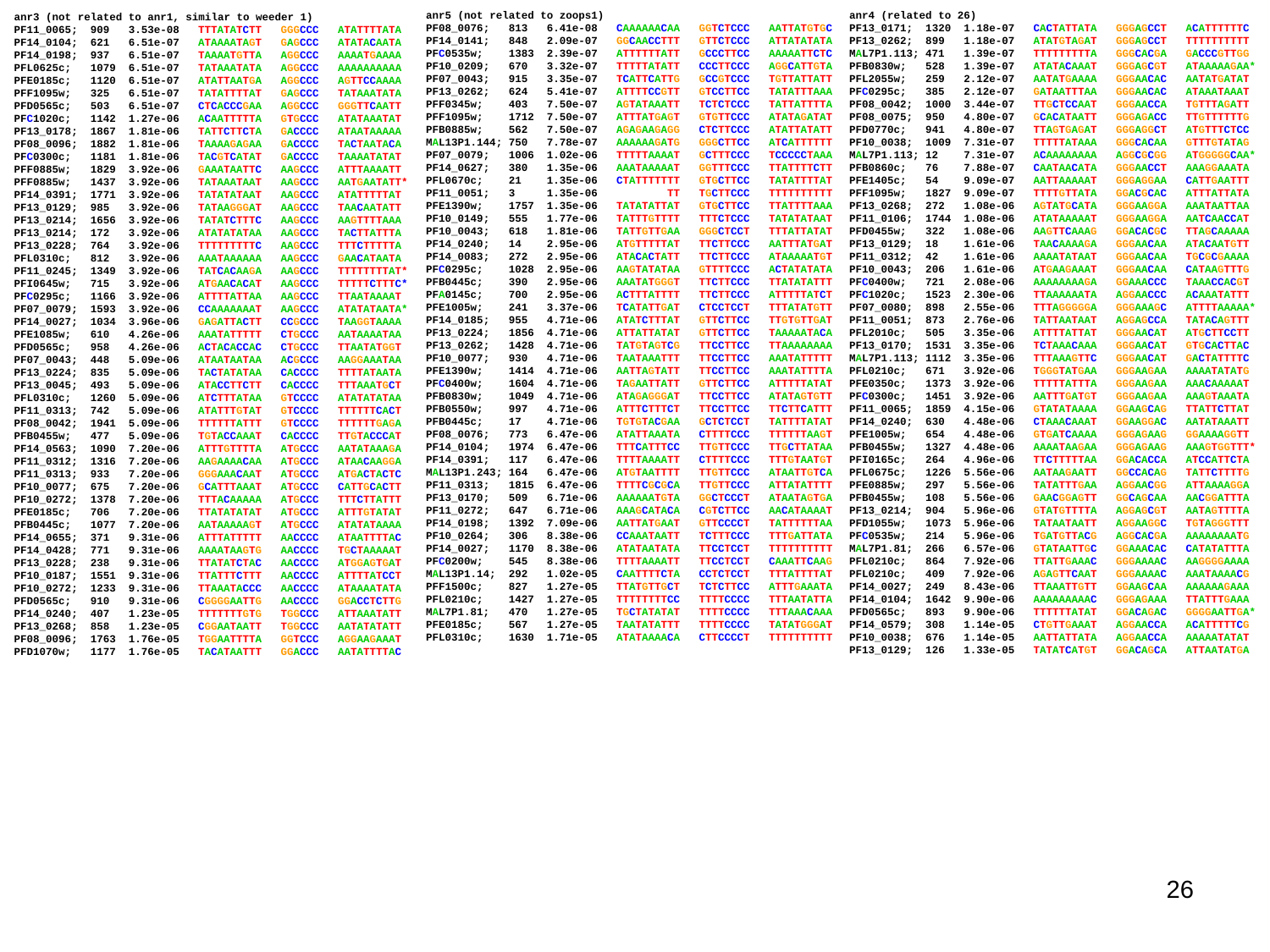

anr5 (not related to zoops1)
PF08_0076; 813 6.41e-08 CAAAAAACAA GGTCTCCC AATTATGTGC
PF14_0141; 848 2.09e-07 GGCAACCTTT GTTCTCCC ATTATATATA
PFC0535w; 1383 2.39e-07 ATTTTTTATT GCCCTTCC AAAAATTCTC
PF10_0209; 670 3.32e-07 TTTTTATATT CCCTTCCC AGGCATTGTA
PF07_0043; 915 3.35e-07 TCATTCATTG GCCGTCCC TGTTATTATT
PF13_0262; 624 5.41e-07 ATTTTCCGTT GTCCTTCC TATATTTAAA
PFF0345w; 403 7.50e-07 AGTATAAATT TCTCTCCC TATTATTTTA
PFF1095w; 1712 7.50e-07 ATTTATGAGT GTGTTCCC ATATAGATAT
PFB0885w; 562 7.50e-07 AGAGAAGAGG CTCTTCCC ATATTATATT
MAL13P1.144; 750 7.78e-07 AAAAAAGATG GGGCTTCC ATCATTTTTT
PF07_0079; 1006 1.02e-06 TTTTTAAAAT GCTTTCCC TCCCCCTAAA
PF14_0627; 380 1.35e-06 AAATAAAAAT GGTTTCCC TTATTTTCTT
PFL0670c; 21 1.35e-06 CTATTTTTTT GTGCTTCC TATATTTTAT
PF11_0051; 3 1.35e-06 TT TGCTTCCC TTTTTTTTTT
PFE1390w; 1757 1.35e-06 TATATATTAT GTGCTTCC TTATTTTAAA
PF10_0149; 555 1.77e-06 TATTTGTTTT TTTCTCCC TATATATAAT
PF10_0043; 618 1.81e-06 TATTGTTGAA GGGCTCCT TTTATTATAT
PF14_0240; 14 2.95e-06 ATGTTTTTAT TTCTTCCC AATTTATGAT
PF14_0083; 272 2.95e-06 ATACACTATT TTCTTCCC ATAAAAATGT
PFC0295c; 1028 2.95e-06 AAGTATATAA GTTTTCCC ACTATATATA
PFB0445c; 390 2.95e-06 AAATATGGGT TTCTTCCC TTATATATTT
PFA0145c; 700 2.95e-06 ACTTTATTTT TTCTTCCC ATTTTTATCT
PFE1005w; 241 3.37e-06 TCATATTGAT CTCCTCCT TTTATATGTT
PF14_0185; 955 4.71e-06 ATATCTTTAT GTTCTTCC TTGTGTTGAT
PF13_0224; 1856 4.71e-06 ATTATTATAT GTTCTTCC TAAAAATACA
PF13_0262; 1428 4.71e-06 TATGTAGTCG TTCCTTCC TTAAAAAAAA
PF10_0077; 930 4.71e-06 TAATAAATTT TTCCTTCC AAATATTTTT
PFE1390w; 1414 4.71e-06 AATTAGTATT TTCCTTCC AAATATTTTA
PFC0400w; 1604 4.71e-06 TAGAATTATT GTTCTTCC ATTTTTATAT
PFB0830w; 1049 4.71e-06 ATAGAGGGAT TTCCTTCC ATATAGTGTT
PFB0550w; 997 4.71e-06 ATTTCTTTCT TTCCTTCC TTCTTCATTT
PFB0445c; 17 4.71e-06 TGTGTACGAA GCTCTCCT TATTTTATAT
PF08_0076; 773 6.47e-06 ATATTAAATA CTTTTCCC TTTTTTAAGT
PF14_0104; 1974 6.47e-06 TTTCATTTCC TTGTTCCC TTGCTTATAA
PF14_0391; 117 6.47e-06 TTTTAAAATT CTTTTCCC TTTGTAATGT
MAL13P1.243; 164 6.47e-06 ATGTAATTTT TTGTTCCC ATAATTGTCA
PF11_0313; 1815 6.47e-06 TTTTCGCGCA TTGTTCCC ATTATATTTT
PF13_0170; 509 6.71e-06 AAAAAATGTA GGCTCCCT ATAATAGTGA
PF11_0272; 647 6.71e-06 AAAGCATACA CGTCTTCC AACATAAAAT
PF14_0198; 1392 7.09e-06 AATTATGAAT GTTCCCCT TATTTTTTAA
PF10_0264; 306 8.38e-06 CCAAATAATT TCTTTCCC TTTGATTATA
PF14_0027; 1170 8.38e-06 ATATAATATA TTCCTCCT TTTTTTTTTT
PFC0200w; 545 8.38e-06 TTTTAAAATT TTCCTCCT CAAATTCAAG
MAL13P1.14; 292 1.02e-05 CAATTTTCTA CCTCTCCT TTTATTTTAT
PFF1500c; 827 1.27e-05 TTATGTTGCT TCTCTTCC ATTTGAAATA
PFL0210c; 1427 1.27e-05 TTTTTTTTCC TTTTCCCC TTTAATATTA
MAL7P1.81; 470 1.27e-05 TGCTATATAT TTTTCCCC TTTAAACAAA
PFE0185c; 567 1.27e-05 TAATATATTT TTTTCCCC TATATGGGAT
PFL0310c; 1630 1.71e-05 ATATAAAACA CTTCCCCT TTTTTTTTTT
anr4 (related to 26)
PF13_0171; 1320 1.18e-07 CACTATTATA GGGAGCCT ACATTTTTTC
PF13_0262; 899 1.18e-07 ATATGTAGAT GGGAGCCT TTTTTTTTTT
MAL7P1.113; 471 1.39e-07 TTTTTTTTTA GGGCACGA GACCCGTTGG
PFB0830w; 528 1.39e-07 ATATACAAAT GGGAGCGT ATAAAAAGAA*
PFL2055w; 259 2.12e-07 AATATGAAAA GGGAACAC AATATGATAT
PFC0295c; 385 2.12e-07 GATAATTTAA GGGAACAC ATAAATAAAT
PF08_0042; 1000 3.44e-07 TTGCTCCAAT GGGAACCA TGTTTAGATT
PF08_0075; 950 4.80e-07 GCACATAATT GGGAGACC TTGTTTTTTG
PFD0770c; 941 4.80e-07 TTAGTGAGAT GGGAGGCT ATGTTTCTCC
PF10_0038; 1009 7.31e-07 TTTTTATAAA GGGCACAA GTTTGTATAG
MAL7P1.113; 12 7.31e-07 ACAAAAAAAA AGGCGCGG ATGGGGGCAA*
PFB0860c; 76 7.88e-07 CAATAACATA GGGAACCT AAAGGAAATA
PFE1405c; 54 9.09e-07 AATTAAAAAT GGGAGGAA CATTGAATTT
PFF1095w; 1827 9.09e-07 TTTTGTTATA GGACGCAC ATTTATTATA
PF13_0268; 272 1.08e-06 AGTATGCATA GGGAAGGA AAATAATTAA
PF11_0106; 1744 1.08e-06 ATATAAAAAT GGGAAGGA AATCAACCAT
PFD0455w; 322 1.08e-06 AAGTTCAAAG GGACACGC TTAGCAAAAA
PF13_0129; 18 1.61e-06 TAACAAAAGA GGGAACAA ATACAATGTT
PF11_0312; 42 1.61e-06 AAAATATAAT GGGAACAA TGCGCGAAAA
PF10_0043; 206 1.61e-06 ATGAAGAAAT GGGAACAA CATAAGTTTG
PFC0400w; 721 2.08e-06 AAAAAAAAGA GGAAACCC TAAACCACGT
PFC1020c; 1523 2.30e-06 TTAAAAAATA AGGAACCC ACAAATATTT
PF07_0080; 898 2.55e-06 TTTAGGGGGA GGGAAAGC ATTTTAAAAA*
PF11_0051; 873 2.76e-06 TATTAATAAT AGGAGCCA TATACAGTTT
PFL2010c; 505 3.35e-06 ATTTTATTAT GGGAACAT ATGCTTCCTT
PF13_0170; 1531 3.35e-06 TCTAAACAAA GGGAACAT GTGCACTTAC
MAL7P1.113; 1112 3.35e-06 TTTAAAGTTC GGGAACAT GACTATTTTC
PFL0210c; 671 3.92e-06 TGGGTATGAA GGGAAGAA AAAATATATG
PFE0350c; 1373 3.92e-06 TTTTTATTTA GGGAAGAA AAACAAAAAT
PFC0300c; 1451 3.92e-06 AATTTGATGT GGGAAGAA AAAGTAAATA
PF11_0065; 1859 4.15e-06 GTATATAAAA GGAAGCAG TTATTCTTAT
PF14_0240; 630 4.48e-06 CTAAACAAAT GGAAGGAC AATATAAATT
PFE1005w; 654 4.48e-06 GTGATCAAAA GGGAGAAG GGAAAAGGTT
PFB0455w; 1327 4.48e-06 AAAATAAGAA GGGAGAAG AAAGTGGTTT*
PFI0165c; 264 4.96e-06 TTCTTTTTAA GGACACCA ATCCATTCTA
PFL0675c; 1226 5.56e-06 AATAAGAATT GGCCACAG TATTCTTTTG
PFE0885w; 297 5.56e-06 TATATTTGAA AGGAACGG ATTAAAAGGA
PFB0455w; 108 5.56e-06 GAACGGAGTT GGCAGCAA AACGGATTTA
PF13_0214; 904 5.96e-06 GTATGTTTTA AGGAGCGT AATAGTTTTA
PFD1055w; 1073 5.96e-06 TATAATAATT AGGAAGGC TGTAGGGTTT
PFC0535w; 214 5.96e-06 TGATGTTACG AGGCACGA AAAAAAAATG
MAL7P1.81; 266 6.57e-06 GTATAATTGC GGAAACAC CATATATTTA
PFL0210c; 864 7.92e-06 TTATTGAAAC GGGAAAAC AAGGGGAAAA
PFL0210c; 409 7.92e-06 AGAGTTCAAT GGGAAAAC AAATAAAACG
PF14_0027; 249 8.43e-06 TTAAATTGTT GGAAGCAA AAAAAAGAAA
PF14_0104; 1642 9.90e-06 AAAAAAAAAC GGGAGAAA TTATTTGAAA
PFD0565c; 893 9.90e-06 TTTTTTATAT GGACAGAC GGGGAATTGA*
PF14_0579; 308 1.14e-05 CTGTTGAAAT AGGAACCA ACATTTTTCG
PF10_0038; 676 1.14e-05 AATTATTATA AGGAACCA AAAAATATAT
PF13_0129; 126 1.33e-05 TATATCATGT GGACAGCA ATTAATATGA
anr3 (not related to anr1, similar to weeder 1)
PF11_0065; 909 3.53e-08 TTTATATCTT GGGCCC ATATTTTATA
PF14_0104; 621 6.51e-07 ATAAAATAGT GAGCCC ATATACAATA
PF14_0198; 937 6.51e-07 TAAAATGTTA AGGCCC AAAATGAAAA
PFL0625c; 1079 6.51e-07 TATAAATATA AGGCCC AAAAAAAAAA
PFE0185c; 1120 6.51e-07 ATATTAATGA AGGCCC AGTTCCAAAA
PFF1095w; 325 6.51e-07 TATATTTTAT GAGCCC TATAAATATA
PFD0565c; 503 6.51e-07 CTCACCCGAA AGGCCC GGGTTCAATT
PFC1020c; 1142 1.27e-06 ACAATTTTTA GTGCCC ATATAAATAT
PF13_0178; 1867 1.81e-06 TATTCTTCTA GACCCC ATAATAAAAA
PF08_0096; 1882 1.81e-06 TAAAAGAGAA GACCCC TACTAATACA
PFC0300c; 1181 1.81e-06 TACGTCATAT GACCCC TAAAATATAT
PFF0885w; 1829 3.92e-06 GAAATAATTC AAGCCC ATTTAAAATT
PFF0885w; 1437 3.92e-06 TATAAATAAT AAGCCC AATGAATATT*
PF14_0391; 1771 3.92e-06 TATATATAAT AAGCCC ATATTTTTAT
PF13_0129; 985 3.92e-06 TATAAGGGAT AAGCCC TAACAATATT
PF13_0214; 1656 3.92e-06 TATATCTTTC AAGCCC AAGTTTTAAA
PF13_0214; 172 3.92e-06 ATATATATAA AAGCCC TACTTATTTA
PF13_0228; 764 3.92e-06 TTTTTTTTTC AAGCCC TTTCTTTTTA
PFL0310c; 812 3.92e-06 AAATAAAAAA AAGCCC GAACATAATA
PF11_0245; 1349 3.92e-06 TATCACAAGA AAGCCC TTTTTTTTAT*
PFI0645w; 715 3.92e-06 ATGAACACAT AAGCCC TTTTTCTTTC*
PFC0295c; 1166 3.92e-06 ATTTTATTAA AAGCCC TTAATAAAAT
PF07_0079; 1593 3.92e-06 CCAAAAAAAT AAGCCC ATATATAATA*
PF14_0027; 1034 3.96e-06 GAGATTACTT CCGCCC TAAGGTAAAA
PFE1085w; 610 4.26e-06 AAATATTTTT CTGCCC AATAAAATAA
PFD0565c; 958 4.26e-06 ACTACACCAC CTGCCC TTAATATGGT
PF07_0043; 448 5.09e-06 ATAATAATAA ACGCCC AAGGAAATAA
PF13_0224; 835 5.09e-06 TACTATATAA CACCCC TTTTATAATA
PF13_0045; 493 5.09e-06 ATACCTTCTT CACCCC TTTAAATGCT
PFL0310c; 1260 5.09e-06 ATCTTTATAA GTCCCC ATATATATAA
PF11_0313; 742 5.09e-06 ATATTTGTAT GTCCCC TTTTTTCACT
PF08_0042; 1941 5.09e-06 TTTTTTATTT GTCCCC TTTTTTGAGA
PFB0455w; 477 5.09e-06 TGTACCAAAT CACCCC TTGTACCCAT
PF14_0563; 1090 7.20e-06 ATTTGTTTTA ATGCCC AATATAAAGA
PF11_0312; 1316 7.20e-06 AAGAAAACAA ATGCCC ATAACAAGGA
PF11_0313; 933 7.20e-06 GGGAAACAAT ATGCCC ATGACTACTC
PF10_0077; 675 7.20e-06 GCATTTAAAT ATGCCC CATTGCACTT
PF10_0272; 1378 7.20e-06 TTTACAAAAA ATGCCC TTTCTTATTT
PFE0185c; 706 7.20e-06 TTATATATAT ATGCCC ATTTGTATAT
PFB0445c; 1077 7.20e-06 AATAAAAAGT ATGCCC ATATATAAAA
PF14_0655; 371 9.31e-06 ATTTATTTTT AACCCC ATAATTTTAC
PF14_0428; 771 9.31e-06 AAAATAAGTG AACCCC TGCTAAAAAT
PF13_0228; 238 9.31e-06 TTATATCTAC AACCCC ATGGAGTGAT
PF10_0187; 1551 9.31e-06 TTATTTCTTT AACCCC ATTTTATCCT
PF10_0272; 1233 9.31e-06 TTAAATACCC AACCCC ATAAAATATA
PFD0565c; 910 9.31e-06 CGGGGAATTG AACCCC GGACCTCTTG
PF14_0240; 407 1.23e-05 TTTTTTTGTG TGGCCC ATTAAATATT
PF13_0268; 858 1.23e-05 CGGAATAATT TGGCCC AATATATATT
PF08_0096; 1763 1.76e-05 TGGAATTTTA GGTCCC AGGAAGAAAT
PFD1070w; 1177 1.76e-05 TACATAATTT GGACCC AATATTTTAC
26

## Slide 27
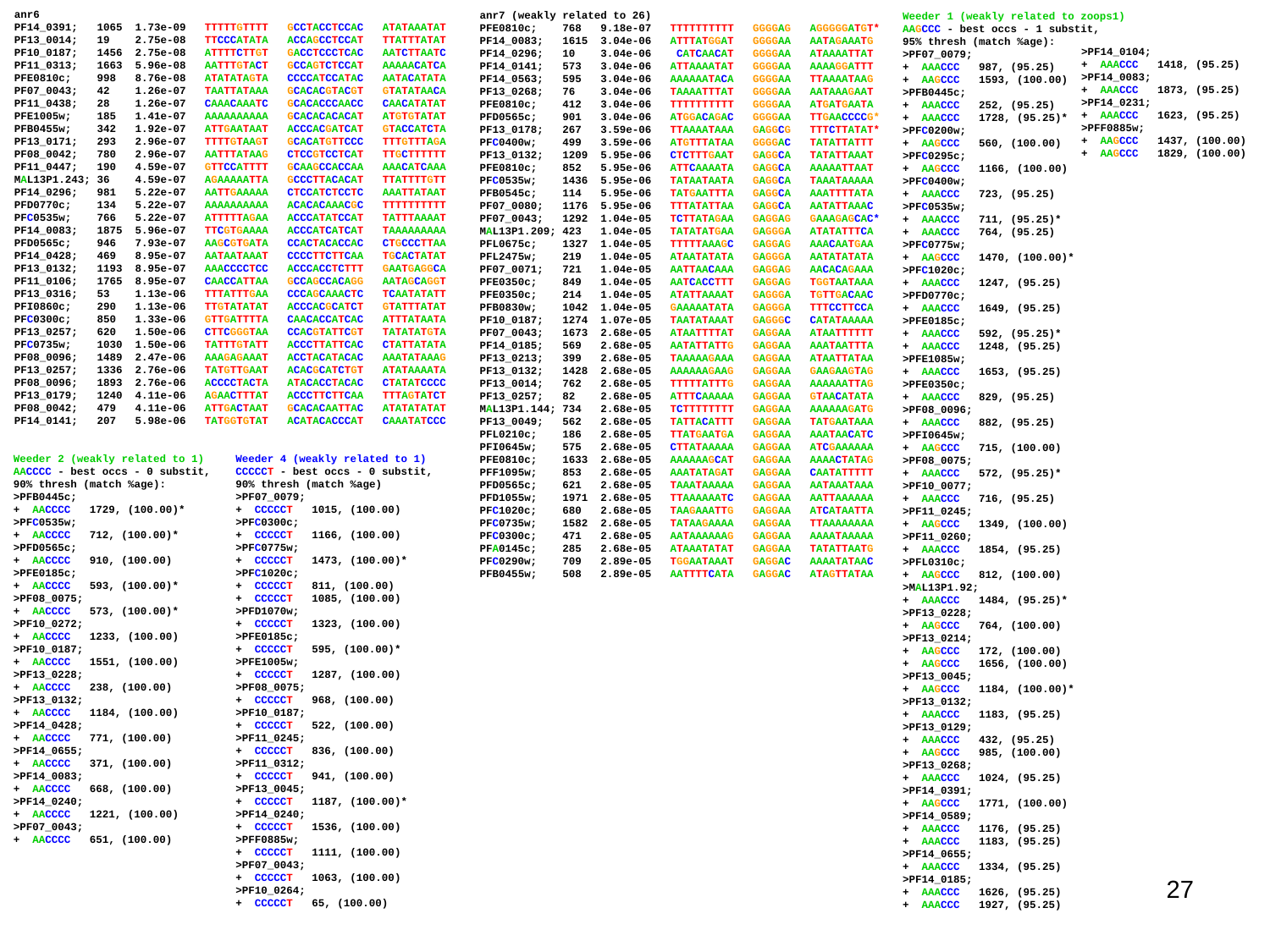

anr6
PF14_0391; 1065 1.73e-09 TTTTTGTTTT GCCTACCTCCAC ATATAAATAT
PF13_0014; 19 2.75e-08 TTCCCATATA ACCAGCCTCCAT TTATTTATAT
PF10_0187; 1456 2.75e-08 ATTTTCTTGT GACCTCCCTCAC AATCTTAATC
PF11_0313; 1663 5.96e-08 AATTTGTACT GCCAGTCTCCAT AAAAACATCA
PFE0810c; 998 8.76e-08 ATATATAGTA CCCCATCCATAC AATACATATA
PF07_0043; 42 1.26e-07 TAATTATAAA GCACACGTACGT GTATATAACA
PF11_0438; 28 1.26e-07 CAAACAAATC GCACACCCAACC CAACATATAT
PFE1005w; 185 1.41e-07 AAAAAAAAAA GCACACACACAT ATGTGTATAT
PFB0455w; 342 1.92e-07 ATTGAATAAT ACCCACGATCAT GTACCATCTA
PF13_0171; 293 2.96e-07 TTTTGTAAGT GCACATGTTCCC TTTGTTTAGA
PF08_0042; 780 2.96e-07 AATTTATAAG CTCCGTCCTCAT TTGCTTTTTT
PF11_0447; 190 4.59e-07 GTTCCATTTT GCAAGCCACCAA AAACATCAAA
MAL13P1.243; 36 4.59e-07 AGAAAAATTA GCCCTTACACAT TTATTTTGTT
PF14_0296; 981 5.22e-07 AATTGAAAAA CTCCATCTCCTC AAATTATAAT
PFD0770c; 134 5.22e-07 AAAAAAAAAA ACACACAAACGC TTTTTTTTTT
PFC0535w; 766 5.22e-07 ATTTTTAGAA ACCCATATCCAT TATTTAAAAT
PF14_0083; 1875 5.96e-07 TTCGTGAAAA ACCCATCATCAT TAAAAAAAAA
PFD0565c; 946 7.93e-07 AAGCGTGATA CCACTACACCAC CTGCCCTTAA
PF14_0428; 469 8.95e-07 AATAATAAAT CCCCTTCTTCAA TGCACTATAT
PF13_0132; 1193 8.95e-07 AAACCCCTCC ACCCACCTCTTT GAATGAGGCA
PF11_0106; 1765 8.95e-07 CAACCATTAA GCCAGCCACAGG AATAGCAGGT
PF13_0316; 53 1.13e-06 TTTATTTGAA CCCAGCAAACTC TCAATATATT
PFI0860c; 290 1.13e-06 TTGTATATAT ACCCACGCATCT GTATTTATAT
PFC0300c; 850 1.33e-06 GTTGATTTTA CAACACCATCAC ATTTATAATA
PF13_0257; 620 1.50e-06 CTTCGGGTAA CCACGTATTCGT TATATATGTA
PFC0735w; 1030 1.50e-06 TATTTGTATT ACCCTTATTCAC CTATTATATA
PF08_0096; 1489 2.47e-06 AAAGAGAAAT ACCTACATACAC AAATATAAAG
PF13_0257; 1336 2.76e-06 TATGTTGAAT ACACGCATCTGT ATATAAAATA
PF08_0096; 1893 2.76e-06 ACCCCTACTA ATACACCTACAC CTATATCCCC
PF13_0179; 1240 4.11e-06 AGAACTTTAT ACCCTTCTTCAA TTTAGTATCT
PF08_0042; 479 4.11e-06 ATTGACTAAT GCACACAATTAC ATATATATAT
PF14_0141; 207 5.98e-06 TATGGTGTAT ACATACACCCAT CAAATATCCC
anr7 (weakly related to 26)
PFE0810c; 768 9.18e-07 TTTTTTTTTT GGGGAG AGGGGGATGT*
PF14_0083; 1615 3.04e-06 ATTTATGGAT GGGGAA AATAGAAATG
PF14_0296; 10 3.04e-06 CATCAACAT GGGGAA ATAAAATTAT
PF14_0141; 573 3.04e-06 ATTAAAATAT GGGGAA AAAAGGATTT
PF14_0563; 595 3.04e-06 AAAAAATACA GGGGAA TTAAAATAAG
PF13_0268; 76 3.04e-06 TAAAATTTAT GGGGAA AATAAAGAAT
PFE0810c; 412 3.04e-06 TTTTTTTTTT GGGGAA ATGATGAATA
PFD0565c; 901 3.04e-06 ATGGACAGAC GGGGAA TTGAACCCCG*
PF13_0178; 267 3.59e-06 TTAAAATAAA GAGGCG TTTCTTATAT*
PFC0400w; 499 3.59e-06 ATGTTTATAA GGGGAC TATATTATTT
PF13_0132; 1209 5.95e-06 CTCTTTGAAT GAGGCA TATATTAAAT
PFE0810c; 852 5.95e-06 ATTCAAAATA GAGGCA AAAAATTAAT
PFC0535w; 1436 5.95e-06 TATAATAATA GAGGCA TAAATAAAAA
PFB0545c; 114 5.95e-06 TATGAATTTA GAGGCA AAATTTTATA
PF07_0080; 1176 5.95e-06 TTTATATTAA GAGGCA AATATTAAAC
PF07_0043; 1292 1.04e-05 TCTTATAGAA GAGGAG GAAAGAGCAC*
MAL13P1.209; 423 1.04e-05 TATATATGAA GAGGGA ATATATTTCA
PFL0675c; 1327 1.04e-05 TTTTTAAAGC GAGGAG AAACAATGAA
PFL2475w; 219 1.04e-05 ATAATATATA GAGGGA AATATATATA
PF07_0071; 721 1.04e-05 AATTAACAAA GAGGAG AACACAGAAA
PFE0350c; 849 1.04e-05 AATCACCTTT GAGGAG TGGTAATAAA
PFE0350c; 214 1.04e-05 ATATTAAAAT GAGGGA TGTTGACAAC
PFB0830w; 1042 1.04e-05 GAAAAATATA GAGGGA TTTCCTTCCA
PF10_0187; 1274 1.07e-05 TAATATAAAT GAGGGC CATATAAAAA
PF07_0043; 1673 2.68e-05 ATAATTTTAT GAGGAA ATAATTTTTT
PF14_0185; 569 2.68e-05 AATATTATTG GAGGAA AAATAATTTA
PF13_0213; 399 2.68e-05 TAAAAAGAAA GAGGAA ATAATTATAA
PF13_0132; 1428 2.68e-05 AAAAAAGAAG GAGGAA GAAGAAGTAG
PF13_0014; 762 2.68e-05 TTTTTATTTG GAGGAA AAAAAATTAG
PF13_0257; 82 2.68e-05 ATTTCAAAAA GAGGAA GTAACATATA
MAL13P1.144; 734 2.68e-05 TCTTTTTTTT GAGGAA AAAAAAGATG
PF13_0049; 562 2.68e-05 TATTACATTT GAGGAA TATGAATAAA
PFL0210c; 186 2.68e-05 TTATGAATGA GAGGAA AAATAACATC
PFI0645w; 575 2.68e-05 CTTATAAAAA GAGGAA ATCGAAAAAA
PFE0810c; 1633 2.68e-05 AAAAAAGCAT GAGGAA AAAACTATAG
PFF1095w; 853 2.68e-05 AAATATAGAT GAGGAA CAATATTTTT
PFD0565c; 621 2.68e-05 TAAATAAAAA GAGGAA AATAAATAAA
PFD1055w; 1971 2.68e-05 TTAAAAAATC GAGGAA AATTAAAAAA
PFC1020c; 680 2.68e-05 TAAGAAATTG GAGGAA ATCATAATTA
PFC0735w; 1582 2.68e-05 TATAAGAAAA GAGGAA TTAAAAAAAA
PFC0300c; 471 2.68e-05 AATAAAAAAG GAGGAA AAAATAAAAA
PFA0145c; 285 2.68e-05 ATAAATATAT GAGGAA TATATTAATG
PFC0290w; 709 2.89e-05 TGGAATAAAT GAGGAC AAAATATAAC
PFB0455w; 508 2.89e-05 AATTTTCATA GAGGAC ATAGTTATAA
Weeder 1 (weakly related to zoops1)
AAGCCC - best occs - 1 substit,
95% thresh (match %age):
>PF07_0079;
+ AAACCC 987, (95.25)
+ AAGCCC 1593, (100.00)
>PFB0445c;
+ AAACCC 252, (95.25)
+ AAACCC 1728, (95.25)*
>PFC0200w;
+ AAGCCC 560, (100.00)
>PFC0295c;
+ AAGCCC 1166, (100.00)
>PFC0400w;
+ AAACCC 723, (95.25)
>PFC0535w;
+ AAACCC 711, (95.25)*
+ AAACCC 764, (95.25)
>PFC0775w;
+ AAGCCC 1470, (100.00)*
>PFC1020c;
+ AAACCC 1247, (95.25)
>PFD0770c;
+ AAACCC 1649, (95.25)
>PFE0185c;
+ AAACCC 592, (95.25)*
+ AAACCC 1248, (95.25)
>PFE1085w;
+ AAACCC 1653, (95.25)
>PFE0350c;
+ AAACCC 829, (95.25)
>PF08_0096;
+ AAACCC 882, (95.25)
>PFI0645w;
+ AAGCCC 715, (100.00)
>PF08_0075;
+ AAACCC 572, (95.25)*
>PF10_0077;
+ AAACCC 716, (95.25)
>PF11_0245;
+ AAGCCC 1349, (100.00)
>PF11_0260;
+ AAACCC 1854, (95.25)
>PFL0310c;
+ AAGCCC 812, (100.00)
>MAL13P1.92;
+ AAACCC 1484, (95.25)*
>PF13_0228;
+ AAGCCC 764, (100.00)
>PF13_0214;
+ AAGCCC 172, (100.00)
+ AAGCCC 1656, (100.00)
>PF13_0045;
+ AAGCCC 1184, (100.00)*
>PF13_0132;
+ AAACCC 1183, (95.25)
>PF13_0129;
+ AAACCC 432, (95.25)
+ AAGCCC 985, (100.00)
>PF13_0268;
+ AAACCC 1024, (95.25)
>PF14_0391;
+ AAGCCC 1771, (100.00)
>PF14_0589;
+ AAACCC 1176, (95.25)
+ AAACCC 1183, (95.25)
>PF14_0655;
+ AAACCC 1334, (95.25)
>PF14_0185;
+ AAACCC 1626, (95.25)
+ AAACCC 1927, (95.25)
>PF14_0104;
+ AAACCC 1418, (95.25)
>PF14_0083;
+ AAACCC 1873, (95.25)
>PF14_0231;
+ AAACCC 1623, (95.25)
>PFF0885w;
+ AAGCCC 1437, (100.00)
+ AAGCCC 1829, (100.00)
Weeder 2 (weakly related to 1)
AACCCC - best occs - 0 substit,
90% thresh (match %age):
>PFB0445c;
+ AACCCC 1729, (100.00)*
>PFC0535w;
+ AACCCC 712, (100.00)*
>PFD0565c;
+ AACCCC 910, (100.00)
>PFE0185c;
+ AACCCC 593, (100.00)*
>PF08_0075;
+ AACCCC 573, (100.00)*
>PF10_0272;
+ AACCCC 1233, (100.00)
>PF10_0187;
+ AACCCC 1551, (100.00)
>PF13_0228;
+ AACCCC 238, (100.00)
>PF13_0132;
+ AACCCC 1184, (100.00)
>PF14_0428;
+ AACCCC 771, (100.00)
>PF14_0655;
+ AACCCC 371, (100.00)
>PF14_0083;
+ AACCCC 668, (100.00)
>PF14_0240;
+ AACCCC 1221, (100.00)
>PF07_0043;
+ AACCCC 651, (100.00)
Weeder 4 (weakly related to 1)
CCCCCT - best occs - 0 substit,
90% thresh (match %age)
>PF07_0079;
+ CCCCCT 1015, (100.00)
>PFC0300c;
+ CCCCCT 1166, (100.00)
>PFC0775w;
+ CCCCCT 1473, (100.00)*
>PFC1020c;
+ CCCCCT 811, (100.00)
+ CCCCCT 1085, (100.00)
>PFD1070w;
+ CCCCCT 1323, (100.00)
>PFE0185c;
+ CCCCCT 595, (100.00)*
>PFE1005w;
+ CCCCCT 1287, (100.00)
>PF08_0075;
+ CCCCCT 968, (100.00)
>PF10_0187;
+ CCCCCT 522, (100.00)
>PF11_0245;
+ CCCCCT 836, (100.00)
>PF11_0312;
+ CCCCCT 941, (100.00)
>PF13_0045;
+ CCCCCT 1187, (100.00)*
>PF14_0240;
+ CCCCCT 1536, (100.00)
>PFF0885w;
+ CCCCCT 1111, (100.00)
>PF07_0043;
+ CCCCCT 1063, (100.00)
>PF10_0264;
+ CCCCCT 65, (100.00)
27

## Slide 28
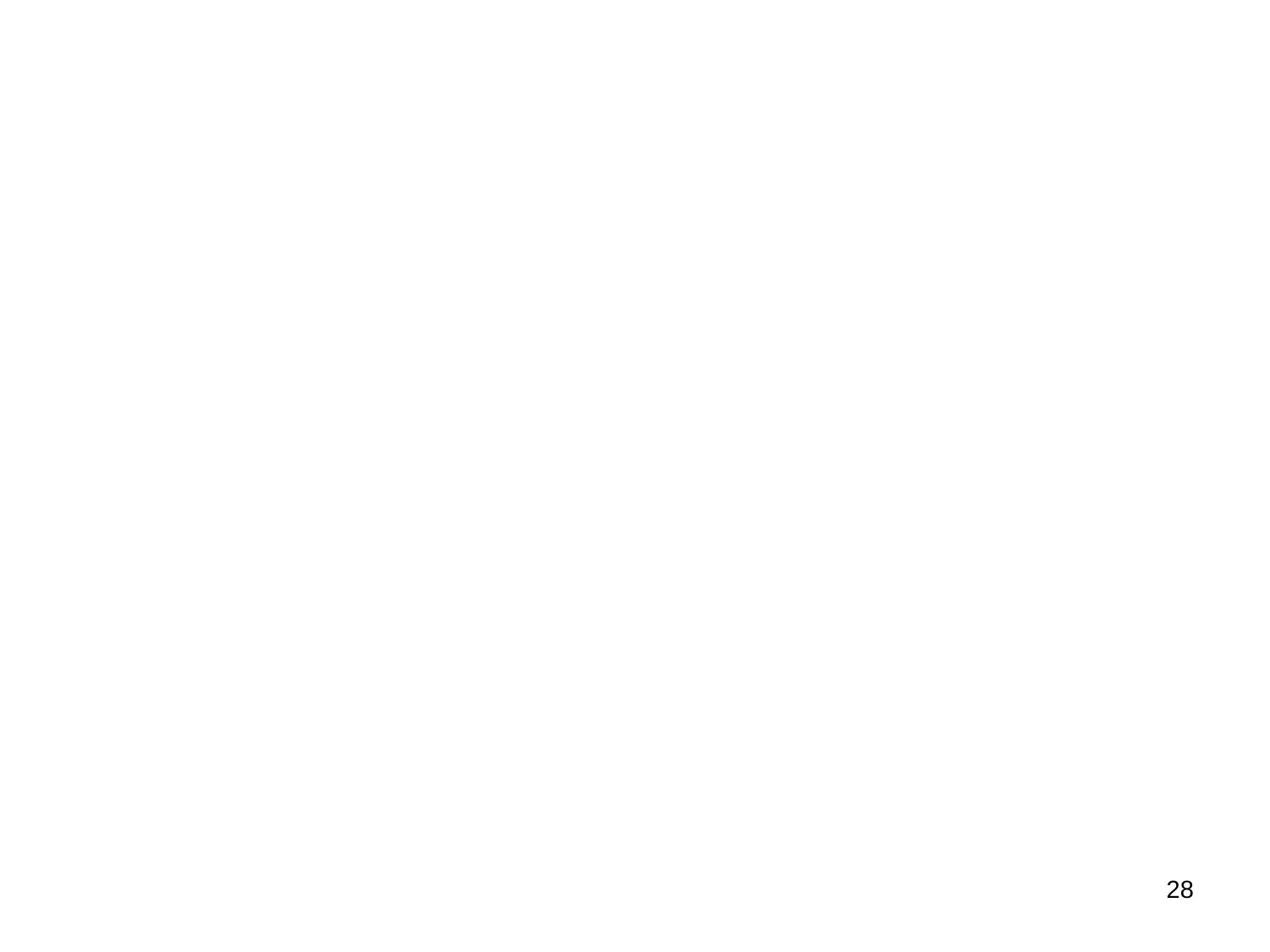

28

## Slide 29
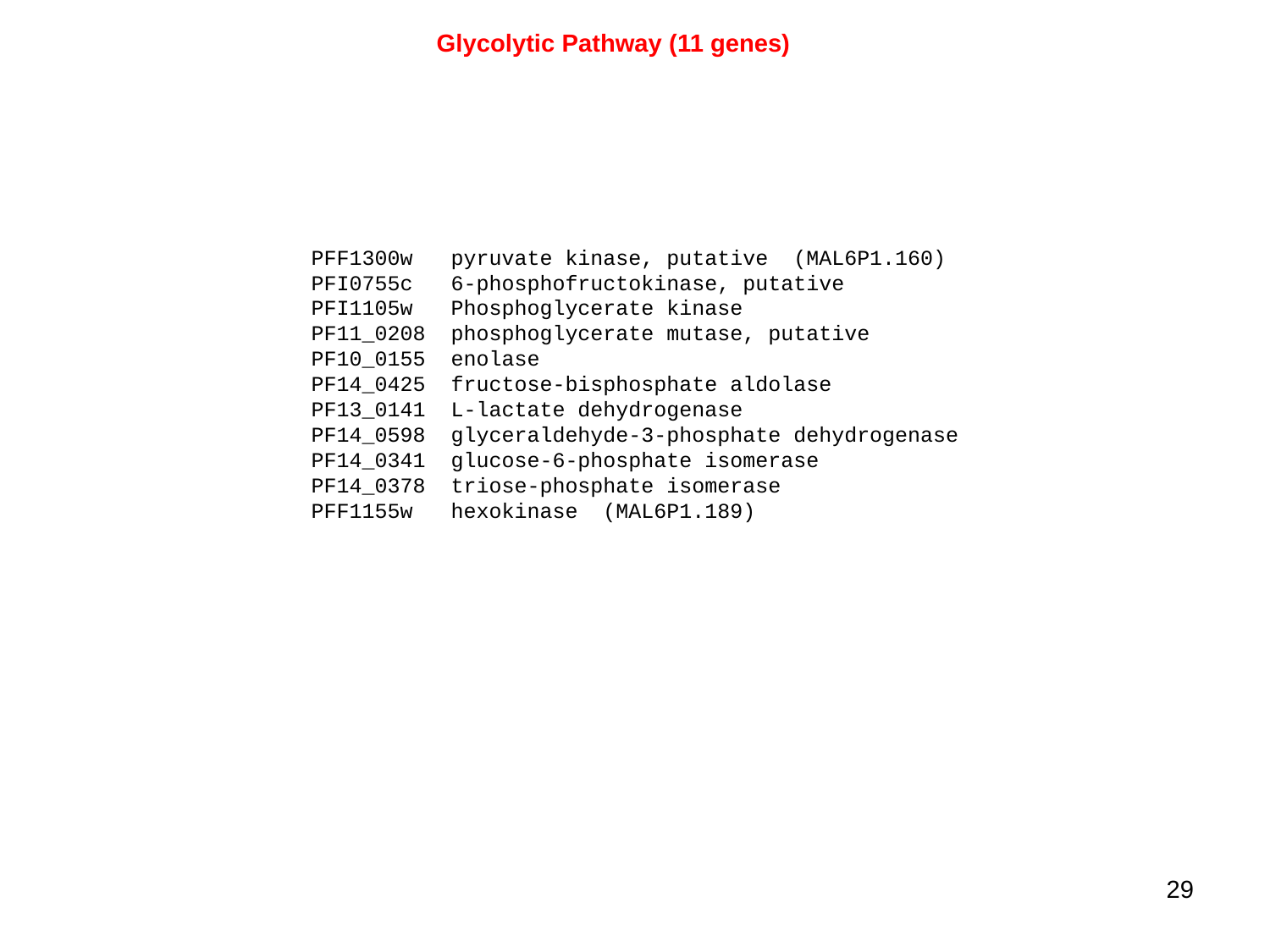

Glycolytic Pathway (11 genes)
PFF1300w pyruvate kinase, putative (MAL6P1.160)
PFI0755c 6-phosphofructokinase, putative
PFI1105w Phosphoglycerate kinase
PF11_0208 phosphoglycerate mutase, putative
PF10_0155 enolase
PF14_0425 fructose-bisphosphate aldolase
PF13_0141 L-lactate dehydrogenase
PF14_0598 glyceraldehyde-3-phosphate dehydrogenase
PF14_0341 glucose-6-phosphate isomerase
PF14_0378 triose-phosphate isomerase
PFF1155w hexokinase (MAL6P1.189)
29

## Slide 30
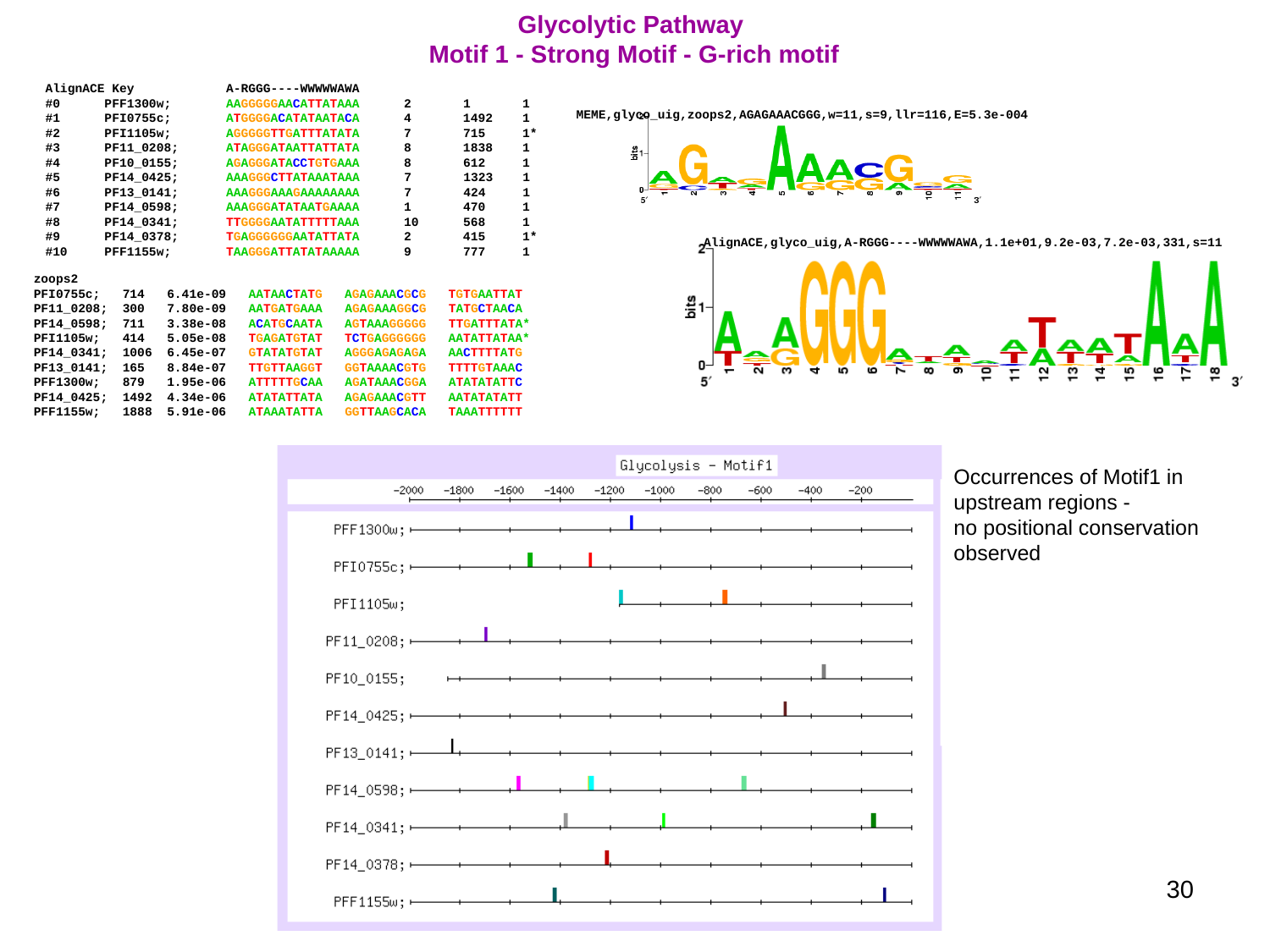

Glycolytic Pathway
Motif 1 - Strong Motif - G-rich motif
AlignACE Key
#0 PFF1300w;
#1 PFI0755c;
#2 PFI1105w;
#3 PF11_0208;
#4 PF10_0155;
#5 PF14_0425;
#6 PF13_0141;
#7 PF14_0598;
#8 PF14_0341;
#9 PF14_0378;
#10 PFF1155w;
A-RGGG----WWWWWAWA
AAGGGGGAACATTATAAA 2 1 1
ATGGGGACATATAATACA 4 1492 1
AGGGGGTTGATTTATATA 7 715 1*
ATAGGGATAATTATTATA 8 1838 1
AGAGGGATACCTGTGAAA 8 612 1
AAAGGGCTTATAAATAAA 7 1323 1
AAAGGGAAAGAAAAAAAA 7 424 1
AAAGGGATATAATGAAAA 1 470 1
TTGGGGAATATTTTTAAA 10 568 1
TGAGGGGGGAATATTATA 2 415 1*
TAAGGGATTATATAAAAA 9 777 1
MEME,glyco_uig,zoops2,AGAGAAACGGG,w=11,s=9,llr=116,E=5.3e-004
AlignACE,glyco_uig,A-RGGG----WWWWWAWA,1.1e+01,9.2e-03,7.2e-03,331,s=11
zoops2
PFI0755c; 714 6.41e-09 AATAACTATG AGAGAAACGCG TGTGAATTAT
PF11_0208; 300 7.80e-09 AATGATGAAA AGAGAAAGGCG TATGCTAACA
PF14_0598; 711 3.38e-08 ACATGCAATA AGTAAAGGGGG TTGATTTATA*
PFI1105w; 414 5.05e-08 TGAGATGTAT TCTGAGGGGGG AATATTATAA*
PF14_0341; 1006 6.45e-07 GTATATGTAT AGGGAGAGAGA AACTTTTATG
PF13_0141; 165 8.84e-07 TTGTTAAGGT GGTAAAACGTG TTTTGTAAAC
PFF1300w; 879 1.95e-06 ATTTTTGCAA AGATAAACGGA ATATATATTC
PF14_0425; 1492 4.34e-06 ATATATTATA AGAGAAACGTT AATATATATT
PFF1155w; 1888 5.91e-06 ATAAATATTA GGTTAAGCACA TAAATTTTTT
Occurrences of Motif1 in
upstream regions -
no positional conservation
observed
30

## Slide 31
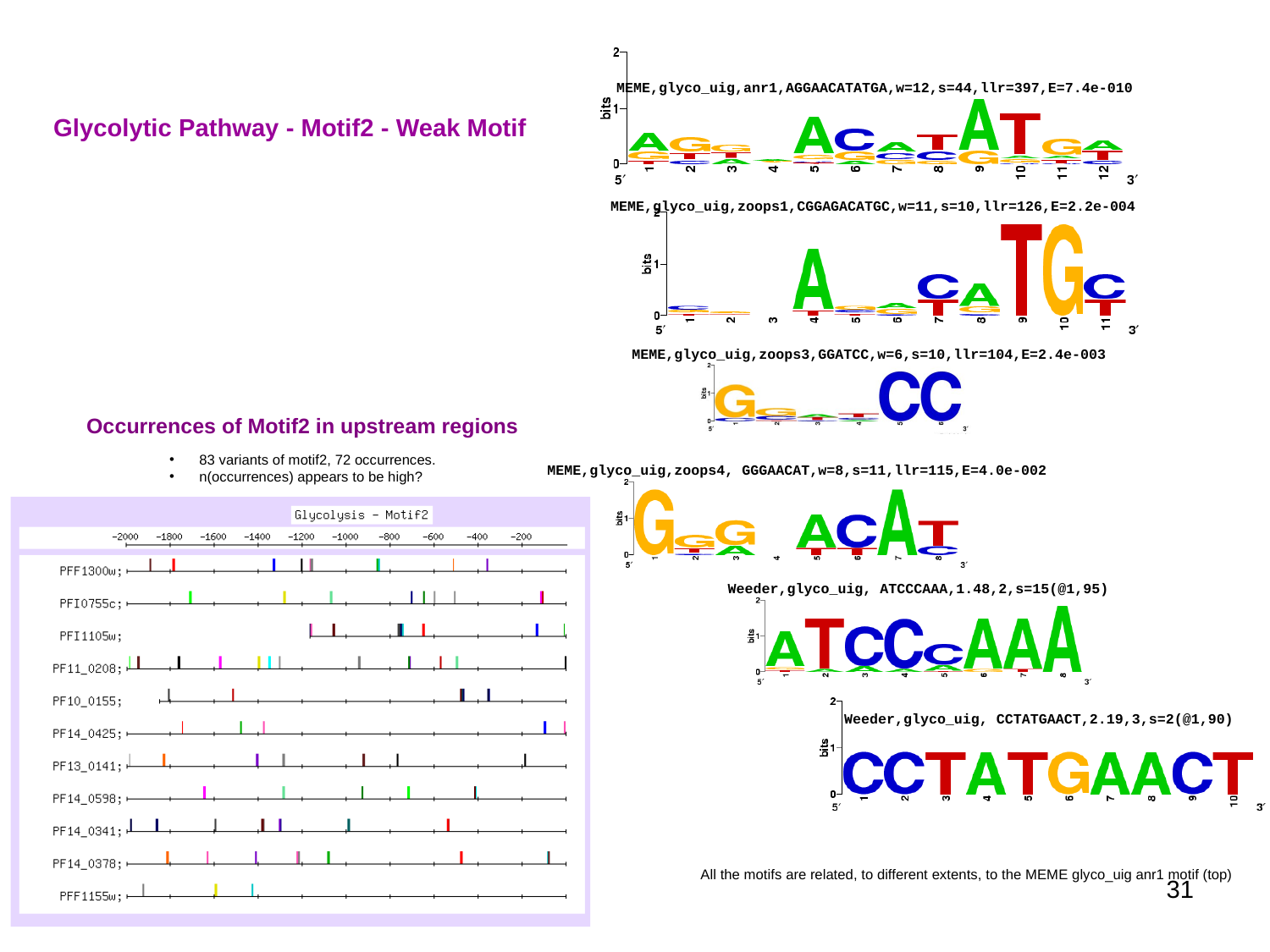

MEME,glyco_uig,anr1,AGGAACATATGA,w=12,s=44,llr=397,E=7.4e-010
MEME,glyco_uig,zoops1,CGGAGACATGC,w=11,s=10,llr=126,E=2.2e-004
MEME,glyco_uig,zoops3,GGATCC,w=6,s=10,llr=104,E=2.4e-003
MEME,glyco_uig,zoops4, GGGAACAT,w=8,s=11,llr=115,E=4.0e-002
Weeder,glyco_uig, ATCCCAAA,1.48,2,s=15(@1,95)
Weeder,glyco_uig, CCTATGAACT,2.19,3,s=2(@1,90)
Glycolytic Pathway - Motif2 - Weak Motif
Occurrences of Motif2 in upstream regions
83 variants of motif2, 72 occurrences.
n(occurrences) appears to be high?
All the motifs are related, to different extents, to the MEME glyco_uig anr1 motif (top)
31

## Slide 32
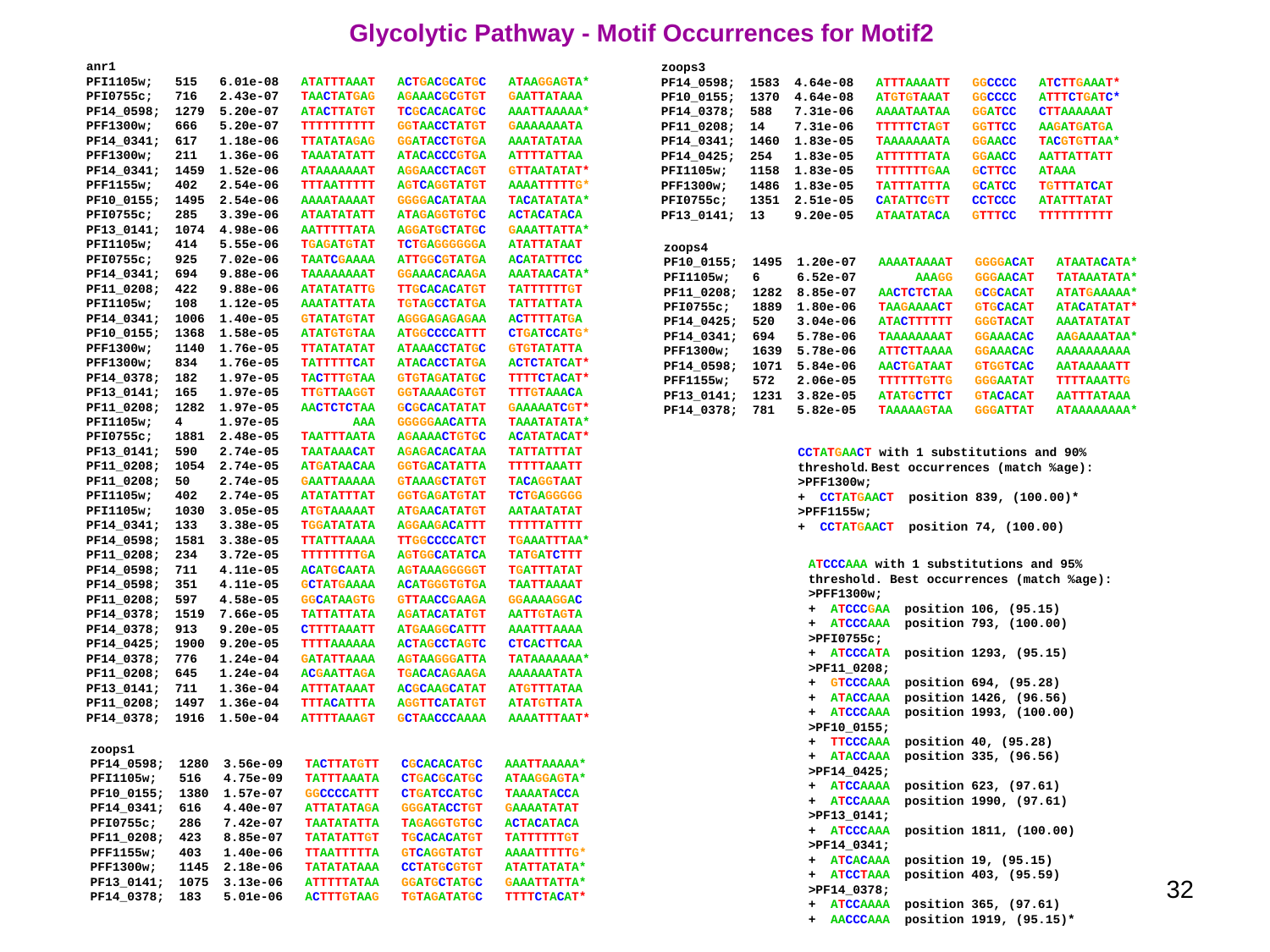

Glycolytic Pathway - Motif Occurrences for Motif2
anr1
PFI1105w; 515 6.01e-08 ATATTTAAAT ACTGACGCATGC ATAAGGAGTA*
PFI0755c; 716 2.43e-07 TAACTATGAG AGAAACGCGTGT GAATTATAAA
PF14_0598; 1279 5.20e-07 ATACTTATGT TCGCACACATGC AAATTAAAAA*
PFF1300w; 666 5.20e-07 TTTTTTTTTT GGTAACCTATGT GAAAAAAATA
PF14_0341; 617 1.18e-06 TTATATAGAG GGATACCTGTGA AAATATATAA
PFF1300w; 211 1.36e-06 TAAATATATT ATACACCCGTGA ATTTTATTAA
PF14_0341; 1459 1.52e-06 ATAAAAAAAT AGGAACCTACGT GTTAATATAT*
PFF1155w; 402 2.54e-06 TTTAATTTTT AGTCAGGTATGT AAAATTTTTG*
PF10_0155; 1495 2.54e-06 AAAATAAAAT GGGGACATATAA TACATATATA*
PFI0755c; 285 3.39e-06 ATAATATATT ATAGAGGTGTGC ACTACATACA
PF13_0141; 1074 4.98e-06 AATTTTTATA AGGATGCTATGC GAAATTATTA*
PFI1105w; 414 5.55e-06 TGAGATGTAT TCTGAGGGGGGA ATATTATAAT
PFI0755c; 925 7.02e-06 TAATCGAAAA ATTGGCGTATGA ACATATTTCC
PF14_0341; 694 9.88e-06 TAAAAAAAAT GGAAACACAAGA AAATAACATA*
PF11_0208; 422 9.88e-06 ATATATATTG TTGCACACATGT TATTTTTTGT
PFI1105w; 108 1.12e-05 AAATATTATA TGTAGCCTATGA TATTATTATA
PF14_0341; 1006 1.40e-05 GTATATGTAT AGGGAGAGAGAA ACTTTTATGA
PF10_0155; 1368 1.58e-05 ATATGTGTAA ATGGCCCCATTT CTGATCCATG*
PFF1300w; 1140 1.76e-05 TTATATATAT ATAAACCTATGC GTGTATATTA
PFF1300w; 834 1.76e-05 TATTTTTCAT ATACACCTATGA ACTCTATCAT*
PF14_0378; 182 1.97e-05 TACTTTGTAA GTGTAGATATGC TTTTCTACAT*
PF13_0141; 165 1.97e-05 TTGTTAAGGT GGTAAAACGTGT TTTGTAAACA
PF11_0208; 1282 1.97e-05 AACTCTCTAA GCGCACATATAT GAAAAATCGT*
PFI1105w; 4 1.97e-05 AAA GGGGGAACATTA TAAATATATA*
PFI0755c; 1881 2.48e-05 TAATTTAATA AGAAAACTGTGC ACATATACAT*
PF13_0141; 590 2.74e-05 TAATAAACAT AGAGACACATAA TATTATTTAT
PF11_0208; 1054 2.74e-05 ATGATAACAA GGTGACATATTA TTTTTAAATT
PF11_0208; 50 2.74e-05 GAATTAAAAA GTAAAGCTATGT TACAGGTAAT
PFI1105w; 402 2.74e-05 ATATATTTAT GGTGAGATGTAT TCTGAGGGGG
PFI1105w; 1030 3.05e-05 ATGTAAAAAT ATGAACATATGT AATAATATAT
PF14_0341; 133 3.38e-05 TGGATATATA AGGAAGACATTT TTTTTATTTT
PF14_0598; 1581 3.38e-05 TTATTTAAAA TTGGCCCCATCT TGAAATTTAA*
PF11_0208; 234 3.72e-05 TTTTTTTTGA AGTGGCATATCA TATGATCTTT
PF14_0598; 711 4.11e-05 ACATGCAATA AGTAAAGGGGGT TGATTTATAT
PF14_0598; 351 4.11e-05 GCTATGAAAA ACATGGGTGTGA TAATTAAAAT
PF11_0208; 597 4.58e-05 GGCATAAGTG GTTAACCGAAGA GGAAAAGGAC
PF14_0378; 1519 7.66e-05 TATTATTATA AGATACATATGT AATTGTAGTA
PF14_0378; 913 9.20e-05 CTTTTAAATT ATGAAGGCATTT AAATTTAAAA
PF14_0425; 1900 9.20e-05 TTTTAAAAAA ACTAGCCTAGTC CTCACTTCAA
PF14_0378; 776 1.24e-04 GATATTAAAA AGTAAGGGATTA TATAAAAAAA*
PF11_0208; 645 1.24e-04 ACGAATTAGA TGACACAGAAGA AAAAAATATA
PF13_0141; 711 1.36e-04 ATTTATAAAT ACGCAAGCATAT ATGTTTATAA
PF11_0208; 1497 1.36e-04 TTTACATTTA AGGTTCATATGT ATATGTTATA
PF14_0378; 1916 1.50e-04 ATTTTAAAGT GCTAACCCAAAA AAAATTTAAT*
zoops3
PF14_0598; 1583 4.64e-08 ATTTAAAATT GGCCCC ATCTTGAAAT*
PF10_0155; 1370 4.64e-08 ATGTGTAAAT GGCCCC ATTTCTGATC*
PF14_0378; 588 7.31e-06 AAAATAATAA GGATCC CTTAAAAAAT
PF11_0208; 14 7.31e-06 TTTTTCTAGT GGTTCC AAGATGATGA
PF14_0341; 1460 1.83e-05 TAAAAAAATA GGAACC TACGTGTTAA*
PF14_0425; 254 1.83e-05 ATTTTTTATA GGAACC AATTATTATT
PFI1105w; 1158 1.83e-05 TTTTTTTGAA GCTTCC ATAAA
PFF1300w; 1486 1.83e-05 TATTTATTTA GCATCC TGTTTATCAT
PFI0755c; 1351 2.51e-05 CATATTCGTT CCTCCC ATATTTATAT
PF13_0141; 13 9.20e-05 ATAATATACA GTTTCC TTTTTTTTTT
zoops4
PF10_0155; 1495 1.20e-07 AAAATAAAAT GGGGACAT ATAATACATA*
PFI1105w; 6 6.52e-07 AAAGG GGGAACAT TATAAATATA*
PF11_0208; 1282 8.85e-07 AACTCTCTAA GCGCACAT ATATGAAAAA*
PFI0755c; 1889 1.80e-06 TAAGAAAACT GTGCACAT ATACATATAT*
PF14_0425; 520 3.04e-06 ATACTTTTTT GGGTACAT AAATATATAT
PF14_0341; 694 5.78e-06 TAAAAAAAAT GGAAACAC AAGAAAATAA*
PFF1300w; 1639 5.78e-06 ATTCTTAAAA GGAAACAC AAAAAAAAAA
PF14_0598; 1071 5.84e-06 AACTGATAAT GTGGTCAC AATAAAAATT
PFF1155w; 572 2.06e-05 TTTTTTGTTG GGGAATAT TTTTAAATTG
PF13_0141; 1231 3.82e-05 ATATGCTTCT GTACACAT AATTTATAAA
PF14_0378; 781 5.82e-05 TAAAAAGTAA GGGATTAT ATAAAAAAAA*
CCTATGAACT with 1 substitutions and 90%
threshold. Best occurrences (match %age):
>PFF1300w;
+ CCTATGAACT position 839, (100.00)*
>PFF1155w;
+ CCTATGAACT position 74, (100.00)
ATCCCAAA with 1 substitutions and 95%
threshold. Best occurrences (match %age):
>PFF1300w;
+ ATCCCGAA position 106, (95.15)
+ ATCCCAAA position 793, (100.00)
>PFI0755c;
+ ATCCCATA position 1293, (95.15)
>PF11_0208;
+ GTCCCAAA position 694, (95.28)
+ ATACCAAA position 1426, (96.56)
+ ATCCCAAA position 1993, (100.00)
>PF10_0155;
+ TTCCCAAA position 40, (95.28)
+ ATACCAAA position 335, (96.56)
>PF14_0425;
+ ATCCAAAA position 623, (97.61)
+ ATCCAAAA position 1990, (97.61)
>PF13_0141;
+ ATCCCAAA position 1811, (100.00)
>PF14_0341;
+ ATCACAAA position 19, (95.15)
+ ATCCTAAA position 403, (95.59)
>PF14_0378;
+ ATCCAAAA position 365, (97.61)
+ AACCCAAA position 1919, (95.15)*
zoops1
PF14_0598; 1280 3.56e-09 TACTTATGTT CGCACACATGC AAATTAAAAA*
PFI1105w; 516 4.75e-09 TATTTAAATA CTGACGCATGC ATAAGGAGTA*
PF10_0155; 1380 1.57e-07 GGCCCCATTT CTGATCCATGC TAAAATACCA
PF14_0341; 616 4.40e-07 ATTATATAGA GGGATACCTGT GAAAATATAT
PFI0755c; 286 7.42e-07 TAATATATTA TAGAGGTGTGC ACTACATACA
PF11_0208; 423 8.85e-07 TATATATTGT TGCACACATGT TATTTTTTGT
PFF1155w; 403 1.40e-06 TTAATTTTTA GTCAGGTATGT AAAATTTTTG*
PFF1300w; 1145 2.18e-06 TATATATAAA CCTATGCGTGT ATATTATATA*
PF13_0141; 1075 3.13e-06 ATTTTTATAA GGATGCTATGC GAAATTATTA*
PF14_0378; 183 5.01e-06 ACTTTGTAAG TGTAGATATGC TTTTCTACAT*
32

## Slide 33
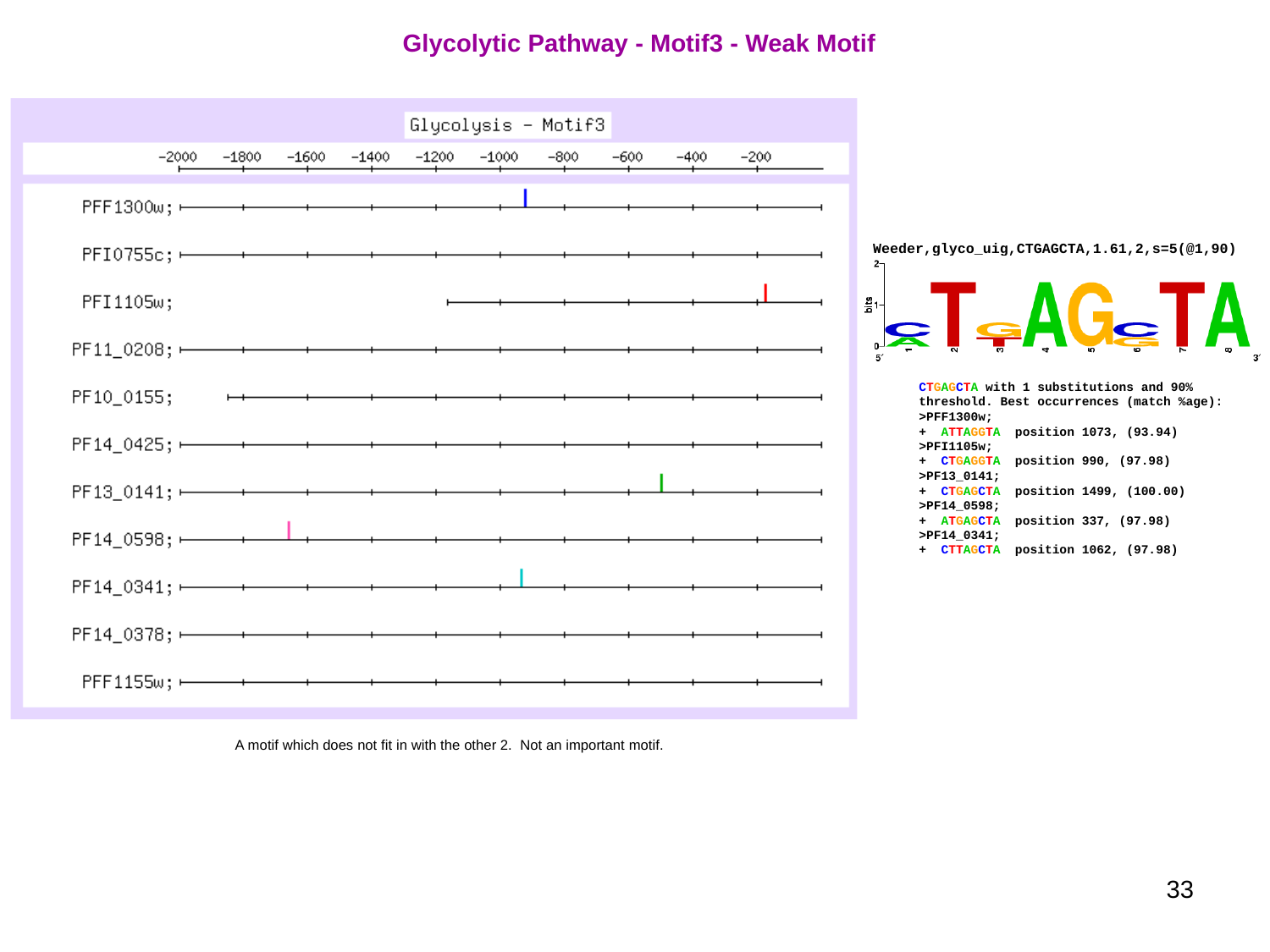

Glycolytic Pathway - Motif3 - Weak Motif
Weeder,glyco_uig,CTGAGCTA,1.61,2,s=5(@1,90)
CTGAGCTA with 1 substitutions and 90%
threshold. Best occurrences (match %age):
>PFF1300w;
+ ATTAGGTA position 1073, (93.94)
>PFI1105w;
+ CTGAGGTA position 990, (97.98)
>PF13_0141;
+ CTGAGCTA position 1499, (100.00)
>PF14_0598;
+ ATGAGCTA position 337, (97.98)
>PF14_0341;
+ CTTAGCTA position 1062, (97.98)
A motif which does not fit in with the other 2. Not an important motif.
33

## Slide 34
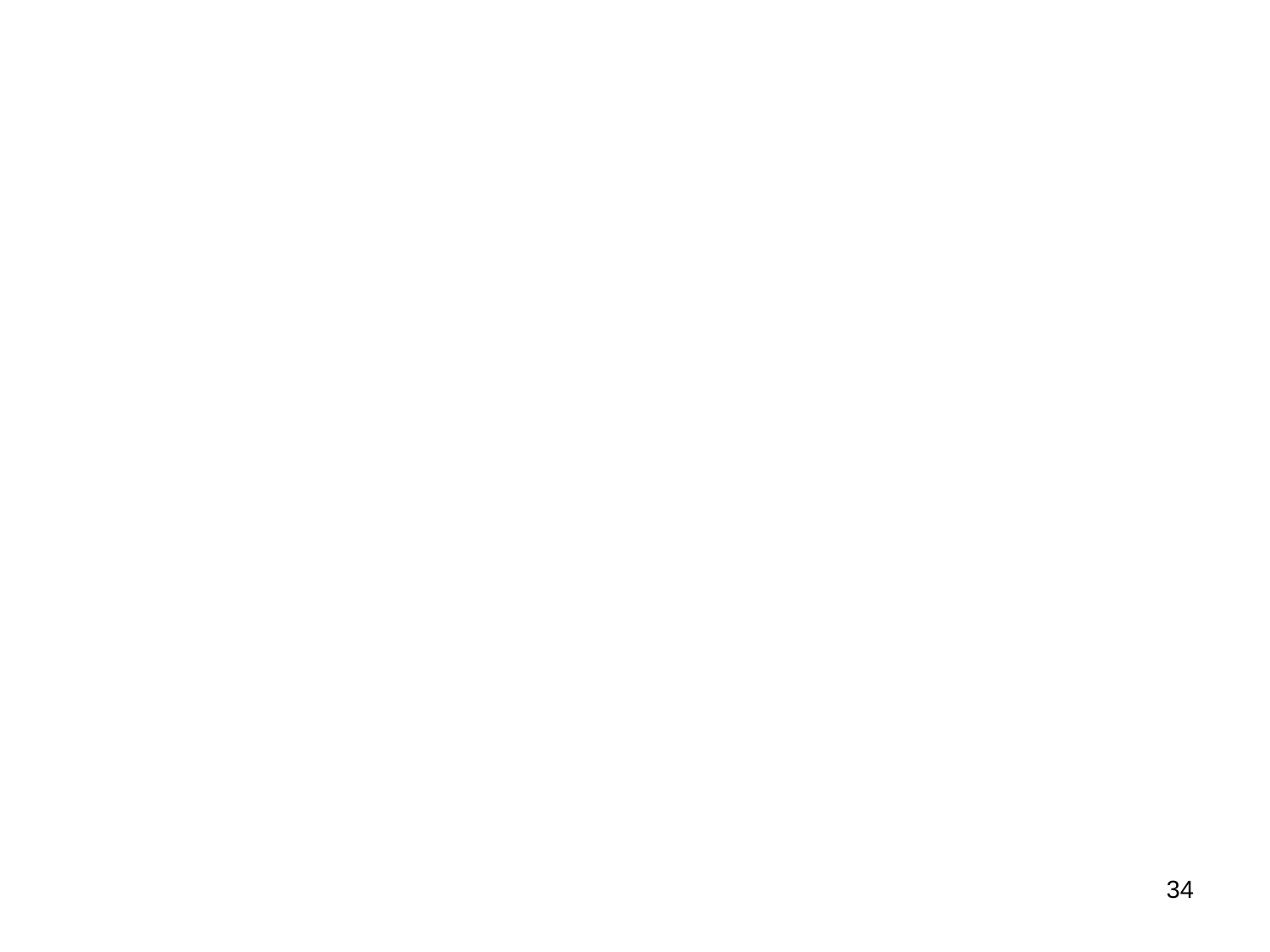

34

## Slide 35
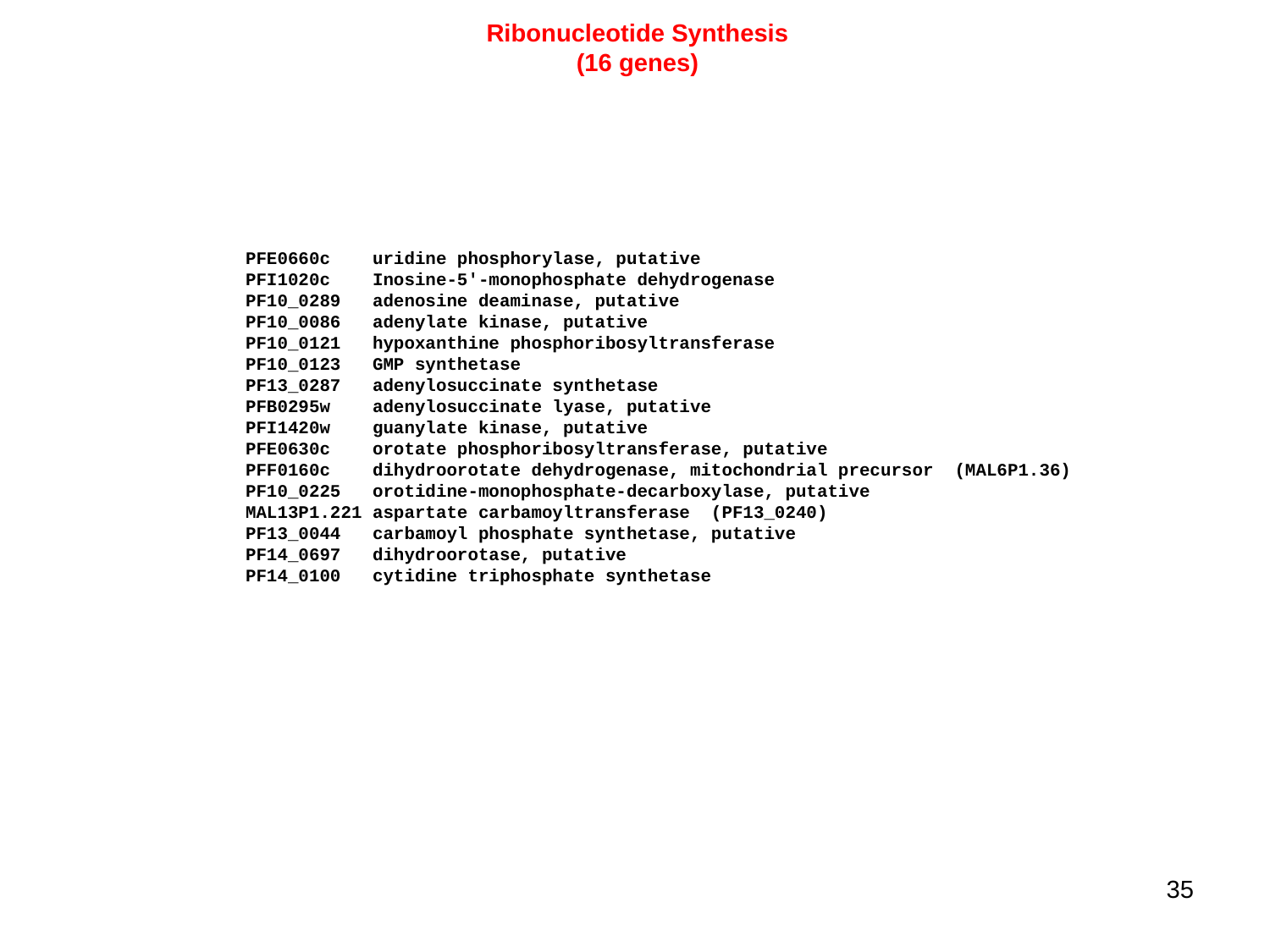

Ribonucleotide Synthesis
(16 genes)
PFE0660c 	uridine phosphorylase, putative
PFI1020c 	Inosine-5'-monophosphate dehydrogenase
PF10_0289 	adenosine deaminase, putative
PF10_0086 	adenylate kinase, putative
PF10_0121 	hypoxanthine phosphoribosyltransferase
PF10_0123 	GMP synthetase
PF13_0287 	adenylosuccinate synthetase
PFB0295w 	adenylosuccinate lyase, putative
PFI1420w 	guanylate kinase, putative
PFE0630c 	orotate phosphoribosyltransferase, putative
PFF0160c 	dihydroorotate dehydrogenase, mitochondrial precursor (MAL6P1.36)
PF10_0225 	orotidine-monophosphate-decarboxylase, putative
MAL13P1.221 aspartate carbamoyltransferase (PF13_0240)
PF13_0044 	carbamoyl phosphate synthetase, putative
PF14_0697 	dihydroorotase, putative
PF14_0100 	cytidine triphosphate synthetase
35

## Slide 36
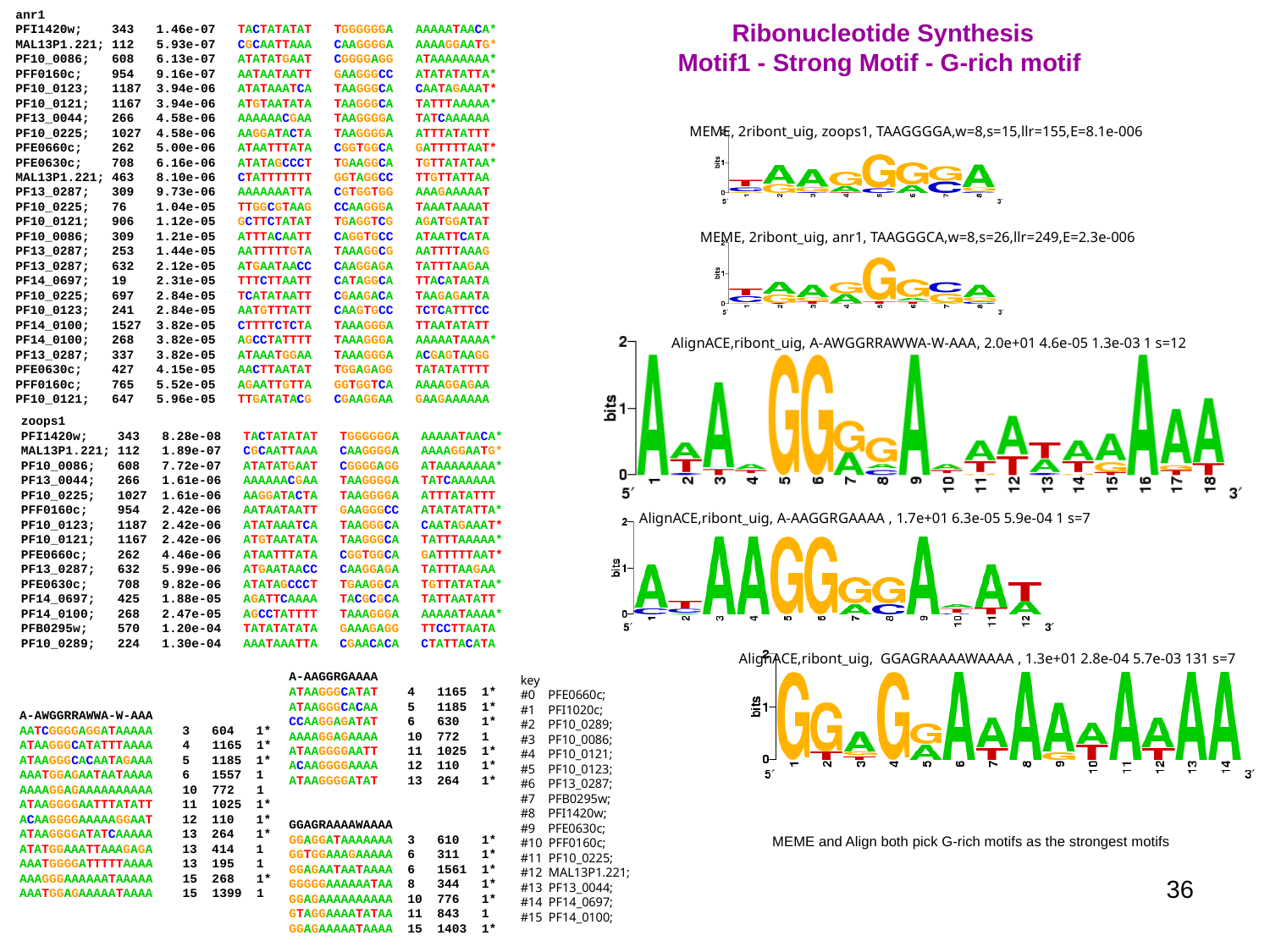

anr1
PFI1420w; 343 1.46e-07 TACTATATAT TGGGGGGA AAAAATAACA*
MAL13P1.221; 112 5.93e-07 CGCAATTAAA CAAGGGGA AAAAGGAATG*
PF10_0086; 608 6.13e-07 ATATATGAAT CGGGGAGG ATAAAAAAAA*
PFF0160c; 954 9.16e-07 AATAATAATT GAAGGGCC ATATATATTA*
PF10_0123; 1187 3.94e-06 ATATAAATCA TAAGGGCA CAATAGAAAT*
PF10_0121; 1167 3.94e-06 ATGTAATATA TAAGGGCA TATTTAAAAA*
PF13_0044; 266 4.58e-06 AAAAAACGAA TAAGGGGA TATCAAAAAA
PF10_0225; 1027 4.58e-06 AAGGATACTA TAAGGGGA ATTTATATTT
PFE0660c; 262 5.00e-06 ATAATTTATA CGGTGGCA GATTTTTAAT*
PFE0630c; 708 6.16e-06 ATATAGCCCT TGAAGGCA TGTTATATAA*
MAL13P1.221; 463 8.10e-06 CTATTTTTTT GGTAGGCC TTGTTATTAA
PF13_0287; 309 9.73e-06 AAAAAAATTA CGTGGTGG AAAGAAAAAT
PF10_0225; 76 1.04e-05 TTGGCGTAAG CCAAGGGA TAAATAAAAT
PF10_0121; 906 1.12e-05 GCTTCTATAT TGAGGTCG AGATGGATAT
PF10_0086; 309 1.21e-05 ATTTACAATT CAGGTGCC ATAATTCATA
PF13_0287; 253 1.44e-05 AATTTTTGTA TAAAGGCG AATTTTAAAG
PF13_0287; 632 2.12e-05 ATGAATAACC CAAGGAGA TATTTAAGAA
PF14_0697; 19 2.31e-05 TTTCTTAATT CATAGGCA TTACATAATA
PF10_0225; 697 2.84e-05 TCATATAATT CGAAGACA TAAGAGAATA
PF10_0123; 241 2.84e-05 AATGTTTATT CAAGTGCC TCTCATTTCC
PF14_0100; 1527 3.82e-05 CTTTTCTCTA TAAAGGGA TTAATATATT
PF14_0100; 268 3.82e-05 AGCCTATTTT TAAAGGGA AAAAATAAAA*
PF13_0287; 337 3.82e-05 ATAAATGGAA TAAAGGGA ACGAGTAAGG
PFE0630c; 427 4.15e-05 AACTTAATAT TGGAGAGG TATATATTTT
PFF0160c; 765 5.52e-05 AGAATTGTTA GGTGGTCA AAAAGGAGAA
PF10_0121; 647 5.96e-05 TTGATATACG CGAAGGAA GAAGAAAAAA
Ribonucleotide Synthesis
Motif1 - Strong Motif - G-rich motif
MEME, 2ribont_uig, zoops1, TAAGGGGA,w=8,s=15,llr=155,E=8.1e-006
MEME, 2ribont_uig, anr1, TAAGGGCA,w=8,s=26,llr=249,E=2.3e-006
AlignACE,ribont_uig, A-AWGGRRAWWA-W-AAA, 2.0e+01 4.6e-05 1.3e-03 1 s=12
AlignACE,ribont_uig, A-AAGGRGAAAA , 1.7e+01 6.3e-05 5.9e-04 1 s=7
AlignACE,ribont_uig, GGAGRAAAAWAAAA , 1.3e+01 2.8e-04 5.7e-03 131 s=7
zoops1
PFI1420w; 343 8.28e-08 TACTATATAT TGGGGGGA AAAAATAACA*
MAL13P1.221; 112 1.89e-07 CGCAATTAAA CAAGGGGA AAAAGGAATG*
PF10_0086; 608 7.72e-07 ATATATGAAT CGGGGAGG ATAAAAAAAA*
PF13_0044; 266 1.61e-06 AAAAAACGAA TAAGGGGA TATCAAAAAA
PF10_0225; 1027 1.61e-06 AAGGATACTA TAAGGGGA ATTTATATTT
PFF0160c; 954 2.42e-06 AATAATAATT GAAGGGCC ATATATATTA*
PF10_0123; 1187 2.42e-06 ATATAAATCA TAAGGGCA CAATAGAAAT*
PF10_0121; 1167 2.42e-06 ATGTAATATA TAAGGGCA TATTTAAAAA*
PFE0660c; 262 4.46e-06 ATAATTTATA CGGTGGCA GATTTTTAAT*
PF13_0287; 632 5.99e-06 ATGAATAACC CAAGGAGA TATTTAAGAA
PFE0630c; 708 9.82e-06 ATATAGCCCT TGAAGGCA TGTTATATAA*
PF14_0697; 425 1.88e-05 AGATTCAAAA TACGCGCA TATTAATATT
PF14_0100; 268 2.47e-05 AGCCTATTTT TAAAGGGA AAAAATAAAA*
PFB0295w; 570 1.20e-04 TATATATATA GAAAGAGG TTCCTTAATA
PF10_0289; 224 1.30e-04 AAATAAATTA CGAACACA CTATTACATA
A-AAGGRGAAAA
ATAAGGGCATAT 4 1165 1*
ATAAGGGCACAA 5 1185 1*
CCAAGGAGATAT 6 630 1*
AAAAGGAGAAAA 10 772 1
ATAAGGGGAATT 11 1025 1*
ACAAGGGGAAAA 12 110 1*
ATAAGGGGATAT 13 264 1*
key
#0 PFE0660c;
#1 PFI1020c;
#2 PF10_0289;
#3 PF10_0086;
#4 PF10_0121;
#5 PF10_0123;
#6 PF13_0287;
#7 PFB0295w;
#8 PFI1420w;
#9 PFE0630c;
#10 PFF0160c;
#11 PF10_0225;
#12 MAL13P1.221;
#13 PF13_0044;
#14 PF14_0697;
#15 PF14_0100;
A-AWGGRRAWWA-W-AAA
AATCGGGGAGGATAAAAA 3 604 1*
ATAAGGGCATATTTAAAA 4 1165 1*
ATAAGGGCACAATAGAAA 5 1185 1*
AAATGGAGAATAATAAAA 6 1557 1
AAAAGGAGAAAAAAAAAA 10 772 1
ATAAGGGGAATTTATATT 11 1025 1*
ACAAGGGGAAAAAGGAAT 12 110 1*
ATAAGGGGATATCAAAAA 13 264 1*
ATATGGAAATTAAAGAGA 13 414 1
AAATGGGGATTTTTAAAA 13 195 1
AAAGGGAAAAAATAAAAA 15 268 1*
AAATGGAGAAAAATAAAA 15 1399 1
GGAGRAAAAWAAAA
GGAGGATAAAAAAA 3 610 1*
GGTGGAAAGAAAAA 6 311 1*
GGAGAATAATAAAA 6 1561 1*
GGGGGAAAAAATAA 8 344 1*
GGAGAAAAAAAAAA 10 776 1*
GTAGGAAAATATAA 11 843 1
GGAGAAAAATAAAA 15 1403 1*
MEME and Align both pick G-rich motifs as the strongest motifs
36

## Slide 37
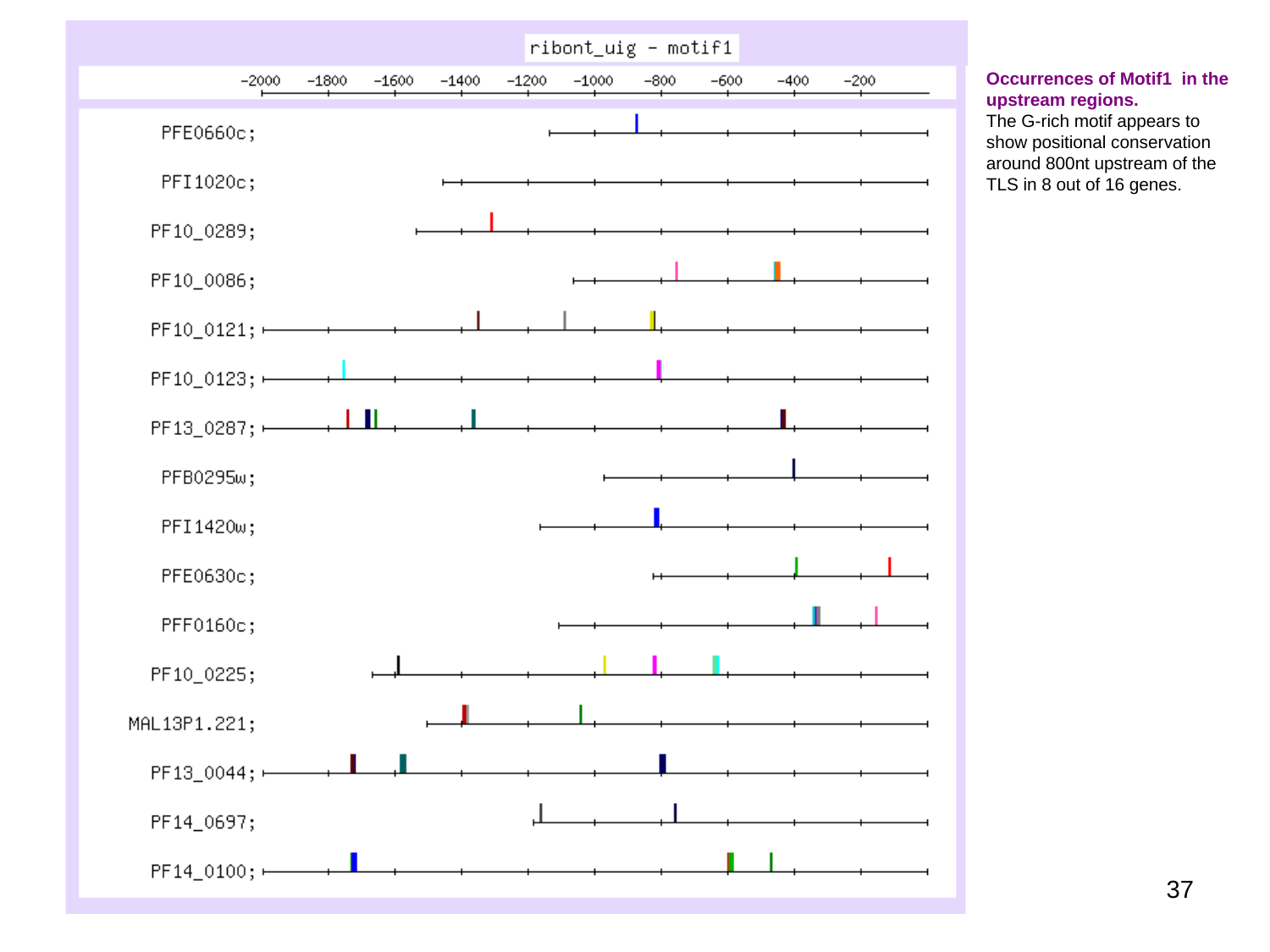

Occurrences of Motif1 in the
upstream regions.
The G-rich motif appears to
show positional conservation
around 800nt upstream of the
TLS in 8 out of 16 genes.
37

## Slide 38
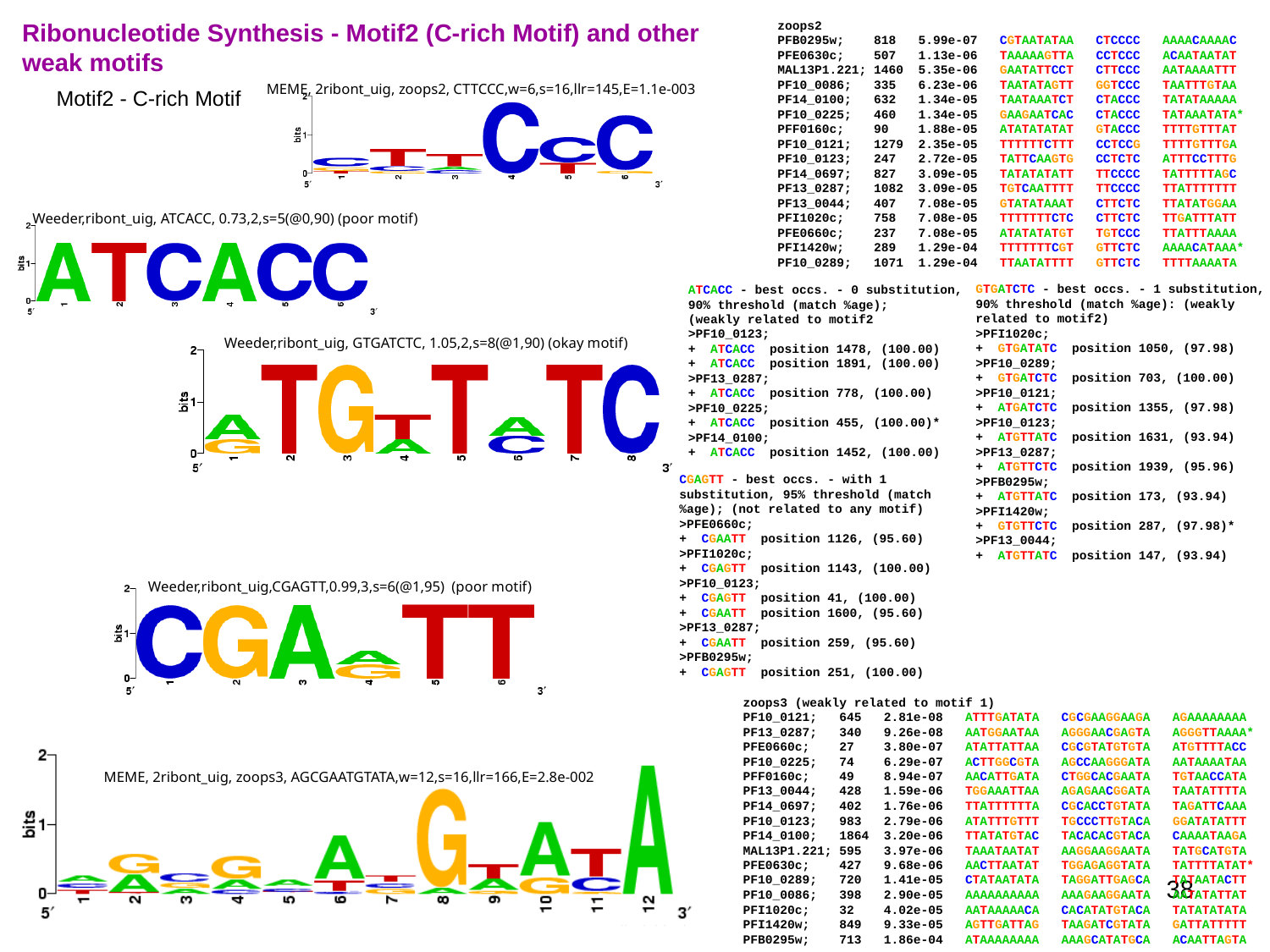

Ribonucleotide Synthesis - Motif2 (C-rich Motif) and other
weak motifs
zoops2
PFB0295w; 818 5.99e-07 CGTAATATAA CTCCCC AAAACAAAAC
PFE0630c; 507 1.13e-06 TAAAAAGTTA CCTCCC ACAATAATAT
MAL13P1.221; 1460 5.35e-06 GAATATTCCT CTTCCC AATAAAATTT
PF10_0086; 335 6.23e-06 TAATATAGTT GGTCCC TAATTTGTAA
PF14_0100; 632 1.34e-05 TAATAAATCT CTACCC TATATAAAAA
PF10_0225; 460 1.34e-05 GAAGAATCAC CTACCC TATAAATATA*
PFF0160c; 90 1.88e-05 ATATATATAT GTACCC TTTTGTTTAT
PF10_0121; 1279 2.35e-05 TTTTTTCTTT CCTCCG TTTTGTTTGA
PF10_0123; 247 2.72e-05 TATTCAAGTG CCTCTC ATTTCCTTTG
PF14_0697; 827 3.09e-05 TATATATATT TTCCCC TATTTTTAGC
PF13_0287; 1082 3.09e-05 TGTCAATTTT TTCCCC TTATTTTTTT
PF13_0044; 407 7.08e-05 GTATATAAAT CTTCTC TTATATGGAA
PFI1020c; 758 7.08e-05 TTTTTTTCTC CTTCTC TTGATTTATT
PFE0660c; 237 7.08e-05 ATATATATGT TGTCCC TTATTTAAAA
PFI1420w; 289 1.29e-04 TTTTTTTCGT GTTCTC AAAACATAAA*
PF10_0289; 1071 1.29e-04 TTAATATTTT GTTCTC TTTTAAAATA
MEME, 2ribont_uig, zoops2, CTTCCC,w=6,s=16,llr=145,E=1.1e-003
Motif2 - C-rich Motif
Weeder,ribont_uig, ATCACC, 0.73,2,s=5(@0,90) (poor motif)
GTGATCTC - best occs. - 1 substitution, 90% threshold (match %age): (weakly related to motif2)
>PFI1020c;
+ GTGATATC position 1050, (97.98)
>PF10_0289;
+ GTGATCTC position 703, (100.00)
>PF10_0121;
+ ATGATCTC position 1355, (97.98)
>PF10_0123;
+ ATGTTATC position 1631, (93.94)
>PF13_0287;
+ ATGTTCTC position 1939, (95.96)
>PFB0295w;
+ ATGTTATC position 173, (93.94)
>PFI1420w;
+ GTGTTCTC position 287, (97.98)*
>PF13_0044;
+ ATGTTATC position 147, (93.94)
ATCACC - best occs. - 0 substitution,
90% threshold (match %age);
(weakly related to motif2
>PF10_0123;
+ ATCACC position 1478, (100.00)
+ ATCACC position 1891, (100.00)
>PF13_0287;
+ ATCACC position 778, (100.00)
>PF10_0225;
+ ATCACC position 455, (100.00)*
>PF14_0100;
+ ATCACC position 1452, (100.00)
Weeder,ribont_uig, GTGATCTC, 1.05,2,s=8(@1,90) (okay motif)
CGAGTT - best occs. - with 1 substitution, 95% threshold (match
%age); (not related to any motif)
>PFE0660c;
+ CGAATT position 1126, (95.60)
>PFI1020c;
+ CGAGTT position 1143, (100.00)
>PF10_0123;
+ CGAGTT position 41, (100.00)
+ CGAATT position 1600, (95.60)
>PF13_0287;
+ CGAATT position 259, (95.60)
>PFB0295w;
+ CGAGTT position 251, (100.00)
Weeder,ribont_uig,CGAGTT,0.99,3,s=6(@1,95) (poor motif)
zoops3 (weakly related to motif 1)
PF10_0121; 645 2.81e-08 ATTTGATATA CGCGAAGGAAGA AGAAAAAAAA
PF13_0287; 340 9.26e-08 AATGGAATAA AGGGAACGAGTA AGGGTTAAAA*
PFE0660c; 27 3.80e-07 ATATTATTAA CGCGTATGTGTA ATGTTTTACC
PF10_0225; 74 6.29e-07 ACTTGGCGTA AGCCAAGGGATA AATAAAATAA
PFF0160c; 49 8.94e-07 AACATTGATA CTGGCACGAATA TGTAACCATA
PF13_0044; 428 1.59e-06 TGGAAATTAA AGAGAACGGATA TAATATTTTA
PF14_0697; 402 1.76e-06 TTATTTTTTA CGCACCTGTATA TAGATTCAAA
PF10_0123; 983 2.79e-06 ATATTTGTTT TGCCCTTGTACA GGATATATTT
PF14_0100; 1864 3.20e-06 TTATATGTAC TACACACGTACA CAAAATAAGA
MAL13P1.221; 595 3.97e-06 TAAATAATAT AAGGAAGGAATA TATGCATGTA
PFE0630c; 427 9.68e-06 AACTTAATAT TGGAGAGGTATA TATTTTATAT*
PF10_0289; 720 1.41e-05 CTATAATATA TAGGATTGAGCA TATAATACTT
PF10_0086; 398 2.90e-05 AAAAAAAAAA AAAGAAGGAATA AATATATTAT
PFI1020c; 32 4.02e-05 AATAAAAACA CACATATGTACA TATATATATA
PFI1420w; 849 9.33e-05 AGTTGATTAG TAAGATCGTATA GATTATTTTT
PFB0295w; 713 1.86e-04 ATAAAAAAAA AAAGCATATGCA ACAATTAGTA
MEME, 2ribont_uig, zoops3, AGCGAATGTATA,w=12,s=16,llr=166,E=2.8e-002
38

## Slide 39
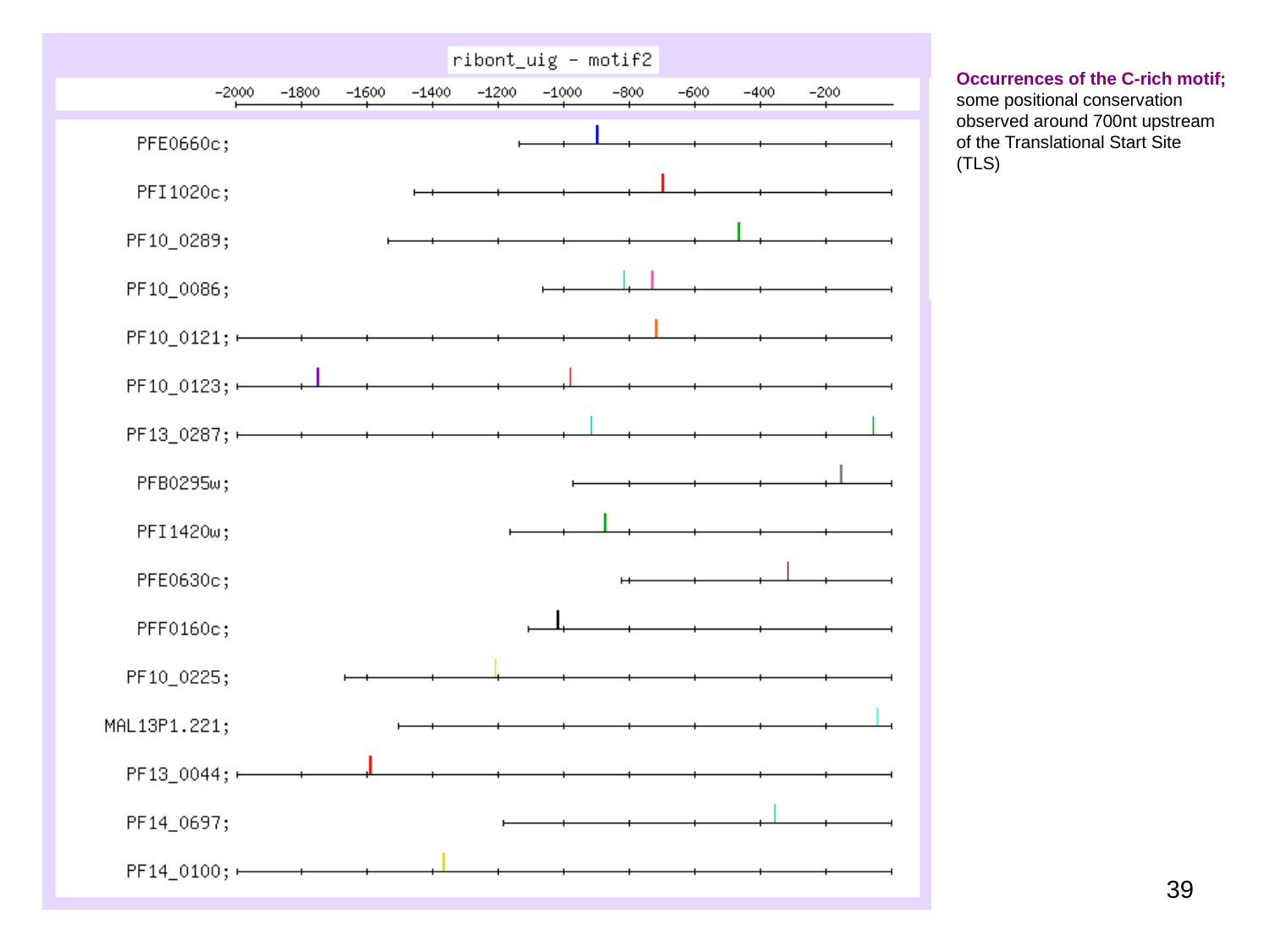

Occurrences of the C-rich motif;
some positional conservation
observed around 700nt upstream
of the Translational Start Site
(TLS)
39

## Slide 40
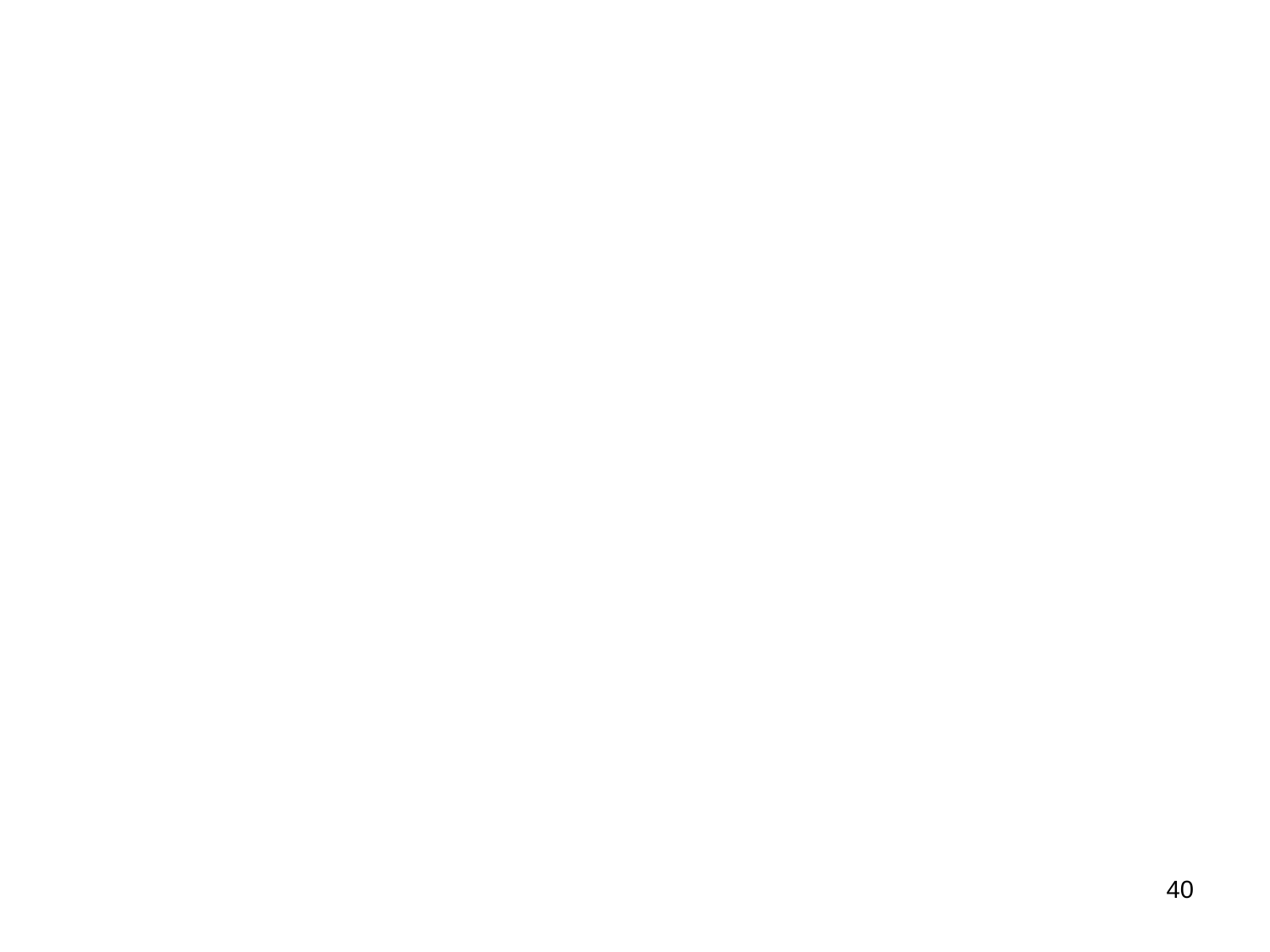

40
