# Supplementary material for: Identification of putative regulatory motifs in the upstream regions of co-expressed functional groups of genes in Plasmodium falciparum
Source: BMC Genomics. 2009 Jan 13;10:18. doi: 10.1186/1471-2164-10-18 (PMC2662883; doi:10.1186/1471-2164-10-18)
Supplement: Additional file 2 — Over-represented upstream motifs identified for the 6 functional groups of genes expressed during the trophozoite to early schizont transition. Strong and weak motif groups identified for each functional group are given. [file 1471-2164-10-18-S2.ppt]

## Slide 1
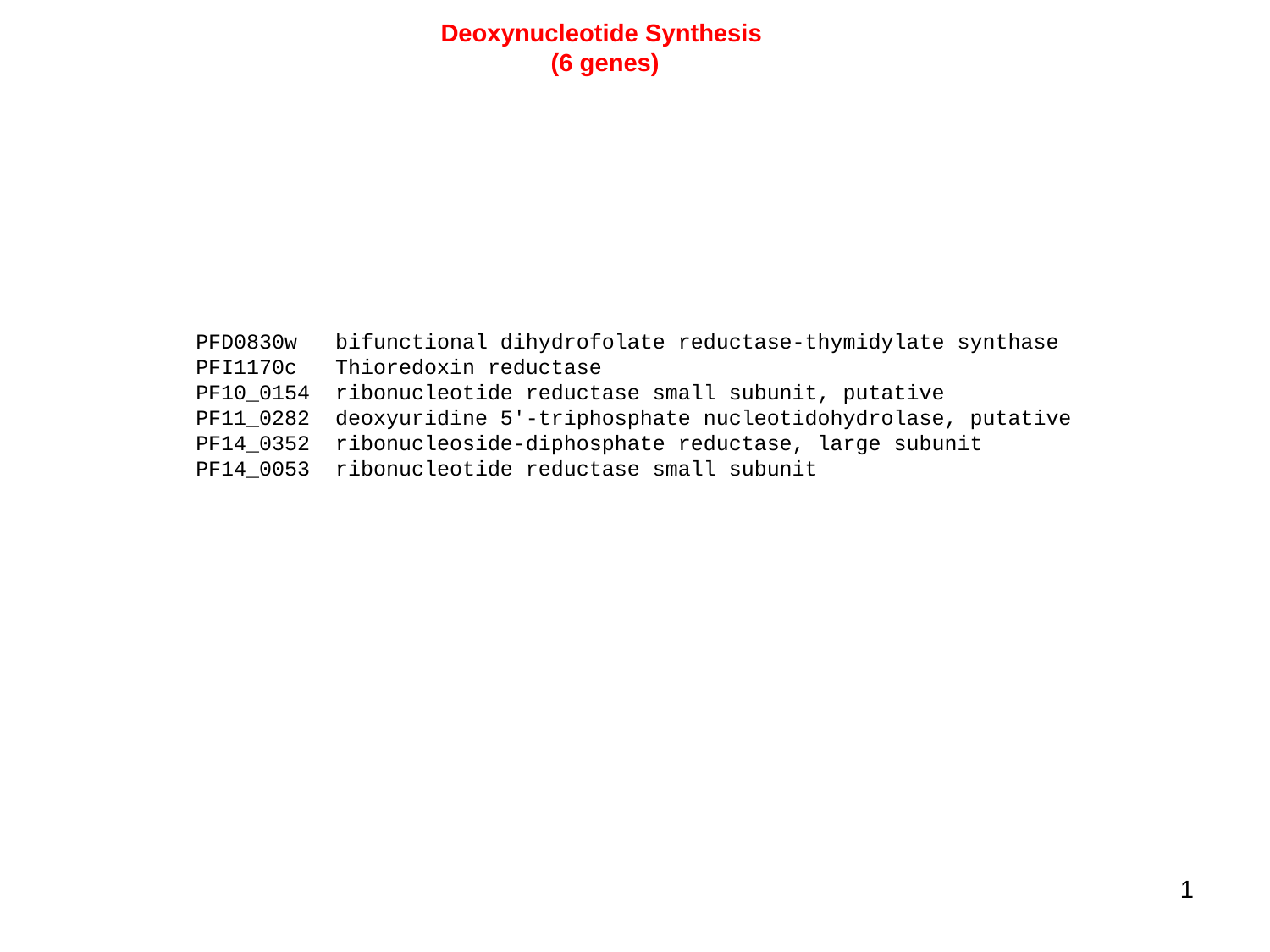

Deoxynucleotide Synthesis
(6 genes)
PFD0830w bifunctional dihydrofolate reductase-thymidylate synthase
PFI1170c Thioredoxin reductase
PF10_0154 ribonucleotide reductase small subunit, putative
PF11_0282 deoxyuridine 5'-triphosphate nucleotidohydrolase, putative
PF14_0352 ribonucleoside-diphosphate reductase, large subunit
PF14_0053 ribonucleotide reductase small subunit
1

## Slide 2
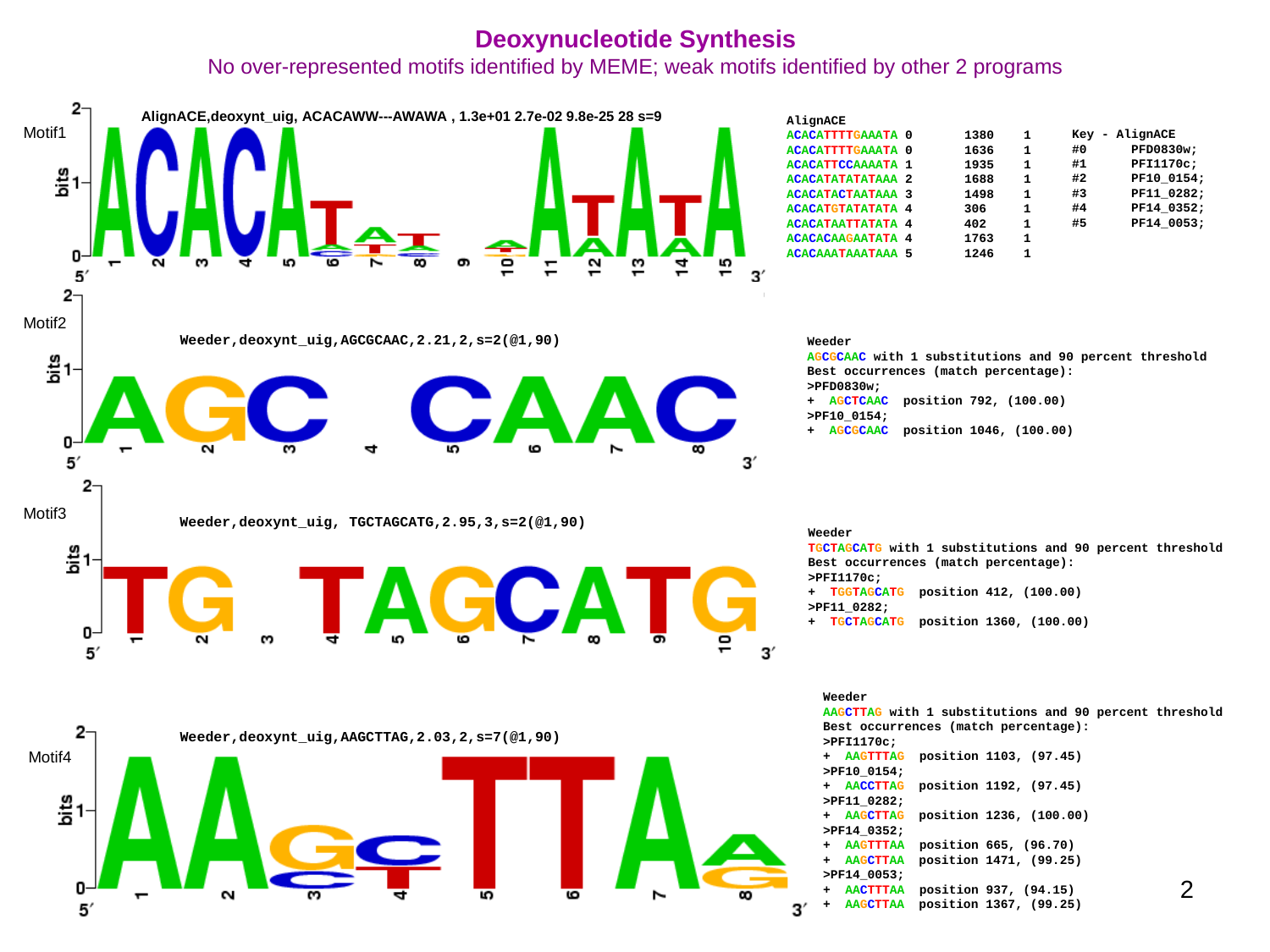

Deoxynucleotide Synthesis
No over-represented motifs identified by MEME; weak motifs identified by other 2 programs
AlignACE,deoxynt_uig, ACACAWW---AWAWA , 1.3e+01 2.7e-02 9.8e-25 28 s=9
Motif1
Motif2
Weeder,deoxynt_uig,AGCGCAAC,2.21,2,s=2(@1,90)
Motif3
Weeder,deoxynt_uig, TGCTAGCATG,2.95,3,s=2(@1,90)
Weeder,deoxynt_uig,AAGCTTAG,2.03,2,s=7(@1,90)
Motif4
AlignACE
ACACATTTTGAAATA 0 1380 1
ACACATTTTGAAATA 0 1636 1
ACACATTCCAAAATA 1 1935 1
ACACATATATATAAA 2 1688 1
ACACATACTAATAAA 3 1498 1
ACACATGTATATATA 4 306 1
ACACATAATTATATA 4 402 1
ACACACAAGAATATA 4 1763 1
ACACAAATAAATAAA 5 1246 1
Key - AlignACE
#0 PFD0830w;
#1 PFI1170c;
#2 PF10_0154;
#3 PF11_0282;
#4 PF14_0352;
#5 PF14_0053;
Weeder
AGCGCAAC with 1 substitutions and 90 percent threshold
Best occurrences (match percentage):
>PFD0830w;
+ AGCTCAAC position 792, (100.00)
>PF10_0154;
+ AGCGCAAC position 1046, (100.00)
Weeder
TGCTAGCATG with 1 substitutions and 90 percent threshold
Best occurrences (match percentage):
>PFI1170c;
+ TGGTAGCATG position 412, (100.00)
>PF11_0282;
+ TGCTAGCATG position 1360, (100.00)
Weeder
AAGCTTAG with 1 substitutions and 90 percent threshold
Best occurrences (match percentage):
>PFI1170c;
+ AAGTTTAG position 1103, (97.45)
>PF10_0154;
+ AACCTTAG position 1192, (97.45)
>PF11_0282;
+ AAGCTTAG position 1236, (100.00)
>PF14_0352;
+ AAGTTTAA position 665, (96.70)
+ AAGCTTAA position 1471, (99.25)
>PF14_0053;
+ AACTTTAA position 937, (94.15)
+ AAGCTTAA position 1367, (99.25)
2

## Slide 3
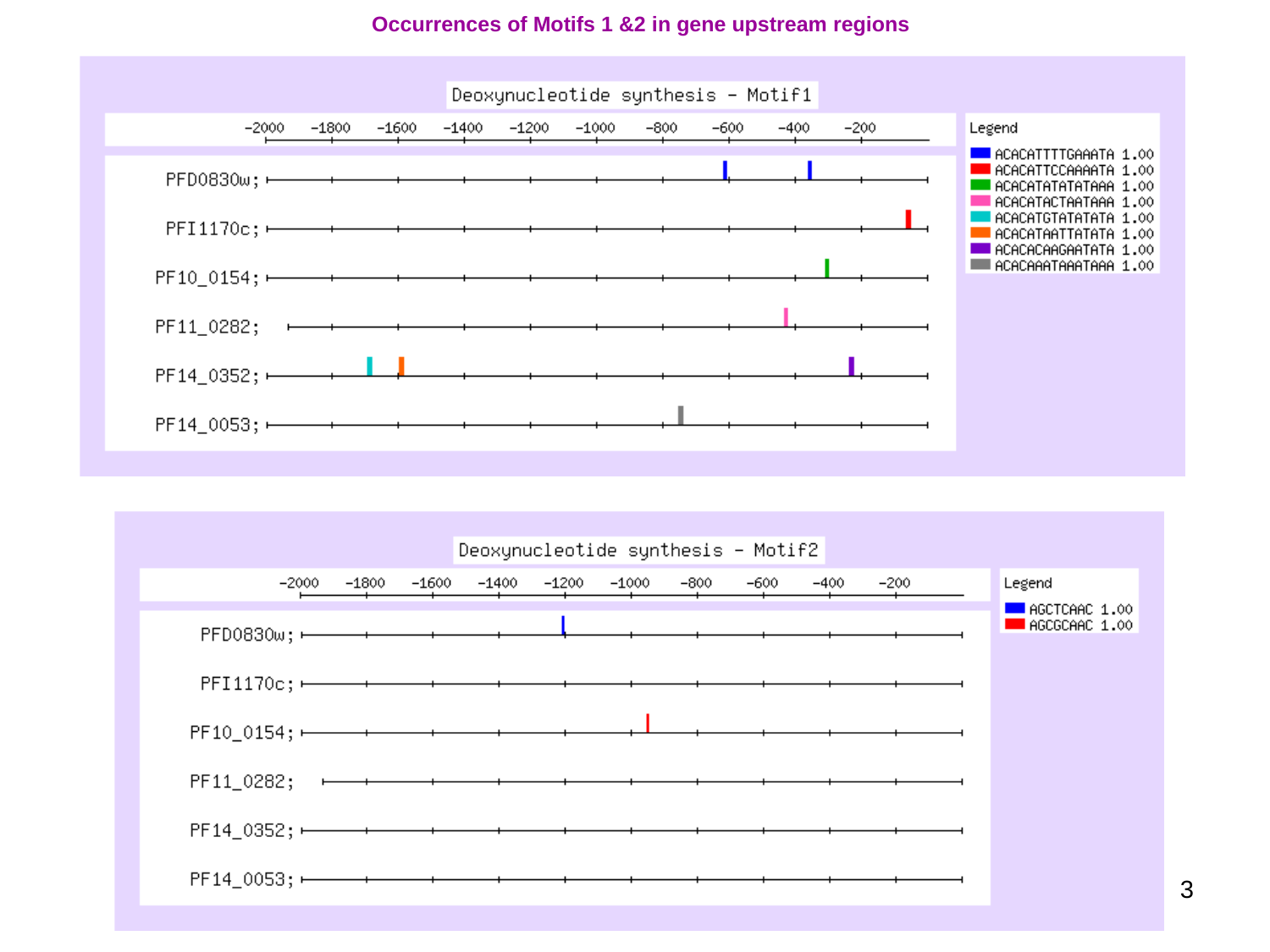

Occurrences of Motifs 1 &2 in gene upstream regions
3

## Slide 4
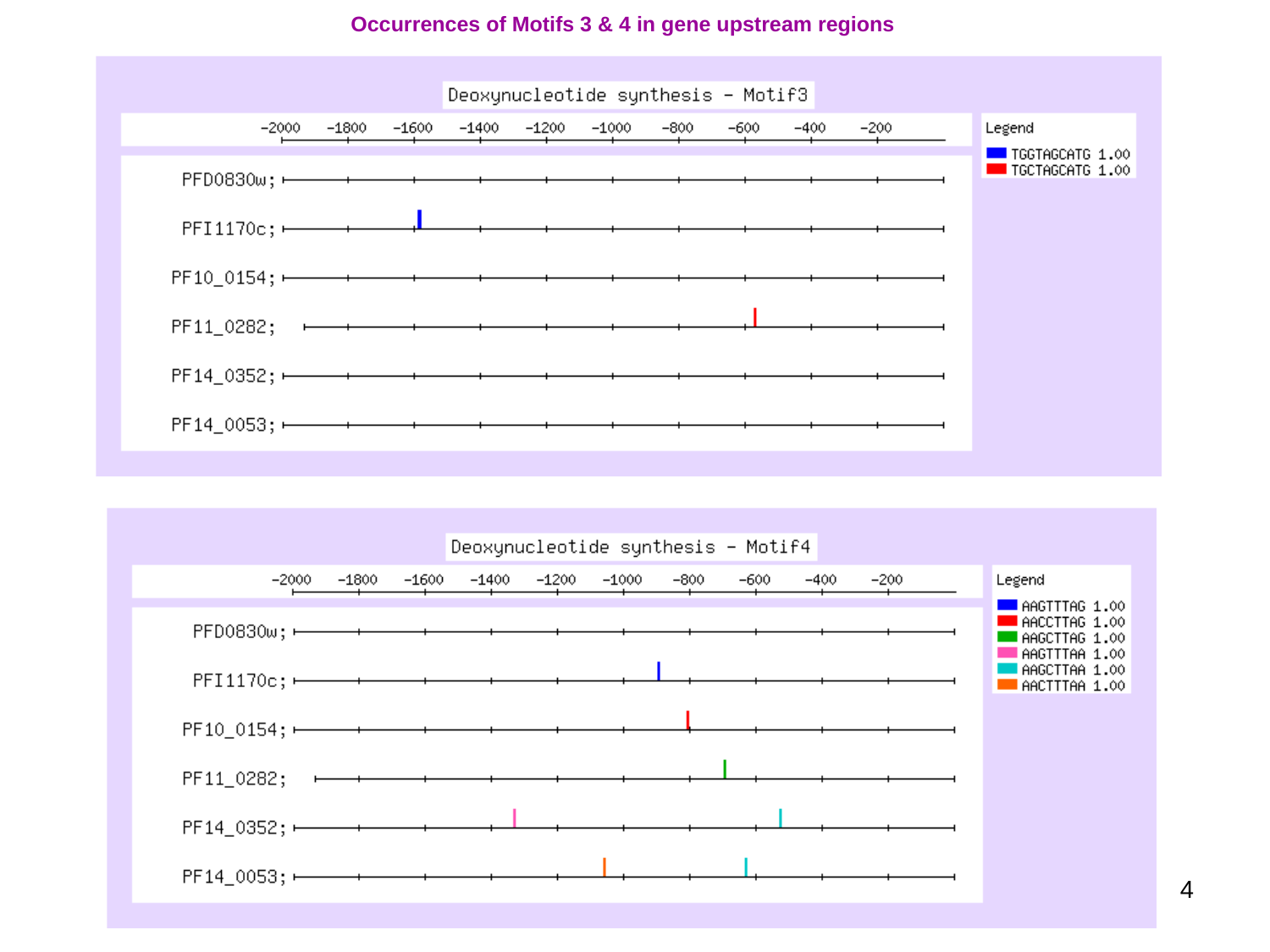

Occurrences of Motifs 3 & 4 in gene upstream regions
4

## Slide 5
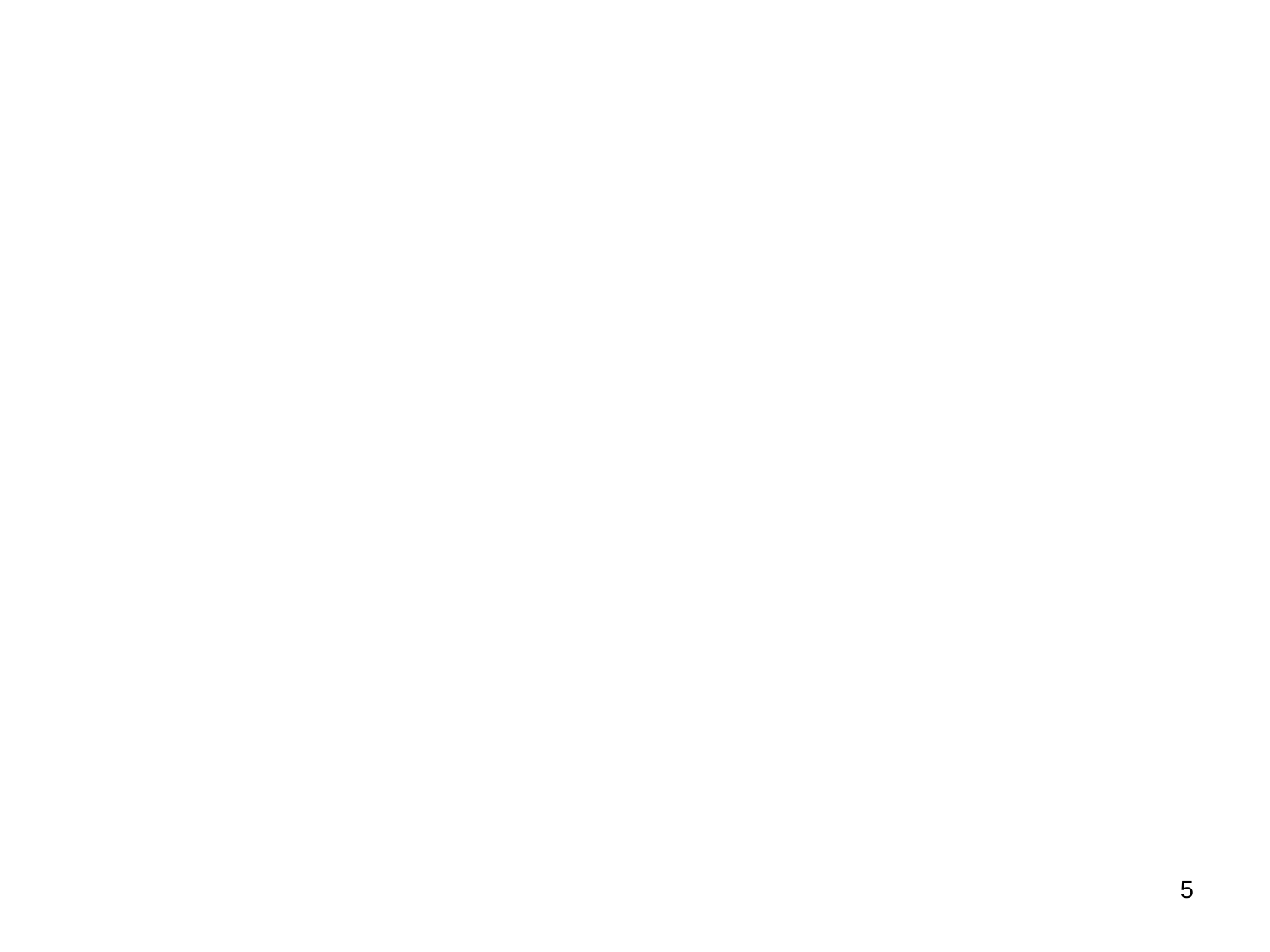

5

## Slide 6
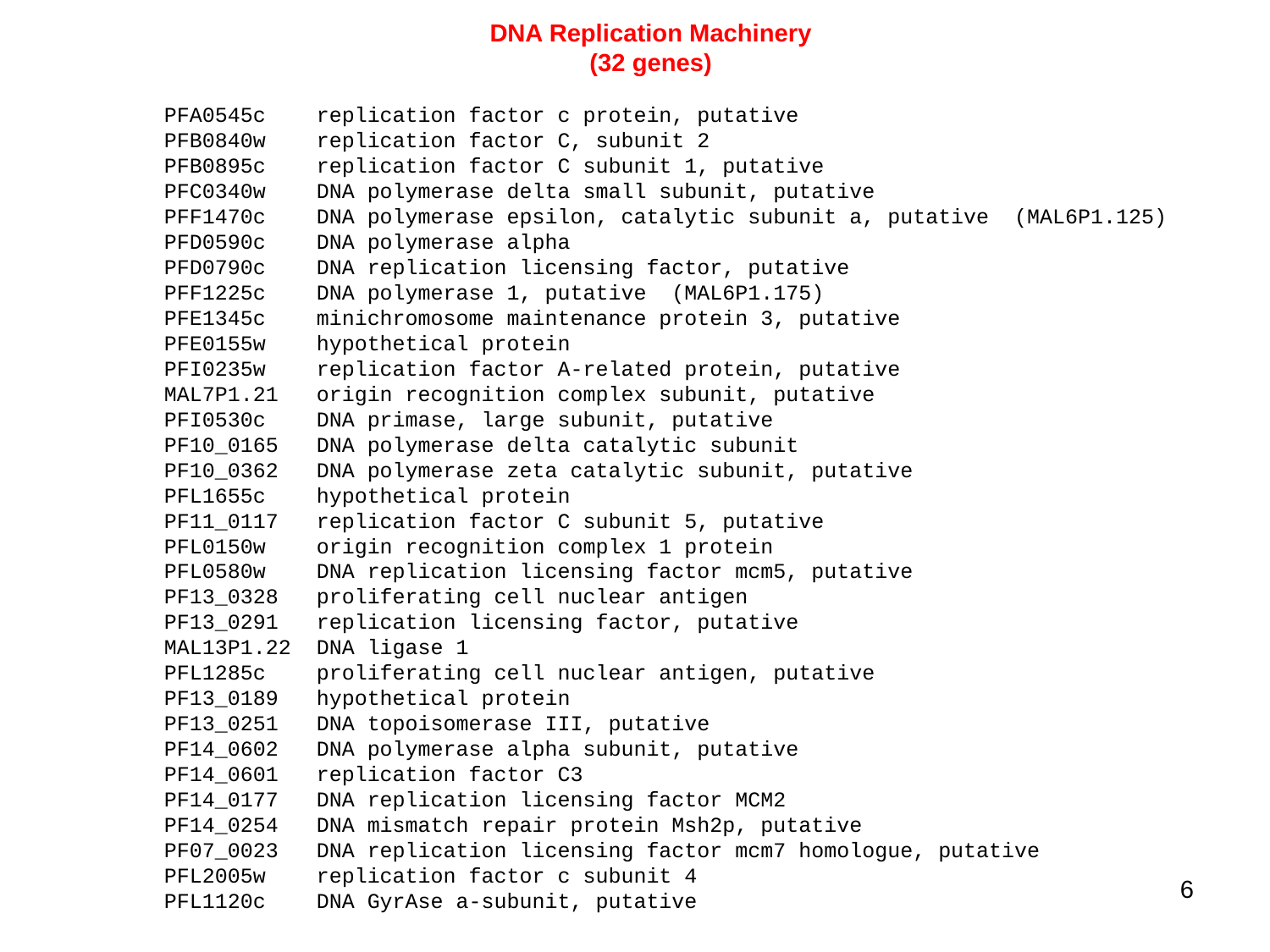

DNA Replication Machinery
(32 genes)
PFA0545c replication factor c protein, putative
PFB0840w replication factor C, subunit 2
PFB0895c replication factor C subunit 1, putative
PFC0340w DNA polymerase delta small subunit, putative
PFF1470c DNA polymerase epsilon, catalytic subunit a, putative (MAL6P1.125)
PFD0590c DNA polymerase alpha
PFD0790c DNA replication licensing factor, putative
PFF1225c DNA polymerase 1, putative (MAL6P1.175)
PFE1345c minichromosome maintenance protein 3, putative
PFE0155w hypothetical protein
PFI0235w replication factor A-related protein, putative
MAL7P1.21 origin recognition complex subunit, putative
PFI0530c DNA primase, large subunit, putative
PF10_0165 DNA polymerase delta catalytic subunit
PF10_0362 DNA polymerase zeta catalytic subunit, putative
PFL1655c hypothetical protein
PF11_0117 replication factor C subunit 5, putative
PFL0150w origin recognition complex 1 protein
PFL0580w DNA replication licensing factor mcm5, putative
PF13_0328 proliferating cell nuclear antigen
PF13_0291 replication licensing factor, putative
MAL13P1.22 DNA ligase 1
PFL1285c proliferating cell nuclear antigen, putative
PF13_0189 hypothetical protein
PF13_0251 DNA topoisomerase III, putative
PF14_0602 DNA polymerase alpha subunit, putative
PF14_0601 replication factor C3
PF14_0177 DNA replication licensing factor MCM2
PF14_0254 DNA mismatch repair protein Msh2p, putative
PF07_0023 DNA replication licensing factor mcm7 homologue, putative
PFL2005w replication factor c subunit 4
PFL1120c DNA GyrAse a-subunit, putative
6

## Slide 7
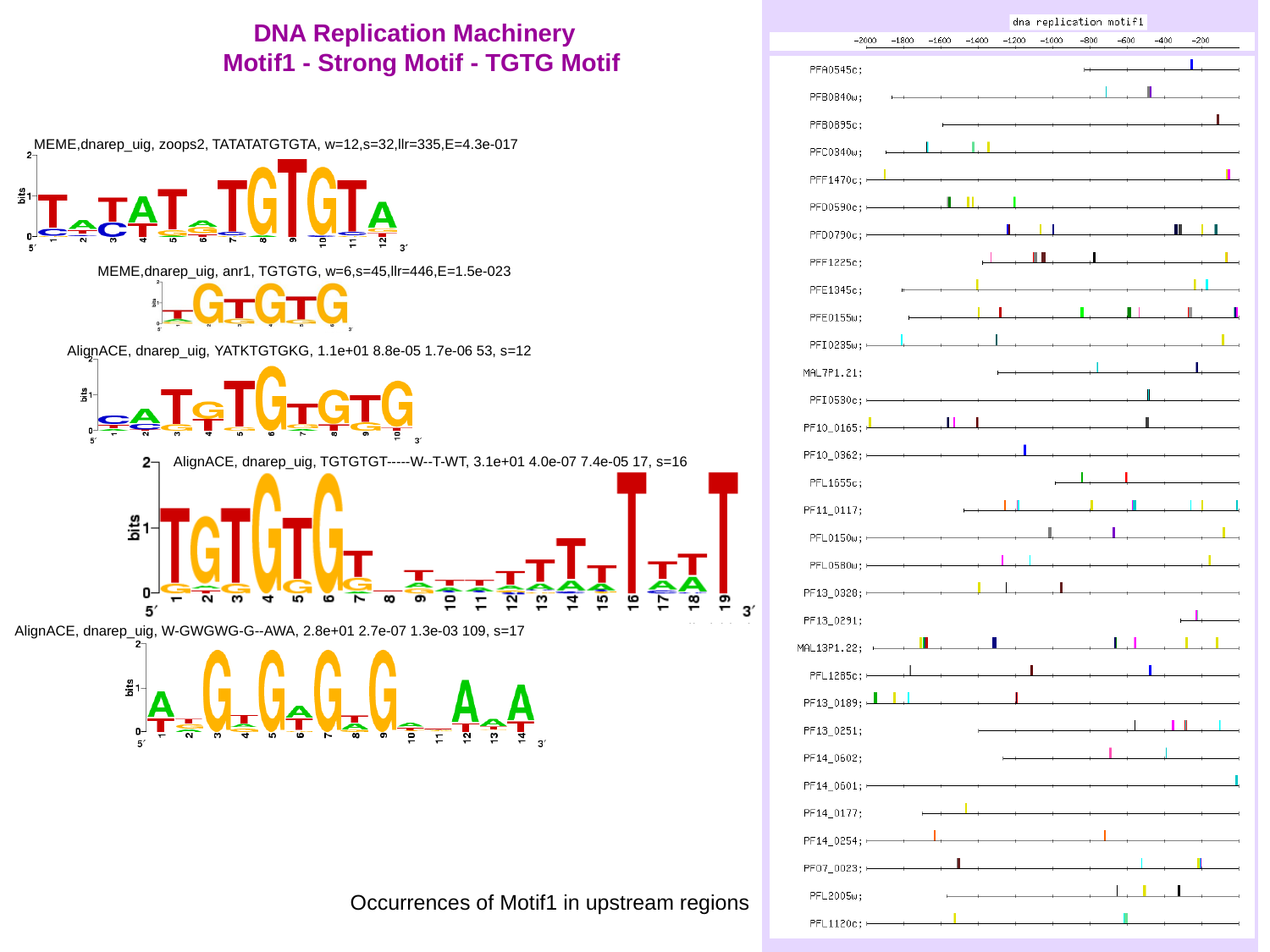

DNA Replication Machinery
Motif1 - Strong Motif - TGTG Motif
MEME,dnarep_uig, zoops2, TATATATGTGTA, w=12,s=32,llr=335,E=4.3e-017
MEME,dnarep_uig, anr1, TGTGTG, w=6,s=45,llr=446,E=1.5e-023
AlignACE, dnarep_uig, YATKTGTGKG, 1.1e+01 8.8e-05 1.7e-06 53, s=12
AlignACE, dnarep_uig, TGTGTGT-----W--T-WT, 3.1e+01 4.0e-07 7.4e-05 17, s=16
AlignACE, dnarep_uig, W-GWGWG-G--AWA, 2.8e+01 2.7e-07 1.3e-03 109, s=17
7
Occurrences of Motif1 in upstream regions

## Slide 8
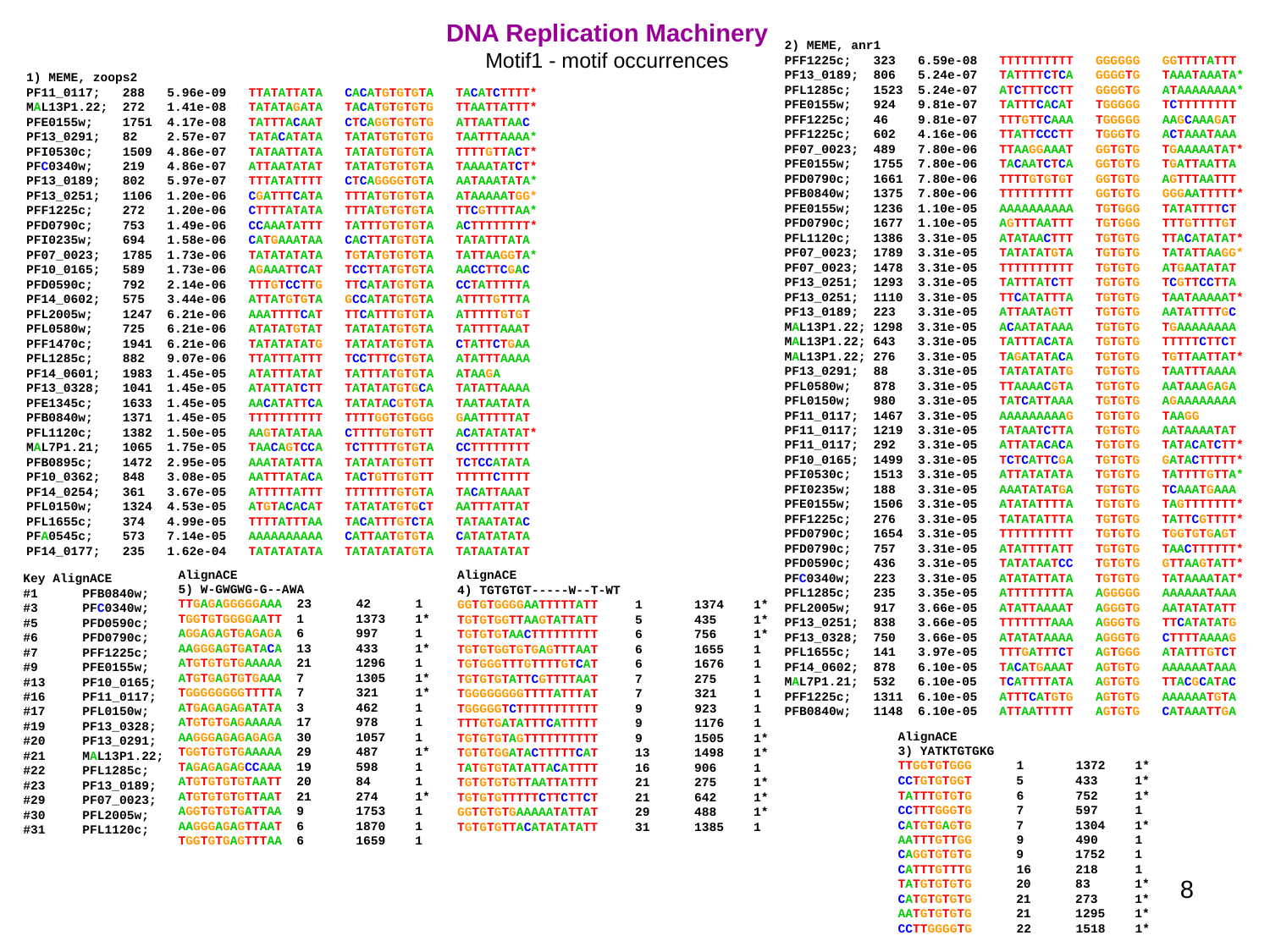

DNA Replication Machinery
Motif1 - motif occurrences
2) MEME, anr1
PFF1225c; 323 6.59e-08 TTTTTTTTTT GGGGGG GGTTTTATTT
PF13_0189; 806 5.24e-07 TATTTTCTCA GGGGTG TAAATAAATA*
PFL1285c; 1523 5.24e-07 ATCTTTCCTT GGGGTG ATAAAAAAAA*
PFE0155w; 924 9.81e-07 TATTTCACAT TGGGGG TCTTTTTTTT
PFF1225c; 46 9.81e-07 TTTGTTCAAA TGGGGG AAGCAAAGAT
PFF1225c; 602 4.16e-06 TTATTCCCTT TGGGTG ACTAAATAAA
PF07_0023; 489 7.80e-06 TTAAGGAAAT GGTGTG TGAAAAATAT*
PFE0155w; 1755 7.80e-06 TACAATCTCA GGTGTG TGATTAATTA
PFD0790c; 1661 7.80e-06 TTTTGTGTGT GGTGTG AGTTTAATTT
PFB0840w; 1375 7.80e-06 TTTTTTTTTT GGTGTG GGGAATTTTT*
PFE0155w; 1236 1.10e-05 AAAAAAAAAA TGTGGG TATATTTTCT
PFD0790c; 1677 1.10e-05 AGTTTAATTT TGTGGG TTTGTTTTGT
PFL1120c; 1386 3.31e-05 ATATAACTTT TGTGTG TTACATATAT*
PF07_0023; 1789 3.31e-05 TATATATGTA TGTGTG TATATTAAGG*
PF07_0023; 1478 3.31e-05 TTTTTTTTTT TGTGTG ATGAATATAT
PF13_0251; 1293 3.31e-05 TATTTATCTT TGTGTG TCGTTCCTTA
PF13_0251; 1110 3.31e-05 TTCATATTTA TGTGTG TAATAAAAAT*
PF13_0189; 223 3.31e-05 ATTAATAGTT TGTGTG AATATTTTGC
MAL13P1.22; 1298 3.31e-05 ACAATATAAA TGTGTG TGAAAAAAAA
MAL13P1.22; 643 3.31e-05 TATTTACATA TGTGTG TTTTTCTTCT
MAL13P1.22; 276 3.31e-05 TAGATATACA TGTGTG TGTTAATTAT*
PF13_0291; 88 3.31e-05 TATATATATG TGTGTG TAATTTAAAA
PFL0580w; 878 3.31e-05 TTAAAACGTA TGTGTG AATAAAGAGA
PFL0150w; 980 3.31e-05 TATCATTAAA TGTGTG AGAAAAAAAA
PF11_0117; 1467 3.31e-05 AAAAAAAAAG TGTGTG TAAGG
PF11_0117; 1219 3.31e-05 TATAATCTTA TGTGTG AATAAAATAT
PF11_0117; 292 3.31e-05 ATTATACACA TGTGTG TATACATCTT*
PF10_0165; 1499 3.31e-05 TCTCATTCGA TGTGTG GATACTTTTT*
PFI0530c; 1513 3.31e-05 ATTATATATA TGTGTG TATTTTGTTA*
PFI0235w; 188 3.31e-05 AAATATATGA TGTGTG TCAAATGAAA
PFE0155w; 1506 3.31e-05 ATATATTTTA TGTGTG TAGTTTTTTT*
PFF1225c; 276 3.31e-05 TATATATTTA TGTGTG TATTCGTTTT*
PFD0790c; 1654 3.31e-05 TTTTTTTTTT TGTGTG TGGTGTGAGT
PFD0790c; 757 3.31e-05 ATATTTTATT TGTGTG TAACTTTTTT*
PFD0590c; 436 3.31e-05 TATATAATCC TGTGTG GTTAAGTATT*
PFC0340w; 223 3.31e-05 ATATATTATA TGTGTG TATAAAATAT*
PFL1285c; 235 3.35e-05 ATTTTTTTTA AGGGGG AAAAAATAAA
PFL2005w; 917 3.66e-05 ATATTAAAAT AGGGTG AATATATATT
PF13_0251; 838 3.66e-05 TTTTTTTAAA AGGGTG TTCATATATG
PF13_0328; 750 3.66e-05 ATATATAAAA AGGGTG CTTTTAAAAG
PFL1655c; 141 3.97e-05 TTTGATTTCT AGTGGG ATATTTGTCT
PF14_0602; 878 6.10e-05 TACATGAAAT AGTGTG AAAAAATAAA
MAL7P1.21; 532 6.10e-05 TCATTTTATA AGTGTG TTACGCATAC
PFF1225c; 1311 6.10e-05 ATTTCATGTG AGTGTG AAAAAATGTA
PFB0840w; 1148 6.10e-05 ATTAATTTTT AGTGTG CATAAATTGA
1) MEME, zoops2
PF11_0117; 288 5.96e-09 TTATATTATA CACATGTGTGTA TACATCTTTT*
MAL13P1.22; 272 1.41e-08 TATATAGATA TACATGTGTGTG TTAATTATTT*
PFE0155w; 1751 4.17e-08 TATTTACAAT CTCAGGTGTGTG ATTAATTAAC
PF13_0291; 82 2.57e-07 TATACATATA TATATGTGTGTG TAATTTAAAA*
PFI0530c; 1509 4.86e-07 TATAATTATA TATATGTGTGTA TTTTGTTACT*
PFC0340w; 219 4.86e-07 ATTAATATAT TATATGTGTGTA TAAAATATCT*
PF13_0189; 802 5.97e-07 TTTATATTTT CTCAGGGGTGTA AATAAATATA*
PF13_0251; 1106 1.20e-06 CGATTTCATA TTTATGTGTGTA ATAAAAATGG*
PFF1225c; 272 1.20e-06 CTTTTATATA TTTATGTGTGTA TTCGTTTTAA*
PFD0790c; 753 1.49e-06 CCAAATATTT TATTTGTGTGTA ACTTTTTTTT*
PFI0235w; 694 1.58e-06 CATGAAATAA CACTTATGTGTA TATATTTATA
PF07_0023; 1785 1.73e-06 TATATATATA TGTATGTGTGTA TATTAAGGTA*
PF10_0165; 589 1.73e-06 AGAAATTCAT TCCTTATGTGTA AACCTTCGAC
PFD0590c; 792 2.14e-06 TTTGTCCTTG TTCATATGTGTA CCTATTTTTA
PF14_0602; 575 3.44e-06 ATTATGTGTA GCCATATGTGTA ATTTTGTTTA
PFL2005w; 1247 6.21e-06 AAATTTTCAT TTCATTTGTGTA ATTTTTGTGT
PFL0580w; 725 6.21e-06 ATATATGTAT TATATATGTGTA TATTTTAAAT
PFF1470c; 1941 6.21e-06 TATATATATG TATATATGTGTA CTATTCTGAA
PFL1285c; 882 9.07e-06 TTATTTATTT TCCTTTCGTGTA ATATTTAAAA
PF14_0601; 1983 1.45e-05 ATATTTATAT TATTTATGTGTA ATAAGA
PF13_0328; 1041 1.45e-05 ATATTATCTT TATATATGTGCA TATATTAAAA
PFE1345c; 1633 1.45e-05 AACATATTCA TATATACGTGTA TAATAATATA
PFB0840w; 1371 1.45e-05 TTTTTTTTTT TTTTGGTGTGGG GAATTTTTAT
PFL1120c; 1382 1.50e-05 AAGTATATAA CTTTTGTGTGTT ACATATATAT*
MAL7P1.21; 1065 1.75e-05 TAACAGTCCA TCTTTTTGTGTA CCTTTTTTTT
PFB0895c; 1472 2.95e-05 AAATATATTA TATATATGTGTT TCTCCATATA
PF10_0362; 848 3.08e-05 AATTTATACA TACTGTTGTGTT TTTTTCTTTT
PF14_0254; 361 3.67e-05 ATTTTTATTT TTTTTTTGTGTA TACATTAAAT
PFL0150w; 1324 4.53e-05 ATGTACACAT TATATATGTGCT AATTTATTAT
PFL1655c; 374 4.99e-05 TTTTATTTAA TACATTTGTCTA TATAATATAC
PFA0545c; 573 7.14e-05 AAAAAAAAAA CATTAATGTGTA CATATATATA
PF14_0177; 235 1.62e-04 TATATATATA TATATATATGTA TATAATATAT
AlignACE
5) W-GWGWG-G--AWA
TTGAGAGGGGGAAA 23 42 1
TGGTGTGGGGAATT 1 1373 1*
AGGAGAGTGAGAGA 6 997 1
AAGGGAGTGATACA 13 433 1*
ATGTGTGTGAAAAA 21 1296 1
ATGTGAGTGTGAAA 7 1305 1*
TGGGGGGGGTTTTA 7 321 1*
ATGAGAGAGATATA 3 462 1
ATGTGTGAGAAAAA 17 978 1
AAGGGAGAGAGAGA 30 1057 1
TGGTGTGTGAAAAA 29 487 1*
TAGAGAGAGCCAAA 19 598 1
ATGTGTGTGTAATT 20 84 1
ATGTGTGTGTTAAT 21 274 1*
AGGTGTGTGATTAA 9 1753 1
AAGGGAGAGTTAAT 6 1870 1
TGGTGTGAGTTTAA 6 1659 1
AlignACE
4) TGTGTGT-----W--T-WT
GGTGTGGGGAATTTTTATT 1 1374 1*
TGTGTGGTTAAGTATTATT 5 435 1*
TGTGTGTAACTTTTTTTTT 6 756 1*
TGTGTGGTGTGAGTTTAAT 6 1655 1
TGTGGGTTTGTTTTGTCAT 6 1676 1
TGTGTGTATTCGTTTTAAT 7 275 1
TGGGGGGGGTTTTATTTAT 7 321 1
TGGGGGTCTTTTTTTTTTT 9 923 1
TTTGTGATATTTCATTTTT 9 1176 1
TGTGTGTAGTTTTTTTTTT 9 1505 1*
TGTGTGGATACTTTTTCAT 13 1498 1*
TATGTGTATATTACATTTT 16 906 1
TGTGTGTGTTAATTATTTT 21 275 1*
TGTGTGTTTTTCTTCTTCT 21 642 1*
GGTGTGTGAAAAATATTAT 29 488 1*
TGTGTGTTACATATATATT 31 1385 1
Key AlignACE
#1 PFB0840w;
#3 PFC0340w;
#5 PFD0590c;
#6 PFD0790c;
#7 PFF1225c;
#9 PFE0155w;
#13 PF10_0165;
#16 PF11_0117;
#17 PFL0150w;
#19 PF13_0328;
#20 PF13_0291;
#21 MAL13P1.22;
#22 PFL1285c;
#23 PF13_0189;
#29 PF07_0023;
#30 PFL2005w;
#31 PFL1120c;
AlignACE
3) YATKTGTGKG
TTGGTGTGGG 1 1372 1*
CCTGTGTGGT 5 433 1*
TATTTGTGTG 6 752 1*
CCTTTGGGTG 7 597 1
CATGTGAGTG 7 1304 1*
AATTTGTTGG 9 490 1
CAGGTGTGTG 9 1752 1
CATTTGTTTG 16 218 1
TATGTGTGTG 20 83 1*
CATGTGTGTG 21 273 1*
AATGTGTGTG 21 1295 1*
CCTTGGGGTG 22 1518 1*
8

## Slide 9
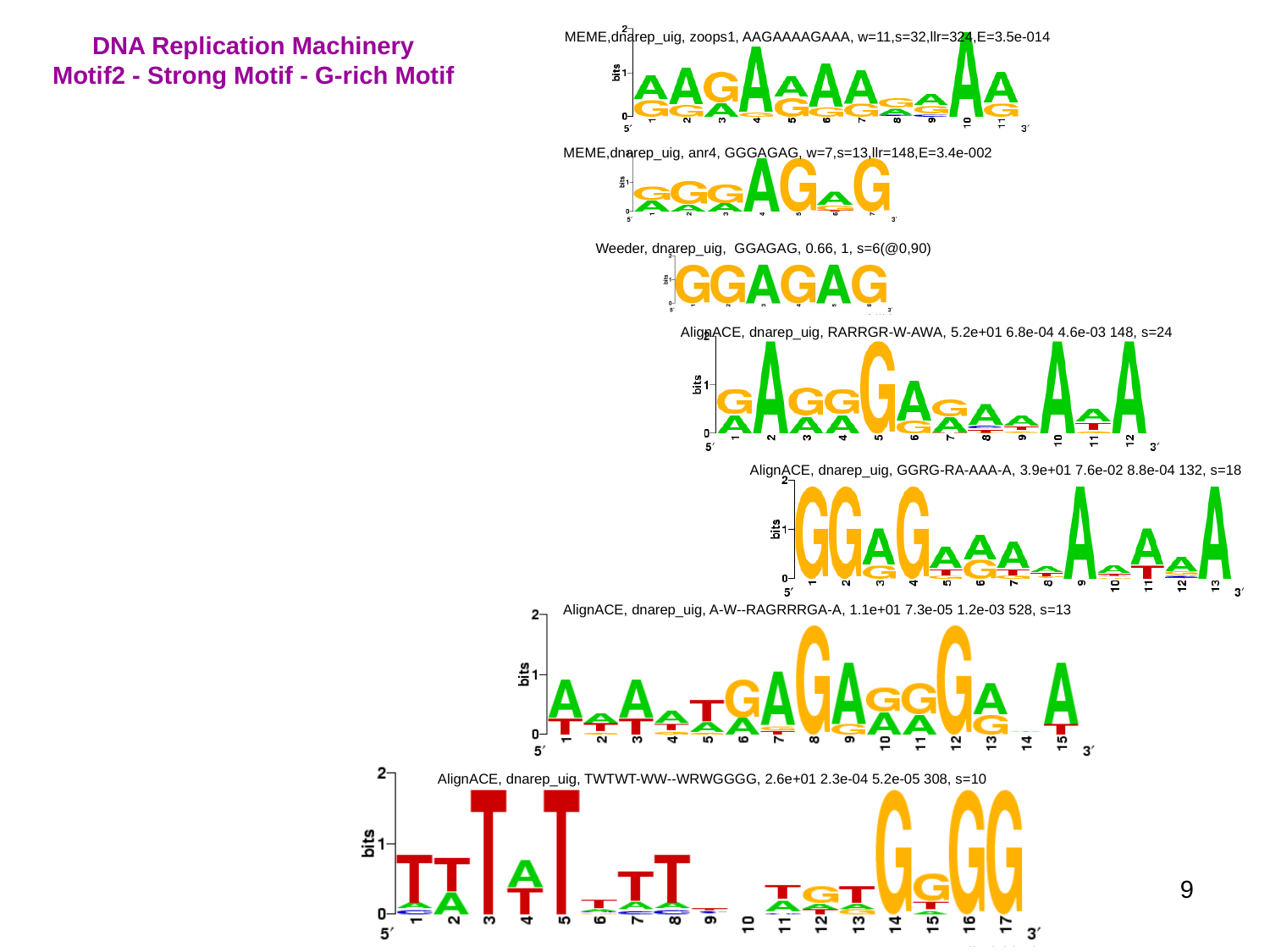

MEME,dnarep_uig, zoops1, AAGAAAAGAAA, w=11,s=32,llr=324,E=3.5e-014
MEME,dnarep_uig, anr4, GGGAGAG, w=7,s=13,llr=148,E=3.4e-002
Weeder, dnarep_uig, GGAGAG, 0.66, 1, s=6(@0,90)
AlignACE, dnarep_uig, RARRGR-W-AWA, 5.2e+01 6.8e-04 4.6e-03 148, s=24
AlignACE, dnarep_uig, GGRG-RA-AAA-A, 3.9e+01 7.6e-02 8.8e-04 132, s=18
AlignACE, dnarep_uig, A-W--RAGRRRGA-A, 1.1e+01 7.3e-05 1.2e-03 528, s=13
AlignACE, dnarep_uig, TWTWT-WW--WRWGGGG, 2.6e+01 2.3e-04 5.2e-05 308, s=10
DNA Replication Machinery
Motif2 - Strong Motif - G-rich Motif
9

## Slide 10
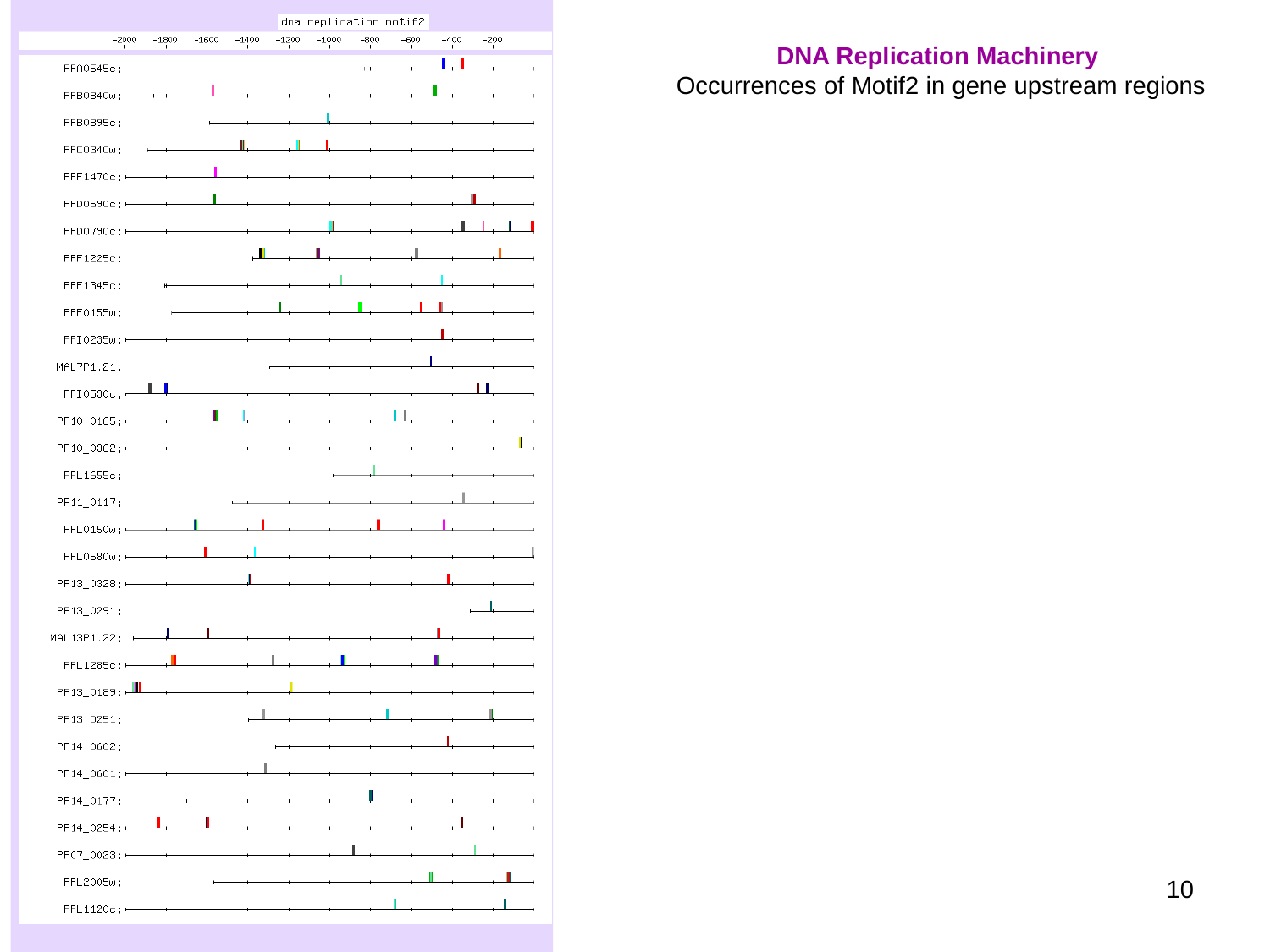

DNA Replication Machinery
Occurrences of Motif2 in gene upstream regions
10

## Slide 11
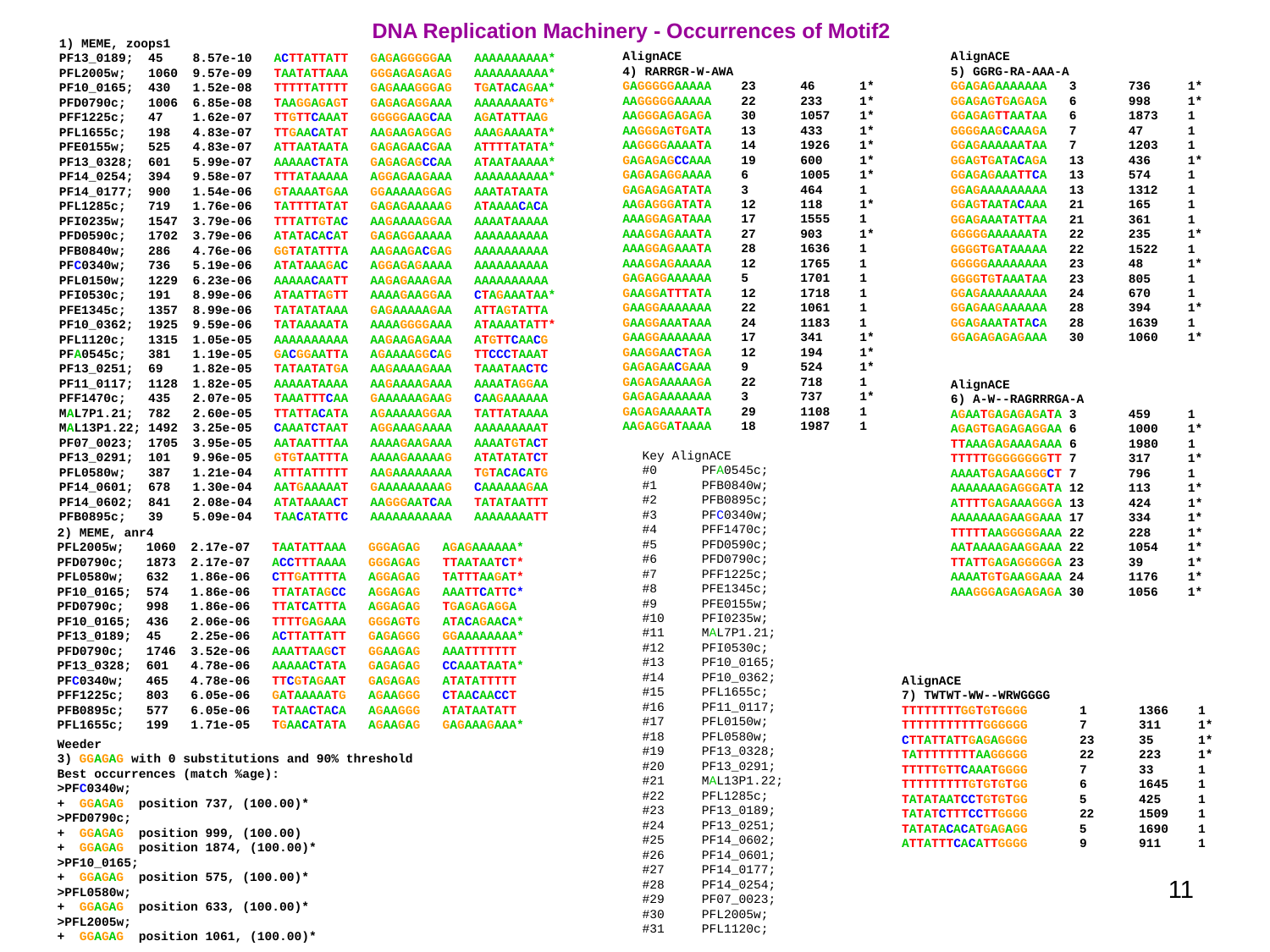

DNA Replication Machinery - Occurrences of Motif2
1) MEME, zoops1
PF13_0189; 45 8.57e-10 ACTTATTATT GAGAGGGGGAA AAAAAAAAAA*
PFL2005w; 1060 9.57e-09 TAATATTAAA GGGAGAGAGAG AAAAAAAAAA*
PF10_0165; 430 1.52e-08 TTTTTATTTT GAGAAAGGGAG TGATACAGAA*
PFD0790c; 1006 6.85e-08 TAAGGAGAGT GAGAGAGGAAA AAAAAAAATG*
PFF1225c; 47 1.62e-07 TTGTTCAAAT GGGGGAAGCAA AGATATTAAG
PFL1655c; 198 4.83e-07 TTGAACATAT AAGAAGAGGAG AAAGAAAATA*
PFE0155w; 525 4.83e-07 ATTAATAATA GAGAGAACGAA ATTTTATATA*
PF13_0328; 601 5.99e-07 AAAAACTATA GAGAGAGCCAA ATAATAAAAA*
PF14_0254; 394 9.58e-07 TTTATAAAAA AGGAGAAGAAA AAAAAAAAAA*
PF14_0177; 900 1.54e-06 GTAAAATGAA GGAAAAAGGAG AAATATAATA
PFL1285c; 719 1.76e-06 TATTTTATAT GAGAGAAAAAG ATAAAACACA
PFI0235w; 1547 3.79e-06 TTTATTGTAC AAGAAAAGGAA AAAATAAAAA
PFD0590c; 1702 3.79e-06 ATATACACAT GAGAGGAAAAA AAAAAAAAAA
PFB0840w; 286 4.76e-06 GGTATATTTA AAGAAGACGAG AAAAAAAAAA
PFC0340w; 736 5.19e-06 ATATAAAGAC AGGAGAGAAAA AAAAAAAAAA
PFL0150w; 1229 6.23e-06 AAAAACAATT AAGAGAAAGAA AAAAAAAAAA
PFI0530c; 191 8.99e-06 ATAATTAGTT AAAAGAAGGAA CTAGAAATAA*
PFE1345c; 1357 8.99e-06 TATATATAAA GAGAAAAAGAA ATTAGTATTA
PF10_0362; 1925 9.59e-06 TATAAAAATA AAAAGGGGAAA ATAAAATATT*
PFL1120c; 1315 1.05e-05 AAAAAAAAAA AAGAAGAGAAA ATGTTCAACG
PFA0545c; 381 1.19e-05 GACGGAATTA AGAAAAGGCAG TTCCCTAAAT
PF13_0251; 69 1.82e-05 TATAATATGA AAGAAAAGAAA TAAATAACTC
PF11_0117; 1128 1.82e-05 AAAAATAAAA AAGAAAAGAAA AAAATAGGAA
PFF1470c; 435 2.07e-05 TAAATTTCAA GAAAAAAGAAG CAAGAAAAAA
MAL7P1.21; 782 2.60e-05 TTATTACATA AGAAAAAGGAA TATTATAAAA
MAL13P1.22; 1492 3.25e-05 CAAATCTAAT AGGAAAGAAAA AAAAAAAAAT
PF07_0023; 1705 3.95e-05 AATAATTTAA AAAAGAAGAAA AAAATGTACT
PF13_0291; 101 9.96e-05 GTGTAATTTA AAAAGAAAAAG ATATATATCT
PFL0580w; 387 1.21e-04 ATTTATTTTT AAGAAAAAAAA TGTACACATG
PF14_0601; 678 1.30e-04 AATGAAAAAT GAAAAAAAAAG CAAAAAAGAA
PF14_0602; 841 2.08e-04 ATATAAAACT AAGGGAATCAA TATATAATTT
PFB0895c; 39 5.09e-04 TAACATATTC AAAAAAAAAAA AAAAAAAATT
AlignACE
4) RARRGR-W-AWA
GAGGGGGAAAAA 23 46 1*
AAGGGGGAAAAA 22 233 1*
AAGGGAGAGAGA 30 1057 1*
AAGGGAGTGATA 13 433 1*
AAGGGGAAAATA 14 1926 1*
GAGAGAGCCAAA 19 600 1*
GAGAGAGGAAAA 6 1005 1*
GAGAGAGATATA 3 464 1
AAGAGGGATATA 12 118 1*
AAAGGAGATAAA 17 1555 1
AAAGGAGAAATA 27 903 1*
AAAGGAGAAATA 28 1636 1
AAAGGAGAAAAA 12 1765 1
GAGAGGAAAAAA 5 1701 1
GAAGGATTTATA 12 1718 1
GAAGGAAAAAAA 22 1061 1
GAAGGAAATAAA 24 1183 1
GAAGGAAAAAAA 17 341 1*
GAAGGAACTAGA 12 194 1*
GAGAGAACGAAA 9 524 1*
GAGAGAAAAAGA 22 718 1
GAGAGAAAAAAA 3 737 1*
GAGAGAAAAATA 29 1108 1
AAGAGGATAAAA 18 1987 1
AlignACE
5) GGRG-RA-AAA-A
GGAGAGAAAAAAA 3 736 1*
GGAGAGTGAGAGA 6 998 1*
GGAGAGTTAATAA 6 1873 1
GGGGAAGCAAAGA 7 47 1
GGAGAAAAAATAA 7 1203 1
GGAGTGATACAGA 13 436 1*
GGAGAGAAATTCA 13 574 1
GGAGAAAAAAAAA 13 1312 1
GGAGTAATACAAA 21 165 1
GGAGAAATATTAA 21 361 1
GGGGGAAAAAATA 22 235 1*
GGGGTGATAAAAA 22 1522 1
GGGGGAAAAAAAA 23 48 1*
GGGGTGTAAATAA 23 805 1
GGAGAAAAAAAAA 24 670 1
GGAGAAGAAAAAA 28 394 1*
GGAGAAATATACA 28 1639 1
GGAGAGAGAGAAA 30 1060 1*
AlignACE
6) A-W--RAGRRRGA-A
AGAATGAGAGAGATA 3 459 1
AGAGTGAGAGAGGAA 6 1000 1*
TTAAAGAGAAAGAAA 6 1980 1
TTTTTGGGGGGGGTT 7 317 1*
AAAATGAGAAGGGCT 7 796 1
AAAAAAAGAGGGATA 12 113 1*
ATTTTGAGAAAGGGA 13 424 1*
AAAAAAAGAAGGAAA 17 334 1*
TTTTTAAGGGGGAAA 22 228 1*
AATAAAAGAAGGAAA 22 1054 1*
TTATTGAGAGGGGGA 23 39 1*
AAAATGTGAAGGAAA 24 1176 1*
AAAGGGAGAGAGAGA 30 1056 1*
Key AlignACE
#0 PFA0545c;
#1 PFB0840w;
#2 PFB0895c;
#3 PFC0340w;
#4 PFF1470c;
#5 PFD0590c;
#6 PFD0790c;
#7 PFF1225c;
#8 PFE1345c;
#9 PFE0155w;
#10 PFI0235w;
#11 MAL7P1.21;
#12 PFI0530c;
#13 PF10_0165;
#14 PF10_0362;
#15 PFL1655c;
#16 PF11_0117;
#17 PFL0150w;
#18 PFL0580w;
#19 PF13_0328;
#20 PF13_0291;
#21 MAL13P1.22;
#22 PFL1285c;
#23 PF13_0189;
#24 PF13_0251;
#25 PF14_0602;
#26 PF14_0601;
#27 PF14_0177;
#28 PF14_0254;
#29 PF07_0023;
#30 PFL2005w;
#31 PFL1120c;
2) MEME, anr4
PFL2005w; 1060 2.17e-07 TAATATTAAA GGGAGAG AGAGAAAAAA*
PFD0790c; 1873 2.17e-07 ACCTTTAAAA GGGAGAG TTAATAATCT*
PFL0580w; 632 1.86e-06 CTTGATTTTA AGGAGAG TATTTAAGAT*
PF10_0165; 574 1.86e-06 TTATATAGCC AGGAGAG AAATTCATTC*
PFD0790c; 998 1.86e-06 TTATCATTTA AGGAGAG TGAGAGAGGA
PF10_0165; 436 2.06e-06 TTTTGAGAAA GGGAGTG ATACAGAACA*
PF13_0189; 45 2.25e-06 ACTTATTATT GAGAGGG GGAAAAAAAA*
PFD0790c; 1746 3.52e-06 AAATTAAGCT GGAAGAG AAATTTTTTT
PF13_0328; 601 4.78e-06 AAAAACTATA GAGAGAG CCAAATAATA*
PFC0340w; 465 4.78e-06 TTCGTAGAAT GAGAGAG ATATATTTTT
PFF1225c; 803 6.05e-06 GATAAAAATG AGAAGGG CTAACAACCT
PFB0895c; 577 6.05e-06 TATAACTACA AGAAGGG ATATAATATT
PFL1655c; 199 1.71e-05 TGAACATATA AGAAGAG GAGAAAGAAA*
AlignACE
7) TWTWT-WW--WRWGGGG
TTTTTTTTGGTGTGGGG 1 1366 1
TTTTTTTTTTTGGGGGG 7 311 1*
CTTATTATTGAGAGGGG 23 35 1*
TATTTTTTTTAAGGGGG 22 223 1*
TTTTTGTTCAAATGGGG 7 33 1
TTTTTTTTTGTGTGTGG 6 1645 1
TATATAATCCTGTGTGG 5 425 1
TATATCTTTCCTTGGGG 22 1509 1
TATATACACATGAGAGG 5 1690 1
ATTATTTCACATTGGGG 9 911 1
Weeder
3) GGAGAG with 0 substitutions and 90% threshold
Best occurrences (match %age):
>PFC0340w;
+ GGAGAG position 737, (100.00)*
>PFD0790c;
+ GGAGAG position 999, (100.00)
+ GGAGAG position 1874, (100.00)*
>PF10_0165;
+ GGAGAG position 575, (100.00)*
>PFL0580w;
+ GGAGAG position 633, (100.00)*
>PFL2005w;
+ GGAGAG position 1061, (100.00)*
11

## Slide 12
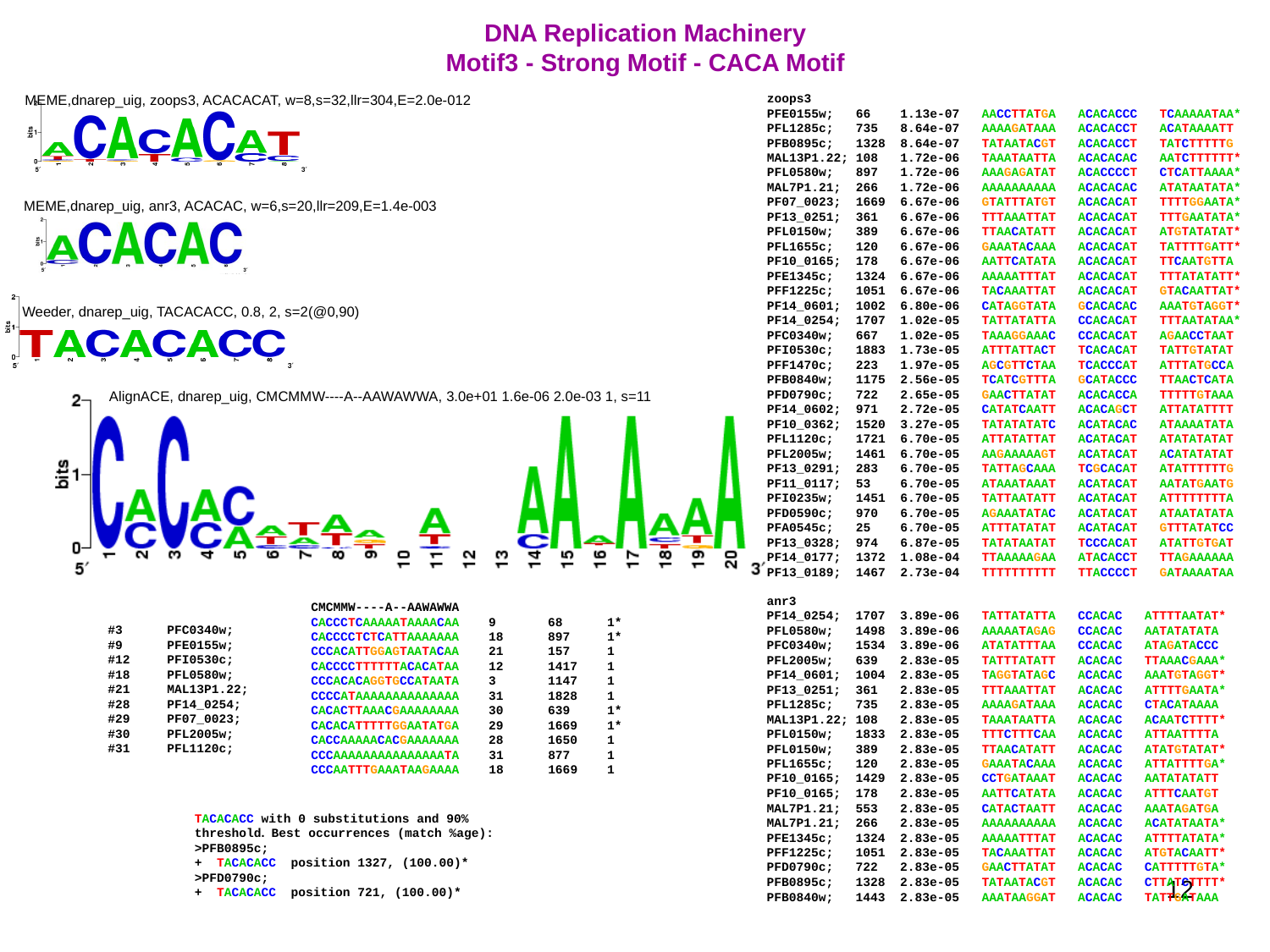

DNA Replication Machinery
Motif3 - Strong Motif - CACA Motif
zoops3
PFE0155w; 66 1.13e-07 AACCTTATGA ACACACCC TCAAAAATAA*
PFL1285c; 735 8.64e-07 AAAAGATAAA ACACACCT ACATAAAATT
PFB0895c; 1328 8.64e-07 TATAATACGT ACACACCT TATCTTTTTG
MAL13P1.22; 108 1.72e-06 TAAATAATTA ACACACAC AATCTTTTTT*
PFL0580w; 897 1.72e-06 AAAGAGATAT ACACCCCT CTCATTAAAA*
MAL7P1.21; 266 1.72e-06 AAAAAAAAAA ACACACAC ATATAATATA*
PF07_0023; 1669 6.67e-06 GTATTTATGT ACACACAT TTTTGGAATA*
PF13_0251; 361 6.67e-06 TTTAAATTAT ACACACAT TTTGAATATA*
PFL0150w; 389 6.67e-06 TTAACATATT ACACACAT ATGTATATAT*
PFL1655c; 120 6.67e-06 GAAATACAAA ACACACAT TATTTTGATT*
PF10_0165; 178 6.67e-06 AATTCATATA ACACACAT TTCAATGTTA
PFE1345c; 1324 6.67e-06 AAAAATTTAT ACACACAT TTTATATATT*
PFF1225c; 1051 6.67e-06 TACAAATTAT ACACACAT GTACAATTAT*
PF14_0601; 1002 6.80e-06 CATAGGTATA GCACACAC AAATGTAGGT*
PF14_0254; 1707 1.02e-05 TATTATATTA CCACACAT TTTAATATAA*
PFC0340w; 667 1.02e-05 TAAAGGAAAC CCACACAT AGAACCTAAT
PFI0530c; 1883 1.73e-05 ATTTATTACT TCACACAT TATTGTATAT
PFF1470c; 223 1.97e-05 AGCGTTCTAA TCACCCAT ATTTATGCCA
PFB0840w; 1175 2.56e-05 TCATCGTTTA GCATACCC TTAACTCATA
PFD0790c; 722 2.65e-05 GAACTTATAT ACACACCA TTTTTGTAAA
PF14_0602; 971 2.72e-05 CATATCAATT ACACAGCT ATTATATTTT
PF10_0362; 1520 3.27e-05 TATATATATC ACATACAC ATAAAATATA
PFL1120c; 1721 6.70e-05 ATTATATTAT ACATACAT ATATATATAT
PFL2005w; 1461 6.70e-05 AAGAAAAAGT ACATACAT ACATATATAT
PF13_0291; 283 6.70e-05 TATTAGCAAA TCGCACAT ATATTTTTTG
PF11_0117; 53 6.70e-05 ATAAATAAAT ACATACAT AATATGAATG
PFI0235w; 1451 6.70e-05 TATTAATATT ACATACAT ATTTTTTTTA
PFD0590c; 970 6.70e-05 AGAAATATAC ACATACAT ATAATATATA
PFA0545c; 25 6.70e-05 ATTTATATAT ACATACAT GTTTATATCC
PF13_0328; 974 6.87e-05 TATATAATAT TCCCACAT ATATTGTGAT
PF14_0177; 1372 1.08e-04 TTAAAAAGAA ATACACCT TTAGAAAAAA
PF13_0189; 1467 2.73e-04 TTTTTTTTTT TTACCCCT GATAAAATAA
MEME,dnarep_uig, zoops3, ACACACAT, w=8,s=32,llr=304,E=2.0e-012
MEME,dnarep_uig, anr3, ACACAC, w=6,s=20,llr=209,E=1.4e-003
Weeder, dnarep_uig, TACACACC, 0.8, 2, s=2(@0,90)
AlignACE, dnarep_uig, CMCMMW----A--AAWAWWA, 3.0e+01 1.6e-06 2.0e-03 1, s=11
anr3
PF14_0254; 1707 3.89e-06 TATTATATTA CCACAC ATTTTAATAT*
PFL0580w; 1498 3.89e-06 AAAAATAGAG CCACAC AATATATATA
PFC0340w; 1534 3.89e-06 ATATATTTAA CCACAC ATAGATACCC
PFL2005w; 639 2.83e-05 TATTTATATT ACACAC TTAAACGAAA*
PF14_0601; 1004 2.83e-05 TAGGTATAGC ACACAC AAATGTAGGT*
PF13_0251; 361 2.83e-05 TTTAAATTAT ACACAC ATTTTGAATA*
PFL1285c; 735 2.83e-05 AAAAGATAAA ACACAC CTACATAAAA
MAL13P1.22; 108 2.83e-05 TAAATAATTA ACACAC ACAATCTTTT*
PFL0150w; 1833 2.83e-05 TTTCTTTCAA ACACAC ATTAATTTTA
PFL0150w; 389 2.83e-05 TTAACATATT ACACAC ATATGTATAT*
PFL1655c; 120 2.83e-05 GAAATACAAA ACACAC ATTATTTTGA*
PF10_0165; 1429 2.83e-05 CCTGATAAAT ACACAC AATATATATT
PF10_0165; 178 2.83e-05 AATTCATATA ACACAC ATTTCAATGT
MAL7P1.21; 553 2.83e-05 CATACTAATT ACACAC AAATAGATGA
MAL7P1.21; 266 2.83e-05 AAAAAAAAAA ACACAC ACATATAATA*
PFE1345c; 1324 2.83e-05 AAAAATTTAT ACACAC ATTTTATATA*
PFF1225c; 1051 2.83e-05 TACAAATTAT ACACAC ATGTACAATT*
PFD0790c; 722 2.83e-05 GAACTTATAT ACACAC CATTTTTGTA*
PFB0895c; 1328 2.83e-05 TATAATACGT ACACAC CTTATCTTTT*
PFB0840w; 1443 2.83e-05 AAATAAGGAT ACACAC TATTGATAAA
CMCMMW----A--AAWAWWA
CACCCTCAAAAATAAAACAA 9 68 1*
CACCCCTCTCATTAAAAAAA 18 897 1*
CCCACATTGGAGTAATACAA 21 157 1
CACCCCTTTTTTACACATAA 12 1417 1
CCCACACAGGTGCCATAATA 3 1147 1
CCCCATAAAAAAAAAAAAAA 31 1828 1
CACACTTAAACGAAAAAAAA 30 639 1*
CACACATTTTTGGAATATGA 29 1669 1*
CACCAAAAACACGAAAAAAA 28 1650 1
CCCAAAAAAAAAAAAAAATA 31 877 1
CCCAATTTGAAATAAGAAAA 18 1669 1
#3 PFC0340w;
#9 PFE0155w;
#12 PFI0530c;
#18 PFL0580w;
#21 MAL13P1.22;
#28 PF14_0254;
#29 PF07_0023;
#30 PFL2005w;
#31 PFL1120c;
TACACACC with 0 substitutions and 90%
threshold. Best occurrences (match %age):
>PFB0895c;
+ TACACACC position 1327, (100.00)*
>PFD0790c;
+ TACACACC position 721, (100.00)*
12

## Slide 13
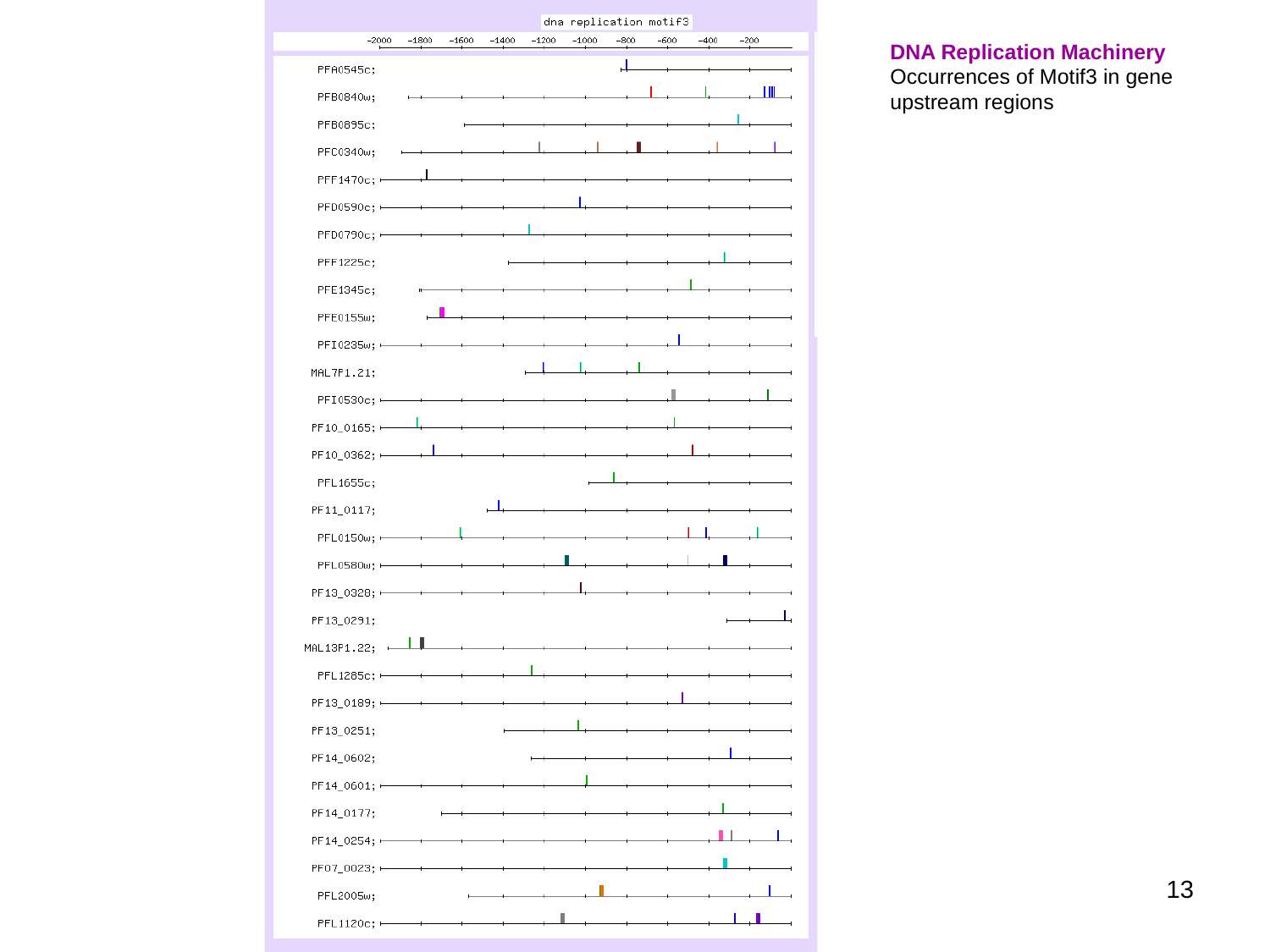

DNA Replication Machinery
Occurrences of Motif3 in gene
upstream regions
13

## Slide 14
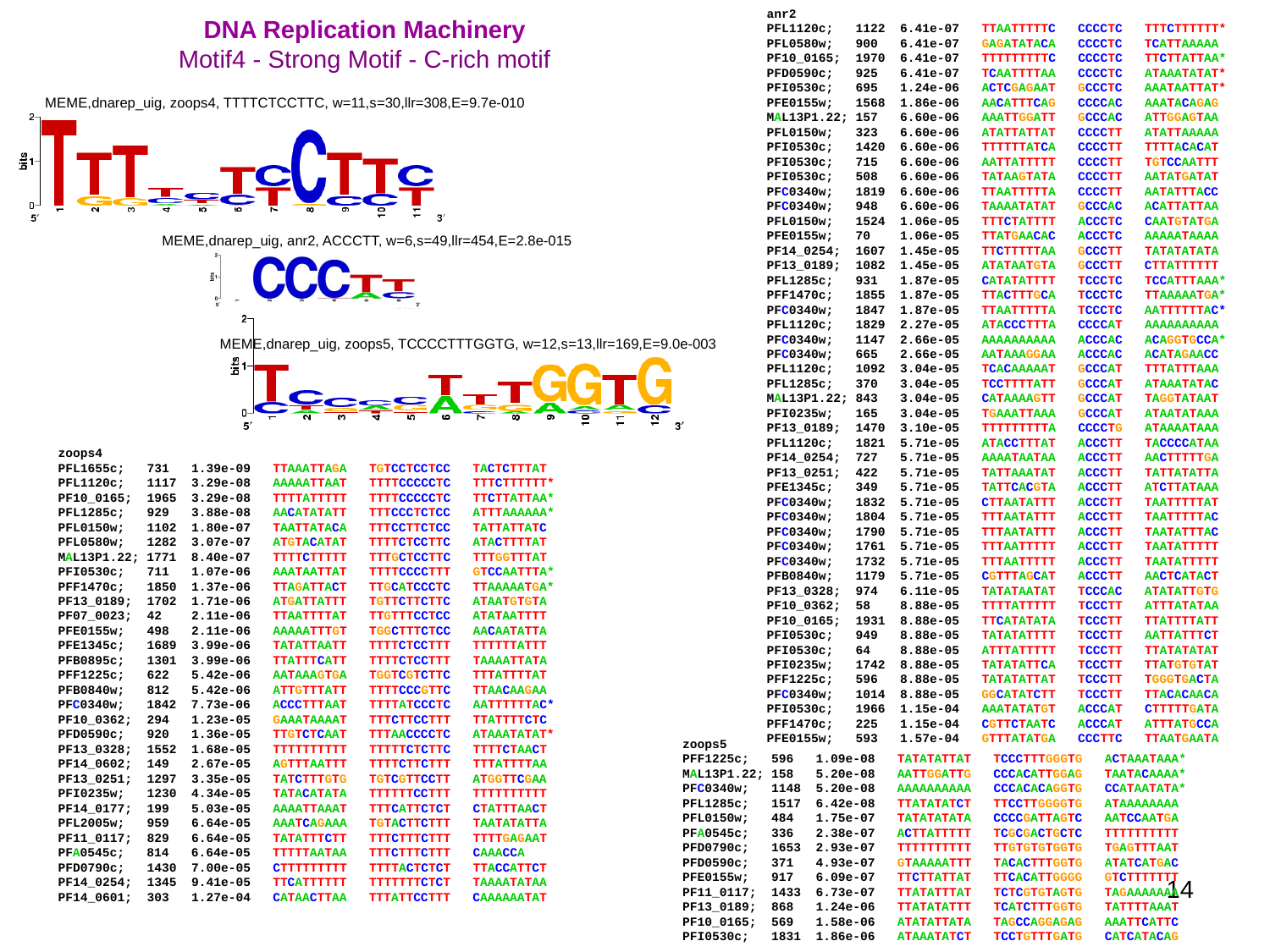

anr2
PFL1120c; 1122 6.41e-07 TTAATTTTTC CCCCTC TTTCTTTTTT*
PFL0580w; 900 6.41e-07 GAGATATACA CCCCTC TCATTAAAAA
PF10_0165; 1970 6.41e-07 TTTTTTTTTC CCCCTC TTCTTATTAA*
PFD0590c; 925 6.41e-07 TCAATTTTAA CCCCTC ATAAATATAT*
PFI0530c; 695 1.24e-06 ACTCGAGAAT GCCCTC AAATAATTAT*
PFE0155w; 1568 1.86e-06 AACATTTCAG CCCCAC AAATACAGAG
MAL13P1.22; 157 6.60e-06 AAATTGGATT GCCCAC ATTGGAGTAA
PFL0150w; 323 6.60e-06 ATATTATTAT CCCCTT ATATTAAAAA
PFI0530c; 1420 6.60e-06 TTTTTTATCA CCCCTT TTTTACACAT
PFI0530c; 715 6.60e-06 AATTATTTTT CCCCTT TGTCCAATTT
PFI0530c; 508 6.60e-06 TATAAGTATA CCCCTT AATATGATAT
PFC0340w; 1819 6.60e-06 TTAATTTTTA CCCCTT AATATTTACC
PFC0340w; 948 6.60e-06 TAAAATATAT GCCCAC ACATTATTAA
PFL0150w; 1524 1.06e-05 TTTCTATTTT ACCCTC CAATGTATGA
PFE0155w; 70 1.06e-05 TTATGAACAC ACCCTC AAAAATAAAA
PF14_0254; 1607 1.45e-05 TTCTTTTTAA GCCCTT TATATATATA
PF13_0189; 1082 1.45e-05 ATATAATGTA GCCCTT CTTATTTTTT
PFL1285c; 931 1.87e-05 CATATATTTT TCCCTC TCCATTTAAA*
PFF1470c; 1855 1.87e-05 TTACTTTGCA TCCCTC TTAAAAATGA*
PFC0340w; 1847 1.87e-05 TTAATTTTTA TCCCTC AATTTTTTAC*
PFL1120c; 1829 2.27e-05 ATACCCTTTA CCCCAT AAAAAAAAAA
PFC0340w; 1147 2.66e-05 AAAAAAAAAA ACCCAC ACAGGTGCCA*
PFC0340w; 665 2.66e-05 AATAAAGGAA ACCCAC ACATAGAACC
PFL1120c; 1092 3.04e-05 TCACAAAAAT GCCCAT TTTATTTAAA
PFL1285c; 370 3.04e-05 TCCTTTTATT GCCCAT ATAAATATAC
MAL13P1.22; 843 3.04e-05 CATAAAAGTT GCCCAT TAGGTATAAT
PFI0235w; 165 3.04e-05 TGAAATTAAA GCCCAT ATAATATAAA
PF13_0189; 1470 3.10e-05 TTTTTTTTTA CCCCTG ATAAAATAAA
PFL1120c; 1821 5.71e-05 ATACCTTTAT ACCCTT TACCCCATAA
PF14_0254; 727 5.71e-05 AAAATAATAA ACCCTT AACTTTTTGA
PF13_0251; 422 5.71e-05 TATTAAATAT ACCCTT TATTATATTA
PFE1345c; 349 5.71e-05 TATTCACGTA ACCCTT ATCTTATAAA
PFC0340w; 1832 5.71e-05 CTTAATATTT ACCCTT TAATTTTTAT
PFC0340w; 1804 5.71e-05 TTTAATATTT ACCCTT TAATTTTTAC
PFC0340w; 1790 5.71e-05 TTTAATATTT ACCCTT TAATATTTAC
PFC0340w; 1761 5.71e-05 TTTAATTTTT ACCCTT TAATATTTTT
PFC0340w; 1732 5.71e-05 TTTAATTTTT ACCCTT TAATATTTTT
PFB0840w; 1179 5.71e-05 CGTTTAGCAT ACCCTT AACTCATACT
PF13_0328; 974 6.11e-05 TATATAATAT TCCCAC ATATATTGTG
PF10_0362; 58 8.88e-05 TTTTATTTTT TCCCTT ATTTATATAA
PF10_0165; 1931 8.88e-05 TTCATATATA TCCCTT TTATTTTATT
PFI0530c; 949 8.88e-05 TATATATTTT TCCCTT AATTATTTCT
PFI0530c; 64 8.88e-05 ATTTATTTTT TCCCTT TTATATATAT
PFI0235w; 1742 8.88e-05 TATATATTCA TCCCTT TTATGTGTAT
PFF1225c; 596 8.88e-05 TATATATTAT TCCCTT TGGGTGACTA
PFC0340w; 1014 8.88e-05 GGCATATCTT TCCCTT TTACACAACA
PFI0530c; 1966 1.15e-04 AAATATATGT ACCCAT CTTTTTGATA
PFF1470c; 225 1.15e-04 CGTTCTAATC ACCCAT ATTTATGCCA
PFE0155w; 593 1.57e-04 GTTTATATGA CCCTTC TTAATGAATA
DNA Replication Machinery
Motif4 - Strong Motif - C-rich motif
MEME,dnarep_uig, zoops4, TTTTCTCCTTC, w=11,s=30,llr=308,E=9.7e-010
MEME,dnarep_uig, anr2, ACCCTT, w=6,s=49,llr=454,E=2.8e-015
MEME,dnarep_uig, zoops5, TCCCCTTTGGTG, w=12,s=13,llr=169,E=9.0e-003
zoops4
PFL1655c; 731 1.39e-09 TTAAATTAGA TGTCCTCCTCC TACTCTTTAT
PFL1120c; 1117 3.29e-08 AAAAATTAAT TTTTCCCCCTC TTTCTTTTTT*
PF10_0165; 1965 3.29e-08 TTTTATTTTT TTTTCCCCCTC TTCTTATTAA*
PFL1285c; 929 3.88e-08 AACATATATT TTTCCCTCTCC ATTTAAAAAA*
PFL0150w; 1102 1.80e-07 TAATTATACA TTTCCTTCTCC TATTATTATC
PFL0580w; 1282 3.07e-07 ATGTACATAT TTTTCTCCTTC ATACTTTTAT
MAL13P1.22; 1771 8.40e-07 TTTTCTTTTT TTTGCTCCTTC TTTGGTTTAT
PFI0530c; 711 1.07e-06 AAATAATTAT TTTTCCCCTTT GTCCAATTTA*
PFF1470c; 1850 1.37e-06 TTAGATTACT TTGCATCCCTC TTAAAAATGA*
PF13_0189; 1702 1.71e-06 ATGATTATTT TGTTCTTCTTC ATAATGTGTA
PF07_0023; 42 2.11e-06 TTAATTTTAT TTGTTTCCTCC ATATAATTTT
PFE0155w; 498 2.11e-06 AAAAATTTGT TGGCTTTCTCC AACAATATTA
PFE1345c; 1689 3.99e-06 TATATTAATT TTTTCTCCTTT TTTTTTATTT
PFB0895c; 1301 3.99e-06 TTATTTCATT TTTTCTCCTTT TAAAATTATA
PFF1225c; 622 5.42e-06 AATAAAGTGA TGGTCGTCTTC TTTATTTTAT
PFB0840w; 812 5.42e-06 ATTGTTTATT TTTTCCCGTTC TTAACAAGAA
PFC0340w; 1842 7.73e-06 ACCCTTTAAT TTTTATCCCTC AATTTTTTAC*
PF10_0362; 294 1.23e-05 GAAATAAAAT TTTCTTCCTTT TTATTTTCTC
PFD0590c; 920 1.36e-05 TTGTCTCAAT TTTAACCCCTC ATAAATATAT*
PF13_0328; 1552 1.68e-05 TTTTTTTTTT TTTTTCTCTTC TTTTCTAACT
PF14_0602; 149 2.67e-05 AGTTTAATTT TTTTCTTCTTT TTTATTTTAA
PF13_0251; 1297 3.35e-05 TATCTTTGTG TGTCGTTCCTT ATGGTTCGAA
PFI0235w; 1230 4.34e-05 TATACATATA TTTTTTCCTTT TTTTTTTTTT
PF14_0177; 199 5.03e-05 AAAATTAAAT TTTCATTCTCT CTATTTAACT
PFL2005w; 959 6.64e-05 AAATCAGAAA TGTACTTCTTT TAATATATTA
PF11_0117; 829 6.64e-05 TATATTTCTT TTTCTTTCTTT TTTTGAGAAT
PFA0545c; 814 6.64e-05 TTTTTAATAA TTTCTTTCTTT CAAACCA
PFD0790c; 1430 7.00e-05 CTTTTTTTTT TTTTACTCTCT TTACCATTCT
PF14_0254; 1345 9.41e-05 TTCATTTTTT TTTTTTTCTCT TAAAATATAA
PF14_0601; 303 1.27e-04 CATAACTTAA TTTATTCCTTT CAAAAAATAT
zoops5
PFF1225c; 596 1.09e-08 TATATATTAT TCCCTTTGGGTG ACTAAATAAA*
MAL13P1.22; 158 5.20e-08 AATTGGATTG CCCACATTGGAG TAATACAAAA*
PFC0340w; 1148 5.20e-08 AAAAAAAAAA CCCACACAGGTG CCATAATATA*
PFL1285c; 1517 6.42e-08 TTATATATCT TTCCTTGGGGTG ATAAAAAAAA
PFL0150w; 484 1.75e-07 TATATATATA CCCCGATTAGTC AATCCAATGA
PFA0545c; 336 2.38e-07 ACTTATTTTT TCGCGACTGCTC TTTTTTTTTT
PFD0790c; 1653 2.93e-07 TTTTTTTTTT TTGTGTGTGGTG TGAGTTTAAT
PFD0590c; 371 4.93e-07 GTAAAAATTT TACACTTTGGTG ATATCATGAC
PFE0155w; 917 6.09e-07 TTCTTATTAT TTCACATTGGGG GTCTTTTTTT
PF11_0117; 1433 6.73e-07 TTATATTTAT TCTCGTGTAGTG TAGAAAAAAA
PF13_0189; 868 1.24e-06 TTATATATTT TCATCTTTGGTG TATTTTAAAT
PF10_0165; 569 1.58e-06 ATATATTATA TAGCCAGGAGAG AAATTCATTC
PFI0530c; 1831 1.86e-06 ATAAATATCT TCCTGTTTGATG CATCATACAG
14

## Slide 15
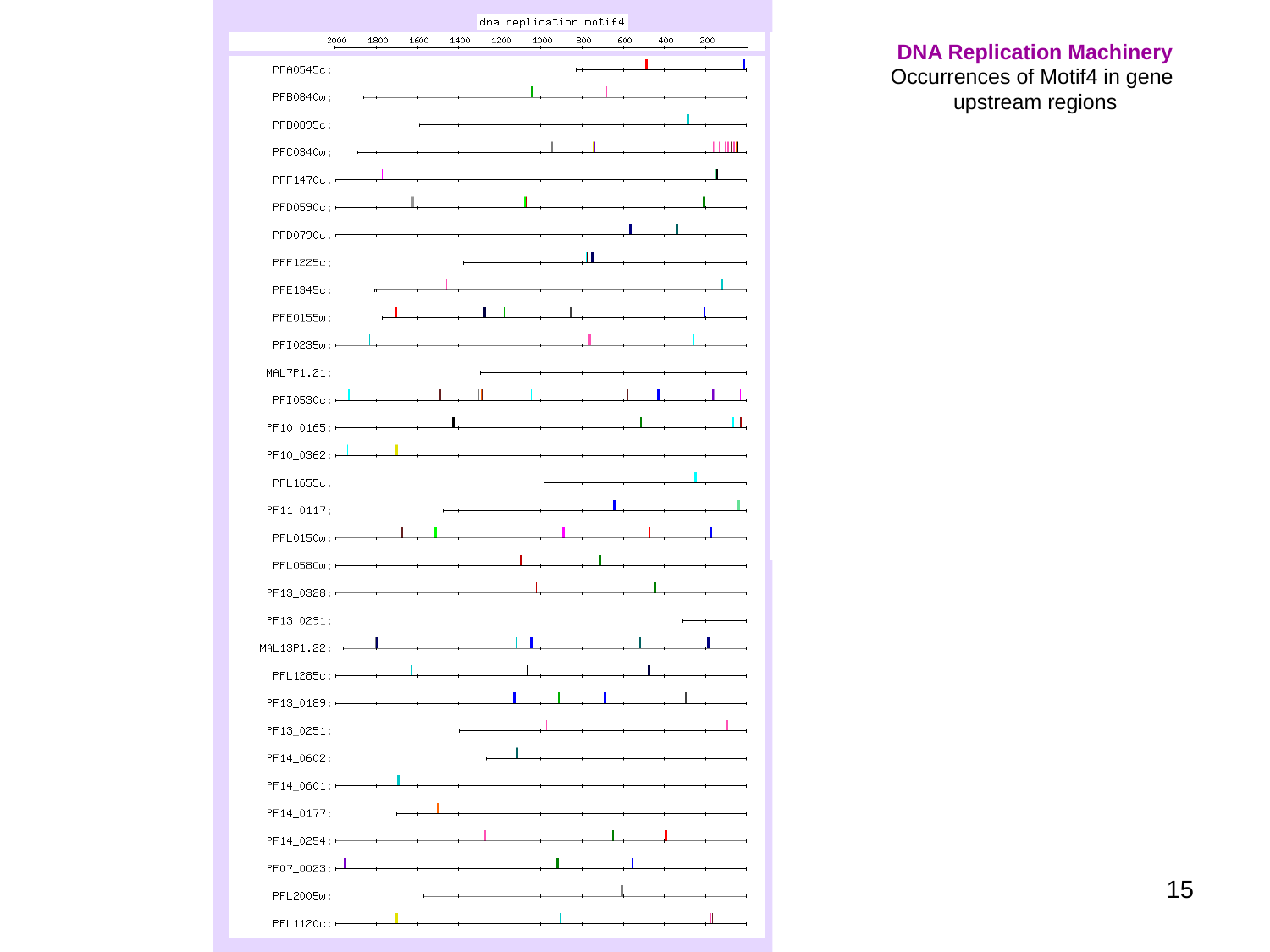

DNA Replication Machinery
Occurrences of Motif4 in gene
upstream regions
15

## Slide 16
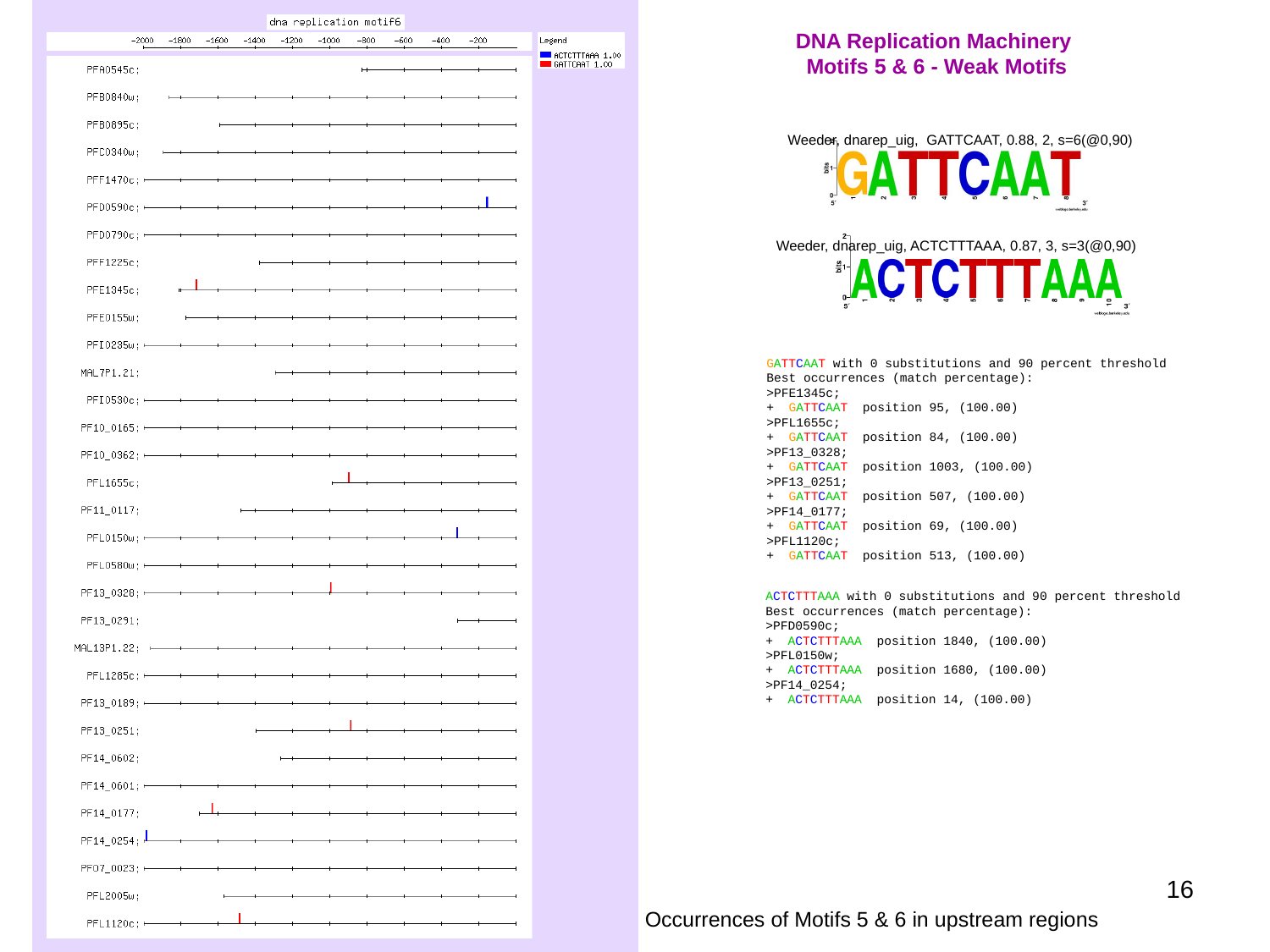

DNA Replication Machinery
Motifs 5 & 6 - Weak Motifs
Weeder, dnarep_uig, GATTCAAT, 0.88, 2, s=6(@0,90)
Weeder, dnarep_uig, ACTCTTTAAA, 0.87, 3, s=3(@0,90)
GATTCAAT with 0 substitutions and 90 percent threshold
Best occurrences (match percentage):
>PFE1345c;
+ GATTCAAT position 95, (100.00)
>PFL1655c;
+ GATTCAAT position 84, (100.00)
>PF13_0328;
+ GATTCAAT position 1003, (100.00)
>PF13_0251;
+ GATTCAAT position 507, (100.00)
>PF14_0177;
+ GATTCAAT position 69, (100.00)
>PFL1120c;
+ GATTCAAT position 513, (100.00)
ACTCTTTAAA with 0 substitutions and 90 percent threshold
Best occurrences (match percentage):
>PFD0590c;
+ ACTCTTTAAA position 1840, (100.00)
>PFL0150w;
+ ACTCTTTAAA position 1680, (100.00)
>PF14_0254;
+ ACTCTTTAAA position 14, (100.00)
16
Occurrences of Motifs 5 & 6 in upstream regions

## Slide 17
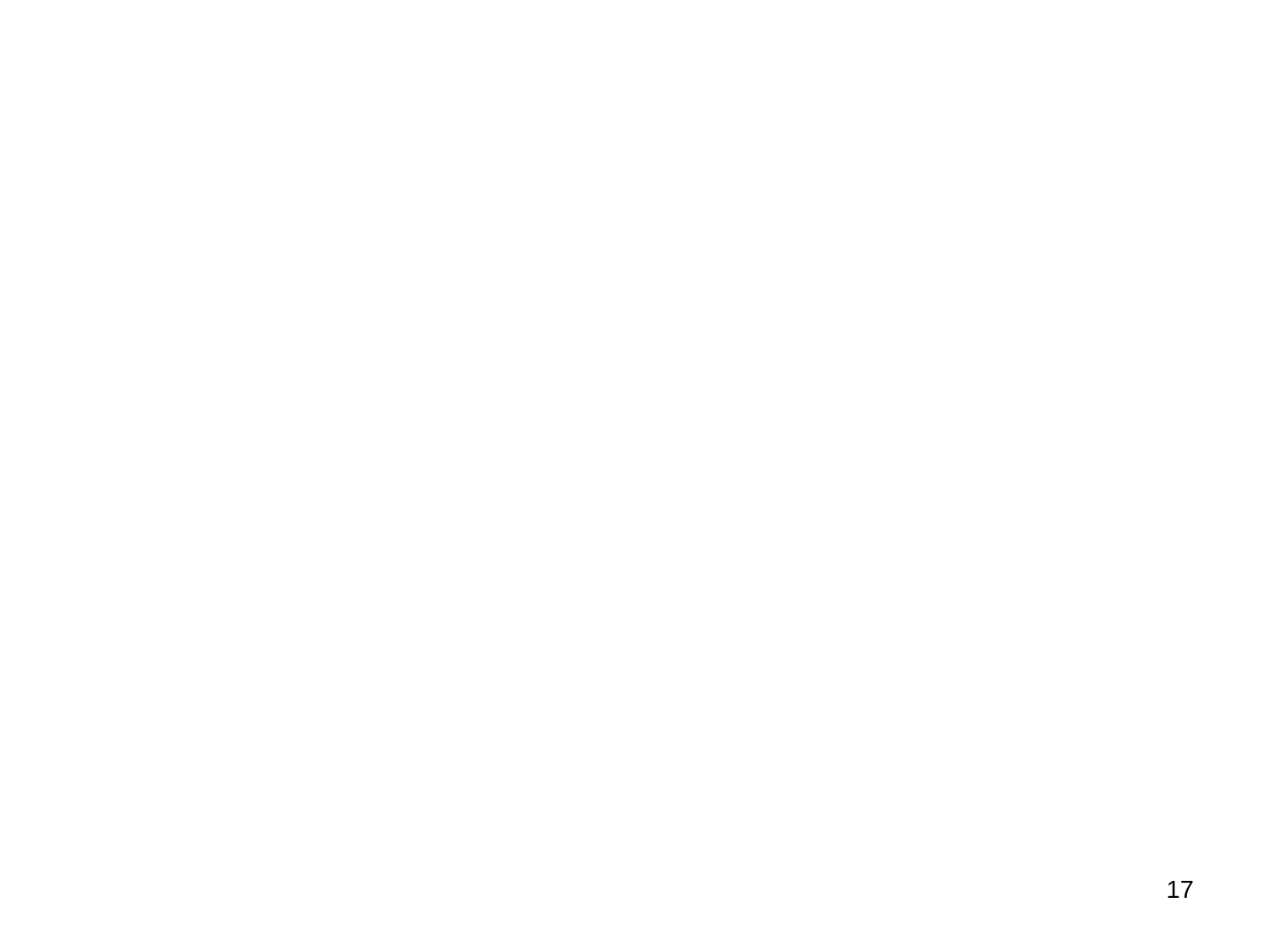

17

## Slide 18
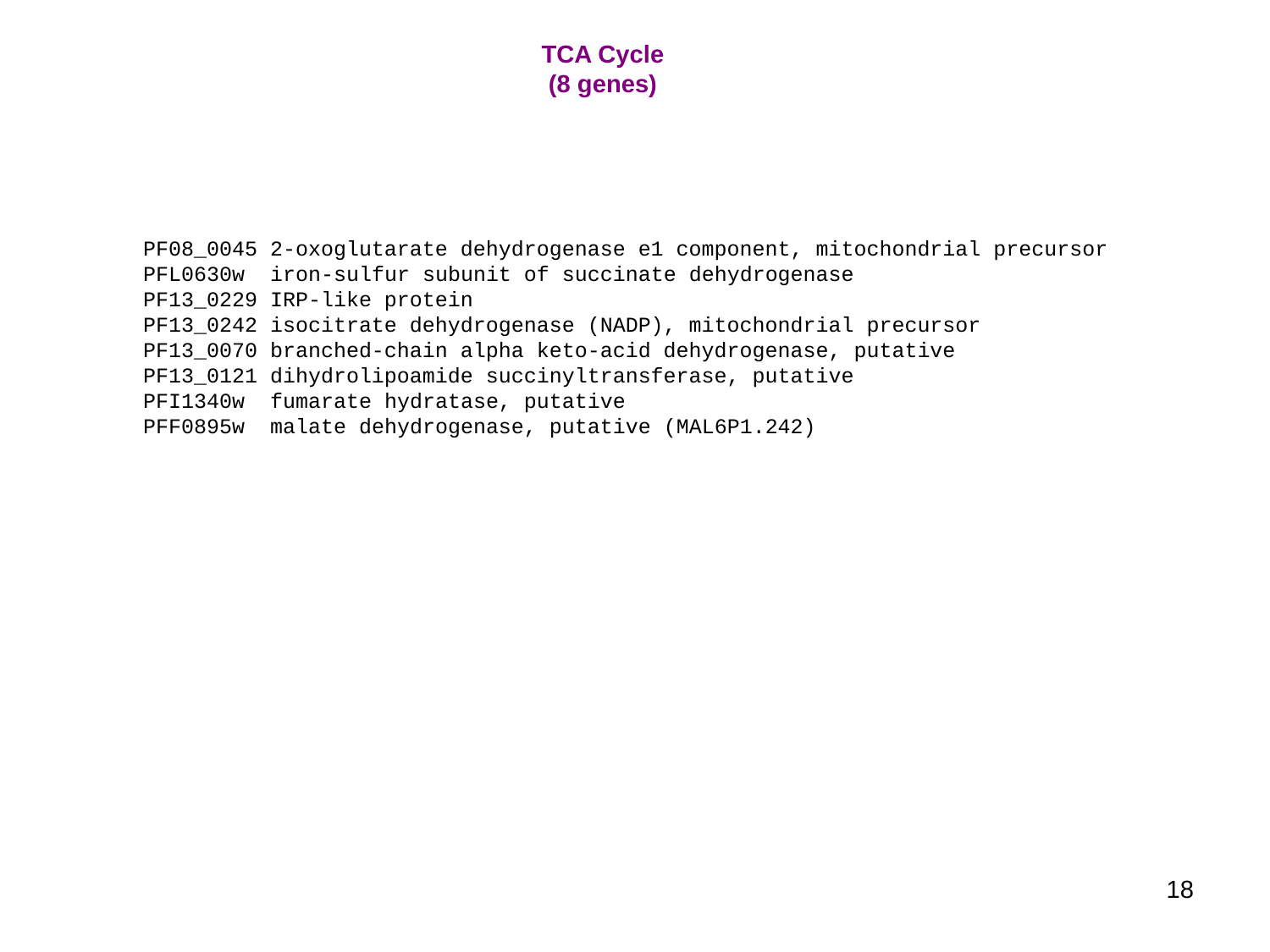

TCA Cycle
(8 genes)
PF08_0045 2-oxoglutarate dehydrogenase e1 component, mitochondrial precursor
PFL0630w 	iron-sulfur subunit of succinate dehydrogenase
PF13_0229 IRP-like protein
PF13_0242 isocitrate dehydrogenase (NADP), mitochondrial precursor
PF13_0070 branched-chain alpha keto-acid dehydrogenase, putative
PF13_0121 dihydrolipoamide succinyltransferase, putative
PFI1340w 	fumarate hydratase, putative
PFF0895w 	malate dehydrogenase, putative (MAL6P1.242)
18

## Slide 19
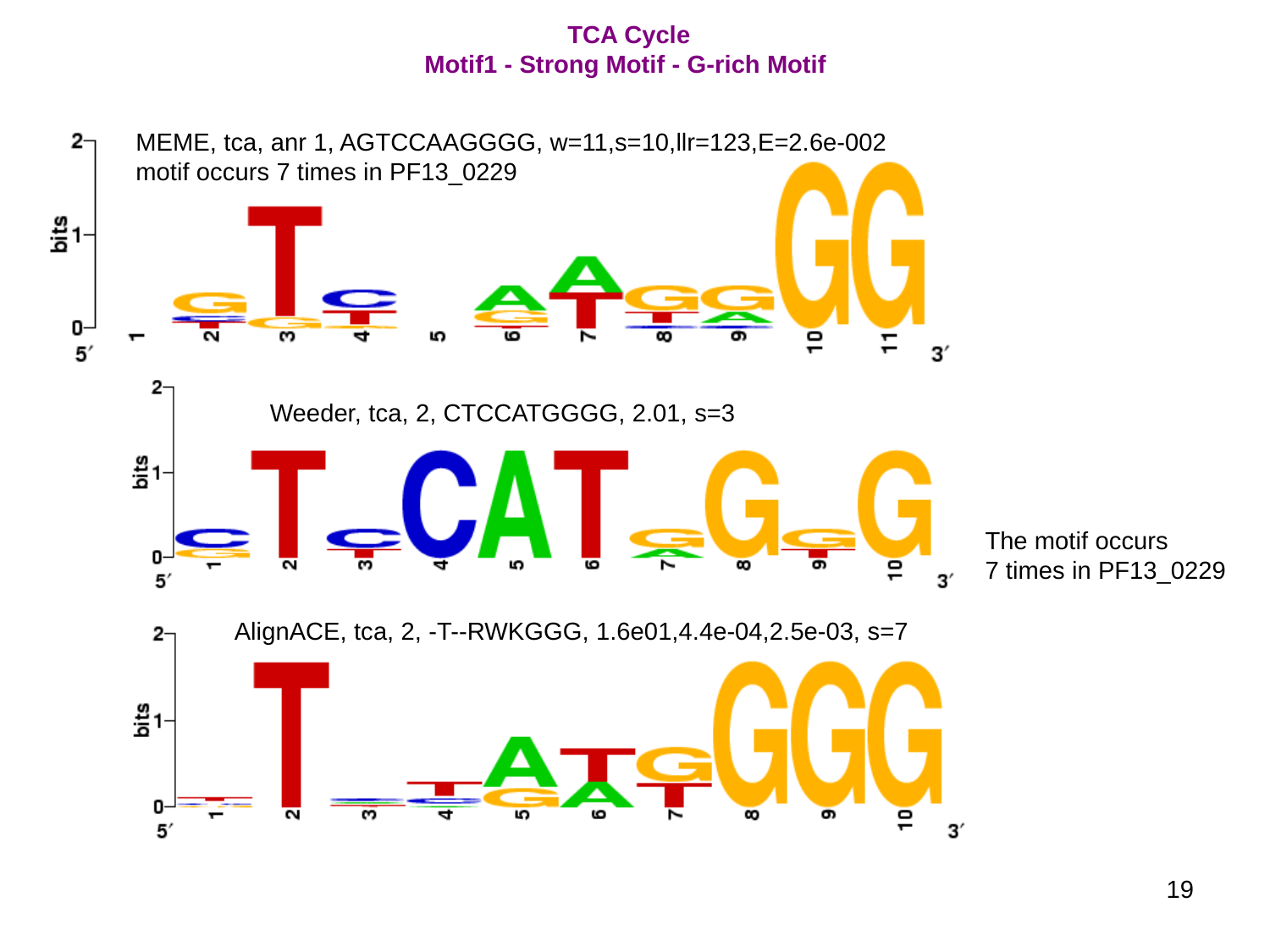

TCA Cycle
Motif1 - Strong Motif - G-rich Motif
MEME, tca, anr 1, AGTCCAAGGGG, w=11,s=10,llr=123,E=2.6e-002
motif occurs 7 times in PF13_0229
Weeder, tca, 2, CTCCATGGGG, 2.01, s=3
The motif occurs
7 times in PF13_0229
AlignACE, tca, 2, -T--RWKGGG, 1.6e01,4.4e-04,2.5e-03, s=7
19

## Slide 20
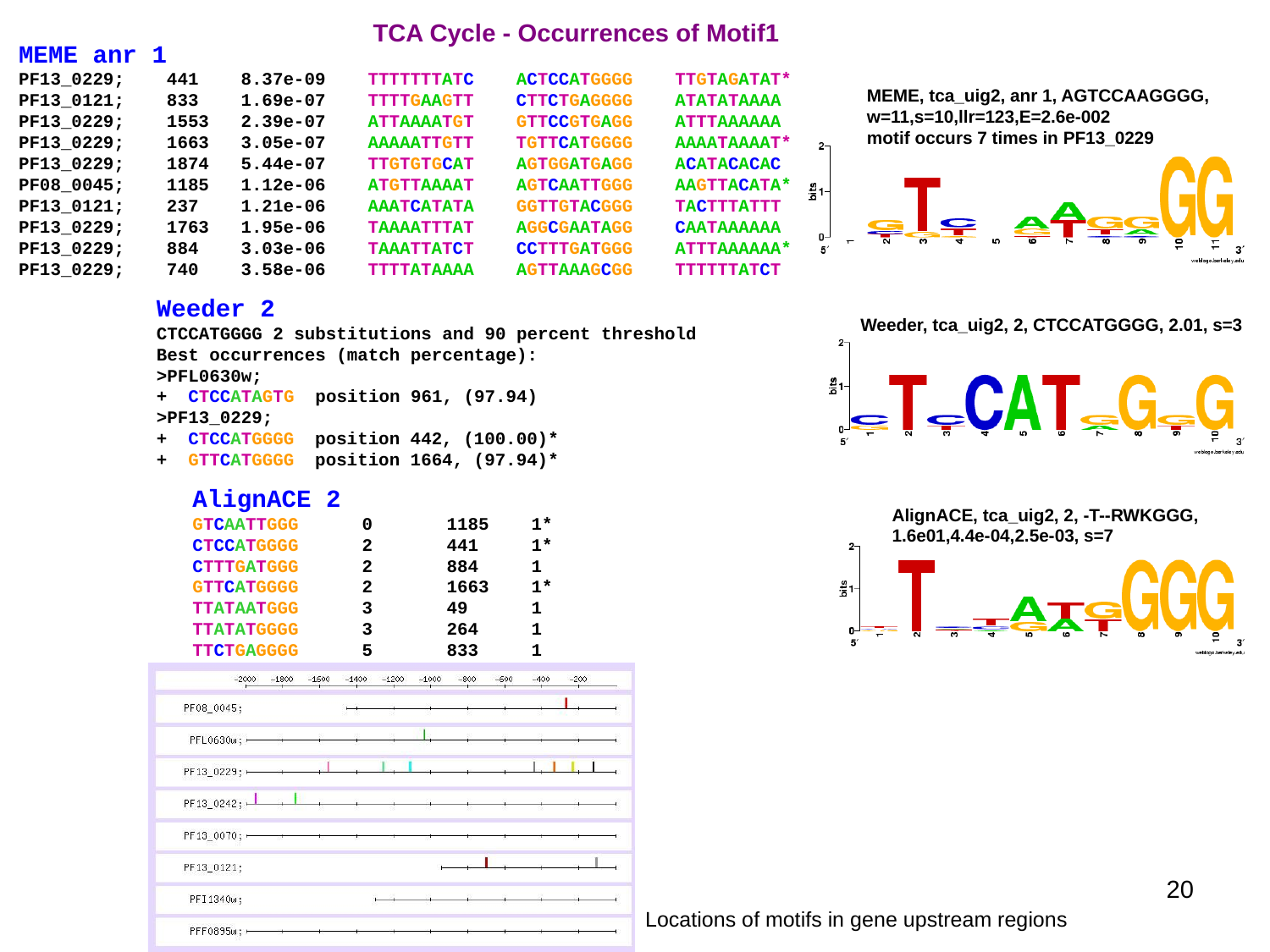

TCA Cycle - Occurrences of Motif1
MEME anr 1
PF13_0229; 441 8.37e-09 TTTTTTTATC ACTCCATGGGG TTGTAGATAT*
PF13_0121; 833 1.69e-07 TTTTGAAGTT CTTCTGAGGGG ATATATAAAA
PF13_0229; 1553 2.39e-07 ATTAAAATGT GTTCCGTGAGG ATTTAAAAAA
PF13_0229; 1663 3.05e-07 AAAAATTGTT TGTTCATGGGG AAAATAAAAT*
PF13_0229; 1874 5.44e-07 TTGTGTGCAT AGTGGATGAGG ACATACACAC
PF08_0045; 1185 1.12e-06 ATGTTAAAAT AGTCAATTGGG AAGTTACATA*
PF13_0121; 237 1.21e-06 AAATCATATA GGTTGTACGGG TACTTTATTT
PF13_0229; 1763 1.95e-06 TAAAATTTAT AGGCGAATAGG CAATAAAAAA
PF13_0229; 884 3.03e-06 TAAATTATCT CCTTTGATGGG ATTTAAAAAA*
PF13_0229; 740 3.58e-06 TTTTATAAAA AGTTAAAGCGG TTTTTTATCT
MEME, tca_uig2, anr 1, AGTCCAAGGGG,
w=11,s=10,llr=123,E=2.6e-002
motif occurs 7 times in PF13_0229
Weeder 2
CTCCATGGGG 2 substitutions and 90 percent threshold
Best occurrences (match percentage):
>PFL0630w;
+ CTCCATAGTG position 961, (97.94)
>PF13_0229;
+ CTCCATGGGG position 442, (100.00)*
+ GTTCATGGGG position 1664, (97.94)*
Weeder, tca_uig2, 2, CTCCATGGGG, 2.01, s=3
AlignACE 2
GTCAATTGGG 0 1185 1*
CTCCATGGGG 2 441 1*
CTTTGATGGG 2 884 1
GTTCATGGGG 2 1663 1*
TTATAATGGG 3 49 1
TTATATGGGG 3 264 1
TTCTGAGGGG 5 833 1
AlignACE, tca_uig2, 2, -T--RWKGGG,
1.6e01,4.4e-04,2.5e-03, s=7
20
Locations of motifs in gene upstream regions

## Slide 21
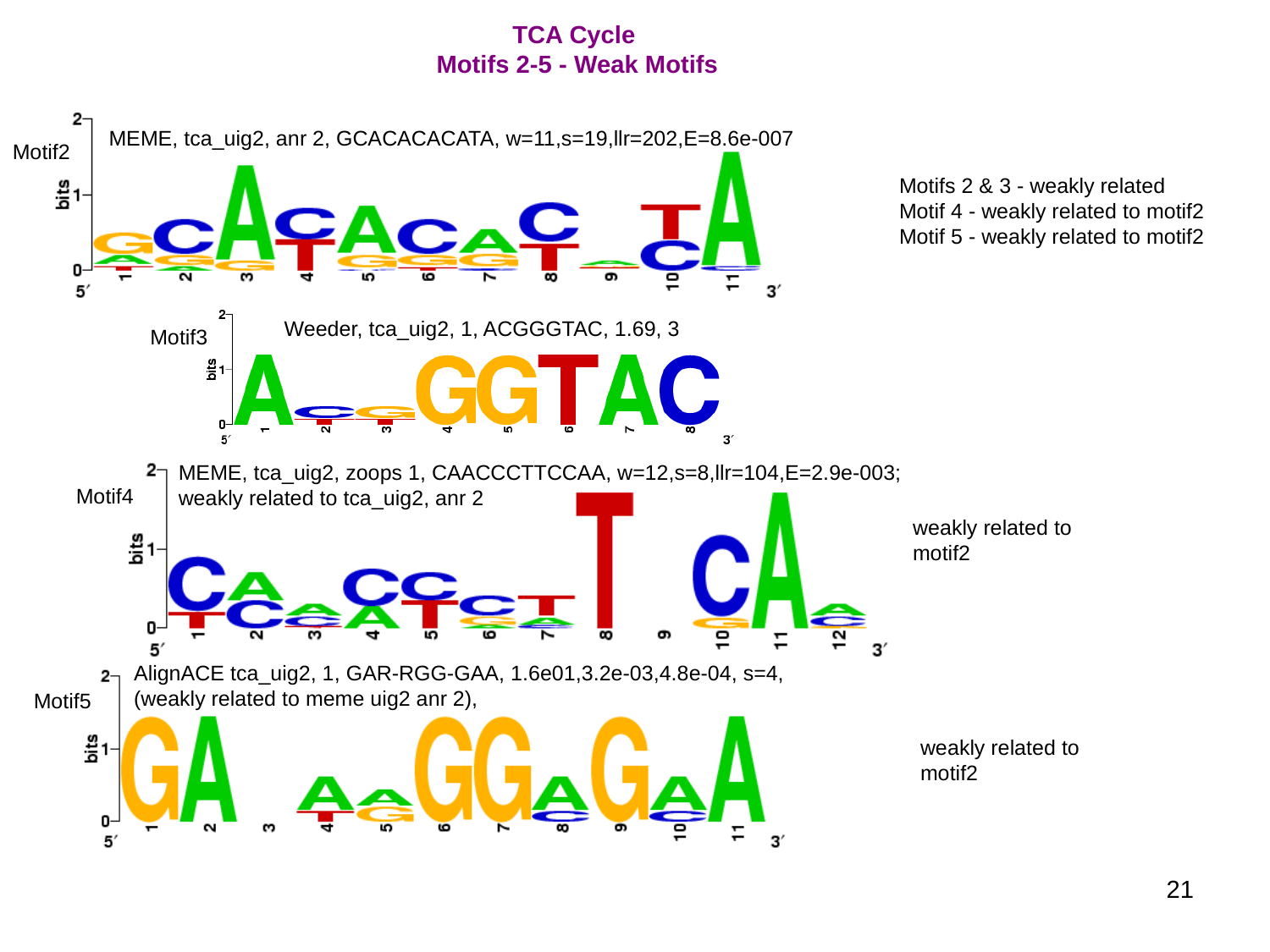

TCA Cycle
Motifs 2-5 - Weak Motifs
MEME, tca_uig2, anr 2, GCACACACATA, w=11,s=19,llr=202,E=8.6e-007
Weeder, tca_uig2, 1, ACGGGTAC, 1.69, 3
MEME, tca_uig2, zoops 1, CAACCCTTCCAA, w=12,s=8,llr=104,E=2.9e-003;
weakly related to tca_uig2, anr 2
weakly related to
motif2
AlignACE tca_uig2, 1, GAR-RGG-GAA, 1.6e01,3.2e-03,4.8e-04, s=4,
(weakly related to meme uig2 anr 2),
weakly related to
motif2
Motif2
Motif3
Motif4
Motif5
Motifs 2 & 3 - weakly related
Motif 4 - weakly related to motif2
Motif 5 - weakly related to motif2
21

## Slide 22
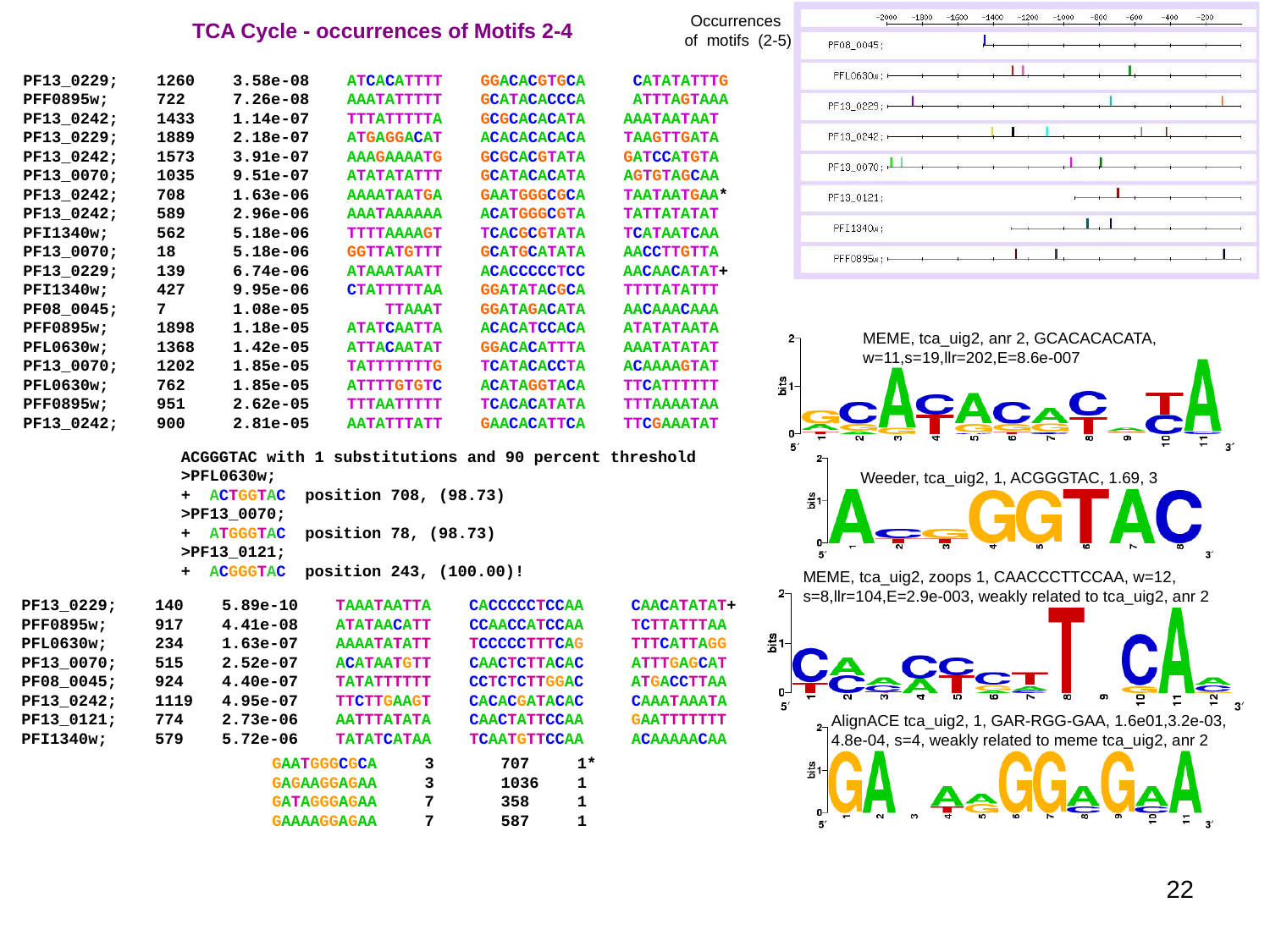

Occurrences
of motifs (2-5)
TCA Cycle - occurrences of Motifs 2-4
PF13_0229; 1260 3.58e-08 ATCACATTTT GGACACGTGCA CATATATTTG
PFF0895w; 722 7.26e-08 AAATATTTTT GCATACACCCA ATTTAGTAAA
PF13_0242; 1433 1.14e-07 TTTATTTTTA GCGCACACATA AAATAATAAT
PF13_0229; 1889 2.18e-07 ATGAGGACAT ACACACACACA TAAGTTGATA
PF13_0242; 1573 3.91e-07 AAAGAAAATG GCGCACGTATA GATCCATGTA
PF13_0070; 1035 9.51e-07 ATATATATTT GCATACACATA AGTGTAGCAA
PF13_0242; 708 1.63e-06 AAAATAATGA GAATGGGCGCA TAATAATGAA*
PF13_0242; 589 2.96e-06 AAATAAAAAA ACATGGGCGTA TATTATATAT
PFI1340w; 562 5.18e-06 TTTTAAAAGT TCACGCGTATA TCATAATCAA
PF13_0070; 18 5.18e-06 GGTTATGTTT GCATGCATATA AACCTTGTTA
PF13_0229; 139 6.74e-06 ATAAATAATT ACACCCCCTCC AACAACATAT+
PFI1340w; 427 9.95e-06 CTATTTTTAA GGATATACGCA TTTTATATTT
PF08_0045; 7 1.08e-05 TTAAAT GGATAGACATA AACAAACAAA
PFF0895w; 1898 1.18e-05 ATATCAATTA ACACATCCACA ATATATAATA
PFL0630w; 1368 1.42e-05 ATTACAATAT GGACACATTTA AAATATATAT
PF13_0070; 1202 1.85e-05 TATTTTTTTG TCATACACCTA ACAAAAGTAT
PFL0630w; 762 1.85e-05 ATTTTGTGTC ACATAGGTACA TTCATTTTTT
PFF0895w; 951 2.62e-05 TTTAATTTTT TCACACATATA TTTAAAATAA
PF13_0242; 900 2.81e-05 AATATTTATT GAACACATTCA TTCGAAATAT
MEME, tca_uig2, anr 2, GCACACACATA,
w=11,s=19,llr=202,E=8.6e-007
Weeder, tca_uig2, 1, ACGGGTAC, 1.69, 3
MEME, tca_uig2, zoops 1, CAACCCTTCCAA, w=12,
s=8,llr=104,E=2.9e-003, weakly related to tca_uig2, anr 2
AlignACE tca_uig2, 1, GAR-RGG-GAA, 1.6e01,3.2e-03,
4.8e-04, s=4, weakly related to meme tca_uig2, anr 2
ACGGGTAC with 1 substitutions and 90 percent threshold
>PFL0630w;
+ ACTGGTAC position 708, (98.73)
>PF13_0070;
+ ATGGGTAC position 78, (98.73)
>PF13_0121;
+ ACGGGTAC position 243, (100.00)!
PF13_0229; 140 5.89e-10 TAAATAATTA CACCCCCTCCAA CAACATATAT+
PFF0895w; 917 4.41e-08 ATATAACATT CCAACCATCCAA TCTTATTTAA
PFL0630w; 234 1.63e-07 AAAATATATT TCCCCCTTTCAG TTTCATTAGG
PF13_0070; 515 2.52e-07 ACATAATGTT CAACTCTTACAC ATTTGAGCAT
PF08_0045; 924 4.40e-07 TATATTTTTT CCTCTCTTGGAC ATGACCTTAA
PF13_0242; 1119 4.95e-07 TTCTTGAAGT CACACGATACAC CAAATAAATA
PF13_0121; 774 2.73e-06 AATTTATATA CAACTATTCCAA GAATTTTTTT
PFI1340w; 579 5.72e-06 TATATCATAA TCAATGTTCCAA ACAAAAACAA
GAATGGGCGCA 3 707 1*
GAGAAGGAGAA 3 1036 1
GATAGGGAGAA 7 358 1
GAAAAGGAGAA 7 587 1
22

## Slide 23
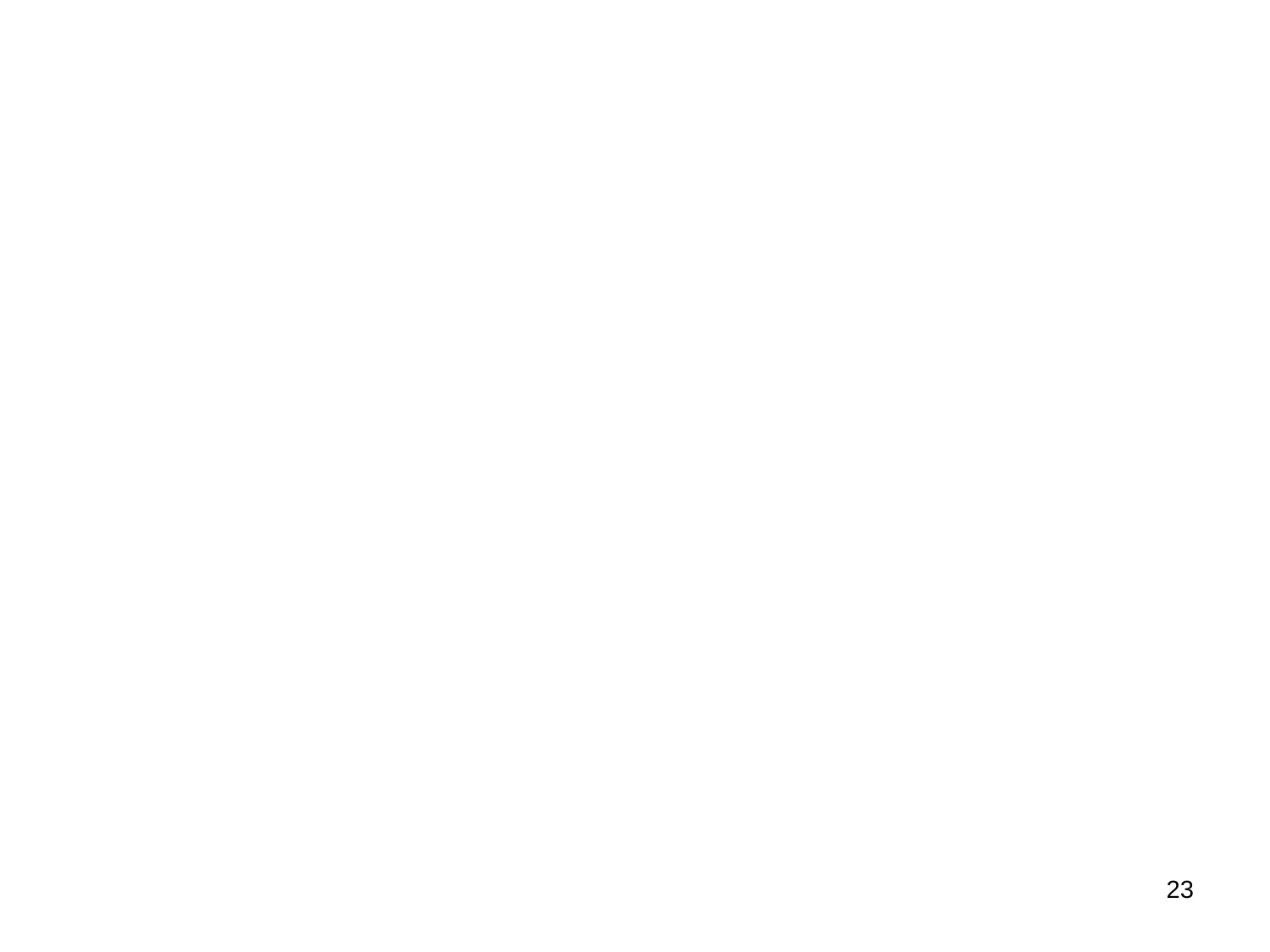

23

## Slide 24
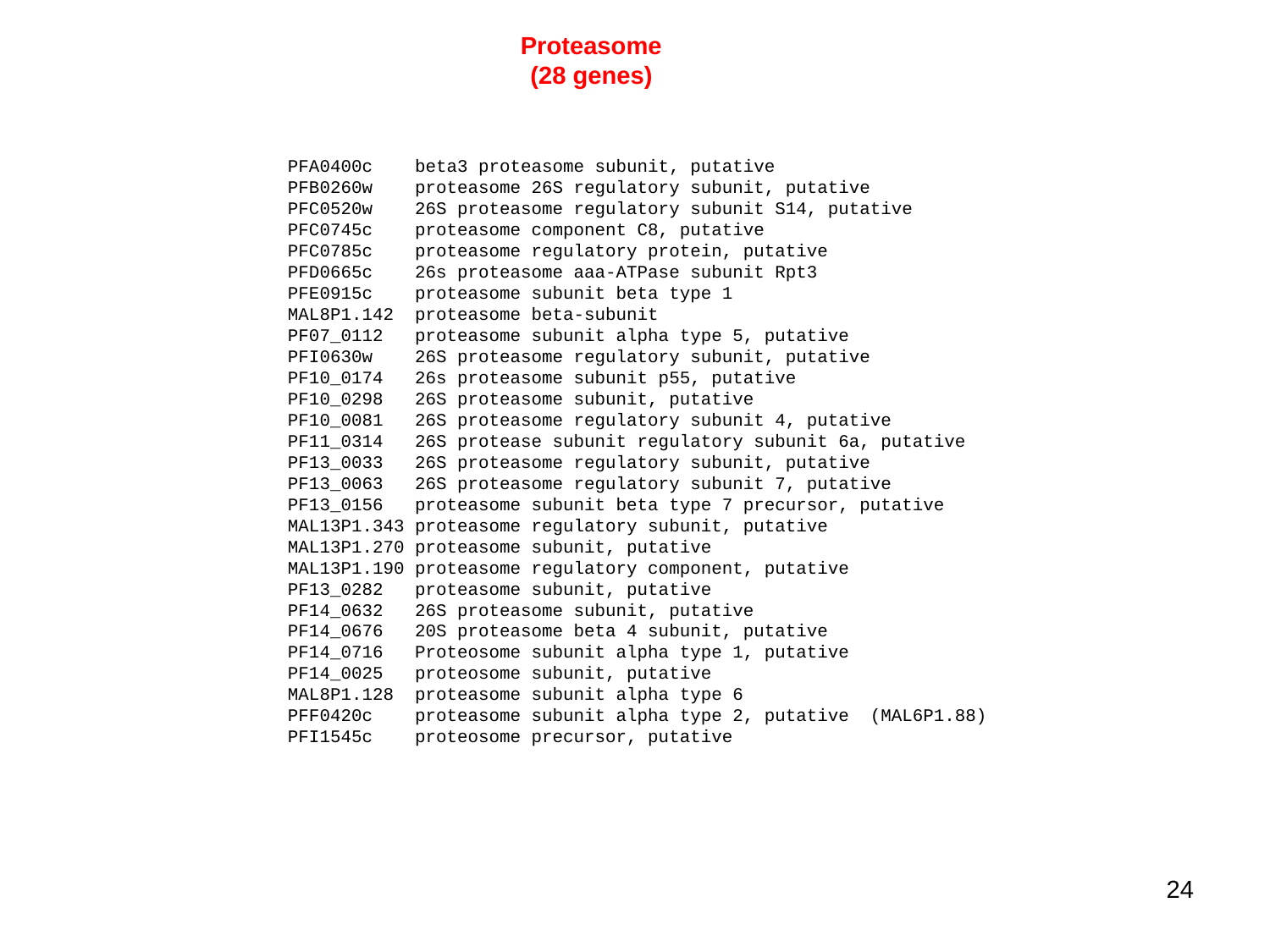

Proteasome
(28 genes)
PFA0400c beta3 proteasome subunit, putative
PFB0260w proteasome 26S regulatory subunit, putative
PFC0520w 26S proteasome regulatory subunit S14, putative
PFC0745c proteasome component C8, putative
PFC0785c proteasome regulatory protein, putative
PFD0665c 26s proteasome aaa-ATPase subunit Rpt3
PFE0915c proteasome subunit beta type 1
MAL8P1.142 proteasome beta-subunit
PF07_0112 proteasome subunit alpha type 5, putative
PFI0630w 26S proteasome regulatory subunit, putative
PF10_0174 26s proteasome subunit p55, putative
PF10_0298 26S proteasome subunit, putative
PF10_0081 26S proteasome regulatory subunit 4, putative
PF11_0314 26S protease subunit regulatory subunit 6a, putative
PF13_0033 26S proteasome regulatory subunit, putative
PF13_0063 26S proteasome regulatory subunit 7, putative
PF13_0156 proteasome subunit beta type 7 precursor, putative
MAL13P1.343 proteasome regulatory subunit, putative
MAL13P1.270 proteasome subunit, putative
MAL13P1.190 proteasome regulatory component, putative
PF13_0282 proteasome subunit, putative
PF14_0632 26S proteasome subunit, putative
PF14_0676 20S proteasome beta 4 subunit, putative
PF14_0716 Proteosome subunit alpha type 1, putative
PF14_0025 proteosome subunit, putative
MAL8P1.128 proteasome subunit alpha type 6
PFF0420c proteasome subunit alpha type 2, putative (MAL6P1.88)
PFI1545c proteosome precursor, putative
24

## Slide 25
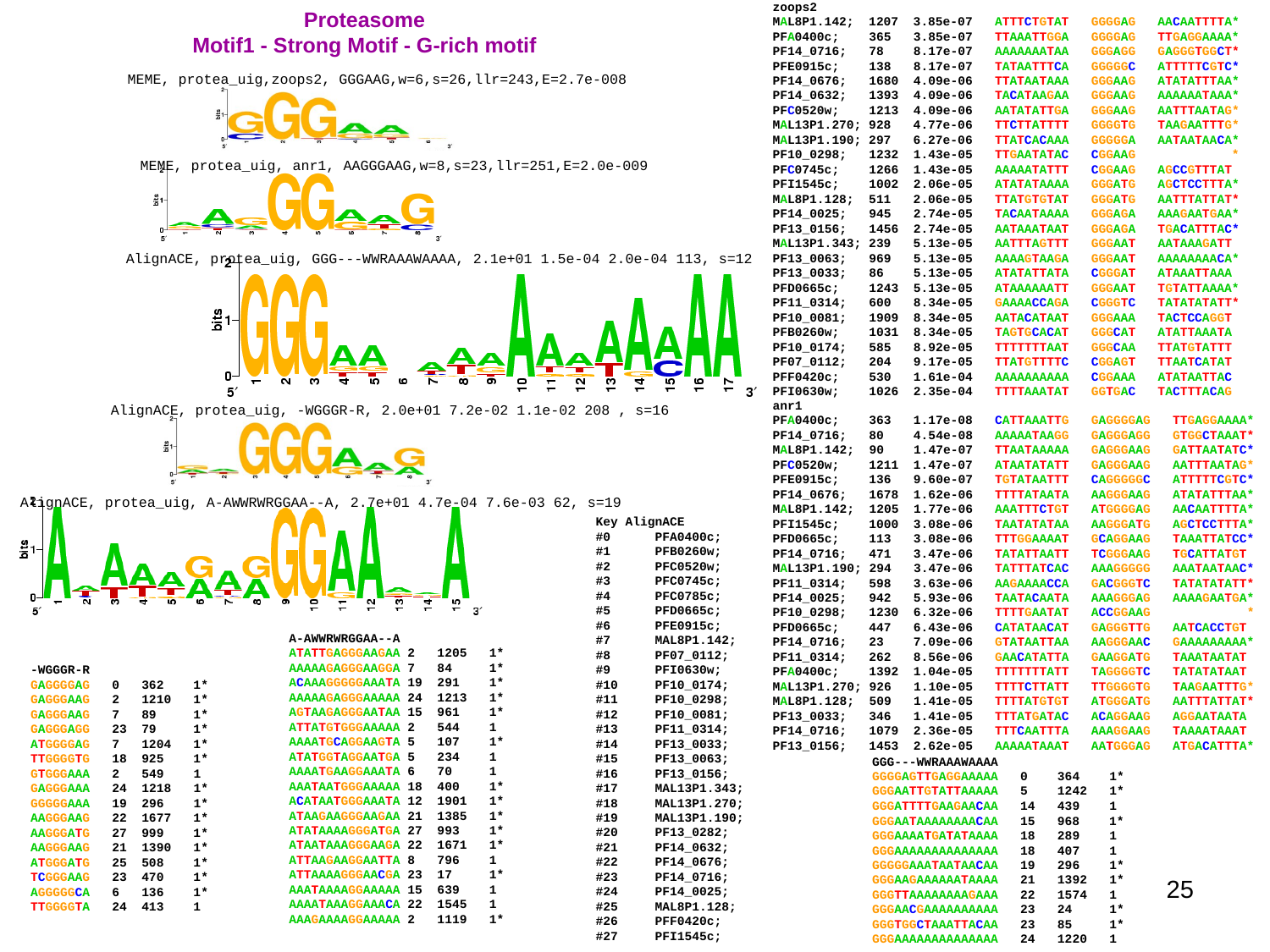

Proteasome
Motif1 - Strong Motif - G-rich motif
zoops2
MAL8P1.142; 1207 3.85e-07 ATTTCTGTAT GGGGAG AACAATTTTA*
PFA0400c; 365 3.85e-07 TTAAATTGGA GGGGAG TTGAGGAAAA*
PF14_0716; 78 8.17e-07 AAAAAAATAA GGGAGG GAGGGTGGCT*
PFE0915c; 138 8.17e-07 TATAATTTCA GGGGGC ATTTTTCGTC*
PF14_0676; 1680 4.09e-06 TTATAATAAA GGGAAG ATATATTTAA*
PF14_0632; 1393 4.09e-06 TACATAAGAA GGGAAG AAAAAATAAA*
PFC0520w; 1213 4.09e-06 AATATATTGA GGGAAG AATTTAATAG*
MAL13P1.270; 928 4.77e-06 TTCTTATTTT GGGGTG TAAGAATTTG*
MAL13P1.190; 297 6.27e-06 TTATCACAAA GGGGGA AATAATAACA*
PF10_0298; 1232 1.43e-05 TTGAATATAC CGGAAG *
PFC0745c; 1266 1.43e-05 AAAAATATTT CGGAAG AGCCGTTTAT
PFI1545c; 1002 2.06e-05 ATATATAAAA GGGATG AGCTCCTTTA*
MAL8P1.128; 511 2.06e-05 TTATGTGTAT GGGATG AATTTATTAT*
PF14_0025; 945 2.74e-05 TACAATAAAA GGGAGA AAAGAATGAA*
PF13_0156; 1456 2.74e-05 AATAAATAAT GGGAGA TGACATTTAC*
MAL13P1.343; 239 5.13e-05 AATTTAGTTT GGGAAT AATAAAGATT
PF13_0063; 969 5.13e-05 AAAAGTAAGA GGGAAT AAAAAAAACA*
PF13_0033; 86 5.13e-05 ATATATTATA CGGGAT ATAAATTAAA
PFD0665c; 1243 5.13e-05 ATAAAAAATT GGGAAT TGTATTAAAA*
PF11_0314; 600 8.34e-05 GAAAACCAGA CGGGTC TATATATATT*
PF10_0081; 1909 8.34e-05 AATACATAAT GGGAAA TACTCCAGGT
PFB0260w; 1031 8.34e-05 TAGTGCACAT GGGCAT ATATTAAATA
PF10_0174; 585 8.92e-05 TTTTTTTAAT GGGCAA TTATGTATTT
PF07_0112; 204 9.17e-05 TTATGTTTTC CGGAGT TTAATCATAT
PFF0420c; 530 1.61e-04 AAAAAAAAAA CGGAAA ATATAATTAC
PFI0630w; 1026 2.35e-04 TTTTAAATAT GGTGAC TACTTTACAG
MEME, protea_uig,zoops2, GGGAAG,w=6,s=26,llr=243,E=2.7e-008
MEME, protea_uig, anr1, AAGGGAAG,w=8,s=23,llr=251,E=2.0e-009
AlignACE, protea_uig, GGG---WWRAAAWAAAA, 2.1e+01 1.5e-04 2.0e-04 113, s=12
AlignACE, protea_uig, -WGGGR-R, 2.0e+01 7.2e-02 1.1e-02 208 , s=16
AlignACE, protea_uig, A-AWWRWRGGAA--A, 2.7e+01 4.7e-04 7.6e-03 62, s=19
anr1
PFA0400c; 363 1.17e-08 CATTAAATTG GAGGGGAG TTGAGGAAAA*
PF14_0716; 80 4.54e-08 AAAAATAAGG GAGGGAGG GTGGCTAAAT*
MAL8P1.142; 90 1.47e-07 TTAATAAAAA GAGGGAAG GATTAATATC*
PFC0520w; 1211 1.47e-07 ATAATATATT GAGGGAAG AATTTAATAG*
PFE0915c; 136 9.60e-07 TGTATAATTT CAGGGGGC ATTTTTCGTC*
PF14_0676; 1678 1.62e-06 TTTTATAATA AAGGGAAG ATATATTTAA*
MAL8P1.142; 1205 1.77e-06 AAATTTCTGT ATGGGGAG AACAATTTTA*
PFI1545c; 1000 3.08e-06 TAATATATAA AAGGGATG AGCTCCTTTA*
PFD0665c; 113 3.08e-06 TTTGGAAAAT GCAGGAAG TAAATTATCC*
PF14_0716; 471 3.47e-06 TATATTAATT TCGGGAAG TGCATTATGT
MAL13P1.190; 294 3.47e-06 TATTTATCAC AAAGGGGG AAATAATAAC*
PF11_0314; 598 3.63e-06 AAGAAAACCA GACGGGTC TATATATATT*
PF14_0025; 942 5.93e-06 TAATACAATA AAAGGGAG AAAAGAATGA*
PF10_0298; 1230 6.32e-06 TTTTGAATAT ACCGGAAG *
PFD0665c; 447 6.43e-06 CATATAACAT GAGGGTTG AATCACCTGT
PF14_0716; 23 7.09e-06 GTATAATTAA AAGGGAAC GAAAAAAAAA*
PF11_0314; 262 8.56e-06 GAACATATTA GAAGGATG TAAATAATAT
PFA0400c; 1392 1.04e-05 TTTTTTTATT TAGGGGTC TATATATAAT
MAL13P1.270; 926 1.10e-05 TTTTCTTATT TTGGGGTG TAAGAATTTG*
MAL8P1.128; 509 1.41e-05 TTTTATGTGT ATGGGATG AATTTATTAT*
PF13_0033; 346 1.41e-05 TTTATGATAC ACAGGAAG AGGAATAATA
PF14_0716; 1079 2.36e-05 TTTCAATTTA AAAGGAAG TAAAATAAAT
PF13_0156; 1453 2.62e-05 AAAAATAAAT AATGGGAG ATGACATTTA*
Key AlignACE
#0 PFA0400c;
#1 PFB0260w;
#2 PFC0520w;
#3 PFC0745c;
#4 PFC0785c;
#5 PFD0665c;
#6 PFE0915c;
#7 MAL8P1.142;
#8 PF07_0112;
#9 PFI0630w;
#10 PF10_0174;
#11 PF10_0298;
#12 PF10_0081;
#13 PF11_0314;
#14 PF13_0033;
#15 PF13_0063;
#16 PF13_0156;
#17 MAL13P1.343;
#18 MAL13P1.270;
#19 MAL13P1.190;
#20 PF13_0282;
#21 PF14_0632;
#22 PF14_0676;
#23 PF14_0716;
#24 PF14_0025;
#25 MAL8P1.128;
#26 PFF0420c;
#27 PFI1545c;
A-AWWRWRGGAA--A
ATATTGAGGGAAGAA 2 1205 1*
AAAAAGAGGGAAGGA 7 84 1*
ACAAAGGGGGAAATA 19 291 1*
AAAAAGAGGGAAAAA 24 1213 1*
AGTAAGAGGGAATAA 15 961 1*
ATTATGTGGGAAAAA 2 544 1
AAAATGCAGGAAGTA 5 107 1*
ATATGGTAGGAATGA 5 234 1
AAAATGAAGGAAATA 6 70 1
AAATAATGGGAAAAA 18 400 1*
ACATAATGGGAAATA 12 1901 1*
ATAAGAAGGGAAGAA 21 1385 1*
ATATAAAAGGGATGA 27 993 1*
ATAATAAAGGGAAGA 22 1671 1*
ATTAAGAAGGAATTA 8 796 1
ATTAAAAGGGAACGA 23 17 1*
AAATAAAAGGAAAAA 15 639 1
AAAATAAAGGAAACA 22 1545 1
AAAGAAAAGGAAAAA 2 1119 1*
-WGGGR-R
GAGGGGAG 0 362 1*
GAGGGAAG 2 1210 1*
GAGGGAAG 7 89 1*
GAGGGAGG 23 79 1*
ATGGGGAG 7 1204 1*
TTGGGGTG 18 925 1*
GTGGGAAA 2 549 1
GAGGGAAA 24 1218 1*
GGGGGAAA 19 296 1*
AAGGGAAG 22 1677 1*
AAGGGATG 27 999 1*
AAGGGAAG 21 1390 1*
ATGGGATG 25 508 1*
TCGGGAAG 23 470 1*
AGGGGGCA 6 136 1*
TTGGGGTA 24 413 1
GGG---WWRAAAWAAAA
GGGGAGTTGAGGAAAAA 0 364 1*
GGGAATTGTATTAAAAA 5 1242 1*
GGGATTTTGAAGAACAA 14 439 1
GGGAATAAAAAAAACAA 15 968 1*
GGGAAAATGATATAAAA 18 289 1
GGGAAAAAAAAAAAAAA 18 407 1
GGGGGAAATAATAACAA 19 296 1*
GGGAAGAAAAAATAAAA 21 1392 1*
GGGTTAAAAAAAAGAAA 22 1574 1
GGGAACGAAAAAAAAAA 23 24 1*
GGGTGGCTAAATTACAA 23 85 1*
GGGAAAAAAAAAAAAAA 24 1220 1
25

## Slide 26
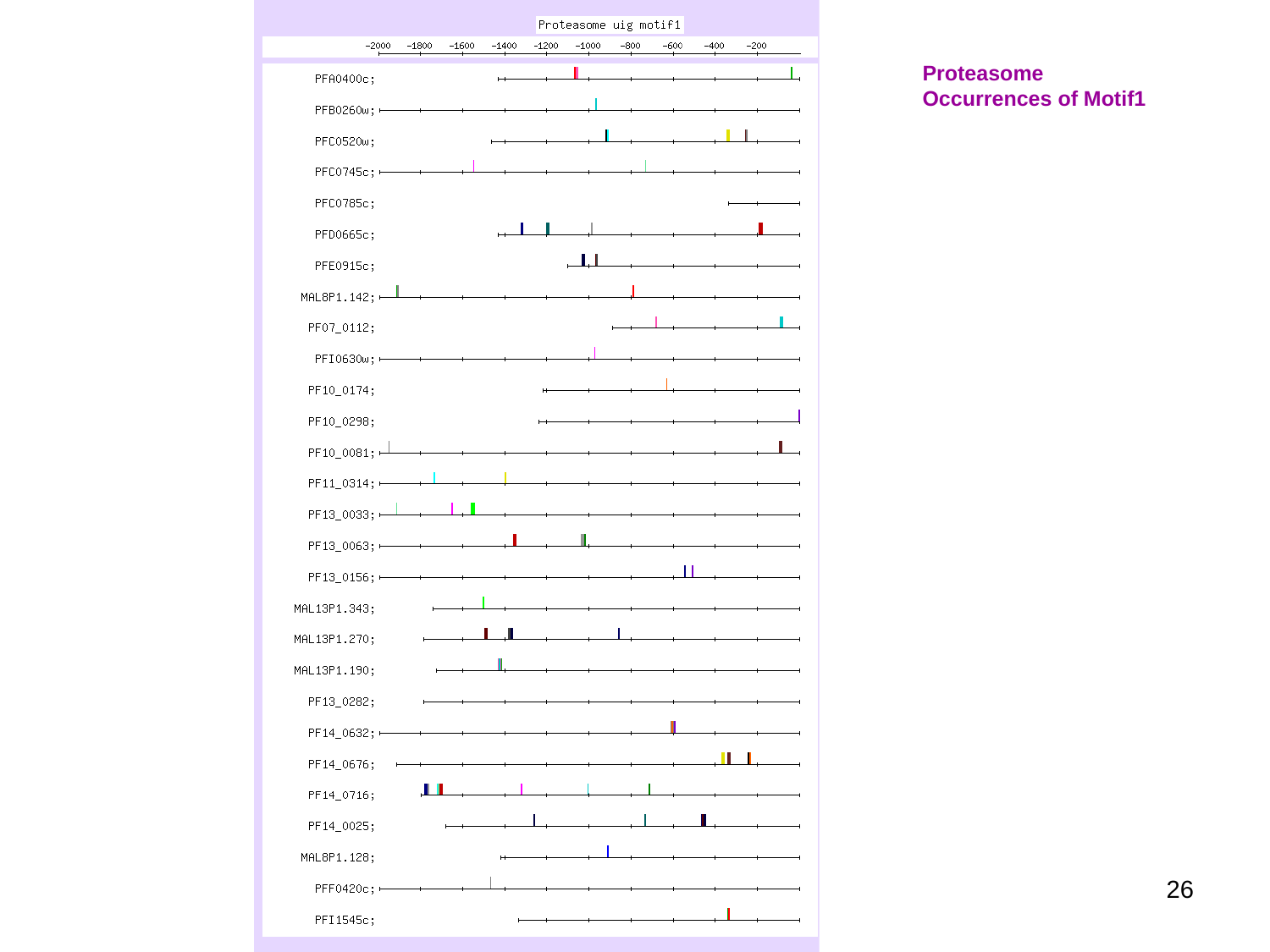

Proteasome
Occurrences of Motif1
26

## Slide 27
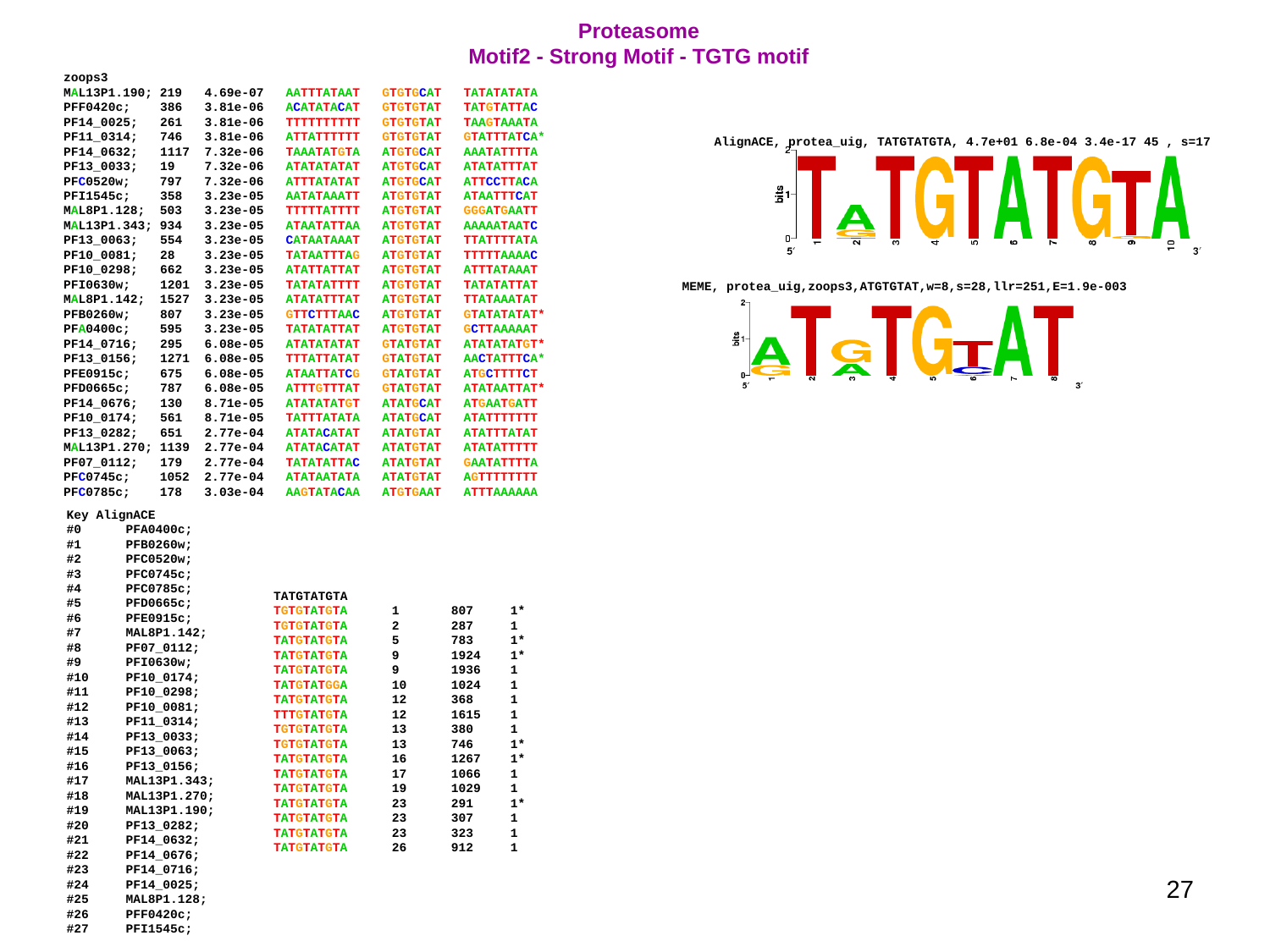

Proteasome
Motif2 - Strong Motif - TGTG motif
zoops3
MAL13P1.190; 219 4.69e-07 AATTTATAAT GTGTGCAT TATATATATA
PFF0420c; 386 3.81e-06 ACATATACAT GTGTGTAT TATGTATTAC
PF14_0025; 261 3.81e-06 TTTTTTTTTT GTGTGTAT TAAGTAAATA
PF11_0314; 746 3.81e-06 ATTATTTTTT GTGTGTAT GTATTTATCA*
PF14_0632; 1117 7.32e-06 TAAATATGTA ATGTGCAT AAATATTTTA
PF13_0033; 19 7.32e-06 ATATATATAT ATGTGCAT ATATATTTAT
PFC0520w; 797 7.32e-06 ATTTATATAT ATGTGCAT ATTCCTTACA
PFI1545c; 358 3.23e-05 AATATAAATT ATGTGTAT ATAATTTCAT
MAL8P1.128; 503 3.23e-05 TTTTTATTTT ATGTGTAT GGGATGAATT
MAL13P1.343; 934 3.23e-05 ATAATATTAA ATGTGTAT AAAAATAATC
PF13_0063; 554 3.23e-05 CATAATAAAT ATGTGTAT TTATTTTATA
PF10_0081; 28 3.23e-05 TATAATTTAG ATGTGTAT TTTTTAAAAC
PF10_0298; 662 3.23e-05 ATATTATTAT ATGTGTAT ATTTATAAAT
PFI0630w; 1201 3.23e-05 TATATATTTT ATGTGTAT TATATATTAT
MAL8P1.142; 1527 3.23e-05 ATATATTTAT ATGTGTAT TTATAAATAT
PFB0260w; 807 3.23e-05 GTTCTTTAAC ATGTGTAT GTATATATAT*
PFA0400c; 595 3.23e-05 TATATATTAT ATGTGTAT GCTTAAAAAT
PF14_0716; 295 6.08e-05 ATATATATAT GTATGTAT ATATATATGT*
PF13_0156; 1271 6.08e-05 TTTATTATAT GTATGTAT AACTATTTCA*
PFE0915c; 675 6.08e-05 ATAATTATCG GTATGTAT ATGCTTTTCT
PFD0665c; 787 6.08e-05 ATTTGTTTAT GTATGTAT ATATAATTAT*
PF14_0676; 130 8.71e-05 ATATATATGT ATATGCAT ATGAATGATT
PF10_0174; 561 8.71e-05 TATTTATATA ATATGCAT ATATTTTTTT
PF13_0282; 651 2.77e-04 ATATACATAT ATATGTAT ATATTTATAT
MAL13P1.270; 1139 2.77e-04 ATATACATAT ATATGTAT ATATATTTTT
PF07_0112; 179 2.77e-04 TATATATTAC ATATGTAT GAATATTTTA
PFC0745c; 1052 2.77e-04 ATATAATATA ATATGTAT AGTTTTTTTT
PFC0785c; 178 3.03e-04 AAGTATACAA ATGTGAAT ATTTAAAAAA
AlignACE, protea_uig, TATGTATGTA, 4.7e+01 6.8e-04 3.4e-17 45 , s=17
MEME, protea_uig,zoops3,ATGTGTAT,w=8,s=28,llr=251,E=1.9e-003
Key AlignACE
#0 PFA0400c;
#1 PFB0260w;
#2 PFC0520w;
#3 PFC0745c;
#4 PFC0785c;
#5 PFD0665c;
#6 PFE0915c;
#7 MAL8P1.142;
#8 PF07_0112;
#9 PFI0630w;
#10 PF10_0174;
#11 PF10_0298;
#12 PF10_0081;
#13 PF11_0314;
#14 PF13_0033;
#15 PF13_0063;
#16 PF13_0156;
#17 MAL13P1.343;
#18 MAL13P1.270;
#19 MAL13P1.190;
#20 PF13_0282;
#21 PF14_0632;
#22 PF14_0676;
#23 PF14_0716;
#24 PF14_0025;
#25 MAL8P1.128;
#26 PFF0420c;
#27 PFI1545c;
TATGTATGTA
TGTGTATGTA 1 807 1*
TGTGTATGTA 2 287 1
TATGTATGTA 5 783 1*
TATGTATGTA 9 1924 1*
TATGTATGTA 9 1936 1
TATGTATGGA 10 1024 1
TATGTATGTA 12 368 1
TTTGTATGTA 12 1615 1
TGTGTATGTA 13 380 1
TGTGTATGTA 13 746 1*
TATGTATGTA 16 1267 1*
TATGTATGTA 17 1066 1
TATGTATGTA 19 1029 1
TATGTATGTA 23 291 1*
TATGTATGTA 23 307 1
TATGTATGTA 23 323 1
TATGTATGTA 26 912 1
27

## Slide 28
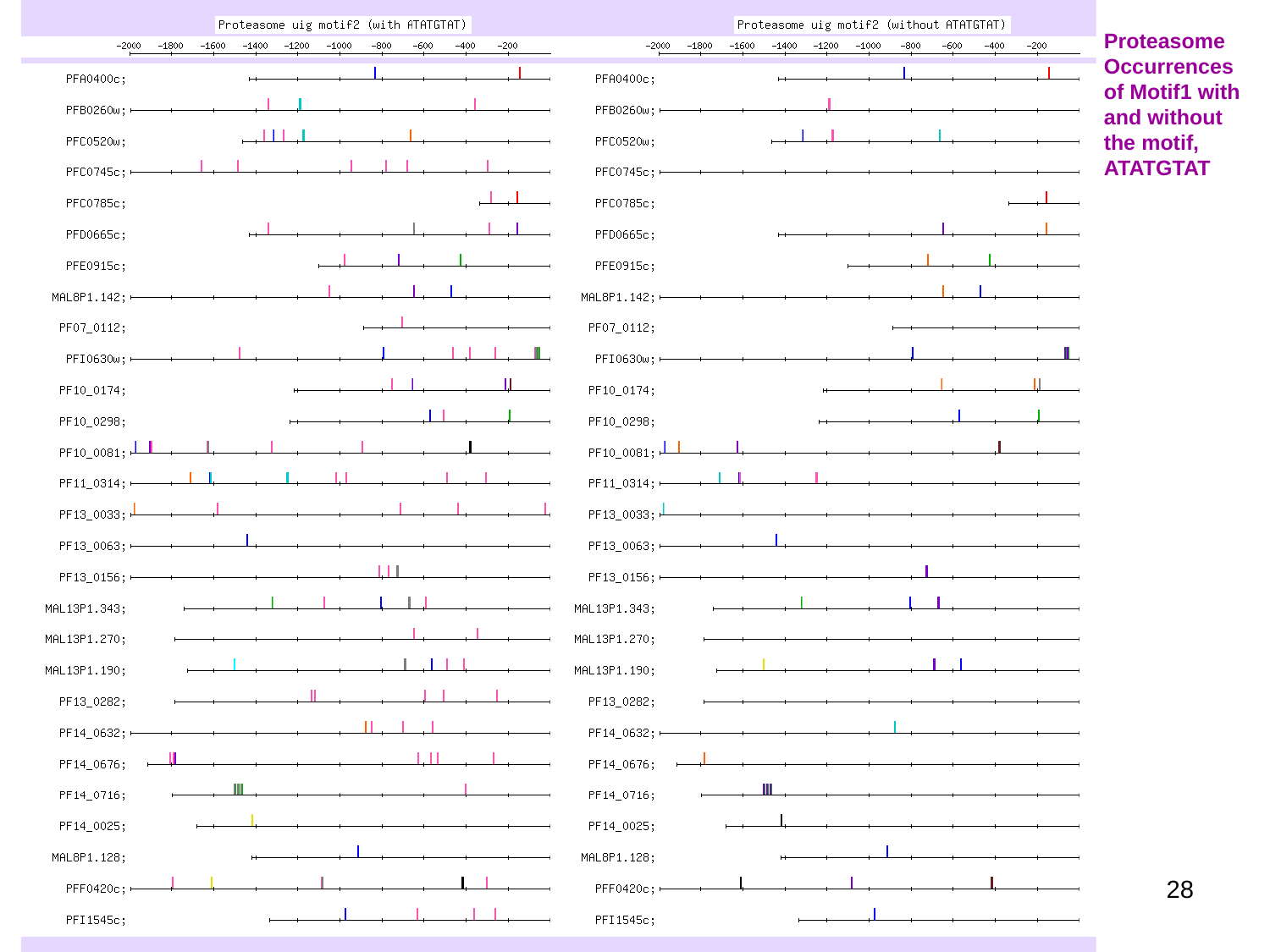

Proteasome
Occurrences
of Motif1 with
and without
the motif,
ATATGTAT
28

## Slide 29
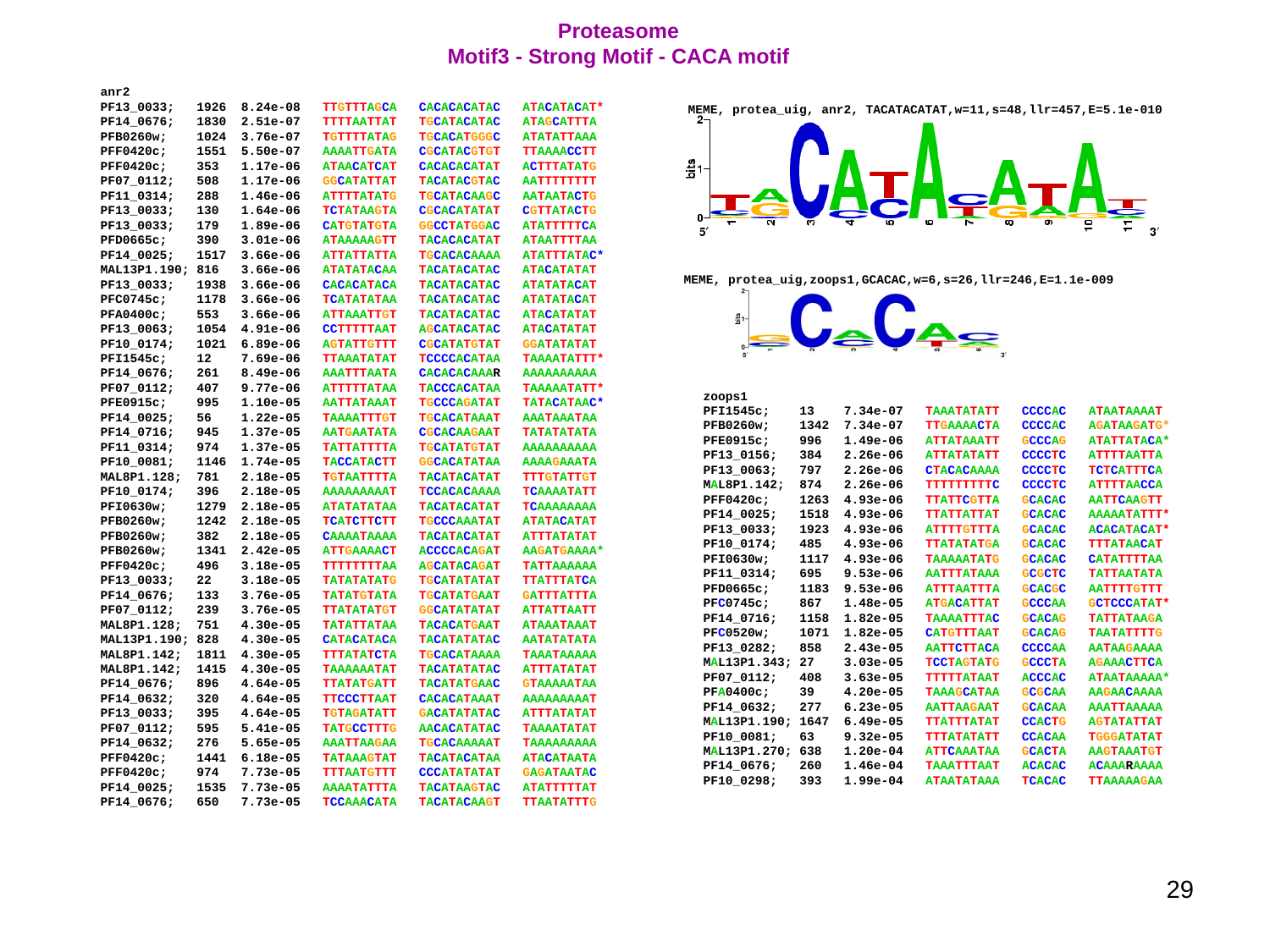

Proteasome
Motif3 - Strong Motif - CACA motif
anr2
PF13_0033; 1926 8.24e-08 TTGTTTAGCA CACACACATAC ATACATACAT*
PF14_0676; 1830 2.51e-07 TTTTAATTAT TGCATACATAC ATAGCATTTA
PFB0260w; 1024 3.76e-07 TGTTTTATAG TGCACATGGGC ATATATTAAA
PFF0420c; 1551 5.50e-07 AAAATTGATA CGCATACGTGT TTAAAACCTT
PFF0420c; 353 1.17e-06 ATAACATCAT CACACACATAT ACTTTATATG
PF07_0112; 508 1.17e-06 GGCATATTAT TACATACGTAC AATTTTTTTT
PF11_0314; 288 1.46e-06 ATTTTATATG TGCATACAAGC AATAATACTG
PF13_0033; 130 1.64e-06 TCTATAAGTA CGCACATATAT CGTTATACTG
PF13_0033; 179 1.89e-06 CATGTATGTA GGCCTATGGAC ATATTTTTCA
PFD0665c; 390 3.01e-06 ATAAAAAGTT TACACACATAT ATAATTTTAA
PF14_0025; 1517 3.66e-06 ATTATTATTA TGCACACAAAA ATATTTATAC*
MAL13P1.190; 816 3.66e-06 ATATATACAA TACATACATAC ATACATATAT
PF13_0033; 1938 3.66e-06 CACACATACA TACATACATAC ATATATACAT
PFC0745c; 1178 3.66e-06 TCATATATAA TACATACATAC ATATATACAT
PFA0400c; 553 3.66e-06 ATTAAATTGT TACATACATAC ATACATATAT
PF13_0063; 1054 4.91e-06 CCTTTTTAAT AGCATACATAC ATACATATAT
PF10_0174; 1021 6.89e-06 AGTATTGTTT CGCATATGTAT GGATATATAT
PFI1545c; 12 7.69e-06 TTAAATATAT TCCCCACATAA TAAAATATTT*
PF14_0676; 261 8.49e-06 AAATTTAATA CACACACAAAR AAAAAAAAAA
PF07_0112; 407 9.77e-06 ATTTTTATAA TACCCACATAA TAAAAATATT*
PFE0915c; 995 1.10e-05 AATTATAAAT TGCCCAGATAT TATACATAAC*
PF14_0025; 56 1.22e-05 TAAAATTTGT TGCACATAAAT AAATAAATAA
PF14_0716; 945 1.37e-05 AATGAATATA CGCACAAGAAT TATATATATA
PF11_0314; 974 1.37e-05 TATTATTTTA TGCATATGTAT AAAAAAAAAA
PF10_0081; 1146 1.74e-05 TACCATACTT GGCACATATAA AAAAGAAATA
MAL8P1.128; 781 2.18e-05 TGTAATTTTA TACATACATAT TTTGTATTGT
PF10_0174; 396 2.18e-05 AAAAAAAAAT TCCACACAAAA TCAAAATATT
PFI0630w; 1279 2.18e-05 ATATATATAA TACATACATAT TCAAAAAAAA
PFB0260w; 1242 2.18e-05 TCATCTTCTT TGCCCAAATAT ATATACATAT
PFB0260w; 382 2.18e-05 CAAAATAAAA TACATACATAT ATTTATATAT
PFB0260w; 1341 2.42e-05 ATTGAAAACT ACCCCACAGAT AAGATGAAAA*
PFF0420c; 496 3.18e-05 TTTTTTTTAA AGCATACAGAT TATTAAAAAA
PF13_0033; 22 3.18e-05 TATATATATG TGCATATATAT TTATTTATCA
PF14_0676; 133 3.76e-05 TATATGTATA TGCATATGAAT GATTTATTTA
PF07_0112; 239 3.76e-05 TTATATATGT GGCATATATAT ATTATTAATT
MAL8P1.128; 751 4.30e-05 TATATTATAA TACACATGAAT ATAAATAAAT
MAL13P1.190; 828 4.30e-05 CATACATACA TACATATATAC AATATATATA
MAL8P1.142; 1811 4.30e-05 TTTATATCTA TGCACATAAAA TAAATAAAAA
MAL8P1.142; 1415 4.30e-05 TAAAAAATAT TACATATATAC ATTTATATAT
PF14_0676; 896 4.64e-05 TTATATGATT TACATATGAAC GTAAAAATAA
PF14_0632; 320 4.64e-05 TTCCCTTAAT CACACATAAAT AAAAAAAAAT
PF13_0033; 395 4.64e-05 TGTAGATATT GACATATATAC ATTTATATAT
PF07_0112; 595 5.41e-05 TATGCCTTTG AACACATATAC TAAAATATAT
PF14_0632; 276 5.65e-05 AAATTAAGAA TGCACAAAAAT TAAAAAAAAA
PFF0420c; 1441 6.18e-05 TATAAAGTAT TACATACATAA ATACATAATA
PFF0420c; 974 7.73e-05 TTTAATGTTT CCCATATATAT GAGATAATAC
PF14_0025; 1535 7.73e-05 AAAATATTTA TACATAAGTAC ATATTTTTAT
PF14_0676; 650 7.73e-05 TCCAAACATA TACATACAAGT TTAATATTTG
MEME, protea_uig, anr2, TACATACATAT,w=11,s=48,llr=457,E=5.1e-010
MEME, protea_uig,zoops1,GCACAC,w=6,s=26,llr=246,E=1.1e-009
zoops1
PFI1545c; 13 7.34e-07 TAAATATATT CCCCAC ATAATAAAAT
PFB0260w; 1342 7.34e-07 TTGAAAACTA CCCCAC AGATAAGATG*
PFE0915c; 996 1.49e-06 ATTATAAATT GCCCAG ATATTATACA*
PF13_0156; 384 2.26e-06 ATTATATATT CCCCTC ATTTTAATTA
PF13_0063; 797 2.26e-06 CTACACAAAA CCCCTC TCTCATTTCA
MAL8P1.142; 874 2.26e-06 TTTTTTTTTC CCCCTC ATTTTAACCA
PFF0420c; 1263 4.93e-06 TTATTCGTTA GCACAC AATTCAAGTT
PF14_0025; 1518 4.93e-06 TTATTATTAT GCACAC AAAAATATTT*
PF13_0033; 1923 4.93e-06 ATTTTGTTTA GCACAC ACACATACAT*
PF10_0174; 485 4.93e-06 TTATATATGA GCACAC TTTATAACAT
PFI0630w; 1117 4.93e-06 TAAAAATATG GCACAC CATATTTTAA
PF11_0314; 695 9.53e-06 AATTTATAAA GCGCTC TATTAATATA
PFD0665c; 1183 9.53e-06 ATTTAATTTA GCACGC AATTTTGTTT
PFC0745c; 867 1.48e-05 ATGACATTAT GCCCAA GCTCCCATAT*
PF14_0716; 1158 1.82e-05 TAAAATTTAC GCACAG TATTATAAGA
PFC0520w; 1071 1.82e-05 CATGTTTAAT GCACAG TAATATTTTG
PF13_0282; 858 2.43e-05 AATTCTTACA CCCCAA AATAAGAAAA
MAL13P1.343; 27 3.03e-05 TCCTAGTATG GCCCTA AGAAACTTCA
PF07_0112; 408 3.63e-05 TTTTTATAAT ACCCAC ATAATAAAAA*
PFA0400c; 39 4.20e-05 TAAAGCATAA GCGCAA AAGAACAAAA
PF14_0632; 277 6.23e-05 AATTAAGAAT GCACAA AAATTAAAAA
MAL13P1.190; 1647 6.49e-05 TTATTTATAT CCACTG AGTATATTAT
PF10_0081; 63 9.32e-05 TTTATATATT CCACAA TGGGATATAT
MAL13P1.270; 638 1.20e-04 ATTCAAATAA GCACTA AAGTAAATGT
PF14_0676; 260 1.46e-04 TAAATTTAAT ACACAC ACAAARAAAA
PF10_0298; 393 1.99e-04 ATAATATAAA TCACAC TTAAAAAGAA
29

## Slide 30
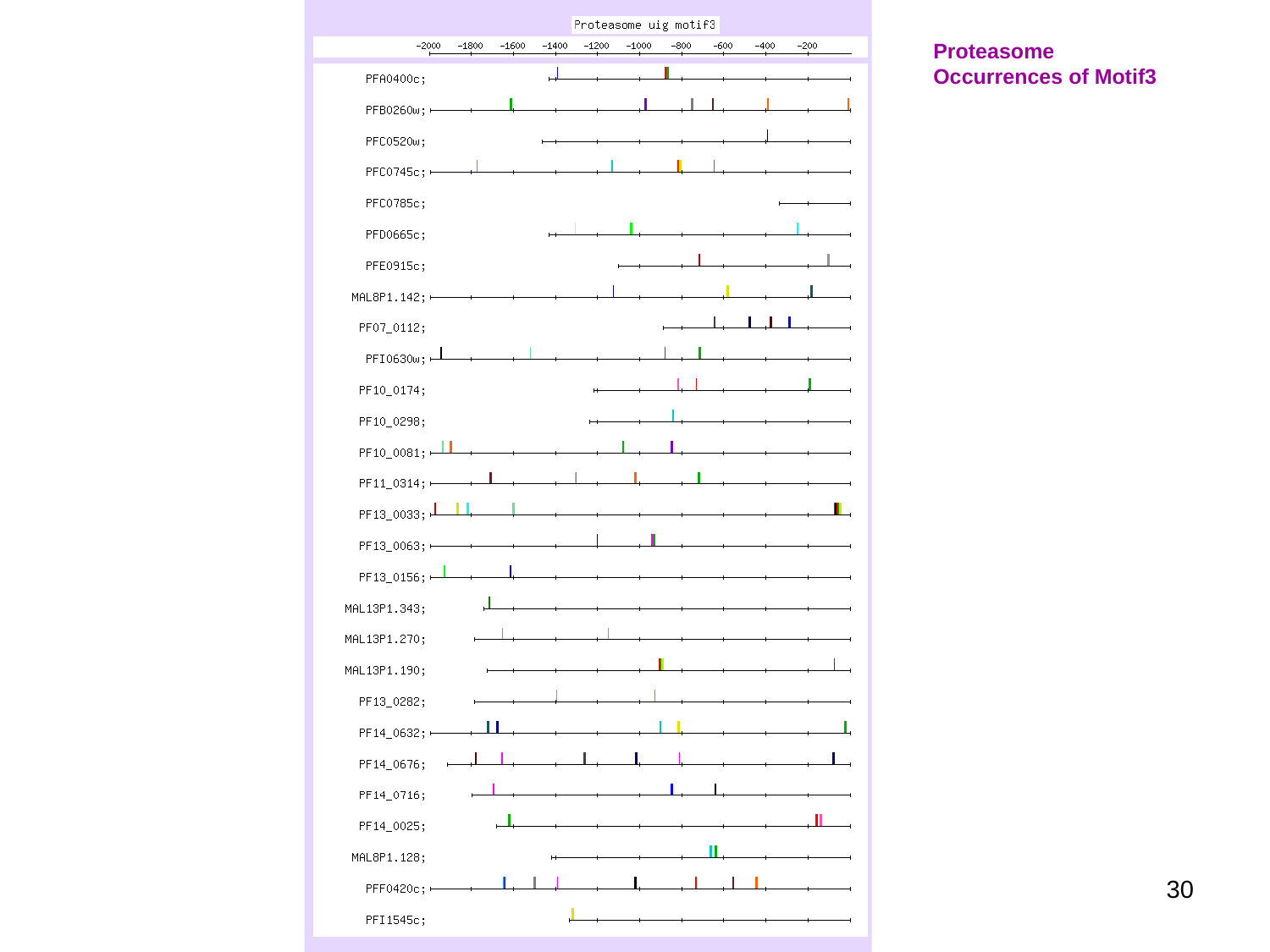

Proteasome
Occurrences of Motif3
30

## Slide 31
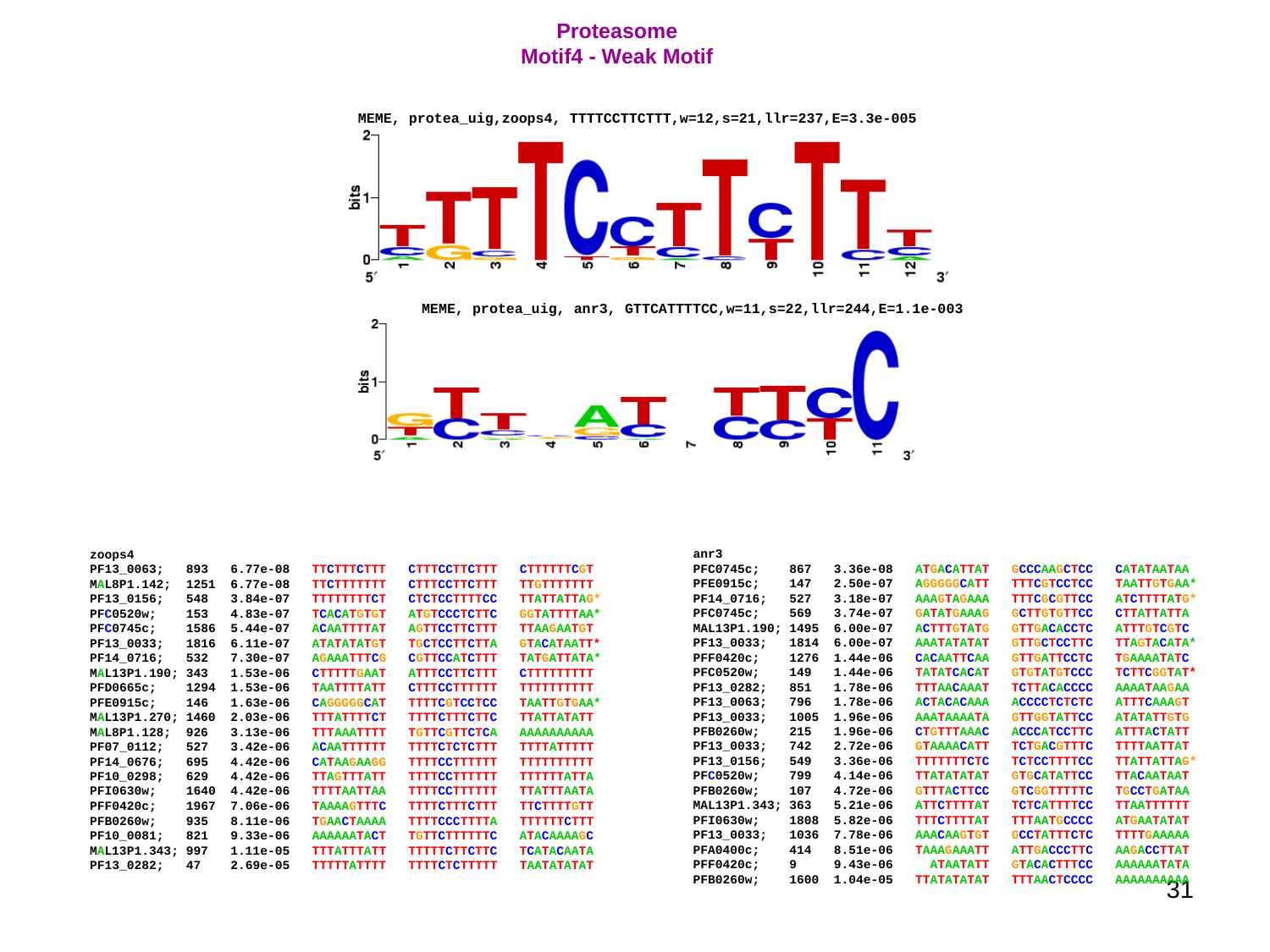

Proteasome
Motif4 - Weak Motif
MEME, protea_uig,zoops4, TTTTCCTTCTTT,w=12,s=21,llr=237,E=3.3e-005
MEME, protea_uig, anr3, GTTCATTTTCC,w=11,s=22,llr=244,E=1.1e-003
anr3
PFC0745c; 867 3.36e-08 ATGACATTAT GCCCAAGCTCC CATATAATAA
PFE0915c; 147 2.50e-07 AGGGGGCATT TTTCGTCCTCC TAATTGTGAA*
PF14_0716; 527 3.18e-07 AAAGTAGAAA TTTCGCGTTCC ATCTTTTATG*
PFC0745c; 569 3.74e-07 GATATGAAAG GCTTGTGTTCC CTTATTATTA
MAL13P1.190; 1495 6.00e-07 ACTTTGTATG GTTGACACCTC ATTTGTCGTC
PF13_0033; 1814 6.00e-07 AAATATATAT GTTGCTCCTTC TTAGTACATA*
PFF0420c; 1276 1.44e-06 CACAATTCAA GTTGATTCCTC TGAAAATATC
PFC0520w; 149 1.44e-06 TATATCACAT GTGTATGTCCC TCTTCGGTAT*
PF13_0282; 851 1.78e-06 TTTAACAAAT TCTTACACCCC AAAATAAGAA
PF13_0063; 796 1.78e-06 ACTACACAAA ACCCCTCTCTC ATTTCAAAGT
PF13_0033; 1005 1.96e-06 AAATAAAATA GTTGGTATTCC ATATATTGTG
PFB0260w; 215 1.96e-06 CTGTTTAAAC ACCCATCCTTC ATTTACTATT
PF13_0033; 742 2.72e-06 GTAAAACATT TCTGACGTTTC TTTTAATTAT
PF13_0156; 549 3.36e-06 TTTTTTTCTC TCTCCTTTTCC TTATTATTAG*
PFC0520w; 799 4.14e-06 TTATATATAT GTGCATATTCC TTACAATAAT
PFB0260w; 107 4.72e-06 GTTTACTTCC GTCGGTTTTTC TGCCTGATAA
MAL13P1.343; 363 5.21e-06 ATTCTTTTAT TCTCATTTTCC TTAATTTTTT
PFI0630w; 1808 5.82e-06 TTTCTTTTAT TTTAATGCCCC ATGAATATAT
PF13_0033; 1036 7.78e-06 AAACAAGTGT GCCTATTTCTC TTTTGAAAAA
PFA0400c; 414 8.51e-06 TAAAGAAATT ATTGACCCTTC AAGACCTTAT
PFF0420c; 9 9.43e-06 ATAATATT GTACACTTTCC AAAAAATATA
PFB0260w; 1600 1.04e-05 TTATATATAT TTTAACTCCCC AAAAAAAAAA
zoops4
PF13_0063; 893 6.77e-08 TTCTTTCTTT CTTTCCTTCTTT CTTTTTTCGT
MAL8P1.142; 1251 6.77e-08 TTCTTTTTTT CTTTCCTTCTTT TTGTTTTTTT
PF13_0156; 548 3.84e-07 TTTTTTTTCT CTCTCCTTTTCC TTATTATTAG*
PFC0520w; 153 4.83e-07 TCACATGTGT ATGTCCCTCTTC GGTATTTTAA*
PFC0745c; 1586 5.44e-07 ACAATTTTAT AGTTCCTTCTTT TTAAGAATGT
PF13_0033; 1816 6.11e-07 ATATATATGT TGCTCCTTCTTA GTACATAATT*
PF14_0716; 532 7.30e-07 AGAAATTTCG CGTTCCATCTTT TATGATTATA*
MAL13P1.190; 343 1.53e-06 CTTTTTGAAT ATTTCCTTCTTT CTTTTTTTTT
PFD0665c; 1294 1.53e-06 TAATTTTATT CTTTCCTTTTTT TTTTTTTTTT
PFE0915c; 146 1.63e-06 CAGGGGGCAT TTTTCGTCCTCC TAATTGTGAA*
MAL13P1.270; 1460 2.03e-06 TTTATTTTCT TTTTCTTTCTTC TTATTATATT
MAL8P1.128; 926 3.13e-06 TTTAAATTTT TGTTCGTTCTCA AAAAAAAAAA
PF07_0112; 527 3.42e-06 ACAATTTTTT TTTTCTCTCTTT TTTTATTTTT
PF14_0676; 695 4.42e-06 CATAAGAAGG TTTTCCTTTTTT TTTTTTTTTT
PF10_0298; 629 4.42e-06 TTAGTTTATT TTTTCCTTTTTT TTTTTTATTA
PFI0630w; 1640 4.42e-06 TTTTAATTAA TTTTCCTTTTTT TTATTTAATA
PFF0420c; 1967 7.06e-06 TAAAAGTTTC TTTTCTTTCTTT TTCTTTTGTT
PFB0260w; 935 8.11e-06 TGAACTAAAA TTTTCCCTTTTA TTTTTTCTTT
PF10_0081; 821 9.33e-06 AAAAAATACT TGTTCTTTTTTC ATACAAAAGC
MAL13P1.343; 997 1.11e-05 TTTATTTATT TTTTTCTTCTTC TCATACAATA
PF13_0282; 47 2.69e-05 TTTTTATTTT TTTTCTCTTTTT TAATATATAT
31

## Slide 32
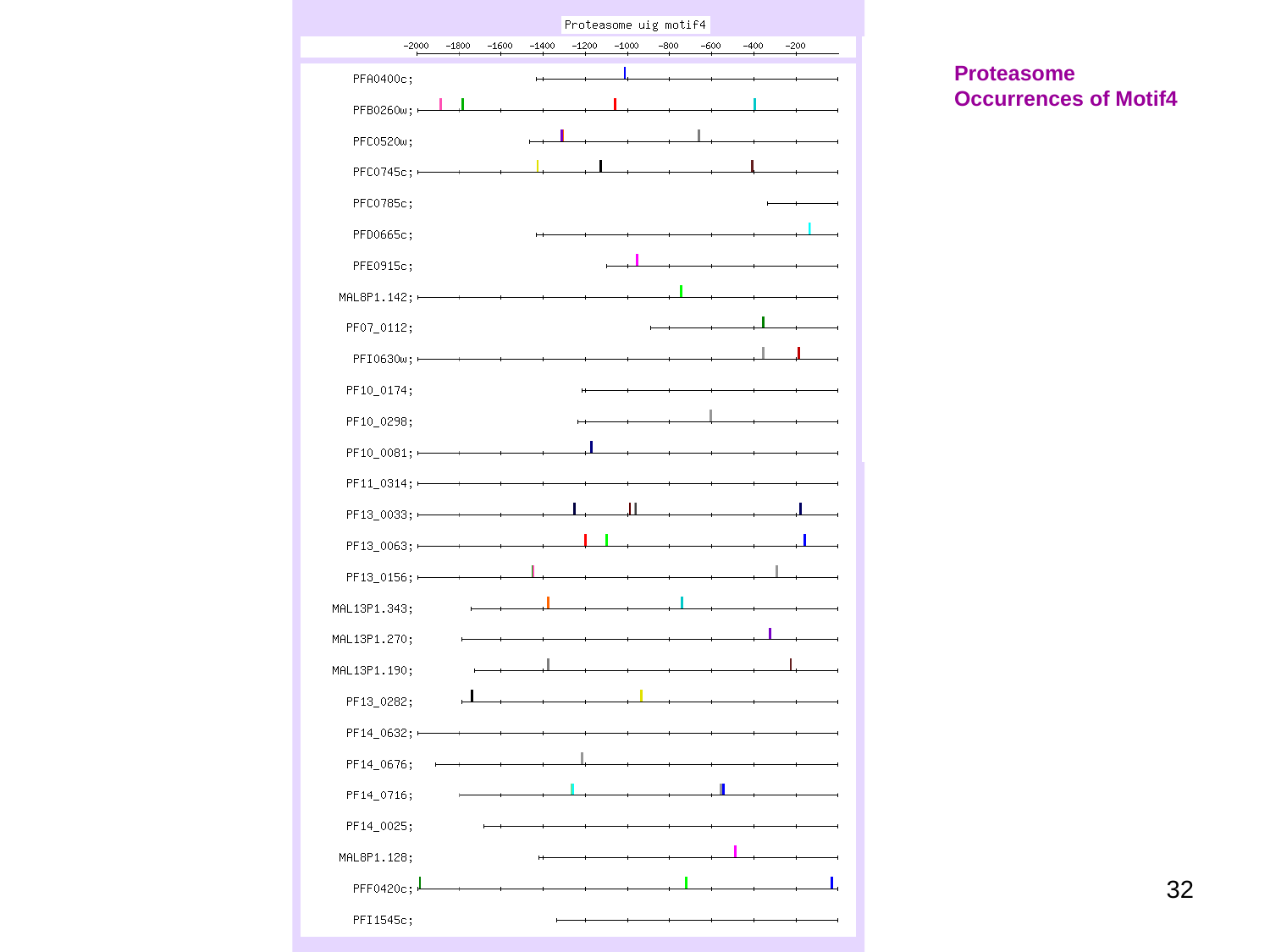

Proteasome
Occurrences of Motif4
32

## Slide 33
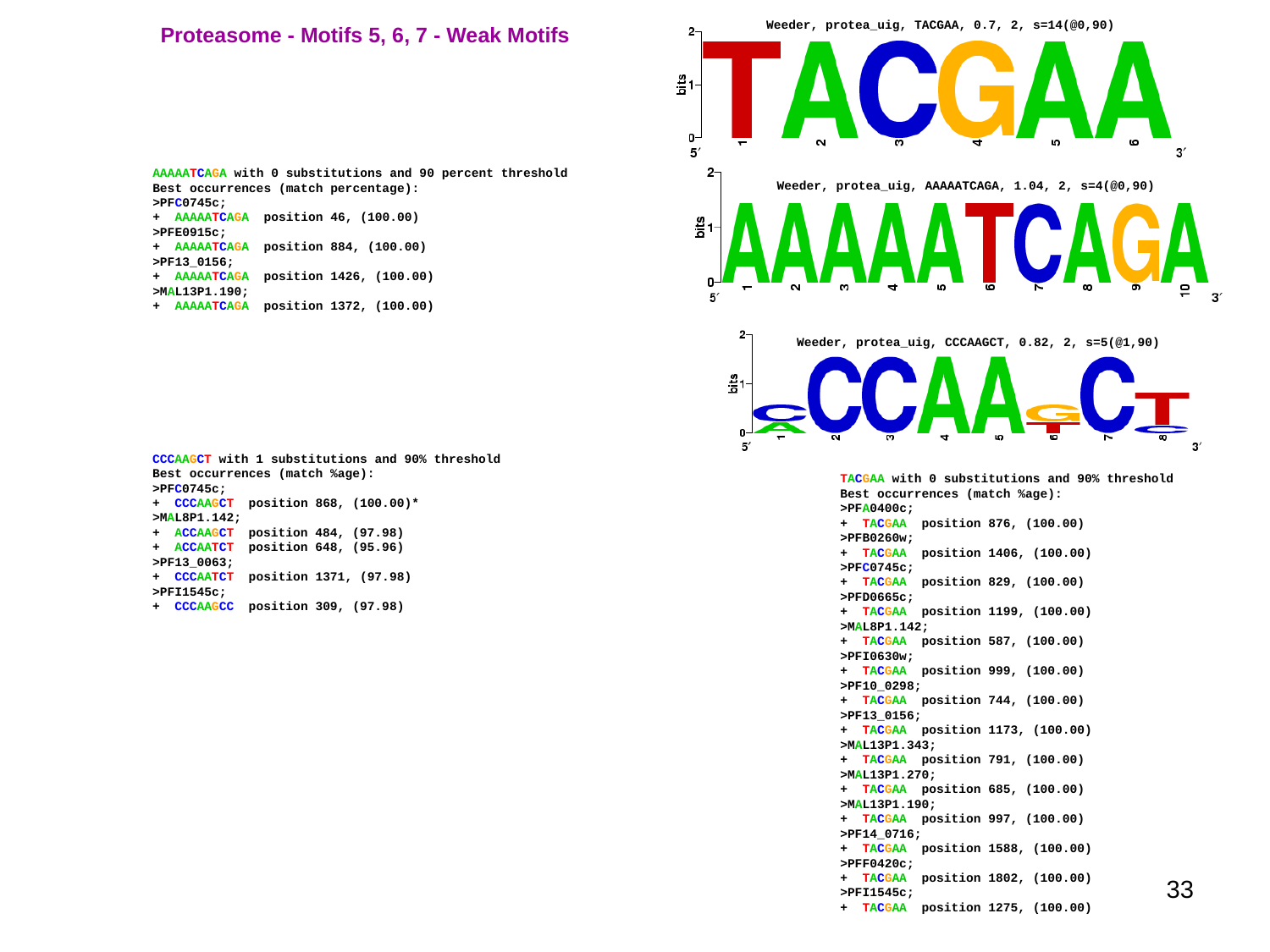

Weeder, protea_uig, TACGAA, 0.7, 2, s=14(@0,90)
Proteasome - Motifs 5, 6, 7 - Weak Motifs
AAAAATCAGA with 0 substitutions and 90 percent threshold
Best occurrences (match percentage):
>PFC0745c;
+ AAAAATCAGA position 46, (100.00)
>PFE0915c;
+ AAAAATCAGA position 884, (100.00)
>PF13_0156;
+ AAAAATCAGA position 1426, (100.00)
>MAL13P1.190;
+ AAAAATCAGA position 1372, (100.00)
Weeder, protea_uig, AAAAATCAGA, 1.04, 2, s=4(@0,90)
Weeder, protea_uig, CCCAAGCT, 0.82, 2, s=5(@1,90)
CCCAAGCT with 1 substitutions and 90% threshold
Best occurrences (match %age):
>PFC0745c;
+ CCCAAGCT position 868, (100.00)*
>MAL8P1.142;
+ ACCAAGCT position 484, (97.98)
+ ACCAATCT position 648, (95.96)
>PF13_0063;
+ CCCAATCT position 1371, (97.98)
>PFI1545c;
+ CCCAAGCC position 309, (97.98)
TACGAA with 0 substitutions and 90% threshold
Best occurrences (match %age):
>PFA0400c;
+ TACGAA position 876, (100.00)
>PFB0260w;
+ TACGAA position 1406, (100.00)
>PFC0745c;
+ TACGAA position 829, (100.00)
>PFD0665c;
+ TACGAA position 1199, (100.00)
>MAL8P1.142;
+ TACGAA position 587, (100.00)
>PFI0630w;
+ TACGAA position 999, (100.00)
>PF10_0298;
+ TACGAA position 744, (100.00)
>PF13_0156;
+ TACGAA position 1173, (100.00)
>MAL13P1.343;
+ TACGAA position 791, (100.00)
>MAL13P1.270;
+ TACGAA position 685, (100.00)
>MAL13P1.190;
+ TACGAA position 997, (100.00)
>PF14_0716;
+ TACGAA position 1588, (100.00)
>PFF0420c;
+ TACGAA position 1802, (100.00)
>PFI1545c;
+ TACGAA position 1275, (100.00)
33

## Slide 34
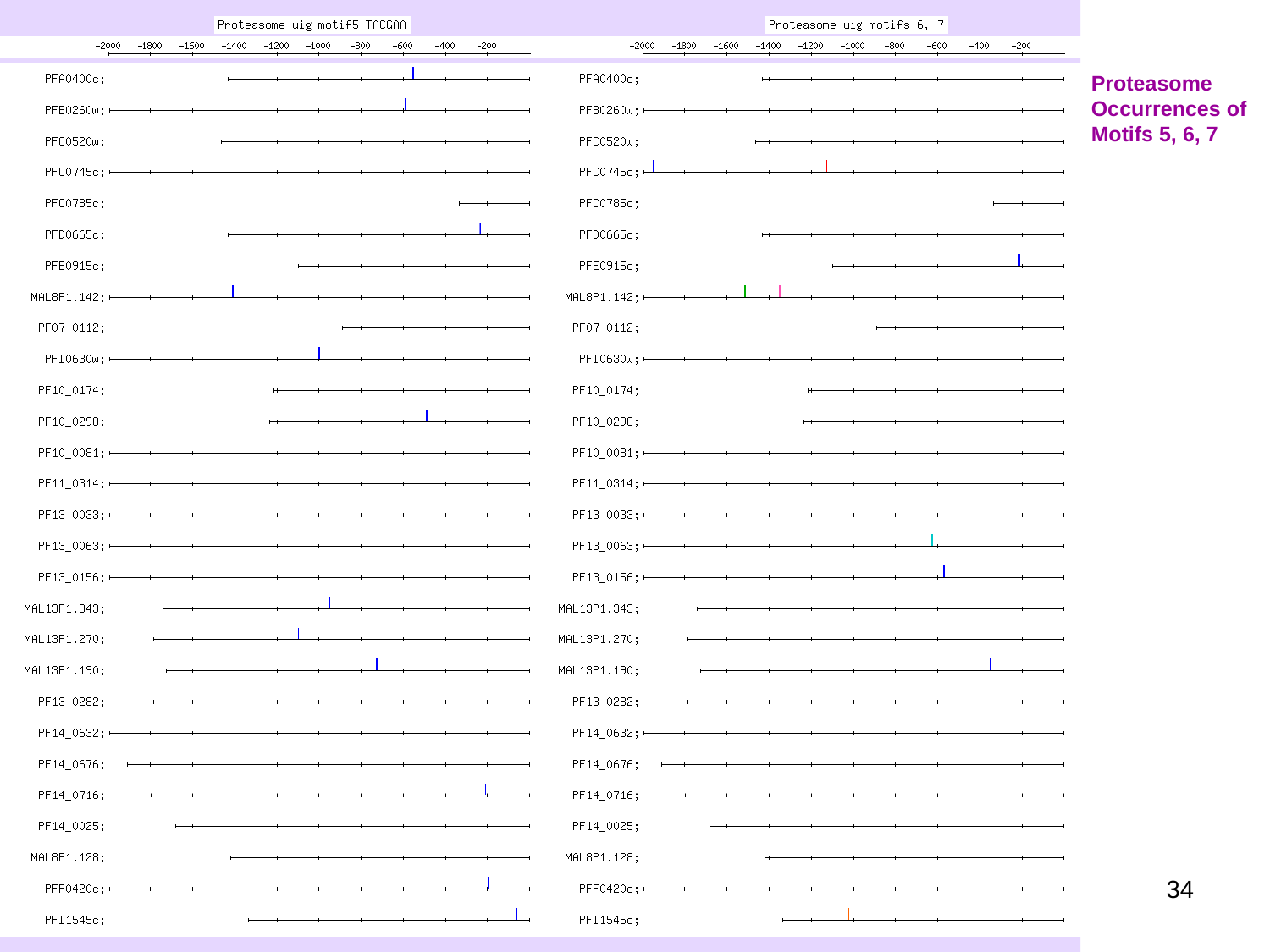

Proteasome
Occurrences of
Motifs 5, 6, 7
34

## Slide 35
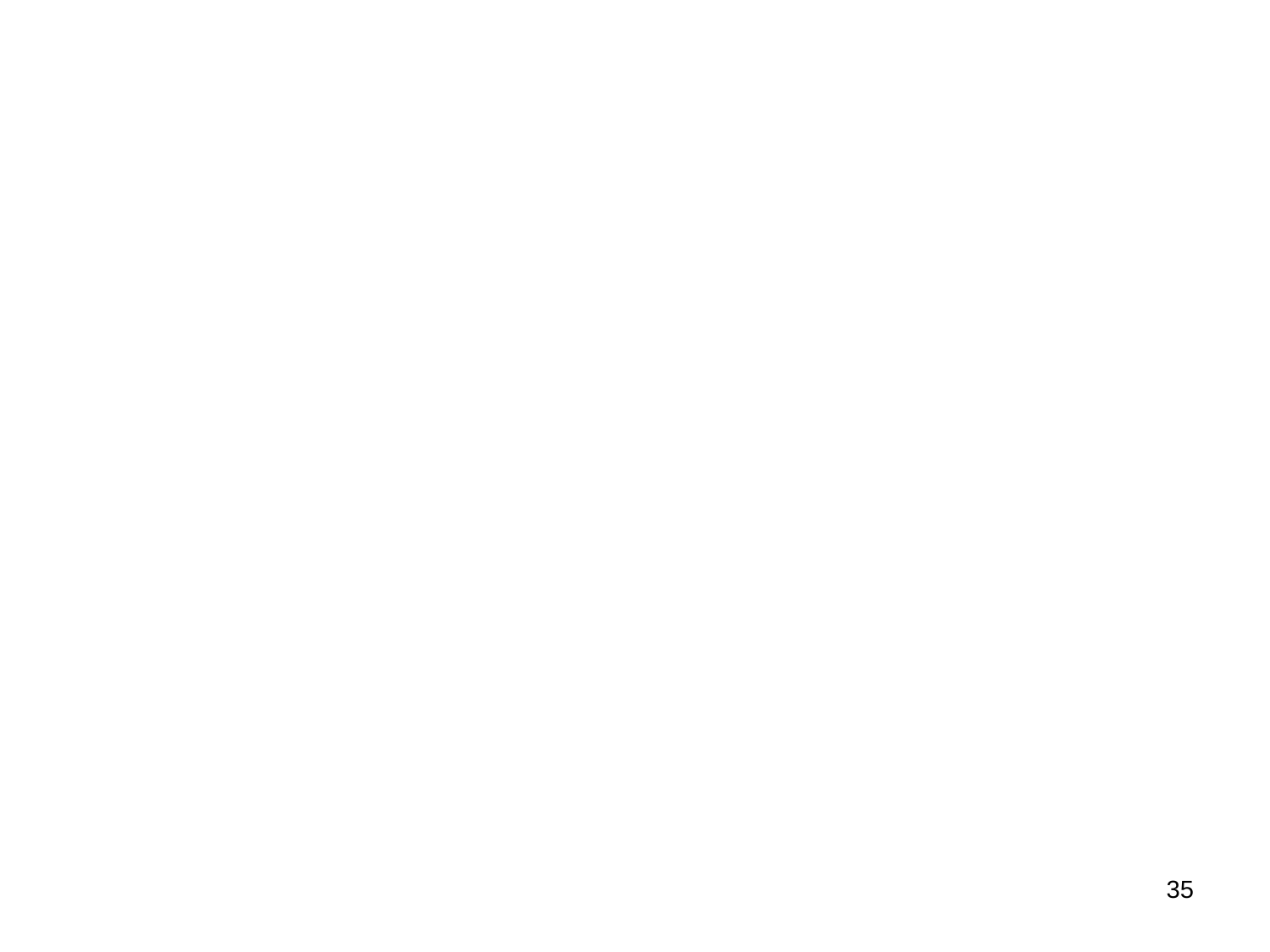

35

## Slide 36
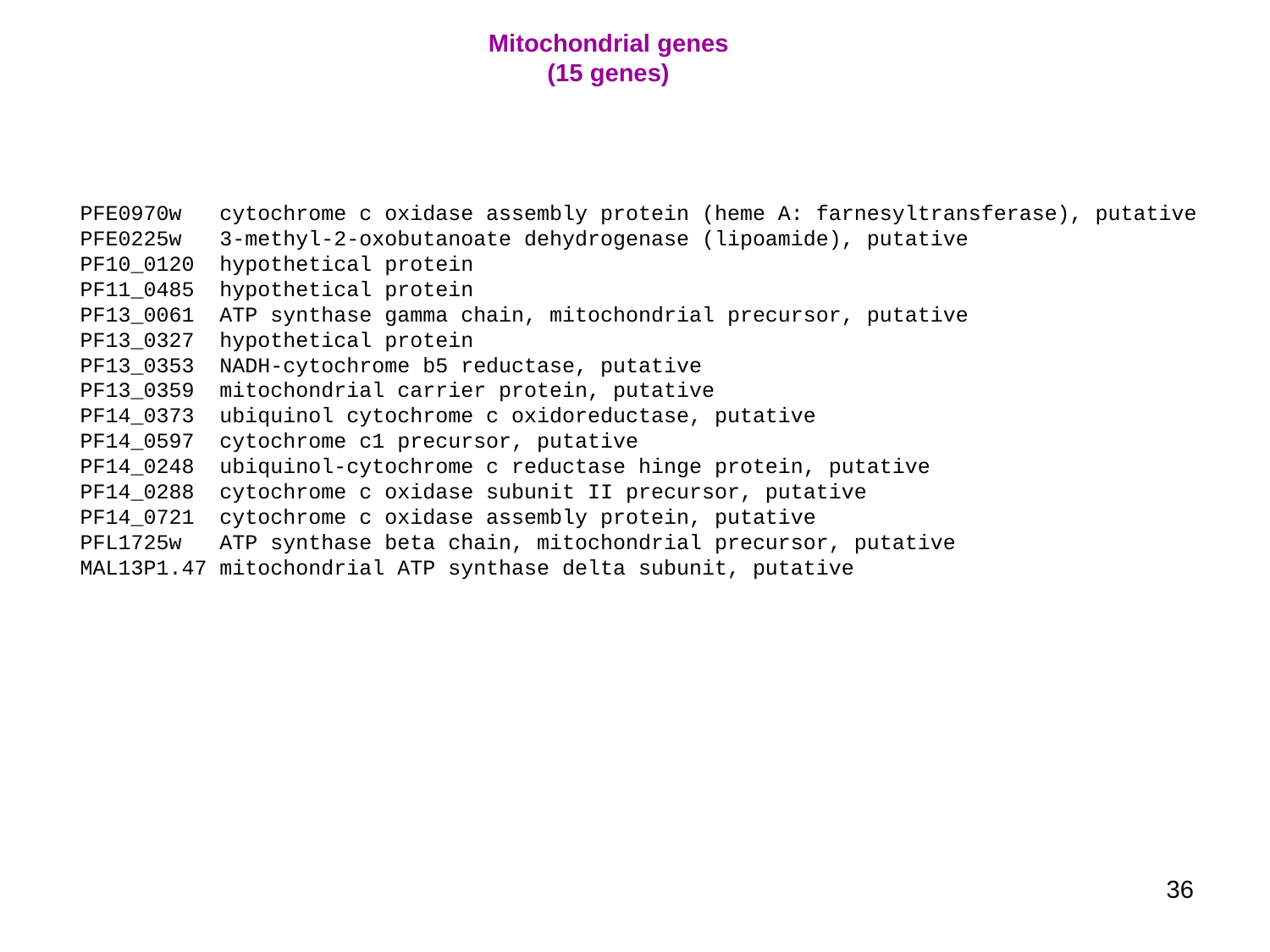

Mitochondrial genes
(15 genes)
PFE0970w cytochrome c oxidase assembly protein (heme A: farnesyltransferase), putative
PFE0225w 3-methyl-2-oxobutanoate dehydrogenase (lipoamide), putative
PF10_0120 hypothetical protein
PF11_0485 hypothetical protein
PF13_0061 ATP synthase gamma chain, mitochondrial precursor, putative
PF13_0327 hypothetical protein
PF13_0353 NADH-cytochrome b5 reductase, putative
PF13_0359 mitochondrial carrier protein, putative
PF14_0373 ubiquinol cytochrome c oxidoreductase, putative
PF14_0597 cytochrome c1 precursor, putative
PF14_0248 ubiquinol-cytochrome c reductase hinge protein, putative
PF14_0288 cytochrome c oxidase subunit II precursor, putative
PF14_0721 cytochrome c oxidase assembly protein, putative
PFL1725w ATP synthase beta chain, mitochondrial precursor, putative
MAL13P1.47 mitochondrial ATP synthase delta subunit, putative
36

## Slide 37
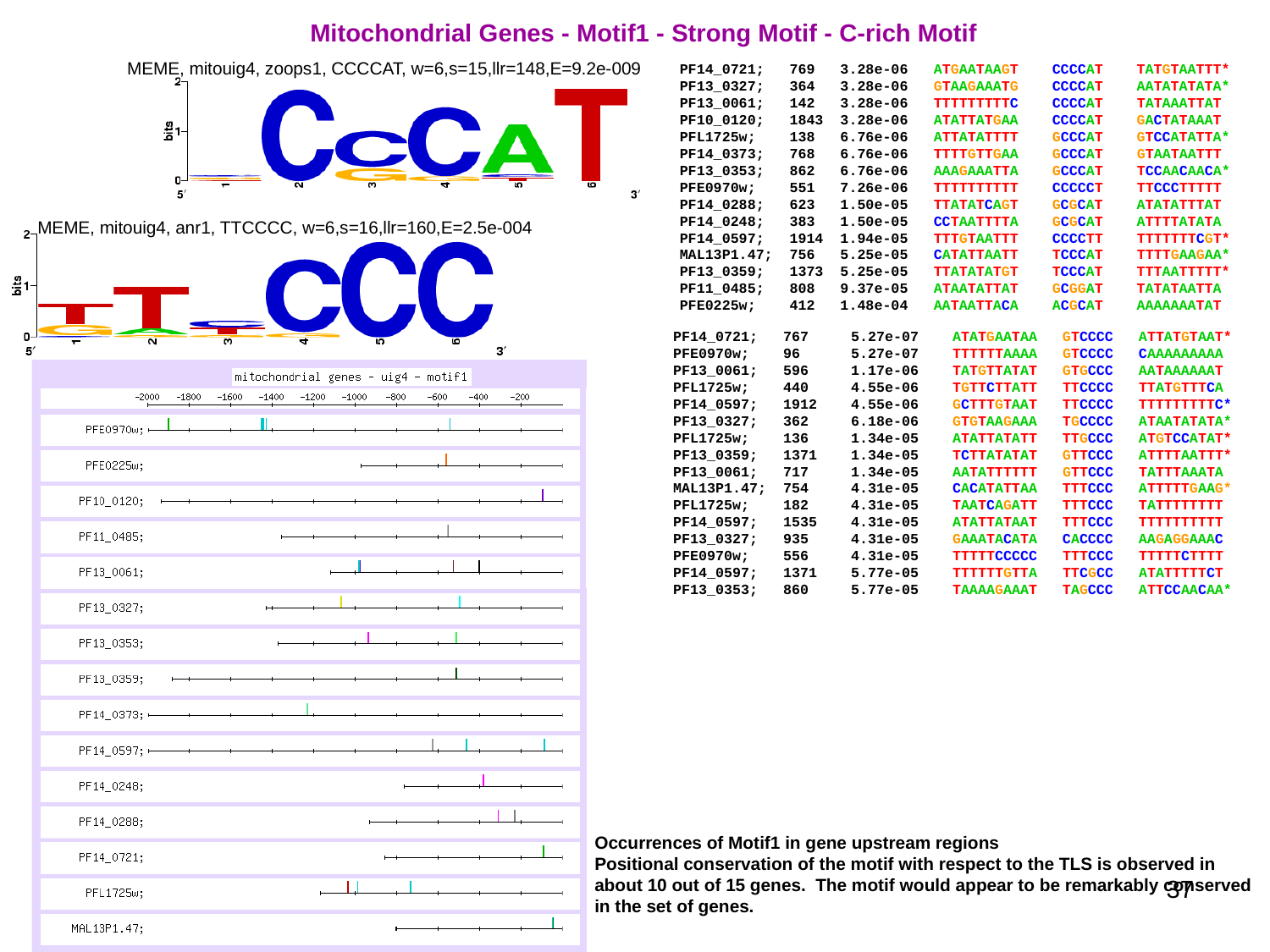

Mitochondrial Genes - Motif1 - Strong Motif - C-rich Motif
MEME, mitouig4, zoops1, CCCCAT, w=6,s=15,llr=148,E=9.2e-009
MEME, mitouig4, anr1, TTCCCC, w=6,s=16,llr=160,E=2.5e-004
PF14_0721; 769 3.28e-06 ATGAATAAGT CCCCAT TATGTAATTT*
PF13_0327; 364 3.28e-06 GTAAGAAATG CCCCAT AATATATATA*
PF13_0061; 142 3.28e-06 TTTTTTTTTC CCCCAT TATAAATTAT
PF10_0120; 1843 3.28e-06 ATATTATGAA CCCCAT GACTATAAAT
PFL1725w; 138 6.76e-06 ATTATATTTT GCCCAT GTCCATATTA*
PF14_0373; 768 6.76e-06 TTTTGTTGAA GCCCAT GTAATAATTT
PF13_0353; 862 6.76e-06 AAAGAAATTA GCCCAT TCCAACAACA*
PFE0970w; 551 7.26e-06 TTTTTTTTTT CCCCCT TTCCCTTTTT
PF14_0288; 623 1.50e-05 TTATATCAGT GCGCAT ATATATTTAT
PF14_0248; 383 1.50e-05 CCTAATTTTA GCGCAT ATTTTATATA
PF14_0597; 1914 1.94e-05 TTTGTAATTT CCCCTT TTTTTTTCGT*
MAL13P1.47; 756 5.25e-05 CATATTAATT TCCCAT TTTTGAAGAA*
PF13_0359; 1373 5.25e-05 TTATATATGT TCCCAT TTTAATTTTT*
PF11_0485; 808 9.37e-05 ATAATATTAT GCGGAT TATATAATTA
PFE0225w; 412 1.48e-04 AATAATTACA ACGCAT AAAAAAATAT
PF14_0721; 767 5.27e-07 ATATGAATAA GTCCCC ATTATGTAAT*
PFE0970w; 96 5.27e-07 TTTTTTAAAA GTCCCC CAAAAAAAAA
PF13_0061; 596 1.17e-06 TATGTTATAT GTGCCC AATAAAAAAT
PFL1725w; 440 4.55e-06 TGTTCTTATT TTCCCC TTATGTTTCA
PF14_0597; 1912 4.55e-06 GCTTTGTAAT TTCCCC TTTTTTTTTC*
PF13_0327; 362 6.18e-06 GTGTAAGAAA TGCCCC ATAATATATA*
PFL1725w; 136 1.34e-05 ATATTATATT TTGCCC ATGTCCATAT*
PF13_0359; 1371 1.34e-05 TCTTATATAT GTTCCC ATTTTAATTT*
PF13_0061; 717 1.34e-05 AATATTTTTT GTTCCC TATTTAAATA
MAL13P1.47; 754 4.31e-05 CACATATTAA TTTCCC ATTTTTGAAG*
PFL1725w; 182 4.31e-05 TAATCAGATT TTTCCC TATTTTTTTT
PF14_0597; 1535 4.31e-05 ATATTATAAT TTTCCC TTTTTTTTTT
PF13_0327; 935 4.31e-05 GAAATACATA CACCCC AAGAGGAAAC
PFE0970w; 556 4.31e-05 TTTTTCCCCC TTTCCC TTTTTCTTTT
PF14_0597; 1371 5.77e-05 TTTTTTGTTA TTCGCC ATATTTTTCT
PF13_0353; 860 5.77e-05 TAAAAGAAAT TAGCCC ATTCCAACAA*
Occurrences of Motif1 in gene upstream regions
Positional conservation of the motif with respect to the TLS is observed in
about 10 out of 15 genes. The motif would appear to be remarkably conserved
in the set of genes.
37

## Slide 38
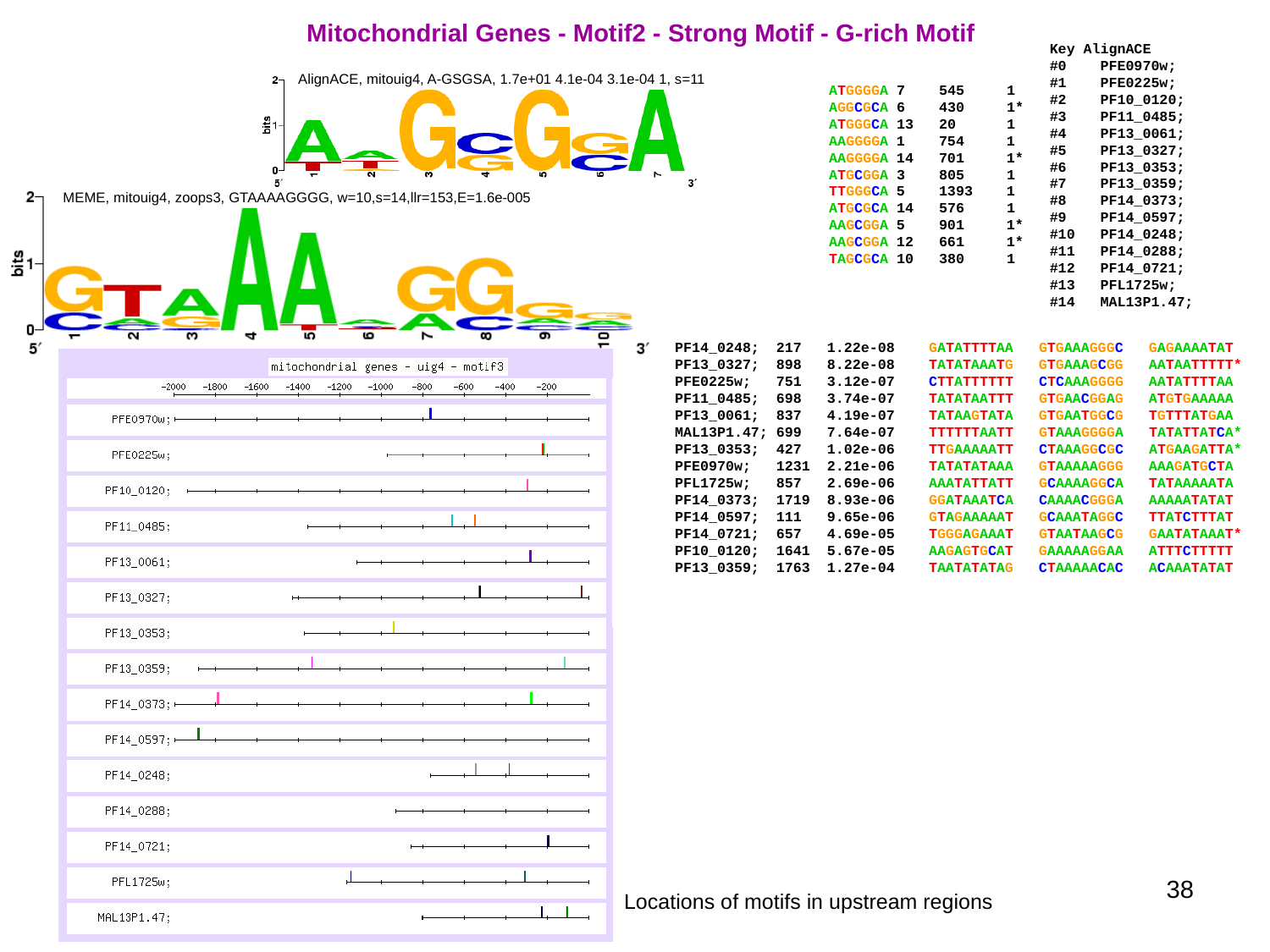

Mitochondrial Genes - Motif2 - Strong Motif - G-rich Motif
Key AlignACE
#0 PFE0970w;
#1 PFE0225w;
#2 PF10_0120;
#3 PF11_0485;
#4 PF13_0061;
#5 PF13_0327;
#6 PF13_0353;
#7 PF13_0359;
#8 PF14_0373;
#9 PF14_0597;
#10 PF14_0248;
#11 PF14_0288;
#12 PF14_0721;
#13 PFL1725w;
#14 MAL13P1.47;
AlignACE, mitouig4, A-GSGSA, 1.7e+01 4.1e-04 3.1e-04 1, s=11
ATGGGGA 7 545 1
AGGCGCA 6 430 1*
ATGGGCA 13 20 1
AAGGGGA 1 754 1
AAGGGGA 14 701 1*
ATGCGGA 3 805 1
TTGGGCA 5 1393 1
ATGCGCA 14 576 1
AAGCGGA 5 901 1*
AAGCGGA 12 661 1*
TAGCGCA 10 380 1
MEME, mitouig4, zoops3, GTAAAAGGGG, w=10,s=14,llr=153,E=1.6e-005
PF14_0248; 217 1.22e-08 GATATTTTAA GTGAAAGGGC GAGAAAATAT
PF13_0327; 898 8.22e-08 TATATAAATG GTGAAAGCGG AATAATTTTT*
PFE0225w; 751 3.12e-07 CTTATTTTTT CTCAAAGGGG AATATTTTAA
PF11_0485; 698 3.74e-07 TATATAATTT GTGAACGGAG ATGTGAAAAA
PF13_0061; 837 4.19e-07 TATAAGTATA GTGAATGGCG TGTTTATGAA
MAL13P1.47; 699 7.64e-07 TTTTTTAATT GTAAAGGGGA TATATTATCA*
PF13_0353; 427 1.02e-06 TTGAAAAATT CTAAAGGCGC ATGAAGATTA*
PFE0970w; 1231 2.21e-06 TATATATAAA GTAAAAAGGG AAAGATGCTA
PFL1725w; 857 2.69e-06 AAATATTATT GCAAAAGGCA TATAAAAATA
PF14_0373; 1719 8.93e-06 GGATAAATCA CAAAACGGGA AAAAATATAT
PF14_0597; 111 9.65e-06 GTAGAAAAAT GCAAATAGGC TTATCTTTAT
PF14_0721; 657 4.69e-05 TGGGAGAAAT GTAATAAGCG GAATATAAAT*
PF10_0120; 1641 5.67e-05 AAGAGTGCAT GAAAAAGGAA ATTTCTTTTT
PF13_0359; 1763 1.27e-04 TAATATATAG CTAAAAACAC ACAAATATAT
38
Locations of motifs in upstream regions

## Slide 39
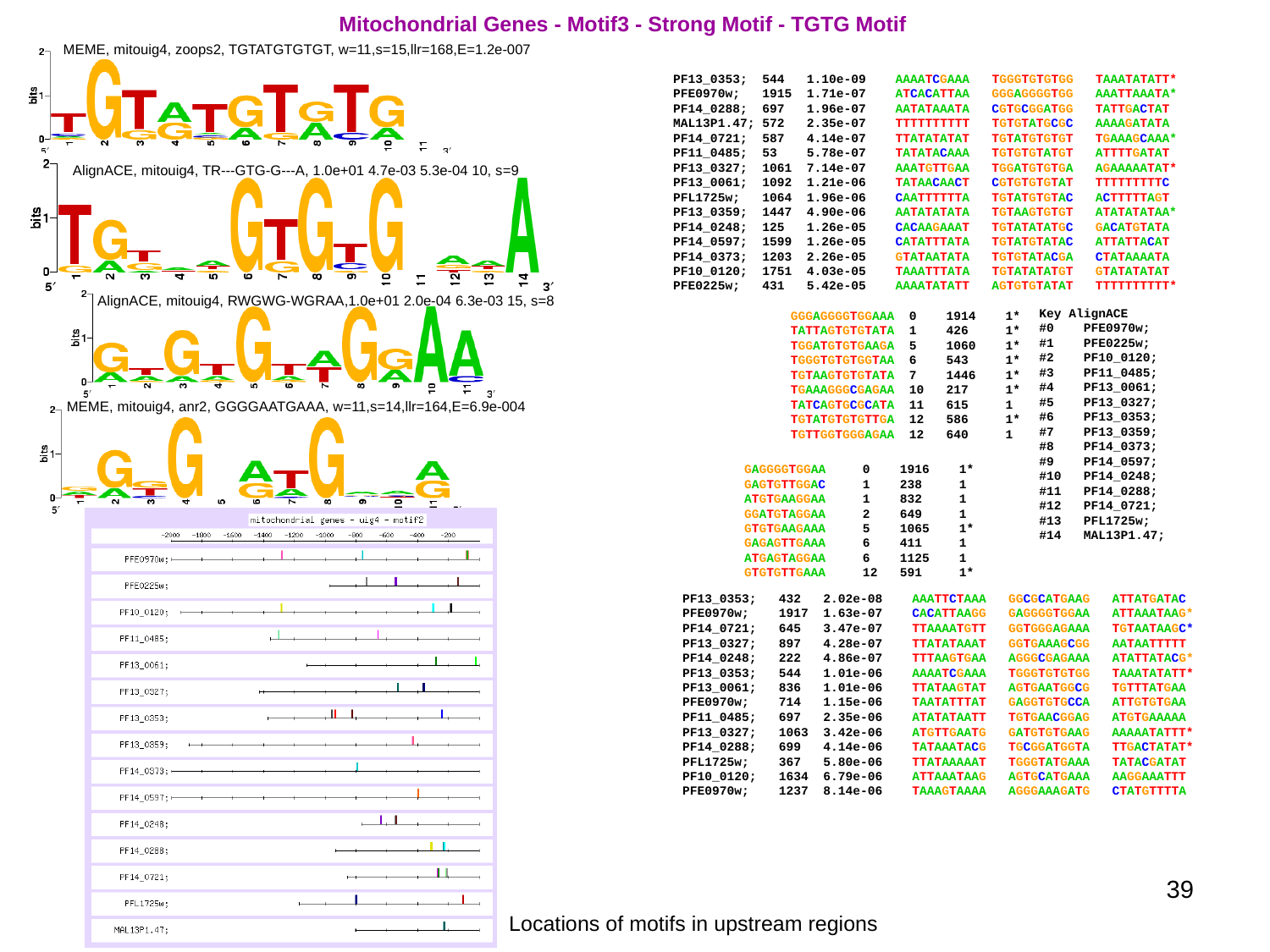

Mitochondrial Genes - Motif3 - Strong Motif - TGTG Motif
MEME, mitouig4, zoops2, TGTATGTGTGT, w=11,s=15,llr=168,E=1.2e-007
PF13_0353; 544 1.10e-09 AAAATCGAAA TGGGTGTGTGG TAAATATATT*
PFE0970w; 1915 1.71e-07 ATCACATTAA GGGAGGGGTGG AAATTAAATA*
PF14_0288; 697 1.96e-07 AATATAAATA CGTGCGGATGG TATTGACTAT
MAL13P1.47; 572 2.35e-07 TTTTTTTTTT TGTGTATGCGC AAAAGATATA
PF14_0721; 587 4.14e-07 TTATATATAT TGTATGTGTGT TGAAAGCAAA*
PF11_0485; 53 5.78e-07 TATATACAAA TGTGTGTATGT ATTTTGATAT
PF13_0327; 1061 7.14e-07 AAATGTTGAA TGGATGTGTGA AGAAAAATAT*
PF13_0061; 1092 1.21e-06 TATAACAACT CGTGTGTGTAT TTTTTTTTTC
PFL1725w; 1064 1.96e-06 CAATTTTTTA TGTATGTGTAC ACTTTTTAGT
PF13_0359; 1447 4.90e-06 AATATATATA TGTAAGTGTGT ATATATATAA*
PF14_0248; 125 1.26e-05 CACAAGAAAT TGTATATATGC GACATGTATA
PF14_0597; 1599 1.26e-05 CATATTTATA TGTATGTATAC ATTATTACAT
PF14_0373; 1203 2.26e-05 GTATAATATA TGTGTATACGA CTATAAAATA
PF10_0120; 1751 4.03e-05 TAAATTTATA TGTATATATGT GTATATATAT
PFE0225w; 431 5.42e-05 AAAATATATT AGTGTGTATAT TTTTTTTTTT*
AlignACE, mitouig4, TR---GTG-G---A, 1.0e+01 4.7e-03 5.3e-04 10, s=9
AlignACE, mitouig4, RWGWG-WGRAA,1.0e+01 2.0e-04 6.3e-03 15, s=8
Key AlignACE
#0 PFE0970w;
#1 PFE0225w;
#2 PF10_0120;
#3 PF11_0485;
#4 PF13_0061;
#5 PF13_0327;
#6 PF13_0353;
#7 PF13_0359;
#8 PF14_0373;
#9 PF14_0597;
#10 PF14_0248;
#11 PF14_0288;
#12 PF14_0721;
#13 PFL1725w;
#14 MAL13P1.47;
GGGAGGGGTGGAAA 0 1914 1*
TATTAGTGTGTATA 1 426 1*
TGGATGTGTGAAGA 5 1060 1*
TGGGTGTGTGGTAA 6 543 1*
TGTAAGTGTGTATA 7 1446 1*
TGAAAGGGCGAGAA 10 217 1*
TATCAGTGCGCATA 11 615 1
TGTATGTGTGTTGA 12 586 1*
TGTTGGTGGGAGAA 12 640 1
MEME, mitouig4, anr2, GGGGAATGAAA, w=11,s=14,llr=164,E=6.9e-004
GAGGGGTGGAA 0 1916 1*
GAGTGTTGGAC 1 238 1
ATGTGAAGGAA 1 832 1
GGATGTAGGAA 2 649 1
GTGTGAAGAAA 5 1065 1*
GAGAGTTGAAA 6 411 1
ATGAGTAGGAA 6 1125 1
GTGTGTTGAAA 12 591 1*
PF13_0353; 432 2.02e-08 AAATTCTAAA GGCGCATGAAG ATTATGATAC
PFE0970w; 1917 1.63e-07 CACATTAAGG GAGGGGTGGAA ATTAAATAAG*
PF14_0721; 645 3.47e-07 TTAAAATGTT GGTGGGAGAAA TGTAATAAGC*
PF13_0327; 897 4.28e-07 TTATATAAAT GGTGAAAGCGG AATAATTTTT
PF14_0248; 222 4.86e-07 TTTAAGTGAA AGGGCGAGAAA ATATTATACG*
PF13_0353; 544 1.01e-06 AAAATCGAAA TGGGTGTGTGG TAAATATATT*
PF13_0061; 836 1.01e-06 TTATAAGTAT AGTGAATGGCG TGTTTATGAA
PFE0970w; 714 1.15e-06 TAATATTTAT GAGGTGTGCCA ATTGTGTGAA
PF11_0485; 697 2.35e-06 ATATATAATT TGTGAACGGAG ATGTGAAAAA
PF13_0327; 1063 3.42e-06 ATGTTGAATG GATGTGTGAAG AAAAATATTT*
PF14_0288; 699 4.14e-06 TATAAATACG TGCGGATGGTA TTGACTATAT*
PFL1725w; 367 5.80e-06 TTATAAAAAT TGGGTATGAAA TATACGATAT
PF10_0120; 1634 6.79e-06 ATTAAATAAG AGTGCATGAAA AAGGAAATTT
PFE0970w; 1237 8.14e-06 TAAAGTAAAA AGGGAAAGATG CTATGTTTTA
39
Locations of motifs in upstream regions

## Slide 40
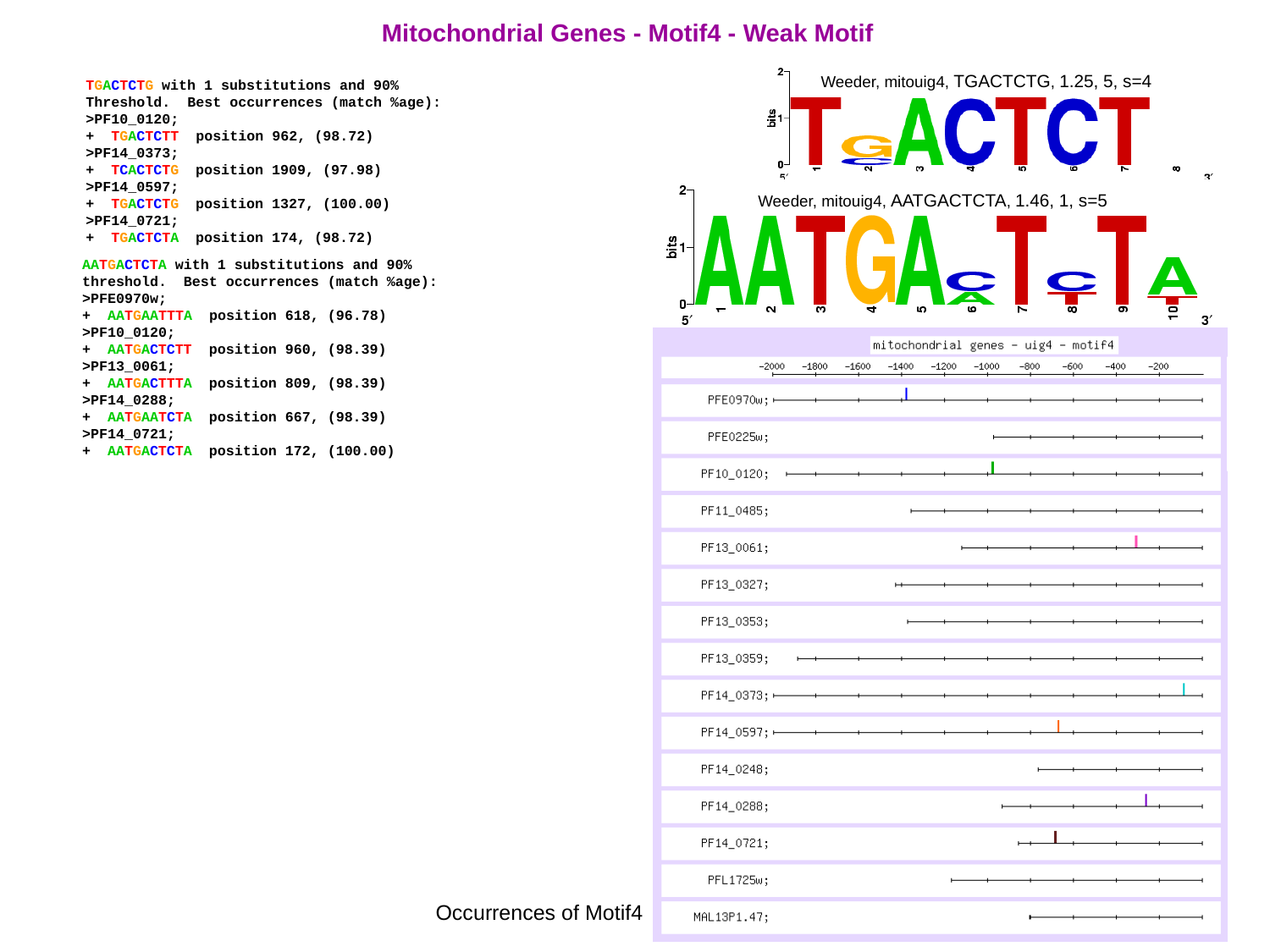

Mitochondrial Genes - Motif4 - Weak Motif
Weeder, mitouig4, TGACTCTG, 1.25, 5, s=4
TGACTCTG with 1 substitutions and 90%
Threshold. Best occurrences (match %age):
>PF10_0120;
+ TGACTCTT position 962, (98.72)
>PF14_0373;
+ TCACTCTG position 1909, (97.98)
>PF14_0597;
+ TGACTCTG position 1327, (100.00)
>PF14_0721;
+ TGACTCTA position 174, (98.72)
Weeder, mitouig4, AATGACTCTA, 1.46, 1, s=5
AATGACTCTA with 1 substitutions and 90%
threshold. Best occurrences (match %age):
>PFE0970w;
+ AATGAATTTA position 618, (96.78)
>PF10_0120;
+ AATGACTCTT position 960, (98.39)
>PF13_0061;
+ AATGACTTTA position 809, (98.39)
>PF14_0288;
+ AATGAATCTA position 667, (98.39)
>PF14_0721;
+ AATGACTCTA position 172, (100.00)
40
Occurrences of Motif4

## Slide 41
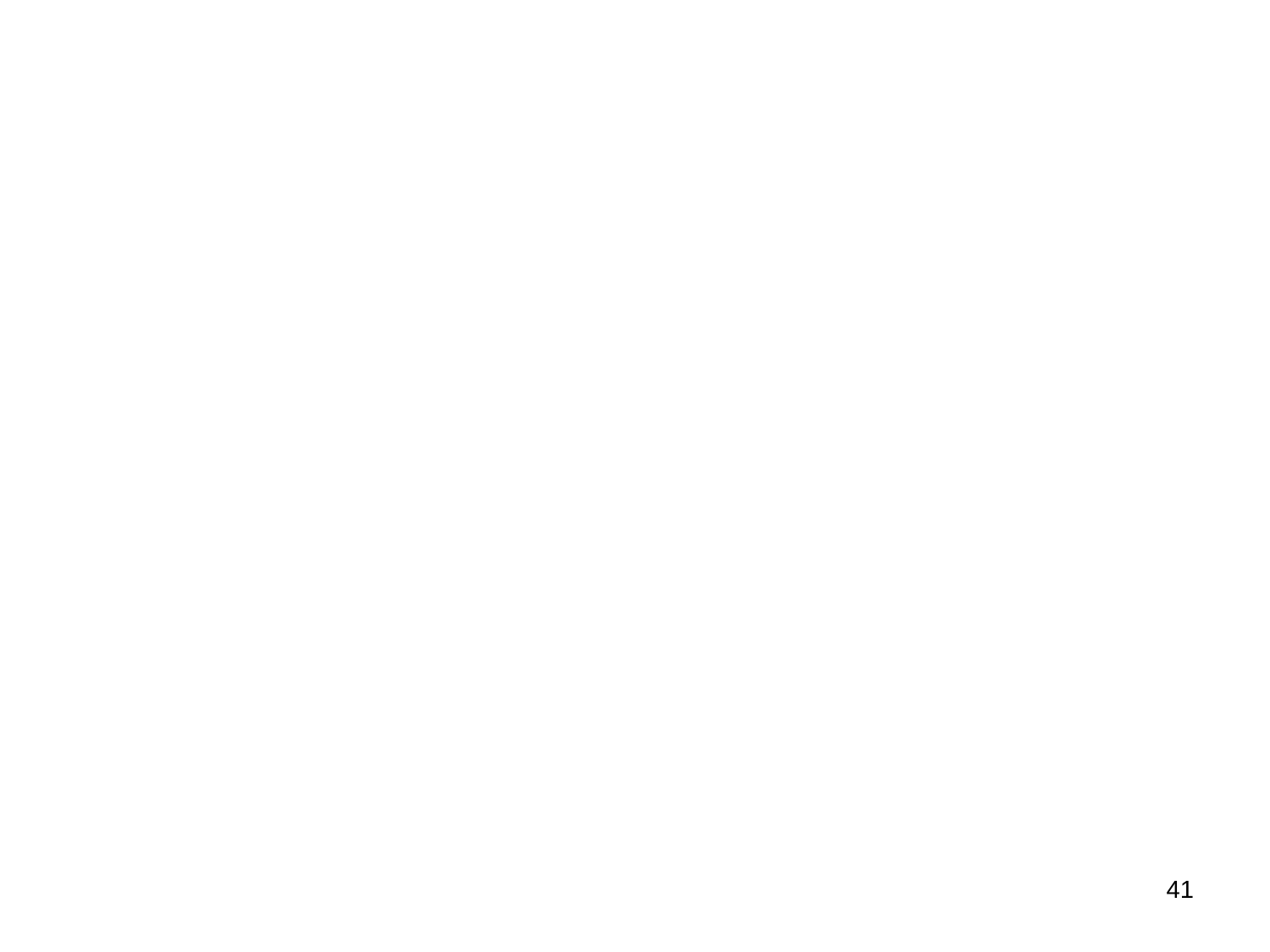

41

## Slide 42
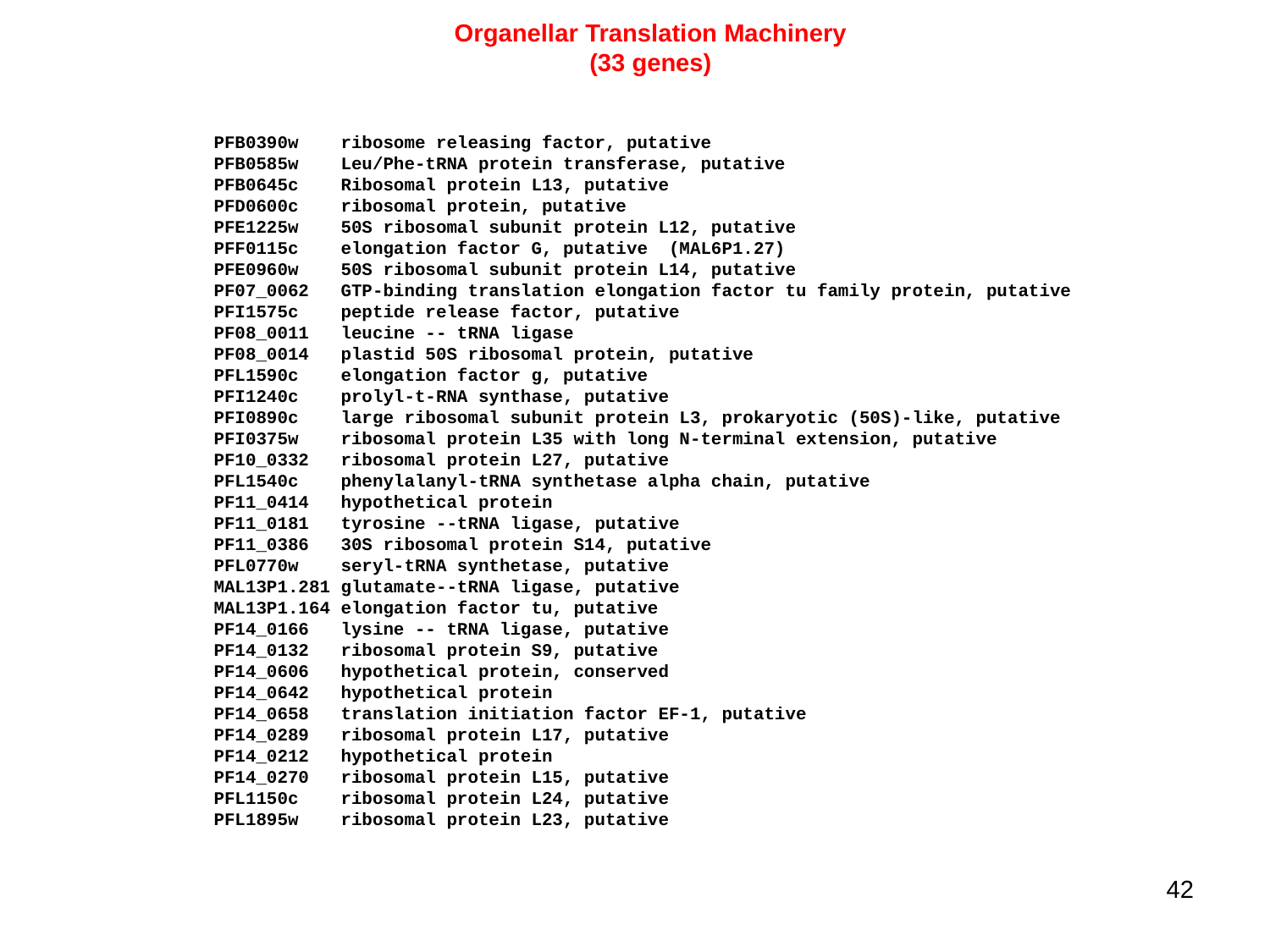

Organellar Translation Machinery
(33 genes)
PFB0390w ribosome releasing factor, putative
PFB0585w Leu/Phe-tRNA protein transferase, putative
PFB0645c Ribosomal protein L13, putative
PFD0600c ribosomal protein, putative
PFE1225w 50S ribosomal subunit protein L12, putative
PFF0115c elongation factor G, putative (MAL6P1.27)
PFE0960w 50S ribosomal subunit protein L14, putative
PF07_0062 GTP-binding translation elongation factor tu family protein, putative
PFI1575c peptide release factor, putative
PF08_0011 leucine -- tRNA ligase
PF08_0014 plastid 50S ribosomal protein, putative
PFL1590c elongation factor g, putative
PFI1240c prolyl-t-RNA synthase, putative
PFI0890c large ribosomal subunit protein L3, prokaryotic (50S)-like, putative
PFI0375w ribosomal protein L35 with long N-terminal extension, putative
PF10_0332 ribosomal protein L27, putative
PFL1540c phenylalanyl-tRNA synthetase alpha chain, putative
PF11_0414 hypothetical protein
PF11_0181 tyrosine --tRNA ligase, putative
PF11_0386 30S ribosomal protein S14, putative
PFL0770w seryl-tRNA synthetase, putative
MAL13P1.281 glutamate--tRNA ligase, putative
MAL13P1.164 elongation factor tu, putative
PF14_0166 lysine -- tRNA ligase, putative
PF14_0132 ribosomal protein S9, putative
PF14_0606 hypothetical protein, conserved
PF14_0642 hypothetical protein
PF14_0658 translation initiation factor EF-1, putative
PF14_0289 ribosomal protein L17, putative
PF14_0212 hypothetical protein
PF14_0270 ribosomal protein L15, putative
PFL1150c ribosomal protein L24, putative
PFL1895w ribosomal protein L23, putative
42

## Slide 43
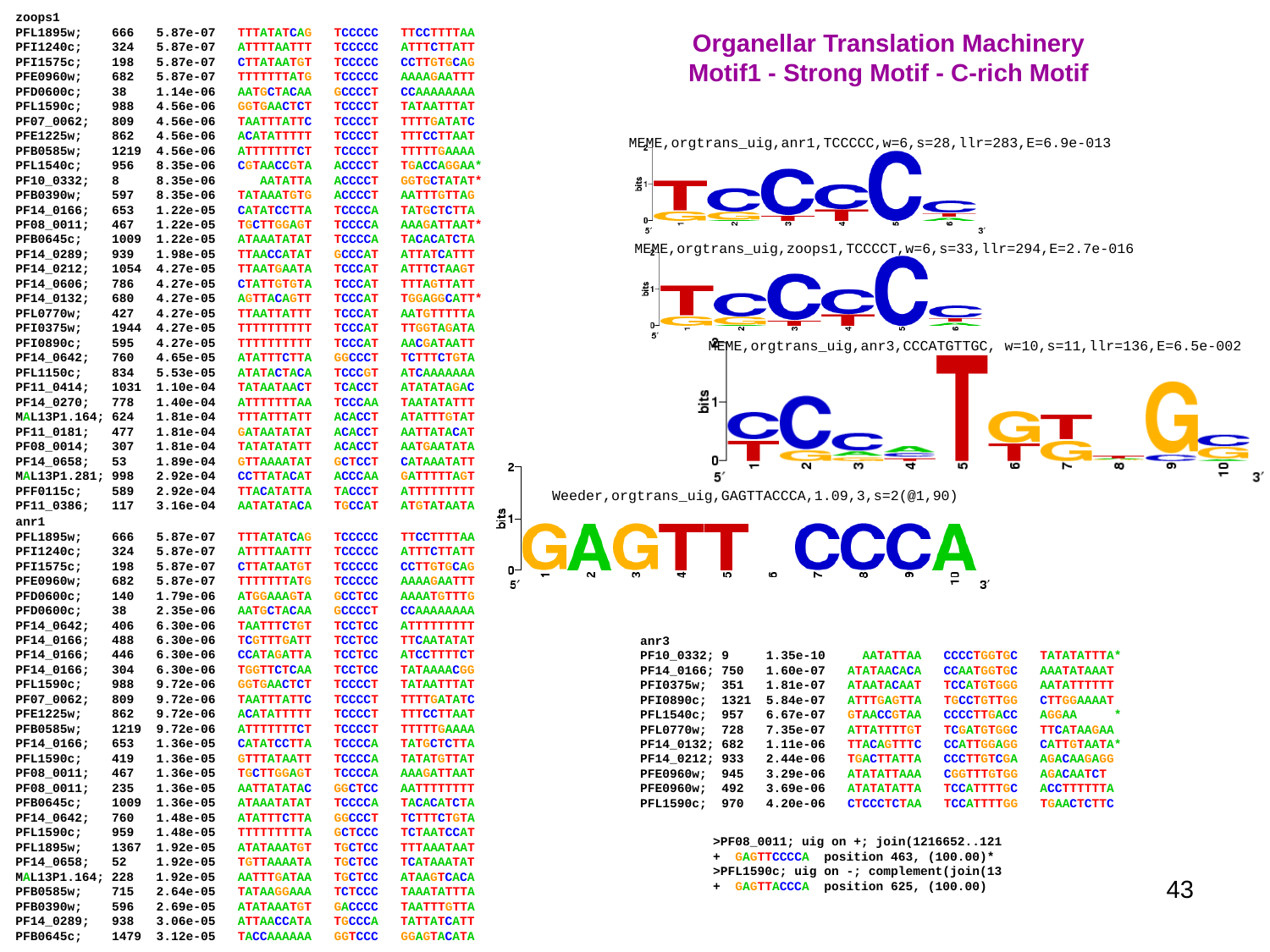

zoops1
PFL1895w; 666 5.87e-07 TTTATATCAG TCCCCC TTCCTTTTAA
PFI1240c; 324 5.87e-07 ATTTTAATTT TCCCCC ATTTCTTATT
PFI1575c; 198 5.87e-07 CTTATAATGT TCCCCC CCTTGTGCAG
PFE0960w; 682 5.87e-07 TTTTTTTATG TCCCCC AAAAGAATTT
PFD0600c; 38 1.14e-06 AATGCTACAA GCCCCT CCAAAAAAAA
PFL1590c; 988 4.56e-06 GGTGAACTCT TCCCCT TATAATTTAT
PF07_0062; 809 4.56e-06 TAATTTATTC TCCCCT TTTTGATATC
PFE1225w; 862 4.56e-06 ACATATTTTT TCCCCT TTTCCTTAAT
PFB0585w; 1219 4.56e-06 ATTTTTTTCT TCCCCT TTTTTGAAAA
PFL1540c; 956 8.35e-06 CGTAACCGTA ACCCCT TGACCAGGAA*
PF10_0332; 8 8.35e-06 AATATTA ACCCCT GGTGCTATAT*
PFB0390w; 597 8.35e-06 TATAAATGTG ACCCCT AATTTGTTAG
PF14_0166; 653 1.22e-05 CATATCCTTA TCCCCA TATGCTCTTA
PF08_0011; 467 1.22e-05 TGCTTGGAGT TCCCCA AAAGATTAAT*
PFB0645c; 1009 1.22e-05 ATAAATATAT TCCCCA TACACATCTA
PF14_0289; 939 1.98e-05 TTAACCATAT GCCCAT ATTATCATTT
PF14_0212; 1054 4.27e-05 TTAATGAATA TCCCAT ATTTCTAAGT
PF14_0606; 786 4.27e-05 CTATTGTGTA TCCCAT TTTAGTTATT
PF14_0132; 680 4.27e-05 AGTTACAGTT TCCCAT TGGAGGCATT*
PFL0770w; 427 4.27e-05 TTAATTATTT TCCCAT AATGTTTTTA
PFI0375w; 1944 4.27e-05 TTTTTTTTTT TCCCAT TTGGTAGATA
PFI0890c; 595 4.27e-05 TTTTTTTTTT TCCCAT AACGATAATT
PF14_0642; 760 4.65e-05 ATATTTCTTA GGCCCT TCTTTCTGTA
PFL1150c; 834 5.53e-05 ATATACTACA TCCCGT ATCAAAAAAA
PF11_0414; 1031 1.10e-04 TATAATAACT TCACCT ATATATAGAC
PF14_0270; 778 1.40e-04 ATTTTTTTAA TCCCAA TAATATATTT
MAL13P1.164; 624 1.81e-04 TTTATTTATT ACACCT ATATTTGTAT
PF11_0181; 477 1.81e-04 GATAATATAT ACACCT AATTATACAT
PF08_0014; 307 1.81e-04 TATATATATT ACACCT AATGAATATA
PF14_0658; 53 1.89e-04 GTTAAAATAT GCTCCT CATAAATATT
MAL13P1.281; 998 2.92e-04 CCTTATACAT ACCCAA GATTTTTAGT
PFF0115c; 589 2.92e-04 TTACATATTA TACCCT ATTTTTTTTT
PF11_0386; 117 3.16e-04 AATATATACA TGCCAT ATGTATAATA
Organellar Translation Machinery
Motif1 - Strong Motif - C-rich Motif
MEME,orgtrans_uig,anr1,TCCCCC,w=6,s=28,llr=283,E=6.9e-013
MEME,orgtrans_uig,zoops1,TCCCCT,w=6,s=33,llr=294,E=2.7e-016
MEME,orgtrans_uig,anr3,CCCATGTTGC, w=10,s=11,llr=136,E=6.5e-002
Weeder,orgtrans_uig,GAGTTACCCA,1.09,3,s=2(@1,90)
anr1
PFL1895w; 666 5.87e-07 TTTATATCAG TCCCCC TTCCTTTTAA
PFI1240c; 324 5.87e-07 ATTTTAATTT TCCCCC ATTTCTTATT
PFI1575c; 198 5.87e-07 CTTATAATGT TCCCCC CCTTGTGCAG
PFE0960w; 682 5.87e-07 TTTTTTTATG TCCCCC AAAAGAATTT
PFD0600c; 140 1.79e-06 ATGGAAAGTA GCCTCC AAAATGTTTG
PFD0600c; 38 2.35e-06 AATGCTACAA GCCCCT CCAAAAAAAA
PF14_0642; 406 6.30e-06 TAATTTCTGT TCCTCC ATTTTTTTTT
PF14_0166; 488 6.30e-06 TCGTTTGATT TCCTCC TTCAATATAT
PF14_0166; 446 6.30e-06 CCATAGATTA TCCTCC ATCCTTTTCT
PF14_0166; 304 6.30e-06 TGGTTCTCAA TCCTCC TATAAAACGG
PFL1590c; 988 9.72e-06 GGTGAACTCT TCCCCT TATAATTTAT
PF07_0062; 809 9.72e-06 TAATTTATTC TCCCCT TTTTGATATC
PFE1225w; 862 9.72e-06 ACATATTTTT TCCCCT TTTCCTTAAT
PFB0585w; 1219 9.72e-06 ATTTTTTTCT TCCCCT TTTTTGAAAA
PF14_0166; 653 1.36e-05 CATATCCTTA TCCCCA TATGCTCTTA
PFL1590c; 419 1.36e-05 GTTTATAATT TCCCCA TATATGTTAT
PF08_0011; 467 1.36e-05 TGCTTGGAGT TCCCCA AAAGATTAAT
PF08_0011; 235 1.36e-05 AATTATATAC GGCTCC AATTTTTTTT
PFB0645c; 1009 1.36e-05 ATAAATATAT TCCCCA TACACATCTA
PF14_0642; 760 1.48e-05 ATATTTCTTA GGCCCT TCTTTCTGTA
PFL1590c; 959 1.48e-05 TTTTTTTTTA GCTCCC TCTAATCCAT
PFL1895w; 1367 1.92e-05 ATATAAATGT TGCTCC TTTAAATAAT
PF14_0658; 52 1.92e-05 TGTTAAAATA TGCTCC TCATAAATAT
MAL13P1.164; 228 1.92e-05 AATTTGATAA TGCTCC ATAAGTCACA
PFB0585w; 715 2.64e-05 TATAAGGAAA TCTCCC TAAATATTTA
PFB0390w; 596 2.69e-05 ATATAAATGT GACCCC TAATTTGTTA
PF14_0289; 938 3.06e-05 ATTAACCATA TGCCCA TATTATCATT
PFB0645c; 1479 3.12e-05 TACCAAAAAA GGTCCC GGAGTACATA
anr3
PF10_0332; 9 1.35e-10 AATATTAA CCCCTGGTGC TATATATTTA*
PF14_0166; 750 1.60e-07 ATATAACACA CCAATGGTGC AAATATAAAT
PFI0375w; 351 1.81e-07 ATAATACAAT TCCATGTGGG AATATTTTTT
PFI0890c; 1321 5.84e-07 ATTTGAGTTA TGCCTGTTGG CTTGGAAAAT
PFL1540c; 957 6.67e-07 GTAACCGTAA CCCCTTGACC AGGAA *
PFL0770w; 728 7.35e-07 ATTATTTTGT TCGATGTGGC TTCATAAGAA
PF14_0132; 682 1.11e-06 TTACAGTTTC CCATTGGAGG CATTGTAATA*
PF14_0212; 933 2.44e-06 TGACTTATTA CCCTTGTCGA AGACAAGAGG
PFE0960w; 945 3.29e-06 ATATATTAAA CGGTTTGTGG AGACAATCT
PFE0960w; 492 3.69e-06 ATATATATTA TCCATTTTGC ACCTTTTTTA
PFL1590c; 970 4.20e-06 CTCCCTCTAA TCCATTTTGG TGAACTCTTC
>PF08_0011; uig on +; join(1216652..121
+ GAGTTCCCCA position 463, (100.00)*
>PFL1590c; uig on -; complement(join(13
+ GAGTTACCCA position 625, (100.00)
43

## Slide 44
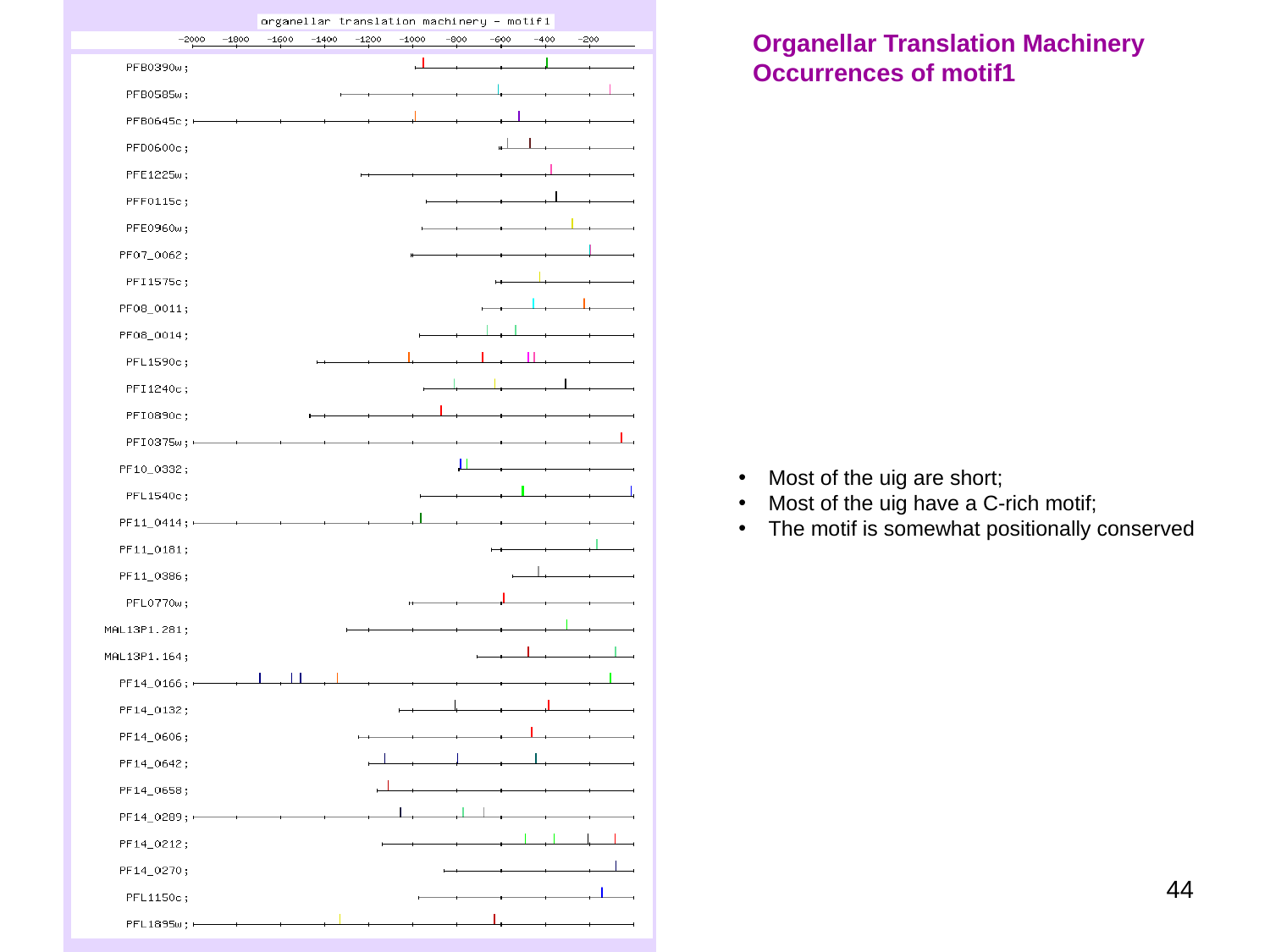

Organellar Translation Machinery
Occurrences of motif1
Most of the uig are short;
Most of the uig have a C-rich motif;
The motif is somewhat positionally conserved
44

## Slide 45
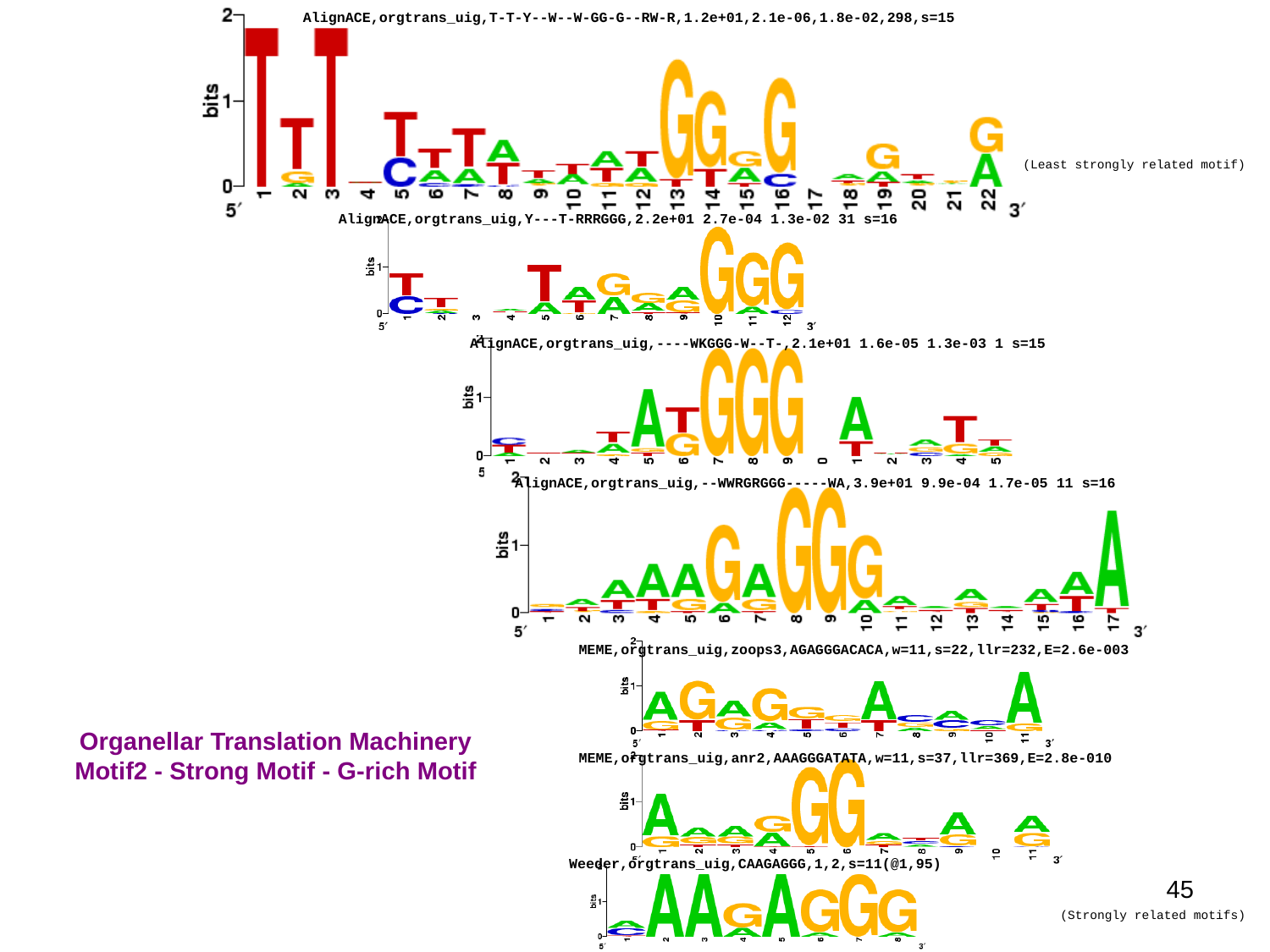

AlignACE,orgtrans_uig,T-T-Y--W--W-GG-G--RW-R,1.2e+01,2.1e-06,1.8e-02,298,s=15
AlignACE,orgtrans_uig,Y---T-RRRGGG,2.2e+01 2.7e-04 1.3e-02 31 s=16
AlignACE,orgtrans_uig,----WKGGG-W--T-,2.1e+01 1.6e-05 1.3e-03 1 s=15
AlignACE,orgtrans_uig,--WWRGRGGG-----WA,3.9e+01 9.9e-04 1.7e-05 11 s=16
MEME,orgtrans_uig,zoops3,AGAGGGACACA,w=11,s=22,llr=232,E=2.6e-003
MEME,orgtrans_uig,anr2,AAAGGGATATA,w=11,s=37,llr=369,E=2.8e-010
Weeder,orgtrans_uig,CAAGAGGG,1,2,s=11(@1,95)
(Least strongly related motif)
Organellar Translation Machinery
Motif2 - Strong Motif - G-rich Motif
45
(Strongly related motifs)

## Slide 46
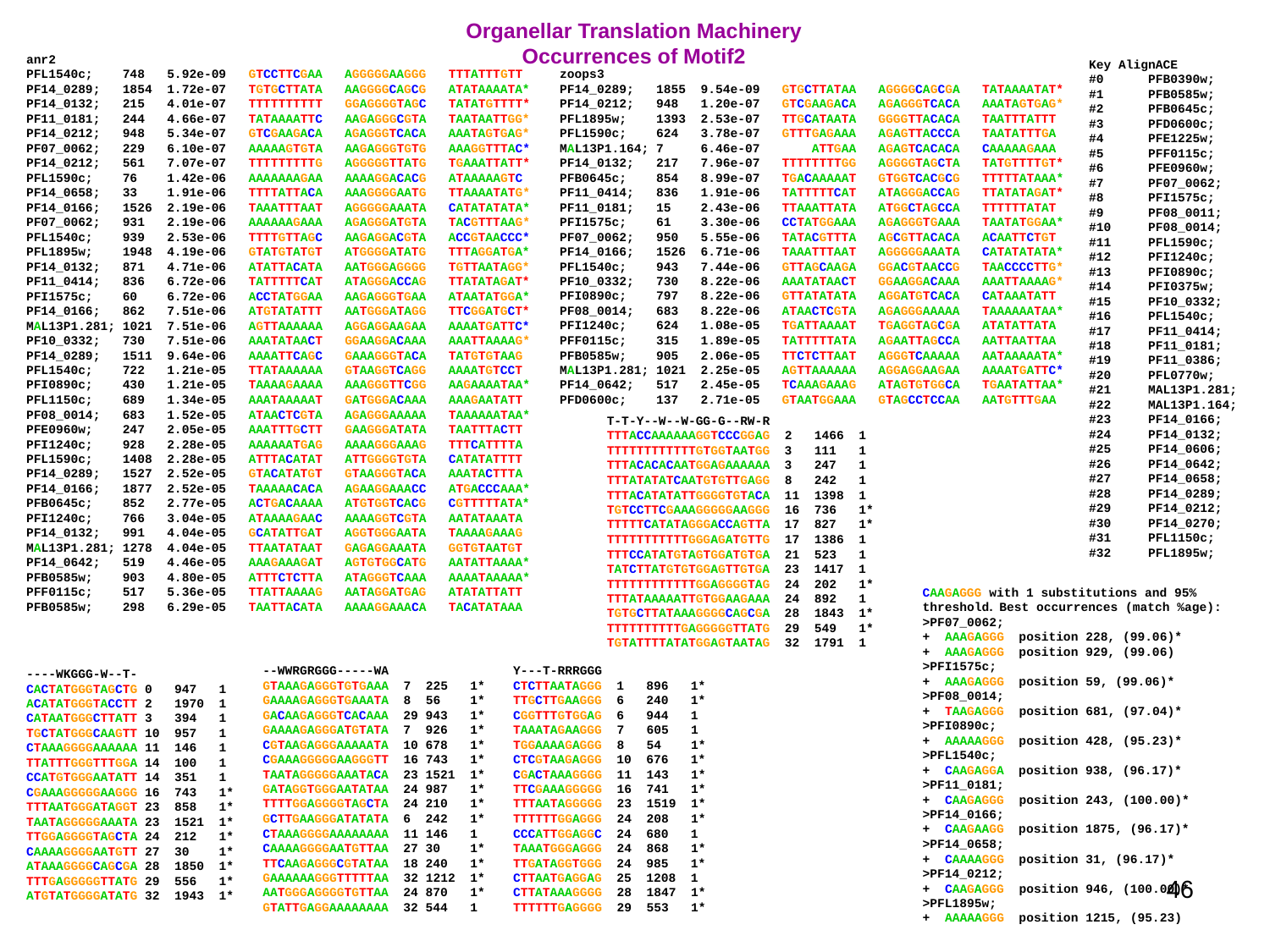

Organellar Translation Machinery
Occurrences of Motif2
anr2
PFL1540c; 748 5.92e-09 GTCCTTCGAA AGGGGGAAGGG TTTATTTGTT
PF14_0289; 1854 1.72e-07 TGTGCTTATA AAGGGGCAGCG ATATAAAATA*
PF14_0132; 215 4.01e-07 TTTTTTTTTT GGAGGGGTAGC TATATGTTTT*
PF11_0181; 244 4.66e-07 TATAAAATTC AAGAGGGCGTA TAATAATTGG*
PF14_0212; 948 5.34e-07 GTCGAAGACA AGAGGGTCACA AAATAGTGAG*
PF07_0062; 229 6.10e-07 AAAAAGTGTA AAGAGGGTGTG AAAGGTTTAC*
PF14_0212; 561 7.07e-07 TTTTTTTTTG AGGGGGTTATG TGAAATTATT*
PFL1590c; 76 1.42e-06 AAAAAAAGAA AAAAGGACACG ATAAAAAGTC
PF14_0658; 33 1.91e-06 TTTTATTACA AAAGGGGAATG TTAAAATATG*
PF14_0166; 1526 2.19e-06 TAAATTTAAT AGGGGGAAATA CATATATATA*
PF07_0062; 931 2.19e-06 AAAAAAGAAA AGAGGGATGTA TACGTTTAAG*
PFL1540c; 939 2.53e-06 TTTTGTTAGC AAGAGGACGTA ACCGTAACCC*
PFL1895w; 1948 4.19e-06 GTATGTATGT ATGGGGATATG TTTAGGATGA*
PF14_0132; 871 4.71e-06 ATATTACATA AATGGGAGGGG TGTTAATAGG*
PF11_0414; 836 6.72e-06 TATTTTTCAT ATAGGGACCAG TTATATAGAT*
PFI1575c; 60 6.72e-06 ACCTATGGAA AAGAGGGTGAA ATAATATGGA*
PF14_0166; 862 7.51e-06 ATGTATATTT AATGGGATAGG TTCGGATGCT*
MAL13P1.281; 1021 7.51e-06 AGTTAAAAAA AGGAGGAAGAA AAAATGATTC*
PF10_0332; 730 7.51e-06 AAATATAACT GGAAGGACAAA AAATTAAAAG*
PF14_0289; 1511 9.64e-06 AAAATTCAGC GAAAGGGTACA TATGTGTAAG
PFL1540c; 722 1.21e-05 TTATAAAAAA GTAAGGTCAGG AAAATGTCCT
PFI0890c; 430 1.21e-05 TAAAAGAAAA AAAGGGTTCGG AAGAAAATAA*
PFL1150c; 689 1.34e-05 AAATAAAAAT GATGGGACAAA AAAGAATATT
PF08_0014; 683 1.52e-05 ATAACTCGTA AGAGGGAAAAA TAAAAAATAA*
PFE0960w; 247 2.05e-05 AAATTTGCTT GAAGGGATATA TAATTTACTT
PFI1240c; 928 2.28e-05 AAAAAATGAG AAAAGGGAAAG TTTCATTTTA
PFL1590c; 1408 2.28e-05 ATTTACATAT ATTGGGGTGTA CATATATTTT
PF14_0289; 1527 2.52e-05 GTACATATGT GTAAGGGTACA AAATACTTTA
PF14_0166; 1877 2.52e-05 TAAAAACACA AGAAGGAAACC ATGACCCAAA*
PFB0645c; 852 2.77e-05 ACTGACAAAA ATGTGGTCACG CGTTTTTATA*
PFI1240c; 766 3.04e-05 ATAAAAGAAC AAAAGGTCGTA AATATAAATA
PF14_0132; 991 4.04e-05 GCATATTGAT AGGTGGGAATA TAAAAGAAAG
MAL13P1.281; 1278 4.04e-05 TTAATATAAT GAGAGGAAATA GGTGTAATGT
PF14_0642; 519 4.46e-05 AAAGAAAGAT AGTGTGGCATG AATATTAAAA*
PFB0585w; 903 4.80e-05 ATTTCTCTTA ATAGGGTCAAA AAAATAAAAA*
PFF0115c; 517 5.36e-05 TTATTAAAAG AATAGGATGAG ATATATTATT
PFB0585w; 298 6.29e-05 TAATTACATA AAAAGGAAACA TACATATAAA
Key AlignACE
#0 PFB0390w;
#1 PFB0585w;
#2 PFB0645c;
#3 PFD0600c;
#4 PFE1225w;
#5 PFF0115c;
#6 PFE0960w;
#7 PF07_0062;
#8 PFI1575c;
#9 PF08_0011;
#10 PF08_0014;
#11 PFL1590c;
#12 PFI1240c;
#13 PFI0890c;
#14 PFI0375w;
#15 PF10_0332;
#16 PFL1540c;
#17 PF11_0414;
#18 PF11_0181;
#19 PF11_0386;
#20 PFL0770w;
#21 MAL13P1.281;
#22 MAL13P1.164;
#23 PF14_0166;
#24 PF14_0132;
#25 PF14_0606;
#26 PF14_0642;
#27 PF14_0658;
#28 PF14_0289;
#29 PF14_0212;
#30 PF14_0270;
#31 PFL1150c;
#32 PFL1895w;
zoops3
PF14_0289; 1855 9.54e-09 GTGCTTATAA AGGGGCAGCGA TATAAAATAT*
PF14_0212; 948 1.20e-07 GTCGAAGACA AGAGGGTCACA AAATAGTGAG*
PFL1895w; 1393 2.53e-07 TTGCATAATA GGGGTTACACA TAATTTATTT
PFL1590c; 624 3.78e-07 GTTTGAGAAA AGAGTTACCCA TAATATTTGA
MAL13P1.164; 7 6.46e-07 ATTGAA AGAGTCACACA CAAAAAGAAA
PF14_0132; 217 7.96e-07 TTTTTTTTGG AGGGGTAGCTA TATGTTTTGT*
PFB0645c; 854 8.99e-07 TGACAAAAAT GTGGTCACGCG TTTTTATAAA*
PF11_0414; 836 1.91e-06 TATTTTTCAT ATAGGGACCAG TTATATAGAT*
PF11_0181; 15 2.43e-06 TTAAATTATA ATGGCTAGCCA TTTTTTATAT
PFI1575c; 61 3.30e-06 CCTATGGAAA AGAGGGTGAAA TAATATGGAA*
PF07_0062; 950 5.55e-06 TATACGTTTA AGCGTTACACA ACAATTCTGT
PF14_0166; 1526 6.71e-06 TAAATTTAAT AGGGGGAAATA CATATATATA*
PFL1540c; 943 7.44e-06 GTTAGCAAGA GGACGTAACCG TAACCCCTTG*
PF10_0332; 730 8.22e-06 AAATATAACT GGAAGGACAAA AAATTAAAAG*
PFI0890c; 797 8.22e-06 GTTATATATA AGGATGTCACA CATAAATATT
PF08_0014; 683 8.22e-06 ATAACTCGTA AGAGGGAAAAA TAAAAAATAA*
PFI1240c; 624 1.08e-05 TGATTAAAAT TGAGGTAGCGA ATATATTATA
PFF0115c; 315 1.89e-05 TATTTTTATA AGAATTAGCCA AATTAATTAA
PFB0585w; 905 2.06e-05 TTCTCTTAAT AGGGTCAAAAA AATAAAAATA*
MAL13P1.281; 1021 2.25e-05 AGTTAAAAAA AGGAGGAAGAA AAAATGATTC*
PF14_0642; 517 2.45e-05 TCAAAGAAAG ATAGTGTGGCA TGAATATTAA*
PFD0600c; 137 2.71e-05 GTAATGGAAA GTAGCCTCCAA AATGTTTGAA
T-T-Y--W--W-GG-G--RW-R
TTTACCAAAAAAGGTCCCGGAG 2 1466 1
TTTTTTTTTTTTGTGGTAATGG 3 111 1
TTTACACACAATGGAGAAAAAA 3 247 1
TTTATATATCAATGTGTTGAGG 8 242 1
TTTACATATATTGGGGTGTACA 11 1398 1
TGTCCTTCGAAAGGGGGAAGGG 16 736 1*
TTTTTCATATAGGGACCAGTTA 17 827 1*
TTTTTTTTTTTGGGAGATGTTG 17 1386 1
TTTCCATATGTAGTGGATGTGA 21 523 1
TATCTTATGTGTGGAGTTGTGA 23 1417 1
TTTTTTTTTTTTGGAGGGGTAG 24 202 1*
TTTATAAAAATTGTGGAAGAAA 24 892 1
TGTGCTTATAAAGGGGCAGCGA 28 1843 1*
TTTTTTTTTTGAGGGGGTTATG 29 549 1*
TGTATTTTATATGGAGTAATAG 32 1791 1
CAAGAGGG with 1 substitutions and 95%
threshold. Best occurrences (match %age):
>PF07_0062;
+ AAAGAGGG position 228, (99.06)*
+ AAAGAGGG position 929, (99.06)
>PFI1575c;
+ AAAGAGGG position 59, (99.06)*
>PF08_0014;
+ TAAGAGGG position 681, (97.04)*
>PFI0890c;
+ AAAAAGGG position 428, (95.23)*
>PFL1540c;
+ CAAGAGGA position 938, (96.17)*
>PF11_0181;
+ CAAGAGGG position 243, (100.00)*
>PF14_0166;
+ CAAGAAGG position 1875, (96.17)*
>PF14_0658;
+ CAAAAGGG position 31, (96.17)*
>PF14_0212;
+ CAAGAGGG position 946, (100.00)*
>PFL1895w;
+ AAAAAGGG position 1215, (95.23)
--WWRGRGGG-----WA
GTAAAGAGGGTGTGAAA 7 225 1*
GAAAAGAGGGTGAAATA 8 56 1*
GACAAGAGGGTCACAAA 29 943 1*
GAAAAGAGGGATGTATA 7 926 1*
CGTAAGAGGGAAAAATA 10 678 1*
CGAAAGGGGGAAGGGTT 16 743 1*
TAATAGGGGGAAATACA 23 1521 1*
GATAGGTGGGAATATAA 24 987 1*
TTTTGGAGGGGTAGCTA 24 210 1*
GCTTGAAGGGATATATA 6 242 1*
CTAAAGGGGAAAAAAAA 11 146 1
CAAAAGGGGAATGTTAA 27 30 1*
TTCAAGAGGGCGTATAA 18 240 1*
GAAAAAAGGGTTTTTAA 32 1212 1*
AATGGGAGGGGTGTTAA 24 870 1*
GTATTGAGGAAAAAAAA 32 544 1
Y---T-RRRGGG
CTCTTAATAGGG 1 896 1*
TTGCTTGAAGGG 6 240 1*
CGGTTTGTGGAG 6 944 1
TAAATAGAAGGG 7 605 1
TGGAAAAGAGGG 8 54 1*
CTCGTAAGAGGG 10 676 1*
CGACTAAAGGGG 11 143 1*
TTCGAAAGGGGG 16 741 1*
TTTAATAGGGGG 23 1519 1*
TTTTTTGGAGGG 24 208 1*
CCCATTGGAGGC 24 680 1
TAAATGGGAGGG 24 868 1*
TTGATAGGTGGG 24 985 1*
CTTAATGAGGAG 25 1208 1
CTTATAAAGGGG 28 1847 1*
TTTTTTGAGGGG 29 553 1*
----WKGGG-W--T-
CACTATGGGTAGCTG 0 947 1
ACATATGGGTACCTT 2 1970 1
CATAATGGGCTTATT 3 394 1
TGCTATGGGCAAGTT 10 957 1
CTAAAGGGGAAAAAA 11 146 1
TTATTTGGGTTTGGA 14 100 1
CCATGTGGGAATATT 14 351 1
CGAAAGGGGGAAGGG 16 743 1*
TTTAATGGGATAGGT 23 858 1*
TAATAGGGGGAAATA 23 1521 1*
TTGGAGGGGTAGCTA 24 212 1*
CAAAAGGGGAATGTT 27 30 1*
ATAAAGGGGCAGCGA 28 1850 1*
TTTGAGGGGGTTATG 29 556 1*
ATGTATGGGGATATG 32 1943 1*
46

## Slide 47
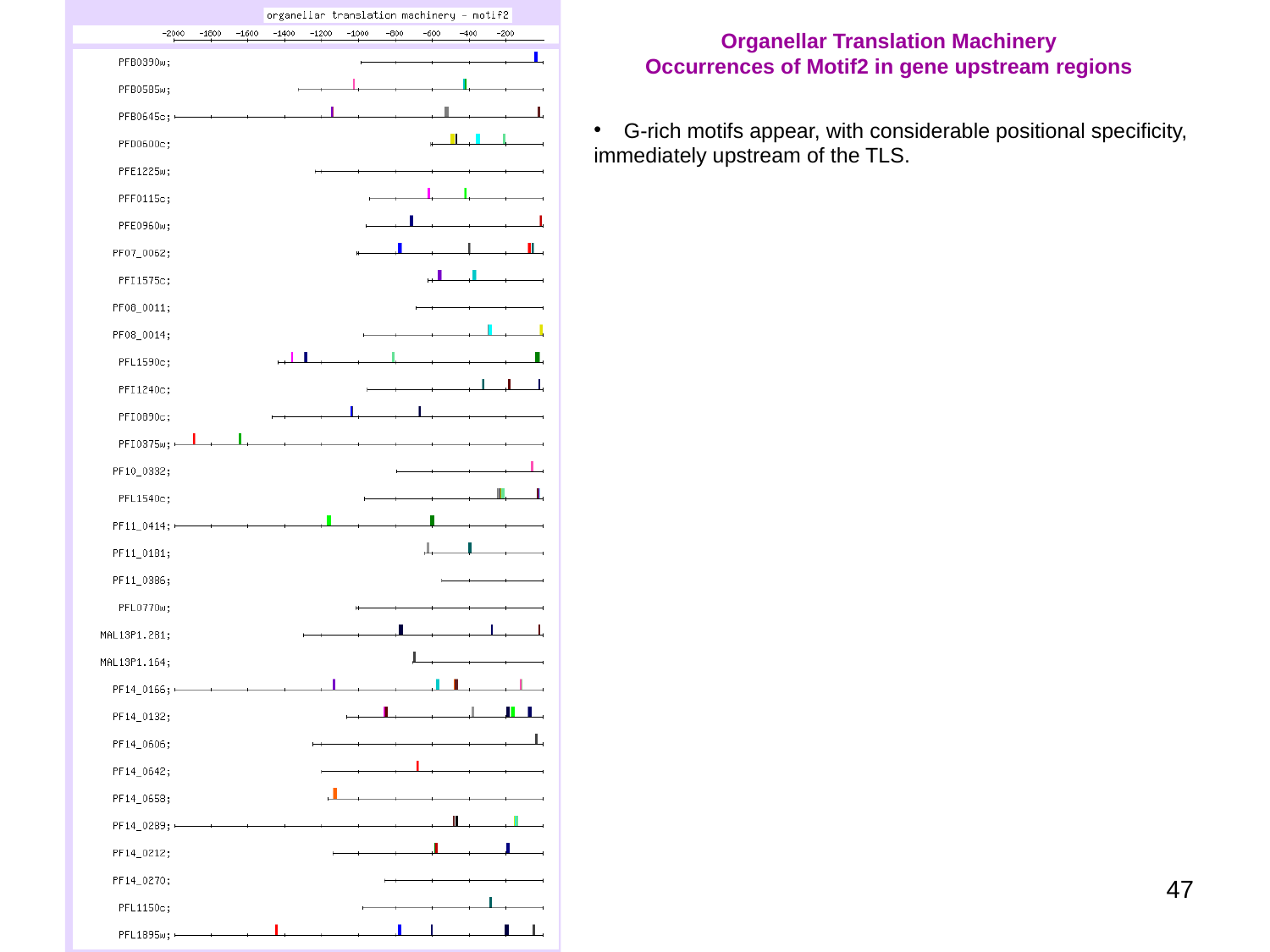

Organellar Translation Machinery
Occurrences of Motif2 in gene upstream regions
G-rich motifs appear, with considerable positional specificity,
immediately upstream of the TLS.
47

## Slide 48
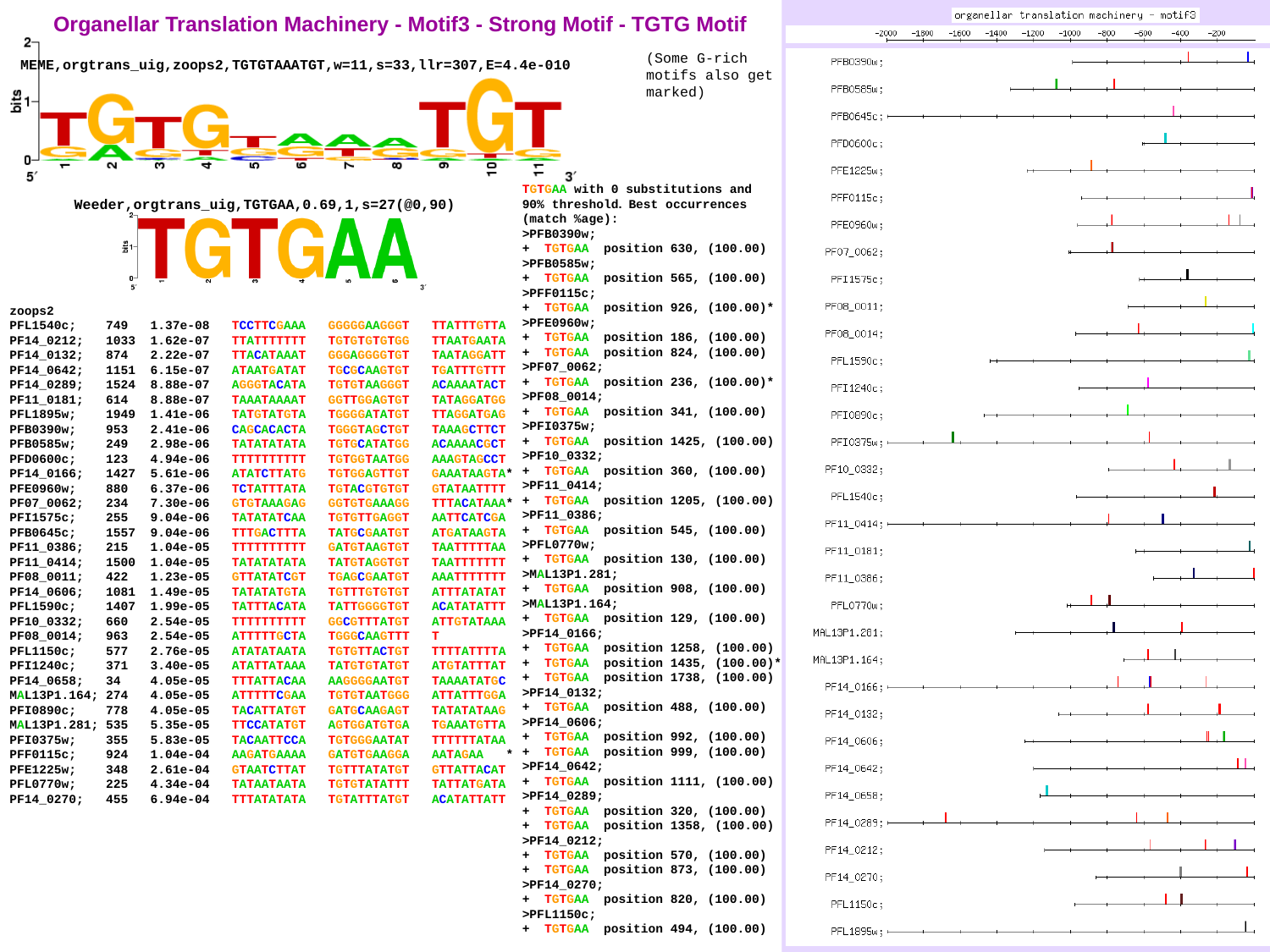

Organellar Translation Machinery - Motif3 - Strong Motif - TGTG Motif
MEME,orgtrans_uig,zoops2,TGTGTAAATGT,w=11,s=33,llr=307,E=4.4e-010
Weeder,orgtrans_uig,TGTGAA,0.69,1,s=27(@0,90)
(Some G-rich
motifs also get
marked)
TGTGAA with 0 substitutions and
90% threshold. Best occurrences
(match %age):
>PFB0390w;
+ TGTGAA position 630, (100.00)
>PFB0585w;
+ TGTGAA position 565, (100.00)
>PFF0115c;
+ TGTGAA position 926, (100.00)*
>PFE0960w;
+ TGTGAA position 186, (100.00)
+ TGTGAA position 824, (100.00)
>PF07_0062;
+ TGTGAA position 236, (100.00)*
>PF08_0014;
+ TGTGAA position 341, (100.00)
>PFI0375w;
+ TGTGAA position 1425, (100.00)
>PF10_0332;
+ TGTGAA position 360, (100.00)
>PF11_0414;
+ TGTGAA position 1205, (100.00)
>PF11_0386;
+ TGTGAA position 545, (100.00)
>PFL0770w;
+ TGTGAA position 130, (100.00)
>MAL13P1.281;
+ TGTGAA position 908, (100.00)
>MAL13P1.164;
+ TGTGAA position 129, (100.00)
>PF14_0166;
+ TGTGAA position 1258, (100.00)
+ TGTGAA position 1435, (100.00)*
+ TGTGAA position 1738, (100.00)
>PF14_0132;
+ TGTGAA position 488, (100.00)
>PF14_0606;
+ TGTGAA position 992, (100.00)
+ TGTGAA position 999, (100.00)
>PF14_0642;
+ TGTGAA position 1111, (100.00)
>PF14_0289;
+ TGTGAA position 320, (100.00)
+ TGTGAA position 1358, (100.00)
>PF14_0212;
+ TGTGAA position 570, (100.00)
+ TGTGAA position 873, (100.00)
>PF14_0270;
+ TGTGAA position 820, (100.00)
>PFL1150c;
+ TGTGAA position 494, (100.00)
zoops2
PFL1540c; 749 1.37e-08 TCCTTCGAAA GGGGGAAGGGT TTATTTGTTA
PF14_0212; 1033 1.62e-07 TTATTTTTTT TGTGTGTGTGG TTAATGAATA
PF14_0132; 874 2.22e-07 TTACATAAAT GGGAGGGGTGT TAATAGGATT
PF14_0642; 1151 6.15e-07 ATAATGATAT TGCGCAAGTGT TGATTTGTTT
PF14_0289; 1524 8.88e-07 AGGGTACATA TGTGTAAGGGT ACAAAATACT
PF11_0181; 614 8.88e-07 TAAATAAAAT GGTTGGAGTGT TATAGGATGG
PFL1895w; 1949 1.41e-06 TATGTATGTA TGGGGATATGT TTAGGATGAG
PFB0390w; 953 2.41e-06 CAGCACACTA TGGGTAGCTGT TAAAGCTTCT
PFB0585w; 249 2.98e-06 TATATATATA TGTGCATATGG ACAAAACGCT
PFD0600c; 123 4.94e-06 TTTTTTTTTT TGTGGTAATGG AAAGTAGCCT
PF14_0166; 1427 5.61e-06 ATATCTTATG TGTGGAGTTGT GAAATAAGTA*
PFE0960w; 880 6.37e-06 TCTATTTATA TGTACGTGTGT GTATAATTTT
PF07_0062; 234 7.30e-06 GTGTAAAGAG GGTGTGAAAGG TTTACATAAA*
PFI1575c; 255 9.04e-06 TATATATCAA TGTGTTGAGGT AATTCATCGA
PFB0645c; 1557 9.04e-06 TTTGACTTTA TATGCGAATGT ATGATAAGTA
PF11_0386; 215 1.04e-05 TTTTTTTTTT GATGTAAGTGT TAATTTTTAA
PF11_0414; 1500 1.04e-05 TATATATATA TATGTAGGTGT TAATTTTTTT
PF08_0011; 422 1.23e-05 GTTATATCGT TGAGCGAATGT AAATTTTTTT
PF14_0606; 1081 1.49e-05 TATATATGTA TGTTTGTGTGT ATTTATATAT
PFL1590c; 1407 1.99e-05 TATTTACATA TATTGGGGTGT ACATATATTT
PF10_0332; 660 2.54e-05 TTTTTTTTTT GGCGTTTATGT ATTGTATAAA
PF08_0014; 963 2.54e-05 ATTTTTGCTA TGGGCAAGTTT T
PFL1150c; 577 2.76e-05 ATATATAATA TGTGTTACTGT TTTTATTTTA
PFI1240c; 371 3.40e-05 ATATTATAAA TATGTGTATGT ATGTATTTAT
PF14_0658; 34 4.05e-05 TTTATTACAA AAGGGGAATGT TAAAATATGC
MAL13P1.164; 274 4.05e-05 ATTTTTCGAA TGTGTAATGGG ATTATTTGGA
PFI0890c; 778 4.05e-05 TACATTATGT GATGCAAGAGT TATATATAAG
MAL13P1.281; 535 5.35e-05 TTCCATATGT AGTGGATGTGA TGAAATGTTA
PFI0375w; 355 5.83e-05 TACAATTCCA TGTGGGAATAT TTTTTTATAA
PFF0115c; 924 1.04e-04 AAGATGAAAA GATGTGAAGGA AATAGAA *
PFE1225w; 348 2.61e-04 GTAATCTTAT TGTTTATATGT GTTATTACAT
PFL0770w; 225 4.34e-04 TATAATAATA TGTGTATATTT TATTATGATA
PF14_0270; 455 6.94e-04 TTTATATATA TGTATTTATGT ACATATTATT
48

## Slide 49
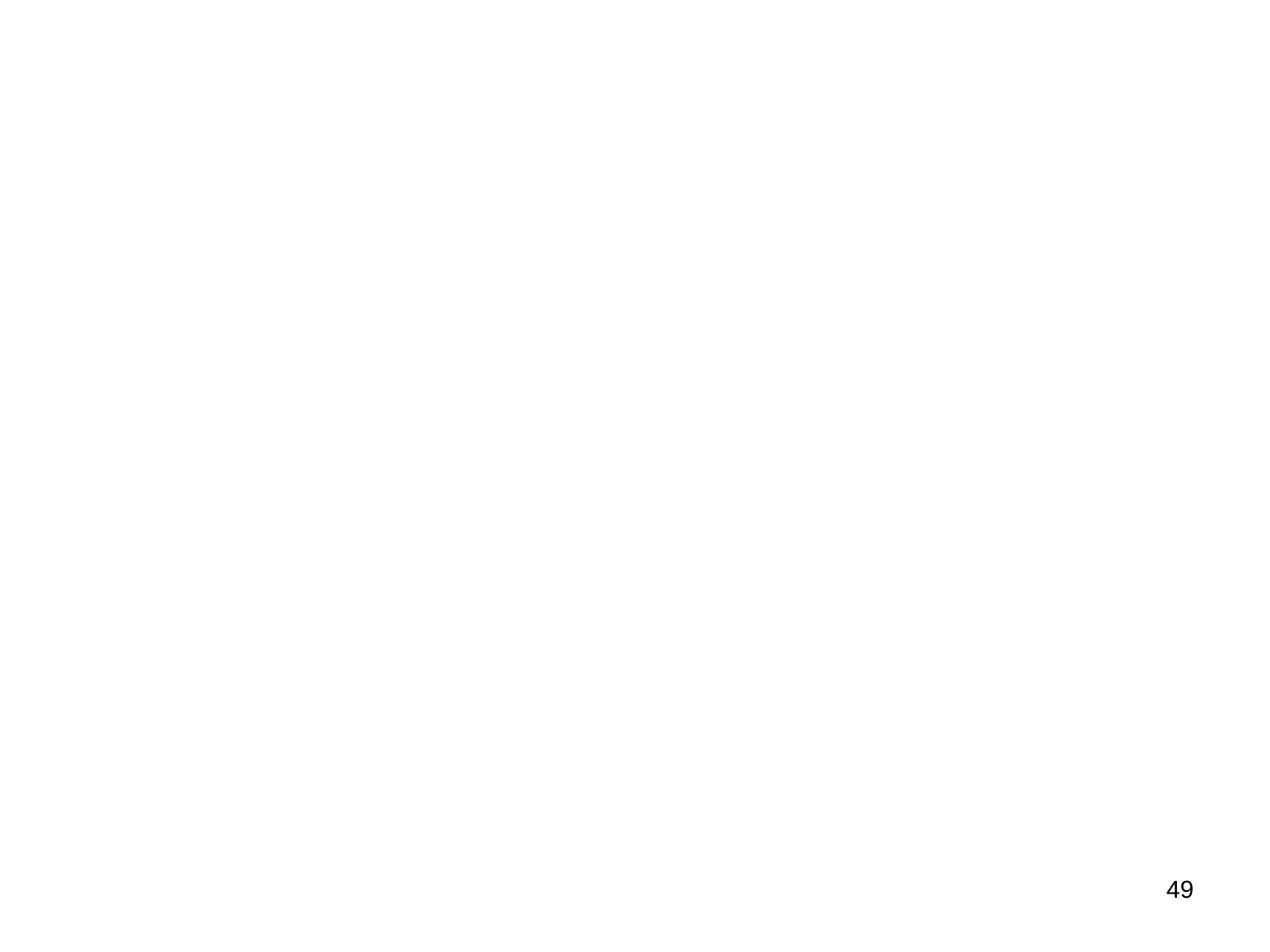

49
