# Supplementary material for: Identification of putative regulatory motifs in the upstream regions of co-expressed functional groups of genes in Plasmodium falciparum
Source: BMC Genomics. 2009 Jan 13;10:18. doi: 10.1186/1471-2164-10-18 (PMC2662883; doi:10.1186/1471-2164-10-18)
Supplement: Additional file 3 — Over-represented upstream motifs identified for the 2 functional groups of genes expressed during the mid- and late-schizont stages. Strong and weak motif groups identified for each functional group are given. [file 1471-2164-10-18-S3.ppt]

## Slide 1
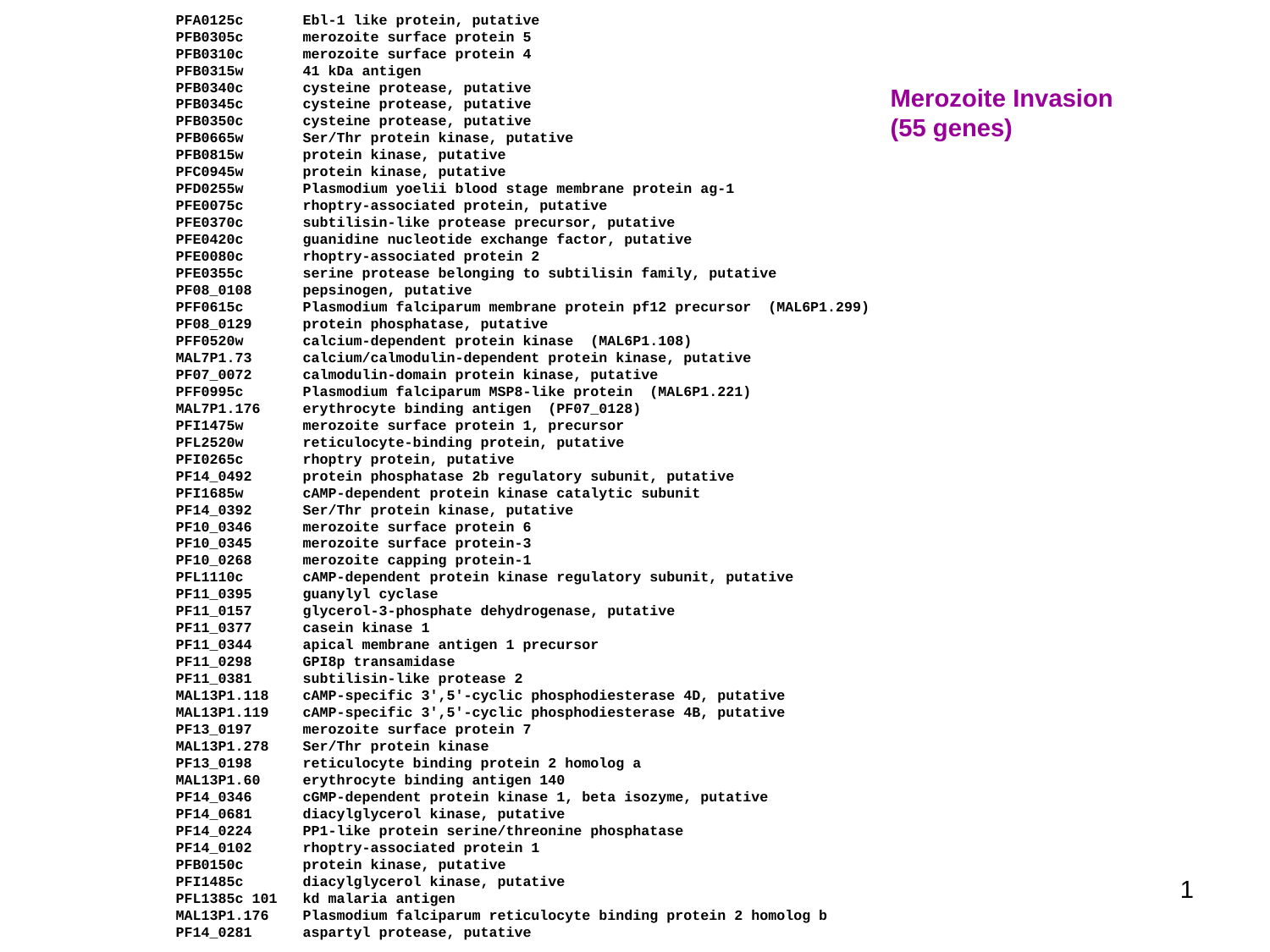

PFA0125c 	Ebl-1 like protein, putative
PFB0305c 	merozoite surface protein 5
PFB0310c 	merozoite surface protein 4
PFB0315w 	41 kDa antigen
PFB0340c 	cysteine protease, putative
PFB0345c 	cysteine protease, putative
PFB0350c 	cysteine protease, putative
PFB0665w 	Ser/Thr protein kinase, putative
PFB0815w 	protein kinase, putative
PFC0945w 	protein kinase, putative
PFD0255w 	Plasmodium yoelii blood stage membrane protein ag-1
PFE0075c 	rhoptry-associated protein, putative
PFE0370c 	subtilisin-like protease precursor, putative
PFE0420c 	guanidine nucleotide exchange factor, putative
PFE0080c 	rhoptry-associated protein 2
PFE0355c 	serine protease belonging to subtilisin family, putative
PF08_0108 	pepsinogen, putative
PFF0615c 	Plasmodium falciparum membrane protein pf12 precursor (MAL6P1.299)
PF08_0129 	protein phosphatase, putative
PFF0520w 	calcium-dependent protein kinase (MAL6P1.108)
MAL7P1.73 	calcium/calmodulin-dependent protein kinase, putative
PF07_0072 	calmodulin-domain protein kinase, putative
PFF0995c 	Plasmodium falciparum MSP8-like protein (MAL6P1.221)
MAL7P1.176 	erythrocyte binding antigen (PF07_0128)
PFI1475w 	merozoite surface protein 1, precursor
PFL2520w 	reticulocyte-binding protein, putative
PFI0265c 	rhoptry protein, putative
PF14_0492 	protein phosphatase 2b regulatory subunit, putative
PFI1685w 	cAMP-dependent protein kinase catalytic subunit
PF14_0392 	Ser/Thr protein kinase, putative
PF10_0346 	merozoite surface protein 6
PF10_0345 	merozoite surface protein-3
PF10_0268 	merozoite capping protein-1
PFL1110c 	cAMP-dependent protein kinase regulatory subunit, putative
PF11_0395 	guanylyl cyclase
PF11_0157 	glycerol-3-phosphate dehydrogenase, putative
PF11_0377 	casein kinase 1
PF11_0344 	apical membrane antigen 1 precursor
PF11_0298 	GPI8p transamidase
PF11_0381 	subtilisin-like protease 2
MAL13P1.118 	cAMP-specific 3',5'-cyclic phosphodiesterase 4D, putative
MAL13P1.119 	cAMP-specific 3',5'-cyclic phosphodiesterase 4B, putative
PF13_0197 	merozoite surface protein 7
MAL13P1.278 	Ser/Thr protein kinase
PF13_0198 	reticulocyte binding protein 2 homolog a
MAL13P1.60 	erythrocyte binding antigen 140
PF14_0346 	cGMP-dependent protein kinase 1, beta isozyme, putative
PF14_0681 	diacylglycerol kinase, putative
PF14_0224 	PP1-like protein serine/threonine phosphatase
PF14_0102 	rhoptry-associated protein 1
PFB0150c 	protein kinase, putative
PFI1485c 	diacylglycerol kinase, putative
PFL1385c 101 	kd malaria antigen
MAL13P1.176 	Plasmodium falciparum reticulocyte binding protein 2 homolog b
PF14_0281 	aspartyl protease, putative
Merozoite Invasion
(55 genes)
1

## Slide 2
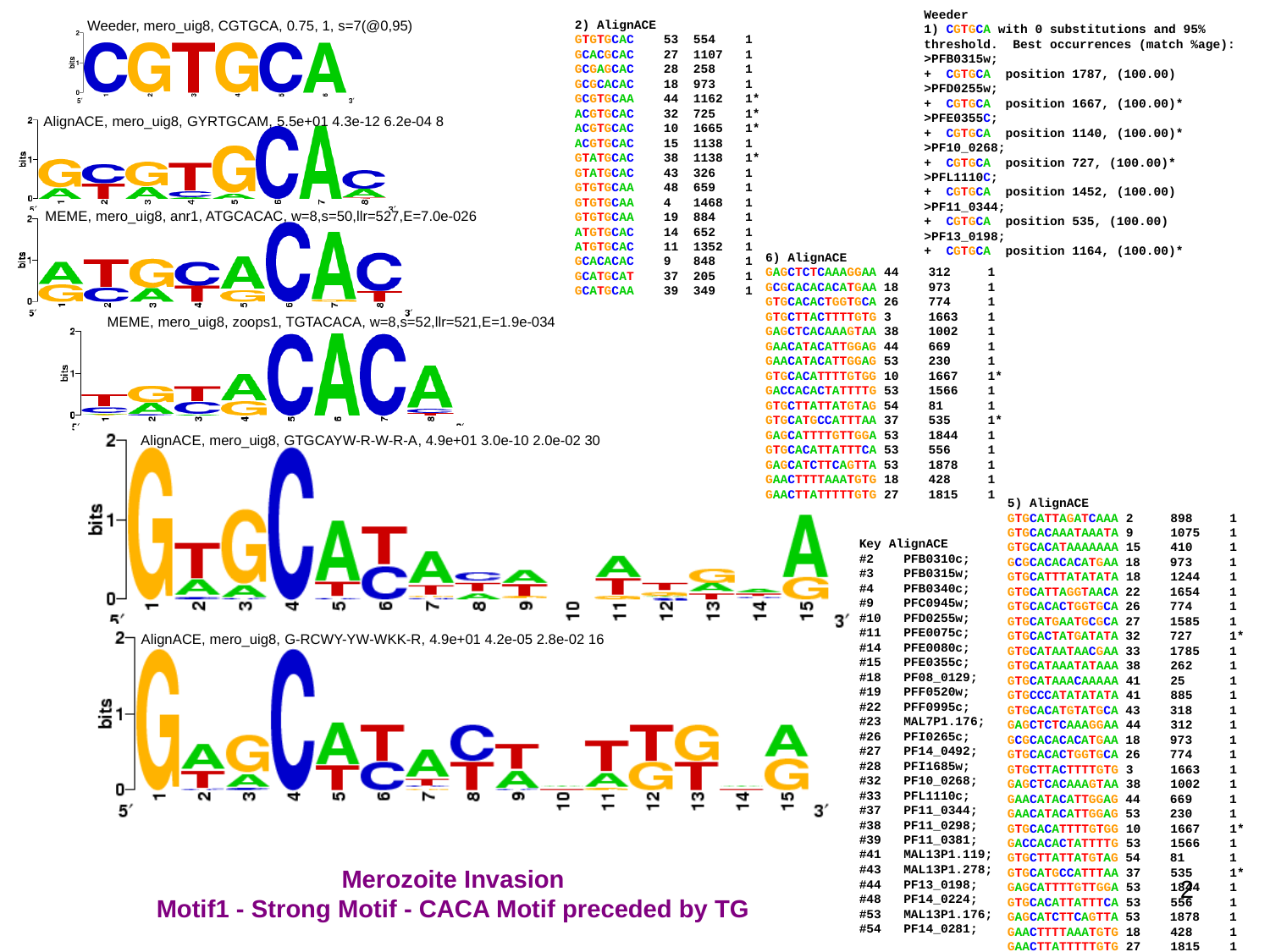

Weeder
1) CGTGCA with 0 substitutions and 95%
threshold. Best occurrences (match %age):
>PFB0315w;
+ CGTGCA position 1787, (100.00)
>PFD0255w;
+ CGTGCA position 1667, (100.00)*
>PFE0355C;
+ CGTGCA position 1140, (100.00)*
>PF10_0268;
+ CGTGCA position 727, (100.00)*
>PFL1110C;
+ CGTGCA position 1452, (100.00)
>PF11_0344;
+ CGTGCA position 535, (100.00)
>PF13_0198;
+ CGTGCA position 1164, (100.00)*
2) AlignACE
GTGTGCAC 53 554 1
GCACGCAC 27 1107 1
GCGAGCAC 28 258 1
GCGCACAC 18 973 1
GCGTGCAA 44 1162 1*
ACGTGCAC 32 725 1*
ACGTGCAC 10 1665 1*
ACGTGCAC 15 1138 1
GTATGCAC 38 1138 1*
GTATGCAC 43 326 1
GTGTGCAA 48 659 1
GTGTGCAA 4 1468 1
GTGTGCAA 19 884 1
ATGTGCAC 14 652 1
ATGTGCAC 11 1352 1
GCACACAC 9 848 1
GCATGCAT 37 205 1
GCATGCAA 39 349 1
Weeder, mero_uig8, CGTGCA, 0.75, 1, s=7(@0,95)
AlignACE, mero_uig8, GYRTGCAM, 5.5e+01 4.3e-12 6.2e-04 8
MEME, mero_uig8, anr1, ATGCACAC, w=8,s=50,llr=527,E=7.0e-026
6) AlignACE
GAGCTCTCAAAGGAA 44 312 1
GCGCACACACATGAA 18 973 1
GTGCACACTGGTGCA 26 774 1
GTGCTTACTTTTGTG 3 1663 1
GAGCTCACAAAGTAA 38 1002 1
GAACATACATTGGAG 44 669 1
GAACATACATTGGAG 53 230 1
GTGCACATTTTGTGG 10 1667 1*
GACCACACTATTTTG 53 1566 1
GTGCTTATTATGTAG 54 81 1
GTGCATGCCATTTAA 37 535 1*
GAGCATTTTGTTGGA 53 1844 1
GTGCACATTATTTCA 53 556 1
GAGCATCTTCAGTTA 53 1878 1
GAACTTTTAAATGTG 18 428 1
GAACTTATTTTTGTG 27 1815 1
MEME, mero_uig8, zoops1, TGTACACA, w=8,s=52,llr=521,E=1.9e-034
AlignACE, mero_uig8, GTGCAYW-R-W-R-A, 4.9e+01 3.0e-10 2.0e-02 30
5) AlignACE
GTGCATTAGATCAAA 2 898 1
GTGCACAAATAAATA 9 1075 1
GTGCACATAAAAAAA 15 410 1
GCGCACACACATGAA 18 973 1
GTGCATTTATATATA 18 1244 1
GTGCATTAGGTAACA 22 1654 1
GTGCACACTGGTGCA 26 774 1
GTGCATGAATGCGCA 27 1585 1
GTGCACTATGATATA 32 727 1*
GTGCATAATAACGAA 33 1785 1
GTGCATAAATATAAA 38 262 1
GTGCATAAACAAAAA 41 25 1
GTGCCCATATATATA 41 885 1
GTGCACATGTATGCA 43 318 1
GAGCTCTCAAAGGAA 44 312 1
GCGCACACACATGAA 18 973 1
GTGCACACTGGTGCA 26 774 1
GTGCTTACTTTTGTG 3 1663 1
GAGCTCACAAAGTAA 38 1002 1
GAACATACATTGGAG 44 669 1
GAACATACATTGGAG 53 230 1
GTGCACATTTTGTGG 10 1667 1*
GACCACACTATTTTG 53 1566 1
GTGCTTATTATGTAG 54 81 1
GTGCATGCCATTTAA 37 535 1*
GAGCATTTTGTTGGA 53 1844 1
GTGCACATTATTTCA 53 556 1
GAGCATCTTCAGTTA 53 1878 1
GAACTTTTAAATGTG 18 428 1
GAACTTATTTTTGTG 27 1815 1
Key AlignACE
#2 PFB0310c;
#3 PFB0315w;
#4 PFB0340c;
#9 PFC0945w;
#10 PFD0255w;
#11 PFE0075c;
#14 PFE0080c;
#15 PFE0355c;
#18 PF08_0129;
#19 PFF0520w;
#22 PFF0995c;
#23 MAL7P1.176;
#26 PFI0265c;
#27 PF14_0492;
#28 PFI1685w;
#32 PF10_0268;
#33 PFL1110c;
#37 PF11_0344;
#38 PF11_0298;
#39 PF11_0381;
#41 MAL13P1.119;
#43 MAL13P1.278;
#44 PF13_0198;
#48 PF14_0224;
#53 MAL13P1.176;
#54 PF14_0281;
AlignACE, mero_uig8, G-RCWY-YW-WKK-R, 4.9e+01 4.2e-05 2.8e-02 16
Merozoite Invasion
Motif1 - Strong Motif - CACA Motif preceded by TG
2

## Slide 3
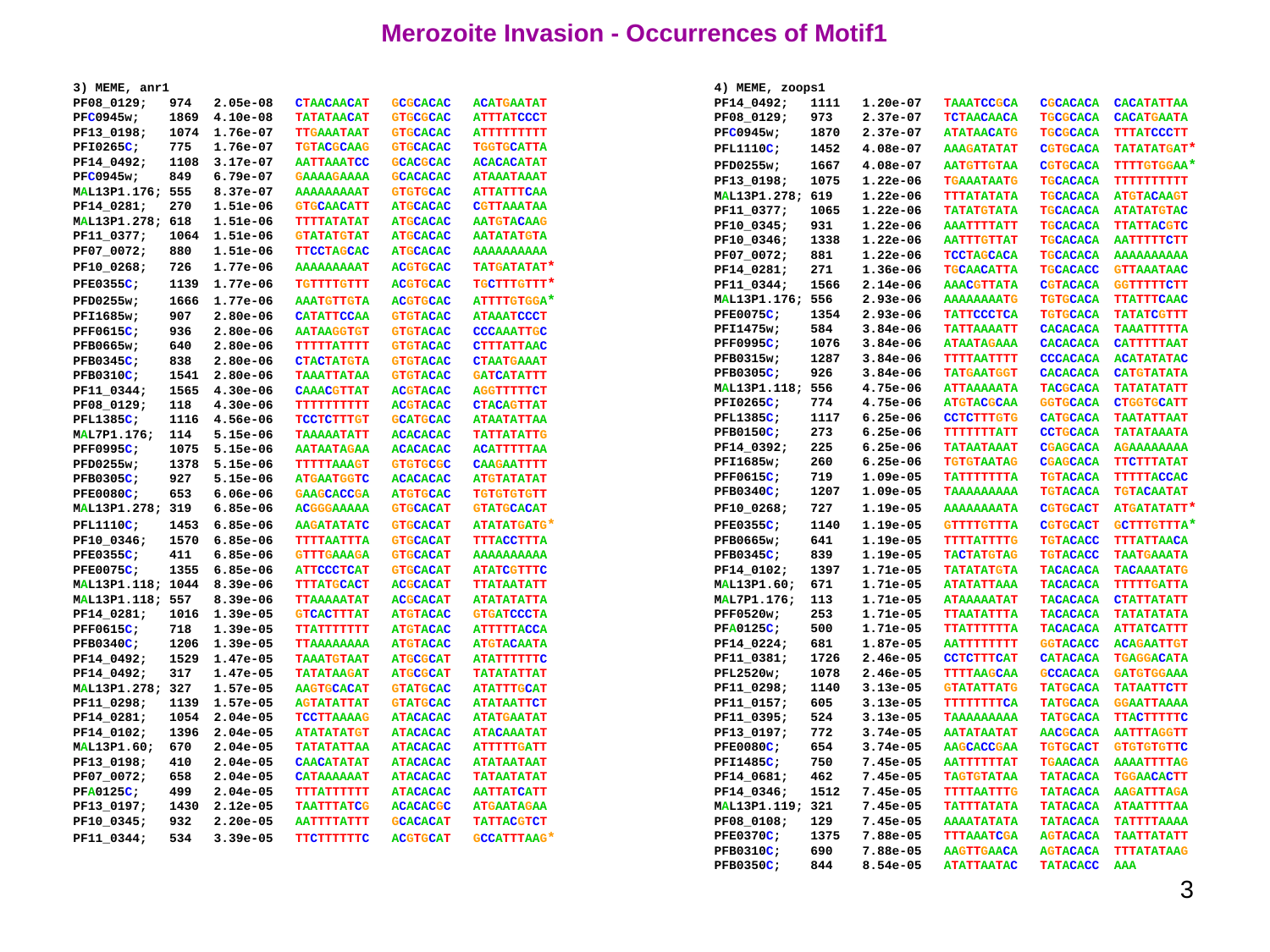

Merozoite Invasion - Occurrences of Motif1
4) MEME, zoops1
PF14_0492; 1111 1.20e-07 TAAATCCGCA CGCACACA CACATATTAA
PF08_0129; 973 2.37e-07 TCTAACAACA TGCGCACA CACATGAATA
PFC0945w; 1870 2.37e-07 ATATAACATG TGCGCACA TTTATCCCTT
PFL1110C; 1452 4.08e-07 AAAGATATAT CGTGCACA TATATATGAT*
PFD0255w; 1667 4.08e-07 AATGTTGTAA CGTGCACA TTTTGTGGAA*
PF13_0198; 1075 1.22e-06 TGAAATAATG TGCACACA TTTTTTTTTT
MAL13P1.278; 619 1.22e-06 TTTATATATA TGCACACA ATGTACAAGT
PF11_0377; 1065 1.22e-06 TATATGTATA TGCACACA ATATATGTAC
PF10_0345; 931 1.22e-06 AAATTTTATT TGCACACA TTATTACGTC
PF10_0346; 1338 1.22e-06 AATTTGTTAT TGCACACA AATTTTTCTT
PF07_0072; 881 1.22e-06 TCCTAGCACA TGCACACA AAAAAAAAAA
PF14_0281; 271 1.36e-06 TGCAACATTA TGCACACC GTTAAATAAC
PF11_0344; 1566 2.14e-06 AAACGTTATA CGTACACA GGTTTTTCTT
MAL13P1.176; 556 2.93e-06 AAAAAAAATG TGTGCACA TTATTTCAAC
PFE0075C; 1354 2.93e-06 TATTCCCTCA TGTGCACA TATATCGTTT
PFI1475w; 584 3.84e-06 TATTAAAATT CACACACA TAAATTTTTA
PFF0995C; 1076 3.84e-06 ATAATAGAAA CACACACA CATTTTTAAT
PFB0315w; 1287 3.84e-06 TTTTAATTTT CCCACACA ACATATATAC
PFB0305C; 926 3.84e-06 TATGAATGGT CACACACA CATGTATATA
MAL13P1.118; 556 4.75e-06 ATTAAAAATA TACGCACA TATATATATT
PFI0265C; 774 4.75e-06 ATGTACGCAA GGTGCACA CTGGTGCATT
PFL1385C; 1117 6.25e-06 CCTCTTTGTG CATGCACA TAATATTAAT
PFB0150C; 273 6.25e-06 TTTTTTTATT CCTGCACA TATATAAATA
PF14_0392; 225 6.25e-06 TATAATAAAT CGAGCACA AGAAAAAAAA
PFI1685w; 260 6.25e-06 TGTGTAATAG CGAGCACA TTCTTTATAT
PFF0615C; 719 1.09e-05 TATTTTTTTA TGTACACA TTTTTACCAC
PFB0340C; 1207 1.09e-05 TAAAAAAAAA TGTACACA TGTACAATAT
PF10_0268; 727 1.19e-05 AAAAAAAATA CGTGCACT ATGATATATT*
PFE0355C; 1140 1.19e-05 GTTTTGTTTA CGTGCACT GCTTTGTTTA*
PFB0665w; 641 1.19e-05 TTTTATTTTG TGTACACC TTTATTAACA
PFB0345C; 839 1.19e-05 TACTATGTAG TGTACACC TAATGAAATA
PF14_0102; 1397 1.71e-05 TATATATGTA TACACACA TACAAATATG
MAL13P1.60; 671 1.71e-05 ATATATTAAA TACACACA TTTTTGATTA
MAL7P1.176; 113 1.71e-05 ATAAAAATAT TACACACA CTATTATATT
PFF0520w; 253 1.71e-05 TTAATATTTA TACACACA TATATATATA
PFA0125C; 500 1.71e-05 TTATTTTTTA TACACACA ATTATCATTT
PF14_0224; 681 1.87e-05 AATTTTTTTT GGTACACC ACAGAATTGT
PF11_0381; 1726 2.46e-05 CCTCTTTCAT CATACACA TGAGGACATA
PFL2520w; 1078 2.46e-05 TTTTAAGCAA GCCACACA GATGTGGAAA
PF11_0298; 1140 3.13e-05 GTATATTATG TATGCACA TATAATTCTT
PF11_0157; 605 3.13e-05 TTTTTTTTCA TATGCACA GGAATTAAAA
PF11_0395; 524 3.13e-05 TAAAAAAAAA TATGCACA TTACTTTTTC
PF13_0197; 772 3.74e-05 AATATAATAT AACGCACA AATTTAGGTT
PFE0080C; 654 3.74e-05 AAGCACCGAA TGTGCACT GTGTGTGTTC
PFI1485C; 750 7.45e-05 AATTTTTTAT TGAACACA AAAATTTTAG
PF14_0681; 462 7.45e-05 TAGTGTATAA TATACACA TGGAACACTT
PF14_0346; 1512 7.45e-05 TTTTAATTTG TATACACA AAGATTTAGA
MAL13P1.119; 321 7.45e-05 TATTTATATA TATACACA ATAATTTTAA
PF08_0108; 129 7.45e-05 AAAATATATA TATACACA TATTTTAAAA
PFE0370C; 1375 7.88e-05 TTTAAATCGA AGTACACA TAATTATATT
PFB0310C; 690 7.88e-05 AAGTTGAACA AGTACACA TTTATATAAG
PFB0350C; 844 8.54e-05 ATATTAATAC TATACACC AAA
3) MEME, anr1
PF08_0129; 974 2.05e-08 CTAACAACAT GCGCACAC ACATGAATAT
PFC0945w; 1869 4.10e-08 TATATAACAT GTGCGCAC ATTTATCCCT
PF13_0198; 1074 1.76e-07 TTGAAATAAT GTGCACAC ATTTTTTTTT
PFI0265C; 775 1.76e-07 TGTACGCAAG GTGCACAC TGGTGCATTA
PF14_0492; 1108 3.17e-07 AATTAAATCC GCACGCAC ACACACATAT
PFC0945w; 849 6.79e-07 GAAAAGAAAA GCACACAC ATAAATAAAT
MAL13P1.176; 555 8.37e-07 AAAAAAAAAT GTGTGCAC ATTATTTCAA
PF14_0281; 270 1.51e-06 GTGCAACATT ATGCACAC CGTTAAATAA
MAL13P1.278; 618 1.51e-06 TTTTATATAT ATGCACAC AATGTACAAG
PF11_0377; 1064 1.51e-06 GTATATGTAT ATGCACAC AATATATGTA
PF07_0072; 880 1.51e-06 TTCCTAGCAC ATGCACAC AAAAAAAAAA
PF10_0268; 726 1.77e-06 AAAAAAAAAT ACGTGCAC TATGATATAT*
PFE0355C; 1139 1.77e-06 TGTTTTGTTT ACGTGCAC TGCTTTGTTT*
PFD0255w; 1666 1.77e-06 AAATGTTGTA ACGTGCAC ATTTTGTGGA*
PFI1685w; 907 2.80e-06 CATATTCCAA GTGTACAC ATAAATCCCT
PFF0615C; 936 2.80e-06 AATAAGGTGT GTGTACAC CCCAAATTGC
PFB0665w; 640 2.80e-06 TTTTTATTTT GTGTACAC CTTTATTAAC
PFB0345C; 838 2.80e-06 CTACTATGTA GTGTACAC CTAATGAAAT
PFB0310C; 1541 2.80e-06 TAAATTATAA GTGTACAC GATCATATTT
PF11_0344; 1565 4.30e-06 CAAACGTTAT ACGTACAC AGGTTTTTCT
PF08_0129; 118 4.30e-06 TTTTTTTTTT ACGTACAC CTACAGTTAT
PFL1385C; 1116 4.56e-06 TCCTCTTTGT GCATGCAC ATAATATTAA
MAL7P1.176; 114 5.15e-06 TAAAAATATT ACACACAC TATTATATTG
PFF0995C; 1075 5.15e-06 AATAATAGAA ACACACAC ACATTTTTAA
PFD0255w; 1378 5.15e-06 TTTTTAAAGT GTGTGCGC CAAGAATTTT
PFB0305C; 927 5.15e-06 ATGAATGGTC ACACACAC ATGTATATAT
PFE0080C; 653 6.06e-06 GAAGCACCGA ATGTGCAC TGTGTGTGTT
MAL13P1.278; 319 6.85e-06 ACGGGAAAAA GTGCACAT GTATGCACAT
PFL1110C; 1453 6.85e-06 AAGATATATC GTGCACAT ATATATGATG*
PF10_0346; 1570 6.85e-06 TTTTAATTTA GTGCACAT TTTACCTTTA
PFE0355C; 411 6.85e-06 GTTTGAAAGA GTGCACAT AAAAAAAAAA
PFE0075C; 1355 6.85e-06 ATTCCCTCAT GTGCACAT ATATCGTTTC
MAL13P1.118; 1044 8.39e-06 TTTATGCACT ACGCACAT TTATAATATT
MAL13P1.118; 557 8.39e-06 TTAAAAATAT ACGCACAT ATATATATTA
PF14_0281; 1016 1.39e-05 GTCACTTTAT ATGTACAC GTGATCCCTA
PFF0615C; 718 1.39e-05 TTATTTTTTT ATGTACAC ATTTTTACCA
PFB0340C; 1206 1.39e-05 TTAAAAAAAA ATGTACAC ATGTACAATA
PF14_0492; 1529 1.47e-05 TAAATGTAAT ATGCGCAT ATATTTTTTC
PF14_0492; 317 1.47e-05 TATATAAGAT ATGCGCAT TATATATTAT
MAL13P1.278; 327 1.57e-05 AAGTGCACAT GTATGCAC ATATTTGCAT
PF11_0298; 1139 1.57e-05 AGTATATTAT GTATGCAC ATATAATTCT
PF14_0281; 1054 2.04e-05 TCCTTAAAAG ATACACAC ATATGAATAT
PF14_0102; 1396 2.04e-05 ATATATATGT ATACACAC ATACAAATAT
MAL13P1.60; 670 2.04e-05 TATATATTAA ATACACAC ATTTTTGATT
PF13_0198; 410 2.04e-05 CAACATATAT ATACACAC ATATAATAAT
PF07_0072; 658 2.04e-05 CATAAAAAAT ATACACAC TATAATATAT
PFA0125C; 499 2.04e-05 TTTATTTTTT ATACACAC AATTATCATT
PF13_0197; 1430 2.12e-05 TAATTTATCG ACACACGC ATGAATAGAA
PF10_0345; 932 2.20e-05 AATTTTATTT GCACACAT TATTACGTCT
PF11_0344; 534 3.39e-05 TTCTTTTTTC ACGTGCAT GCCATTTAAG*
3

## Slide 4
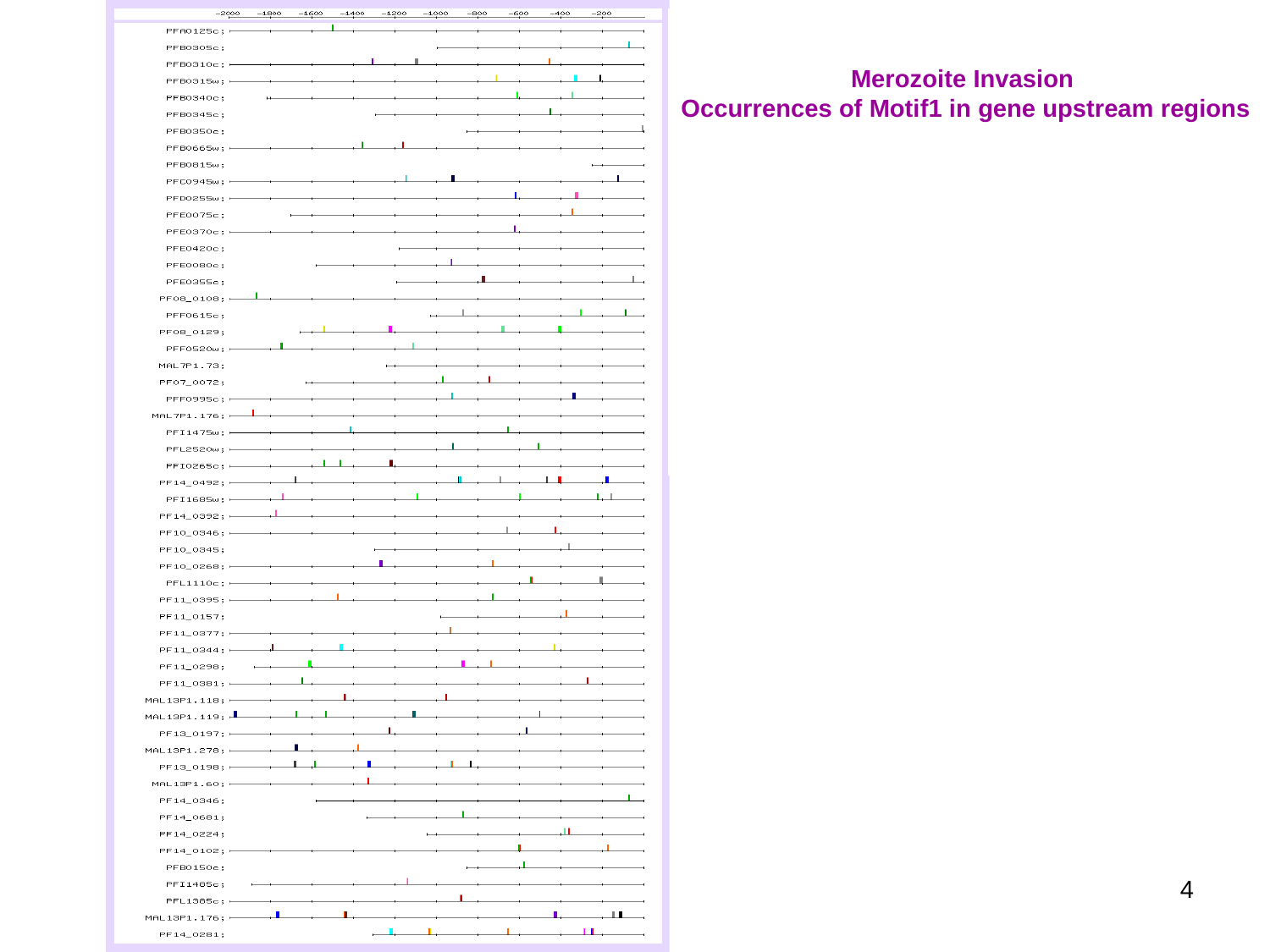

Merozoite Invasion
Occurrences of Motif1 in gene upstream regions
4

## Slide 5
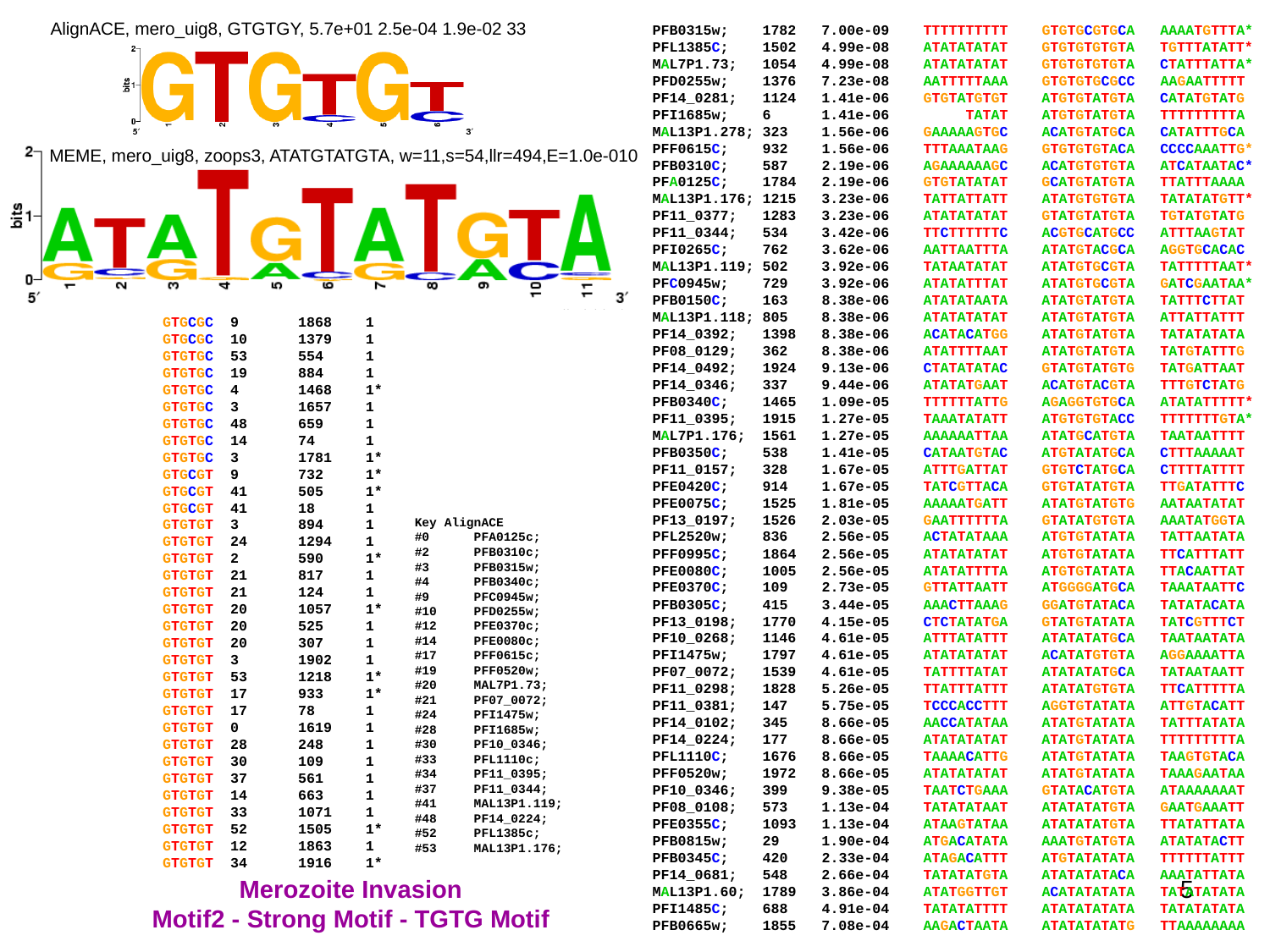

AlignACE, mero_uig8, GTGTGY, 5.7e+01 2.5e-04 1.9e-02 33
PFB0315w; 1782 7.00e-09 TTTTTTTTTT GTGTGCGTGCA AAAATGTTTA*
PFL1385C; 1502 4.99e-08 ATATATATAT GTGTGTGTGTA TGTTTATATT*
MAL7P1.73; 1054 4.99e-08 ATATATATAT GTGTGTGTGTA CTATTTATTA*
PFD0255w; 1376 7.23e-08 AATTTTTAAA GTGTGTGCGCC AAGAATTTTT
PF14_0281; 1124 1.41e-06 GTGTATGTGT ATGTGTATGTA CATATGTATG
PFI1685w; 6 1.41e-06 TATAT ATGTGTATGTA TTTTTTTTTA
MAL13P1.278; 323 1.56e-06 GAAAAAGTGC ACATGTATGCA CATATTTGCA
PFF0615C; 932 1.56e-06 TTTAAATAAG GTGTGTGTACA CCCCAAATTG*
PFB0310C; 587 2.19e-06 AGAAAAAAGC ACATGTGTGTA ATCATAATAC*
PFA0125C; 1784 2.19e-06 GTGTATATAT GCATGTATGTA TTATTTAAAA
MAL13P1.176; 1215 3.23e-06 TATTATTATT ATATGTGTGTA TATATATGTT*
PF11_0377; 1283 3.23e-06 ATATATATAT GTATGTATGTA TGTATGTATG
PF11_0344; 534 3.42e-06 TTCTTTTTTC ACGTGCATGCC ATTTAAGTAT
PFI0265C; 762 3.62e-06 AATTAATTTA ATATGTACGCA AGGTGCACAC
MAL13P1.119; 502 3.92e-06 TATAATATAT ATATGTGCGTA TATTTTTAAT*
PFC0945w; 729 3.92e-06 ATATATTTAT ATATGTGCGTA GATCGAATAA*
PFB0150C; 163 8.38e-06 ATATATAATA ATATGTATGTA TATTTCTTAT
MAL13P1.118; 805 8.38e-06 ATATATATAT ATATGTATGTA ATTATTATTT
PF14_0392; 1398 8.38e-06 ACATACATGG ATATGTATGTA TATATATATA
PF08_0129; 362 8.38e-06 ATATTTTAAT ATATGTATGTA TATGTATTTG
PF14_0492; 1924 9.13e-06 CTATATATAC GTATGTATGTG TATGATTAAT
PF14_0346; 337 9.44e-06 ATATATGAAT ACATGTACGTA TTTGTCTATG
PFB0340C; 1465 1.09e-05 TTTTTTATTG AGAGGTGTGCA ATATATTTTT*
PF11_0395; 1915 1.27e-05 TAAATATATT ATGTGTGTACC TTTTTTTGTA*
MAL7P1.176; 1561 1.27e-05 AAAAAATTAA ATATGCATGTA TAATAATTTT
PFB0350C; 538 1.41e-05 CATAATGTAC ATGTATATGCA CTTTAAAAAT
PF11_0157; 328 1.67e-05 ATTTGATTAT GTGTCTATGCA CTTTTATTTT
PFE0420C; 914 1.67e-05 TATCGTTACA GTGTATATGTA TTGATATTTC
PFE0075C; 1525 1.81e-05 AAAAATGATT ATATGTATGTG AATAATATAT
PF13_0197; 1526 2.03e-05 GAATTTTTTA GTATATGTGTA AAATATGGTA
PFL2520w; 836 2.56e-05 ACTATATAAA ATGTGTATATA TATTAATATA
PFF0995C; 1864 2.56e-05 ATATATATAT ATGTGTATATA TTCATTTATT
PFE0080C; 1005 2.56e-05 ATATATTTTA ATGTGTATATA TTACAATTAT
PFE0370C; 109 2.73e-05 GTTATTAATT ATGGGGATGCA TAAATAATTC
PFB0305C; 415 3.44e-05 AAACTTAAAG GGATGTATACA TATATACATA
PF13_0198; 1770 4.15e-05 CTCTATATGA GTATGTATATA TATCGTTTCT
PF10_0268; 1146 4.61e-05 ATTTATATTT ATATATATGCA TAATAATATA
PFI1475w; 1797 4.61e-05 ATATATATAT ACATATGTGTA AGGAAAATTA
PF07_0072; 1539 4.61e-05 TATTTTATAT ATATATATGCA TATAATAATT
PF11_0298; 1828 5.26e-05 TTATTTATTT ATATATGTGTA TTCATTTTTA
PF11_0381; 147 5.75e-05 TCCCACCTTT AGGTGTATATA ATTGTACATT
PF14_0102; 345 8.66e-05 AACCATATAA ATATGTATATA TATTTATATA
PF14_0224; 177 8.66e-05 ATATATATAT ATATGTATATA TTTTTTTTTA
PFL1110C; 1676 8.66e-05 TAAAACATTG ATATGTATATA TAAGTGTACA
PFF0520w; 1972 8.66e-05 ATATATATAT ATATGTATATA TAAAGAATAA
PF10_0346; 399 9.38e-05 TAATCTGAAA GTATACATGTA ATAAAAAAAT
PF08_0108; 573 1.13e-04 TATATATAAT ATATATATGTA GAATGAAATT
PFE0355C; 1093 1.13e-04 ATAAGTATAA ATATATATGTA TTATATTATA
PFB0815w; 29 1.90e-04 ATGACATATA AAATGTATGTA ATATATACTT
PFB0345C; 420 2.33e-04 ATAGACATTT ATGTATATATA TTTTTTATTT
PF14_0681; 548 2.66e-04 TATATATGTA ATATATATACA AAATATTATA
MAL13P1.60; 1789 3.86e-04 ATATGGTTGT ACATATATATA TATATATATA
PFI1485C; 688 4.91e-04 TATATATTTT ATATATATATA TATATATATA
PFB0665w; 1855 7.08e-04 AAGACTAATA ATATATATATG TTAAAAAAAA
MEME, mero_uig8, zoops3, ATATGTATGTA, w=11,s=54,llr=494,E=1.0e-010
GTGCGC 9 1868 1
GTGCGC 10 1379 1
GTGTGC 53 554 1
GTGTGC 19 884 1
GTGTGC 4 1468 1*
GTGTGC 3 1657 1
GTGTGC 48 659 1
GTGTGC 14 74 1
GTGTGC 3 1781 1*
GTGCGT 9 732 1*
GTGCGT 41 505 1*
GTGCGT 41 18 1
GTGTGT 3 894 1
GTGTGT 24 1294 1
GTGTGT 2 590 1*
GTGTGT 21 817 1
GTGTGT 21 124 1
GTGTGT 20 1057 1*
GTGTGT 20 525 1
GTGTGT 20 307 1
GTGTGT 3 1902 1
GTGTGT 53 1218 1*
GTGTGT 17 933 1*
GTGTGT 17 78 1
GTGTGT 0 1619 1
GTGTGT 28 248 1
GTGTGT 30 109 1
GTGTGT 37 561 1
GTGTGT 14 663 1
GTGTGT 33 1071 1
GTGTGT 52 1505 1*
GTGTGT 12 1863 1
GTGTGT 34 1916 1*
Key AlignACE
#0 PFA0125c;
#2 PFB0310c;
#3 PFB0315w;
#4 PFB0340c;
#9 PFC0945w;
#10 PFD0255w;
#12 PFE0370c;
#14 PFE0080c;
#17 PFF0615c;
#19 PFF0520w;
#20 MAL7P1.73;
#21 PF07_0072;
#24 PFI1475w;
#28 PFI1685w;
#30 PF10_0346;
#33 PFL1110c;
#34 PF11_0395;
#37 PF11_0344;
#41 MAL13P1.119;
#48 PF14_0224;
#52 PFL1385c;
#53 MAL13P1.176;
5
Merozoite Invasion
Motif2 - Strong Motif - TGTG Motif

## Slide 6
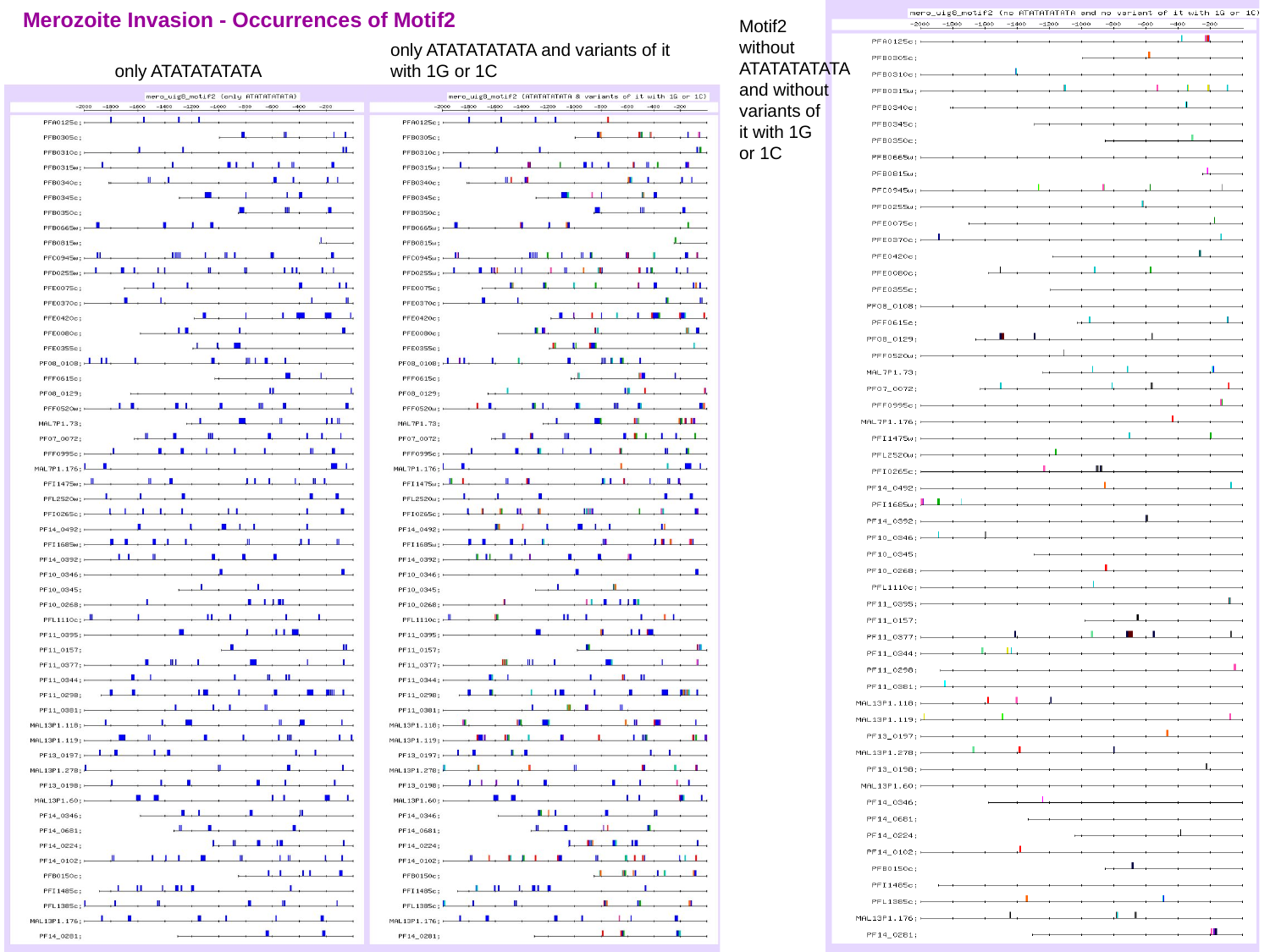

Merozoite Invasion - Occurrences of Motif2
Motif2
without
ATATATATATA
and without
variants of
it with 1G
or 1C
only ATATATATATA and variants of it
with 1G or 1C
only ATATATATATA
6

## Slide 7
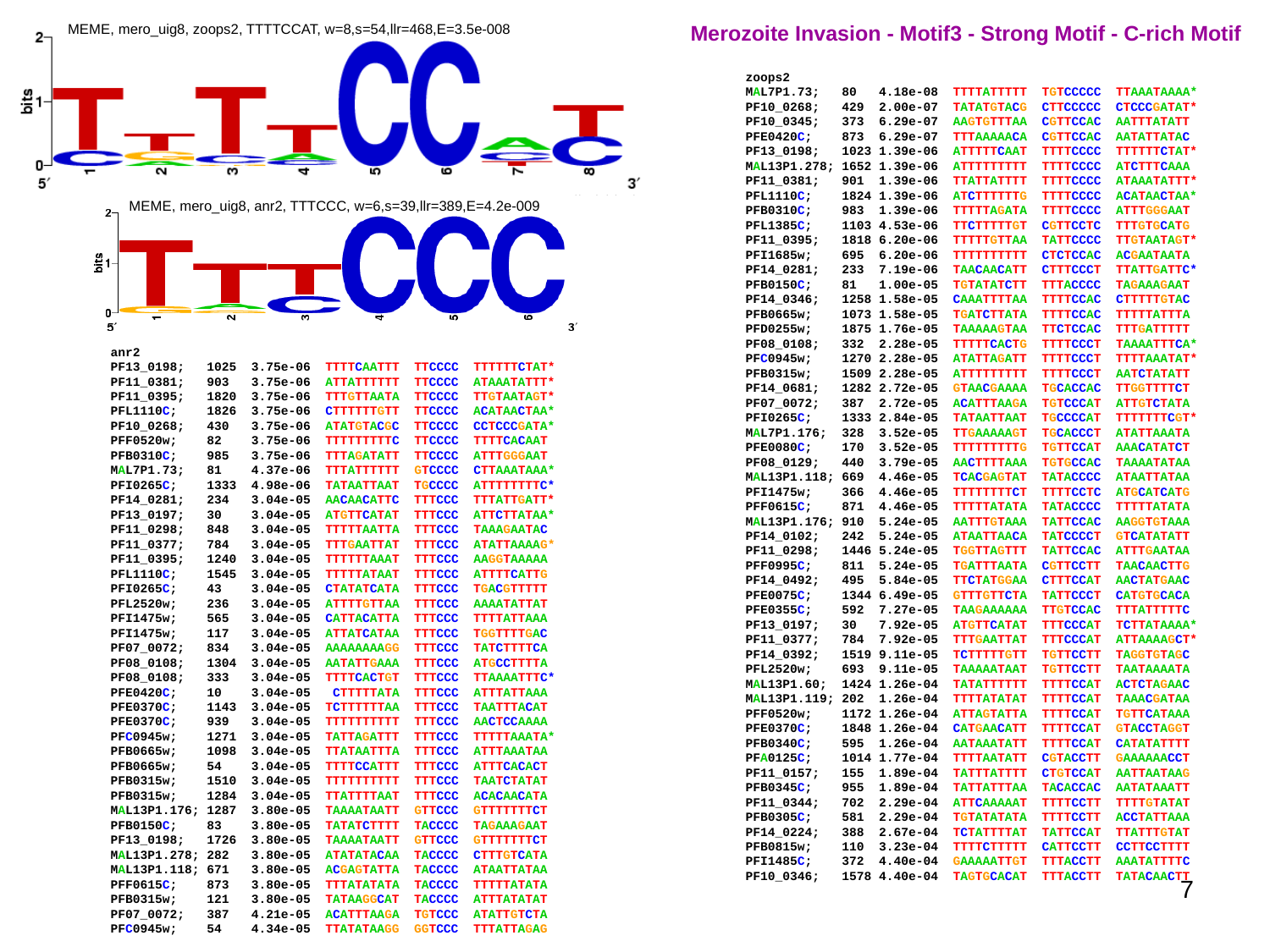

Merozoite Invasion - Motif3 - Strong Motif - C-rich Motif
MEME, mero_uig8, zoops2, TTTTCCAT, w=8,s=54,llr=468,E=3.5e-008
zoops2
MAL7P1.73; 80 4.18e-08 TTTTATTTTT TGTCCCCC TTAAATAAAA*
PF10_0268; 429 2.00e-07 TATATGTACG CTTCCCCC CTCCCGATAT*
PF10_0345; 373 6.29e-07 AAGTGTTTAA CGTTCCAC AATTTATATT
PFE0420C; 873 6.29e-07 TTTAAAAACA CGTTCCAC AATATTATAC
PF13_0198; 1023 1.39e-06 ATTTTTCAAT TTTTCCCC TTTTTTCTAT*
MAL13P1.278; 1652 1.39e-06 ATTTTTTTTT TTTTCCCC ATCTTTCAAA
PF11_0381; 901 1.39e-06 TTATTATTTT TTTTCCCC ATAAATATTT*
PFL1110C; 1824 1.39e-06 ATCTTTTTTG TTTTCCCC ACATAACTAA*
PFB0310C; 983 1.39e-06 TTTTTAGATA TTTTCCCC ATTTGGGAAT
PFL1385C; 1103 4.53e-06 TTCTTTTTGT CGTTCCTC TTTGTGCATG
PF11_0395; 1818 6.20e-06 TTTTTGTTAA TATTCCCC TTGTAATAGT*
PFI1685w; 695 6.20e-06 TTTTTTTTTT CTCTCCAC ACGAATAATA
PF14_0281; 233 7.19e-06 TAACAACATT CTTTCCCT TTATTGATTC*
PFB0150C; 81 1.00e-05 TGTATATCTT TTTACCCC TAGAAAGAAT
PF14_0346; 1258 1.58e-05 CAAATTTTAA TTTTCCAC CTTTTTGTAC
PFB0665w; 1073 1.58e-05 TGATCTTATA TTTTCCAC TTTTTATTTA
PFD0255w; 1875 1.76e-05 TAAAAAGTAA TTCTCCAC TTTGATTTTT
PF08_0108; 332 2.28e-05 TTTTTCACTG TTTTCCCT TAAAATTTCA*
PFC0945w; 1270 2.28e-05 ATATTAGATT TTTTCCCT TTTTAAATAT*
PFB0315w; 1509 2.28e-05 ATTTTTTTTT TTTTCCCT AATCTATATT
PF14_0681; 1282 2.72e-05 GTAACGAAAA TGCACCAC TTGGTTTTCT
PF07_0072; 387 2.72e-05 ACATTTAAGA TGTCCCAT ATTGTCTATA
PFI0265C; 1333 2.84e-05 TATAATTAAT TGCCCCAT TTTTTTTCGT*
MAL7P1.176; 328 3.52e-05 TTGAAAAAGT TGCACCCT ATATTAAATA
PFE0080C; 170 3.52e-05 TTTTTTTTTG TGTTCCAT AAACATATCT
PF08_0129; 440 3.79e-05 AACTTTTAAA TGTGCCAC TAAAATATAA
MAL13P1.118; 669 4.46e-05 TCACGAGTAT TATACCCC ATAATTATAA
PFI1475w; 366 4.46e-05 TTTTTTTTCT TTTTCCTC ATGCATCATG
PFF0615C; 871 4.46e-05 TTTTTATATA TATACCCC TTTTTATATA
MAL13P1.176; 910 5.24e-05 AATTTGTAAA TATTCCAC AAGGTGTAAA
PF14_0102; 242 5.24e-05 ATAATTAACA TATCCCCT GTCATATATT
PF11_0298; 1446 5.24e-05 TGGTTAGTTT TATTCCAC ATTTGAATAA
PFF0995C; 811 5.24e-05 TGATTTAATA CGTTCCTT TAACAACTTG
PF14_0492; 495 5.84e-05 TTCTATGGAA CTTTCCAT AACTATGAAC
PFE0075C; 1344 6.49e-05 GTTTGTTCTA TATTCCCT CATGTGCACA
PFE0355C; 592 7.27e-05 TAAGAAAAAA TTGTCCAC TTTATTTTTC
PF13_0197; 30 7.92e-05 ATGTTCATAT TTTCCCAT TCTTATAAAA*
PF11_0377; 784 7.92e-05 TTTGAATTAT TTTCCCAT ATTAAAAGCT*
PF14_0392; 1519 9.11e-05 TCTTTTTGTT TGTTCCTT TAGGTGTAGC
PFL2520w; 693 9.11e-05 TAAAAATAAT TGTTCCTT TAATAAAATA
MAL13P1.60; 1424 1.26e-04 TATATTTTTT TTTTCCAT ACTCTAGAAC
MAL13P1.119; 202 1.26e-04 TTTTATATAT TTTTCCAT TAAACGATAA
PFF0520w; 1172 1.26e-04 ATTAGTATTA TTTTCCAT TGTTCATAAA
PFE0370C; 1848 1.26e-04 CATGAACATT TTTTCCAT GTACCTAGGT
PFB0340C; 595 1.26e-04 AATAAATATT TTTTCCAT CATATATTTT
PFA0125C; 1014 1.77e-04 TTTTAATATT CGTACCTT GAAAAAACCT
PF11_0157; 155 1.89e-04 TATTTATTTT CTGTCCAT AATTAATAAG
PFB0345C; 955 1.89e-04 TATTATTTAA TACACCAC AATATAAATT
PF11_0344; 702 2.29e-04 ATTCAAAAAT TTTTCCTT TTTTGTATAT
PFB0305C; 581 2.29e-04 TGTATATATA TTTTCCTT ACCTATTAAA
PF14_0224; 388 2.67e-04 TCTATTTTAT TATTCCAT TTATTTGTAT
PFB0815w; 110 3.23e-04 TTTTCTTTTT CATTCCTT CCTTCCTTTT
PFI1485C; 372 4.40e-04 GAAAAATTGT TTTACCTT AAATATTTTC
PF10_0346; 1578 4.40e-04 TAGTGCACAT TTTACCTT TATACAACTT
MEME, mero_uig8, anr2, TTTCCC, w=6,s=39,llr=389,E=4.2e-009
anr2
PF13_0198; 1025 3.75e-06 TTTTCAATTT TTCCCC TTTTTTCTAT*
PF11_0381; 903 3.75e-06 ATTATTTTTT TTCCCC ATAAATATTT*
PF11_0395; 1820 3.75e-06 TTTGTTAATA TTCCCC TTGTAATAGT*
PFL1110C; 1826 3.75e-06 CTTTTTTGTT TTCCCC ACATAACTAA*
PF10_0268; 430 3.75e-06 ATATGTACGC TTCCCC CCTCCCGATA*
PFF0520w; 82 3.75e-06 TTTTTTTTTC TTCCCC TTTTCACAAT
PFB0310C; 985 3.75e-06 TTTAGATATT TTCCCC ATTTGGGAAT
MAL7P1.73; 81 4.37e-06 TTTATTTTTT GTCCCC CTTAAATAAA*
PFI0265C; 1333 4.98e-06 TATAATTAAT TGCCCC ATTTTTTTTC*
PF14_0281; 234 3.04e-05 AACAACATTC TTTCCC TTTATTGATT*
PF13_0197; 30 3.04e-05 ATGTTCATAT TTTCCC ATTCTTATAA*
PF11_0298; 848 3.04e-05 TTTTTAATTA TTTCCC TAAAGAATAC
PF11_0377; 784 3.04e-05 TTTGAATTAT TTTCCC ATATTAAAAG*
PF11_0395; 1240 3.04e-05 TTTTTTAAAT TTTCCC AAGGTAAAAA
PFL1110C; 1545 3.04e-05 TTTTTATAAT TTTCCC ATTTTCATTG
PFI0265C; 43 3.04e-05 CTATATCATA TTTCCC TGACGTTTTT
PFL2520w; 236 3.04e-05 ATTTTGTTAA TTTCCC AAAATATTAT
PFI1475w; 565 3.04e-05 CATTACATTA TTTCCC TTTTATTAAA
PFI1475w; 117 3.04e-05 ATTATCATAA TTTCCC TGGTTTTGAC
PF07_0072; 834 3.04e-05 AAAAAAAAGG TTTCCC TATCTTTTCA
PF08_0108; 1304 3.04e-05 AATATTGAAA TTTCCC ATGCCTTTTA
PF08_0108; 333 3.04e-05 TTTTCACTGT TTTCCC TTAAAATTTC*
PFE0420C; 10 3.04e-05 CTTTTTATA TTTCCC ATTTATTAAA
PFE0370C; 1143 3.04e-05 TCTTTTTTAA TTTCCC TAATTTACAT
PFE0370C; 939 3.04e-05 TTTTTTTTTT TTTCCC AACTCCAAAA
PFC0945w; 1271 3.04e-05 TATTAGATTT TTTCCC TTTTTAAATA*
PFB0665w; 1098 3.04e-05 TTATAATTTA TTTCCC ATTTAAATAA
PFB0665w; 54 3.04e-05 TTTTCCATTT TTTCCC ATTTCACACT
PFB0315w; 1510 3.04e-05 TTTTTTTTTT TTTCCC TAATCTATAT
PFB0315w; 1284 3.04e-05 TTATTTTAAT TTTCCC ACACAACATA
MAL13P1.176; 1287 3.80e-05 TAAAATAATT GTTCCC GTTTTTTTCT
PFB0150C; 83 3.80e-05 TATATCTTTT TACCCC TAGAAAGAAT
PF13_0198; 1726 3.80e-05 TAAAATAATT GTTCCC GTTTTTTTCT
MAL13P1.278; 282 3.80e-05 ATATATACAA TACCCC CTTTGTCATA
MAL13P1.118; 671 3.80e-05 ACGAGTATTA TACCCC ATAATTATAA
PFF0615C; 873 3.80e-05 TTTATATATA TACCCC TTTTTATATA
PFB0315w; 121 3.80e-05 TATAAGGCAT TACCCC ATTTATATAT
PF07_0072; 387 4.21e-05 ACATTTAAGA TGTCCC ATATTGTCTA
PFC0945w; 54 4.34e-05 TTATATAAGG GGTCCC TTTATTAGAG
7

## Slide 8
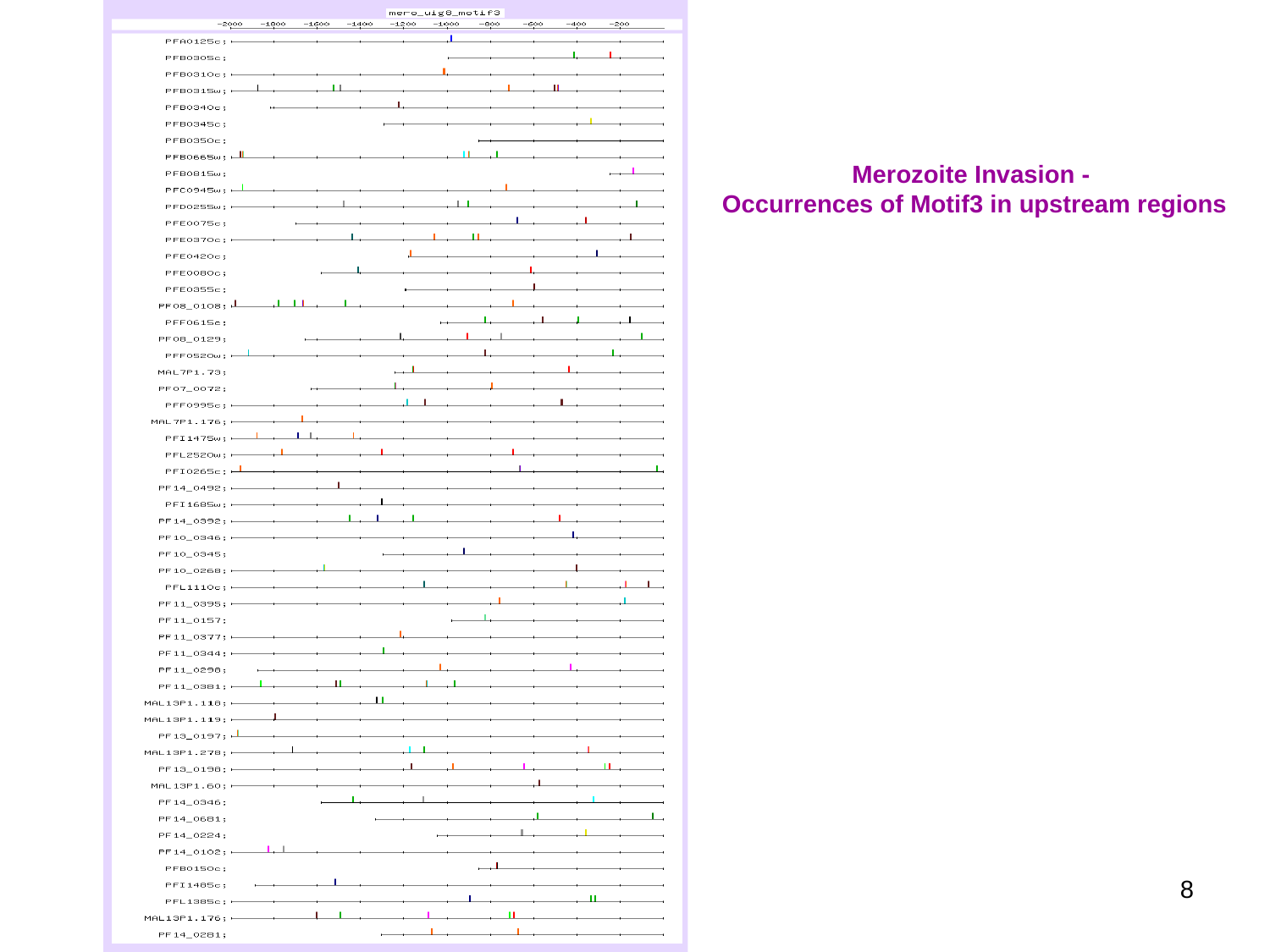

Merozoite Invasion -
Occurrences of Motif3 in upstream regions
8

## Slide 9
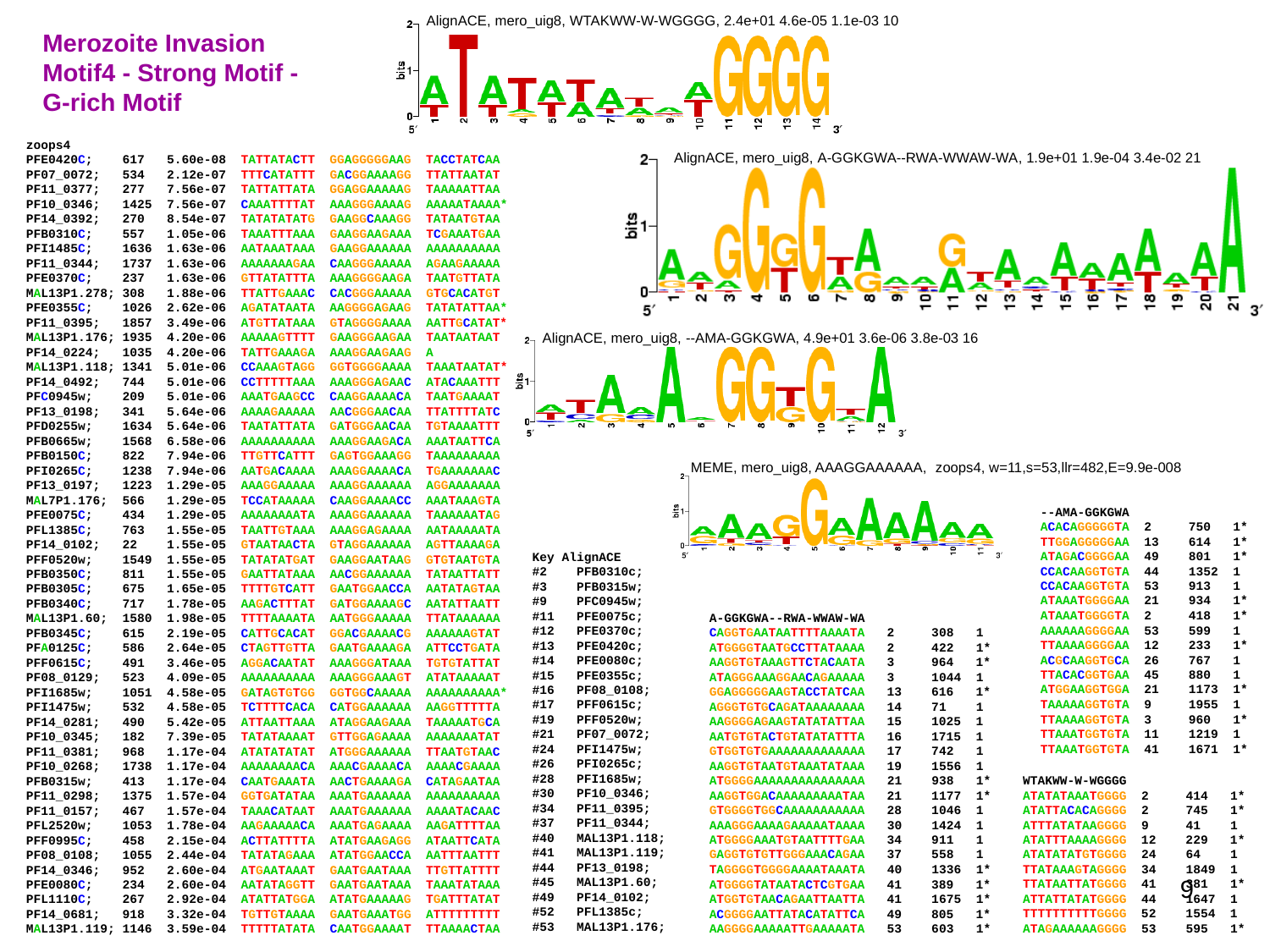

AlignACE, mero_uig8, WTAKWW-W-WGGGG, 2.4e+01 4.6e-05 1.1e-03 10
AlignACE, mero_uig8, A-GGKGWA--RWA-WWAW-WA, 1.9e+01 1.9e-04 3.4e-02 21
AlignACE, mero_uig8, --AMA-GGKGWA, 4.9e+01 3.6e-06 3.8e-03 16
MEME, mero_uig8, AAAGGAAAAAA, zoops4, w=11,s=53,llr=482,E=9.9e-008
Merozoite Invasion
Motif4 - Strong Motif -
G-rich Motif
zoops4
PFE0420C; 617 5.60e-08 TATTATACTT GGAGGGGGAAG TACCTATCAA
PF07_0072; 534 2.12e-07 TTTCATATTT GACGGAAAAGG TTATTAATAT
PF11_0377; 277 7.56e-07 TATTATTATA GGAGGAAAAAG TAAAAATTAA
PF10_0346; 1425 7.56e-07 CAAATTTTAT AAAGGGAAAAG AAAAATAAAA*
PF14_0392; 270 8.54e-07 TATATATATG GAAGGCAAAGG TATAATGTAA
PFB0310C; 557 1.05e-06 TAAATTTAAA GAAGGAAGAAA TCGAAATGAA
PFI1485C; 1636 1.63e-06 AATAAATAAA GAAGGAAAAAA AAAAAAAAAA
PF11_0344; 1737 1.63e-06 AAAAAAAGAA CAAGGGAAAAA AGAAGAAAAA
PFE0370C; 237 1.63e-06 GTTATATTTA AAAGGGGAAGA TAATGTTATA
MAL13P1.278; 308 1.88e-06 TTATTGAAAC CACGGGAAAAA GTGCACATGT
PFE0355C; 1026 2.62e-06 AGATATAATA AAGGGGAGAAG TATATATTAA*
PF11_0395; 1857 3.49e-06 ATGTTATAAA GTAGGGGAAAA AATTGCATAT*
MAL13P1.176; 1935 4.20e-06 AAAAAGTTTT GAAGGGAAGAA TAATAATAAT
PF14_0224; 1035 4.20e-06 TATTGAAAGA AAAGGAAGAAG A
MAL13P1.118; 1341 5.01e-06 CCAAAGTAGG GGTGGGGAAAA TAAATAATAT*
PF14_0492; 744 5.01e-06 CCTTTTTAAA AAAGGGAGAAC ATACAAATTT
PFC0945w; 209 5.01e-06 AAATGAAGCC CAAGGAAAACA TAATGAAAAT
PF13_0198; 341 5.64e-06 AAAAGAAAAA AACGGGAACAA TTATTTTATC
PFD0255w; 1634 5.64e-06 TAATATTATA GATGGGAACAA TGTAAAATTT
PFB0665w; 1568 6.58e-06 AAAAAAAAAA AAAGGAAGACA AAATAATTCA
PFB0150C; 822 7.94e-06 TTGTTCATTT GAGTGGAAAGG TAAAAAAAAA
PFI0265C; 1238 7.94e-06 AATGACAAAA AAAGGAAAACA TGAAAAAAAC
PF13_0197; 1223 1.29e-05 AAAGGAAAAA AAAGGAAAAAA AGGAAAAAAA
MAL7P1.176; 566 1.29e-05 TCCATAAAAA CAAGGAAAACC AAATAAAGTA
PFE0075C; 434 1.29e-05 AAAAAAAATA AAAGGAAAAAA TAAAAAATAG
PFL1385C; 763 1.55e-05 TAATTGTAAA AAAGGAGAAAA AATAAAAATA
PF14_0102; 22 1.55e-05 GTAATAACTA GTAGGAAAAAA AGTTAAAAGA
PFF0520w; 1549 1.55e-05 TATATATGAT GAAGGAATAAG GTGTAATGTA
PFB0350C; 811 1.55e-05 GAATTATAAA AACGGAAAAAA TATAATTATT
PFB0305C; 675 1.65e-05 TTTTGTCATT GAATGGAACCA AATATAGTAA
PFB0340C; 717 1.78e-05 AAGACTTTAT GATGGAAAAGC AATATTAATT
MAL13P1.60; 1580 1.98e-05 TTTTAAAATA AATGGGAAAAA TTATAAAAAA
PFB0345C; 615 2.19e-05 CATTGCACAT GGACGAAAACG AAAAAAGTAT
PFA0125C; 586 2.64e-05 CTAGTTGTTA GAATGAAAAGA ATTCCTGATA
PFF0615C; 491 3.46e-05 AGGACAATAT AAAGGGATAAA TGTGTATTAT
PF08_0129; 523 4.09e-05 AAAAAAAAAA AAAGGGAAAGT ATATAAAAAT
PFI1685w; 1051 4.58e-05 GATAGTGTGG GGTGGCAAAAA AAAAAAAAAA*
PFI1475w; 532 4.58e-05 TCTTTTCACA CATGGAAAAAA AAGGTTTTTA
PF14_0281; 490 5.42e-05 ATTAATTAAA ATAGGAAGAAA TAAAAATGCA
PF10_0345; 182 7.39e-05 TATATAAAAT GTTGGAGAAAA AAAAAAATAT
PF11_0381; 968 1.17e-04 ATATATATAT ATGGGAAAAAA TTAATGTAAC
PF10_0268; 1738 1.17e-04 AAAAAAAACA AAACGAAAACA AAAACGAAAA
PFB0315w; 413 1.17e-04 CAATGAAATA AACTGAAAAGA CATAGAATAA
PF11_0298; 1375 1.57e-04 GGTGATATAA AAATGAAAAAA AAAAAAAAAA
PF11_0157; 467 1.57e-04 TAAACATAAT AAATGAAAAAA AAAATACAAC
PFL2520w; 1053 1.78e-04 AAGAAAAACA AAATGAGAAAA AAGATTTTAA
PFF0995C; 458 2.15e-04 ACTTATTTTA ATATGAAGAGG ATAATTCATA
PF08_0108; 1055 2.44e-04 TATATAGAAA ATATGGAACCA AATTTAATTT
PF14_0346; 952 2.60e-04 ATGAATAAAT GAATGAATAAA TTGTTATTTT
PFE0080C; 234 2.60e-04 AATATAGGTT GAATGAATAAA TAAATATAAA
PFL1110C; 267 2.92e-04 ATATTATGGA ATATGAAAAAG TGATTTATAT
PF14_0681; 918 3.32e-04 TGTTGTAAAA GAATGAAATGG ATTTTTTTTT
MAL13P1.119; 1146 3.59e-04 TTTTTATATA CAATGGAAAAT TTAAAACTAA
--AMA-GGKGWA
ACACAGGGGGTA 2 750 1*
TTGGAGGGGGAA 13 614 1*
ATAGACGGGGAA 49 801 1*
CCACAAGGTGTA 44 1352 1
CCACAAGGTGTA 53 913 1
ATAAATGGGGAA 21 934 1*
ATAAATGGGGTA 2 418 1*
AAAAAAGGGGAA 53 599 1
TTAAAAGGGGAA 12 233 1*
ACGCAAGGTGCA 26 767 1
TTACACGGTGAA 45 880 1
ATGGAAGGTGGA 21 1173 1*
TAAAAAGGTGTA 9 1955 1
TTAAAAGGTGTA 3 960 1*
TTAAATGGTGTA 11 1219 1
TTAAATGGTGTA 41 1671 1*
Key AlignACE
#2 PFB0310c;
#3 PFB0315w;
#9 PFC0945w;
#11 PFE0075c;
#12 PFE0370c;
#13 PFE0420c;
#14 PFE0080c;
#15 PFE0355c;
#16 PF08_0108;
#17 PFF0615c;
#19 PFF0520w;
#21 PF07_0072;
#24 PFI1475w;
#26 PFI0265c;
#28 PFI1685w;
#30 PF10_0346;
#34 PF11_0395;
#37 PF11_0344;
#40 MAL13P1.118;
#41 MAL13P1.119;
#44 PF13_0198;
#45 MAL13P1.60;
#49 PF14_0102;
#52 PFL1385c;
#53 MAL13P1.176;
A-GGKGWA--RWA-WWAW-WA
CAGGTGAATAATTTTAAAATA 2 308 1
ATGGGGTAATGCCTTATAAAA 2 422 1*
AAGGTGTAAAGTTCTACAATA 3 964 1*
ATAGGGAAAGGAACAGAAAAA 3 1044 1
GGAGGGGGAAGTACCTATCAA 13 616 1*
AGGGTGTGCAGATAAAAAAAA 14 71 1
AAGGGGAGAAGTATATATTAA 15 1025 1
AATGTGTACTGTATATATTTA 16 1715 1
GTGGTGTGAAAAAAAAAAAAA 17 742 1
AAGGTGTAATGTAAATATAAA 19 1556 1
ATGGGGAAAAAAAAAAAAAAA 21 938 1*
AAGGTGGACAAAAAAAAATAA 21 1177 1*
GTGGGGTGGCAAAAAAAAAAA 28 1046 1
AAAGGGAAAAGAAAAATAAAA 30 1424 1
ATGGGGAAATGTAATTTTGAA 34 911 1
GAGGTGTGTTGGGAAACAGAA 37 558 1
TAGGGGTGGGGAAAATAAATA 40 1336 1*
ATGGGGTATAATACTCGTGAA 41 389 1*
ATGGTGTAACAGAATTAATTA 41 1675 1*
ACGGGGAATTATACATATTCA 49 805 1*
AAGGGGAAAAATTGAAAAATA 53 603 1*
WTAKWW-W-WGGGG
ATATATAAATGGGG 2 414 1*
ATATTACACAGGGG 2 745 1*
ATTTATATAAGGGG 9 41 1
ATATTTAAAAGGGG 12 229 1*
ATATATATGTGGGG 24 64 1
TTATAAAGTAGGGG 34 1849 1
TTATAATTATGGGG 41 381 1*
ATTATTATATGGGG 44 1647 1
TTTTTTTTTTGGGG 52 1554 1
ATAGAAAAAAGGGG 53 595 1*
9

## Slide 10
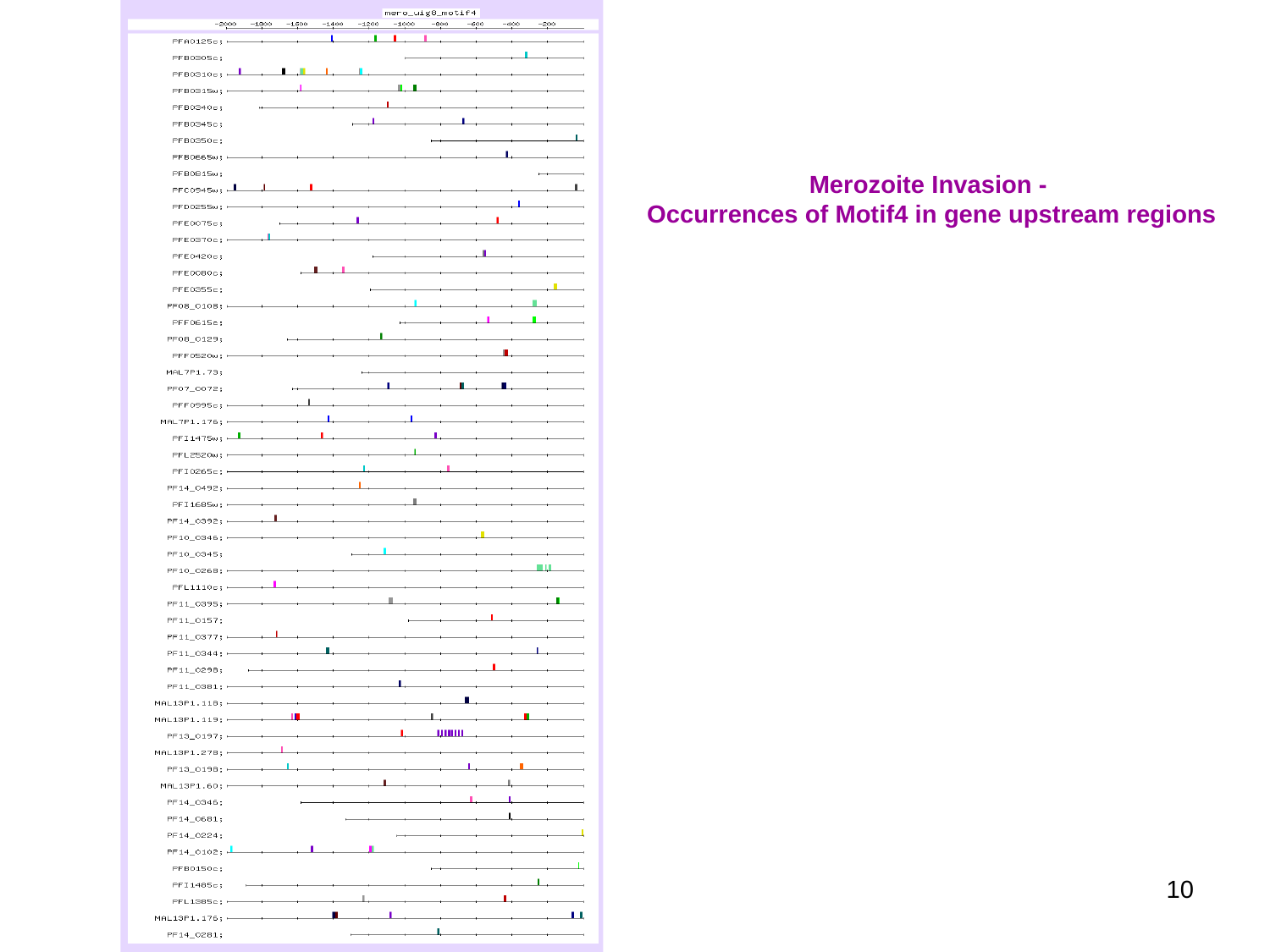

Merozoite Invasion -
Occurrences of Motif4 in gene upstream regions
10

## Slide 11
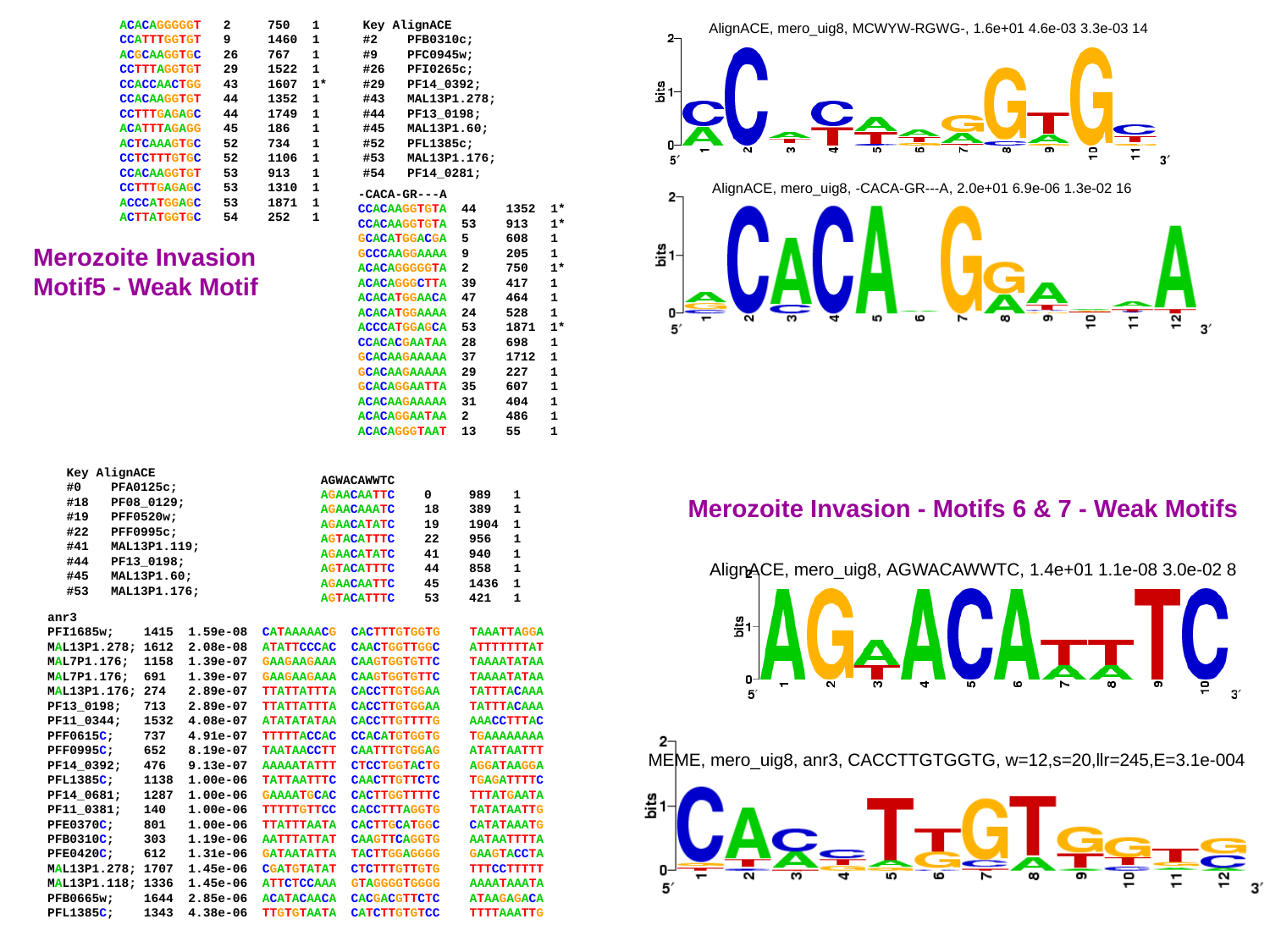

ACACAGGGGGT 2 750 1
CCATTTGGTGT 9 1460 1
ACGCAAGGTGC 26 767 1
CCTTTAGGTGT 29 1522 1
CCACCAACTGG 43 1607 1*
CCACAAGGTGT 44 1352 1
CCTTTGAGAGC 44 1749 1
ACATTTAGAGG 45 186 1
ACTCAAAGTGC 52 734 1
CCTCTTTGTGC 52 1106 1
CCACAAGGTGT 53 913 1
CCTTTGAGAGC 53 1310 1
ACCCATGGAGC 53 1871 1
ACTTATGGTGC 54 252 1
Key AlignACE
#2 PFB0310c;
#9 PFC0945w;
#26 PFI0265c;
#29 PF14_0392;
#43 MAL13P1.278;
#44 PF13_0198;
#45 MAL13P1.60;
#52 PFL1385c;
#53 MAL13P1.176;
#54 PF14_0281;
AlignACE, mero_uig8, MCWYW-RGWG-, 1.6e+01 4.6e-03 3.3e-03 14
AlignACE, mero_uig8, -CACA-GR---A, 2.0e+01 6.9e-06 1.3e-02 16
-CACA-GR---A
CCACAAGGTGTA 44 1352 1*
CCACAAGGTGTA 53 913 1*
GCACATGGACGA 5 608 1
GCCCAAGGAAAA 9 205 1
ACACAGGGGGTA 2 750 1*
ACACAGGGCTTA 39 417 1
ACACATGGAACA 47 464 1
ACACATGGAAAA 24 528 1
ACCCATGGAGCA 53 1871 1*
CCACACGAATAA 28 698 1
GCACAAGAAAAA 37 1712 1
GCACAAGAAAAA 29 227 1
GCACAGGAATTA 35 607 1
ACACAAGAAAAA 31 404 1
ACACAGGAATAA 2 486 1
ACACAGGGTAAT 13 55 1
Merozoite Invasion
Motif5 - Weak Motif
Key AlignACE
#0 PFA0125c;
#18 PF08_0129;
#19 PFF0520w;
#22 PFF0995c;
#41 MAL13P1.119;
#44 PF13_0198;
#45 MAL13P1.60;
#53 MAL13P1.176;
AGWACAWWTC
AGAACAATTC 0 989 1
AGAACAAATC 18 389 1
AGAACATATC 19 1904 1
AGTACATTTC 22 956 1
AGAACATATC 41 940 1
AGTACATTTC 44 858 1
AGAACAATTC 45 1436 1
AGTACATTTC 53 421 1
Merozoite Invasion - Motifs 6 & 7 - Weak Motifs
AlignACE, mero_uig8, AGWACAWWTC, 1.4e+01 1.1e-08 3.0e-02 8
anr3
PFI1685w; 1415 1.59e-08 CATAAAAACG CACTTTGTGGTG TAAATTAGGA
MAL13P1.278; 1612 2.08e-08 ATATTCCCAC CAACTGGTTGGC ATTTTTTTAT
MAL7P1.176; 1158 1.39e-07 GAAGAAGAAA CAAGTGGTGTTC TAAAATATAA
MAL7P1.176; 691 1.39e-07 GAAGAAGAAA CAAGTGGTGTTC TAAAATATAA
MAL13P1.176; 274 2.89e-07 TTATTATTTA CACCTTGTGGAA TATTTACAAA
PF13_0198; 713 2.89e-07 TTATTATTTA CACCTTGTGGAA TATTTACAAA
PF11_0344; 1532 4.08e-07 ATATATATAA CACCTTGTTTTG AAACCTTTAC
PFF0615C; 737 4.91e-07 TTTTTACCAC CCACATGTGGTG TGAAAAAAAA
PFF0995C; 652 8.19e-07 TAATAACCTT CAATTTGTGGAG ATATTAATTT
PF14_0392; 476 9.13e-07 AAAAATATTT CTCCTGGTACTG AGGATAAGGA
PFL1385C; 1138 1.00e-06 TATTAATTTC CAACTTGTTCTC TGAGATTTTC
PF14_0681; 1287 1.00e-06 GAAAATGCAC CACTTGGTTTTC TTTATGAATA
PF11_0381; 140 1.00e-06 TTTTTGTTCC CACCTTTAGGTG TATATAATTG
PFE0370C; 801 1.00e-06 TTATTTAATA CACTTGCATGGC CATATAAATG
PFB0310C; 303 1.19e-06 AATTTATTAT CAAGTTCAGGTG AATAATTTTA
PFE0420C; 612 1.31e-06 GATAATATTA TACTTGGAGGGG GAAGTACCTA
MAL13P1.278; 1707 1.45e-06 CGATGTATAT CTCTTTGTTGTG TTTCCTTTTT
MAL13P1.118; 1336 1.45e-06 ATTCTCCAAA GTAGGGGTGGGG AAAATAAATA
PFB0665w; 1644 2.85e-06 ACATACAACA CACGACGTTCTC ATAAGAGACA
PFL1385C; 1343 4.38e-06 TTGTGTAATA CATCTTGTGTCC TTTTAAATTG
MEME, mero_uig8, anr3, CACCTTGTGGTG, w=12,s=20,llr=245,E=3.1e-004
11

## Slide 12
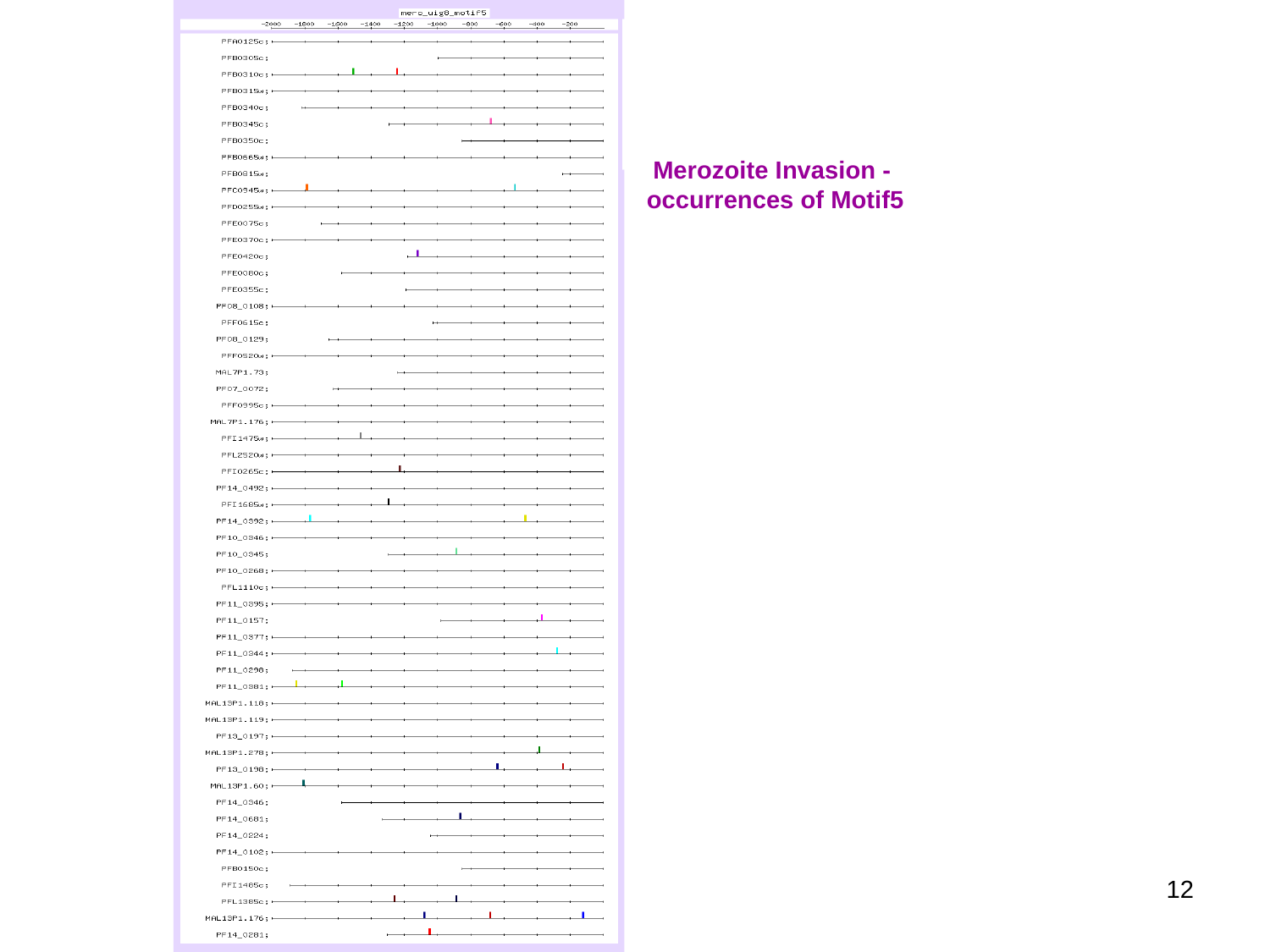

Merozoite Invasion -
occurrences of Motif5
12

## Slide 13
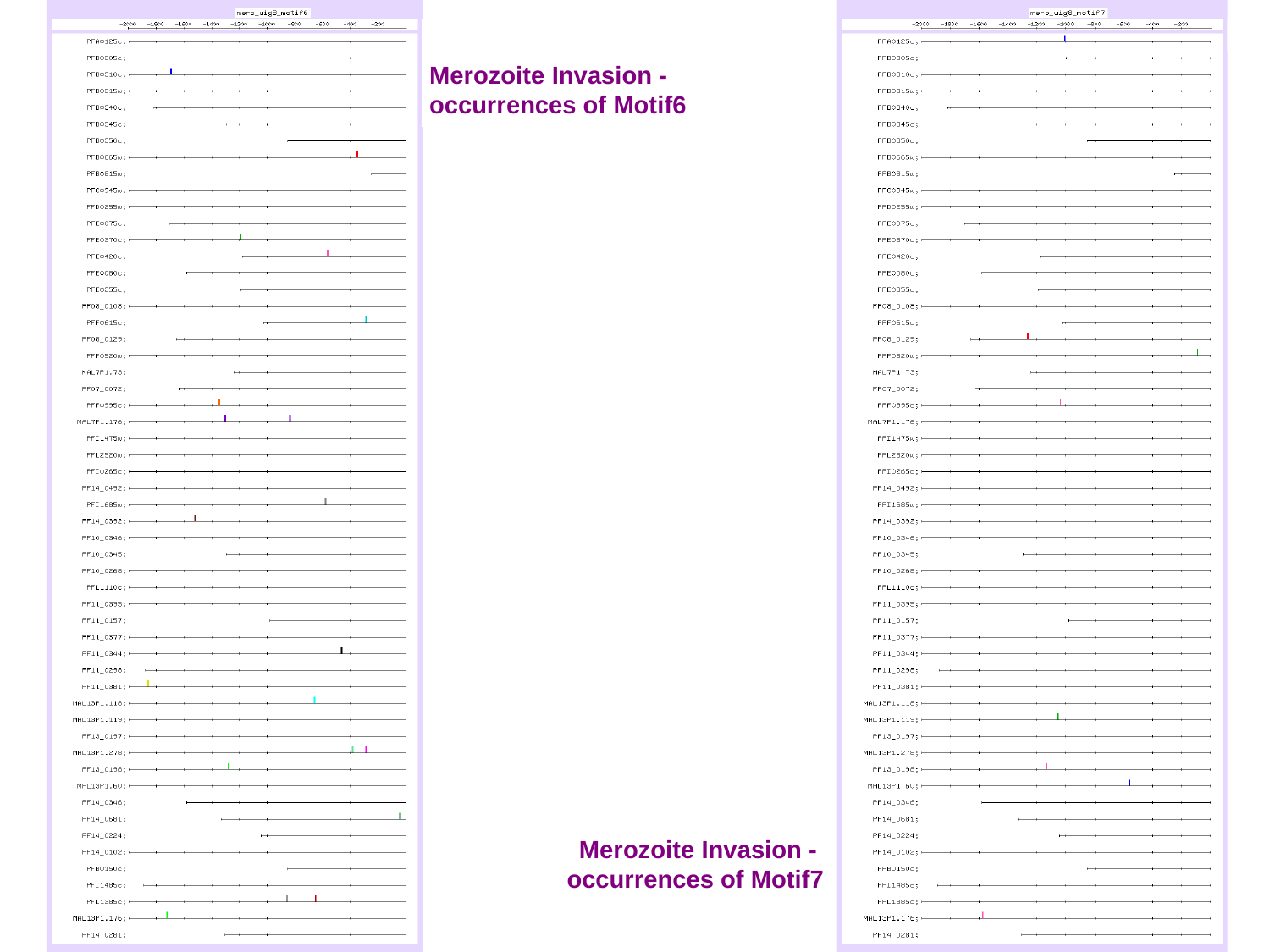

Merozoite Invasion -
occurrences of Motif6
Merozoite Invasion -
occurrences of Motif7
13

## Slide 14
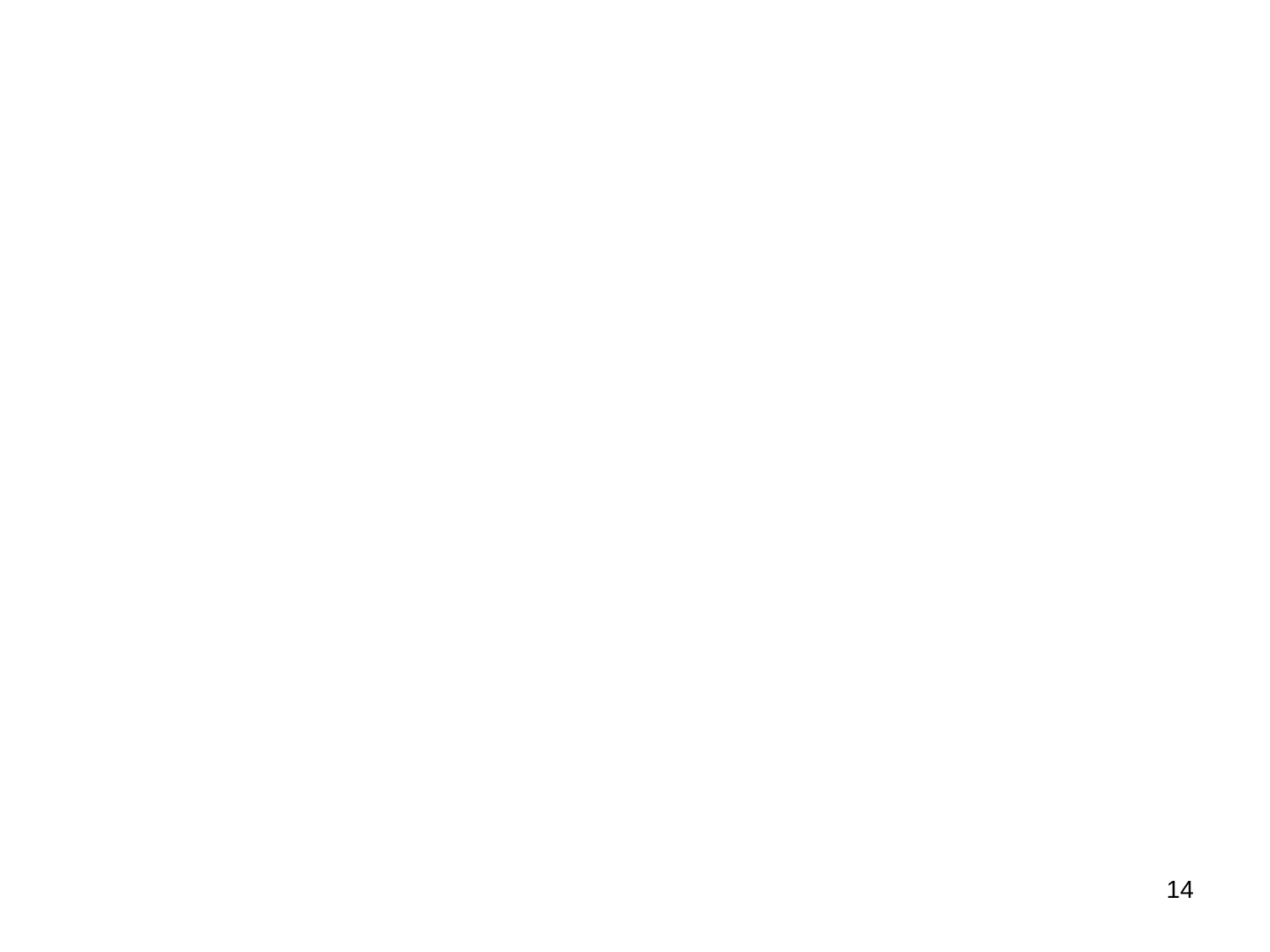

14

## Slide 15
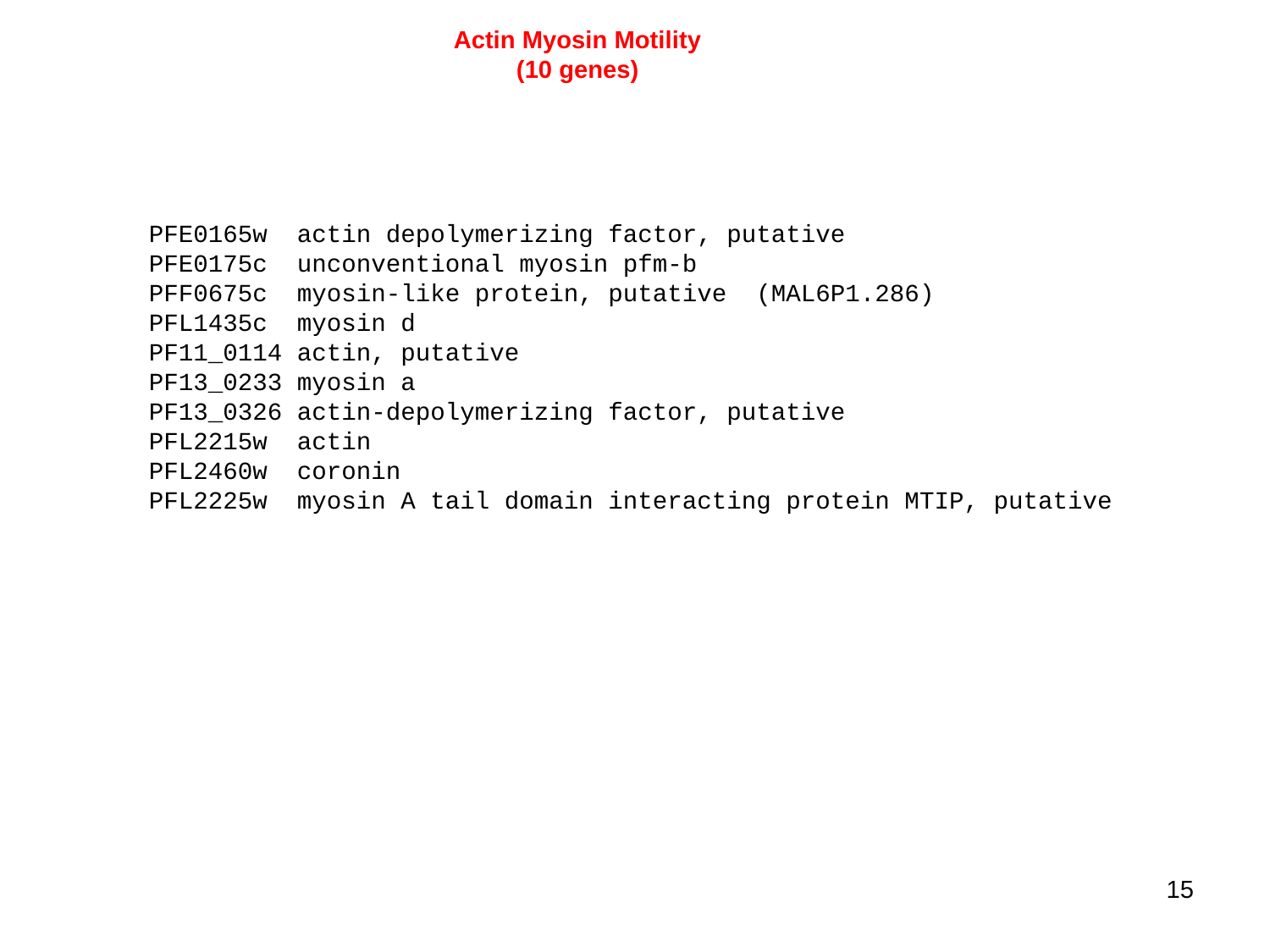

Actin Myosin Motility
(10 genes)
PFE0165w actin depolymerizing factor, putative
PFE0175c unconventional myosin pfm-b
PFF0675c myosin-like protein, putative (MAL6P1.286)
PFL1435c myosin d
PF11_0114 actin, putative
PF13_0233 myosin a
PF13_0326 actin-depolymerizing factor, putative
PFL2215w actin
PFL2460w coronin
PFL2225w myosin A tail domain interacting protein MTIP, putative
15

## Slide 16
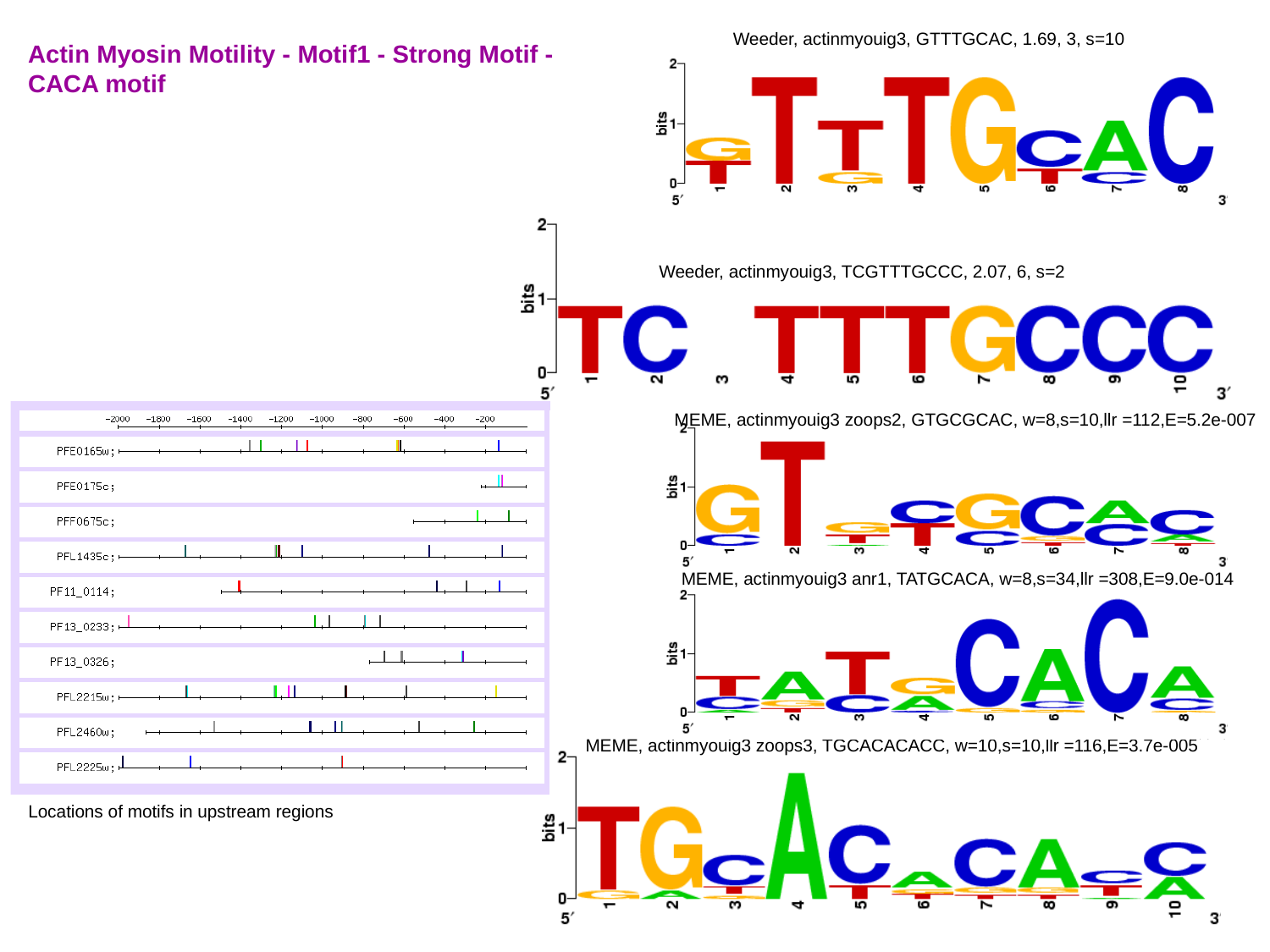

Weeder, actinmyouig3, GTTTGCAC, 1.69, 3, s=10
Actin Myosin Motility - Motif1 - Strong Motif -
CACA motif
Weeder, actinmyouig3, TCGTTTGCCC, 2.07, 6, s=2
MEME, actinmyouig3 zoops2, GTGCGCAC, w=8,s=10,llr =112,E=5.2e-007
MEME, actinmyouig3 anr1, TATGCACA, w=8,s=34,llr =308,E=9.0e-014
MEME, actinmyouig3 zoops3, TGCACACACC, w=10,s=10,llr =116,E=3.7e-005
Locations of motifs in upstream regions
16

## Slide 17
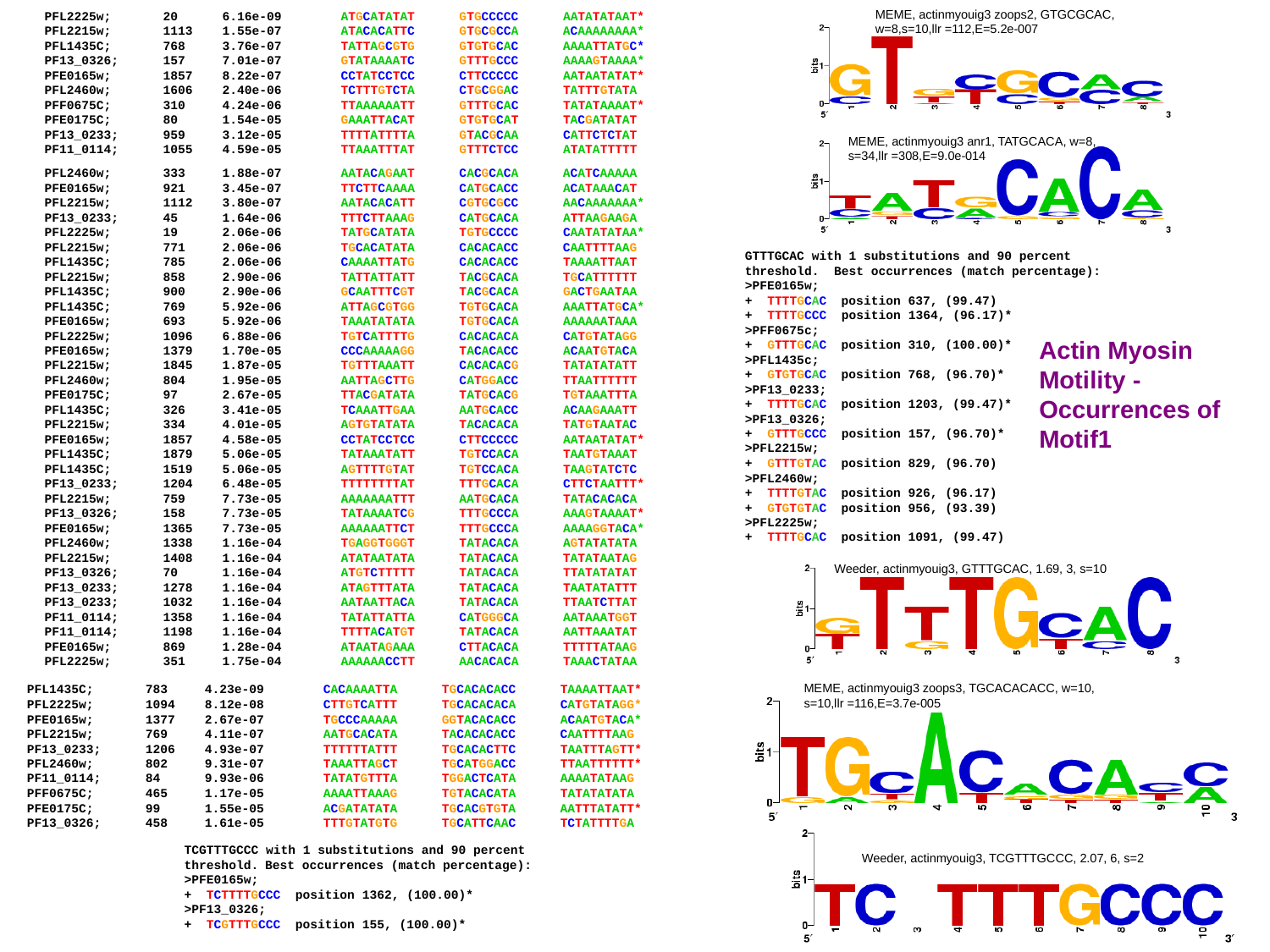

MEME, actinmyouig3 zoops2, GTGCGCAC,
w=8,s=10,llr =112,E=5.2e-007
PFL2225w; 20 6.16e-09 ATGCATATAT GTGCCCCC AATATATAAT*
PFL2215w; 1113 1.55e-07 ATACACATTC GTGCGCCA ACAAAAAAAA*
PFL1435C; 768 3.76e-07 TATTAGCGTG GTGTGCAC AAAATTATGC*
PF13_0326; 157 7.01e-07 GTATAAAATC GTTTGCCC AAAAGTAAAA*
PFE0165w; 1857 8.22e-07 CCTATCCTCC CTTCCCCC AATAATATAT*
PFL2460w; 1606 2.40e-06 TCTTTGTCTA CTGCGGAC TATTTGTATA
PFF0675C; 310 4.24e-06 TTAAAAAATT GTTTGCAC TATATAAAAT*
PFE0175C; 80 1.54e-05 GAAATTACAT GTGTGCAT TACGATATAT
PF13_0233; 959 3.12e-05 TTTTATTTTA GTACGCAA CATTCTCTAT
PF11_0114; 1055 4.59e-05 TTAAATTTAT GTTTCTCC ATATATTTTT
MEME, actinmyouig3 anr1, TATGCACA, w=8,
s=34,llr =308,E=9.0e-014
PFL2460w; 333 1.88e-07 AATACAGAAT CACGCACA ACATCAAAAA
PFE0165w; 921 3.45e-07 TTCTTCAAAA CATGCACC ACATAAACAT
PFL2215w; 1112 3.80e-07 AATACACATT CGTGCGCC AACAAAAAAA*
PF13_0233; 45 1.64e-06 TTTCTTAAAG CATGCACA ATTAAGAAGA
PFL2225w; 19 2.06e-06 TATGCATATA TGTGCCCC CAATATATAA*
PFL2215w; 771 2.06e-06 TGCACATATA CACACACC CAATTTTAAG
PFL1435C; 785 2.06e-06 CAAAATTATG CACACACC TAAAATTAAT
PFL2215w; 858 2.90e-06 TATTATTATT TACGCACA TGCATTTTTT
PFL1435C; 900 2.90e-06 GCAATTTCGT TACGCACA GACTGAATAA
PFL1435C; 769 5.92e-06 ATTAGCGTGG TGTGCACA AAATTATGCA*
PFE0165w; 693 5.92e-06 TAAATATATA TGTGCACA AAAAAATAAA
PFL2225w; 1096 6.88e-06 TGTCATTTTG CACACACA CATGTATAGG
PFE0165w; 1379 1.70e-05 CCCAAAAAGG TACACACC ACAATGTACA
PFL2215w; 1845 1.87e-05 TGTTTAAATT CACACACG TATATATATT
PFL2460w; 804 1.95e-05 AATTAGCTTG CATGGACC TTAATTTTTT
PFE0175C; 97 2.67e-05 TTACGATATA TATGCACG TGTAAATTTA
PFL1435C; 326 3.41e-05 TCAAATTGAA AATGCACC ACAAGAAATT
PFL2215w; 334 4.01e-05 AGTGTATATA TACACACA TATGTAATAC
PFE0165w; 1857 4.58e-05 CCTATCCTCC CTTCCCCC AATAATATAT*
PFL1435C; 1879 5.06e-05 TATAAATATT TGTCCACA TAATGTAAAT
PFL1435C; 1519 5.06e-05 AGTTTTGTAT TGTCCACA TAAGTATCTC
PF13_0233; 1204 6.48e-05 TTTTTTTTAT TTTGCACA CTTCTAATTT*
PFL2215w; 759 7.73e-05 AAAAAAATTT AATGCACA TATACACACA
PF13_0326; 158 7.73e-05 TATAAAATCG TTTGCCCA AAAGTAAAAT*
PFE0165w; 1365 7.73e-05 AAAAAATTCT TTTGCCCA AAAAGGTACA*
PFL2460w; 1338 1.16e-04 TGAGGTGGGT TATACACA AGTATATATA
PFL2215w; 1408 1.16e-04 ATATAATATA TATACACA TATATAATAG
PF13_0326; 70 1.16e-04 ATGTCTTTTT TATACACA TTATATATAT
PF13_0233; 1278 1.16e-04 ATAGTTTATA TATACACA TAATATATTT
PF13_0233; 1032 1.16e-04 AATAATTACA TATACACA TTAATCTTAT
PF11_0114; 1358 1.16e-04 TATATTATTA CATGGGCA AATAAATGGT
PF11_0114; 1198 1.16e-04 TTTTACATGT TATACACA AATTAAATAT
PFE0165w; 869 1.28e-04 ATAATAGAAA CTTACACA TTTTTATAAG
PFL2225w; 351 1.75e-04 AAAAAACCTT AACACACA TAAACTATAA
GTTTGCAC with 1 substitutions and 90 percent
threshold. Best occurrences (match percentage):
>PFE0165w;
+ TTTTGCAC position 637, (99.47)
+ TTTTGCCC position 1364, (96.17)*
>PFF0675c;
+ GTTTGCAC position 310, (100.00)*
>PFL1435c;
+ GTGTGCAC position 768, (96.70)*
>PF13_0233;
+ TTTTGCAC position 1203, (99.47)*
>PF13_0326;
+ GTTTGCCC position 157, (96.70)*
>PFL2215w;
+ GTTTGTAC position 829, (96.70)
>PFL2460w;
+ TTTTGTAC position 926, (96.17)
+ GTGTGTAC position 956, (93.39)
>PFL2225w;
+ TTTTGCAC position 1091, (99.47)
Actin Myosin Motility -
Occurrences of
Motif1
Weeder, actinmyouig3, GTTTGCAC, 1.69, 3, s=10
MEME, actinmyouig3 zoops3, TGCACACACC, w=10,
s=10,llr =116,E=3.7e-005
PFL1435C; 783 4.23e-09 CACAAAATTA TGCACACACC TAAAATTAAT*
PFL2225w; 1094 8.12e-08 CTTGTCATTT TGCACACACA CATGTATAGG*
PFE0165w; 1377 2.67e-07 TGCCCAAAAA GGTACACACC ACAATGTACA*
PFL2215w; 769 4.11e-07 AATGCACATA TACACACACC CAATTTTAAG
PF13_0233; 1206 4.93e-07 TTTTTTATTT TGCACACTTC TAATTTAGTT*
PFL2460w; 802 9.31e-07 TAAATTAGCT TGCATGGACC TTAATTTTTT*
PF11_0114; 84 9.93e-06 TATATGTTTA TGGACTCATA AAAATATAAG
PFF0675C; 465 1.17e-05 AAAATTAAAG TGTACACATA TATATATATA
PFE0175C; 99 1.55e-05 ACGATATATA TGCACGTGTA AATTTATATT*
PF13_0326; 458 1.61e-05 TTTGTATGTG TGCATTCAAC TCTATTTTGA
TCGTTTGCCC with 1 substitutions and 90 percent
threshold. Best occurrences (match percentage):
>PFE0165w;
+ TCTTTTGCCC position 1362, (100.00)*
>PF13_0326;
+ TCGTTTGCCC position 155, (100.00)*
Weeder, actinmyouig3, TCGTTTGCCC, 2.07, 6, s=2
17

## Slide 18
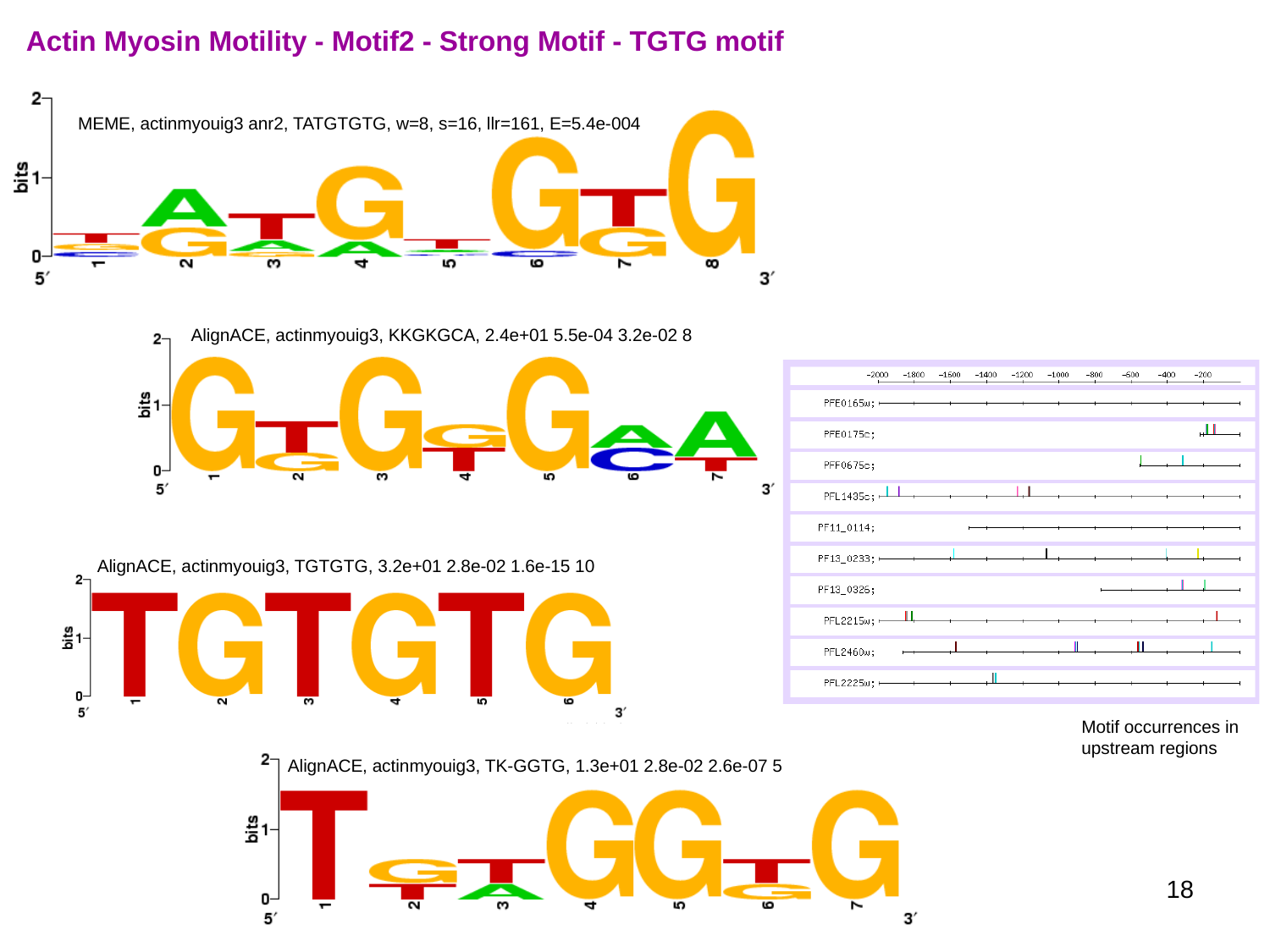

Actin Myosin Motility - Motif2 - Strong Motif - TGTG motif
MEME, actinmyouig3 anr2, TATGTGTG, w=8, s=16, llr=161, E=5.4e-004
AlignACE, actinmyouig3, KKGKGCA, 2.4e+01 5.5e-04 3.2e-02 8
AlignACE, actinmyouig3, TGTGTG, 3.2e+01 2.8e-02 1.6e-15 10
Motif occurrences in
upstream regions
AlignACE, actinmyouig3, TK-GGTG, 1.3e+01 2.8e-02 2.6e-07 5
18

## Slide 19
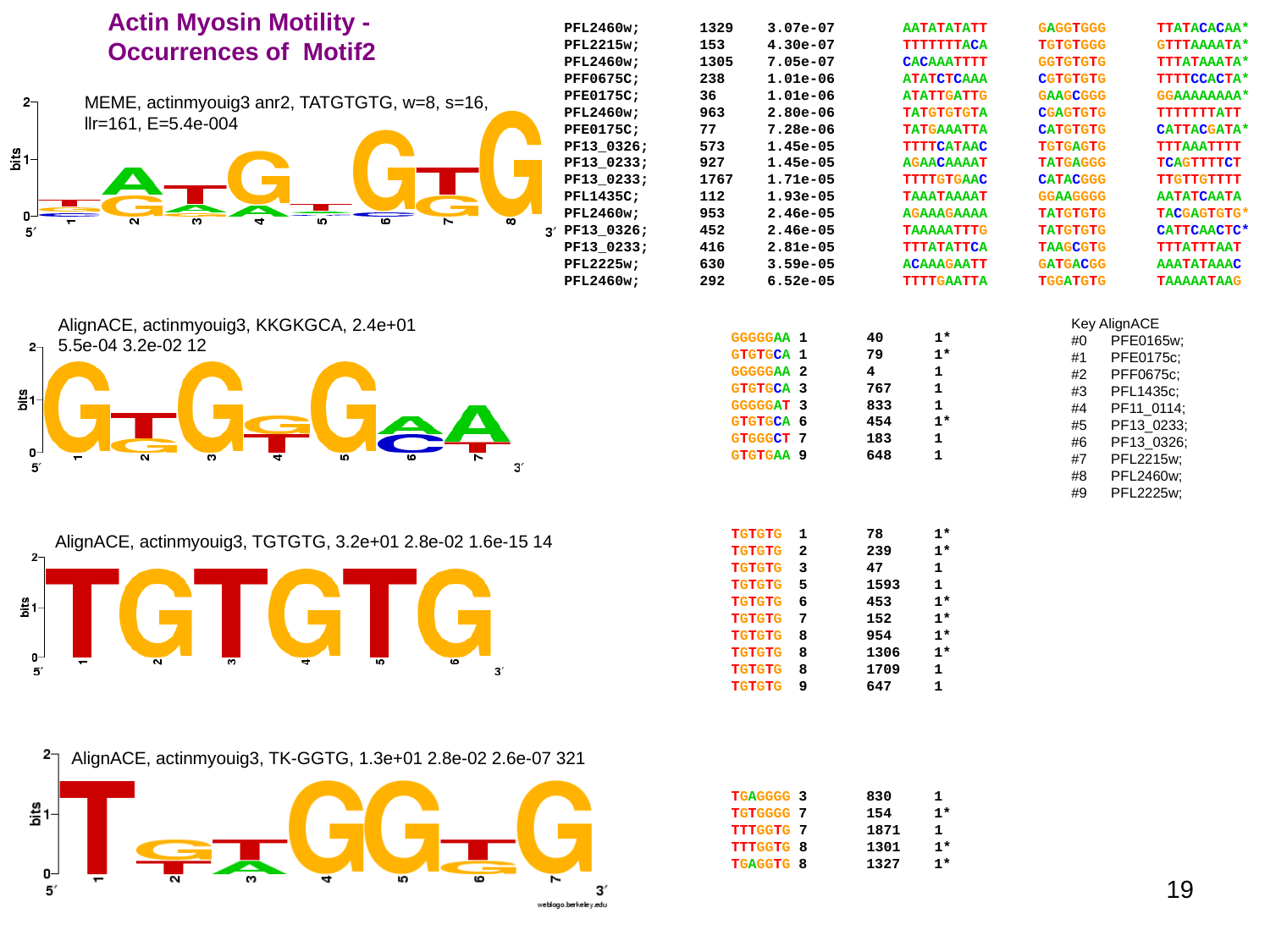

Actin Myosin Motility -
Occurrences of Motif2
PFL2460w; 1329 3.07e-07 AATATATATT GAGGTGGG TTATACACAA*
PFL2215w; 153 4.30e-07 TTTTTTTACA TGTGTGGG GTTTAAAATA*
PFL2460w; 1305 7.05e-07 CACAAATTTT GGTGTGTG TTTATAAATA*
PFF0675C; 238 1.01e-06 ATATCTCAAA CGTGTGTG TTTTCCACTA*
PFE0175C; 36 1.01e-06 ATATTGATTG GAAGCGGG GGAAAAAAAA*
PFL2460w; 963 2.80e-06 TATGTGTGTA CGAGTGTG TTTTTTTATT
PFE0175C; 77 7.28e-06 TATGAAATTA CATGTGTG CATTACGATA*
PF13_0326; 573 1.45e-05 TTTTCATAAC TGTGAGTG TTTAAATTTT
PF13_0233; 927 1.45e-05 AGAACAAAAT TATGAGGG TCAGTTTTCT
PF13_0233; 1767 1.71e-05 TTTTGTGAAC CATACGGG TTGTTGTTTT
PFL1435C; 112 1.93e-05 TAAATAAAAT GGAAGGGG AATATCAATA
PFL2460w; 953 2.46e-05 AGAAAGAAAA TATGTGTG TACGAGTGTG*
PF13_0326; 452 2.46e-05 TAAAAATTTG TATGTGTG CATTCAACTC*
PF13_0233; 416 2.81e-05 TTTATATTCA TAAGCGTG TTTATTTAAT
PFL2225w; 630 3.59e-05 ACAAAGAATT GATGACGG AAATATAAAC
PFL2460w; 292 6.52e-05 TTTTGAATTA TGGATGTG TAAAAATAAG
MEME, actinmyouig3 anr2, TATGTGTG, w=8, s=16,
llr=161, E=5.4e-004
AlignACE, actinmyouig3, KKGKGCA, 2.4e+01
5.5e-04 3.2e-02 12
Key AlignACE
#0 PFE0165w;
#1 PFE0175c;
#2 PFF0675c;
#3 PFL1435c;
#4 PF11_0114;
#5 PF13_0233;
#6 PF13_0326;
#7 PFL2215w;
#8 PFL2460w;
#9 PFL2225w;
GGGGGAA 1 40 1*
GTGTGCA 1 79 1*
GGGGGAA 2 4 1
GTGTGCA 3 767 1
GGGGGAT 3 833 1
GTGTGCA 6 454 1*
GTGGGCT 7 183 1
GTGTGAA 9 648 1
TGTGTG 1 78 1*
TGTGTG 2 239 1*
TGTGTG 3 47 1
TGTGTG 5 1593 1
TGTGTG 6 453 1*
TGTGTG 7 152 1*
TGTGTG 8 954 1*
TGTGTG 8 1306 1*
TGTGTG 8 1709 1
TGTGTG 9 647 1
AlignACE, actinmyouig3, TGTGTG, 3.2e+01 2.8e-02 1.6e-15 14
AlignACE, actinmyouig3, TK-GGTG, 1.3e+01 2.8e-02 2.6e-07 321
TGAGGGG 3 830 1
TGTGGGG 7 154 1*
TTTGGTG 7 1871 1
TTTGGTG 8 1301 1*
TGAGGTG 8 1327 1*
19

## Slide 20
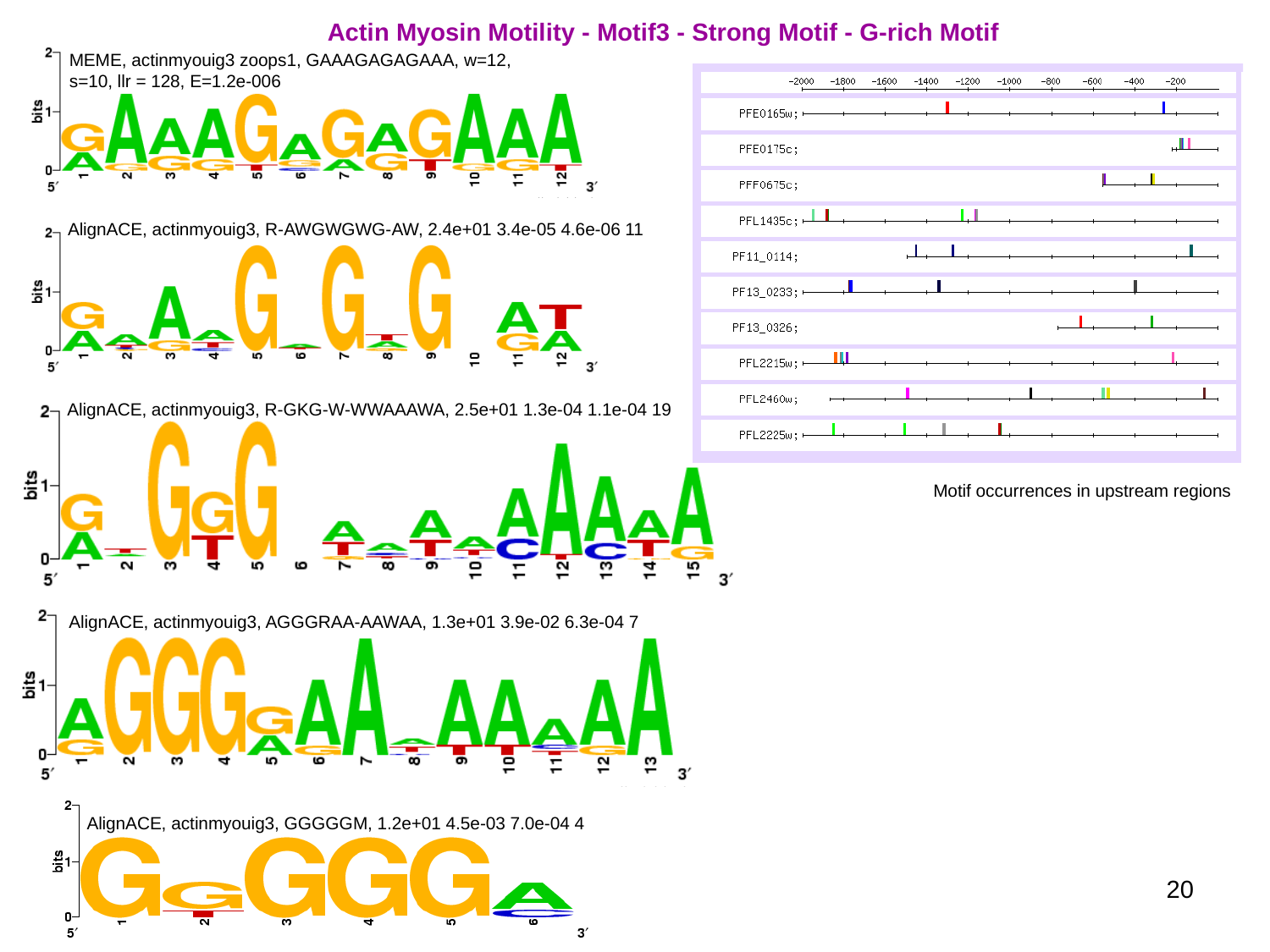

Actin Myosin Motility - Motif3 - Strong Motif - G-rich Motif
MEME, actinmyouig3 zoops1, GAAAGAGAGAAA, w=12,
s=10, llr = 128, E=1.2e-006
AlignACE, actinmyouig3, R-AWGWGWG-AW, 2.4e+01 3.4e-05 4.6e-06 11
AlignACE, actinmyouig3, R-GKG-W-WWAAAWA, 2.5e+01 1.3e-04 1.1e-04 19
AlignACE, actinmyouig3, AGGGRAA-AAWAA, 1.3e+01 3.9e-02 6.3e-04 7
AlignACE, actinmyouig3, GGGGGM, 1.2e+01 4.5e-03 7.0e-04 4
Motif occurrences in upstream regions
20

## Slide 21
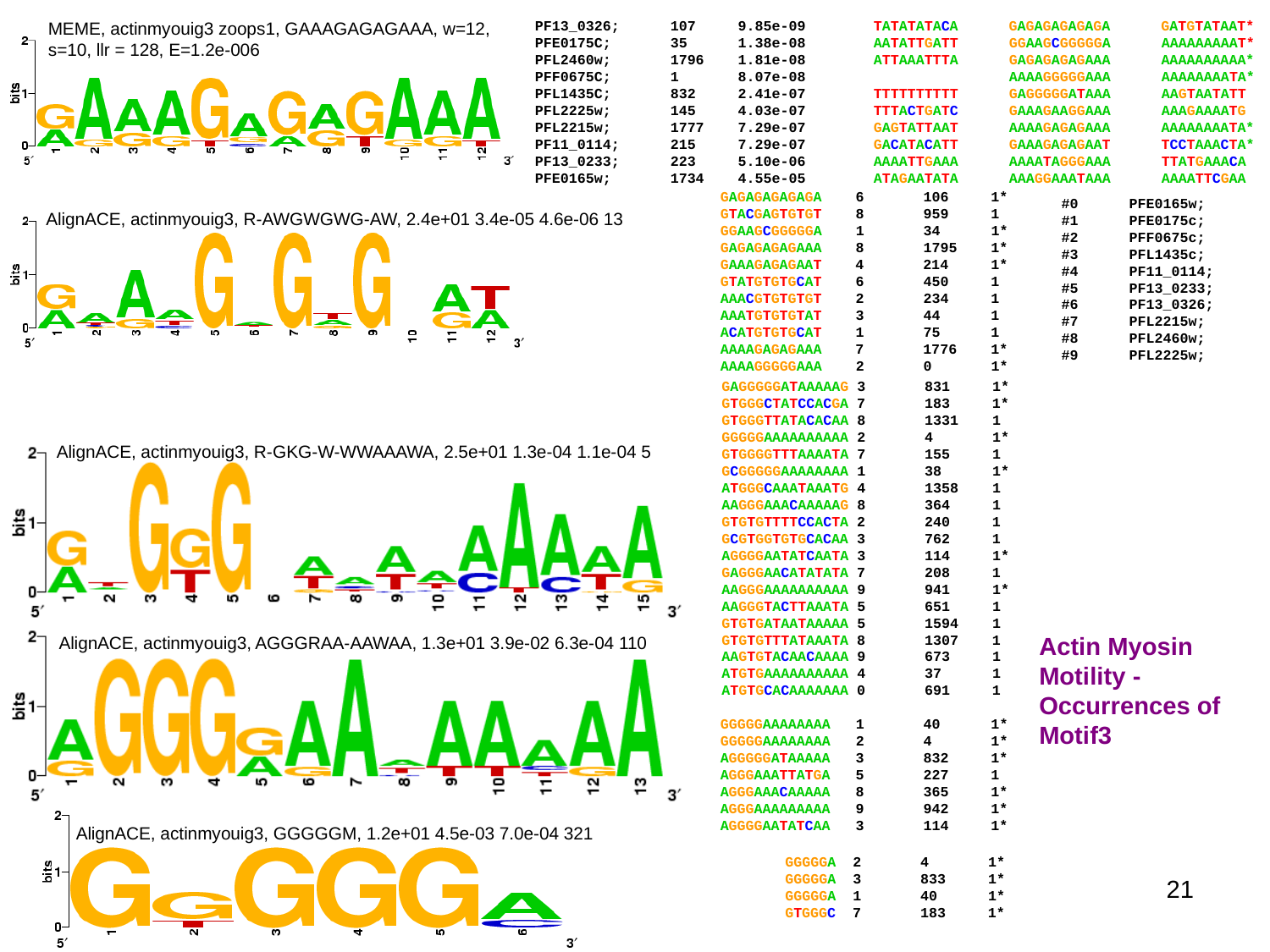

MEME, actinmyouig3 zoops1, GAAAGAGAGAAA, w=12,
s=10, llr = 128, E=1.2e-006
PF13_0326; 107 9.85e-09 TATATATACA GAGAGAGAGAGA GATGTATAAT*
PFE0175C; 35 1.38e-08 AATATTGATT GGAAGCGGGGGA AAAAAAAAAT*
PFL2460w; 1796 1.81e-08 ATTAAATTTA GAGAGAGAGAAA AAAAAAAAAA*
PFF0675C; 1 8.07e-08 AAAAGGGGGAAA AAAAAAAATA*
PFL1435C; 832 2.41e-07 TTTTTTTTTT GAGGGGGATAAA AAGTAATATT
PFL2225w; 145 4.03e-07 TTTACTGATC GAAAGAAGGAAA AAAGAAAATG
PFL2215w; 1777 7.29e-07 GAGTATTAAT AAAAGAGAGAAA AAAAAAAATA*
PF11_0114; 215 7.29e-07 GACATACATT GAAAGAGAGAAT TCCTAAACTA*
PF13_0233; 223 5.10e-06 AAAATTGAAA AAAATAGGGAAA TTATGAAACA
PFE0165w; 1734 4.55e-05 ATAGAATATA AAAGGAAATAAA AAAATTCGAA
GAGAGAGAGAGA 6 106 1*
GTACGAGTGTGT 8 959 1
GGAAGCGGGGGA 1 34 1*
GAGAGAGAGAAA 8 1795 1*
GAAAGAGAGAAT 4 214 1*
GTATGTGTGCAT 6 450 1
AAACGTGTGTGT 2 234 1
AAATGTGTGTAT 3 44 1
ACATGTGTGCAT 1 75 1
AAAAGAGAGAAA 7 1776 1*
AAAAGGGGGAAA 2 0 1*
#0 PFE0165w;
#1 PFE0175c;
#2 PFF0675c;
#3 PFL1435c;
#4 PF11_0114;
#5 PF13_0233;
#6 PF13_0326;
#7 PFL2215w;
#8 PFL2460w;
#9 PFL2225w;
AlignACE, actinmyouig3, R-AWGWGWG-AW, 2.4e+01 3.4e-05 4.6e-06 13
GAGGGGGATAAAAAG 3 831 1*
GTGGGCTATCCACGA 7 183 1*
GTGGGTTATACACAA 8 1331 1
GGGGGAAAAAAAAAA 2 4 1*
GTGGGGTTTAAAATA 7 155 1
GCGGGGGAAAAAAAA 1 38 1*
ATGGGCAAATAAATG 4 1358 1
AAGGGAAACAAAAAG 8 364 1
GTGTGTTTTCCACTA 2 240 1
GCGTGGTGTGCACAA 3 762 1
AGGGGAATATCAATA 3 114 1*
GAGGGAACATATATA 7 208 1
AAGGGAAAAAAAAAA 9 941 1*
AAGGGTACTTAAATA 5 651 1
GTGTGATAATAAAAA 5 1594 1
GTGTGTTTATAAATA 8 1307 1
AAGTGTACAACAAAA 9 673 1
ATGTGAAAAAAAAAA 4 37 1
ATGTGCACAAAAAAA 0 691 1
AlignACE, actinmyouig3, R-GKG-W-WWAAAWA, 2.5e+01 1.3e-04 1.1e-04 5
Actin Myosin
Motility -
Occurrences of
Motif3
AlignACE, actinmyouig3, AGGGRAA-AAWAA, 1.3e+01 3.9e-02 6.3e-04 110
GGGGGAAAAAAAA 1 40 1*
GGGGGAAAAAAAA 2 4 1*
AGGGGGATAAAAA 3 832 1*
AGGGAAATTATGA 5 227 1
AGGGAAACAAAAA 8 365 1*
AGGGAAAAAAAAA 9 942 1*
AGGGGAATATCAA 3 114 1*
AlignACE, actinmyouig3, GGGGGM, 1.2e+01 4.5e-03 7.0e-04 321
GGGGGA 2 4 1*
GGGGGA 3 833 1*
GGGGGA 1 40 1*
GTGGGC 7 183 1*
21

## Slide 22
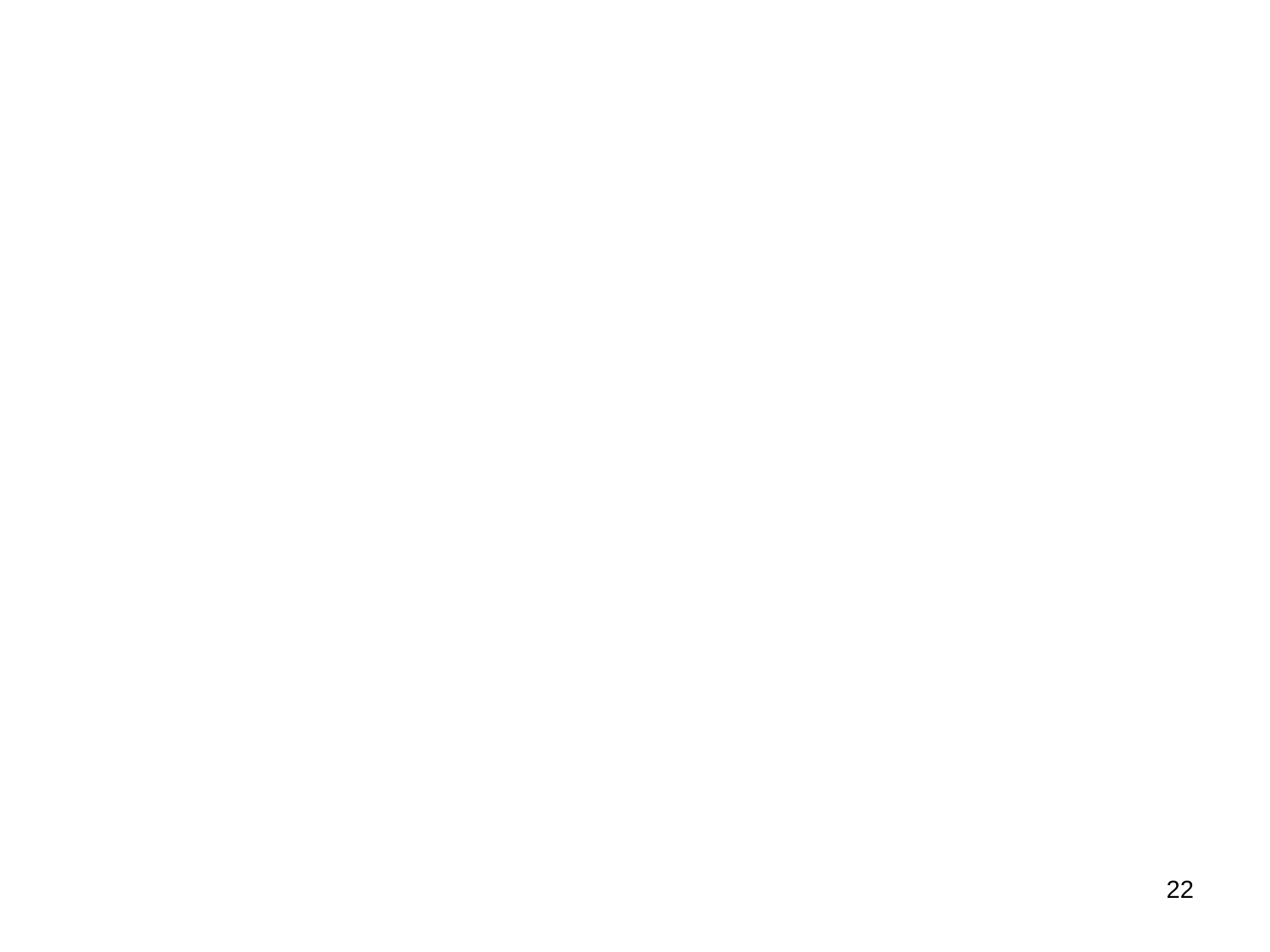

22
