# Supplementary material for: Identification of putative regulatory motifs in the upstream regions of co-expressed functional groups of genes in Plasmodium falciparum
Source: BMC Genomics. 2009 Jan 13;10:18. doi: 10.1186/1471-2164-10-18 (PMC2662883; doi:10.1186/1471-2164-10-18)
Supplement: Additional file 4 — Over-represented upstream motifs identified for the single functional group of genes expressed during the early ring stage. Over-represented motif sets identified for the functional group are given. [file 1471-2164-10-18-S4.ppt]

## Slide 1
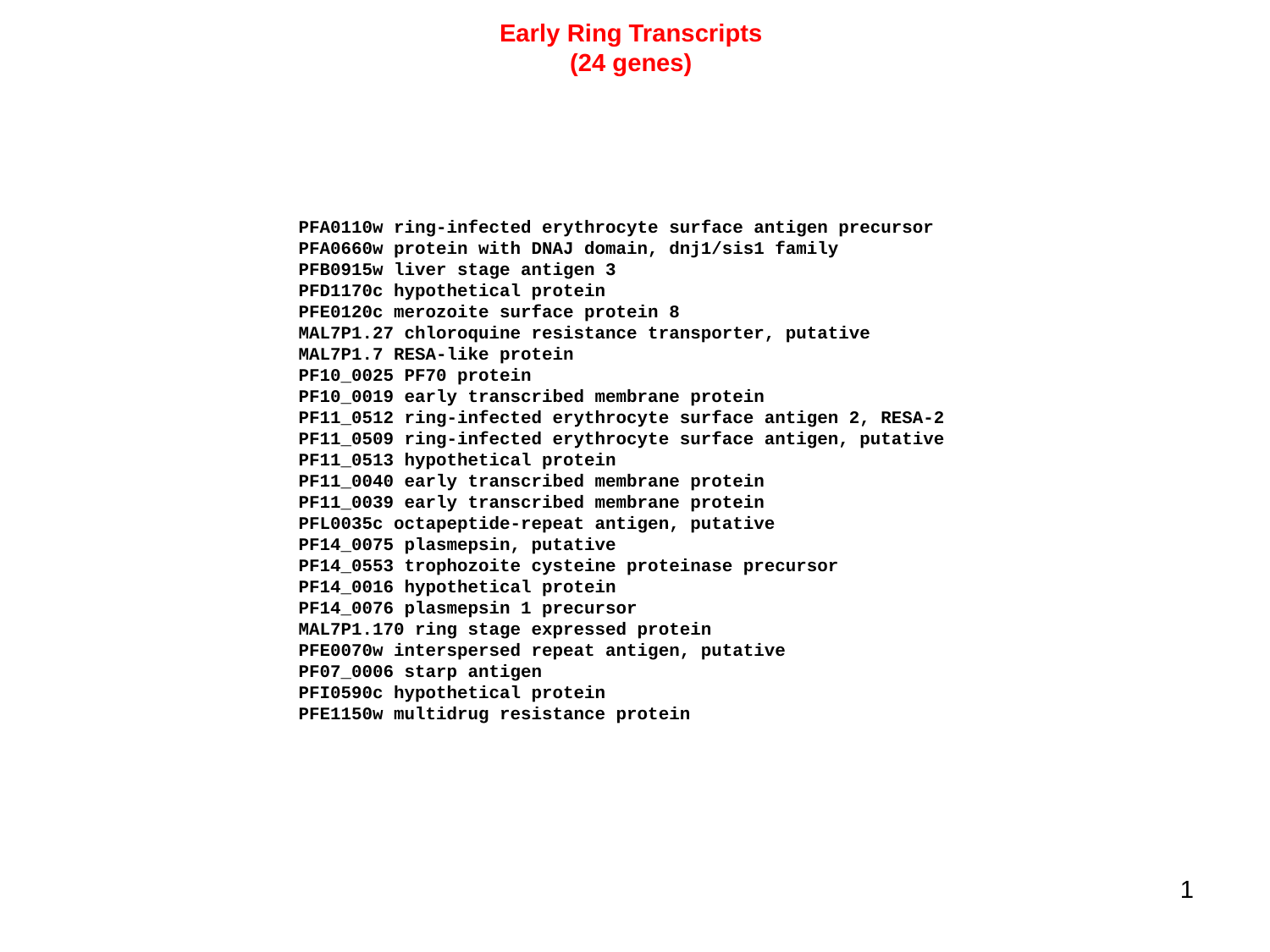

Early Ring Transcripts
(24 genes)
PFA0110w ring-infected erythrocyte surface antigen precursor
PFA0660w protein with DNAJ domain, dnj1/sis1 family
PFB0915w liver stage antigen 3
PFD1170c hypothetical protein
PFE0120c merozoite surface protein 8
MAL7P1.27 chloroquine resistance transporter, putative
MAL7P1.7 RESA-like protein
PF10_0025 PF70 protein
PF10_0019 early transcribed membrane protein
PF11_0512 ring-infected erythrocyte surface antigen 2, RESA-2
PF11_0509 ring-infected erythrocyte surface antigen, putative
PF11_0513 hypothetical protein
PF11_0040 early transcribed membrane protein
PF11_0039 early transcribed membrane protein
PFL0035c octapeptide-repeat antigen, putative
PF14_0075 plasmepsin, putative
PF14_0553 trophozoite cysteine proteinase precursor
PF14_0016 hypothetical protein
PF14_0076 plasmepsin 1 precursor
MAL7P1.170 ring stage expressed protein
PFE0070w interspersed repeat antigen, putative
PF07_0006 starp antigen
PFI0590c hypothetical protein
PFE1150w multidrug resistance protein
1

## Slide 2
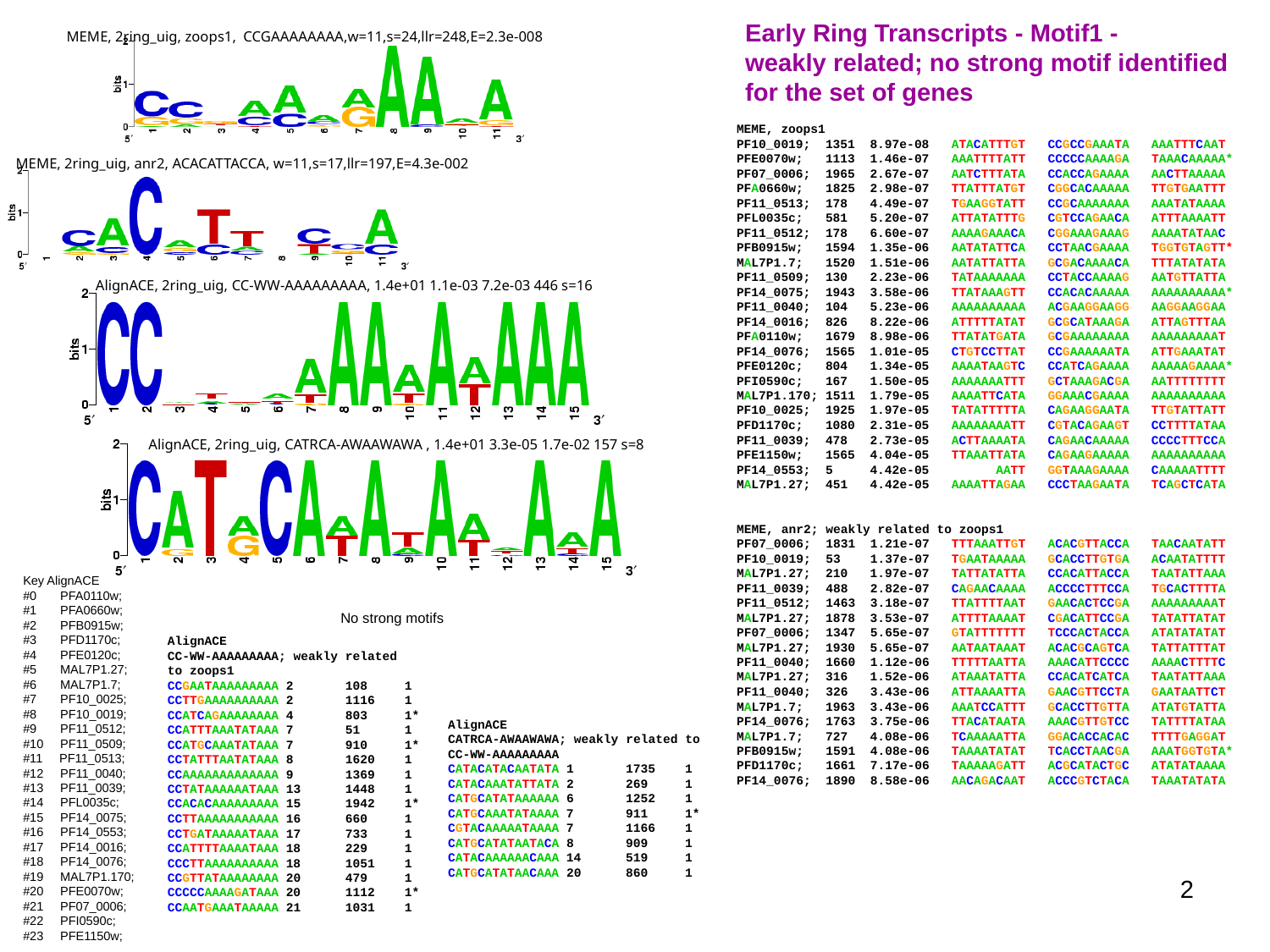

Early Ring Transcripts - Motif1 -
weakly related; no strong motif identified
for the set of genes
MEME, 2ring_uig, zoops1, CCGAAAAAAAA,w=11,s=24,llr=248,E=2.3e-008
MEME, 2ring_uig, anr2, ACACATTACCA, w=11,s=17,llr=197,E=4.3e-002
AlignACE, 2ring_uig, CC-WW-AAAAAAAAA, 1.4e+01 1.1e-03 7.2e-03 446 s=16
AlignACE, 2ring_uig, CATRCA-AWAAWAWA , 1.4e+01 3.3e-05 1.7e-02 157 s=8
MEME, zoops1
PF10_0019; 1351 8.97e-08 ATACATTTGT CCGCCGAAATA AAATTTCAAT
PFE0070w; 1113 1.46e-07 AAATTTTATT CCCCCAAAAGA TAAACAAAAA*
PF07_0006; 1965 2.67e-07 AATCTTTATA CCACCAGAAAA AACTTAAAAA
PFA0660w; 1825 2.98e-07 TTATTTATGT CGGCACAAAAA TTGTGAATTT
PF11_0513; 178 4.49e-07 TGAAGGTATT CCGCAAAAAAA AAATATAAAA
PFL0035c; 581 5.20e-07 ATTATATTTG CGTCCAGAACA ATTTAAAATT
PF11_0512; 178 6.60e-07 AAAAGAAACA CGGAAAGAAAG AAAATATAAC
PFB0915w; 1594 1.35e-06 AATATATTCA CCTAACGAAAA TGGTGTAGTT*
MAL7P1.7; 1520 1.51e-06 AATATTATTA GCGACAAAACA TTTATATATA
PF11_0509; 130 2.23e-06 TATAAAAAAA CCTACCAAAAG AATGTTATTA
PF14_0075; 1943 3.58e-06 TTATAAAGTT CCACACAAAAA AAAAAAAAAA*
PF11_0040; 104 5.23e-06 AAAAAAAAAA ACGAAGGAAGG AAGGAAGGAA
PF14_0016; 826 8.22e-06 ATTTTTATAT GCGCATAAAGA ATTAGTTTAA
PFA0110w; 1679 8.98e-06 TTATATGATA GCGAAAAAAAA AAAAAAAAAT
PF14_0076; 1565 1.01e-05 CTGTCCTTAT CCGAAAAAATA ATTGAAATAT
PFE0120c; 804 1.34e-05 AAAATAAGTC CCATCAGAAAA AAAAAGAAAA*
PFI0590c; 167 1.50e-05 AAAAAAATTT GCTAAAGACGA AATTTTTTTT
MAL7P1.170; 1511 1.79e-05 AAAATTCATA GGAAACGAAAA AAAAAAAAAA
PF10_0025; 1925 1.97e-05 TATATTTTTA CAGAAGGAATA TTGTATTATT
PFD1170c; 1080 2.31e-05 AAAAAAAATT CGTACAGAAGT CCTTTTATAA
PF11_0039; 478 2.73e-05 ACTTAAAATA CAGAACAAAAA CCCCTTTCCA
PFE1150w; 1565 4.04e-05 TTAAATTATA CAGAAGAAAAA AAAAAAAAAA
PF14_0553; 5 4.42e-05 AATT GGTAAAGAAAA CAAAAATTTT
MAL7P1.27; 451 4.42e-05 AAAATTAGAA CCCTAAGAATA TCAGCTCATA
MEME, anr2; weakly related to zoops1
PF07_0006; 1831 1.21e-07 TTTAAATTGT ACACGTTACCA TAACAATATT
PF10_0019; 53 1.37e-07 TGAATAAAAA GCACCTTGTGA ACAATATTTT
MAL7P1.27; 210 1.97e-07 TATTATATTA CCACATTACCA TAATATTAAA
PF11_0039; 488 2.82e-07 CAGAACAAAA ACCCCTTTCCA TGCACTTTTA
PF11_0512; 1463 3.18e-07 TTATTTTAAT GAACACTCCGA AAAAAAAAAT
MAL7P1.27; 1878 3.53e-07 ATTTTAAAAT CGACATTCCGA TATATTATAT
PF07_0006; 1347 5.65e-07 GTATTTTTTT TCCCACTACCA ATATATATAT
MAL7P1.27; 1930 5.65e-07 AATAATAAAT ACACGCAGTCA TATTATTTAT
PF11_0040; 1660 1.12e-06 TTTTTAATTA AAACATTCCCC AAAACTTTTC
MAL7P1.27; 316 1.52e-06 ATAAATATTA CCACATCATCA TAATATTAAA
PF11_0040; 326 3.43e-06 ATTAAAATTA GAACGTTCCTA GAATAATTCT
MAL7P1.7; 1963 3.43e-06 AAATCCATTT GCACCTTGTTA ATATGTATTA
PF14_0076; 1763 3.75e-06 TTACATAATA AAACGTTGTCC TATTTTATAA
MAL7P1.7; 727 4.08e-06 TCAAAAATTA GGACACCACAC TTTTGAGGAT
PFB0915w; 1591 4.08e-06 TAAAATATAT TCACCTAACGA AAATGGTGTA*
PFD1170c; 1661 7.17e-06 TAAAAAGATT ACGCATACTGC ATATATAAAA
PF14_0076; 1890 8.58e-06 AACAGACAAT ACCCGTCTACA TAAATATATA
Key AlignACE
#0 PFA0110w;
#1 PFA0660w;
#2 PFB0915w;
#3 PFD1170c;
#4 PFE0120c;
#5 MAL7P1.27;
#6 MAL7P1.7;
#7 PF10_0025;
#8 PF10_0019;
#9 PF11_0512;
#10 PF11_0509;
#11 PF11_0513;
#12 PF11_0040;
#13 PF11_0039;
#14 PFL0035c;
#15 PF14_0075;
#16 PF14_0553;
#17 PF14_0016;
#18 PF14_0076;
#19 MAL7P1.170;
#20 PFE0070w;
#21 PF07_0006;
#22 PFI0590c;
#23 PFE1150w;
No strong motifs
AlignACE
CC-WW-AAAAAAAAA; weakly related
to zoops1
CCGAATAAAAAAAAA 2 108 1
CCTTGAAAAAAAAAA 2 1116 1
CCATCAGAAAAAAAA 4 803 1*
CCATTTAAATATAAA 7 51 1
CCATGCAAATATAAA 7 910 1*
CCTATTTAATATAAA 8 1620 1
CCAAAAAAAAAAAAA 9 1369 1
CCTATAAAAAATAAA 13 1448 1
CCACACAAAAAAAAA 15 1942 1*
CCTTAAAAAAAAAAA 16 660 1
CCTGATAAAAATAAA 17 733 1
CCATTTTAAAATAAA 18 229 1
CCCTTAAAAAAAAAA 18 1051 1
CCGTTATAAAAAAAA 20 479 1
CCCCCAAAAGATAAA 20 1112 1*
CCAATGAAATAAAAA 21 1031 1
AlignACE
CATRCA-AWAAWAWA; weakly related to
CC-WW-AAAAAAAAA
CATACATACAATATA 1 1735 1
CATACAAATATTATA 2 269 1
CATGCATATAAAAAA 6 1252 1
CATGCAAATATAAAA 7 911 1*
CGTACAAAAATAAAA 7 1166 1
CATGCATATAATACA 8 909 1
CATACAAAAAACAAA 14 519 1
CATGCATATAACAAA 20 860 1
2

## Slide 3
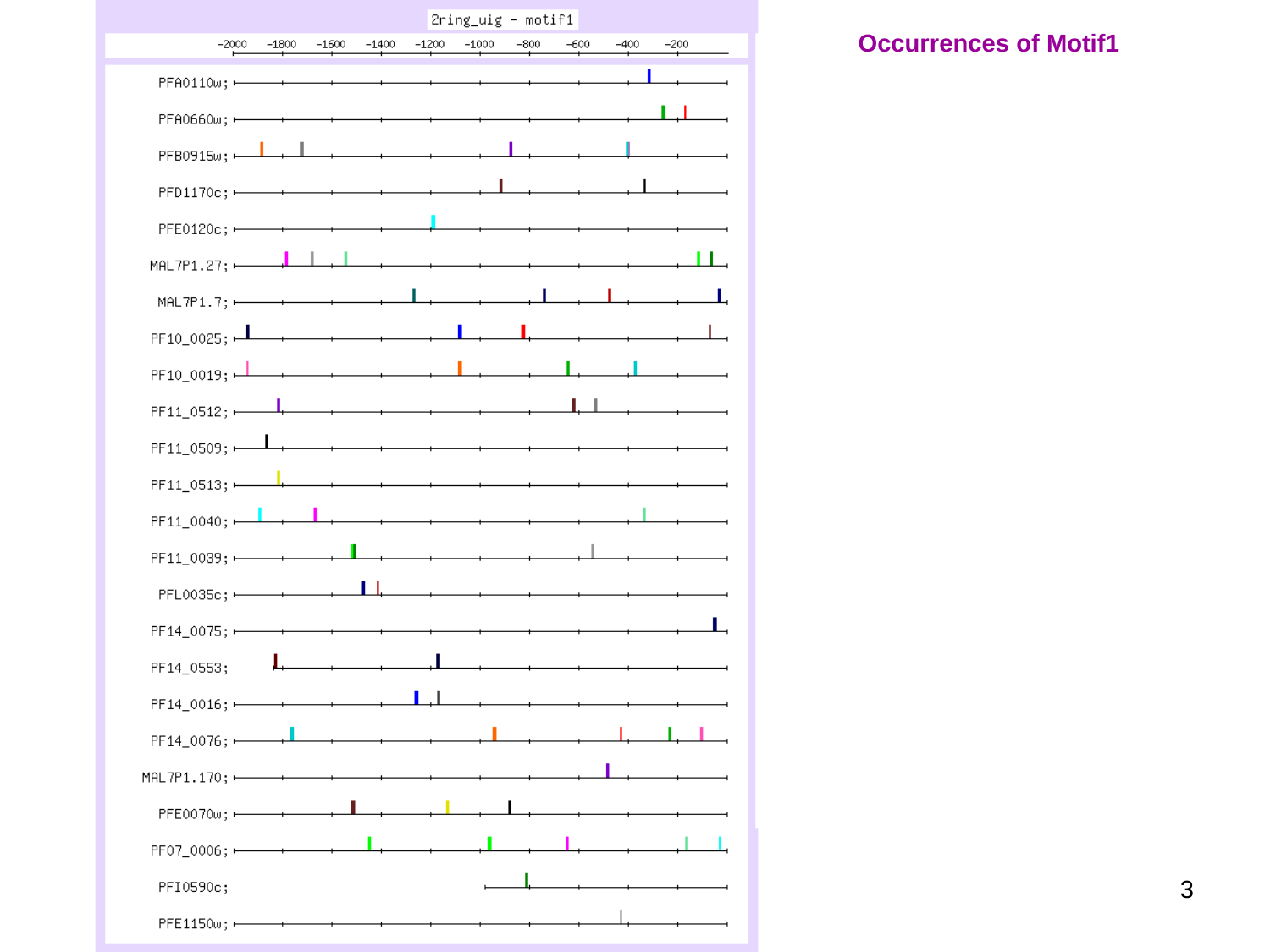

Occurrences of Motif1
3

## Slide 4
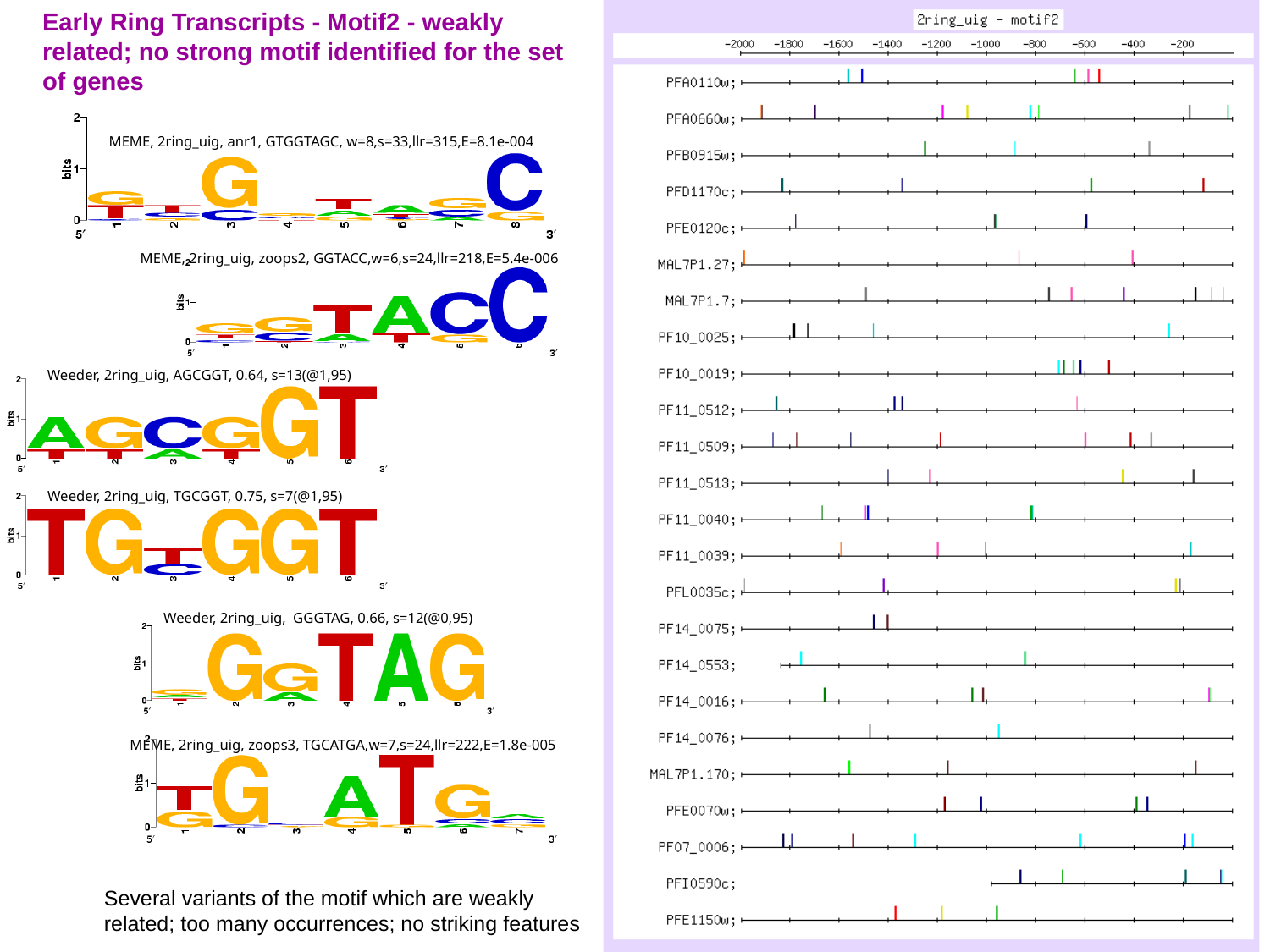

Early Ring Transcripts - Motif2 - weakly
related; no strong motif identified for the set
of genes
MEME, 2ring_uig, anr1, GTGGTAGC, w=8,s=33,llr=315,E=8.1e-004
MEME, 2ring_uig, zoops2, GGTACC,w=6,s=24,llr=218,E=5.4e-006
Weeder, 2ring_uig, AGCGGT, 0.64, s=13(@1,95)
Weeder, 2ring_uig, TGCGGT, 0.75, s=7(@1,95)
Weeder, 2ring_uig, GGGTAG, 0.66, s=12(@0,95)
MEME, 2ring_uig, zoops3, TGCATGA,w=7,s=24,llr=222,E=1.8e-005
4
Several variants of the motif which are weakly
related; too many occurrences; no striking features

## Slide 5
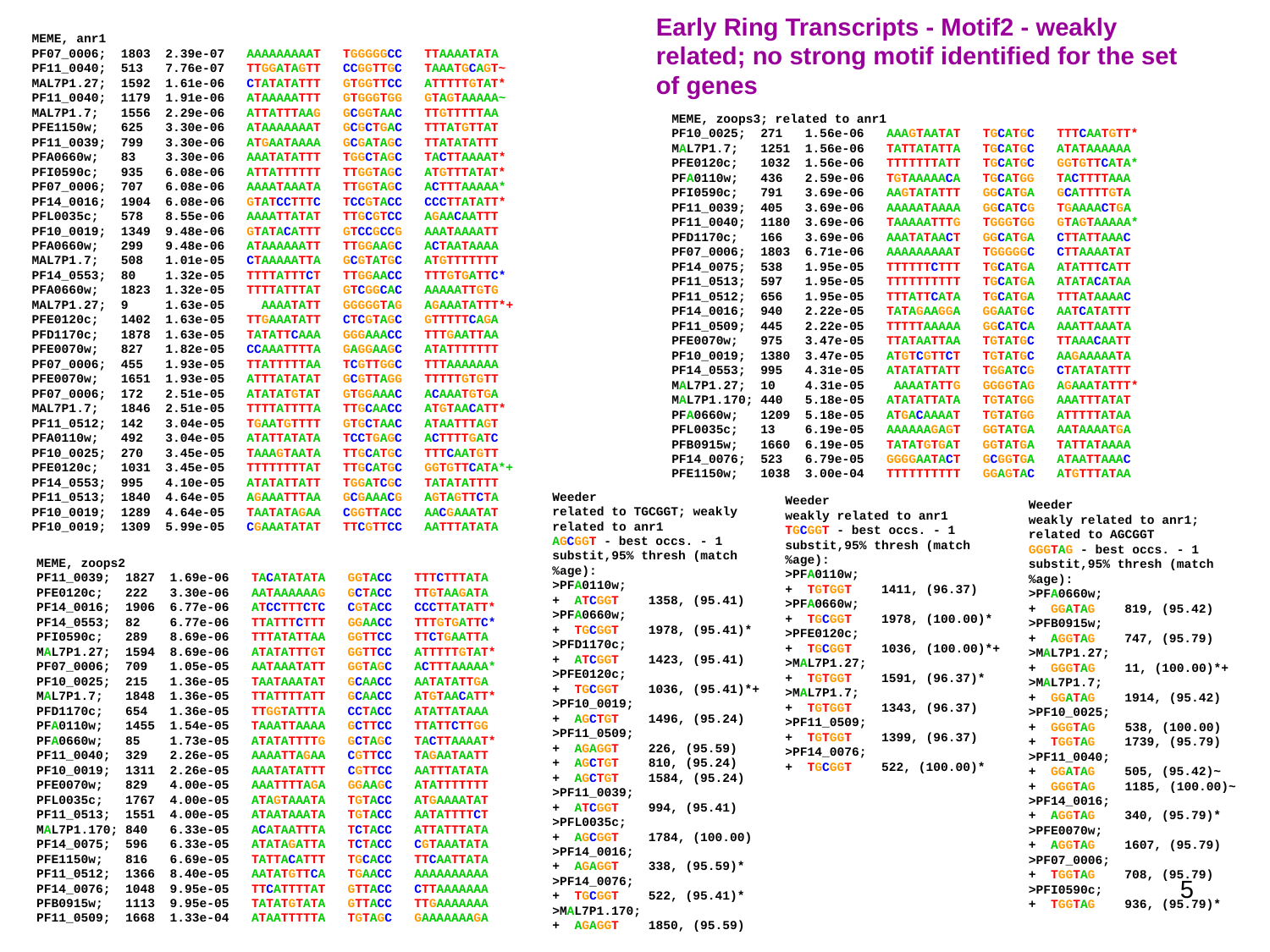

Early Ring Transcripts - Motif2 - weakly
related; no strong motif identified for the set
of genes
MEME, anr1
PF07_0006; 1803 2.39e-07 AAAAAAAAAT TGGGGGCC TTAAAATATA
PF11_0040; 513 7.76e-07 TTGGATAGTT CCGGTTGC TAAATGCAGT~
MAL7P1.27; 1592 1.61e-06 CTATATATTT GTGGTTCC ATTTTTGTAT*
PF11_0040; 1179 1.91e-06 ATAAAAATTT GTGGGTGG GTAGTAAAAA~
MAL7P1.7; 1556 2.29e-06 ATTATTTAAG GCGGTAAC TTGTTTTTAA
PFE1150w; 625 3.30e-06 ATAAAAAAAT GCGCTGAC TTTATGTTAT
PF11_0039; 799 3.30e-06 ATGAATAAAA GCGATAGC TTATATATTT
PFA0660w; 83 3.30e-06 AAATATATTT TGGCTAGC TACTTAAAAT*
PFI0590c; 935 6.08e-06 ATTATTTTTT TTGGTAGC ATGTTTATAT*
PF07_0006; 707 6.08e-06 AAAATAAATA TTGGTAGC ACTTTAAAAA*
PF14_0016; 1904 6.08e-06 GTATCCTTTC TCCGTACC CCCTTATATT*
PFL0035c; 578 8.55e-06 AAAATTATAT TTGCGTCC AGAACAATTT
PF10_0019; 1349 9.48e-06 GTATACATTT GTCCGCCG AAATAAAATT
PFA0660w; 299 9.48e-06 ATAAAAAATT TTGGAAGC ACTAATAAAA
MAL7P1.7; 508 1.01e-05 CTAAAAATTA GCGTATGC ATGTTTTTTT
PF14_0553; 80 1.32e-05 TTTTATTTCT TTGGAACC TTTGTGATTC*
PFA0660w; 1823 1.32e-05 TTTTATTTAT GTCGGCAC AAAAATTGTG
MAL7P1.27; 9 1.63e-05 AAAATATT GGGGGTAG AGAAATATTT*+
PFE0120c; 1402 1.63e-05 TTGAAATATT CTCGTAGC GTTTTTCAGA
PFD1170c; 1878 1.63e-05 TATATTCAAA GGGAAACC TTTGAATTAA
PFE0070w; 827 1.82e-05 CCAAATTTTA GAGGAAGC ATATTTTTTT
PF07_0006; 455 1.93e-05 TTATTTTTAA TCGTTGGC TTTAAAAAAA
PFE0070w; 1651 1.93e-05 ATTTATATAT GCGTTAGG TTTTTGTGTT
PF07_0006; 172 2.51e-05 ATATATGTAT GTGGAAAC ACAAATGTGA
MAL7P1.7; 1846 2.51e-05 TTTTATTTTA TTGCAACC ATGTAACATT*
PF11_0512; 142 3.04e-05 TGAATGTTTT GTGCTAAC ATAATTTAGT
PFA0110w; 492 3.04e-05 ATATTATATA TCCTGAGC ACTTTTGATC
PF10_0025; 270 3.45e-05 TAAAGTAATA TTGCATGC TTTCAATGTT
PFE0120c; 1031 3.45e-05 TTTTTTTTAT TTGCATGC GGTGTTCATA*+
PF14_0553; 995 4.10e-05 ATATATTATT TGGATCGC TATATATTTT
PF11_0513; 1840 4.64e-05 AGAAATTTAA GCGAAACG AGTAGTTCTA
PF10_0019; 1289 4.64e-05 TAATATAGAA CGGTTACC AACGAAATAT
PF10_0019; 1309 5.99e-05 CGAAATATAT TTCGTTCC AATTTATATA
MEME, zoops3; related to anr1
PF10_0025; 271 1.56e-06 AAAGTAATAT TGCATGC TTTCAATGTT*
MAL7P1.7; 1251 1.56e-06 TATTATATTA TGCATGC ATATAAAAAA
PFE0120c; 1032 1.56e-06 TTTTTTTATT TGCATGC GGTGTTCATA*
PFA0110w; 436 2.59e-06 TGTAAAAACA TGCATGG TACTTTTAAA
PFI0590c; 791 3.69e-06 AAGTATATTT GGCATGA GCATTTTGTA
PF11_0039; 405 3.69e-06 AAAAATAAAA GGCATCG TGAAAACTGA
PF11_0040; 1180 3.69e-06 TAAAAATTTG TGGGTGG GTAGTAAAAA*
PFD1170c; 166 3.69e-06 AAATATAACT GGCATGA CTTATTAAAC
PF07_0006; 1803 6.71e-06 AAAAAAAAAT TGGGGGC CTTAAAATAT
PF14_0075; 538 1.95e-05 TTTTTTCTTT TGCATGA ATATTTCATT
PF11_0513; 597 1.95e-05 TTTTTTTTTT TGCATGA ATATACATAA
PF11_0512; 656 1.95e-05 TTTATTCATA TGCATGA TTTATAAAAC
PF14_0016; 940 2.22e-05 TATAGAAGGA GGAATGC AATCATATTT
PF11_0509; 445 2.22e-05 TTTTTAAAAA GGCATCA AAATTAAATA
PFE0070w; 975 3.47e-05 TTATAATTAA TGTATGC TTAAACAATT
PF10_0019; 1380 3.47e-05 ATGTCGTTCT TGTATGC AAGAAAAATA
PF14_0553; 995 4.31e-05 ATATATTATT TGGATCG CTATATATTT
MAL7P1.27; 10 4.31e-05 AAAATATTG GGGGTAG AGAAATATTT*
MAL7P1.170; 440 5.18e-05 ATATATTATA TGTATGG AAATTTATAT
PFA0660w; 1209 5.18e-05 ATGACAAAAT TGTATGG ATTTTTATAA
PFL0035c; 13 6.19e-05 AAAAAAGAGT GGTATGA AATAAAATGA
PFB0915w; 1660 6.19e-05 TATATGTGAT GGTATGA TATTATAAAA
PF14_0076; 523 6.79e-05 GGGGAATACT GCGGTGA ATAATTAAAC
PFE1150w; 1038 3.00e-04 TTTTTTTTTT GGAGTAC ATGTTTATAA
Weeder
related to TGCGGT; weakly related to anr1
AGCGGT - best occs. - 1
substit,95% thresh (match
%age):
>PFA0110w;
+ ATCGGT 1358, (95.41)
>PFA0660w;
+ TGCGGT 1978, (95.41)*
>PFD1170c;
+ ATCGGT 1423, (95.41)
>PFE0120c;
+ TGCGGT 1036, (95.41)*+
>PF10_0019;
+ AGCTGT 1496, (95.24)
>PF11_0509;
+ AGAGGT 226, (95.59)
+ AGCTGT 810, (95.24)
+ AGCTGT 1584, (95.24)
>PF11_0039;
+ ATCGGT 994, (95.41)
>PFL0035c;
+ AGCGGT 1784, (100.00)
>PF14_0016;
+ AGAGGT 338, (95.59)*
>PF14_0076;
+ TGCGGT 522, (95.41)*
>MAL7P1.170;
+ AGAGGT 1850, (95.59)
Weeder
weakly related to anr1
TGCGGT - best occs. - 1
substit,95% thresh (match
%age):
>PFA0110w;
+ TGTGGT 1411, (96.37)
>PFA0660w;
+ TGCGGT 1978, (100.00)*
>PFE0120c;
+ TGCGGT 1036, (100.00)*+
>MAL7P1.27;
+ TGTGGT 1591, (96.37)*
>MAL7P1.7;
+ TGTGGT 1343, (96.37)
>PF11_0509;
+ TGTGGT 1399, (96.37)
>PF14_0076;
+ TGCGGT 522, (100.00)*
Weeder
weakly related to anr1; related to AGCGGT
GGGTAG - best occs. - 1 substit,95% thresh (match %age):
>PFA0660w;
+ GGATAG 819, (95.42)
>PFB0915w;
+ AGGTAG 747, (95.79)
>MAL7P1.27;
+ GGGTAG 11, (100.00)*+
>MAL7P1.7;
+ GGATAG 1914, (95.42)
>PF10_0025;
+ GGGTAG 538, (100.00)
+ TGGTAG 1739, (95.79)
>PF11_0040;
+ GGATAG 505, (95.42)~
+ GGGTAG 1185, (100.00)~
>PF14_0016;
+ AGGTAG 340, (95.79)*
>PFE0070w;
+ AGGTAG 1607, (95.79)
>PF07_0006;
+ TGGTAG 708, (95.79)
>PFI0590c;
+ TGGTAG 936, (95.79)*
MEME, zoops2
PF11_0039; 1827 1.69e-06 TACATATATA GGTACC TTTCTTTATA
PFE0120c; 222 3.30e-06 AATAAAAAAG GCTACC TTGTAAGATA
PF14_0016; 1906 6.77e-06 ATCCTTTCTC CGTACC CCCTTATATT*
PF14_0553; 82 6.77e-06 TTATTTCTTT GGAACC TTTGTGATTC*
PFI0590c; 289 8.69e-06 TTTATATTAA GGTTCC TTCTGAATTA
MAL7P1.27; 1594 8.69e-06 ATATATTTGT GGTTCC ATTTTTGTAT*
PF07_0006; 709 1.05e-05 AATAAATATT GGTAGC ACTTTAAAAA*
PF10_0025; 215 1.36e-05 TAATAAATAT GCAACC AATATATTGA
MAL7P1.7; 1848 1.36e-05 TTATTTTATT GCAACC ATGTAACATT*
PFD1170c; 654 1.36e-05 TTGGTATTTA CCTACC ATATTATAAA
PFA0110w; 1455 1.54e-05 TAAATTAAAA GCTTCC TTATTCTTGG
PFA0660w; 85 1.73e-05 ATATATTTTG GCTAGC TACTTAAAAT*
PF11_0040; 329 2.26e-05 AAAATTAGAA CGTTCC TAGAATAATT
PF10_0019; 1311 2.26e-05 AAATATATTT CGTTCC AATTTATATA
PFE0070w; 829 4.00e-05 AAATTTTAGA GGAAGC ATATTTTTTT
PFL0035c; 1767 4.00e-05 ATAGTAAATA TGTACC ATGAAAATAT
PF11_0513; 1551 4.00e-05 ATAATAAATA TGTACC AATATTTTCT
MAL7P1.170; 840 6.33e-05 ACATAATTTA TCTACC ATTATTTATA
PF14_0075; 596 6.33e-05 ATATAGATTA TCTACC CGTAAATATA
PFE1150w; 816 6.69e-05 TATTACATTT TGCACC TTCAATTATA
PF11_0512; 1366 8.40e-05 AATATGTTCA TGAACC AAAAAAAAAA
PF14_0076; 1048 9.95e-05 TTCATTTTAT GTTACC CTTAAAAAAA
PFB0915w; 1113 9.95e-05 TATATGTATA GTTACC TTGAAAAAAA
PF11_0509; 1668 1.33e-04 ATAATTTTTA TGTAGC GAAAAAAAGA
5

## Slide 6
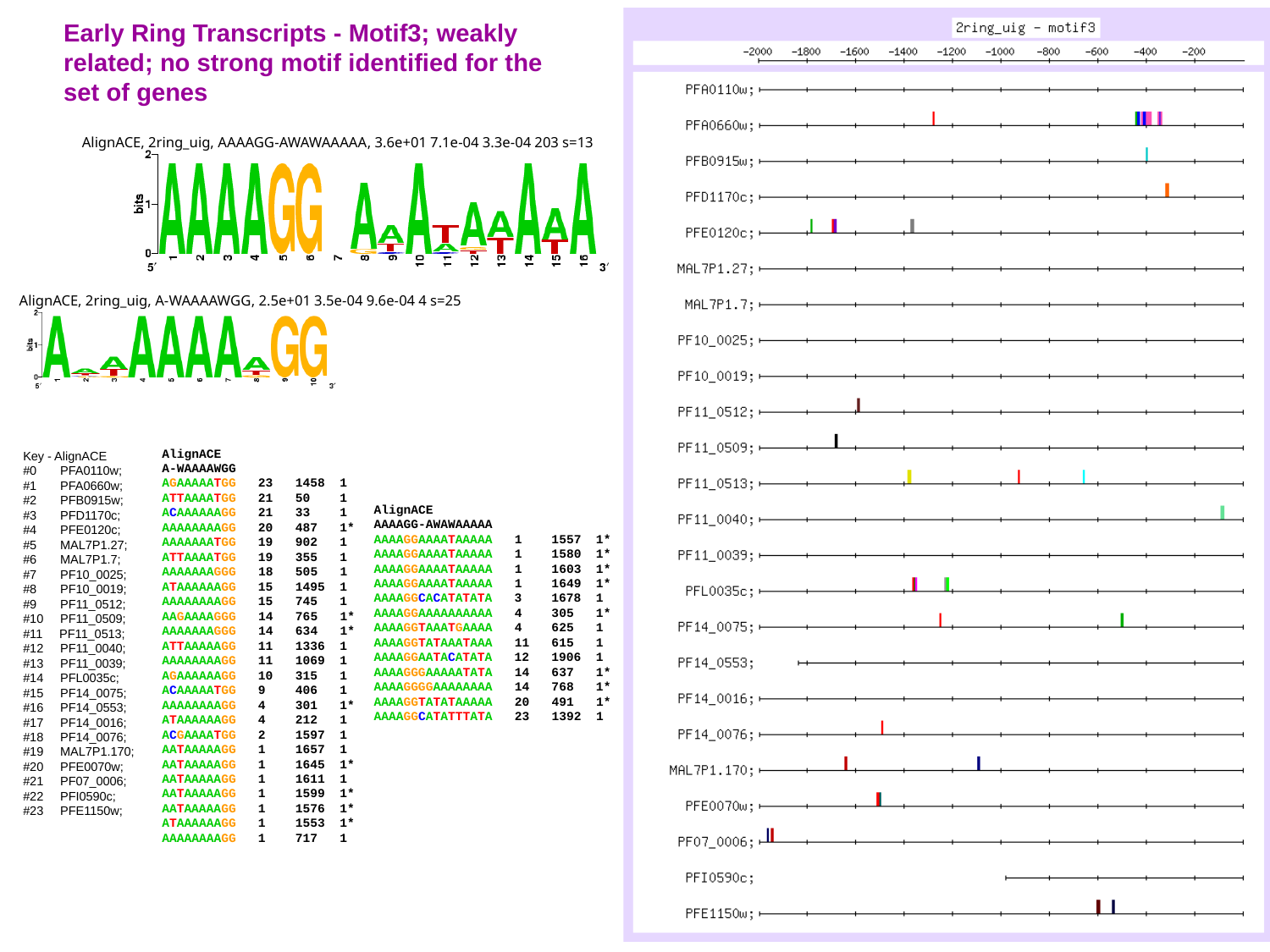

Early Ring Transcripts - Motif3; weakly
related; no strong motif identified for the
set of genes
AlignACE, 2ring_uig, AAAAGG-AWAWAAAAA, 3.6e+01 7.1e-04 3.3e-04 203 s=13
AlignACE, 2ring_uig, A-WAAAAWGG, 2.5e+01 3.5e-04 9.6e-04 4 s=25
AlignACE
A-WAAAAWGG
AGAAAAATGG 23 1458 1
ATTAAAATGG 21 50 1
ACAAAAAAGG 21 33 1
AAAAAAAAGG 20 487 1*
AAAAAAATGG 19 902 1
ATTAAAATGG 19 355 1
AAAAAAAGGG 18 505 1
ATAAAAAAGG 15 1495 1
AAAAAAAAGG 15 745 1
AAGAAAAGGG 14 765 1*
AAAAAAAGGG 14 634 1*
ATTAAAAAGG 11 1336 1
AAAAAAAAGG 11 1069 1
AGAAAAAAGG 10 315 1
ACAAAAATGG 9 406 1
AAAAAAAAGG 4 301 1*
ATAAAAAAGG 4 212 1
ACGAAAATGG 2 1597 1
AATAAAAAGG 1 1657 1
AATAAAAAGG 1 1645 1*
AATAAAAAGG 1 1611 1
AATAAAAAGG 1 1599 1*
AATAAAAAGG 1 1576 1*
ATAAAAAAGG 1 1553 1*
AAAAAAAAGG 1 717 1
Key - AlignACE
#0 PFA0110w;
#1 PFA0660w;
#2 PFB0915w;
#3 PFD1170c;
#4 PFE0120c;
#5 MAL7P1.27;
#6 MAL7P1.7;
#7 PF10_0025;
#8 PF10_0019;
#9 PF11_0512;
#10 PF11_0509;
#11 PF11_0513;
#12 PF11_0040;
#13 PF11_0039;
#14 PFL0035c;
#15 PF14_0075;
#16 PF14_0553;
#17 PF14_0016;
#18 PF14_0076;
#19 MAL7P1.170;
#20 PFE0070w;
#21 PF07_0006;
#22 PFI0590c;
#23 PFE1150w;
AlignACE
AAAAGG-AWAWAAAAA
AAAAGGAAAATAAAAA 1 1557 1*
AAAAGGAAAATAAAAA 1 1580 1*
AAAAGGAAAATAAAAA 1 1603 1*
AAAAGGAAAATAAAAA 1 1649 1*
AAAAGGCACATATATA 3 1678 1
AAAAGGAAAAAAAAAA 4 305 1*
AAAAGGTAAATGAAAA 4 625 1
AAAAGGTATAAATAAA 11 615 1
AAAAGGAATACATATA 12 1906 1
AAAAGGGAAAAATATA 14 637 1*
AAAAGGGGAAAAAAAA 14 768 1*
AAAAGGTATATAAAAA 20 491 1*
AAAAGGCATATTTATA 23 1392 1
6

## Slide 7
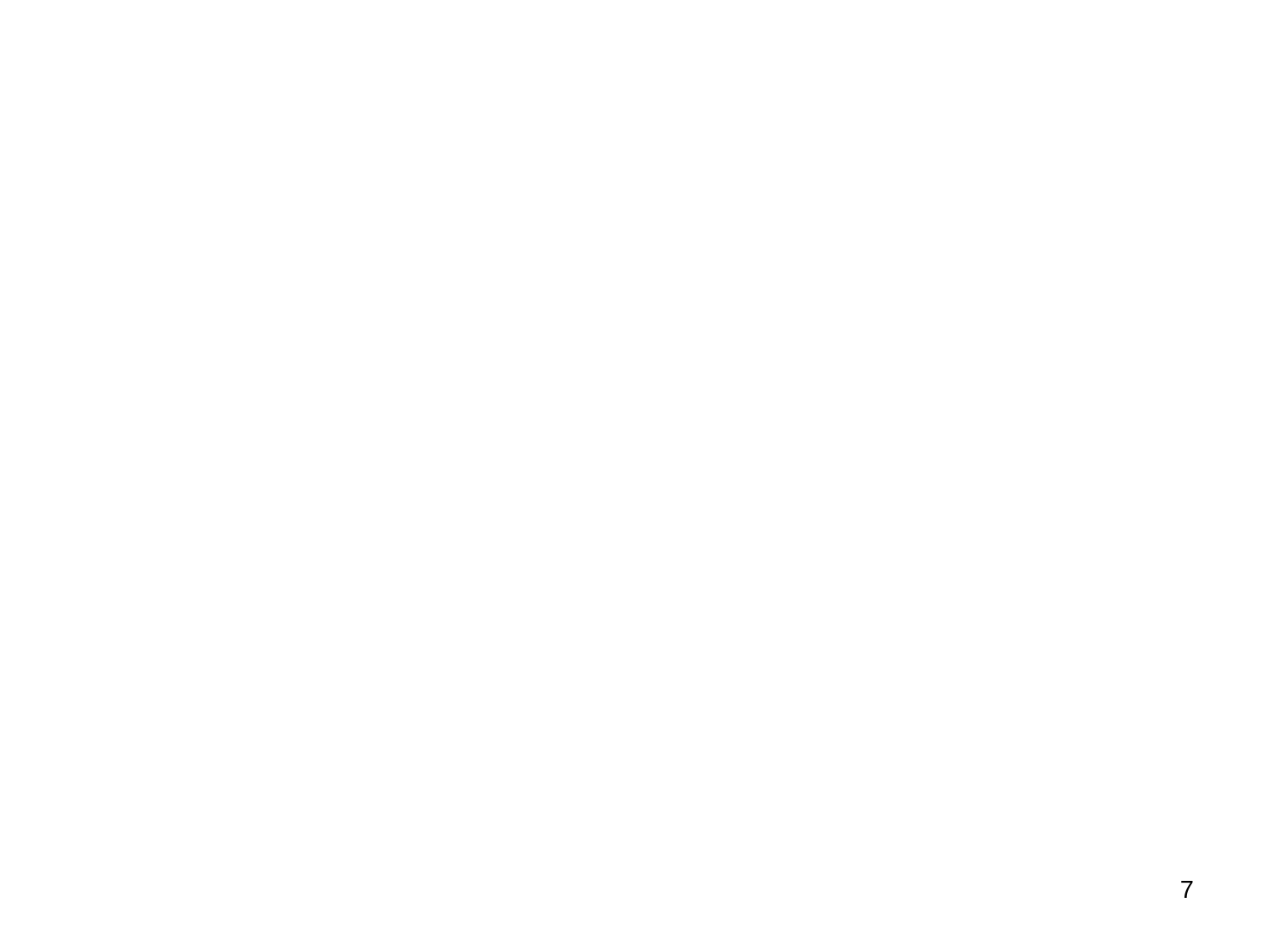

7
