# Supplementary material for: Identification of putative regulatory motifs in the upstream regions of co-expressed functional groups of genes in Plasmodium falciparum
Source: BMC Genomics. 2009 Jan 13;10:18. doi: 10.1186/1471-2164-10-18 (PMC2662883; doi:10.1186/1471-2164-10-18)
Supplement: Additional file 5 — Motif occurrences in the strong motif groups, identified for each of the 13 functional groups of genes, after sorting. These motif occurrences were used to obtain feature maps. [file 1471-2164-10-18-S5.doc]

Additional file 5. Motif occurrences in the strong motif groups identified for each of the 13 functional groups of genes, after sorting.

Motif occurrences in the strong motif groups were sorted (Methods). The sorted motif occurrences for the functional groups discussed under Results, are listed. These were the sorted motif occurrences that were used to obtain the feature maps presented in the study. Each set of motifs, below, was input to the DNA pattern and feature map programs (along with the relevant set of upstream sequences) to obtain a feature map (Methods). This list of sorted motifs is often longer than the number of occurrences marked in the corresponding feature map. This is because the same motif may have been picked: (a) multiple times by the same program (e.g., in 2 related motif-sets identified by AlignACE), or (ii) multiple times by multiple programs (e.g., once each in motif-sets identified by AlignACE and MEME). While motifs listed in this table may be repeated, the occurrences of these motifs in the upstream regions (shown in feature maps) are not. Thus, the latter are fewer in number compared to the former.

transcription machinery (4g,3g,2g,1g)

GGGGGAAAAATAAA

GGGGTGTAATGATAAAAAGGGA

GAGGGGAAAATAAAATAATAAA

GGGGGGGAAAAATAAAATAATA

GTGGGGTCACATTTATATTGAA

AAGGGGAAAAAAAAAAATAAAA

GAGGGGGAAAGTATTAATTATT

GTGGGGTCACA

GAGGGGGGGAA

GTGGGGTGTAA

GTGGGGAGTGA

GTGGAGGGGGA

TTGGGGAGTTA

GGAGGGGGAAA

GGAGGGGGGGA

GGGGAGTGAAA

GTGGGGTGTAA

GGGGAGTTATA

GTGGGGTCACA

TGGGGAGT

TGGGGTGT

TGGGGAGT

TGGGGAG

TGTGGGG

AGAGGGG

TGTGGGG

GGAGGGG

TGTGGGG

GGAGGGGGAAA

GGGGGGGAAAA

AGAGGGGAAAA

GGGGAGTGAAA

AGGGGAAAAAA

GCGGGTCTACAAGAAAAATGAA

GTGGGAAAAAAAAAAAAAAAAA

GAGGGCTCAAAAAAAAAAAAAA

GAGGGCTCAAA

TAGGGAGT

AGAGGGC

GGTGGGAAAAA

GGTTGGGAAAA

AATGGGAAAAA

GGGAAAAAACAAAA

GGGAAAAAAAAAAA

GGGTAAAAAAAAAA

GGGAAAAAAAAAAA

GGGTCACATTTATA

GGGTGAAAATAAAA

GGGAAAAAAAAAAA

GGGAAAAAATAATA

GGTTGGG

GTGGAGACTTA

GTGTGGACCAA

TGTGGAC

TGTAGGC

TGTGGAG

GGTGGAG

GGTGGAAAAAA

AGAGGAAGAAA

AGTGGAAAAAA

AGAGGAAAAAA

GAAGGAAGACA

CATGGAAGAAA

GGTGGAG

GGTGGAAAAAA

GGAGAAAAAAA

AGTGAGAGAAA

AGAGAAGAAAA

AGTGAAGACAA

AGCGAAAAAAA

GCTGAGTAAAA

GCTGAGTAAAA

----------------------------------------------------------------

ribonucleotide synthesis (4g,3g,2g,1g)

TGGGGGGA

CAAGGGGA

CGGGGAGG

TAAGGGGA

TAAGGGGA

TGGGGGGA

CAAGGGGA

CGGGGAGG

TAAGGGGA

TAAGGGGA

AATCGGGGAGGATAAAAA

ATAAGGGGAATTTATATT

ACAAGGGGAAAAAGGAAT

ATAAGGGGATATCAAAAA

AAATGGGGATTTTTAAAA

ATAAGGGGAATT

ACAAGGGGAAAA

ATAAGGGGATAT

GGGGGAAAAAATAA

GAAGGGCC

TAAGGGCA

TAAGGGCA

CCAAGGGA

TAAAGGGA

TAAAGGGA

TAAAGGGA

GAAGGGCC

TAAGGGCA

TAAGGGCA

TAAAGGGA

ATAAGGGCATATTTAAAA

ATAAGGGCACAATAGAAA

AAAGGGAAAAAATAAAAA

ATAAGGGCATAT

ATAAGGGCACAA

CGGTGGCA

TGAAGGCA

GGTAGGCC

CGTGGTGG

TGAGGTCG

CAGGTGCC

TAAAGGCG

CAAGGAGA

CATAGGCA

TGGAGAGG

GGTGGTCA

CGAAGGAA

CGGTGGCA

CAAGGAGA

TGAAGGCA

AAATGGAGAATAATAAAA

AAAAGGAGAAAAAAAAAA

ATATGGAAATTAAAGAGA

AAATGGAGAAAAATAAAA

CCAAGGAGATAT

AAAAGGAGAAAA

GGAGGATAAAAAAA

GGTGGAAAGAAAAA

GTAGGAAAATATAA

GGTAGGCC

GGTGGTCA

GGAGGATAAAAAAA

GGTGGAAAGAAAAA

GGAGAATAATAAAA

GGAGAAAAAAAAAA

GGAGAAAAATAAAA

CGTGGTGG

TGGAGAGG

GAAAGAGG

CGAAGACA

CAAGTGCC

TACGCGCA

----------------------------------------------------------------

dna replication (caca)

CACCCCTTTTTTACACATAA

CACACATTTTTGGAATATGA

TACACACC

TACACACC

ACACACCC

ACACACCT

ACACACCT

ACACACAC

ACACACAC

ACACACAT

ACACACAT

ACACACAT

ACACACAT

ACACACAT

ACACACAT

ACACACAT

GCACACAC

CCACACAT

CCACACAT

TCACACAT

ACACACCA

ACATACAC

ACATACAT

ACATACAT

ACATACAT

ACATACAT

ACATACAT

ACATACAT

ACACAC

ACACAC

ACACAC

ACACAC

ACACAC

ACACAC

ACACAC

ACACAC

ACACAC

ACACAC

ACACAC

ACACAC

ACACAC

ACACAC

ACACAC

ACACAC

ACACAC

TCGCACAT

ATACACCT

GCATACCC

CCCACACAGGTG

TACACTTTGGTG

CCCACACAGGTGCCATAATA

CACACTTAAACGAAAAAAAA

CACCAAAAACACGAAAAAAA

ACACCCCT

ACACAGCT

CCACAC

CCACAC

CCACAC

CCCACATTGGAG

TCGCGACTGCTC

TTCACATTGGGG

----------------------------------------------------------------

proteasome (caca)

CACACACATAC

TGCATACATAC

CGCATACGTGT

CACACACATAT

TACATACGTAC

CGCACATATAT

TACACACATAT

TACATACATAC

TACATACATAC

TACATACATAC

TACATACATAC

AGCATACATAC

CACACACAAAR

GGCACATATAA

TACATACATAT

TACATACATAT

TACATACATAT

TACACATGAAT

TACATATATAC

TACATATATAC

TACATATGAAC

CACACATAAAT

GACATATATAC

AACACATATAC

TACATACATAA

TACATACAAGT

ACACAC

TGCACATGGGC

TGCACACAAAA

TGCACATAAAT

TGCACATAAAA

GCACAC

GCACAC

GCACAC

GCACAC

GCACAC

GCACGC

GTACACTTTCC

TGCATACAAGC

AGCATACAGAT

TCCACACAAAA

TCACAC

GTTGACACCTC

TCTTACACCCC

CGCATATGTAT

TCCCCACATAA

TACCCACATAA

CGCACAAGAAT

ACCCCACAGAT

TGCACAAAAAT

GCACAG

GCACAG

GCGCAA

GCACAA

CCACAA

TTTCGCGTTCC

----------------------------------------------------------------

mitochondrial genes (4c,3c,2c)

CCCCAT

CCCCAT

CCCCAT

CCCCAT

CCCCCT

CCCCTT

GTCCCC

GTCCCC

TTCCCC

TTCCCC

TGCCCC

CACCCC

GCCCAT

GCCCAT

GCCCAT

TCCCAT

TCCCAT

GTGCCC

TTGCCC

GTTCCC

GTTCCC

TTTCCC

TTTCCC

TTTCCC

TTTCCC

TAGCCC

TTCGCC

----------------------------------------------------------------

organellar translation machinery (4g,3g,2g)

AGGGGGAAGGG

AAGGGGCAGCG

GGAGGGGTAGC

AGGGGGTTATG

AAAGGGGAATG

AGGGGGAAATA

ATGGGGATATG

AATGGGAGGGG

ATTGGGGTGTA

AGGGGCAGCGA

GGGGTTACACA

AGGGGTAGCTA

AGGGGGAAATA

TTTACATATATTGGGGTGTACA

TGTCCTTCGAAAGGGGGAAGGG

TTTTTTTTTTTTGGAGGGGTAG

TGTGCTTATAAAGGGGCAGCGA

TTTTTTTTTTGAGGGGGTTATG

CTAAAGGGGAAAAAA

CGAAAGGGGGAAGGG

TAATAGGGGGAAATA

TTGGAGGGGTAGCTA

CAAAAGGGGAATGTT

ATAAAGGGGCAGCGA

TTTGAGGGGGTTATG

ATGTATGGGGATATG

CGAAAGGGGGAAGGGTT

TAATAGGGGGAAATACA

TTTTGGAGGGGTAGCTA

CTAAAGGGGAAAAAAAA

CAAAAGGGGAATGTTAA

AATGGGAGGGGTGTTAA

CGACTAAAGGGG

TTCGAAAGGGGG

TTTAATAGGGGG

CTTATAAAGGGG

TTTTTTGAGGGG

AAGAGGGCGTA

AGAGGGTCACA

AAGAGGGTGTG

AGAGGGATGTA

ATAGGGACCAG

AAGAGGGTGAA

AATGGGATAGG

GAAAGGGTACA

AAAGGGTTCGG

GATGGGACAAA

AGAGGGAAAAA

GAAGGGATATA

AAAAGGGAAAG

GTAAGGGTACA

AGGTGGGAATA

ATAGGGTCAAA

AGAGGGTCACA

ATAGGGACCAG

AGAGGGTGAAA

AGAGGGAAAAA

AGGGTCAAAAA

TTTTTCATATAGGGACCAGTTA

TTTTTTTTTTTGGGAGATGTTG

CACTATGGGTAGCTG

ACATATGGGTACCTT

CATAATGGGCTTATT

TGCTATGGGCAAGTT

TTATTTGGGTTTGGA

CCATGTGGGAATATT

TTTAATGGGATAGGT

GTAAAGAGGGTGTGAAA

GAAAAGAGGGTGAAATA

GACAAGAGGGTCACAAA

GAAAAGAGGGATGTATA

CGTAAGAGGGAAAAATA

GATAGGTGGGAATATAA

GCTTGAAGGGATATATA

TTCAAGAGGGCGTATAA

GAAAAAAGGGTTTTTAA

TAAATGGGAGGG

CTCTTAATAGGG

TTGCTTGAAGGG

TAAATAGAAGGG

TGGAAAAGAGGG

CTCGTAAGAGGG

TTTTTTGGAGGG

TAAATGGGAGGG

TTGATAGGTGGG

AAAGAGGG

AAAGAGGG

AAAGAGGG

TAAGAGGG

AAAAAGGG

CAAGAGGG

CAAAAGGG

CAAGAGGG

AAAAAGGG

AAAAGGACACG

AAGAGGACGTA

AGGAGGAAGAA

GGAAGGACAAA

GTAAGGTCAGG

AGAAGGAAACC

ATGTGGTCACG

AAAAGGTCGTA

GAGAGGAAATA

AGTGTGGCATG

AATAGGATGAG

AAAAGGAAACA

GTGGTCACGCG

ATGGCTAGCCA

GGAAGGACAAA

AGGATGTCACA

TGAGGTAGCGA

AGGAGGAAGAA

ATAGTGTGGCA

TTTACCAAAAAAGGTCCCGGAG

TTTTTTTTTTTTGTGGTAATGG

TTTACACACAATGGAGAAAAAA

TTTCCATATGTAGTGGATGTGA

TATCTTATGTGTGGAGTTGTGA

TTTATAAAAATTGTGGAAGAAA

TGTATTTTATATGGAGTAATAG

GTATTGAGGAAAAAAAA

CGGTTTGTGGAG

CCCATTGGAGGC

CTTAATGAGGAG

CAAGAGGA

GGAAGGACAAA

GGACGTAACCG

GGAAGGACAAA

GTAAGGTCAGG

TTTTTTTTTTTTGTGGTAATGG

TTTATATATCAATGTGTTGAGG

CAAGAAGG

----------------------------------------------------------------

organellar translation machinery (tgtg)

TGTGTGTGTGG

TGTGTAAGGGT

TGTGCATATGG

TGTGGTAATGG

TGTGGAGTTGT

TGTACGTGTGT

GGTGTGAAAGG

TGTGTTGAGGT

TGTTTGTGTGT

TGTGTTACTGT

TATGTGTATGT

TGTGTAATGGG

AGTGGATGTGA

TGTGGGAATAT

GATGTGAAGGA

TGTGTATATTT

TGTGAA

TGTGAA

TGTGAA

TGTGAA

TGTGAA

TGTGAA

TGTGAA

TGTGAA

TGTGAA

TGTGAA

TGTGAA

TGTGAA

TGTGAA

TGTGAA

TGTGAA

TGTGAA

TGTGAA

TGTGAA

TGTGAA

TGTGAA

TGTGAA

TGTGAA

TGTGAA

TGTGAA

TGTGAA

TGTGAA

TGTGAA

GGGAGGGGTGT

TGCGCAAGTGT

GGTTGGAGTGT

GATGTAAGTGT

TATGTAGGTGT

TATTGGGGTGT

GGCGTTTATGT

----------------------------------------------------------------

----------------------------------------------------------------

----------------------------------------------------------------

cytoplasmic translation machinery (4g,3g,2g; 4c,3c,2c; tgtg)

4g

GAGGGGTGATT

TAGGGGGAGGG

GTGGGGTATAT

AAGGGGTTTAA

AAGGGGGAATA

AAGGGGACTAT

AAGGGGAAAAA

AAGGGGGAAAA

ATGGGGGTGGG

AAGGGGTTAAA

GAGGGGGTTCA

AAGGGGAAAAT

AAGGGGGAAAA

TTGGGGGTGCA

GAGGGGGATGT

TAGGGGGAAAA

TTGGGGAAATG

AAGGGGGTATG

GAGGGGAAAAA

ATGGGGGAAAA

ATGGGGGCAAT

TAGGGGTTATT

AAGGGGTGATG

GTGGGGGAATG

AAGGGGAATAA

AAGGGGATATG

GTGGGGGAAAA

AAGGGGTAGAG

AAGGGGATAAA

AAGGGGGGGAA

AAGGGGACATA

ATGGGGAAAAA

TAGGGGTATAA

AAGGGGAAAAA

AAGGGGTGTTA

TAGGGGAAAAA

GAGGGGGTGTA

TTGGGGGATTT

ATGGGGAAAAT

AAGGGGGGAAA

ATGGGGAAAAA

AAGGGGATATA

ATGGGGAAATA

TAGGGGGGAAT

ATGGGGAAATA

ATGGGGAAAAT

AAGGGGAAAAA

AAGGGGTTTTT

ATGGGGTGTTA

TAGGGGGGAGG

AAGGGGGCAAA

GTGGGGGGTTT

AAGGGGAAAAA

AAGGGGAAAAG

AAGGGGGCTCT

AAGGGGTTTAA

GGGGTAT

GGGGTTT

GGGGAAT

GGGGTTC

GGGGTGC

GGGGCAT

GGGGGAT

GGGGTAT

GGGGGCC

GGGGCTT

GGGGTTT

GGGGTAT

GGGGTAT

GGGGTGT

GGGGCTT

GGGGGAT

GGGGCAT

GGGGAAT

GGGGTGT

GGGGTGT

GGGGAAT

GGGGTTC

GGGGTGT

GGGGTTT

GGGGGGT

GGGGCTC

GGGGTTT

AGGGGTTT

GGGGATAT

GGGGTTAC

GGGGGTTC

GGGGGTGC

GGGGGTAT

GGGGTTAT

GGGGATAT

GGGGGCCT

AGGGGCTT

GGGGGTGT

AGGGGTAC

CGGGGTGT

TGGGGTTC

GGGGTTTT

GGGGTTTC

GGGGGCTC

AGGGGTTT

AGGGGGAGG

TGGGGGTGG

GGGGAATTG

GGGGAGAGG

GGGGGTATG

GGGGGAATG

GGGGGCCTT

GGAGGGGGT

GGGGGATTT

AGGGGCATG

AGGGGAATT

GGGGGGAGG

GGGGGGTTT

AAGGGGAC

ATGGGGGC

ATGGGGGC

AAGGGGGG

AAGGGGAC

TAGGGGGG

TAGGGGGG

AAGGGGGC

AAGGGGGC

CACGGGGT

CATGGGGG

CATGGGGG

CAAGGGGA

CAAGGGGT

CATGGGGA

GGGGCCT

GGGGCTT

GGGGCTT

GGGGCAT

GGGGCTC

GGGGGAGGGAAAGC

GGGGGTGGGAAAAT

GAGGGGGTTCATTT

GTGGCGGGGCATAC

AAGTGGGGGAATGT

GTGTGGGGGAAAAT

GAGGGGGTGTAATT

TAGGGGGGAATTTT

GGGGGAGGACTTTT

GTGGGGGGTTTCTT

GGGGCATA

GGGGCTTC

GGGGCATG

GGGGCAAA

GAAAGAGGGG

GGGGGAGGGA

GGGGGTGGGA

GATGAAGGGG

GAAAGGGGGA

GGTGGCGGGG

GGGAGAGGGG

GGATGGGGGC

GGATATGGGG

TGTGTGGGGG

TAAAGGGGGG

TGCGTAGGGG

GGTAAAGGGG

TATAGGGGGG

TGCAATGGGG

GGGGGGAGGA

GAAAGGGGGC

TGTGGGGGGT

TAGGGGGAGGGA

GGGGTTTAAAGG

ATGGGGGTGGGA

GAGATGAAGGGG

GGGGGTGCATGC

AAGGTGGCGGGG

TTGGGGAGAGGG

GGGGGTATGAGT

GGGGTGATGAGA

GTGGGGGAATGT

AAGGGGTAGAGA

AAGGGGGGGAAA

TGTTGGAGGGGG

AGGGGCATGAGG

GGGGGGAAATAA

GGGGAAAAAAGG

GTGATTAAGGGG

GGGGTGTCATGA

ATAGGGGGGAGG

GGGGAGGGAAAGC

GGAGATGAAGGGG

AAAGGTGGCGGGG

TGGGGAGAGGGGG

GGCGCGGATGGGG

AGGGGTGATGAGA

GGGGATAAAAGGG

GTTGTTGGAGGGG

AGGGGCATGAGGC

GGGGAAAAAAGGA

GAGGGTAAAGGGG

AGTGATTAAGGGG

TAGTGGAAAGAGGGG

TAGGGTTTTGAGGGG

TGGGGAGAGGGGGAT

GCATTTTAAGGGGTG

AGGGGATATGGGGGC

GGGATAAAAGGGGCT

ATGTTGTTGGAGGGG

TAGGGGCATGAGGCT

GAGGGTAAAGGGGTT

AAGAAATGCGGGGTG

ATGATTATAGGGGGG

GTAATTAAAGGGGTT

AGTGGAAAGAGGGGTG

AGTTTTAAAAGGGGTT

TGCGTAAAAGGGGGAA

GGAGATGAAGGGGTTA

AGGGTTTTGAGGGGGT

TGGGGAGAGGGGGATG

GGCGCGGATGGGGGCA

AGCATTTTAAGGGGTG

AGGGGATATGGGGGCC

GGGGATAAAAGGGGCT

AGTGAAAAAAGGGGAC

GGGAAAACAAGGGGAA

GTTGTTGGAGGGGGTG

AGGGGCATGAGGCTGC

AGAGGGTAAAGGGGTT

AGAAATGCGGGGTGTC

AGTGATTAAGGGGATA

AGTGATGAAAGGGGGC

GGGGAAAAAATGGAAG

AGAGGTTGAAGGGGAA

TGTAATTAAAGGGGTT

TTAAAAGGGG

ATTAAGGGGG

TTATAAGGGG

TAAAAGGGGG

AAAAAAGGGG

AGAAAGGGGG

GCATAGGGGG

AAATAAGGGG

AAAGTGGGGG

ATAAAAGGGG

ATAAAGGGGG

AAAAAAGGGG

AAACAAGGGG

ATAAAAGGGG

AAAAAAGGGG

AAACAAGGGG

ATAAAAGGGG

ACATAGGGGG

TGAAAGGGGG

AAAAAAGGGG

AAAAAAGGGG

ATTAAAGGGG

AAGGGG

AAGGGG

AAGGGG

AAGGGG

AAGGGG

AAGGGG

AAGGGG

AAGGGG

AAGGGG

AAGGGG

AAGGGG

AGGGGGAGGG

GAAAGAGGGG

ATGGGGGTGG

ACAGACGGGG

AAAAAAGGGG

GGGGAGAGGG

ATAGGAGGGG

AAAGTGGGGG

ATAAAAGGGG

ATAAAGGGGG

AAAGATGGGG

AAAAAGGGGG

ATAGGGGGGA

ATGGATGGGG

ATAAAAGGGG

ATAGGGGGGA

AAAAAGGGGG

GGGGAG

GGGGAA

GGGGAA

GGGGAA

GGGGAA

GGGGAA

GGGGAA

GGGGAA

GGGGAC

GGGGAGAGGGGG

GCGGATGGGGGC

GGACAGACGGGG

GGATATGGGGGC

AAGGTGGCGGGG

GAACACACGGGG

GATGAAAGGGGG

AAAAAAGGGGGC

ATAGGGGGGAGG

AGAAATGCGGGG

GGATAAAAGGGG

GAAAAAAGGGGA

AAAAGTGGGGGA

GGGGGTGCATGC

GATTATAGGGGG

AAATACAGGGGA

ATAAACAAGGGG

AAATTAAGGGGG

ATGTATAAGGGG

GGTTTTGAGGGG

ATGGATGGGGAA

GGGGAAAAATGT

GGGGGG

GGGGGA

GGGGGA

GGGGGA

GGGGGA

GGGGTG

GGGGTG

GGGGTG

GGGGTG

GGGGGT

GGGGTA

GGGGTA

GGGGTA

GGGGTA

CGGGGA

GGGGTT

GGGGTT

GGGGTT

GGGGTT

GGGGTT

GGGGAA

GGGGAA

GGGGAA

GGGGAA

GGGGAA

GGGGAT

GGGGGG

GGGGGG

GGGGGG

GGGGGG

GGGGGG

AGGGGG

AGGGGG

AGGGGG

AGGGGG

AGGGGG

AGGGGG

AGGGGG

AGGGGG

AGGGGG

AGGGGG

AGGGGG

GGGGGC

GGGGGC

GAGGGG

GAGGGG

GCGGGG

GCGGGG

TGGGGG

TGGGGG

TGGGGG

TGGGGG

TGGGGG

TGGGGG

AAGGGG

AAGGGG

AAGGGG

AAGGGG

AAGGGG

AAGGGG

AAGGGG

AAGGGG

AGGGGC

AAGGGG

AAGGGG

AAGGGG

AGGGGC

AAGGGG

AAGGGG

AAGGGG

AAGGGG

AAGGGG

AAGGGG

AAGGGG

AAGGGG

3g

TGGGTTTC

AAGGGCAC

AAGGGCTC

AGGGATTC

AGGGTCAC

AGGGTCAC

GAGGGTTC

GGAGGGAGG

AAGGGAAC

ATGGGAGC

ATGGGAAC

ATGGGAAC

AAGGGAAG

ATGGGAGC

ATGGGAAC

TGGGCTC

AGGGCAC

AGGGCAC

AGGGCTC

TGGGCCC

TGGGCGT

TGGGCAT

TGGGCAT

AGGGCCC

TGGGCTC

AGGGCTT

TGGGCTT

GAGGGAGGGAAAAT

ATGGGAGGCTATGT

ATGGGAGGAACATT

ATGGGTGGTATTAT

GGAGGGAGGG

TGATGTGGGA

GATGGGAGGC

TGTAGAGGGT

TGTTATGGGC

TGAAAAGGGC

TGAAGAGGGA

AAGGAGGGAGGG

AGGGTTTTGAGG

AGGGAGAAGGGA

TAGGGCACGAGA

TGGGTGAAGAGA

AAGGGCTTAAAG

AAGGGAAAAAGG

AGAGGGTAAAGG

AAGGGTCTGGGC

ATTGAGGATGGGA

AAGGAGGGAGGGA

AAGGCTGTAGGGT

ATTGTTGAAGGGC

GGGTATGAAGGGA

AGGGCTTAAAGTG

AGGGATCAAAGTG

AGGGAAAAAGGGA

GTGAGATGGGAGGCT

GTGATCAAAAGGGAG

AGTGTATAAAGGGAAT

GTCGAAAGGAGGGAGG

AGTGAGATGGGAGGCT

AGAGCAGAAAGGGTTT

GGTGATCAAAAGGGAG

TGGGTATGAAGGGAAG

AGGGCTTAAAGTGTAT

AGGGATCAAAGTGAAG

AGTGTTTTAAGGGATT

AGGGATAAAAGAGGTT

AGTGACATAAGGGTCA

AAGAAGGGAG

AAAATGGGAG

AAAAAGGGAG

ATGGGAGCGT

AAAAAAGGGA

GAGGGA

GAGGGA

GAGGGA

GAGGGA

GAGGGC

GAGGGTAAAGGG

GAGATGGGAGGC

AGGGAAAAAGGG

GTAGATGGGAGC

AAAAATGGGTGG

ACAAATGGGAGC

AAAAACGGGAGA

ATAGAAGGGAGA

ATAAATGAGGGC

AGTAACGGGTGA

AAACAAAGGGAA

CGGGTG

CGGGTA

CGGGTA

CGGGTT

GGGATTT

GGGAGAAGG

GGGACATC

GGGTCTTG

GGGACACG

GGGACATA

GGGACATA

GGGTCACG

GGGTCACA

GGGACTTA

GGGACATA

GGGAGAAGGG

GGGAGGCTATGT

GGGTATGAAGGGA

GGGATAAAAGAGGTT

GGGAGCCT

GGGAGCCT

GGGCACGA

GGGAGCGT

GGGAACAC

GGGAACAC

GGGAACCA

GGGAGACC

GGGAGGCT

GGGCACAA

GGGAACCT

GGGAGGAA

GGGAAGGA

GGGAAGGA

GGGAACAA

GGGAACAA

GGGAACAA

GGGAAAGC

GGGAACAT

GGGAACAT

GGGAACAT

GGGAAGAA

GGGAAGAA

GGGAAGAA

GGGAGAAG

GGGAGAAG

GGGAAAAC

GGGAAAAC

GGGAGAAA

GGGTATGAAGGG

GGTGGCGGG

CATGTGGG

TGAGGATGGG

GGAGGGAGGG

GGCTGTAGGG

GGGAGAAGGG

TGTTGAAGGG

TATGGTTGGG

GGAAAAAGGG

TGTTGGAGGG

TGTAGATGGG

TATGGATGGG

GGAAAAAGGG

GGTTGAAGGG

AAGGAGGGAGGG

GTGATGAAAGGG

GAGGTTGAAGGG

AGTGGAAAGAGGG

AGAGCAGAAAGGG

AGTGTTTTAAGGG

AGTGAACAACGGG

AGTGACATAAGGG

AGTGATGAAAGGG

AGAGGTTGAAGGG

AAAGGG

AAAGGG

AAAGGG

AAAGGG

AAAGGG

AAAGGG

AAAGGG

AAAGGG

AAAGGG

AAAGGG

AAAGGG

AAAGGG

AAAGGG

AAAGGG

AAAGGG

AAAGGG

AAAGGG

AAAGGG

AAAGGG

AAAGGG

AAAGGG

AAAGGG

AAAGGG

AAAGGG

AAAGGG

AAAGGG

AAAGGG

AAAGGG

AAAGGG

AAAGGG

AAAAGTAGGG

ATAAGAAGGG

GAAAGGAGGG

AAAGAAAGGG

ATAAGTAGGG

GTGGAAAGAGGG

GGAGATGAAGGG

GAGGGTAAAGGG

GAGCAGAAAGGG

GGGTATGAAGGG

AGGGAAAAAGGG

GAAAAAAAAGGG

AAAGAAAAAGGG

GTGAACAACGGG

AAACATGAAGGG

ATATGAAGAGGG

AAAAAAAAAGGG

GTGTATAAAGGG

GTTGTTGGAGGG

GAAATAAAAGGG

ATATATAGAGGG

AAGTTCAAAGGG

GGAGGG

2g

AGTGGATGG

GGTGGACGA

AGTGGTGTG

ATGGCAAC

ATGGCAAC

ATGGCAAC

CACGTGGT

CACGTGGT

CATGTGGA

CATGTGGT

ATGTGAGGATTTTT

TCGTGTGGTATAAT

TGGACACG

TGGACACG

GAAGGAAGGA

GGAGACTGGC

TGTGGAAGGA

GGAAAAAGGC

GAAAGAAGGC

GTGGTCTAGTGG

AGGCGCGGATGG

GTGGTGAAAAGG

AGGTTGCCGAGG

GGTGATATAGGTG

AGTGGTGAAAAGG

AGTGATGAGAGGA

TTGTCATGTGAGGAT

AAGAGAGAAGAGGCT

AGGTGATAAGTGGAT

GAGTTATAAGTGGTG

GAGCCTTAAGTGGTG

AAGGTTGCCGAGGTG

TTAATATTTGAGGTG

GTGATCTAAGAGGTT

GGAGAAGAAAGTGGTT

AGAGAGAAGAGGCTCT

AGTGATTAAAGAGGTT

GGTGATATAGGTGTAC

AGAGTTATAAGTGGTG

AGAGCCTTAAGTGGTG

AAAAAAGGAG

AAATAAGGAG

ATAATAGGAG

AAAAAAGGAA

TCAAAAGGAG

AAAAAAGGAG

ATAATAGGAG

AAATAAGGAG

AAAGAAGGAG

AAAAAAGGAG

ACAAAAGGAG

AAAAAAGGAG

AAACAAGGAG

TTAAAAGGAG

ACAAAAGGAG

AAAGGCCCGG

AAGGTGGCGG

AAAGGCGCGG

AAAGAGGCGT

GAGGCG

GAGGCA

GAGGCA

GAGGCA

GAGGCA

GAGGCA

GAGGAG

GAGGAG

GAGGAG

GAGGAG

GAGGAA

GAGGAA

GAGGAA

GAGGAA

GAGGAA

GAGGAA

GAGGAA

GAGGAA

GAGGAA

GAGGAA

GAGGAA

GAGGAA

GAGGAA

GAGGAA

GAGGAA

GAGGAA

GAGGAA

GAGGAA

GAGGAC

GAGGAC

AGGCGCGG

AGGAACCC

AGGAGCCA

GGAAGGAC

AGGAACGG

AGGAGCGT

AGGAAGGC

AGGCACGA

AGGAACCA

AGGAACCA

GCAAAAAGGTGG

AAGAAAGAAGGC

AAAAAAGGAGGA

AAAGAAAGAGGA

GAAGAAAAAGGA

AAAGGAAAAGGC

GAGAAGGAAGGT

GAAGAGGCGGAA

ACAAATGGAAGG

AGGAAAAGGAGA

AAATAAAGAGGC

AAAAAAAGGTGG

GAAAGGAACGGA

AAAAAAGGATGG

GAAATTGGAGGA

GCATGAGGCTGC

AAAAAAAGAGGA

GGAGTTGGCAGC

GTGATGAGAGGA

ACAAAGAGGAGA

AAAAATAGGTGG

GTATGAAAAGGC

AAAGAAAAAGGA

GAAGTGGCAAGA

CAACACGGAAGG

GAATAAAAAGGA

GAATAAAAAGGA

AAAAAAAGGAGA

AAAAAAGGAAGA

ATAAAAAGAGGA

ATGTGAAAAGGC

GAAAAAGGAAGT

AAAAGTGGAGAG

GAAAAAACAGGT

AAACGGAAGAGA

ACAAAAAAAGGA

AAAAAAAAAGGA

AAAAAAAAAGGA

GAAAAAGGAAAG

AAAAAAAAAGGA

AAAAAGAAAGGT

ATAGGCAAAGAG

ATATAGGAAGAG

ACTAATAAGGAG

AATCGGAAATGA

GGAGAAC

GGAGCAT

GGAGCGT

GGAGCCT

GGTGGACGA

GGAGACTGGC

GGAAAAAGGC

GGAGAAGAAAGTG

GGTGATCAAAAGG

GGTGATATAGGTG

GGAGAAGAAAGTGGTT

GGTGATATAGGTGTAC

GGAGATTAAAGTGTAC

GGACGCAC

GGACACGC

GGAAACCC

GGAAGCAG

GGAAGGAC

GGACACCA

GGCCACAG

GGCAGCAA

GGAAACAC

GGAAGCAA

GGACAGAC

GGACAGCA

GGATAAAAGAGG

GGATAAAGAAGC

GGAGTTGGCAGC

GGTGATAAGTGG

AGTGGATGG

AGAGAGAAGAGG

GTGGTCTAGTGG

AGGCGCGGATGG

GTGGTGAAAAGG

AGGTTGCCGAGG

GGTGATCAAAAGG

AGTGGTGAAAAGG

AAAGGCCCGG

AAGGTGGCGG

AAAGGCGCGG

ATAGAAGAGG

AGGCGCGG

AGGAACGG

GCAAAAAGGTGG

GAAAAAAGAAGG

GGATAAAAGAGG

AAAGAAAGAAGG

GAAAAAGAAAGG

ACAAATGGAAGG

AAAAAAAGGTGG

AAAAAAGGATGG

AAGTAAAGAAGG

AAAAATAGGTGG

GAAAGTAAAAGG

CAACACGGAAGG

GGTGATAAGTGG

ACAAACACAAGG

AAATAAACGTGG

GTGAAAAAATGG

GAATATAAAAGG

AAAAAAAAAAGG

AAAAATAAGTGG

ATATAAAAAAGG

ACTCAAAAAAGG

4c

CCCCCC

CCCCCC

CCCCCC

CCCCCC

CCCCCC

CCCCCC

GCCCCC

GCCCCC

GCCCCC

GCCCCC

GCCCCC

CCCCCT

CCCCCT

CCCCCT

CCCCCT

CCCCCT

CCCCCT

CCCCCT

CCCCCT

CCCCCT

CCCCCT

CCCCCT

CCCCTC

CCCCTC

CCCCTC

CCCCTC

CCCCTC

CCCCTC

CCCCTC

CCCCTC

CCCCTC

CCCCAC

CCCCAC

CCCCAC

CCCCAC

CCCCAC

CCCCAC

GCCCCT

GCCCCT

GCCCCT

CCCCCA

CCCCCA

CCCCCA

CCCCTT

CCCCTT

CCCCTT

CCCCTT

CCCCTT

CCCCCTGGAGC

CCCCTCCACCC

CCCCCTACGTC

CCCCTTGTACC

CCCCCCCAAGT

CCCCTTTGAGC

CCCCACATCCC

CCCCTTATAGC

CCCCTCTTTCC

CCCCCTTTATC

CCCCCCCCAAA

CCCCTTTCATC

CCCCTCAACTC

CCCCCTATAGT

CCCCCCCTTTA

CCCCACGCACA

CCCCCCATAAG

CCCCCTTGTGT

CCCCTTATGGG

CCCCATGGAGT

CCCCCTTTTGT

CCCCCCTTTTT

CCCCTTAAAGG

CCCCTTTTTTC

CCCCTTATAGT

ACCCCTATAGC

CCCCCTTTATT

CCCCACAGTGA

CCCCTTCTTCA

CCCCTTACAAG

CCCCCTATATA

CCCCATTGCAC

CCCCATCCATA

CCCCCTTTTTT

CCCCACAACAC

CCCCTTATTAC

CCCCTCTTTTT

CCCCTTTTTTG

TCCCCCATACC

CCCCACTTTGA

CCCCTCTTTTT

CCCCTTGGCAA

CCCCTCAAATT

CCCCTTTAAAC

CCCCATTTTTG

CCCCTTATTTT

CCCCTTTTATA

CCCCTTTTTTT

CCCCTTTAACA

CCCCTTTAATA

GCCCCTATATA

CCCCATTTTAT

CCCCATTATTA

CCCCATAAAAT

CCCCATAATAA

GACCCC

GACCCC

GACCCC

CACCCC

CACCCC

GTCCCC

GTCCCC

GTCCCC

CACCCC

AACCCC

AACCCC

AACCCC

AACCCC

AACCCC

AACCCC

GTTCCCCT

TTTTCCCC

TTTTCCCC

TTTTCCCC

CTTCCCCT

CCCCATCCATAC

CCCCTTCTTCAA

AACCCC

AACCCC

AACCCC

AACCCC

AACCCC

AACCCC

AACCCC

AACCCC

AACCCC

AACCCC

AACCCC

AACCCC

AACCCC

AACCCC

CCCCCT

CCCCCT

CCCCCT

CCCCCT

CCCCCT

CCCCCT

CCCCCT

CCCCCT

CCCCCT

CCCCCT

CCCCCT

CCCCCT

CCCCCT

CCCCCT

CCCCCT

CCCCCT

CCCCCT

CCCCATATTAATAAAAAAA

CCCCCTATTATTAGAAATA

CCCCAAAAAAAAAAAAAAA

CCCCCTTTATCACCACATA

CCCCTCTATAATATAAATA

CCCCCTGGAGCTCTATAAA

CCCCCAAAAAAAAAAAAAA

GCCCCTATATATATATATA

CCCCTTTAAACAAAAAAAA

CCCAACCCCATAAAATATA

CCCCTTTTTTCACTACATA

CCCCCCCAAAAAAAAAATA

CCCCATGGAGTGATAAAAA

CCCCATAATAAAAAAAAAA

CCCCCATAAGTTATATATA

CCCCACATATAAAAAAAAA

CCCCAAATAATACAAAAAA

AATAAACCCCTC

AAAAAAAACCCC

AATAAAAGCCCC

AAGAAACCCCCC

CCCCAAAAAAAA

CCCCAAAAAAAA

CCCCATAAAATA

CCCCAAAAAAAA

3c

GCCCAC

GCCCAC

CCGCCCTAAGG

CCCTTCCCAGG

ACCCACGCATC

ACCCTTGTACC

ACCCTTAGATC

ACCCATTGACC

GCCCTTACACA

ACCCTTATGTC

ACCCTTATTTC

ACCCTCTCTTT

GCCCACTTATT

ACCCTTCTTCA

ACCCTTGAACT

GCCCATATACA

ACCCATATAAC

GCCCTTATTTA

TCCCTTACCTT

TCCCGTATAAC

ACCCTTAAACA

ACCCATTTATT

ACCCATTTTTT

ACCTCCCAATT

ACCCATCAAAT

TCCCTTATTTT

ACCCGTAAAAA

TCCCTTTTAAA

GCCCTTCC

GGCTCCCT

GACCTCCCTCAC

GCACACCCAACC

ACCCACGATCAT

GCCCTTACACAT

ACCCATATCCAT

ACCCATCATCAT

ACCCACCTCTTT

ACCCACGCATCT

ACCCTTATTCAC

ACCCTTCTTCAA

ACATACACCCAT

GCCCATTTGTATATACAAA

GCCCAATAAAATAAATATA

GCCCAAAAAAAAAAAAAAA

GCCCACTTATTAAAATAAA

GCCCAAAATGAAAAAAAGA

GCCCTAAGGTAAAAAAACA

GCCCAAGGAAATAAAAAAA

AAAAAACCCTAC

AAGAAAACCCGC

AAAAAACCCGTC

CCCGGCGTGGG

CCCTTCCCAGG

CCCTCTGCCTG

CCCGTTGGTTT

CCCTTTTAATC

CCCTACTTATT

CCCTTCATAAT

CCCTTTTTATT

CCCTTTTTTTT

CCCTATATATA

CCCTTTATTAT

CCCTTCCC

CCCAGCAAACTC

CCCATATATAAAAAAAATA

CCCTTTTTTTTAAAAAAAA

CCCAATACATAAAAATATA

CCCAAAAAAAAAAAAAAAA

CCCAATATTTTACAAAATA

CCCAACATTTCTCAAAATA

CCCAGTTCCAAAATAAAAA

CCCATCCATACAATACATA

CCCAAAAAAAAAAAAAAAA

CCCAAAAAAAAAAAAAAAA

CCCATATTTTATAAATAAA

CCCTCTTCAACAATATATA

CCCTGAACTAAAAAAAAAA

CCCAACATATATACATACA

CCCAAATAATATATATATA

CCCTAGATAATAACAAAAA

CCCATAAAAATAAAATATA

CCCAATAACTTTAAATATA

CCCTATTTATTTAAAGAAA

CCCAATATAAAGAAAAAAA

CCCATTATATATATATATA

CCCTATTATATTCTATATA

CCCAAAATTAAAAAATAAA

CCCTCAACTCTACCACAGA

CCCTTAAATATTATATATA

CCCTTTGAGCGTAGATAGA

CCCATCATCATTAAAAAAA

CCCAATAAAAAA

CCCTTTAAAAGA

CCCAAAAAAAAA

CCCAATAAAATA

CCCAAAAAAAAA

CCCAAAAAAAAA

CCCTTTAAAAAA

CCCAAAAAAAAA

CCCAAAAAAATA

CCCAAAAAAAAA

CCCATAAAAATA

CCCACCAAAAAA

CCCTATAAAAAA

GGGCCC

GAGCCC

AGGCCC

AGGCCC

AGGCCC

GAGCCC

AGGCCC

GTGCCC

AAGCCC

AAGCCC

AAGCCC

AAGCCC

AAGCCC

AAGCCC

AAGCCC

AAGCCC

AAGCCC

AAGCCC

AAGCCC

AAGCCC

CCGCCC

CTGCCC

CTGCCC

ACGCCC

ATGCCC

ATGCCC

ATGCCC

ATGCCC

ATGCCC

ATGCCC

ATGCCC

TGGCCC

TGGCCC

GGTCCC

GGACCC

GGTCTCCC

GTTCTCCC

CCCTTCCC

GCCGTCCC

TCTCTCCC

GTGTTCCC

CTCTTCCC

GCTTTCCC

GGTTTCCC

TGCTTCCC

TTTCTCCC

TTCTTCCC

TTCTTCCC

GTTTTCCC

TTCTTCCC

TTCTTCCC

CTTTTCCC

TTGTTCCC

CTTTTCCC

TTGTTCCC

TTGTTCCC

TCTTTCCC

GCACATGTTCCC

AAACCC

AAGCCC

AAACCC

AAACCC

AAGCCC

AAGCCC

AAACCC

AAACCC

AAACCC

AAGCCC

AAACCC

AAACCC

AAACCC

AAACCC

AAACCC

AAACCC

AAACCC

AAGCCC

AAACCC

AAACCC

AAGCCC

AAACCC

AAGCCC

AAACCC

AAGCCC

AAGCCC

AAGCCC

AAGCCC

AAACCC

AAACCC

AAGCCC

AAACCC

AAGCCC

AAACCC

AAACCC

AAACCC

AAACCC

AAACCC

AAACCC

AAACCC

AAACCC

AAGCCC

AAGCCC

AAAAAAAAGCCC

2c

CCACCTCAGTC

CCAGCCACAGG

ACCTTCACACC

ACGCCTATATG

CCACCTTTTTT

ACGCCTTTTGT

TCCGTTCAAGC

ACGCCTCTTTA

GCCTGTCCATT

ACCTCTCCTTT

CCAGCCTCCAT

ACCGTTGAATG

GCACGTCCTTT

ACCGTTACATA

ACCTTCAGTCA

GCCTTCTTTTT

ACCTACAACAC

TCCTTTTTATC

GCCTTTATGAA

GTCCTTCC

GGGCTCCT

CTCCTCCT

TTCCTTCC

TTCCTTCC

TTCCTTCC

TTCCTTCC

TTCCTTCC

GCTCTCCT

TTCCTCCT

TTCCTCCT

CCTCTCCT

GCCTACCTCCAC

ACCAGCCTCCAT

GCCAGTCTCCAT

CTCCGTCCTCAT

GCAAGCCACCAA

CTCCATCTCCTC

CCACTACACCAC

GCCAGCCACAGG

CAACACCATCAC

ACCTACATACAC

ATACACCTACAC

GCCTCTAAATTCATAAAAA

GCCTTTTTATATATATATA

GCCTAATATGTTATAAAAA

GCCATATAATATATATATA

GCCTATATGATTATATATA

GCCATTAGATATATATATA

GCCATAAGAAAAAAAAAAA

GCCAGTCTCCATAAAAACA

GCCAAATAAATAATATAAA

GCCTTCTTCATAATATATA

GCCATATATAAAAGAAAAA

GCCTGTATACAAAAAAAAA

GCCATATTAAAAAAAAAAA

GCCATTAAAAACAAACAAA

GCCTACCTCCACATATAAA

GCCTTAAAAGTAAAAAATA

GCCTATATATTTATAAAAA

GCCTAAAAAATAAGATATA

GCCTCGTAATATAAATATA

AATAAAACCTTC

AAGAAACGCCTC

AATAAAAGCCAC

CCGCTTGAATC

CCACCTCAGTC

CCACGTGTATC

CCAGCCACAGG

CCGCATGAAAC

CCGCTTTTATT

CCACGTATTCG

CCACCTTTTTT

CCACTTAACAC

CCGGTCATATT

CCATTTCGAGG

CCGGTCATATA

CCACTTCAATA

CCGCATGAAAA

CCAGCCTCCAT

CCACTTATTTT

CCACATAAGGA

CCGTTTCTTTT

CCACATTGAAA

CCACTTAATTA

CCTCTCCT

CCACTACACCAC

CCACGTATTCGT

ACCTTCACACC

GTCCTTCC

GGGCTTCC

GTGCTTCC

GTGCTTCC

GTTCTTCC

GTTCTTCC

TTCCTTCC

TTCCTTCC

TTCCTTCC

GTTCTTCC

TTCCTTCC

TTCCTTCC

CGTCTTCC

TCTCTTCC

tgtg

ACATGTGCAC

ATATGTGCTC

ATATGTGCAC

ATATGTGCTC

ATATGTGCAC

ATATGTGGTC

ACATGTGCAT

TCATGTGGAC

ATATGTGCCT

ATATGTGCAT

ATATGTGCAT

ATATGTGCAT

ATATGTGCTT

TTGTGTGGCC

TTATGTGCTT

TTATGTGCTT

TTATGTGCAT

TTATGTGCTT

ATATGTGGTT

ATATGTGGTT

TCATGTGGGT

TATGTGCTCCG

TGTGACCCCAC

TGTGTGGCCCA

TGTGACCTCCC

TATGTGCTTCC

TATGTGCATCG

TATGTGCACAT

TTTGTGCTTCC

TATGTGCCTCA

CATGTGGACAG

TGTGTTCCCAT

TATGTGCTCTT

TATGTGCATAC

TATGTGCATAC

TATGTGTCCCT

TATGTGACCAC

TGTGTGCGTGT

CATGTGCATAT

TGTGTGTACCG

TATGTGCATAT

TGTGTTCCTTG

TGTGTTCCTTT

TGTGAGGATGT

CATGTGATCAT

TGTGTCATTTT

TTAGTGCCCAT

TAAGTGCACTC

TAAGTGCACAT

TAAGTGCTCTT

GGAGTGCATAT

TTAGTGCCTAT

TTTGGTGCTGC

TTAGTGCTTGT

TGAGGTGTCCA

GATATGCGTAG

GTAATGCATGT

TGTATGCATAA

----------------------------------------------------------------

dna replication (tgtg)

CACATGTGTGTA

TACATGTGTGTG

CTCAGGTGTGTG

TATATGTGTGTG

TATATGTGTGTA

TATATGTGTGTA

TTTATGTGTGTA

TTTATGTGTGTA

TATTTGTGTGTA

CACTTATGTGTA

TGTATGTGTGTA

TCCTTATGTGTA

TTCATATGTGTA

GCCATATGTGTA

TTCATTTGTGTA

TATATATGTGTA

TATATATGTGTA

TATTTATGTGTA

TATATATGTGCA

TTTTGGTGTGGG

CTTTTGTGTGTT

TCTTTTTGTGTA

TATATATGTGTT

TACTGTTGTGTT

TTTTTTTGTGTA

TATATATGTGCT

CATTAATGTGTA

GGTGTG

GGTGTG

GGTGTG

GGTGTG

TGTGGG

TGTGGG

TGTGTG

TGTGTG

TGTGTG

TGTGTG

TGTGTG

TGTGTG

TGTGTG

TGTGTG

TGTGTG

TGTGTG

TGTGTG

TGTGTG

TGTGTG

TGTGTG

TGTGTG

TGTGTG

TGTGTG

TGTGTG

TGTGTG

TGTGTG

TGTGTG

TGTGTG

TGTGTG

TGTGTG

AGTGTG

AGTGTG

AGTGTG

AGTGTG

TGGTGTGGGGAATT

ATGTGTGTGAAAAA

ATGTGAGTGTGAAA

ATGTGTGAGAAAAA

TGGTGTGTGAAAAA

ATGTGTGTGTAATT

ATGTGTGTGTTAAT

AGGTGTGTGATTAA

TGGTGTGAGTTTAA

GGTGTGGGGAATTTTTATT

TGTGTGGTTAAGTATTATT

TGTGTGTAACTTTTTTTTT

TGTGTGGTGTGAGTTTAAT

TGTGGGTTTGTTTTGTCAT

TGTGTGTATTCGTTTTAAT

TTTGTGATATTTCATTTTT

TGTGTGTAGTTTTTTTTTT

TGTGTGGATACTTTTTCAT

TATGTGTATATTACATTTT

TGTGTGTGTTAATTATTTT

TGTGTGTTTTTCTTCTTCT

GGTGTGTGAAAAATATTAT

TGTGTGTTACATATATATT

TTGGTGTGGG

CCTGTGTGGT

TATTTGTGTG

CATGTGAGTG

CAGGTGTGTG

TATGTGTGTG

CATGTGTGTG

AATGTGTGTG

CTCAGGGGTGTA

TCCTTTCGTGTA

TATATACGTGTA

dna replication (4g,3g,2g,1g)

GAGAGGGGGAA

GGGGGAAGCAA

AAAAGGGGAAA

GAGGGGGAAAAA

AAGGGGGAAAAA

AAGGGGAAAATA

GGGGAAGCAAAGA

GGGGGAAAAAATA

GGGGTGATAAAAA

GGGGGAAAAAAAA

GGGGTGTAAATAA

TTTTTGGGGGGGGTT

TTTTTAAGGGGGAAA

TTATTGAGAGGGGGA

TTTTTTTTGGTGTGGGG

TTTTTTTTTTTGGGGGG

CTTATTATTGAGAGGGG

TATTTTTTTTAAGGGGG

TTTTTGTTCAAATGGGG

TATATCTTTCCTTGGGG

ATTATTTCACATTGGGG

GAGAAAGGGAG

AAGGGAATCAA

AAGGGAGAGAGA

AAGGGAGTGATA

AAGAGGGATATA

AAAATGAGAAGGGCT

AAAAAAAGAGGGATA

ATTTTGAGAAAGGGA

AAAGGGAGAGAGAGA

GGGAGAGAGAG

GGGAGAG

GGGAGAG

GGGAGTG

GAGAGGG

AGAAGGG

AGAAGGG

GAGAGAGGAAA

AAGAAGAGGAG

AGGAGAAGAAA

GGAAAAAGGAG

AAGAAAAGGAA

GAGAGGAAAAA

AGGAGAGAAAA

AAAAGAAGGAA

AGAAAAGGCAG

AGAAAAAGGAA

AGGAAAGAAAA

AGGAGAG

AGGAGAG

AGGAGAG

GAGAGAGGAAAA

AAAGGAGATAAA

AAAGGAGAAATA

AAAGGAGAAATA

AAAGGAGAAAAA

GAGAGGAAAAAA

GAAGGATTTATA

GAAGGAAAAAAA

GAAGGAAATAAA

GAAGGAAAAAAA

GAAGGAACTAGA

AAGAGGATAAAA

AGAGTGAGAGAGGAA

AAAAAAAGAAGGAAA

AATAAAAGAAGGAAA

AAAATGTGAAGGAAA

GGAAAAAGGAG

GGAAGAG

GGAGAG

GGAGAG

GGAGAG

GGAGAG

GGAGAG

GGAGAG

GGAGAGAAAAAAA

GGAGAGTGAGAGA

GGAGAGTTAATAA

GGAGAAAAAATAA

GGAGTGATACAGA

GGAGAGAAATTCA

GGAGAAAAAAAAA

GGAGTAATACAAA

GGAGAAATATTAA

GGAGAAAAAAAAA

GGAGAAGAAAAAA

GGAGAAATATACA

GGAGAGAGAGAAA

TTTTTTTTTGTGTGTGG

TATATAATCCTGTGTGG

TATATACACATGAGAGG

GAGAGAACGAA

GAGAGAGCCAA

GAGAGAAAAAG

AAGAAGACGAG

AAGAGAAAGAA

GAGAAAAAGAA

AAGAAGAGAAA

AAGAAAAGAAA

AAGAAAAGAAA

AAAAGAAGAAA

GAGAGAG

GAGAGAG

AGAAGAG

GAGAGAGCCAAA

GAGAGAGATATA

GAGAGAACGAAA

GAGAGAAAAAGA

GAGAGAAAAAAA

GAGAGAAAAATA

AGAATGAGAGAGATA

TTAAAGAGAAAGAAA

GAGAGAACGAA

GAGAGAGCCAA

GAGAGAAAAAG

GAGAAAAAGAA

GAAAAAAGAAG

GAGAGAG

GAGAGAG

GAGAGAGCCAAA

GAGAGAGATATA

GAGAGAACGAAA

GAGAGAAAAAGA

GAGAGAAAAAAA

GAGAGAAAAATA

GAGAGAAAAAG

AAGAAGACGAG

GAAAAAAGAAG

AAAAGAAAAAG

GAGAGAG

GAGAGAG

AGAAGAG

GAGAGAAAAAG

GAAAAAAGAAG

GAAAAAAAAAG

GAGAGAG

GAGAGAG

AAGAAAAAAAA

----------------------------------------------------------------

proteasome - tgtg

GTGTGCAT

GTGTGTAT

GTGTGTAT

GTGTGTAT

ATGTGCAT

ATGTGCAT

ATGTGCAT

ATGTGTAT

ATGTGTAT

ATGTGTAT

ATGTGTAT

ATGTGTAT

ATGTGTAT

ATGTGTAT

ATGTGTAT

ATGTGTAT

ATGTGTAT

ATGTGAAT

TGTGTATGTA

TGTGTATGTA

TGTGTATGTA

TGTGTATGTA

TATGTATGTA

TATGTATGTA

TATGTATGTA

TATGTATGGA

TATGTATGTA

TTTGTATGTA

TATGTATGTA

TATGTATGTA

TATGTATGTA

TATGTATGTA

TATGTATGTA

TATGTATGTA

TATGTATGTA

GTATGTAT

GTATGTAT

GTATGTAT

GTATGTAT

Proteasome – g-rich – 4g, 3g, 2g

GAGGGGAG

ATGGGGAG

TTGGGGTG

GGGGGAAA

AGGGGGCA

TTGGGGTA

ACAAAGGGGGAAATA

GGGGAGTTGAGGAAAAA

GGGGGAAATAATAACAA

GGGGAG

GGGGAG

GGGGGC

GGGGTG

GGGGGA

GAGGGGAG

CAGGGGGC

ATGGGGAG

AAAGGGGG

TAGGGGTC

TTGGGGTG

GAGGGAAG

GAGGGAAG

GAGGGAGG

GTGGGAAA

GAGGGAAA

AAGGGAAG

AAGGGATG

AAGGGAAG

ATGGGATG

TCGGGAAG

ATATTGAGGGAAGAA

AAAAAGAGGGAAGGA

AAAAAGAGGGAAAAA

AGTAAGAGGGAATAA

ATTATGTGGGAAAAA

AAATAATGGGAAAAA

ACATAATGGGAAATA

ATAAGAAGGGAAGAA

ATATAAAAGGGATGA

ATAATAAAGGGAAGA

ATTAAAAGGGAACGA

CGGGAT

CGGGTC

GAGGGAGG

GAGGGAAG

GAGGGAAG

AAGGGAAG

AAGGGATG

TCGGGAAG

GACGGGTC

AAAGGGAG

GAGGGTTG

AAGGGAAC

ATGGGATG

AATGGGAG

GGGAATTGTATTAAAAA

GGGATTTTGAAGAACAA

GGGAATAAAAAAAACAA

GGGAAAATGATATAAAA

GGGAAAAAAAAAAAAAA

GGGAAGAAAAAATAAAA

GGGTTAAAAAAAAGAAA

GGGAACGAAAAAAAAAA

GGGTGGCTAAATTACAA

GGGAAAAAAAAAAAAAA

GGGAGG

GGGAAG

GGGAAG

GGGAAG

GGGATG

GGGATG

GGGAGA

GGGAGA

GGGAAT

GGGAAT

GGGAAT

GGGAAA

GGGCAT

GGGCAA

AAAATGCAGGAAGTA

ATATGGTAGGAATGA

AAAATGAAGGAAATA

ATTAAGAAGGAATTA

AAATAAAAGGAAAAA

AAAATAAAGGAAACA

AAAGAAAAGGAAAAA

CGGAAG

CGGAAG

CGGAGT

CGGAAA

GCAGGAAG

ACCGGAAG

GAAGGATG

ACAGGAAG

AAAGGAAG

GGTGAC

----------------------------------------------------------------

mitochondrial genes – G-rich - 4g,3g,2g,1g

ATGGGGA

AAGGGGA

AAGGGGA

CTCAAAGGGG

GTAAAGGGGA

ATGGGCA

TTGGGCA

GTGAAAGGGC

CAAAACGGGA

GTAAAAAGGG

AGGCGCA

ATGCGGA

AAGCGGA

AAGCGGA

GTGAACGGAG

GTGAATGGCG

CTAAAGGCGC

GCAAAAGGCA

GCAAATAGGC

GAAAAAGGAA

GTGAAAGCGG

ATGCGCA

TAGCGCA

GTAATAAGCG

GTAATAAGCG

GTAATAAGCG

mitochondrial genes – tgtg

TGGGTGTGTGG

TGTGTATGCGC

TGTATGTGTGT

TGTGTGTATGT

TGGATGTGTGA

CGTGTGTGTAT

TGTATGTGTAC

TGTAAGTGTGT

TGTGTATACGA

AGTGTGTATAT

TATTAGTGTGTATA

TGGATGTGTGAAGA

TGGGTGTGTGGTAA

TGTAAGTGTGTATA

TGTATGTGTGTTGA

ATGTGAAGGAA

GTGTGAAGAAA

GTGTGTTGAAA

TGGGTGTGTGG

GAGGTGTGCCA

TGTGAACGGAG

GATGTGTGAAG

CGTGCGGATGG

TATCAGTGCGCATA

GAGTGTTGGAC

GGCGCATGAAG

AGTGCATGAAA

TGTATGTATAC

----------------------------------------------------------------

organellar translation machinery (4c,3c,2c)

TCCCCC

TCCCCC

TCCCCC

TCCCCC

GCCCCT

TCCCCT

TCCCCT

TCCCCT

TCCCCT

ACCCCT

ACCCCT

ACCCCT

TCCCCA

TCCCCA

TCCCCA

TCCCCC

TCCCCC

TCCCCC

TCCCCC

GCCCCT

TCCCCT

TCCCCT

TCCCCT

TCCCCT

TCCCCA

TCCCCA

TCCCCA

TCCCCA

GACCCC

CCCCTGGTGC

CCCCTTGACC

GAGTTCCCCA

GCCCAT

TCCCAT

TCCCAT

TCCCAT

TCCCAT

TCCCAT

TCCCAT

GGCCCT

TCCCGT

TCCCAA

ACCCAA

TACCCT

GGCCCT

TGCCCA

GAGTTACCCA

CCCTTGTCGA

GCTCCC

TCTCCC

GGTCCC

TCACCT

ACACCT

ACACCT

ACACCT

GCTCCT

TGCCAT

GCCTCC

TCCTCC

TCCTCC

TCCTCC

TCCTCC

TCCATGTGGG

TGCCTGTTGG

TCCATTTTGC

TCCATTTTGG

CCAATGGTGC

CCATTGGAGG

GCCTCC

TCCTCC

TCCTCC

TCCTCC

TCCTCC

GGCTCC

TGCTCC

TGCTCC

TGCTCC

----------------------------------------------------------------
