# Supplementary material for: Identification of putative regulatory motifs in the upstream regions of co-expressed functional groups of genes in Plasmodium falciparum
Source: BMC Genomics. 2009 Jan 13;10:18. doi: 10.1186/1471-2164-10-18 (PMC2662883; doi:10.1186/1471-2164-10-18)
Supplement: Additional file 6 — The list of putative regulatory motifs occurring in the upstream regions of the 13 functional groups of genes. The sequence and upstream position of each putative regulatory motif is given. [file 1471-2164-10-18-S6.doc]

Additional file 6. Putative regulatory motifs, occurring in the upstream sequences of each of the 13 sets of functionally related and co-expressed genes, are listed.

This table provides information that complements the feature maps discussed in the study. For each motif occurrence marked in a feature map, this table gives the gene ID, the position of the motif with respect to the TLS, and the sequence of the motif. To make this list, all motif positions in each feature map (obtained as output from the DNA pattern program) have been pooled and arranged in ascending order. As a result, in each feature map, motifs occurring farthest away from the TLS have have been listed at the top, while motifs occurring closest to the TLS have been listed at the bottom. This list makes it easy to spot positional conservation: motifs that are positionally conserved occur one after the other, in a bunch, in the list. Sets of positionally conserved motifs have been highlighted in color in the table. Each highlighted set has been statistically validated in Additional file 8. Information is first provided for the feature maps in Figures 5, 6, 7, 8, 9, 10 and 11 [‘first set’, in Additional file 8] and then for other feature maps discussed in the text [‘second set’, in Additional file 8].

Sets of motifs marked by ‘~’ are positionally conserved motifs that are interesting but are statistically weakly significant (p>0.05). Pmot and Pseq values [see Additional file 8 for definitions] for these sets of motifs are given.

Putative regulatory motifs listed in this table can be used to experimentally probe transcriptional regulation of gene co-expression in *Pf*.

transcription machinery - g-rich - 4g+3g PF11_0445; -1998 TGTGGGG

PFE0465c; -1968 TGTGGGG

PFC0155c; -1605 AATGGGAAAAA

PFB0715w; -1489 GGGAAAAAACAAAA

PFF1390w; -1382 GGTGGGAAAAA

PF11_0445; -1374 GGGTGAAAATAAAA

PFE0465c; -1366 AGAGGGGAAAA

PFC0155c; -1322 GCGGGTCTACAAGAAAAATGAA

PF07_0027; -1055 TGTGGGG

PF11_0445; -856 AAGGGGAAAAAAAAAAATAAAA

PFB0290c; -825 TTGGGGAGTTA

PF11_0264; -618 GGAGGGGGGGA

PFC0155c; -542 GGGTAAAAAAAAAA

PF13_0023; -420 GTGGAGGGGGA

PF13_0150; -349 TAGGGAGT

PF07_0027; -222 AGAGGGC

PF14_0207; -27 GGTTGGGAAAA

----------------------------------------------------------------

ribonucleotide synthesis - g-rich - 4g+3g+2g+1g

PF10_0123; -1760 CAAGTGCC

PF13_0287; -1748 TAAAGGCG

PF13_0044; -1736 ATAAGGGGATATCAAAAA

PF14_0100; -1733 TAAAGGGA

PF13_0287; -1692 CGTGGTGG

PF13_0287; -1664 TAAAGGGA

PF10_0225; -1595 CCAAGGGA

PF13_0044; -1586 ATATGGAAATTAAAGAGA

MAL13P1.221; -1397 ACAAGGGGAAAAAGGAAT

PF13_0287; -1370 CCAAGGAGATAT

PF10_0121; -1354 CGAAGGAA

PF10_0289; -1316 CGAACACA

PF14_0697; -1167 CATAGGCA

PF10_0121; -1095 TGAGGTCG

MAL13P1.221; -1045 GGTAGGCC

PF10_0225; -974 CGAAGACA

PFE0660c; -877 CGGTGGCA

PF10_0121; -835 ATAAGGGCATATTTAAAA

PF10_0225; -827 GTAGGAAAATATAA

PFI1420w; -824 TGGGGGGA

PF10_0123; -815 ATAAGGGCACAATAGAAA

PF13_0044; -805 AAATGGGGATTTTTAAAA

PF14_0697; -761 TACGCGCA

PF10_0086; -759 CAGGTGCC

PF10_0225; -645 ATAAGGGGAATTTATATT

PF14_0100; -601 AAATGGAGAAAAATAAAA

PF14_0100; ~ -474 TAAAGGGA Pmot<0.22, Pseq<0.19

PF10_0086; ~ -463 AATCGGGGAGGATAAAAA

PF13_0287; ~ -443 AAATGGAGAATAATAAAA

PFB0295w; ~ -405 GAAAGAGG

PFE0630c; ~ -400 TGGAGAGG

PFF0160c; ~ -347 GGTGGTCA

PFF0160c; -339 AAAAGGAGAAAAAAAAAA

PFF0160c; -158 GAAGGGCC

PFE0630c; -119 TGAAGGCA

----------------------------------------------------------------

dna replication - caca

MAL13P1.22; -1857 ACACACAC

PF10_0165; -1823 ACACACAT

MAL13P1.22; -1807 CCCACATTGGAGTAATACAA

PF10_0362; -1743 ACATACAT

PFE0155w; -1710 ACACACCC

PFD0590c; -1630 TACACTTTGGTG

PFL0150w; -1612 ACACACAT

PF11_0117; -1425 ACATACAT

PFD0790c; -1280 TACACACC

PFL1285c; -1266 ACACACCT

PFC0340w; -1230 CCACACAT

MAL7P1.21; -1210 ACATACAT

PFL0580w; -1104 ACACCCCT

PF13_0251; ~ -1037 ACACACAT Pmot<0.15, Pseq<0.13

PFD0590c; ~ -1031 ACATACAT

MAL7P1.21; ~ -1028 ACACACAC

PF13_0328; ~ -1027 TCCCACAT

PF14_0601; ~ -999 GCACACAC

PFC0340w; -947 CCACACAT

PFL2005w; -933 ACACAC

PFL1655c; -866 ACACACAT

PFE0155w; -859 TTCACATTGGGG

PFA0545c; -807 ACATACAT

PFC0340w; -749 CCCACACAGGTGCCATAATA

MAL7P1.21; -741 ACACAC

PFB0840w; -688 GCATACCC

PFI0530c; -583 CACCCCTTTTTTACACATAA

PF10_0165; -572 ACACAC

PFI0235w; -550 ACATACAT

PFL0150w; ~ -503 ACATACAC Pmot<0.10, Pseq<0.07

PFL0580w; ~ -503 CCACAC

PFA0545c; ~ -496 TCGCGACTGCTC

PFE1345c; ~ -489 ACACACAT

PF10_0362; ~ -481 ACATACAC

PFB0840w; -420 ACACAC

PFL0150w; -418 ACATACAT

PFC0340w; -363 CCACACAT

PF14_0254; -350 CACCAAAAACACGAAAAAAA

PF14_0177; -333 ATACACCT

PF07_0023; -332 ACACACAT

PFF1225c; -328 ACACACAT

PF14_0602; -298 ACACAGCT

PF14_0254; -294 CCACACAT

PFL1120c; -280 ACATACAT

PFB0895c; -264 TACACACC

PFL0150w; -168 ACACACAT

PFB0840w; -133 ACATACAT

PFI0530c; -118 TCACACAT

PFL2005w; -111 ACATACAT

PFB0840w; -109 ACATACAT

PFB0840w; -97 ACATACAT

PFB0840w; -85 ACATACAT

PF14_0254; -65 ACATACAT

PF13_0291; -34 TCGCACAT

----------------------------------------------------------------

proteasome - caca

PFF0420c; -1992 GTACACTTTCC

PFI0630w; -1946 GCACAG

PF10_0081; -1938 CCACAA

PF13_0156; -1930 GCACAA

PF13_0033; -1871 CGCACATATAT

PF14_0632; -1725 TGCACAAAAAT

PF11_0314; -1713 TGCATACAAGC

PF14_0716; -1697 ACACAC

PF14_0632; -1682 TCACAC

PF14_0676; -1657 ACACAC

MAL13P1.270; -1655 TCACAC

PFF0420c; -1649 TCACAC

PF14_0025; -1626 TGCACATAAAT

PFB0260w; -1619 TACATACATAT

PF13_0033; -1606 GACATATATAC

PFI0630w; -1523 CCACAA

PFF0420c; -1505 AGCATACAGAT

PFF0420c; -1396 ACACAC

PFA0400c; -1395 GCGCAA

PFI1545c; -1326 TCCCCACATAA

PFD0665c; -1307 CCACAA

PF14_0716; -1272 TTTCGCGTTCC

PF14_0676; -1267 TACATACAAGT

PF10_0081; -1084 TACATACATAT

PFD0665c; -1046 TACACACATAT

PF14_0676; -1021 TACATATGAAC

PFB0260w; -977 TGCACATGGGC

PF13_0063; -947 AGCATACATAC

PF13_0282; -938 TCTTACACCCC

MAL13P1.190; -911 TACATACATAC

PF14_0632; -905 TCACAC

MAL13P1.190; -903 TACATACATAT

PFI0630w; -884 GCACAC

PFA0400c; -881 TACATACATAC

PF10_0081; -855 GGCACATATAA

PF14_0716; -854 CGCACAAGAAT

PF10_0298; -845 TCACAC

PF10_0174; -824 TCCACACAAAA

PF14_0632; -823 TACATATATAC

PFC0745c; -823 TACATACATAC

PF14_0676; -816 ACACAC

PFC0745c; -815 TACATATATAC

PFF0420c; -738 GCACAC

PF10_0174; -735 GCACAC

PF11_0314; -725 TACATACATAT

PFI0630w; -722 TACATACATAT

MAL8P1.128; -671 TACACATGAAT

PFB0260w; -660 ACCCCACAGAT

PFC0745c; -652 TACATACATAT

MAL8P1.128; -641 TACATACATAT

PF14_0716; -641 GCACAG

MAL8P1.142; -586 TACATATATAC

PFF0420c; -560 TACATACATAA

PF07_0112; -483 TACCCACATAA

PFF0420c; -450 CGCATACGTGT

PFC0520w; -395 GCACAG

PF07_0112; -382 TACATACGTAC

PF07_0112; -295 AACACATATAC

PFD0665c; -253 GCACGC

MAL13P1.190; -232 GTTGACACCTC

PF10_0174; -199 CGCATATGTAT

MAL8P1.142; -190 TGCACATAAAA

PF14_0025; -165 TGCACACAAAA

PF14_0676; -87 TGCATACATAC

PF13_0033; -78 GCACAC

PF13_0033; -67 TACATACATAC

PF14_0632; -31 TACATACATAT

----------------------------------------------------------------

mitochondrial genes - c-rich - 4c+3c+2c

PFE0970w; -1905 GTCCCC

PFE0970w; -1453 TTTCCC

PFE0970w; -1445 TTTCCC

PFE0970w; -1432 TTTCCC

PF14_0373; -1233 GCCCAT

PF13_0327; -1070 TGCCCC

PFL1725w; -1037 TTGCCC

PFL1725w; -991 TTTCCC

PF13_0061; -985 TTTCCC

PFL1725w; -734 TTTCCC

PF14_0597; -630 TTCGCC

PFE0225w; -565 ACGCAT

PFE0970w; -544 TTTCCC

PF13_0061; -527 GTGCCC

PF13_0353; -515 TAGCCC

PF13_0359; -515 GTTCCC

PF13_0327; -497 CACCCC

PF14_0597; -466 TTTCCC

PF13_0061; -406 GTTCCC

PF10_0120; ~ -98 CCCCAT Pmot<0.08, Pseq<0.06

PF14_0721; ~ -94 GTCCCC

PF14_0597; ~ -90 TTTCCC

MAL13P1.47; ~ -52 TTTCCC

----------------------------------------------------------------

organellar translation machinery - g-rich - 4g+3g+2g

PFI0375w; -1900 TTATTTGGGTTTGGA

PFI0375w; -1649 CCATGTGGGAATATT

PFL1895w; -1456 GTATTGAGGAAAAAAAA

PFL1590c; -1365 AAAAGGACACG

PFL1590c; -1297 CGACTAAAGGGG

PF11_0414; -1173 TTTTTCATATAGGGACCAGTTA

PFB0645c; -1149 ATGTGGTCACG

PF14_0166; -1142 TTTAATGGGATAGGT

PF14_0658; -1137 CAAAAGGGGAATGTTAA

PFI0890c; -1044 AAAAAGGG

PFB0585w; -1032 AAAAGGAAACA

PF14_0132; -866 TTTTTTTTTTTTGGAGGGGTAG

PFL1895w; -788 GAAAAAAGGGTTTTTAA

PF07_0062; -785 GTAAAGAGGGTGTGAAA

MAL13P1.281; -781 TTTCCATATGTAGTGGATGTGA

PFE0960w; -723 TTGCTTGAAGGG

PF14_0642; -688 ATAGTGTGGCA

PFI0890c; -675 AGGATGTCACA

PF11_0181; -631 ATGGCTAGCCA

PF11_0414; -614 TTTTTTTTTTTGGGAGATGTTG

PFL1895w; -608 GGGGTTACACA

PF14_0212; -592 TTTTTTTTTTGAGGGGGTTATG

PF14_0166; -583 TATCTTATGTGTGGAGTTGTGA

PFI1575c; -571 TGGAAAAGAGGG

PFB0645c; -534 TTTACCAAAAAAGGTCCCGGAG

PFD0600c; -501 TTTTTTTTTTTTGTGGTAATGG

PF14_0289; -490 GAAAGGGTACA

PF14_0166; -481 TTTAATAGGGGG

PF14_0289; -474 GTAAGGGTACA

PFB0585w; -433 CTCTTAATAGGG

PFF0115c; -425 AATAGGATGAG

PF07_0062; -405 TAAATAGAAGGG

PF11_0181; -405 TTCAAGAGGGCGTATAA

PF14_0132; -388 CCCATTGGAGGC

PFI1575c; -383 TTTATATATCAATGTGTTGAGG

PFD0600c; -365 TTTACACACAATGGAGAAAAAA

PFI1240c; -331 TGAGGTAGCGA

PF08_0014; -298 CTCGTAAGAGGG

PFL1150c; -290 GATGGGACAAA

MAL13P1.281; -284 AGGAGGAAGAA

PFL1540c; -250 GTAAGGTCAGG

PFL1540c; -235 TGTCCTTCGAAAGGGGGAAGGG

PFD0600c; -218 CATAATGGGCTTATT

PFL1895w; -209 TGTATTTTATATGGAGTAATAG

PF14_0132; -200 TAAATGGGAGGG

PF14_0212; -198 GACAAGAGGGTCACAAA

PFI1240c; -189 AAAAGGTCGTA

PF14_0132; -176 TTTATAAAAATTGTGGAAGAAA

PF14_0289; -157 TGTGCTTATAAAGGGGCAGCGA

PF14_0166; -126 CAAGAAGG

PF07_0062; -84 GAAAAGAGGGATGTATA

PF14_0132; -83 TTGATAGGTGGG

PF10_0332; -67 GGAAGGACAAA

PFL1895w; -57 ATGTATGGGGATATG

PFB0390w; -45 CACTATGGGTAGCTG

PF14_0606; -42 CTTAATGAGGAG

PFL1590c; -42 TTTACATATATTGGGGTGTACA

PFL1540c; -34 CAAGAGGA

PFB0645c; -30 ACATATGGGTACCTT

MAL13P1.281; -27 GAGAGGAAATA

PFI1240c; -27 AAAAGGGAAAG

PFE0960w; -19 CGGTTTGTGGAG

PF08_0014; -17 TGCTATGGGCAAGTT

organellar translation machinery - tgtg

PF14_0289; -1681 TGTGAA

PFI0375w; -1646 TGTGGGAATAT

PFB0585w; -1081 TGTGCATATGG

PFL0770w; -890 TGTGAA

PF11_0414; -796 TGTGAA

PFL0770w; -795 TGTGTATATTT

PFE0960w; -778 TGTGAA

PF07_0062; -777 GGTGTGAAAGG

MAL13P1.281; -770 AGTGGATGTGA

PFB0585w; -765 TGTGAA

PF14_0166; -743 TGTGAA

PF14_0289; -643 TGTGAA

PF08_0014; -634 TGTGAA

PFI1240c; -584 TATGTGTATGT

MAL13P1.164; -581 TGTGAA

PF14_0132; -581 TGTGAA

PFI0375w; -576 TGTGAA

PF14_0166; -574 TGTGGAGTTGT

PF14_0212; -572 TGTGAA

PF11_0414; -501 TATGTAGGTGT

PFD0600c; -490 TGTGGTAATGG

PFL1150c; -485 TGTGAA

PF14_0289; -477 TGTGTAAGGGT

PF10_0332; ~ -437 TGTGAA Pmot<0.29, Pseq<0.25

MAL13P1.164;~ -436 TGTGTAATGGG

PFL1150c; ~ -402 TGTGTTACTGT

MAL13P1.281;~ -397 TGTGAA

PFI1575c; ~ -371 TGTGTTGAGGT

PFB0390w; ~ -363 TGTGAA

PF11_0386; -336 GATGTAAGTGT

PF14_0212; -269 TGTGAA

PF14_0166; -263 TGTGAA

PF14_0606; -259 TGTGAA

PF14_0606; -252 TGTGAA

PF14_0132; -195 GGGAGGGGTGT

PF14_0606; -170 TGTTTGTGTGT

PFE0960w; -140 TGTGAA

PF10_0332; -137 GGCGTTTATGT

PF14_0212; ~ -109 TGTGTGTGTGG Pmot<0.08, Pseq<0.13

PF14_0642; ~ -94 TGTGAA

PFE0960w; ~ -84 TGTACGTGTGT

PF14_0642; ~ -54 TGCGCAAGTGT

PF14_0270; ~ -42 TGTGAA

PFL1590c; ~ -34 TATTGGGGTGT

PF11_0181; ~ -32 GGTTGGAGTGT

PFF0115c; ~ -18 GATGTGAAGGA

PF11_0386; ~ -6 TGTGAA

----------------------------------------------------------------

----------------------------------------------------------------

----------------------------------------------------------------

cytoplasmic translation machinery - 4g,3g; 4c,3c; tgtg

4g

PF07_0088; -1983 TGCAATGGGG

PF10_0043; -1963 TGTGTGGGGG

PF14_0401; -1935 AAAAAAGGGG

PF13_0268; -1927 ATGGGGAAAAT

PF14_0231; -1822 ATAAAAGGGG

PF11_0106; -1814 GGGGTTTT

PFI0645w; -1709 ATAGGAGGGG

PF14_0296; -1617 CATGGGGA

PFE0810c; -1591 TTGGGGAAATG

PFC0735w; -1567 GAACACACGGGG

PF14_0198; -1538 ATGGGGAAATA

PFC0400w; -1508 TTATAAGGGG

PFE1085w; -1483 TTGGGGGTGCA

PF13_0228; -1467 ATGTTGTTGGAGGGG

MAL7P1.113; -1456 GGCGCGGATGGGGGCA

PF08_0096; -1443 AAATAAGGGG

PF14_0141; -1430 ATGGGGAAAAA

PF13_0268; -1425 TGCGTAGGGG

PFC0735w; -1415 CATGGGGG

PFC0735w; -1399 TGCGTAAAAGGGGGAA

PFE1405c; -1374 AAAGGTGGCGGGG

PF14_0141; -1365 AGAGGGTAAAGGGGTT

PF11_0065; -1309 AAGGGGTAGAGA

PF11_0043; -1275 ATGGGGAAAAA

PFE0810c; -1235 TTGGGGAGAGGG

PF08_0076; -1213 AGTGATGAAAGGGGGC

PF08_0039; -1192 AAGGGGAAAAG

PF11_0051; -1181 AGGGGTTT

MAL13P1.209; -1161 ATAAAAGGGG

PF10_0043; -1146 AAGGGGATATG

PFL0210c; -1137 GGGAAAACAAGGGGAA

MAL13P1.92; -1135 GGGGTAT

PF10_0043; -1135 GGGGCCT

PF07_0088; -1132 ATGTATAAGGGG

PFF0885w; -1120 AAAAAAGGGG

PF07_0080; -1111 TAGGGGGAGGGA

PF11_0043; -1031 TAGGGGTATAA

PF14_0627; -1031 AGTGATTAAGGGGATA

PF10_0187; -991 AGCATTTTAAGGGGTG

PF07_0080; -971 TAGTGGAAAGAGGGG

PFB0885w; -970 AAATTAAGGGGG

PFE1085w; -967 AGAAAGGGGG

PFB0830w; -957 AGTTTTAAAAGGGGTT

PF08_0039; -950 AAAAAAGGGGGC

PF11_0313; -950 ATAAAGGGGG

PF08_0039; -946 AAGGGGGCTCT

PFD1055w; -918 TAGGGTTTTGAGGGG

PF10_0077; -898 AAGGGGAATAA

PF14_0296; -887 ATGATTATAGGGGGG

PF14_0563; -878 AAATACAGGGGA

PFC0775w; -852 GGAGATGAAGGGGTTA

PF14_0240; -848 TGGGGTTC

PF08_0076; -814 TGTGGGGGGT

PFL2055w; -801 TGTAATTAAAGGGGTT

PF14_0627; -793 AAGAAATGCGGGGTG

PFE0845c; -791 ACATAGGGGG

MAL13P1.209; -790 TAGGGGAAAAA

PF10_0149; -790 AAAAGTGGGGGA

PFE0845c; -789 ATAGGGGGGAGG

MAL13P1.209; -788 GGGGAA

PF10_0149; -786 GTGGGGGAATGT

PF14_0627; -786 GCGGGG

PF10_0038; -771 TAGGGGTTATT

PF11_0312; -757 AGTGAAAAAAGGGGAC

PFE0810c; -747 GCATAGGGGG

PFD0565c; -724 GGACAGACGGGG

MAL7P1.113; -675 CATGGGGG

PF14_0589; -615 ATAAACAAGGGG

PFC0735w; -558 AAGGGGAAAAA

MAL13P1.14; -548 TTGGGGGATTT

PFC0295c; -542 CGGGGA

PF11_0260; -485 CAAGGGGA

PF11_0260; -474 AAGGGG

PF14_0083; -392 ATGGATGGGGAA

PFB0455w; -348 GTGGGGTATAT

MAL13P1.144; -282 AAAGATGGGG

PFE0185c; -122 AAAAAAGGGG

PF14_0589; -73 GGGGTGT

3g

PF14_0401; -1938 AAAAAAAAAGGG

PFL0625c; -1859 GGGACATA

PF11_0312; -1824 ATGGGAAC

PF14_0231; -1819 AAAGGG

PF13_0129; -1814 GAGGGA

PF14_0231; -1801 GGGAAGAA

PF10_0043; -1797 ATGGGAAC

PFE0350c; -1787 GAGGGA

PF13_0268; -1729 GGGAAGGA

PFI0645w; -1706 GGAGGG

PF07_0080; -1689 TGGGCTT

PFL0210c; -1646 TGGGTGAAGAGA

PFC0300c; -1625 GTCGAAAGGAGGGAGG

PFB0445c; -1616 TGGGTTTC

PFL0210c; -1592 GGGAAAAC

MAL13P1.209; -1585 ATATGAAGAGGG

PFL0675c; -1534 TGGGCTC

PFF0885w; -1491 GGGACATA

PF13_0170; -1479 AGTGACATAAGGGTCA

PF13_0228; -1465 GTTGTTGGAGGG

PF11_0065; -1457 AAACATGAAGGG

PF10_0043; -1451 TGTAGAGGGT

PF14_0027; -1440 AAAGGG

PFB0830w; -1438 ACAAATGGGAGC

PFE1405c; -1399 AAAATGGGAG

PFC0735w; -1393 AAAGGG

PFL0675c; -1393 GGGATTT

PF10_0043; -1392 ATTGTTGAAGGGC

PF13_0257; -1388 CGGGTA

PFE1405c; -1371 GGTGGCGGG

PF14_0141; -1365 AGAGGGTAAAGG

PF13_0045; -1348 GAAATAAAAGGG

PFL0210c; -1344 CATGTGGG

PFC0295c; -1342 AGTGTATAAAGGGAAT

PFL2055w; -1342 AAAGGG

PFL0210c; -1340 TGGGTATGAAGGGAAG

PF14_0296; -1338 GGGACTTA

PFC0295c; -1335 AAAGGG

PF11_0313; -1327 TGTTATGGGC

PF14_0027; -1316 AAAGGG

PF07_0043; -1313 GGAAAAAGGG

PF14_0401; -1301 AAAGGG

PF13_0213; -1296 ATAAGTAGGG

MAL13P1.92; -1283 AAAAATGGGTGG

PFC0295c; -1251 AAGGGAAC

MAL13P1.209; -1237 AGTGTTTTAAGGGATT

PFL2010c; -1229 ATGGGAAC

PF08_0076; -1213 AGTGATGAAAGGG

PF08_0039; -1211 CGGGTG

PF08_0039; -1200 AGAGGTTGAAGGG

PF14_0027; -1183 AAAGAAAAAGGG

PFB0445c; -1168 GGGACATC

MAL13P1.209; -1158 AAAGGG

PFB0860c; -1153 GGGAACCT

PF10_0043; -1147 AAAGGG

PFL0210c; -1137 GGGAAAAC

PF11_0438; -1132 ATAGAAGGGAGA

PFF0885w; -1117 AAAGGG

PFD0565c; -1109 CGGGTT

PF07_0080; -1106 GGAGGG

PF11_0065; -1093 TGGGCCC

PF07_0080; -1089 AAAAGTAGGG

PFD0770c; -1068 AGTGAGATGGGAGGCT

MAL7P1.113; -1062 AAAGGG

PF10_0264; -1057 AAAGGG

PF08_0075; -1051 GGGAGACC

PF10_0264; -1049 TGGGCTT

PF08_0075; -1017 AAAAAAGGGA

PF08_0042; -1003 ATGGGAAC

PF13_0049; -1001 AGGGATCAAAGTGAAG

MAL7P1.113; -1000 TAGGGCACGAGA

PF10_0038; -995 AAAGGG

PF07_0080; -985 AAAGGG

PF07_0080; -970 AGTGGAAAGAGGG

PFE1085w; -969 AAAGAAAGGG

PF08_0039; -953 AAAAAAAAAGGG

PFB0830w; -950 AAAGGG

PF11_0313; -948 AAAGGG

PFA0145c; -944 AGTAACGGGTGA

PF11_0312; -939 TGGGCAT

PFC0290w; -939 ATTGAGGATGGGA

PFD1055w; -925 AAGGCTGTAGGGT

MAL13P1.92; -924 TGAAAAGGGC

PFB0830w; -918 GAGGGA

PFE1005w; -898 GGTGATCAAAAGGGAG

PF13_0129; -876 AGGGATAAAAGAGGTT

PFC0775w; -852 GGAGATGAAGGG

PF10_0209; -830 AAAAAAAAAGGG

PF07_0079; -817 AAAGGG

PFB0455w; -798 AAAGGG

PFL2475w; -795 ATATATAGAGGG

PF11_0272; -794 AGGGCCC

PFL2055w; -794 AAAGGG

PF11_0312; -750 AAAGGG

PF10_0187; -733 ATAAATGAGGGC

PF13_0171; -681 GGGAGCCT

PFB0455w; -681 ATAAGAAGGG

PFL2475w; -648 TATGGTTGGG

PF13_0262; -635 TGTAGATGGG

PFE0350c; -628 GGGAAGAA

PFC0775w; -610 TGGGCTC

PF13_0354; -607 AGGGTCAC

PF08_0075; -604 AAAGGG

PFD0455w; -594 AAGTTCAAAGGG

PFC0300c; -588 GGGTCTTG

PF14_0584; -579 CGGGTA

PF13_0014; -557 AAAGGG

PFC0300c; -556 TGATGTGGGA

MAL13P1.14; -544 GGGATTT

PFD0245c; -535 AGAGCAGAAAGGGTTT

PF14_0627; -512 ATGGGAGC

PF13_0177; -479 AGTGAACAACGGG

PF13_0170; -477 AAACAAAGGGAA

PFB0455w; -476 AAAGGG

PF11_0260; -475 AAAGGG

PF13_0177; -470 CGGGTT

PF10_0043; -468 GGGATTT

PF14_0083; -393 TATGGATGGG

PF14_0104; -365 AAAAACGGGAGA

MAL7P1.113; -357 GGGAACAT

PF07_0071; -313 CGGGTT

PF11_0106; -257 GGGAAGGA

PFD0245c; -244 AAAGGG

PF07_0080; -237 AAAGGG

PFE0185c; -236 AAAGGG

PFE0715w; -234 AAAAAAGGGA

PF07_0043; -218 GAAAAAAAAGGG

PF13_0049; -217 AAAAAAAAAGGG

PF13_0049; -203 AAAGGG

PF11_0313; -137 AAAGGG

PFE0185c; -125 AAAAAAAAAGGG

PF14_0141; -113 GAGGGTTC

PFD1055w; -107 AAAAAAGGGA

PF11_0065; -43 TGGGCGT

4c

PFE0885w; -1960 TTTTCCCC

PFC0735w; -1792 TTTTCCCC

PF13_0228; -1763 AACCCC

PFC0735w; -1759 ACCCCTATAGC

PFB0455w; -1695 TTTTCCCC

PF14_0655; -1630 AACCCC

PF10_0038; -1607 CCCCTTTAACA

PF11_0245; -1541 CCCCTC

PF13_0224; -1535 TTTTCCCC

MAL7P1.81; -1531 TTTTCCCC

PFB0455w; -1524 CACCCC

PF13_0045; -1508 CACCCC

PF14_0486; -1502 CCCCTTTGAGC

PF10_0187; -1479 CCCCCTTTATT

PF08_0075; -1432 AAGAAACCCCCC

PF14_0486; -1427 CCCCTC

PFI0645w; -1404 GCCCCTATATATATATATA

PF11_0447; -1379 TTTTCCCC

PFL2010c; -1359 CCCCAAATAATACAAAAAA

PF07_0043; -1350 AACCCC

PF14_0083; -1333 AACCCC

PF10_0077; -1323 CCCCATTGCAC

PFC0535w; -1295 AAAAAAAACCCC

PF14_0486; -1258 CTTCCCCT

PFC1020c; -1190 CCCCCT

PF14_0428; -1177 CCCCTTCTTCAA

PF13_0224; -1166 CACCCC

PF07_0079; -1136 CACCCC

PF11_0313; -1122 GTCCCC

PF10_0264; -1064 CCCCCT

PF08_0075; -1034 GCCCCC

PF11_0245; -1004 TTTTCCCC

PFE0810c; -1003 CCCCATCCATAC

PF07_0079; -986 CCCCCT

PFC0295c; -948 CCCCATTTTTG

PF07_0043; -942 TTTTCCCC

PFE1005w; -942 GCCCCT

PF11_0272; -939 CCCCTT

PFE0185c; -938 TTTTCCCC

PF11_0312; -925 CCCCCCCTTTA

PFC1020c; -916 CCCCCTTTATCACCACATA

PFE0185c; -912 AACCCC

PFC0400w; -909 CCCCACAGTGA

PFF0885w; -890 CCCCCT

PF14_0428; -875 AACCCC

PFC0300c; -835 CCCCCTACGTC

PF13_0132; -817 AACCCC

PF13_0045; -815 GCCCCC

PFC0200w; -806 CCCCACTTTGA

PF11_0272; -791 GCCCCC

PF14_0240; -780 AACCCC

PF10_0272; -771 CCCAACCCCATAAAATATA

PF14_0391; -769 CCCCCCATAAG

PFD0565c; -707 AACCCC

PF14_0579; -703 CCCCTCAACTC

PFD0770c; -702 CCCCACATCCC

PF14_0655; -654 CCCCTTACAAG

PFC1020c; -638 CCCCAAAAAAAAAAAAAAA

PFC0400w; -621 CCCCATATTAATAAAAAAA

PFC0775w; -621 TTTTCCCC

PFL0210c; -574 TTTTCCCC

PF13_0132; -558 GCCCCC

PF13_0213; -544 CCCCATTATTA

PFC0775w; -536 AATAAAAGCCCC

PF14_0198; -527 GTTCCCCT

PF14_0240; -469 TTTTCCCC

PFL0380c; -465 TTTTCCCC

PF10_0187; -450 AACCCC

PFE0810c; -442 CCCCCAAAAAAAAAAAAAA

PFL0310c; -424 GTCCCC

PF11_0043; -354 CCCCTCTTTTT

PFB0445c; -276 AATAAACCCCTC

PFE1005w; -255 CCCCCCTTTTT

PF11_0447; -236 GACCCC

PF13_0178; -134 GACCCC

PF08_0096; -119 GACCCC

PF08_0096; -90 CCCCACGCACA

PF11_0438; -63 GCCCCT

PF08_0042; -60 GTCCCC

PFL0310c; -54 CTTCCCCT

PFE1085w; -42 CCCCATTTTAT

PFD1070w; -29 GCCCCC

PF14_0141; -21 TTTTCCCC

PF08_0076; -19 TTTTCCCC

3c

PF14_0240; -1987 TTCTTCCC

PFD1055w; -1967 CCCTTTATTAT

PF13_0316; -1948 CCCAGCAAACTC

PF14_0240; -1941 CCCACCAAAAAA

PF14_0391; -1884 CTTTTCCC

PF11_0272; -1875 CCCAAATAATATATATATA

PF13_0268; -1875 TCCCGTATAAC

PF07_0079; -1860 TCCCTTTTAAA

MAL13P1.243; -1839 GCCCTTACACAT

PF13_0214; -1829 AAGCCC

PF14_0141; -1794 ACATACACCCAT

PFB0445c; -1749 AAACCC

PFI0645w; -1746 CCCTTTTTTTT

PF11_0312; -1732 CCCTTTAAAAAA

PF14_0083; -1729 TTCTTCCC

MAL13P1.243; -1711 TTGTTCCC

PF13_0171; -1708 GCACATGTTCCC

PFF1095w; -1676 GAGCCC

PFB0455w; -1659 ACCCACGATCAT

PF11_0065; -1637 CCCTCTGCCTG

PF14_0585; -1636 CCCAAAATTAAAAAATAAA

PF11_0438; -1618 GCACACCCAACC

PFB0445c; -1611 TTCTTCCC

PFF0345w; -1598 TCTCTCCC

PF14_0240; -1594 TGGCCC

PF07_0043; -1553 ACGCCC

PF11_0245; -1540 CCCTCTTCAACAATATATA

PF14_0486; -1501 CCCTTTGAGCGTAGATAGA

PF13_0170; -1492 GGCTCCCT

PF08_0075; -1429 AAACCC

PFI0645w; -1406 ATGCCC

PF13_0129; -1398 AAACCC

PFE1085w; -1391 CTGCCC

PF14_0104; -1380 GAGCCC

PF11_0051; -1370 TGCTTCCC

PFB0455w; -1368 CCCTTTTTTTTAAAAAAAA

PFB0830w; -1345 CCCTTTAAAAGA

PF14_0563; -1340 ACCTCCCAATT

PF10_0077; -1326 ATGCCC

PFC0535w; -1290 AAACCC

PF10_0077; -1288 AAAAAACCCGTC

PF07_0080; -1279 CCCTTTTTATT

PFC0400w; -1278 AAACCC

PF13_0316; -1238 ACCCTTAGATC

PF13_0228; -1237 AAGCCC

PFC0535w; -1237 AAACCC

PF08_0076; -1228 CTTTTCCC

PF10_0272; -1214 CCCTTTTTTTT

PF08_0076; -1188 GGTCTCCC

PFC0535w; -1178 CCCAAAAAAAAAAAAAAAA

PFE0350c; -1172 AAACCC

PF14_0141; -1153 GTTCTCCC

PF13_0268; -1143 TGGCCC

PFI0645w; -1126 AAGCCC

PFL2010c; -1125 TCCCTTACCTT

PF08_0096; -1119 AAACCC

PFD0565c; -1114 AGGCCC

PF14_0240; -1111 CCCTATAAAAAA

PFD0565c; -1098 CCCGGCGTGGG

PF11_0065; -1092 GGGCCC

PF10_0149; -1089 TTTCTCCC

PF07_0043; -1086 GCCGTCCC

PF14_0627; -1056 GGTTTCCC

PF14_0198; -1032 CCCTTAAATATTATATATA

PF07_0079; -1017 AAAAAACCCTAC

PF13_0049; -1016 ACCCGTAAAAA

PFE0810c; -1002 CCCATCCATACAATACATA

PF07_0079; -995 GCTTTCCC

MAL7P1.113; -988 CCCGTTGGTTT

PF14_0198; -982 AGGCCC

PF13_0268; -977 AAACCC

PFC0735w; -971 ACCCTTATTCAC

PFE1005w; -944 ATGCCC

PF11_0313; -931 ATGCCC

PFB0445c; -924 ATGCCC

PFL0625c; -922 AGGCCC

PF07_0080; -921 CCCAATAAAAAA

PFB0445c; -921 CCCATATATAAAAAAAATA

PFE0185c; -913 AAACCC

PF13_0132; -881 GTTTTCCC

PFL0310c; -878 AAAAAAAAGCCC

PF14_0401; -877 AGGCCC

PF13_0132; -876 CCCTTTTTTTT

PFL0310c; -872 AAGCCC

PFC1020c; -859 GTGCCC

PF13_0129; -845 AAGCCC

PF14_0296; -841 ACCCTTGTACC

PF14_0589; -825 AAACCC

PF10_0264; -823 TCTTTCCC

PF13_0132; -818 AAACCC

PF14_0589; -818 AAACCC

PF13_0045; -817 AAGCCC

PF14_0589; -816 ACCCTTGAACT

PFC0200w; -809 AAGCCC

PF13_0132; -808 ACCCACCTCTTT

PFE0185c; -799 ATGCCC

PF11_0272; -793 GGGCCC

PF11_0272; -785 CCCAAAAAAAAA

PF14_0401; -773 CCCTATTTATTTAAAGAAA

PFC1020c; -754 AAACCC

PFL2475w; -751 ACCCTTAAACA

PF10_0209; -748 CCCTTCCCAGG

PFD0455w; -739 CCCAACATTTCTCAAAATA

PFB0885w; -712 CTCTTCCC

PF14_0579; -702 CCCTCAACTCTACCACAGA

PF13_0171; -701 ACCCTTATGTC

PF14_0655; -667 AAACCC

PF07_0043; -659 CCCTTTATTAT

PFD0565c; -659 CTGCCC

PFC1020c; -637 CCCAAAAAAAAAAAAAAAA

PF14_0027; -635 GCCCAC

MAL13P1.92; -632 CCCAAAAAAATA

PF10_0272; -623 ATGCCC

PFC0535w; -618 GCCCTTCC

PF14_0627; -617 CCCTATTATATTCTATATA

PFC0295c; -606 GTTTTCCC

PF14_0104; -583 AAACCC

PFL0210c; -575 CTTTTCCC

PFF0885w; -564 AAGCCC

PF13_0132; -555 CCCAATAACTTTAAATATA

PF11_0312; -548 ATGCCC

PF10_0187; -545 GACCTCCCTCAC

PFC0775w; -531 AAGCCC

PFI0860c; -525 ACCCACGCATCT

PFE1005w; -497 ACCCTTAAACA

PFA0145c; -493 TTCTTCCC

PF11_0245; -487 AAGCCC

PFC0295c; -468 AAGCCC

PFL0675c; -468 ACCCATTGACC

PFL0625c; -465 CCCTAGATAATAACAAAAA

PF10_0272; -449 CCCAAAAAAAAAAAAAAAA

PFE0810c; -440 CCCAAAAAAAAA

PFI0415c; -437 ACCCATTTTTT

PF14_0027; -409 CCGCCCTAAGG

PF07_0079; -408 AAGCCC

PFE0185c; -385 AGGCCC

PF11_0051; -383 CCCTGAACTAAAAAAAAAA

PF14_0231; -378 AAACCC

PF14_0563; -376 ATGCCC

PF14_0185; -375 AAACCC

PFD0770c; -356 AAGAAAACCCGC

PFE1085w; -348 AAACCC

PF13_0214; -345 AAGCCC

PFC0290w; -338 CCCAATACATAAAAATATA

PF13_0214; -305 CCCATAAAAATAAAATATA

PFF1095w; -289 GTGTTCCC

PFB0885w; -280 GCCCTTATTTA

PF14_0183; -276 TCCCTTTTAAA

PFB0445c; -273 AAACCC

MAL13P1.92; -267 AAACCC

PFE0185c; -257 AAACCC

PFE1005w; -252 CCCTTTTTTTT

PF08_0096; -238 GGTCCC

PF14_0391; -230 AAGCCC

PF13_0049; -220 CCCAAAAAAAAA

PF13_0179; -185 ACCCTTCTTCAA

PF10_0038; -174 CCCAAAAAAAAAAAAAAAA

PFD1070w; -174 GGACCC

PFF0885w; -172 AAGCCC

PFA0145c; -168 CCCTTCATAAT

PF11_0260; -147 AAACCC

PF14_0083; -128 AAACCC

PF14_0185; -74 AAACCC

PFL0310c; -50 CCCTTTTTTTT

PF11_0313; -49 TTGTTCCC

PFD1070w; -31 ATGCCC

PF14_0104; -27 TTGTTCCC

tgtg

PFL0670c; -1983 TTTGTGCTTCC

PFC0300c; -1981 TGTGTGCGTGT

PF14_0083; -1877 ATATGTGGTT

PFE0885w; -1877 ATATGTGCAC

PF13_0132; -1860 GATATGCGTAG

PF13_0257; -1802 TTATGTGCAT

PFC0775w; -1794 TGTGTTCCTTT

PF11_0245; -1790 ATATGTGCAC

PFE0885w; -1761 ATATGTGCAT

PFB0830w; -1746 TATGTGTCCCT

PF13_0171; -1713 TAAGTGCACAT

PF13_0129; -1710 TCATGTGGAC

PFL0625c; -1691 ATATGTGCAT

PFC0535w; -1640 ATATGTGCTC

MAL7P1.81; -1633 TGTGTTCCTTT

PF07_0043; -1604 TGAGGTGTCCA

PF14_0240; -1599 TTGTGTGGCC

PFF0885w; -1593 TGTGTTCCTTG

PFF1500c; -1570 GGAGTGCATAT

PFE0845c; -1548 GTAATGCATGT

PF14_0296; -1387 ATATGTGCAT

PFL0210c; -1345 TCATGTGGGT

PF08_0076; -1178 TTATGTGCTT

PF14_0083; -1173 ATATGTGCCT

PF07_0088; -1143 TGTGAGGATGT

PF13_0045; -1142 TAAGTGCACTC

PF14_0198; -1126 ATATGTGGTC

PFB0885w; -1078 CATGTGATCAT

PFF0345w; -986 TATGTGACCAC

PF10_0149; -961 ATATGTGGTT

PF14_0391; -910 TAAGTGCTCTT

PFC1020c; -862 TTAGTGCCCAT

PFL2055w; -844 TTAGTGCTTGT

PFC0295c; -728 ACATGTGCAT

PFE0715w; -605 TGTGTGTACCG

PF10_0187; -548 TGTGACCTCCC

PF13_0268; -542 ATATGTGCTT

MAL13P1.14; -513 ATATGTGCTC

PF14_0185; -497 TTAGTGCCTAT

PF13_0170; -466 ACATGTGCAC

PF11_0065; -370 TTATGTGCTT

PFF1095w; -290 TGTGTTCCCAT

PF13_0205; -265 TGTGTCATTTT

PFE1390w; -248 TTATGTGCTT

PF11_0447; -239 TGTGACCCCAC

PF10_0077; -119 TTTGGTGCTGC

PFA0145c; -19 TGTATGCATAA

----------------------------------------------------------------

dna replication machinery - tgtg

PFI0235w; -1813 TGTGTG

PF13_0189; -1778 TGTGTG

MAL13P1.22; -1693 TACATGTGTGTG

PFC0340w; -1678 TATATGTGTGTA

PF14_0254; -1640 TTTTTTTGTGTA

PFD0590c; -1567 CCTGTGTGGT

PF10_0165; -1534 TATATATGTGTA

PF07_0023; -1513 TGGTGTGTGAAAAA

PF10_0165; -1412 TCCTTATGTGTA

MAL13P1.22; -1322 TGTGTGTTTTTCTTCTTCT

PFI0235w; -1307 CACTTATGTGTA

PFL0580w; -1276 TATATATGTGTA

PFD0790c; -1248 TATTTGTGTGTA

PFD0590c; -1209 TTCATATGTGTA

PF13_0189; -1199 CTCAGGGGTGTA

PF11_0117; -1190 CACATGTGTGTA

PF10_0362; -1153 TACTGTTGTGTT

PFL0580w; -1123 TGTGTG

PFL1285c; -1119 TCCTTTCGTGTA

PFF1225c; -1107 TTTATGTGTGTA

PFL0150w; -1022 ATGTGTGAGAAAAA

PF13_0328; -960 TATATATGTGCA

MAL7P1.21; -762 AGTGTG

PFB0840w; -715 AGTGTG

PF14_0602; -694 GCCATATGTGTA

PFL0150w; -677 TATATATGTGCT

MAL13P1.22; -669 AATGTGTGTG

PFL1120c; -619 CTTTTGTGTGTT

PFE0155w; -599 TTTGTGATATTTCATTTTT

PF11_0117; -575 TATATATGTGTA

MAL13P1.22; -563 TATATATGTGTA

PFE0155w; -540 TGTGGG

PF07_0023; -523 TGTGTG

PF10_0165; -502 TGTGTGGATACTTTTTCAT

PFB0840w; -492 TTTTGGTGTGGG

PFI0530c; -492 TATATGTGTGTA

PF14_0602; -391 AGTGTG

PF13_0251; -357 TATATATGTGTA

PFD0790c; -347 TGTGTG

PFL2005w; -325 TTCATTTGTGTA

PFD0790c; -324 TGTGGGTTTGTTTTGTCAT

PF13_0251; -292 TTTATGTGTGTA

PFE0155w; -274 TTTATGTGTGTA

PF11_0117; -259 TGTGTG

PFA0545c; -259 CATTAATGTGTA

PF13_0291; -235 TATATGTGTGTG

MAL7P1.21; -229 TCTTTTTGTGTA

PF07_0023; -216 TGTATGTGTGTA

PFE1345c; -180 TATATACGTGTA

PFB0895c; -119 TATATATGTGTT

PF13_0251; -105 TGTGTG

PFF1225c; -74 CATGTGAGTG

PFF1470c; -60 TATATATGTGTA

PFE0155w; -25 CTCAGGTGTGTG

PF14_0601; -18 TATTTATGTGTA

PF11_0117; -13 AGTGTG

dna replication machinery - g-rich – 4g+3g+2g+1g

PF13_0189; -1965 CTTATTATTGAGAGGGG

PFI0530c; -1887 AAAAAAAGAGGGATA

PFI0530c; -1810 AAAAGAAGGAA

MAL13P1.22; -1799 GGAGTAATACAAA

PFL1285c; -1777 TATTTTTTTTAAGGGGG

PFL0150w; -1666 AAAAAAAGAAGGAAA

PF14_0254; -1607 AGGAGAAGAAA

MAL13P1.22; -1603 GGAGAAATATTAA

PFB0840w; -1577 AAGAAGACGAG

PF10_0165; -1576 ATTTTGAGAAAGGGA

PFD0590c; -1575 TATATAATCCTGTGTGG

PFF1470c; -1566 GAAAAAAGAAG

PFC0340w; -1437 AGAATGAGAGAGATA

PF10_0165; -1427 AGGAGAG

PF13_0328; -1400 GAGAGAGCCAAA

PFL0580w; -1369 AGGAGAG

PFF1225c; -1345 TTTTTGTTCAAATGGGG

PF13_0251; -1329 AAGAAAAGAAA

PF14_0601; -1323 GAAAAAAAAAG

PFL1285c; -1282 GAGAGAAAAAGA

PFE0155w; -1251 GAGAGAACGAAA

PF13_0189; -1195 GGGGTGTAAATAA

PFC0340w; -1161 AGGAGAGAAAA

PFF1225c; -1067 TTTTTTTTTTTGGGGGG

PFB0895c; -1014 AGAAGGG

PFD0790c; -1003 AGGAGAG

PFE1345c; -951 AAAAGAAGAAA

PFL1285c; -946 AATAAAAGAAGGAAA

PF07_0023; -892 GAGAGAAAAATA

PFE0155w; -864 ATTATTTCACATTGGGG

PF14_0177; -805 GGAAAAAGGAG

PFL1655c; -788 AAGAAGAGGAG

PFL0150w; -772 AAGAGAAAGAA

PF13_0251; -727 GGAGAAAAAAAAA

PF10_0165; -688 GGAGAAAAAAAAA

PFL1120c; -686 AAGAAGAGAAA

PF10_0165; -638 GAAAAAAAAAG

PFF1225c; -582 AAAATGAGAAGGGCT

PFL2005w; -515 AAAGGGAGAGAGAGA

MAL7P1.21; -512 AGAAAAAGGAA

PFB0840w; -496 TTTTTTTTGGTGTGGGG

PFL1285c; -491 TATATCTTTCCTTGGGG

MAL13P1.22; -473 AGGAAAGAAAA

PFE0155w; -458 AAGAAAAGAAA

PFE1345c; -456 GAGAAAAAGAA

PFI0235w; -454 AAGAAAAGGAA

PFA0545c; -451 AGAAAAGGCAG

PFL0150w; -445 AAAGGAGATAAA

PF14_0602; -428 AAGGGAATCAA

PF14_0254; -364 AAAGGAGAAATA

PFD0790c; -355 TTTTTTTTTGTGTGTGG

PF11_0117; -350 AAGAAAAGAAA

PFD0590c; -310 TATATACACATGAGAGG

PF07_0023; -296 AAAAGAAGAAA

PFI0530c; -282 GAAGGATTTATA

PFD0790c; -255 GGAAGAG

PFI0530c; -235 AAAGGAGAAAAA

PF13_0251; -221 AAAATGTGAAGGAAA

PF13_0291; -216 AAAAGAAAAAG

PFF1225c; -175 GGAGAAAAAATAA

PFL1120c; -149 AAAAGAAAAAG

PFL2005w; -139 AAAAGAAGAAA

PFD0790c; -128 GGGAGAG

PFL2005w; -123 AAAAGAAAAAG

PF10_0362; -76 AAAAGGGGAAA

PFD0790c; -20 TTAAAGAGAAAGAAA

PFL0580w; -13 AAGAGGATAAAA

----------------------------------------------------------------

proteasome – g-rich – 4g+3g

MAL8P1.142; -1916 AAAAAGAGGGAAGGA

PF13_0033; -1915 CGGGAT

PF14_0716; -1781 ATTAAAAGGGAACGA

PF14_0716; -1721 GGGAGG

PF13_0033; -1561 GGGATTTTGAAGAACAA

MAL13P1.343; -1506 GGGAAT

MAL13P1.270; -1499 GGGAAAATGATATAAAA

MAL13P1.190; -1435 ACAAAGGGGGAAATA

PF11_0314; -1403 GACGGGTC

MAL13P1.270; -1388 AAATAATGGGAAAAA

PF14_0716; -1328 TCGGGAAG

PF14_0025; -1268 TTGGGGTA

PFA0400c; -1071 GAGGGGAG

PF13_0063; -1039 AGTAAGAGGGAATAA

PF14_0716; -1007 GGGCAT

PFD0665c; -989 GAGGGTTG

PFB0260w; -970 GGGCAT

PFE0915c; -969 CAGGGGGC

PFC0520w; -921 ATTATGTGGGAAAAA

MAL8P1.128; -913 ATGGGATG

MAL13P1.270; -863 TTGGGGTG

MAL8P1.142; -796 ATGGGGAG

PF14_0025; -740 AAAGGGAG

PF10_0174; -635 GGGCAA

PF14_0632; -615 ATAAGAAGGGAAGAA

PF13_0156; -548 AATGGGAG

PF13_0156; -510 GGGAAA

PF14_0025; -468 AAAAAGAGGGAAAAA

PFI1545c; -344 ATATAAAAGGGATGA

PF14_0676; -342 GGGTTAAAAAAAAGAAA

PFC0520w; -260 ATATTGAGGGAAGAA

PF14_0676; -245 ATAATAAAGGGAAGA

PFD0665c; -193 GGGAATTGTATTAAAAA

PF10_0081; -99 ACATAATGGGAAATA

PFA0400c; -42 TAGGGGTC

proteasome - tgtg

PF13_0033; -1982 ATGTGCAT

PF10_0081; -1973 ATGTGTAT

PF11_0314; -1716 ATGTGCAT

PF10_0081; -1632 TATGTATGTA

PF11_0314; -1621 ATGTGTAT

PFF0420c; -1615 GTGTGTAT

MAL13P1.190; -1508 GTGTGCAT

PF14_0716; -1507 TATGTATGTA

PF14_0716; -1491 TATGTATGTA

PF14_0716; -1475 TATGTATGTA

PF13_0063; -1447 ATGTGTAT

PF14_0025; -1421 GTGTGTAT

MAL13P1.343; -1326 GTATGTAT

PFC0520w; -1319 ATGTGTAT

PF11_0314; -1255 GTGTGTAT

PFB0260w; -1194 ATGTGTAT

PFC0520w; -1178 TGTGTATGTA

PFF0420c; -1088 TATGTATGTA

PFI1545c; -980 ATGTGTAT

MAL8P1.128; -919 ATGTGTAT

PF14_0632; -884 ATGTGCAT

PFA0400c; -839 ATGTGTAT

MAL13P1.343; -811 ATGTGTAT

PFI0630w; -800 ATGTGTAT

PF13_0156; -733 TATGTATGTA

MAL13P1.190; -697 TATGTATGTA

MAL13P1.343; -678 TATGTATGTA

PFC0520w; -669 ATGTGCAT

PFD0665c; -652 TATGTATGTA

PF10_0298; -576 ATGTGTAT

MAL13P1.190; -565 ATGTGTAT

MAL8P1.142; -474 ATGTGTAT

PFE0915c; -430 GTATGTAT

PFF0420c; -422 TTTGTATGTA

PF10_0081; -385 TTTGTATGTA

PF10_0298; -197 GTATGTAT

PF10_0174; -195 TATGTATGGA

PFC0785c; -162 ATGTGAAT

PFA0400c; -149 ATGTGAAT

PFI0630w; -76 TATGTATGTA

PFI0630w; -65 GTATGTAT

PFI0630w; -57 GTATGTAT

----------------------------------------------------------------

mitochondrial genes - g-rich – 4g+3g+2g+1g

PF14_0597; -1890 GCAAATAGGC

PF14_0373; -1793 GAAAAAGGAA

PF13_0359; -1340 ATGGGGA

PFL1725w; -1152 ATGGGCA

PF13_0353; -948 CTAAAGGCGC

PFE0970w; -770 GTAAAAAGGG

PF11_0485; -663 GTGAACGGAG

PF11_0485; -555 ATGCGGA

PF14_0248; -552 GTGAAAGGGC

PF13_0327; -534 GTGAAAGCGG

PF14_0248; -388 TAGCGCA

PFL1725w; -316 GCAAAAGGCA

PF10_0120; -300 GAAAAAGGAA

PF13_0061; -286 GTGAATGGCG

PF14_0373; -282 CAAAACGGGA

MAL13P1.47; -229 ATGCGCA

PFE0225w; -226 CTCAAAGGGG

PF14_0721; -204 GTAATAAGCG

MAL13P1.47; -107 GTAAAGGGGA

PF13_0327; -38 TTGGGCA

mitochondrial genes – tgtg

PF11_0485; -1308 TGTGTGTATGT

PFE0970w; -1287 GAGGTGTGCCA

PF13_0353; -943 GGCGCATGAAG

PF13_0353; -831 TGGGTGTGTGGTAA

PF14_0373; -798 TGTGTATACGA

PFE0225w; -738 GAGTGTTGGAC

PF11_0485; -664 TGTGAACGGAG

PFE0225w; -550 TATTAGTGTGTATA

PF13_0359; -439 TGTAAGTGTGTATA

PF14_0597; -402 TGTATGTATAC

PF13_0327; -371 TGGATGTGTGAAGA

PF14_0288; -318 TATCAGTGCGCATA

PF10_0120; -307 AGTGCATGAAA

PF14_0721; -274 TGTATGTGTGTTGA

PF14_0288; -237 CGTGCGGATGG

MAL13P1.47; -234 TGTGTATGCGC

PFE0225w; -144 ATGTGAAGGAA

PFL1725w; -109 TGTATGTGTAC

PF13_0061; -31 CGTGTGTGTAT

----------------------------------------------------------------

organellar translation machinery - c-rich - 4c+3c+2c

PF14_0166; -1697 TCCTCC

PFI0375w; -1650 TCCATGTGGG

PF14_0166; -1555 TCCTCC

PF14_0166; -1513 TCCTCC

PF14_0166; -1348 TCCCCA

PFL1895w; -1335 TCCCCC

PF14_0166; -1251 CCAATGGTGC

PF14_0642; -1131 TCCCAA

PF14_0658; -1116 TGCTCC

PF14_0289; -1063 TGCCCA

PFL1590c; -1022 TCCCCA

PFB0645c; -992 TCCCCA

PF11_0414; -970 TCACCT

PFB0390w; -957 TCCCAT

PFI0890c; -877 TCCCAT

PFL1590c; -816 GAGTTACCCA

PFI1240c; -815 ACACCT

PF14_0132; -811 TACCCT

PF14_0642; -799 TCCTCC

PF10_0332; -789 ACCCCT

PF14_0289; -778 ACACCT

PF10_0332; -759 ACCCAA

PFL1590c; -690 TCCCAT

PF14_0289; -684 TGCCAT

PF08_0014; -668 ACACCT

PFL1895w; -634 TGCTCC

PFI1240c; -631 TCCCCC

PFB0585w; -615 TCTCCC

PFL0770w; -593 TCCCAT

PFD0600c; -575 GCCCCT

PF08_0014; -538 ACACCT

PFB0645c; -522 GGTCCC

PFL1540c; -505 ACCCAA

PF14_0212; -496 ACCCAA

MAL13P1.164; -482 TGCTCC

PFL1590c; -482 GCTCCC

PFD0600c; -473 GCCTCC

PFE0960w; -472 TCCATTTTGC

PFL1590c; -471 TCCATTTTGG

PF14_0606; -465 TCCCAT

PF08_0011; -457 GGCTCC

PFL1590c; -453 TCCCCT

PF14_0642; -445 GGCCCT

PF11_0386; -434 TGCCAT

PFI1575c; -428 TCCCCC

PFB0390w; -397 GACCCC

PF14_0132; -389 TCCCAT

PFE1225w; -378 TCCCCT

PF14_0212; -363 ACCCAA

PFF0115c; -353 TACCCT

PFI1240c; -312 TACCCT

MAL13P1.281; -307 ACCCAA

PFE0960w; -282 TCCCCC

PF08_0011; -229 GAGTTCCCCA

PF14_0212; -211 TACCCT

PF07_0062; -204 TCTCCC

PF11_0181; -169 ACACCT

PFI0890c; -151 TGCCTGTTGG

PFL1150c; -145 TCCCGT

PFB0585w; -111 TCCCCT

PF14_0166; -110 ACCCAA

PF14_0212; -88 TCCCAT

MAL13P1.164; -86 ACACCT

PF14_0270; -84 TCCCAA

PFI0375w; -57 TCCCAT

PFL1540c; -16 ACCCCT

----------------------------------------------------------------

merozoite invasion – caca

MAL7P1.176; -1888 TACACACA

PF08_0108; -1872 TATACACA

PF14_0392; -1776 CGAGCACA

MAL13P1.176; -1770 GAACATACATTGGAG

PFF0520w; -1750 TATACACA

PFI1685w; -1741 CGAGCACA

PF14_0492; -1684 ATGCGCAT

MAL13P1.278; -1682 GTGCACATGTATGCA

MAL13P1.119; -1680 TATACACA

PF13_0198; -1592 TATACACA

PF11_0381; -1583 ACACAGGGCTTA

MAL13P1.176; -1579 AGTACATTTC

PF08_0129; -1542 ACGTACAC

PFI0265c; -1541 TATACACA

MAL13P1.119; -1535 TATACACA

PFB0310c; -1514 ACACAGGAATAA

PFA0125c; -1503 TATACACA

PF11_0395; -1477 TATGCACA

PFI1475w; -1472 ACACATGGAAAA

PF11_0344; -1467 ACGTGCAT

PFI0265c; -1465 TATACACA

MAL13P1.176; -1446 GTGTGCAC

MAL13P1.118; -1445 TACGCACA

PFI1475w; -1417 CACACACA

MAL13P1.278; -1384 TATGCACA

PFB0665w; -1361 GTGTACAC

MAL13P1.60; -1331 ATACACAC

PF13_0198; -1331 GAACATACATTGGAG

PFB0310c; -1311 AGTACACA

PFI1685w; -1302 CCACACGAATAA

PF10_0268; -1275 ACGTGCAC

PFB0310c; -1250 ACACAGGGGGTA

PFI0265c; -1233 ACGCAAGGTGC

PF13_0197; -1229 AACGCACA

PFB0665w; -1166 CATACACA

PFC0945w; -1152 GCACACAC

PFI1485c; -1143 TGAACACA

PF13_0198; -1142 AGTACATTTC

PFE0420c; -1127 ACACAGGGTAAT

PFI1685w; -1094 GTGTACAC

MAL13P1.176; -1087 CCACAAGGTGTA

MAL13P1.119; -1060 AGAACATATC

PFF0995c; -1044 AGTACATTTC

PF14_0281; -1038 TATGCACA

PF07_0072; -974 TATACACA

MAL13P1.118; -958 TACGCACA

PF11_0377; -938 TATGCACA

PFE0080c; -930 ATGTGCAC

PF13_0198; -929 ATGTGCAC

PFC0945w; -926 GGTGCACA

PFF0995c; -926 ACACACAC

PFL2520w; -923 GCCACACA

PF10_0345; -894 ACACAAGAAAAA

PF14_0492; -893 GCACGCAC

PFL1385c; -885 GCATGCAC

PF11_0298; -878 GAGCTCACAAAGTAA

PF14_0681; -874 TATACACA

PFF0615c; -873 TATACACC

PFE0355c; -783 GTGCACATAAAAAAA

PF07_0072; -752 CATGCACA

PF11_0298; -742 GTATGCAC

PF11_0395; -736 TATACACA

PF10_0268; -735 TATGCACA

PFB0315w; -714 CCCACACA

PF14_0492; -696 TATACACC

PF08_0129; -687 TGCGCACA

PFB0345c; -685 GCACATGGACGA

PF10_0346; -663 TGCACACA

PF14_0281; -658 TATGCACA

PFI1475w; -658 TATACACA

PF13_0198; -648 CCACAAGGTGTA

PFE0370c; -626 AGTACACA

PFB0340c; -614 ATGTACAC

PF14_0102; -606 TATACACA

PFI1685w; -602 TGTACACA

PFB0150c; -581 CCTGCACA

PF13_0197; -571 ACACACGC

PFL1110c; -549 CGTGCACA

PFL2520w; -513 TATACACA

MAL13P1.119; -506 AGTACACA

PF14_0492; -472 ATGCGCAT

PFB0310c; -460 GTGTACAC

PFB0345c; -456 GTGTACAC

PF11_0344; -436 ACGTACAC

MAL13P1.176; -434 GACCACACTATTTTG

PF10_0346; -431 GTGCACAT

PF14_0492; -415 GTGCATGAATGCGCA

PF11_0157; -380 TATGCACA

PF10_0345; -368 TGCACACA

PF14_0224; -366 GGTACACC

PFB0665w; -357 CACGACGTTCTC

PFE0075c; -352 ATGTGCAC

PFB0345c; -339 TACACCAC

PFD0255w; -335 ACGTGCAC

PFF0615c; -312 ATGTACAC

PFF0615c; -293 CCACATGTGGTG

PF14_0281; -291 ATGTACAC

PF11_0344; -288 GCACAAGAAAAA

PF11_0381; -275 CATACACA

PF14_0281; -253 ATACACAC

PFI1685w; -227 TATACACA

PF14_0102; -177 TATGCACA

PFI1685w; -164 TATACACC

PFC0945w; -132 GTGCGCAC

PFF0520w; -96 AGAACATATC

PFF0615c; -94 GTGTACAC

PFB0305c; -74 CACACACA

PF14_0346; -73 TATACACA

PFB0305c; -73 ACACACAC

PFE0355c; -55 ACGTGCAC

PFB0350c; -11 TATACACC

merozoite invasion - tgtg

PFI1685w; -1999 ATATATGTGTA

MAL13P1.119; -1982 GTGCGT

PFI1685w; -1895 ATGTGTATATA

PF10_0346; -1891 GTGTGT

PF11_0381; -1854 AGGTGTATATA

PFI1685w; -1752 GTGTGT

MAL13P1.278; -1678 ACATGTATGCA

PF08_0129; -1509 ATATGTATGTA

PFE0080c; -1508 GTGTGC

PF07_0072; -1506 GTGTGT

MAL13P1.119; -1499 ATATGTGCGTA

PF08_0129; -1493 GTATGTATGTA

PF11_0344; -1467 ACGTGCATGCC

MAL13P1.176; -1446 GTGTGC

PF11_0344; -1439 GTGTGT

PF11_0377; -1418 ATATGTATGTA

PFB0310c; -1414 ACATGTGTGTA

MAL13P1.118; -1411 ATATATGTGTA

PFL1385c; -1345 GTATATGTGTA

PF08_0129; -1298 ATATGTATGTA

PFC0945w; -1272 ATATGTGCGTA

MAL13P1.118; -1196 ATATGTATGTA

PFL2520w; -1165 ATGTGTATATA

PFF0520w; -1116 GTGTGC

PFB0315w; -1110 ATATGTGTGTA

PFF0615c; -953 GTGTGT

MAL7P1.73; -935 GTGTGT

PFL1110c; -929 GTGTGT

PFE0080c; -921 GTGTGT

PFI0265c; -909 ATATGTATGTA

PFI0265c; -886 ATATGTATGTA

PFC0945w; -872 ATATATGTGTA

PF14_0492; -861 GTATATGTGTA

PF07_0072; -813 GTGTGT

MAL13P1.278; -804 ATATGTATGTA

MAL13P1.176; -786 ATATGTGTGTA

PF11_0377; -722 ATATGTATGTA

MAL7P1.73; -717 GTGTGT

PF11_0377; -706 GTATGTATGTA

PFI1475w; -706 GTGTGT

PF11_0377; -694 GTATGTATGTA

PFB0150c; -691 ATATGTATGTA

MAL13P1.176; -670 GTATGTATATA

PF11_0157; -657 GTGTCTATGCA

PFD0255w; -625 GTGTGTGCGCC

PF14_0392; -603 ATATGTATGTA

PFC0945w; -580 ATATGTATGTG

PFE0080c; -578 ATGTGTATATA

PF07_0072; -570 GTATGTATATA

PF08_0129; -568 GTATGTATATA

PF11_0377; -559 ATATGTATGTA

PFB0315w; -535 ATATATGTGTA

PFL1385c; -499 GTGTGTGTGTA

PF13_0197; -475 GTATATGTGTA

MAL7P1.176; -440 ATATGCATGTA

PF14_0224; -387 GTGTGC

PFA0125c; -381 GTGTGT

PFB0340c; -355 AGAGGTGTGCA

PFB0315w; -345 GTGTGT

PFE0420c; -269 GTGTATATGTA

PFA0125c; -233 ATATATGTGTA

PF13_0198; -231 GTATGTATATA

PFB0815w; -224 AAATGTATGTA

PFA0125c; -221 ATATGCATGTA

PFB0315w; -219 GTGTGCGTGCA

PFI1475w; -204 ACATATGTGTA

PF14_0281; -199 ATATATGTGTA

MAL7P1.73; -189 GTGTGTGTGTA

PF14_0281; -183 ATGTGTATGTA

PFE0075c; -180 ATATGTATGTG

PF14_0281; -171 ATATGTATGTA

PFF0995c; -141 ATATATGTGTA

PFE0370c; -137 GTGTGT

PFC0945w; -132 GTGCGC

PFB0315w; -98 GTGTGT

PFF0615c; -98 GTGTGTGTACA

PF11_0395; -86 ATGTGTGTACC

MAL13P1.119; -81 ATATATGTGTA

PF11_0377; -79 GTATGTATATA

PF14_0492; -77 GTATGTATGTG

PF11_0298; -53 ATATATGTGTA

merozoite invasion – g-rich - 4g+3g+2g

PF14_0102; -1979 GTAGGAAAAAA

PFC0945w; -1959 ATTTATATAAGGGG

PFI1475w; -1936 ATATATATGTGGGG

PFB0310c; -1930 AAAGGAAAAAA

PFC0945w; -1792 CAAGGAAAACA

PFE0370c; -1771 ATATTTAAAAGGGG

PF14_0392; -1731 GAAGGCAAAGG

PF11_0377; -1724 GGAGGAAAAAG

MAL13P1.278; -1693 CACGGGAAAAA

PFB0310c; -1692 CAGGTGAATAATTTTAAAATA

PF13_0198; -1660 AACGGGAACAA

MAL13P1.119; -1638 AAAGGAAAACA

MAL13P1.119; -1619 TTATAATTATGGGG

PFB0310c; -1586 ATATATAAATGGGG

PFF0995c; -1543 ATATGAAGAGG

PF14_0102; -1527 AAAGGAAAAAA

PFE0080c; -1511 AGGGTGTGCAGATAAAAAAAA

PFI1475w; -1469 CATGGAAAAAA

PFB0310c; -1444 GAAGGAAGAAA

PF11_0344; -1442 GAGGTGTGTTGGGAAACAGAA

MAL7P1.176; -1435 CAAGGAAAACC

MAL13P1.176; -1405 ATAGAAAAAAGGGG

PFE0075c; -1271 AAAGGAAAAAA

PF14_0492; -1257 AAAGGGAGAAC

PFB0310c; -1255 ATATTACACAGGGG

PFL1385c; -1238 AAAGGAGAAAA

PFI0265c; -1233 ACGCAAGGTGCA

PF14_0102; -1199 ATAGACGGGGAA

PFB0345c; -1182 AAAGGAAAAAA

PF08_0129; -1137 AAAGGGAAAGT

MAL13P1.60; -1120 TTACACGGTGAA

PF10_0345; -1117 GTTGGAGAAAA

PFB0340c; -1103 GATGGAAAAGC

PF07_0072; -1097 GACGGAAAAGG

PF11_0395; -1089 ATGGGGAAATGTAATTTTGAA

MAL13P1.176; -1087 CCACAAGGTGTA

PFB0315w; -1040 TTAAAAGGTGTA

PF11_0381; -1033 ATGGGAAAAAA

MAL7P1.176; -968 CAAGGAAAACC

PFB0315w; -956 ATAGGGAAAGGAACAGAAAAA

PFI1685w; -954 GTGGGGTGGCAAAAAAAAAAA

PF08_0108; -946 ATATGGAACCA

MAL13P1.119; -855 CAATGGAAAAT

PFI1475w; -835 AAAGGAAAAAA

PF13_0197; -817 AAAGGAAAAAA

PF14_0281; -817 ATAGGAAGAAA

PF13_0197; -798 AAAGGAAAAAA

PF13_0197; -778 AAAGGAAAAAA

PFI0265c; -763 AAAGGAAAACA

PF13_0197; -759 AAAGGAAAAAA

PF13_0197; -741 AAAGGAAAAAA

PF13_0197; -723 AAAGGAAAAAA

PF13_0197; -704 AAAGGAAAAAA

PF07_0072; -696 ATAAATGGGGAA

PF13_0197; -684 AAAGGAAAAAA

PFB0345c; -679 GGACGAAAACG

MAL13P1.118; -664 TAGGGGTGGGGAAAATAAATA

PF13_0198; -648 CCACAAGGTGTA

PF10_0346; -576 AAAGGGAAAAGAAAAATAAAA

PFE0420c; -568 TTGGAGGGGGAA

PFF0615c; -539 AAAGGGATAAA

PFE0075c; -485 TTAAATGGTGTA

PF07_0072; -457 ATGGAAGGTGGA

PFF0520w; -452 GAAGGAATAAG

PFL1385c; -446 TTTTTTTTTTGGGG

PFB0665w; -433 AAAGGAAGACA

MAL13P1.60; -421 AATGGGAAAAA

PF14_0346; -419 AAAGGAAAAAA

PF14_0681; -418 GAATGAAATGG

PFD0255w; -367 GATGGGAACAA

PF13_0198; -353 ATTATTATATGGGG

MAL13P1.119; -329 TTAAATGGTGTA

PFB0305c; -325 GAATGGAACCA

PFF0615c; -287 GTGGTGTGAAAAAAAAAAAAA

PF11_0344; -264 CAAGGGAAAAA

PFI1485c; -257 GAAGGAAAAAA

PFE0355c; -168 AAGGGGAGAAGTATATATTAA

PF11_0395; -151 TTATAAAGTAGGGG

MAL13P1.176; -66 GAAGGGAAGAA

PFC0945w; -45 TAAAAAGGTGTA

PFB0350c; -44 AACGGAAAAAA

PFB0150c; -32 GAGTGGAAAGG

MAL13P1.176; -17 ATAGGAAGAAA

PF14_0224; -12 AAAGGAAGAAG

merozoite invasion – c-rich - 4c+3c

PF13_0197; -1971 TTTCCCAT

PFI0265c; -1958 TTTCCC

PFB0665w; -1947 TTTCCCAT

PFC0945w; -1947 GGTCCC

PFF0520w; -1919 TTCCCC

PFI1475w; -1884 TTTCCC

PFB0315w; -1880 TACCCC

PF11_0381; -1866 GTTCCC

PFC0945w; -1795 GCCCAAGGAAAA

PFL2520w; -1765 TTTCCC

PF14_0102; -1759 TATCCCCT

MAL13P1.278; -1719 TACCCC

MAL7P1.176; -1673 TGCACCCT

PF08_0108; -1669 TTTTCCCT

PF10_0268; -1572 CTTCCCCC

PFI1475w; -1436 TTTCCC

MAL13P1.118; -1332 TATACCCC

PF07_0072; -1244 TGTCCCAT

PF11_0377; -1217 TTTCCCAT

PFE0420c; -1173 TTTCCCAT

MAL7P1.73; -1163 TGTCCCCC

MAL13P1.119; -1115 GTGCCCATATATATA

PF11_0381; -1100 TTTTCCCC

PF14_0281; -1074 CTTTCCCT

PFE0370c; -1062 TTTCCC

PF11_0298; -1033 TTTCCC

PFB0310c; -1018 TTTTCCCC

PF13_0198; -978 TTTTCCCC

PFB0665w; -903 TTTCCCAT

PFE0370c; -858 TTTCCC

PF07_0072; -797 TTTCCC

PFB0150c; -773 TTTACCCC

PF11_0395; -761 TTTCCC

PFC0945w; -731 TTTTCCCT

PFB0315w; -717 TTTCCC

MAL13P1.176; -714 GTTCCC

PFB0315w; -714 CCCACACA

PF08_0108; -697 TTTCCCAT

PF14_0281; -675 TTTTCCCT

PFI0265c; -668 TGCCCCAT

PFB0315w; -492 TTTTCCCT

PFL1110c; -456 TTTCCCAT

PFE0075c; -361 TATTCCCT

MAL13P1.278; -349 TTTTCCCC

PF13_0198; -275 GTTCCC

PF11_0395; -183 TATTCCCC

PFL1110c; -177 TTTTCCCC

PFF0615c; -159 TATACCCC

MAL13P1.176; -129 ACCCATGGAGCA

----------------------------------------------------------------

actin myosin motility – g-rich – 4g+3g+2g+1g

PFL1435c; -1956 AAATGTGTGTAT

PFL1435c; -1886 AGGGGAATATCAATA

PFL2225w; -1856 GAAAGAAGGAAA

PFL2215w; -1845 GTGGGGTTTAAAATA

PFL2215w; -1817 GTGGGCTATCCACGA

PFL2215w; -1792 GAGGGAACATATATA

PF13_0233; -1778 AAAATAGGGAAA

PFL2225w; -1514 GAAAGAAGGAAA

PFL2460w; -1502 AAGGGAAACAAAAAG

PF11_0114; -1460 ATGTGAAAAAAAAAA

PF13_0233; -1349 AAGGGTACTTAAATA

PFL2225w; -1327 AAGTGTACAACAAAA

PFE0165w; -1309 ATGTGCACAAAAAAA

PF11_0114; -1283 GAAAGAGAGAAT

PFL1435c; -1238 GCGTGGTGTGCACAA

PFL1435c; -1169 GAGGGGGATAAAAAG

PFL2225w; -1059 AAGGGAAAAAAAAAA

PFL2460w; -907 GTACGAGTGTGT

PF13_0326; -666 GAGAGAGAGAGA

PFL2460w; -559 GTGTGTTTATAAATA

PFF0675c; -556 AAAAGGGGGAAA

PFL2460w; -535 GTGGGTTATACACAA

PF13_0233; -406 GTGTGATAATAAAAA

PF13_0326; -322 GTATGTGTGCAT

PFF0675c; -322 AAACGTGTGTGT

PFE0165w; -267 AAAGGAAATAAA

PFL2215w; -224 AAAAGAGAGAAA

PFE0175c; -188 GGAAGCGGGGGA

PFE0175c; -147 ACATGTGTGCAT

PF11_0114; -139 ATGGGCAAATAAATG

PFL2460w; -71 GAGAGAGAGAAA

actin myosin motility – caca

PF13_0233; -1956 CATGCACA

PFL1435c; -1675 AATGCACC

PFL2215w; -1669 TATACACA

PFL2225w; -1650 AACACACA

PFL2460w; -1534 CACGCACA

PFE0165w; -1363 TTTGCACA

PFE0165w; -1308 TGTGCACA

PFL2215w; -1242 AATGCACA

PFL2215w; -1234 TATACACA

PFL1435c; -1233 GTGTGCAC

PFL1435c; -1218 TGCACACACC

PFL2215w; -1143 TACGCACA

PFE0165w; -1132 CTTACACA

PFL1435c; -1101 TACGCACA

PFE0165w; -1080 CATGCACC

PF13_0233; -1042 GTACGCAA

PF13_0233; -969 TATACACA

PFL2460w; -911 GTGTGTAC

PFL2225w; -909 TTTGCACA

PF13_0233; -797 TTTGCACA

PF13_0233; -723 TATACACA

PF13_0326; -703 TATACACA

PFE0165w; -624 GGTACACACC

PFL2215w; -593 TATACACA

PFL2460w; -529 TATACACA

PFL1435c; -482 TGTCCACA

PF11_0114; -300 TATACACA

PFL2215w; -156 CACACACG

PFE0175c; -126 TATGCACG

PFL1435c; -122 TGTCCACA

PFF0675c; -92 TGTACACATA

actin myosin motility – tgtg

PFL1435c; -1953 TGTGTG

PFL2215w; -1850 CATGTGTG

PF13_0233; -1585 TAAGCGTG

PFL2460w; -1575 TGGATGTG

PFL2225w; -1353 TGTGTG

PFL1435c; -1233 GTGTGCA

PFL2460w; -914 TATGTGTG

PFL2460w; -904 CGAGTGTG

PFL2460w; -562 GGTGTGTG

PF13_0233; -407 TGTGTG

PF13_0326; -321 TATGTGTG

PFF0675c; -319 CGTGTGTG

PF13_0326; -200 TGTGAGTG

PFL2460w; -159 TATGTGTG

PFE0175c; -146 CATGTGTG

----------------------------------------------------------------
