# Supplementary material for: Identification of putative regulatory motifs in the upstream regions of co-expressed functional groups of genes in Plasmodium falciparum
Source: BMC Genomics. 2009 Jan 13;10:18. doi: 10.1186/1471-2164-10-18 (PMC2662883; doi:10.1186/1471-2164-10-18)
Supplement: Additional file 7 — Feature maps for the strong motif groups identified in the upstream regions of the 13 functional groups of genes. The maps have been obtained after sorting the motifs in each strong motif group. [file 1471-2164-10-18-S7.ppt]

## Slide 1
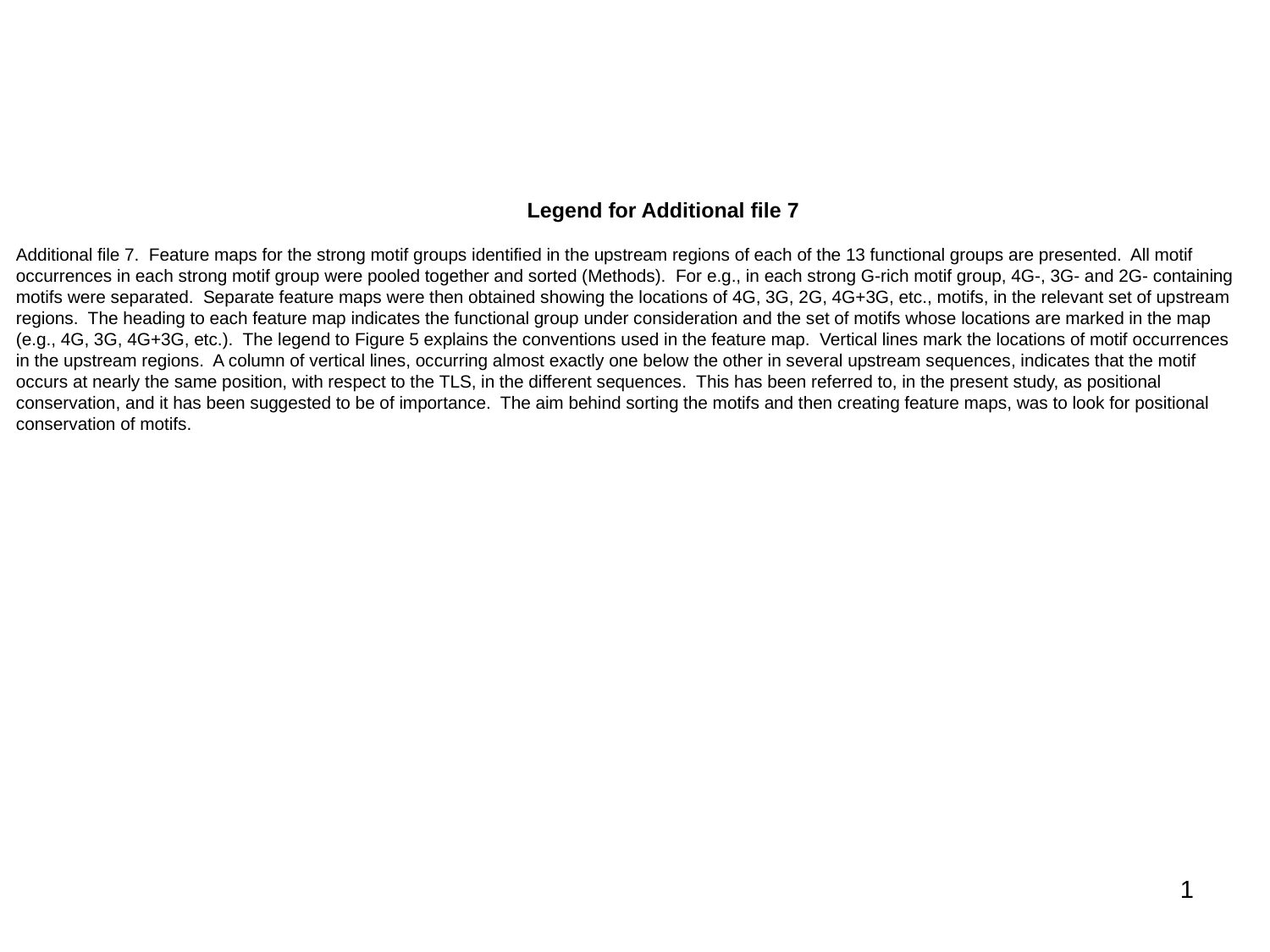

Legend for Additional file 7
Additional file 7. Feature maps for the strong motif groups identified in the upstream regions of each of the 13 functional groups are presented. All motif
occurrences in each strong motif group were pooled together and sorted (Methods). For e.g., in each strong G-rich motif group, 4G-, 3G- and 2G- containing
motifs were separated. Separate feature maps were then obtained showing the locations of 4G, 3G, 2G, 4G+3G, etc., motifs, in the relevant set of upstream
regions. The heading to each feature map indicates the functional group under consideration and the set of motifs whose locations are marked in the map
(e.g., 4G, 3G, 4G+3G, etc.). The legend to Figure 5 explains the conventions used in the feature map. Vertical lines mark the locations of motif occurrences
in the upstream regions. A column of vertical lines, occurring almost exactly one below the other in several upstream sequences, indicates that the motif
occurs at nearly the same position, with respect to the TLS, in the different sequences. This has been referred to, in the present study, as positional
conservation, and it has been suggested to be of importance. The aim behind sorting the motifs and then creating feature maps, was to look for positional
conservation of motifs.
1

## Slide 2
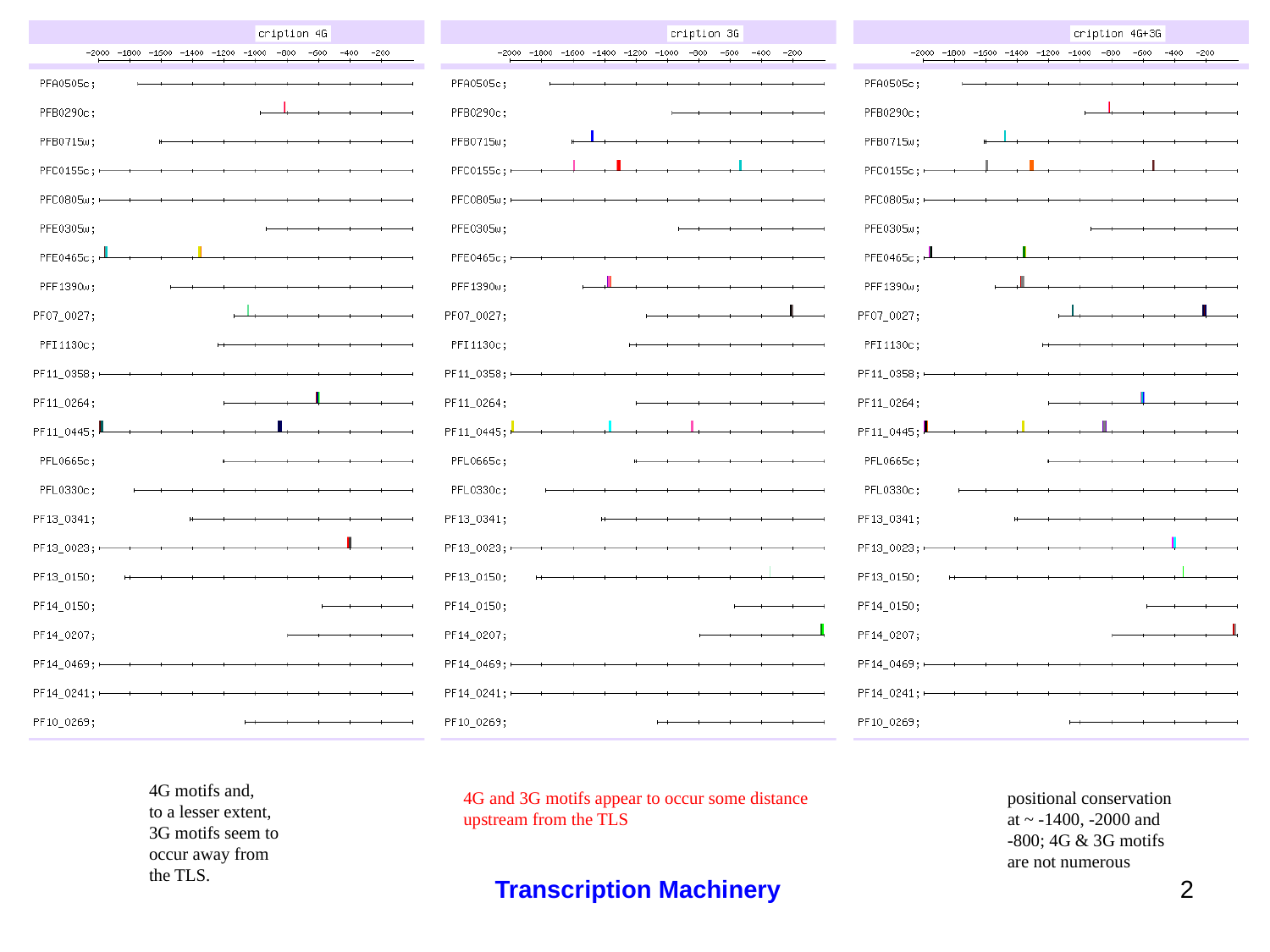

4G motifs and,
to a lesser extent,
3G motifs seem to
occur away from
the TLS.
4G and 3G motifs appear to occur some distance
upstream from the TLS
positional conservation
at ~ -1400, -2000 and
-800; 4G & 3G motifs
are not numerous
2
Transcription Machinery

## Slide 3
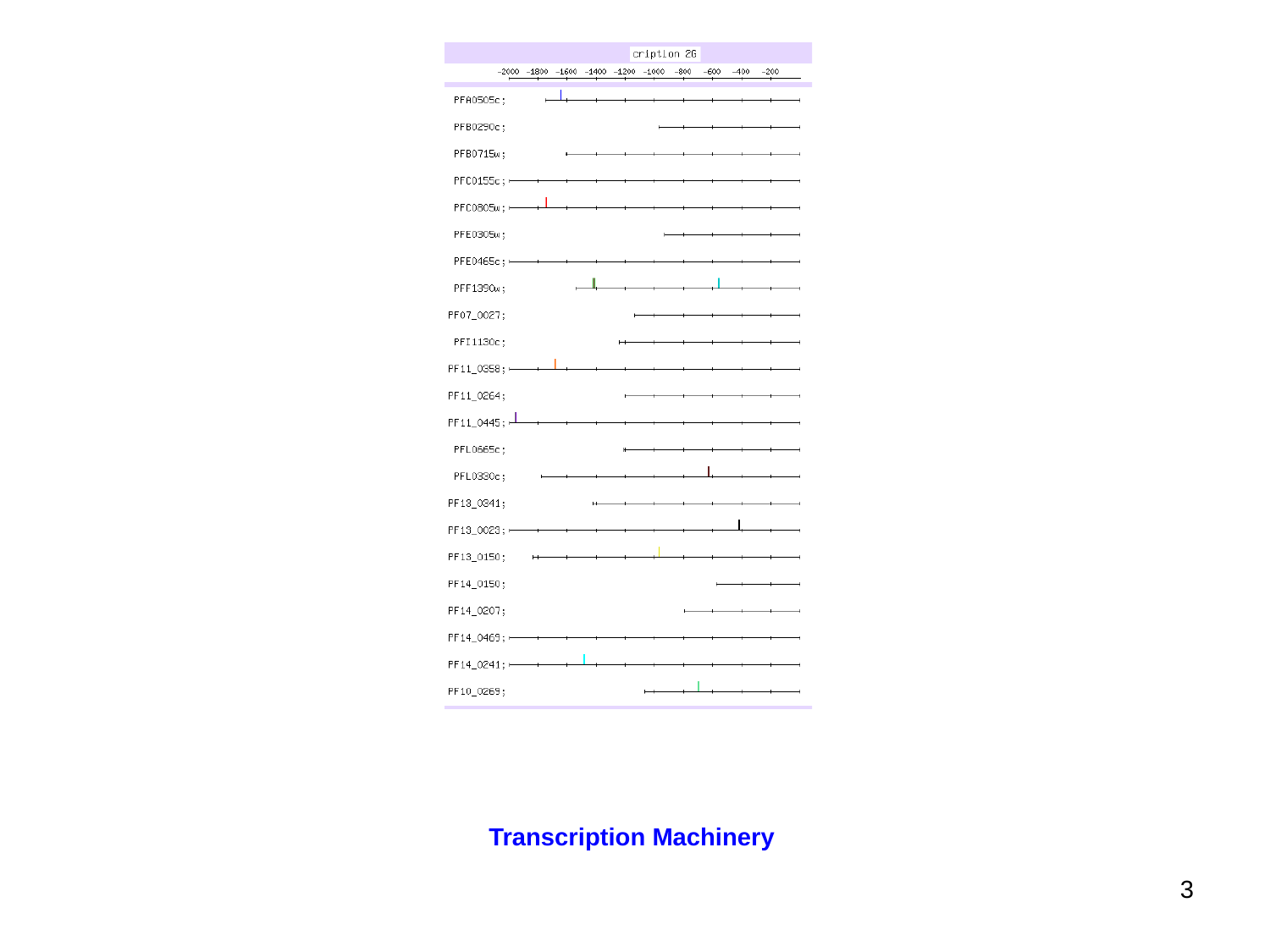

Transcription Machinery
3

## Slide 4
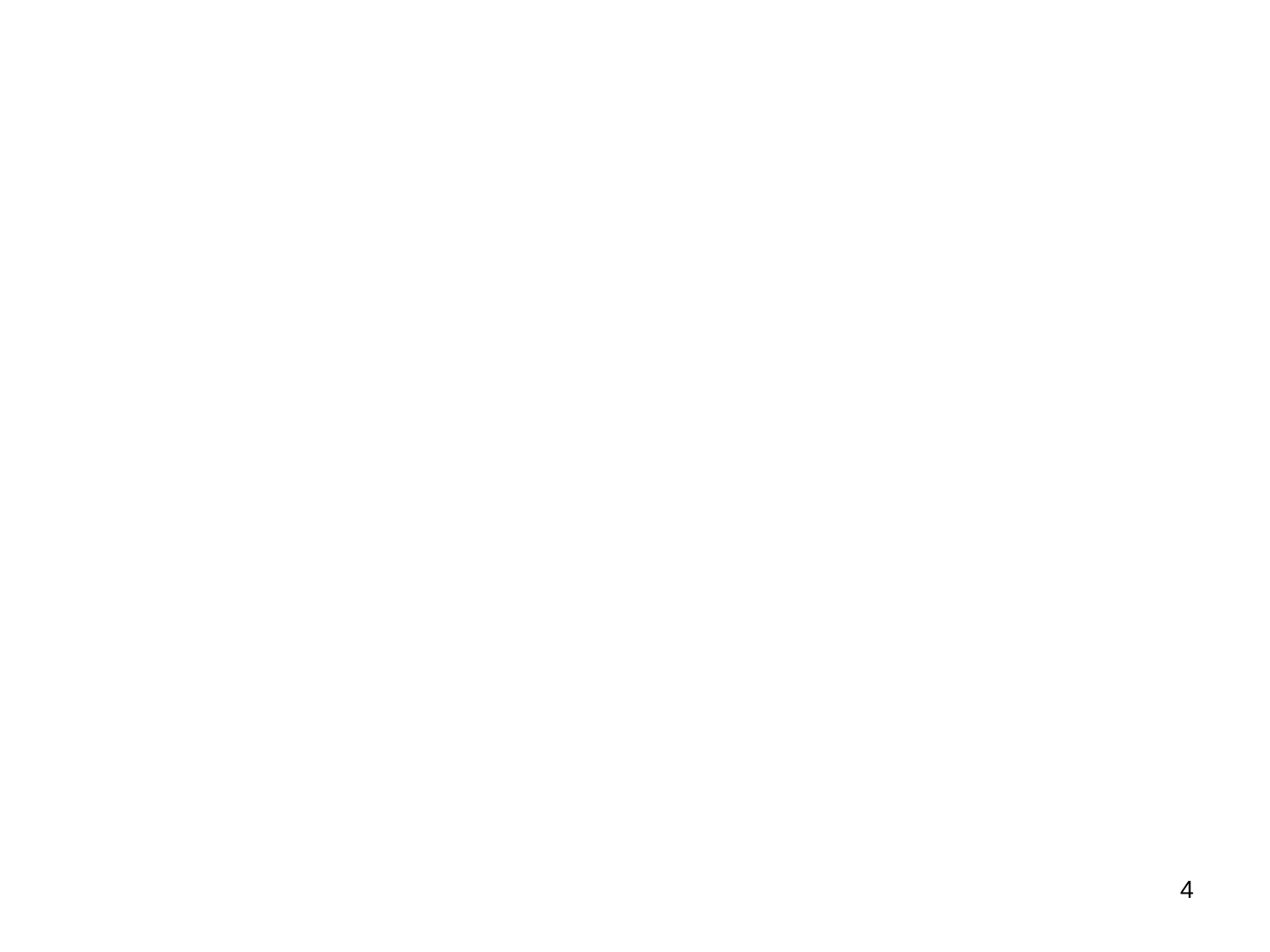

4

## Slide 5
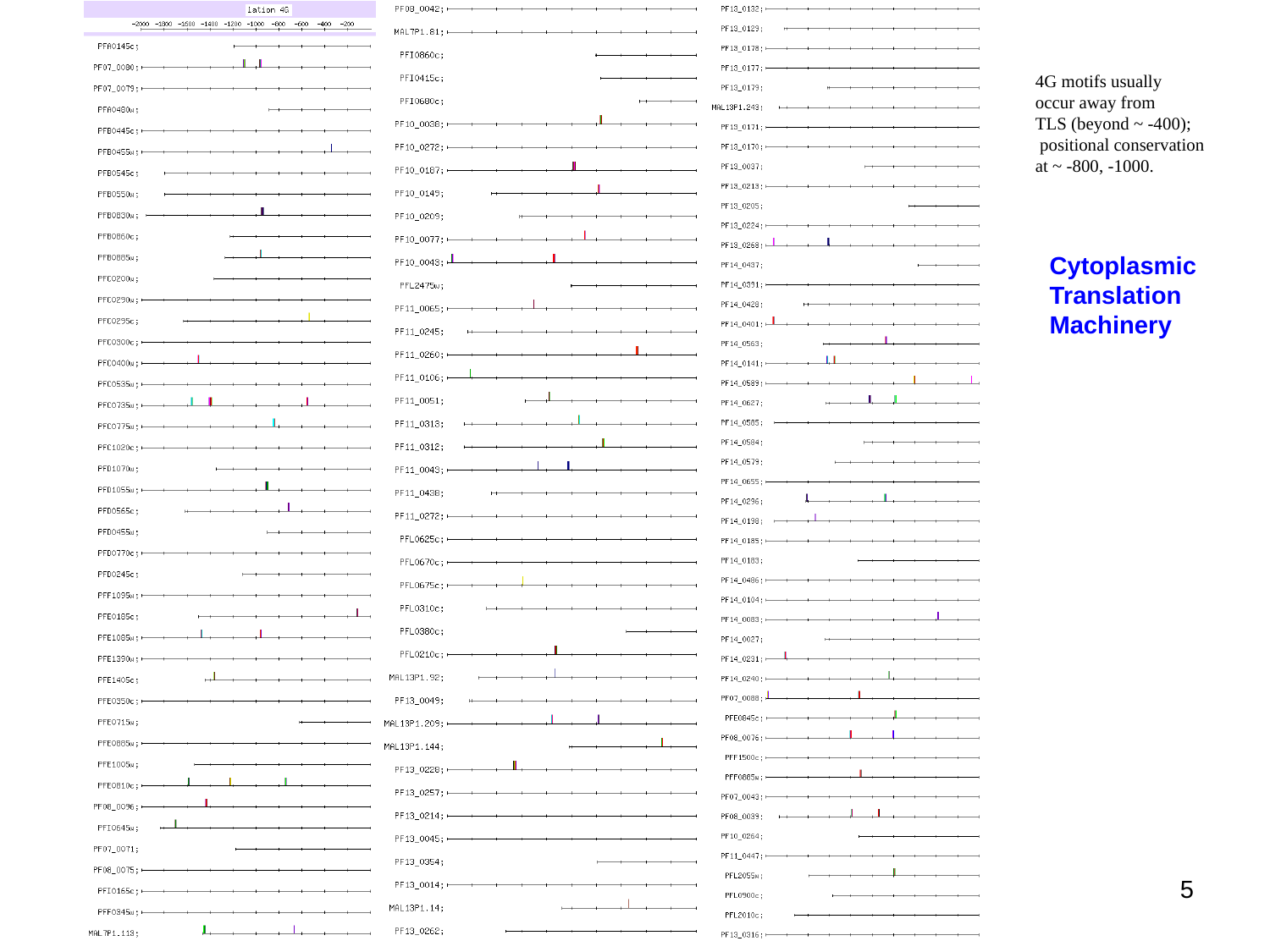

4G motifs usually
occur away from
TLS (beyond ~ -400);
 positional conservation
at ~ -800, -1000.
Cytoplasmic
Translation
Machinery
5

## Slide 6
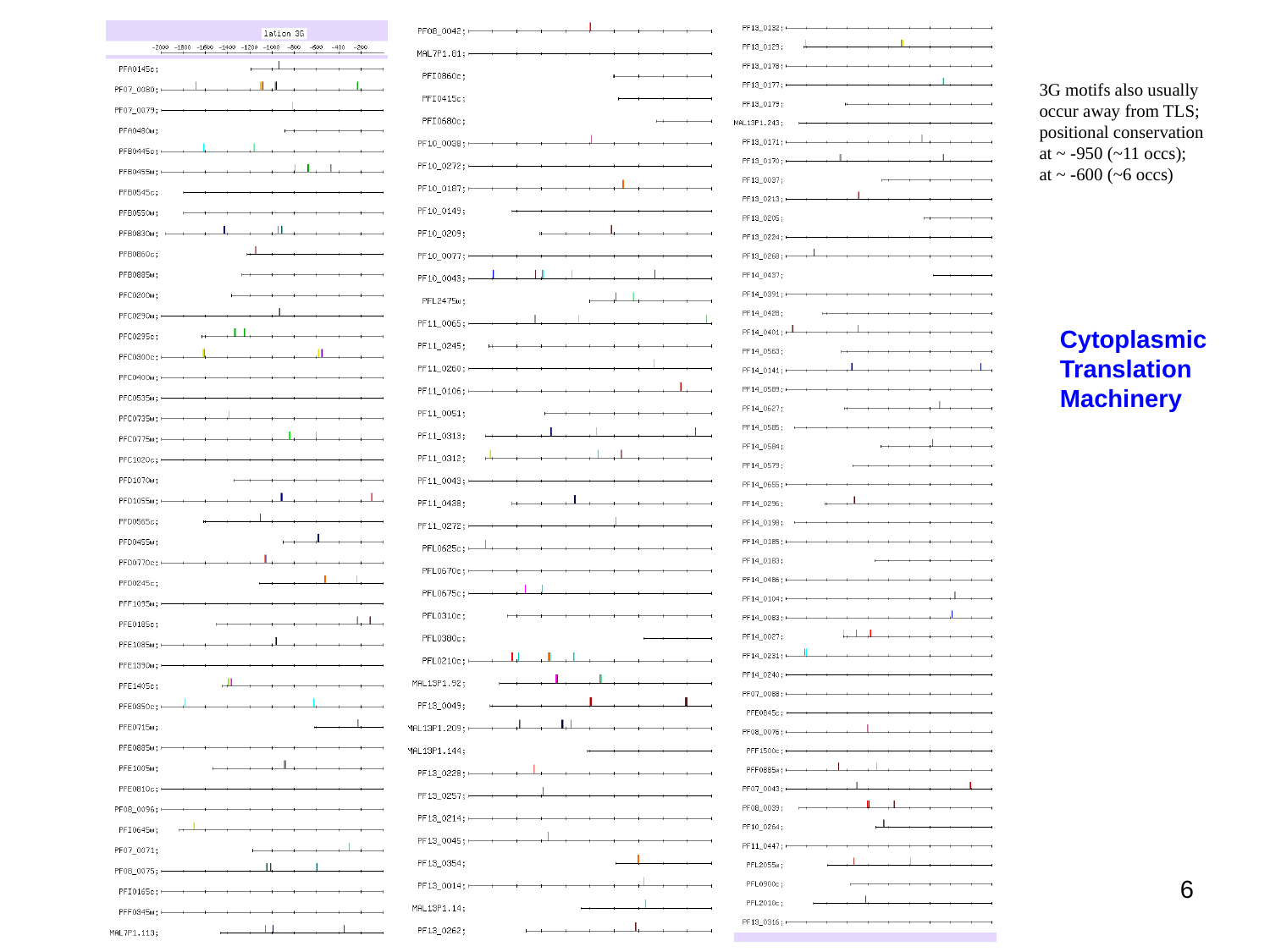

3G motifs also usually
occur away from TLS;
positional conservation
at ~ -950 (~11 occs);
at ~ -600 (~6 occs)
Cytoplasmic
Translation
Machinery
6

## Slide 7
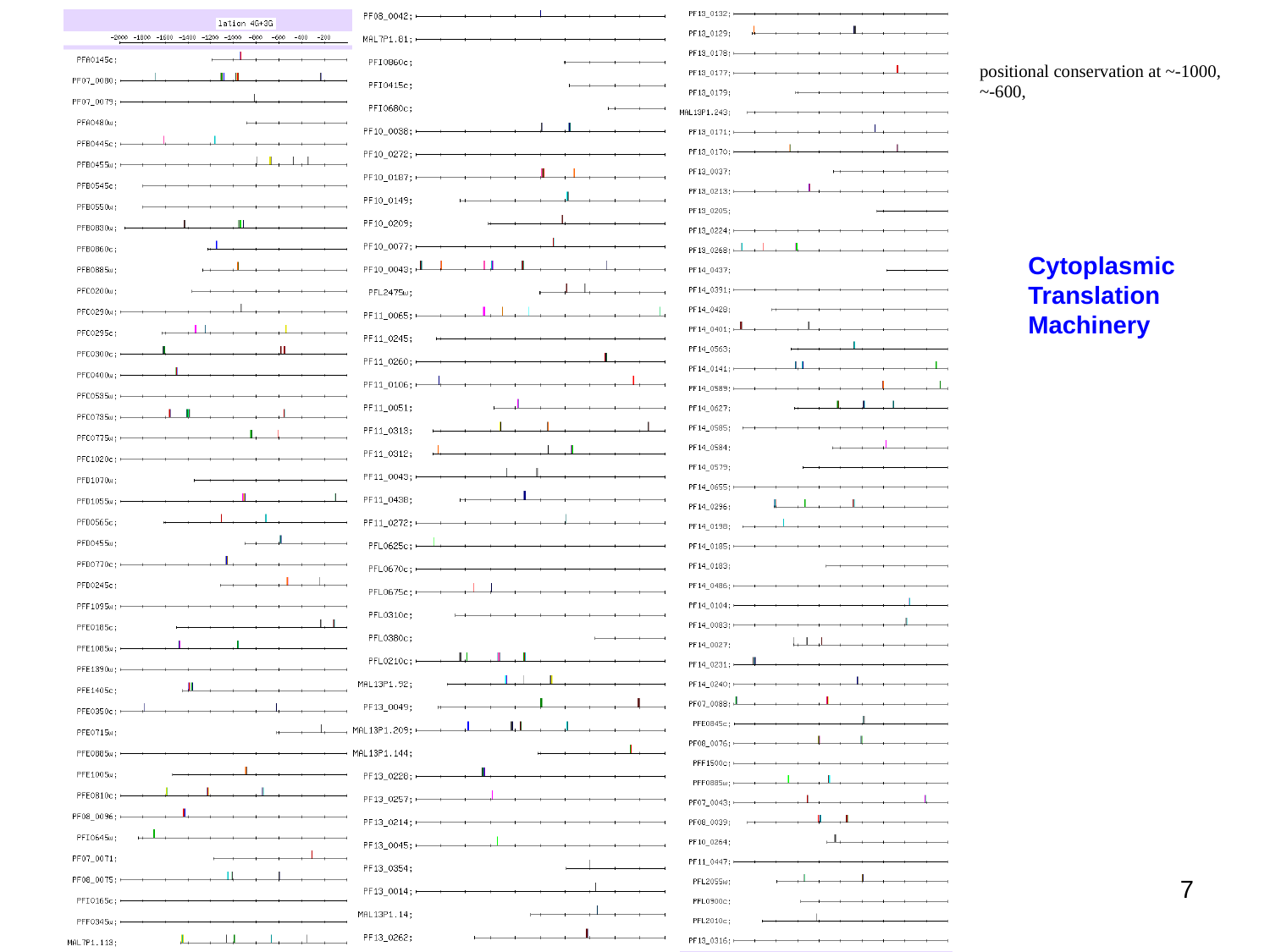

positional conservation at ~-1000,
~-600,
Cytoplasmic
Translation
Machinery
7

## Slide 8
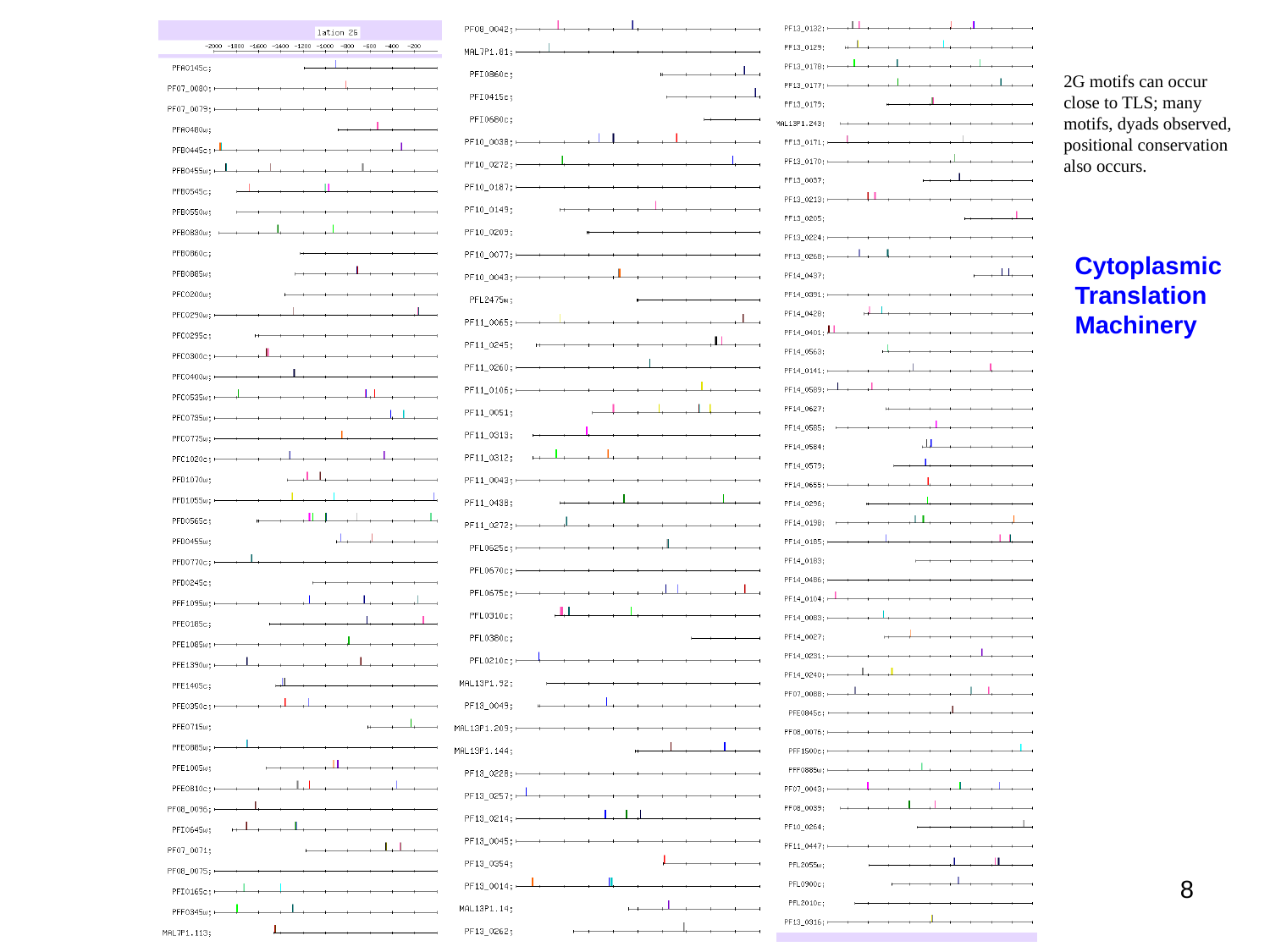

2G motifs can occur
close to TLS; many
motifs, dyads observed,
positional conservation
also occurs.
Cytoplasmic
Translation
Machinery
8

## Slide 9
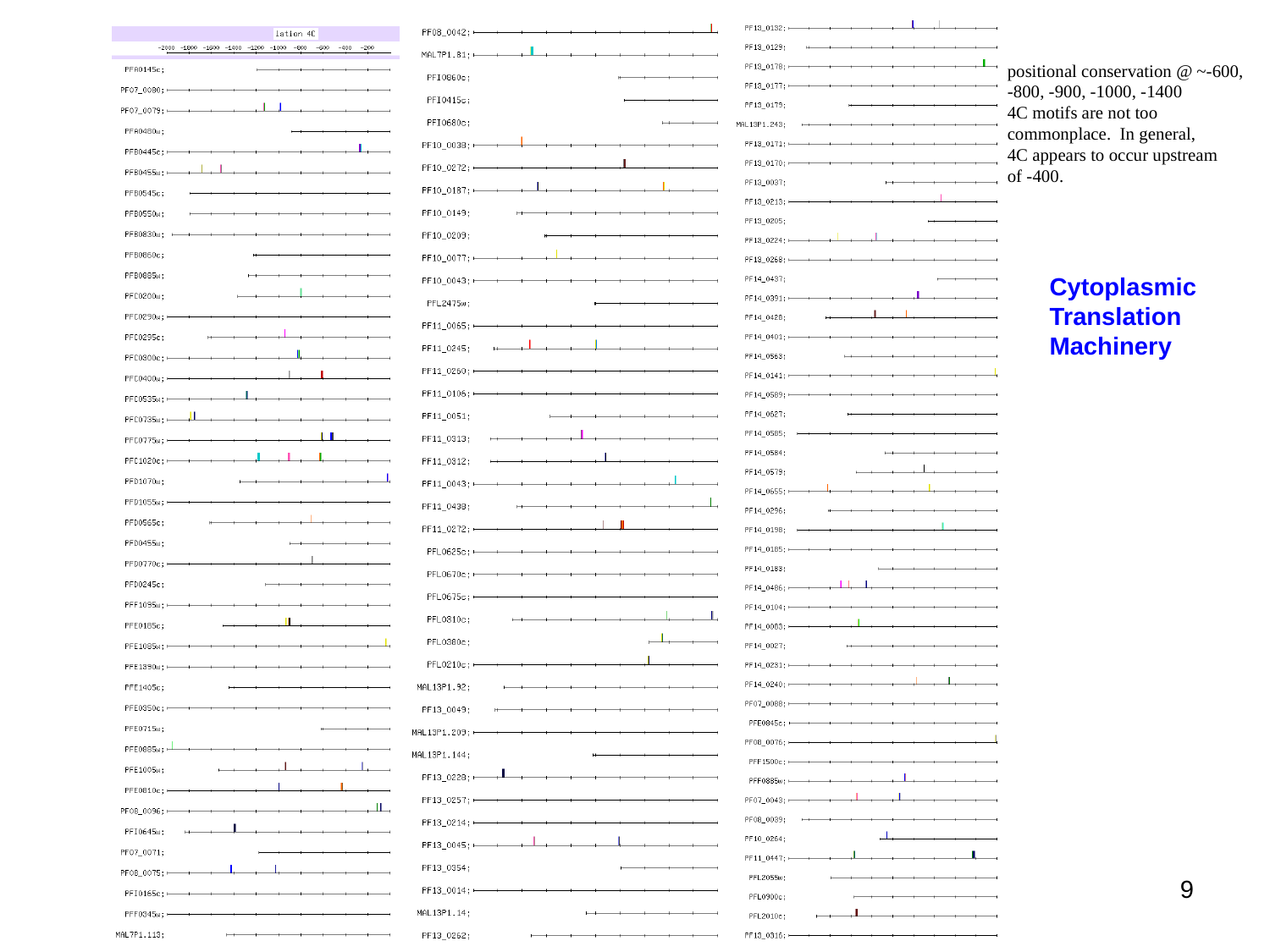

positional conservation @ ~-600,
-800, -900, -1000, -1400
4C motifs are not too
commonplace. In general,
4C appears to occur upstream
of -400.
Cytoplasmic
Translation
Machinery
9

## Slide 10
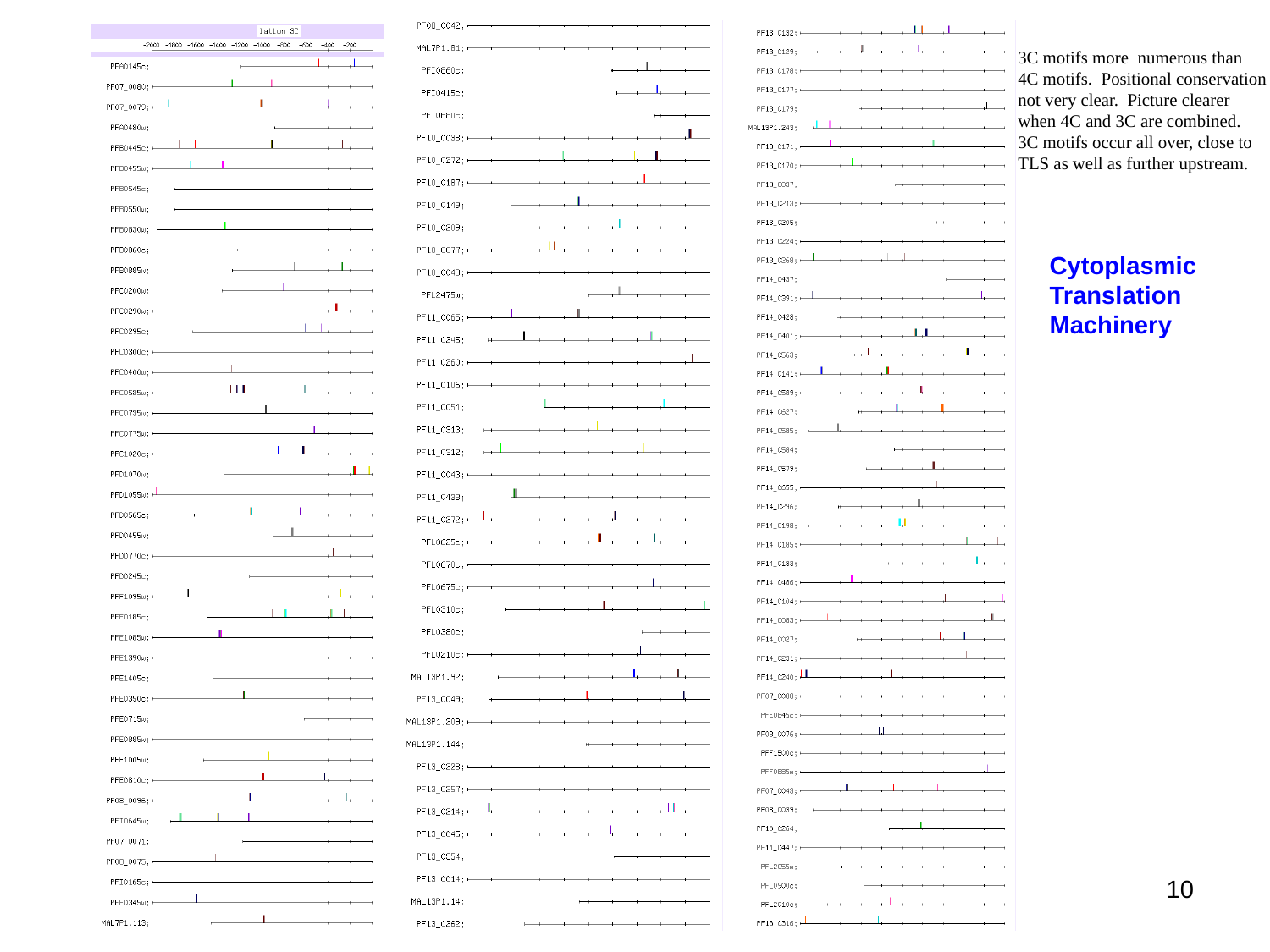

3C motifs more numerous than
4C motifs. Positional conservation
not very clear. Picture clearer
when 4C and 3C are combined.
3C motifs occur all over, close to
TLS as well as further upstream.
Cytoplasmic
Translation
Machinery
10

## Slide 11
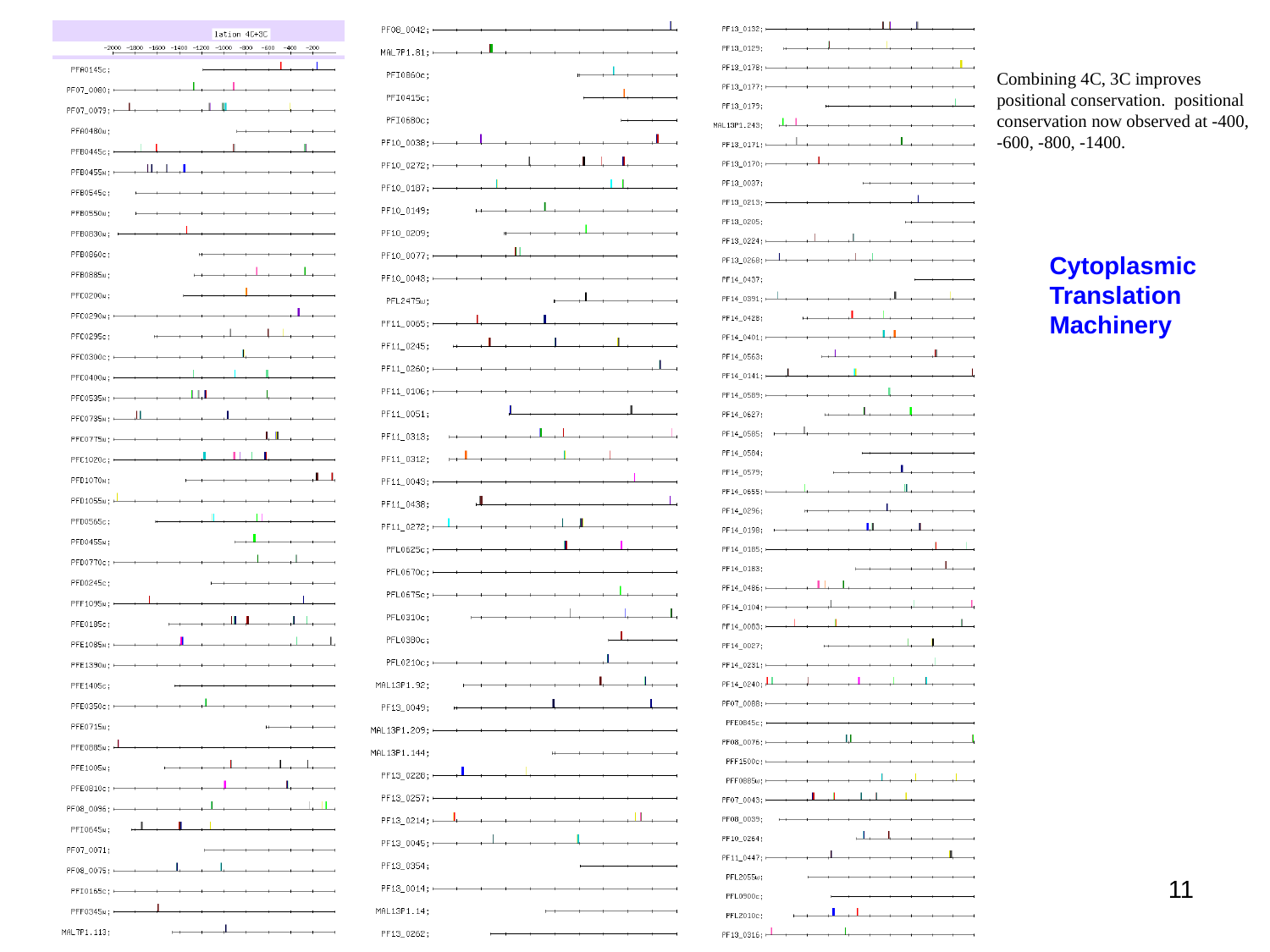

Combining 4C, 3C improves
positional conservation. positional
conservation now observed at -400,
-600, -800, -1400.
Cytoplasmic
Translation
Machinery
11

## Slide 12
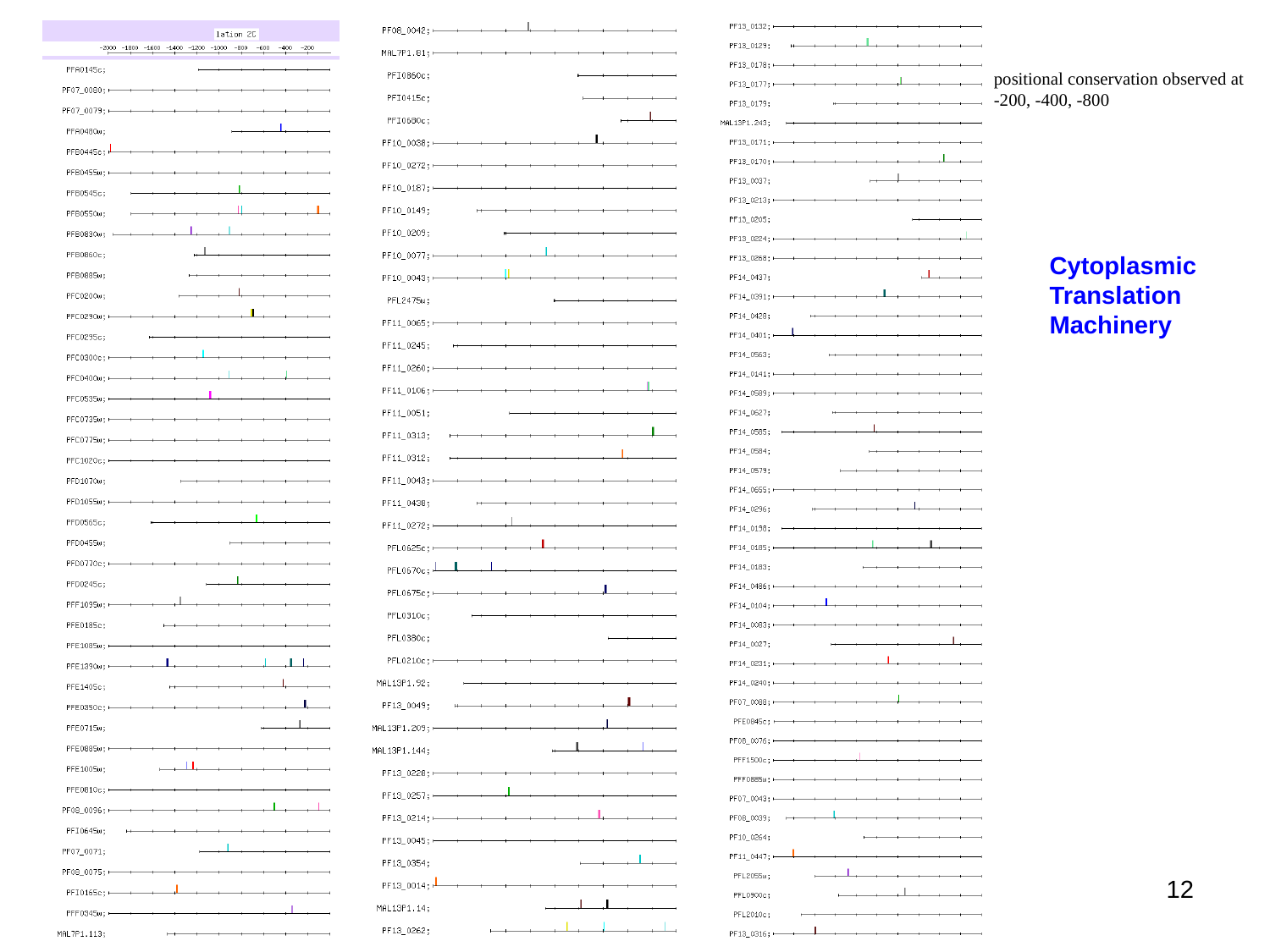

positional conservation observed at
-200, -400, -800
Cytoplasmic
Translation
Machinery
12

## Slide 13
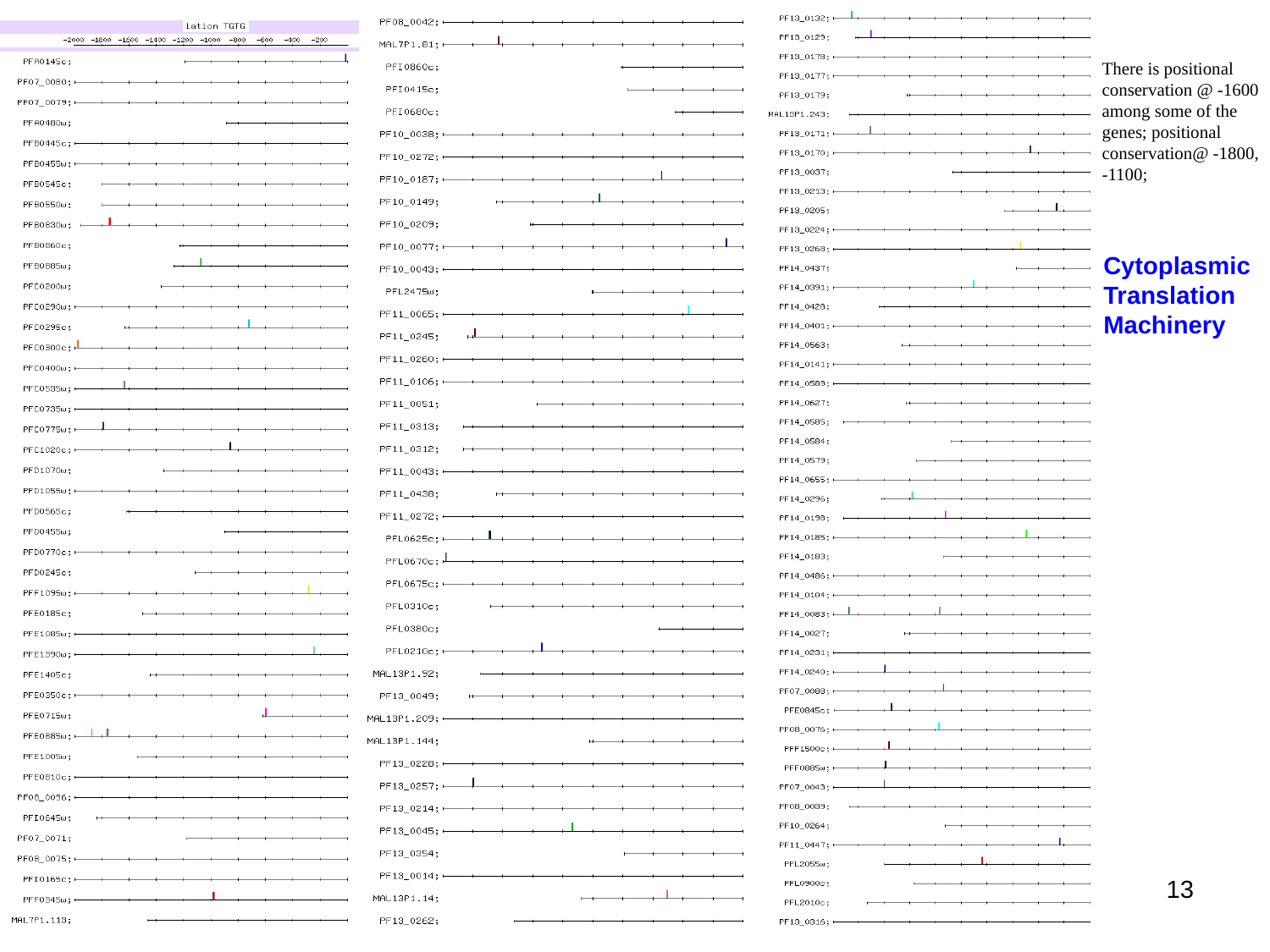

There is positional
conservation @ -1600
among some of the
genes; positional
conservation@ -1800,
-1100;
Cytoplasmic
Translation
Machinery
13

## Slide 14
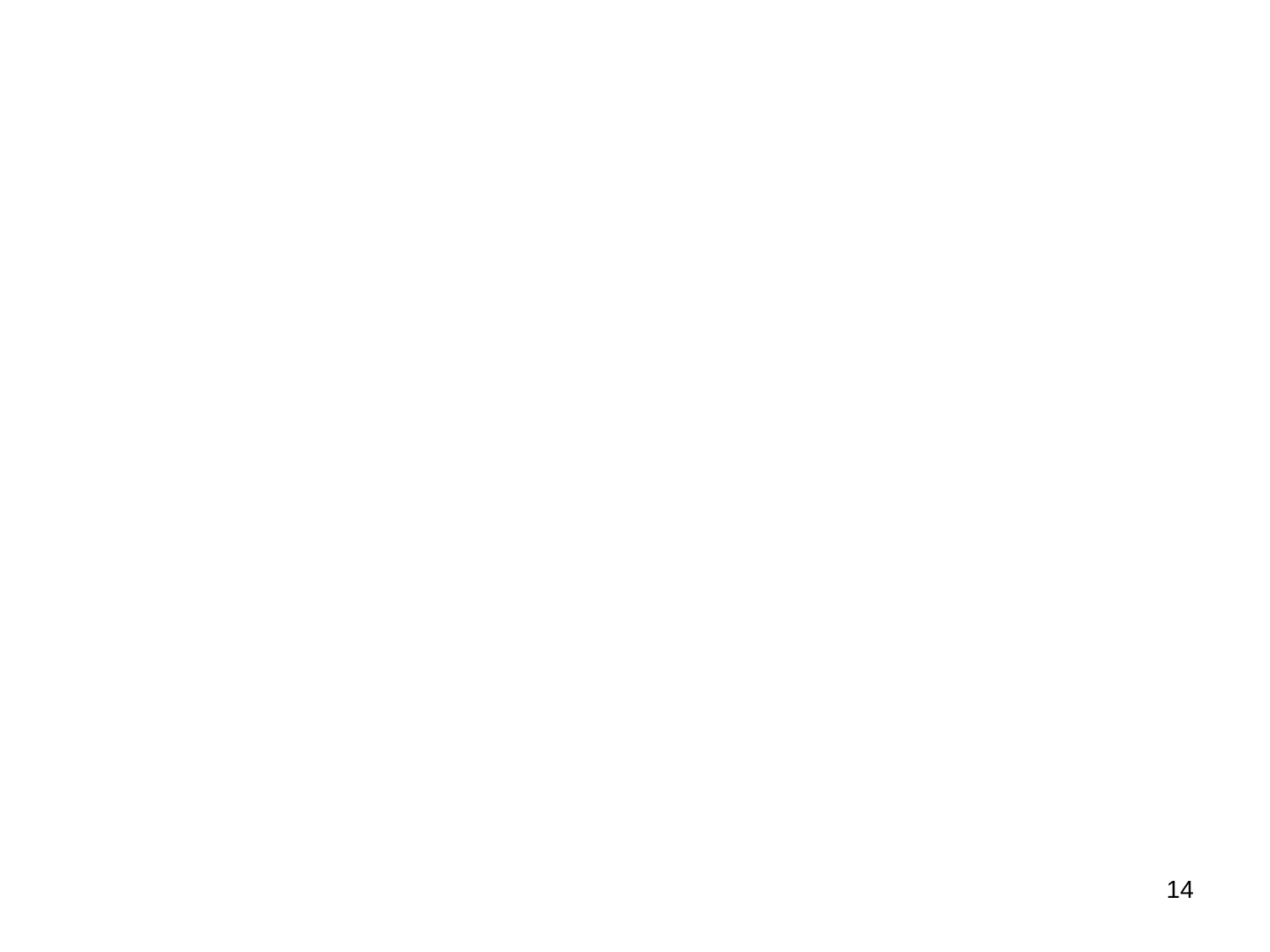

14

## Slide 15
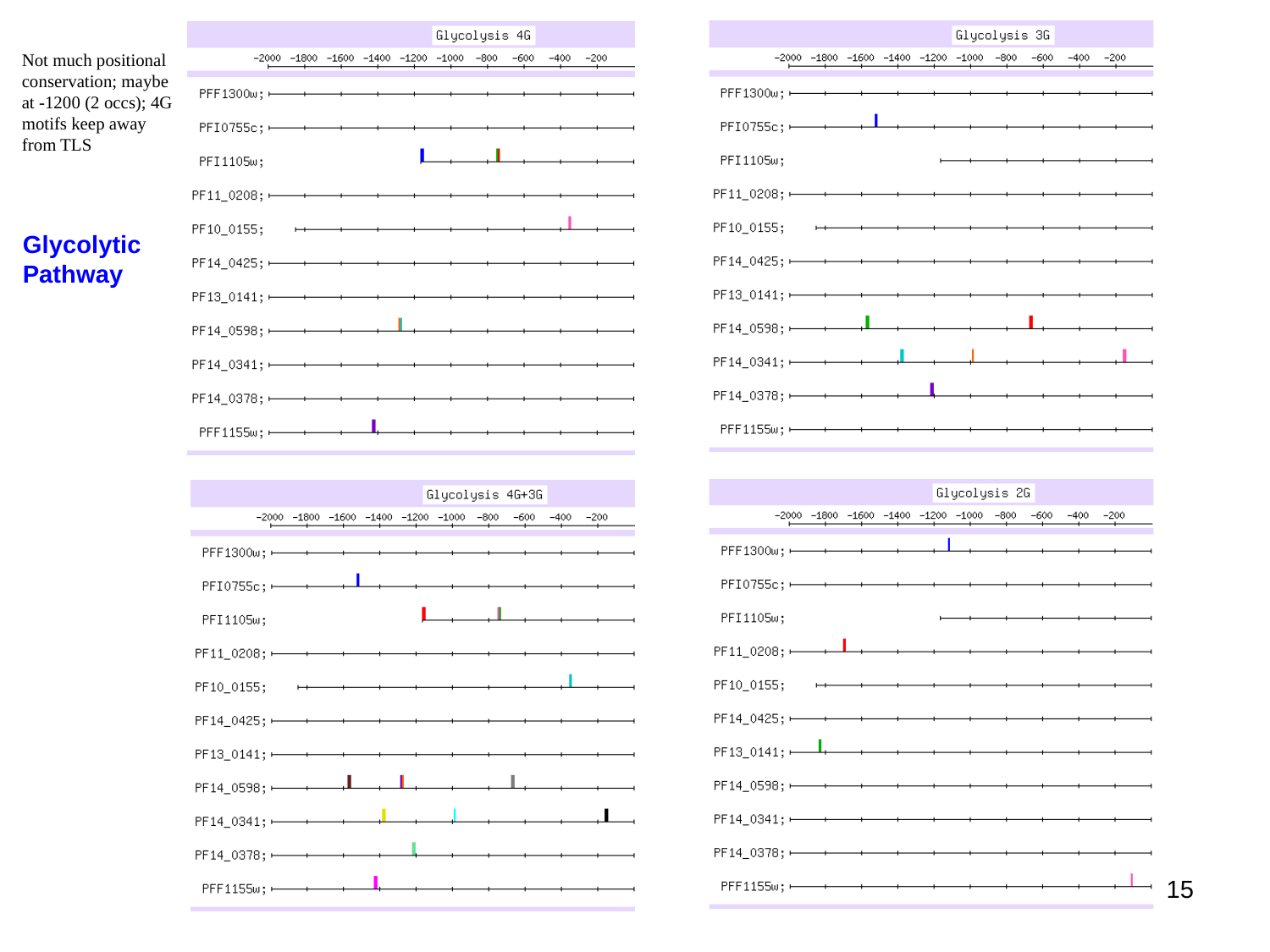

Not much positional
conservation; maybe
at -1200 (2 occs); 4G
motifs keep away
from TLS
Glycolytic
Pathway
15

## Slide 16
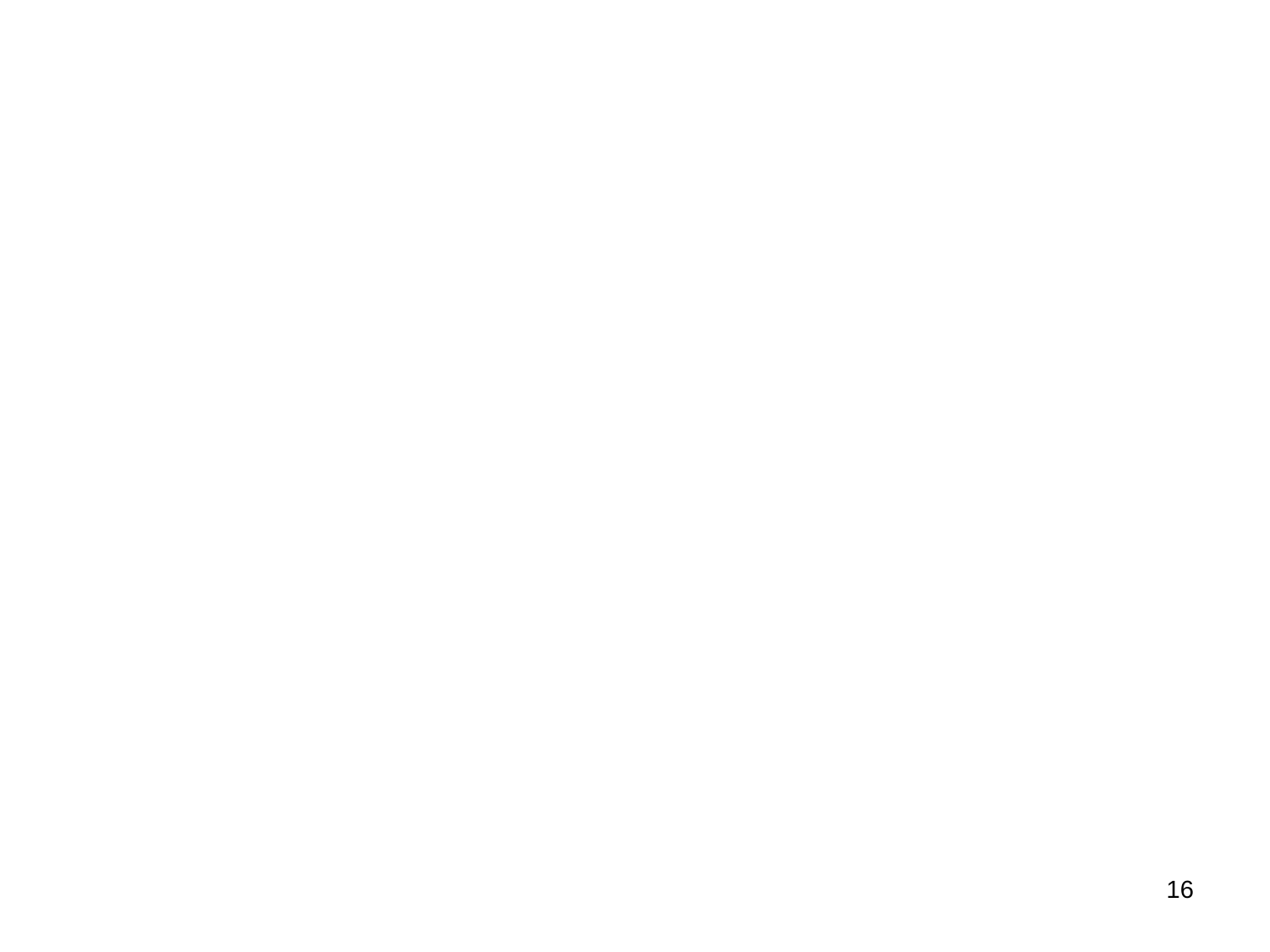

16

## Slide 17
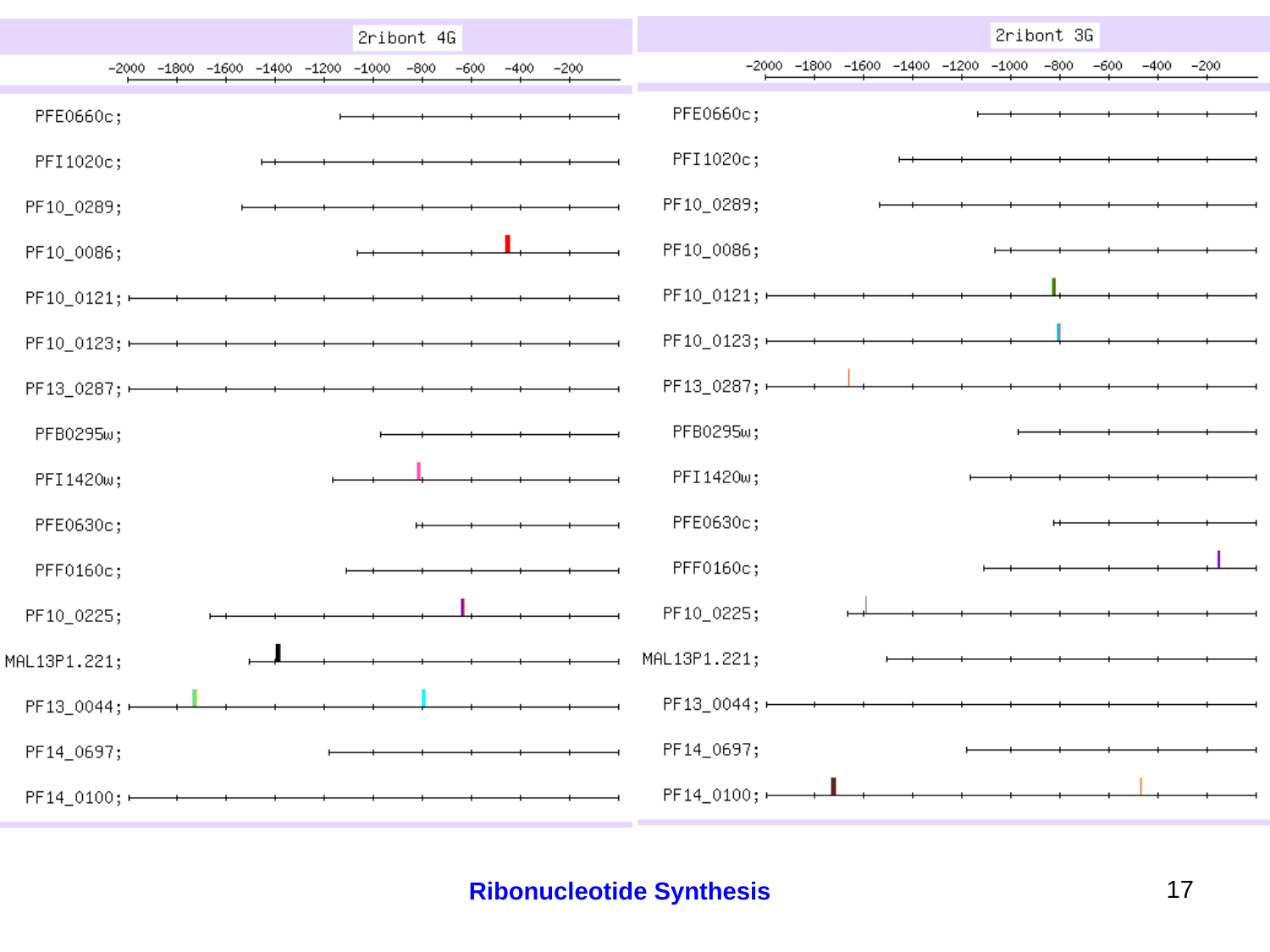

17
Ribonucleotide Synthesis

## Slide 18
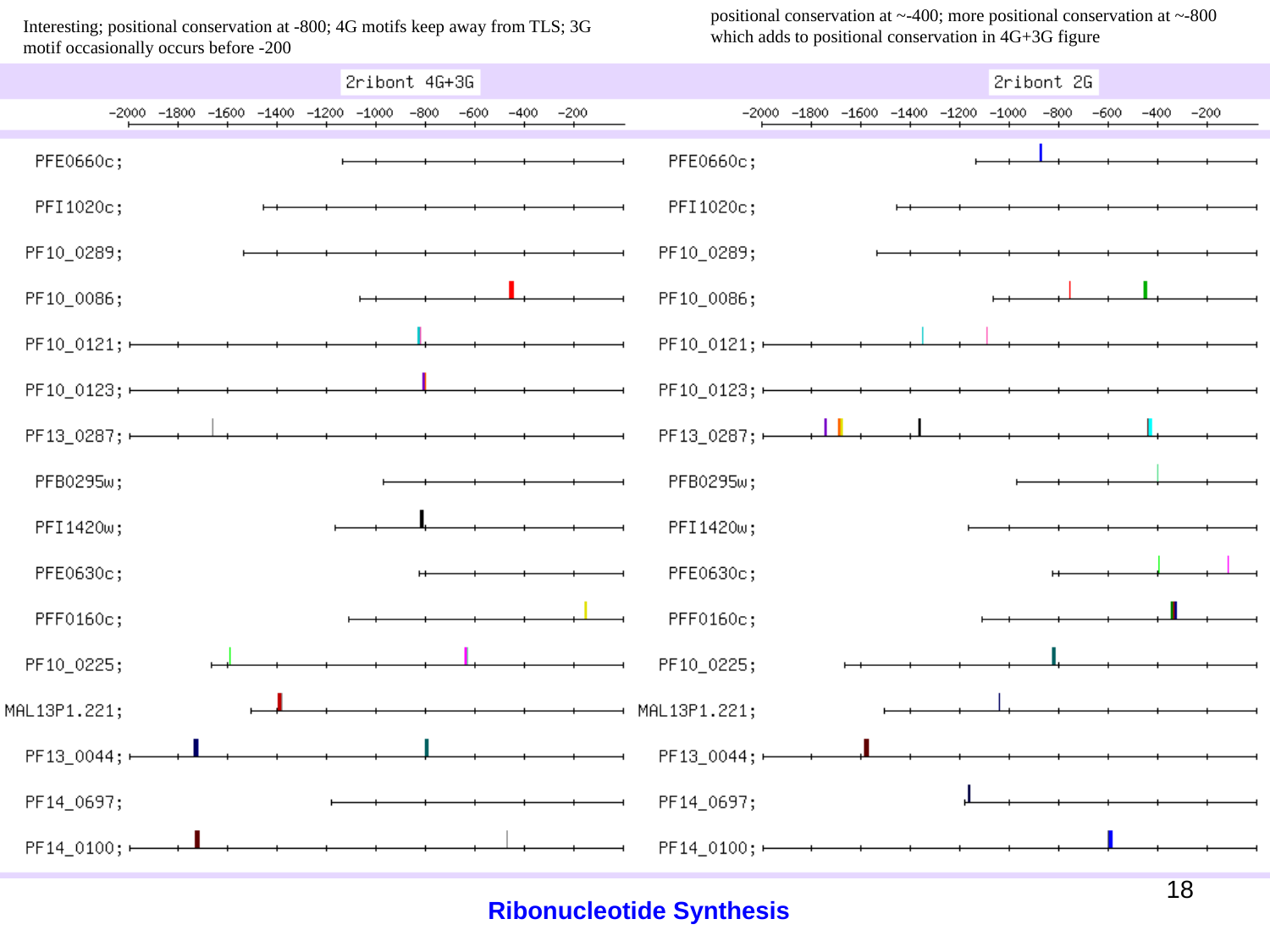

positional conservation at ~-400; more positional conservation at ~-800
which adds to positional conservation in 4G+3G figure
Interesting; positional conservation at -800; 4G motifs keep away from TLS; 3G
motif occasionally occurs before -200
18
Ribonucleotide Synthesis

## Slide 19
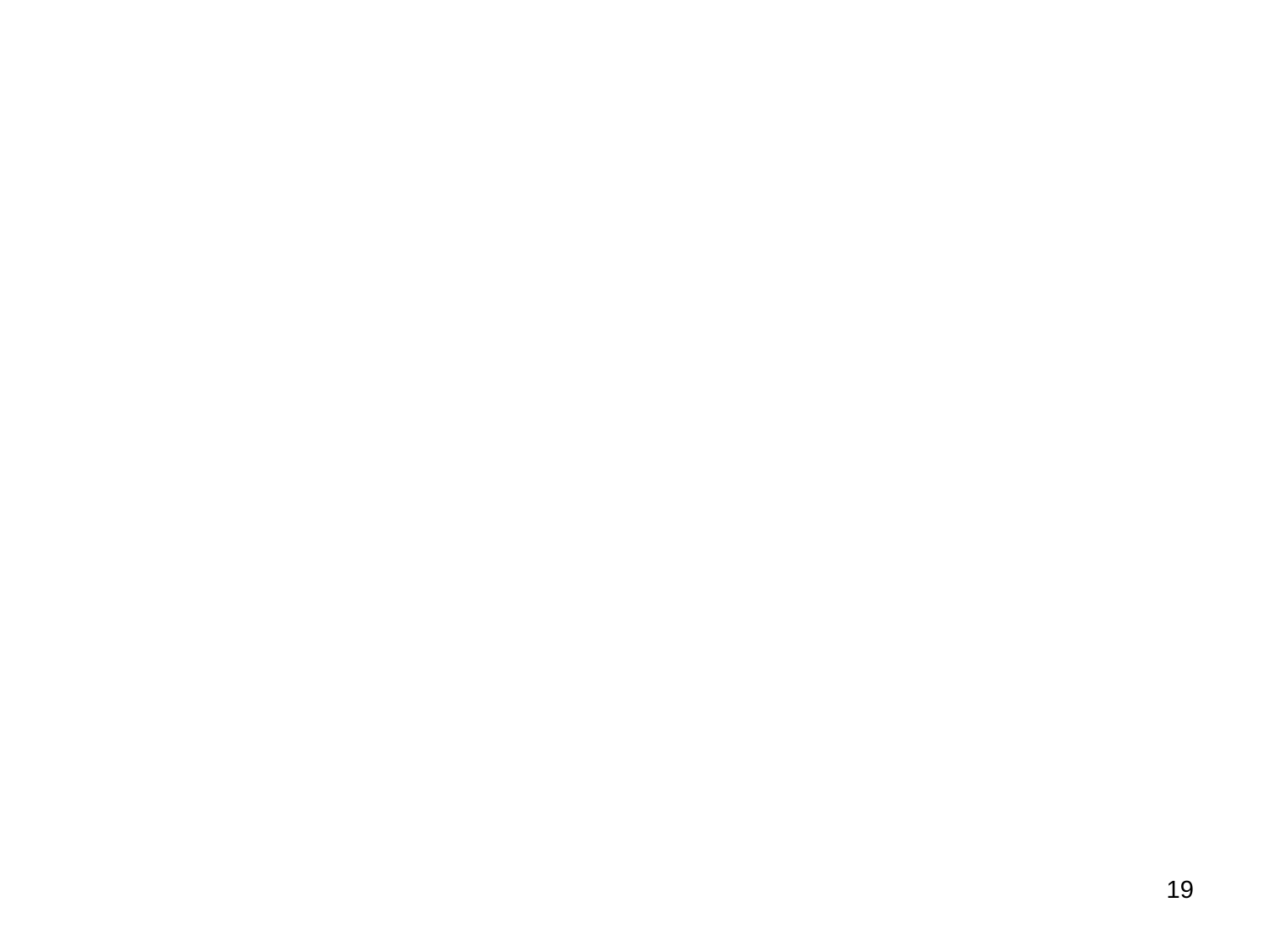

19

## Slide 20
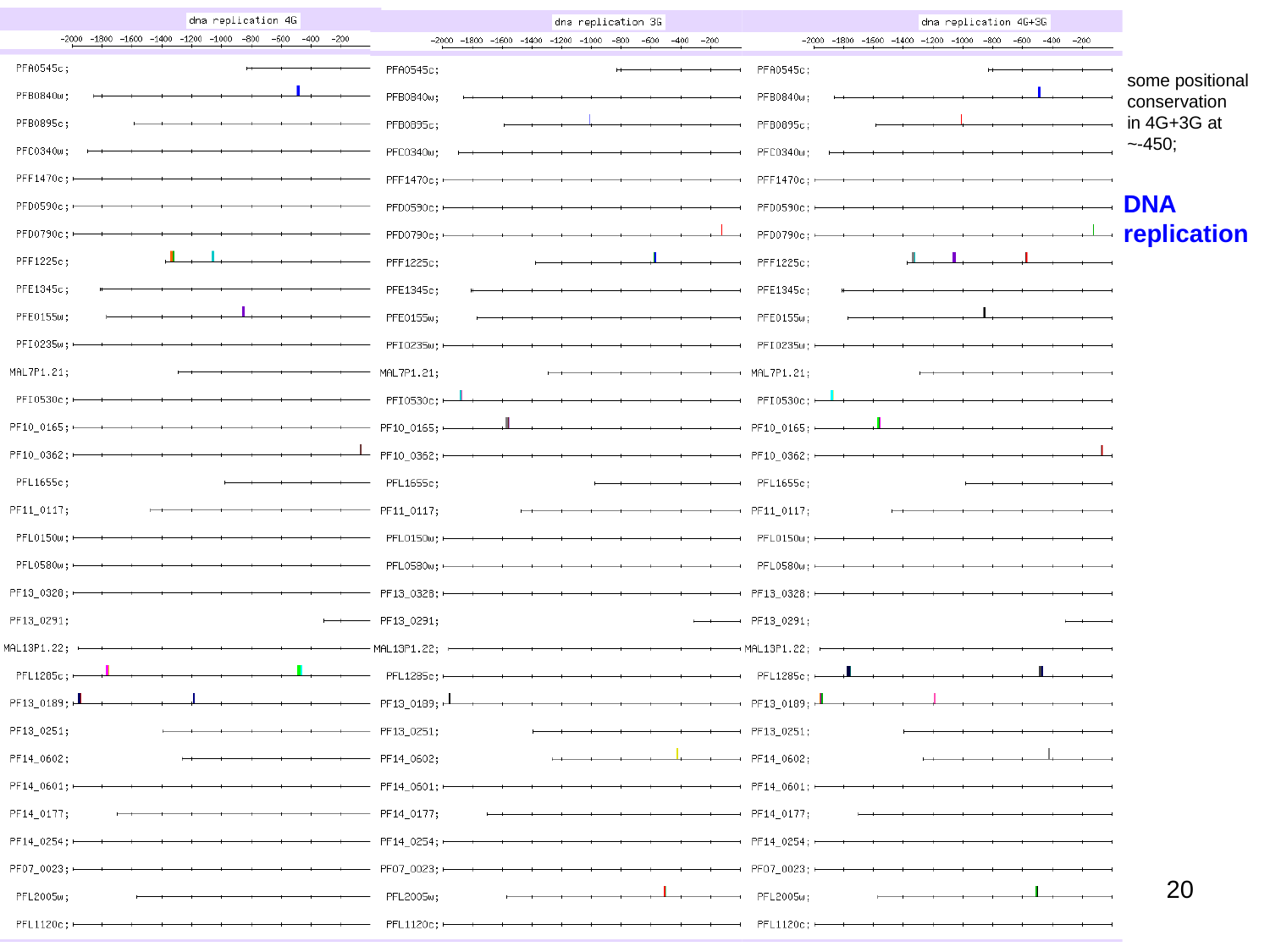

some positional
conservation
in 4G+3G at
~-450;
DNA
replication
20

## Slide 21
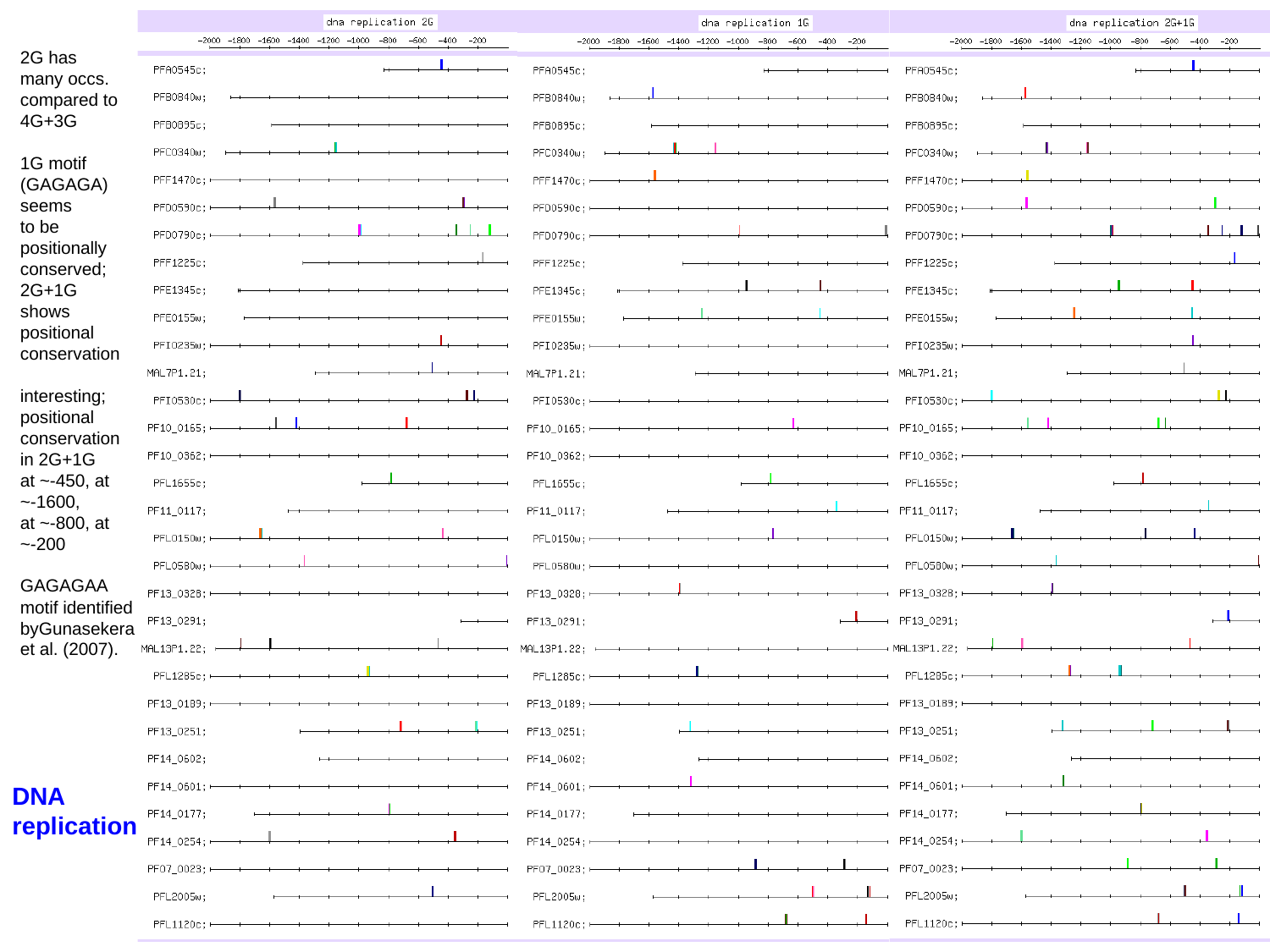

2G has
many occs.
compared to
4G+3G
1G motif
(GAGAGA)
seems
to be
positionally
conserved;
2G+1G
shows
positional
conservation
interesting;
positional
conservation
in 2G+1G
at ~-450, at
~-1600,
at ~-800, at
~-200
GAGAGAA
motif identified
byGunasekera
et al. (2007).
DNA
replication
21

## Slide 22
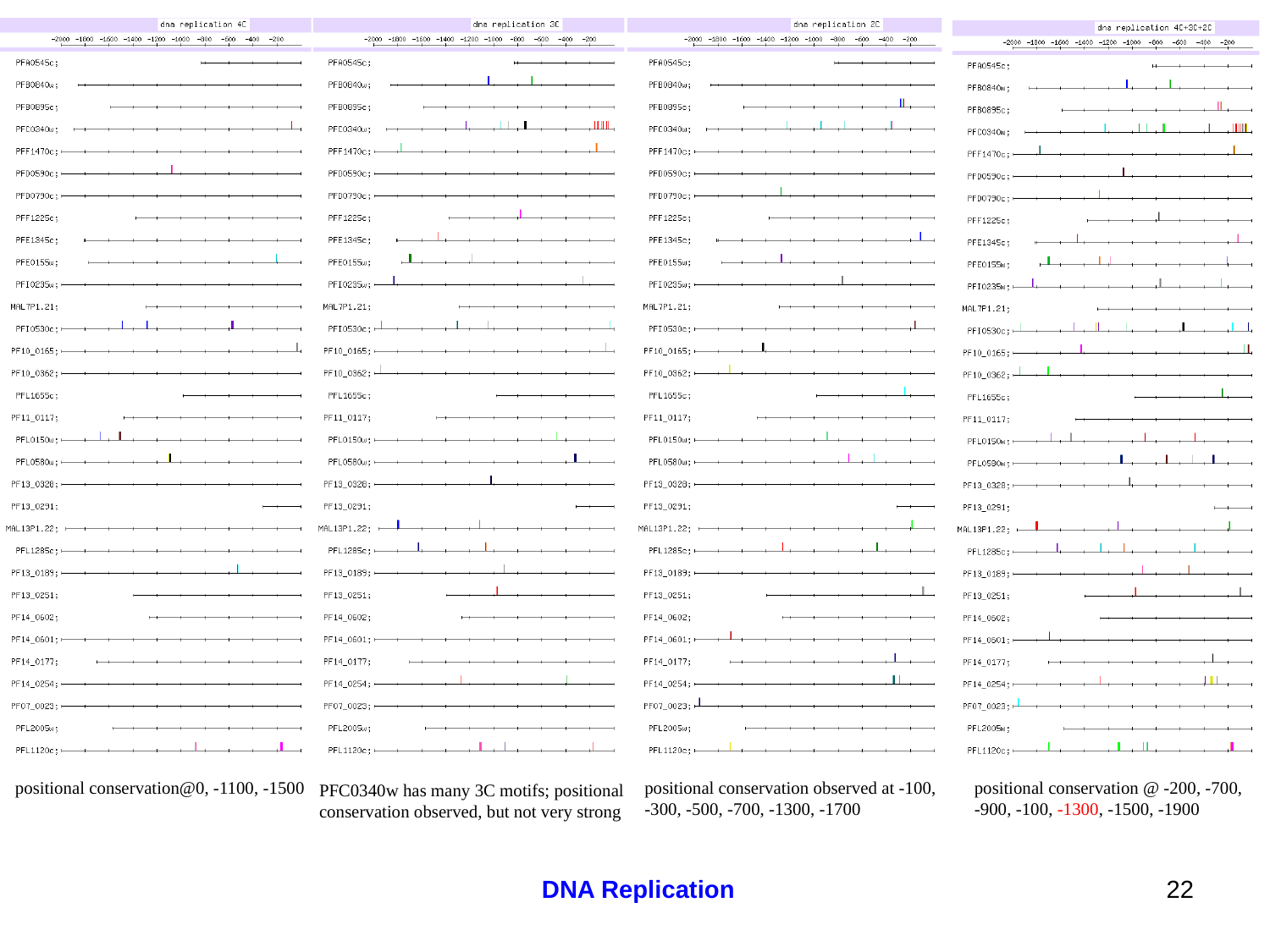

positional conservation@0, -1100, -1500
positional conservation observed at -100,
-300, -500, -700, -1300, -1700
positional conservation @ -200, -700,
-900, -100, -1300, -1500, -1900
PFC0340w has many 3C motifs; positional
conservation observed, but not very strong
22
DNA Replication

## Slide 23
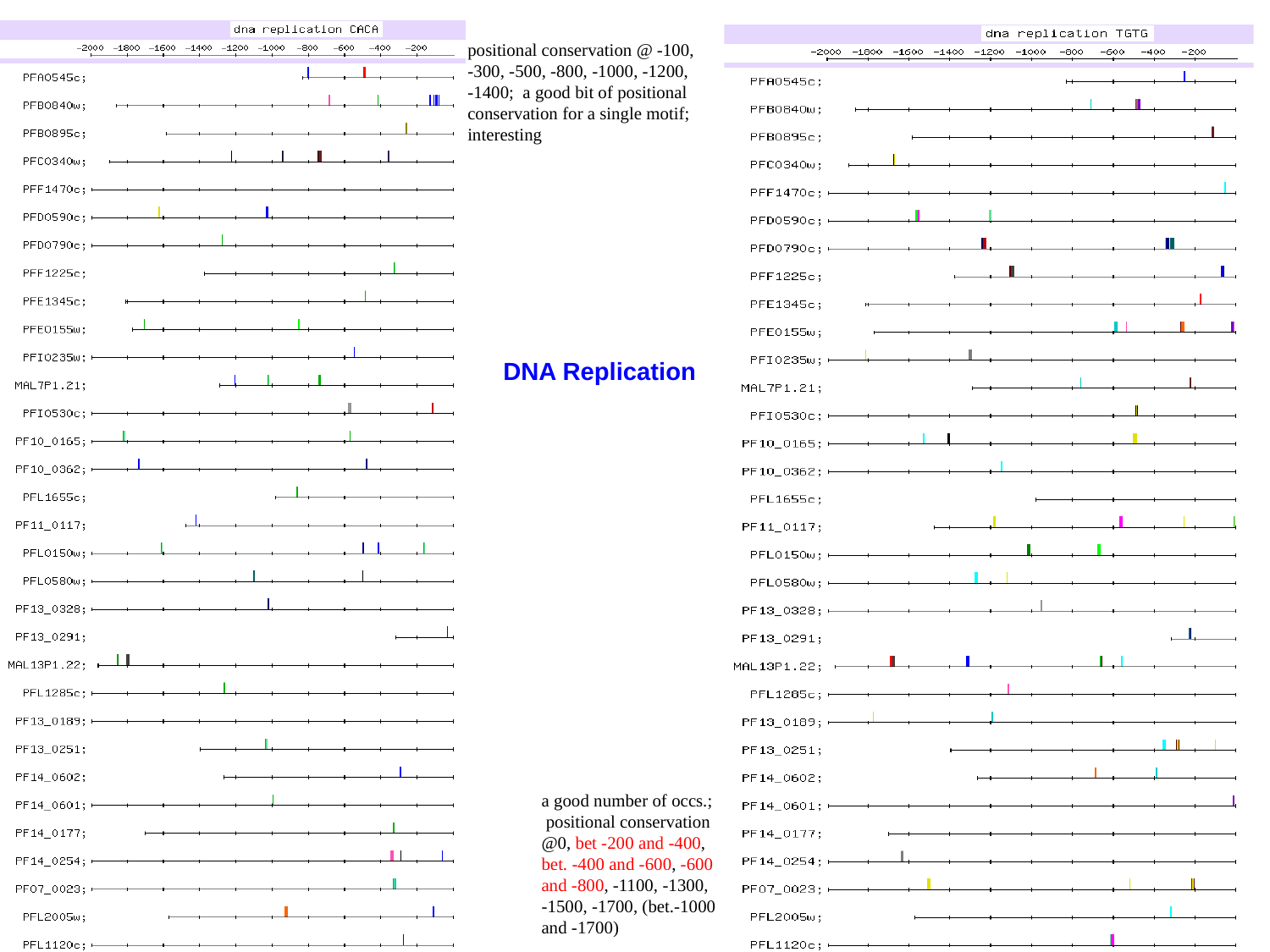

positional conservation @ -100,
-300, -500, -800, -1000, -1200,
-1400; a good bit of positional
conservation for a single motif;
interesting
DNA Replication
a good number of occs.;
 positional conservation
@0, bet -200 and -400,
bet. -400 and -600, -600
and -800, -1100, -1300,
-1500, -1700, (bet.-1000
and -1700)
23

## Slide 24
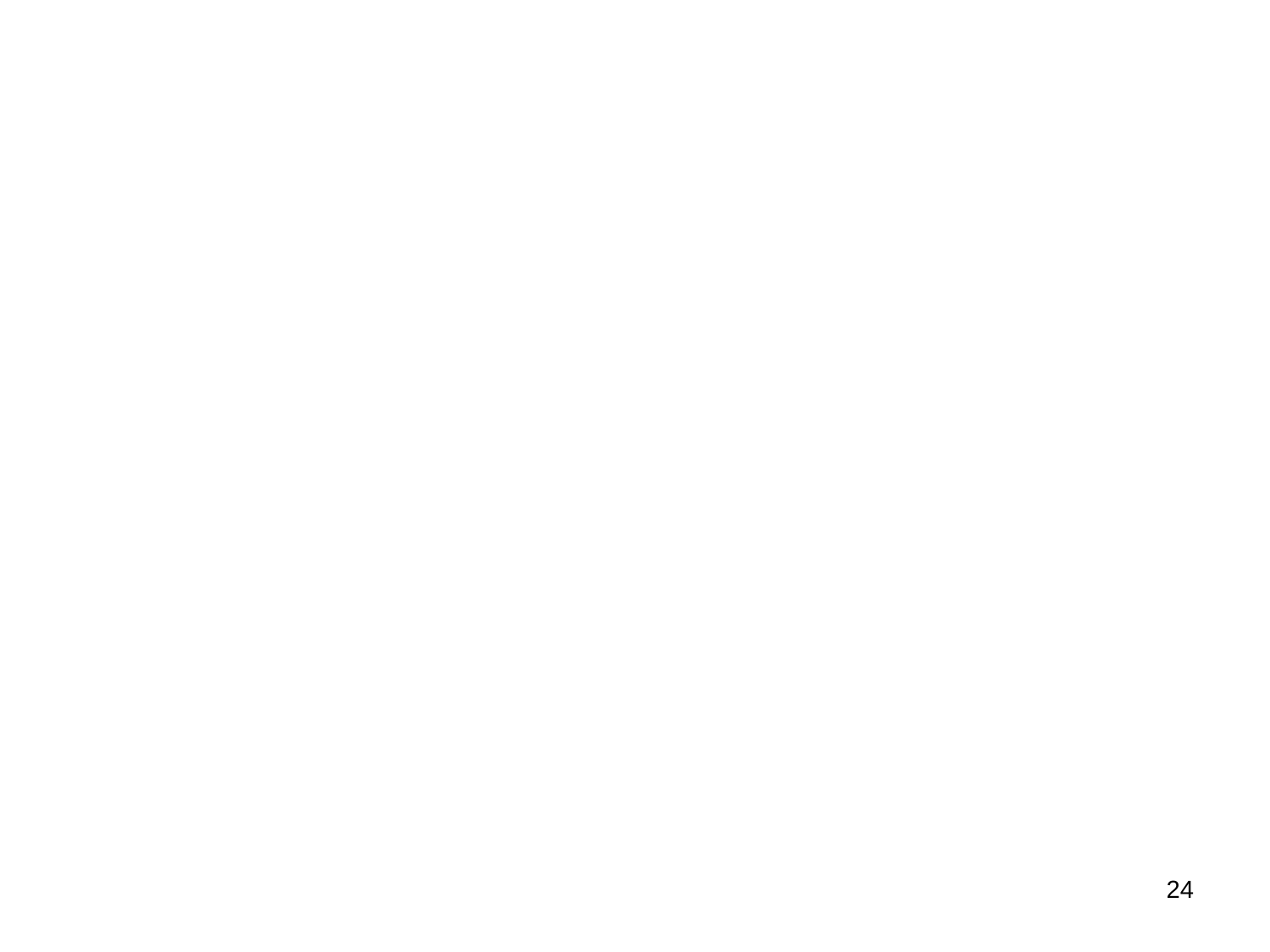

24

## Slide 25
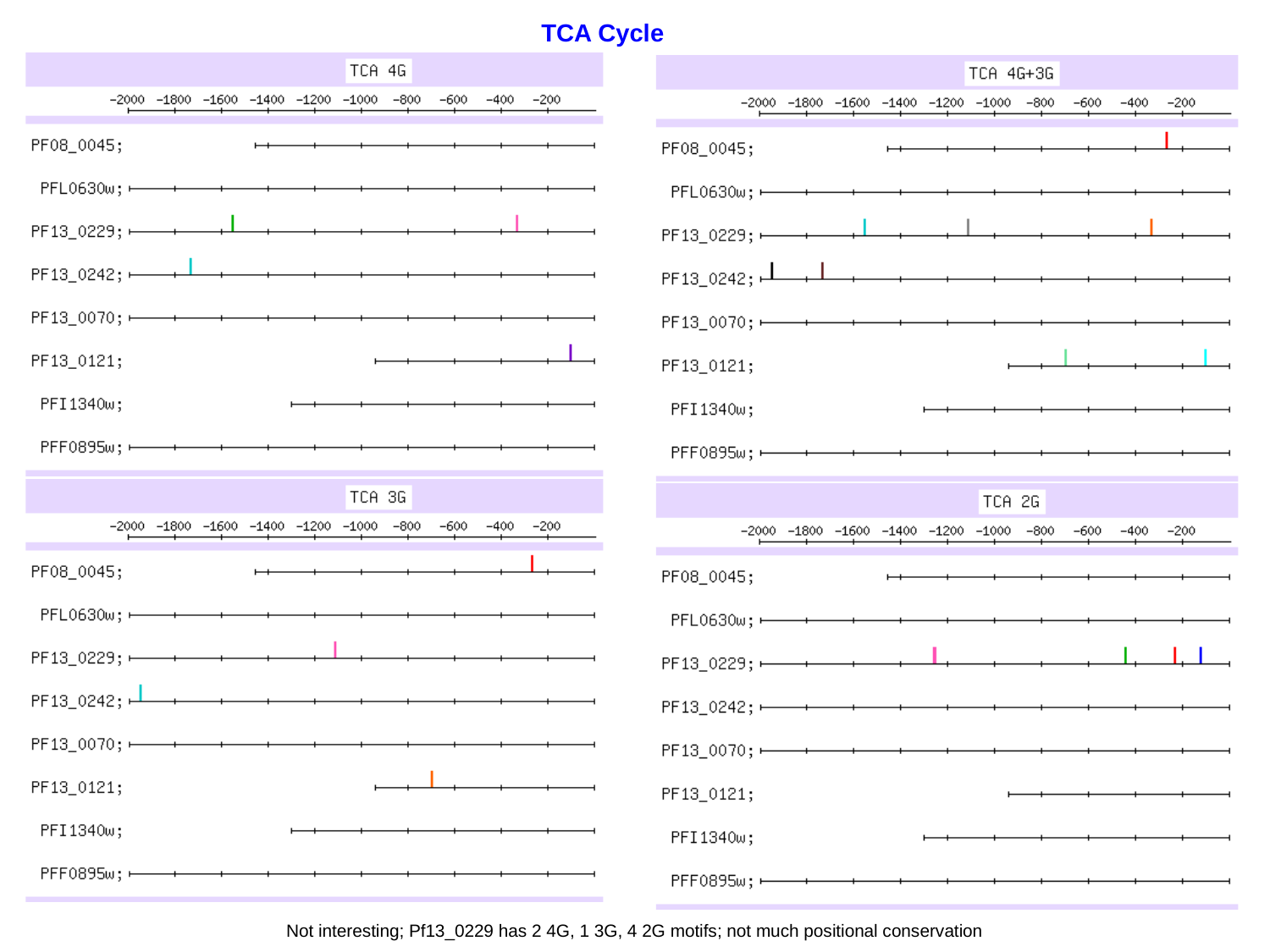

TCA Cycle
25
Not interesting; Pf13_0229 has 2 4G, 1 3G, 4 2G motifs; not much positional conservation

## Slide 26
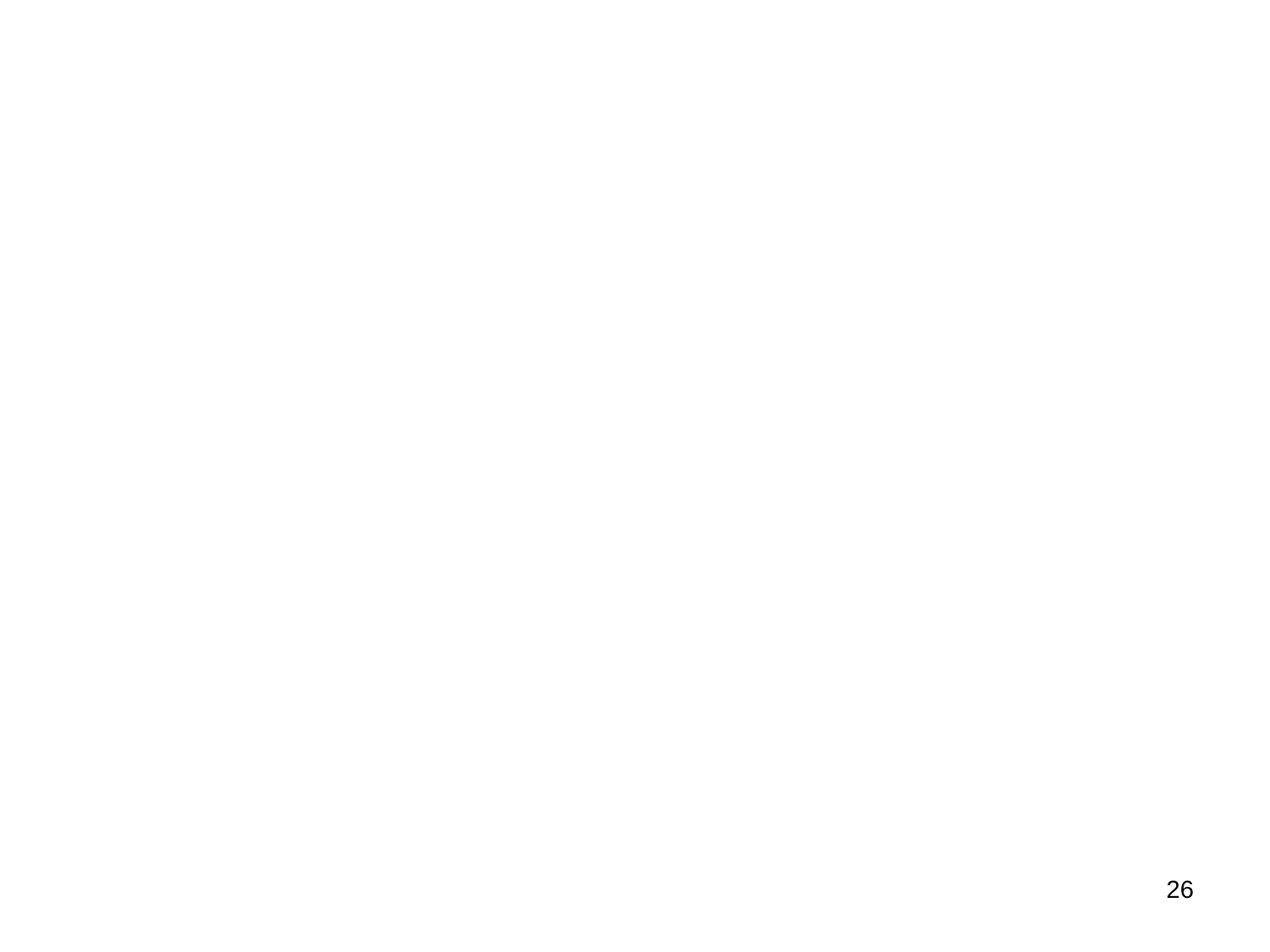

26

## Slide 27
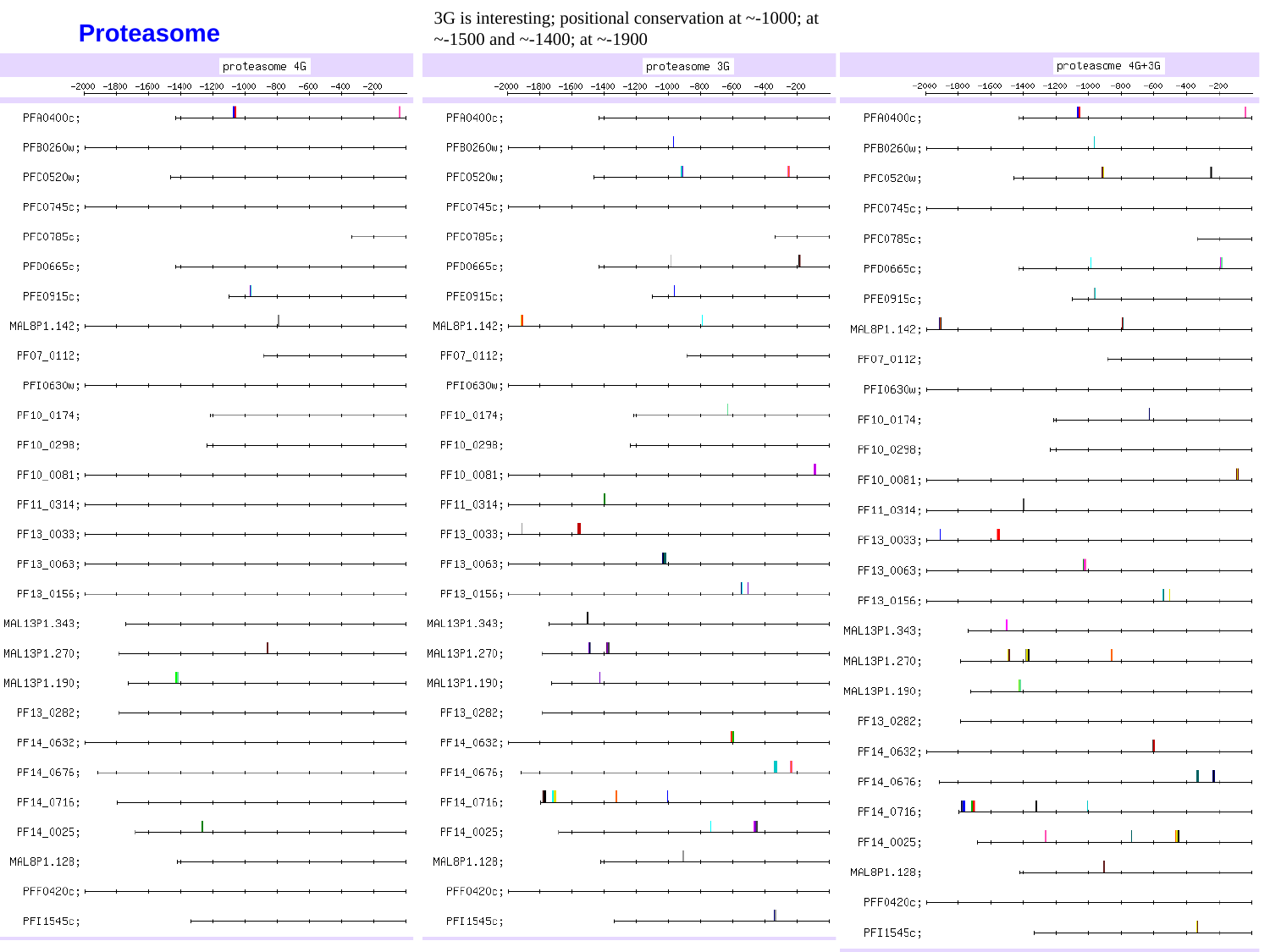

3G is interesting; positional conservation at ~-1000; at
~-1500 and ~-1400; at ~-1900
Proteasome
27

## Slide 28
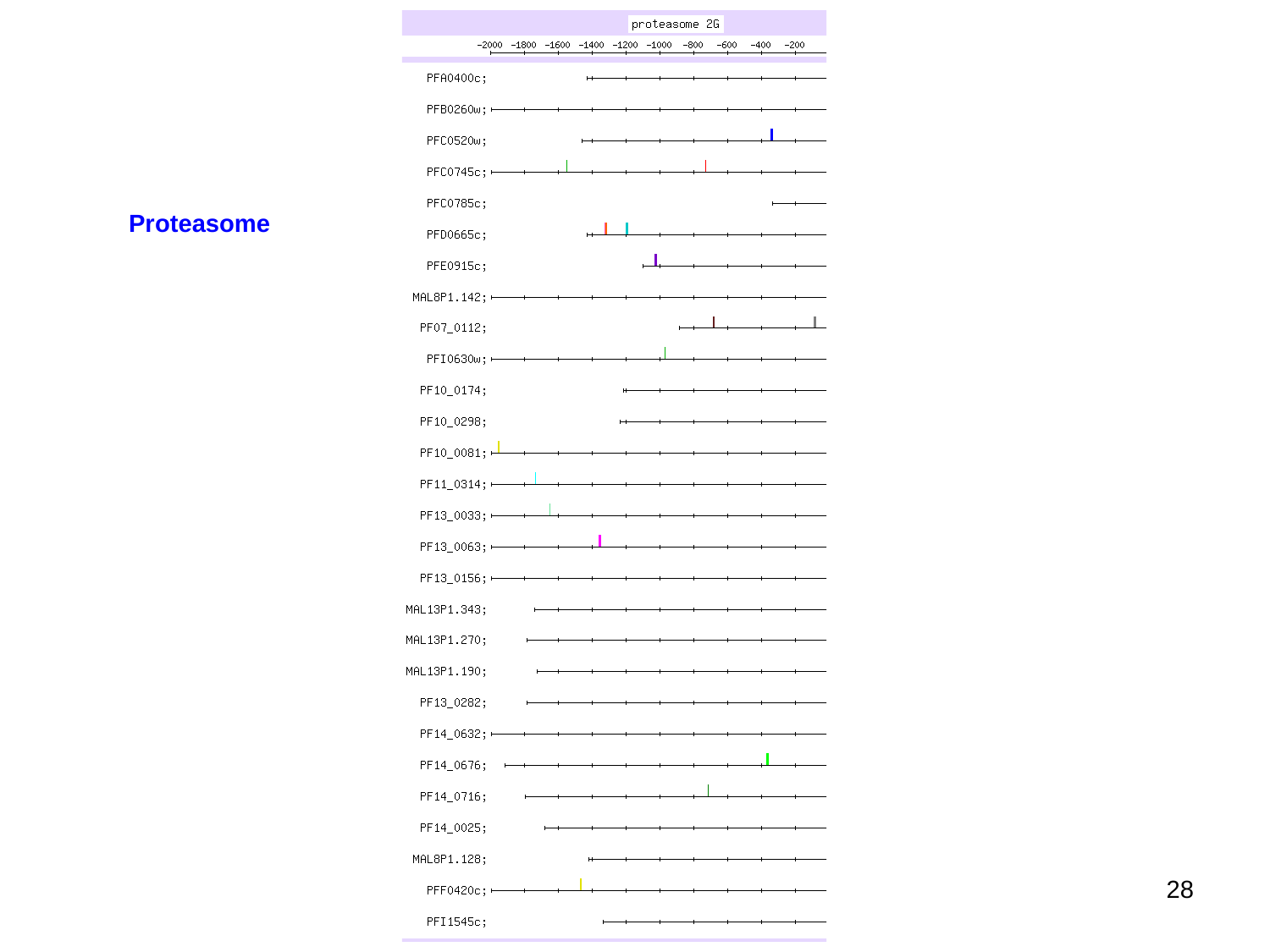

Proteasome
28

## Slide 29
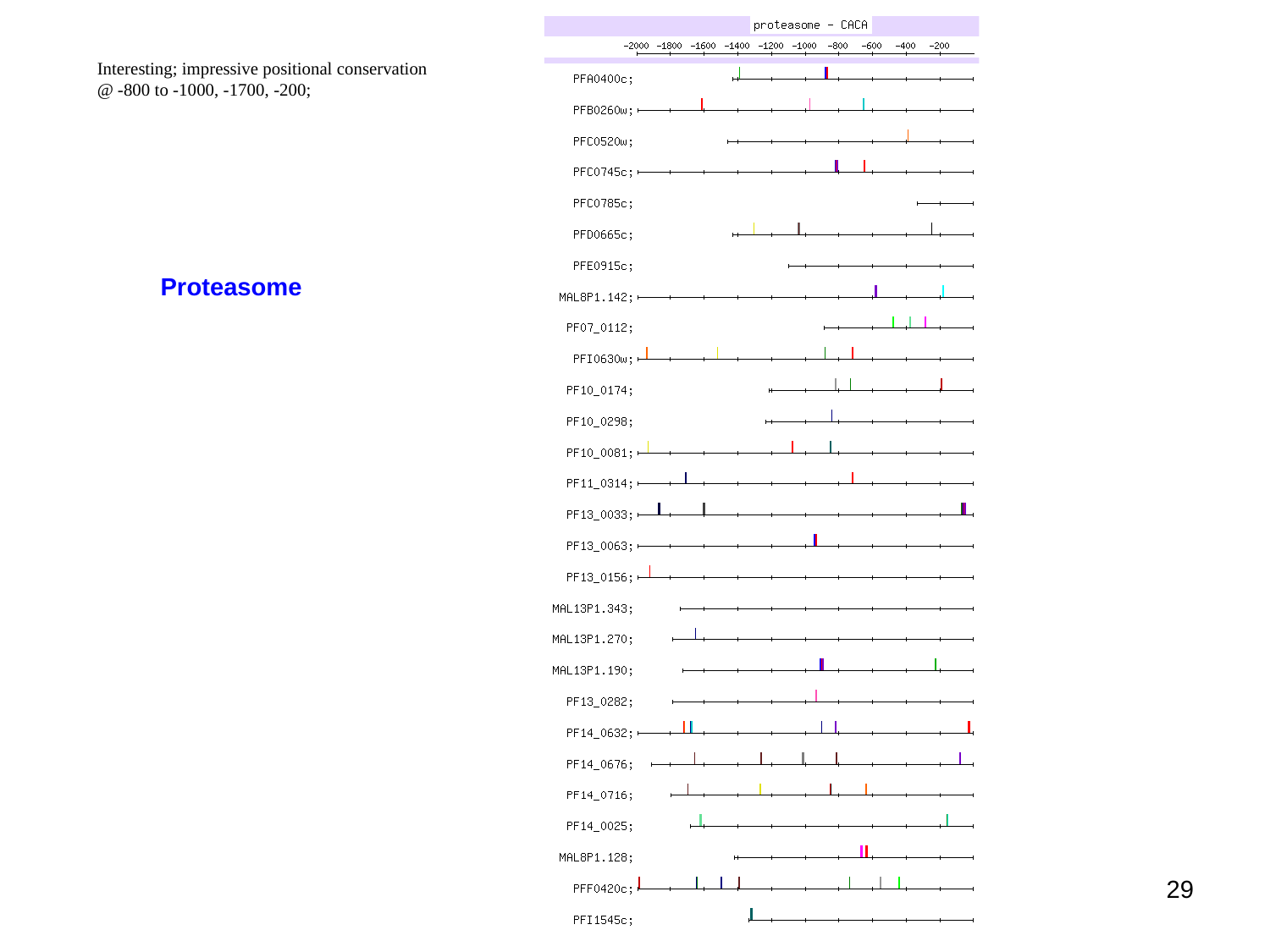

Interesting; impressive positional conservation
@ -800 to -1000, -1700, -200;
Proteasome
29

## Slide 30
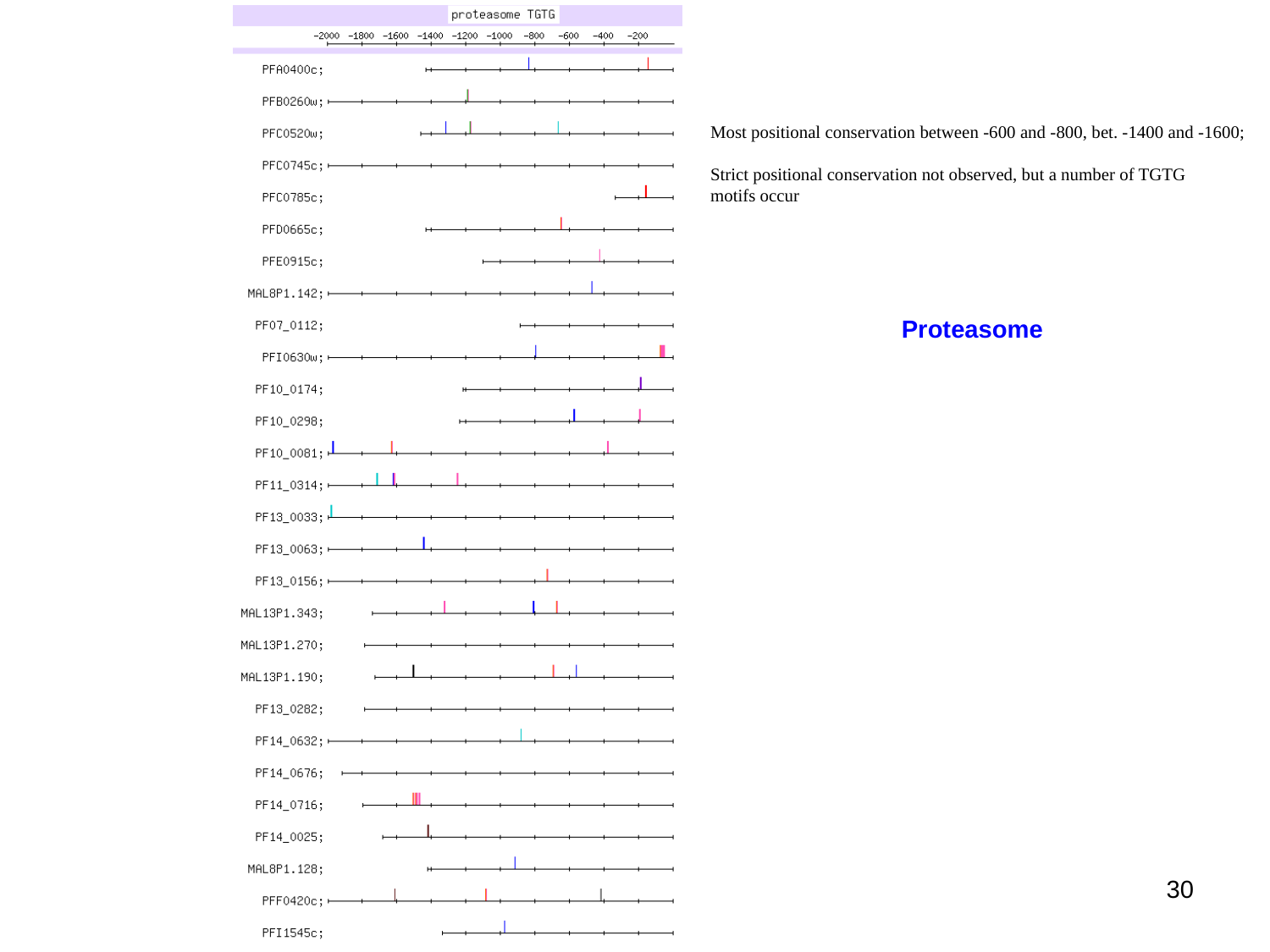

Most positional conservation between -600 and -800, bet. -1400 and -1600;
Strict positional conservation not observed, but a number of TGTG
motifs occur
Proteasome
30

## Slide 31
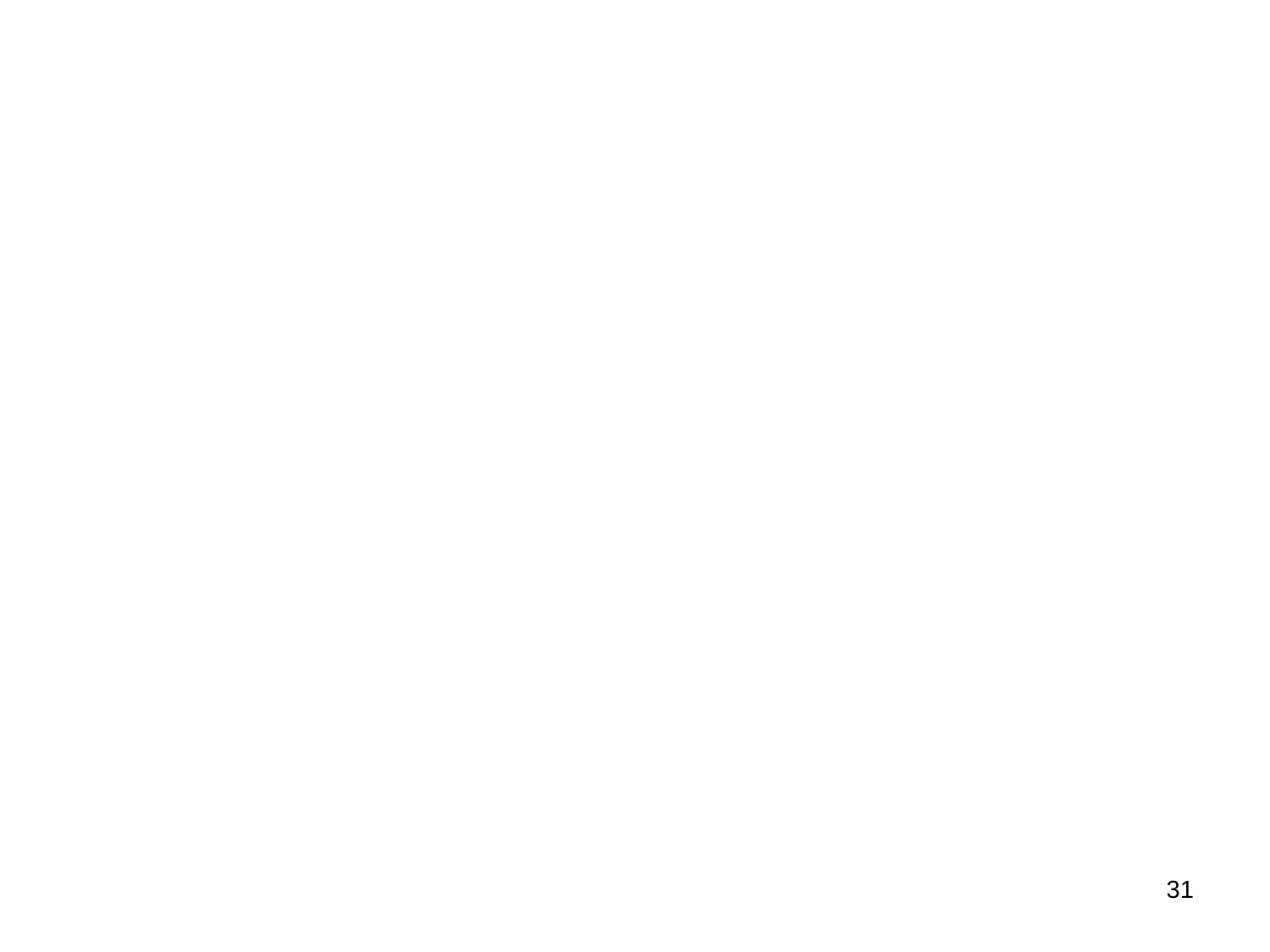

31

## Slide 32
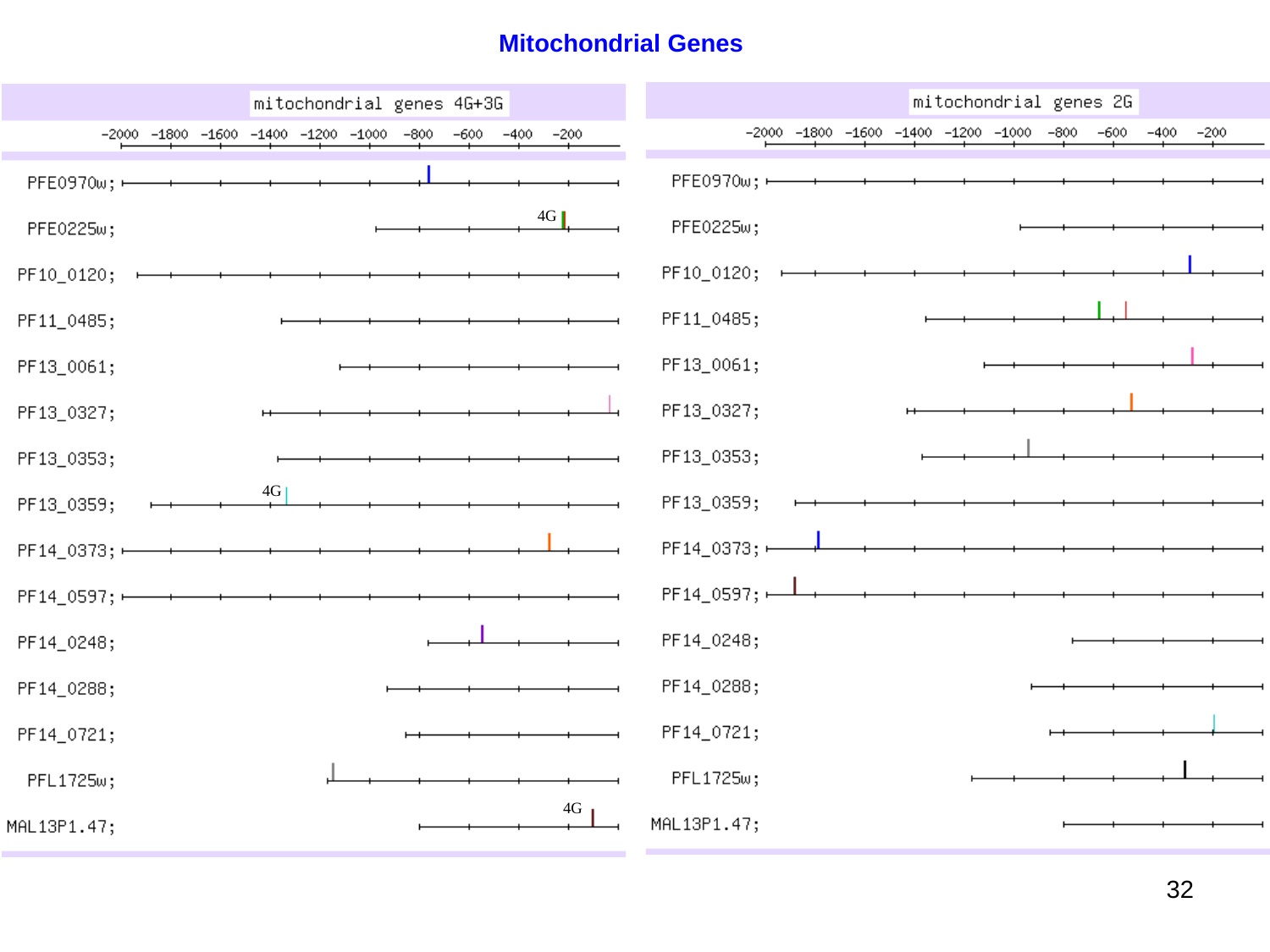

Mitochondrial Genes
4G
4G
4G
32

## Slide 33
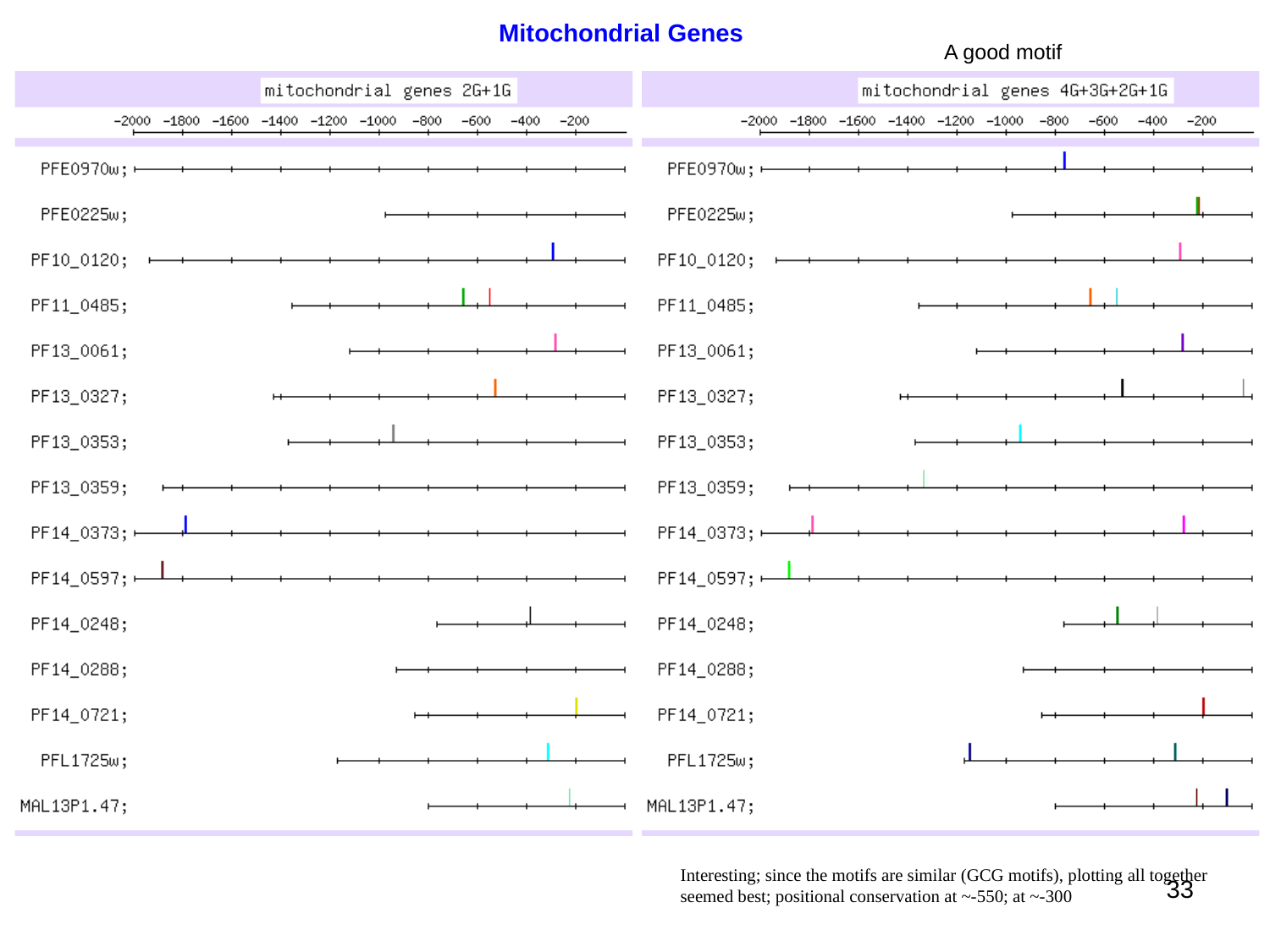

Mitochondrial Genes
A good motif
Interesting; since the motifs are similar (GCG motifs), plotting all together
seemed best; positional conservation at ~-550; at ~-300
33

## Slide 34
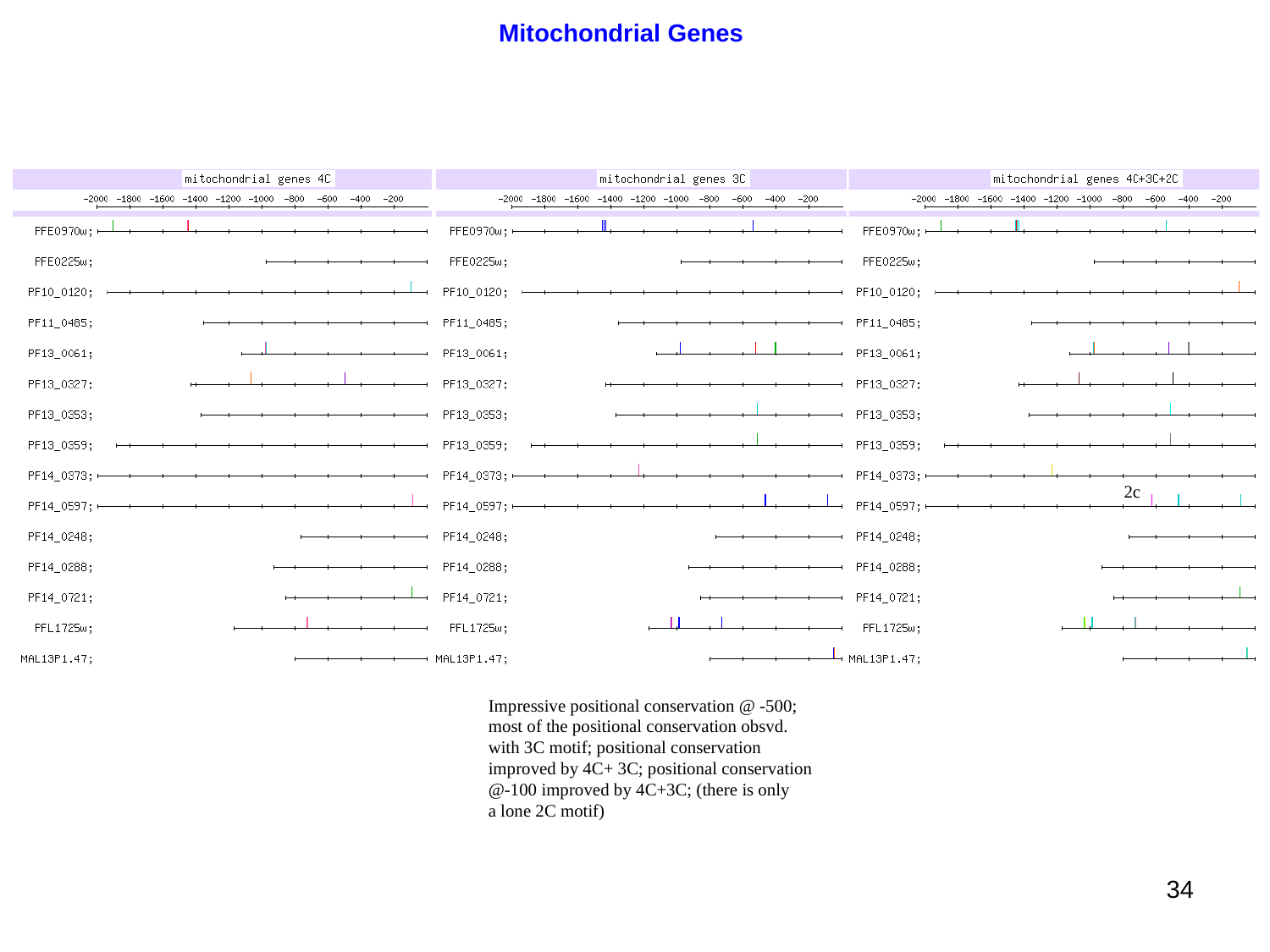

Mitochondrial Genes
2c
Impressive positional conservation @ -500;
most of the positional conservation obsvd.
with 3C motif; positional conservation
improved by 4C+ 3C; positional conservation
@-100 improved by 4C+3C; (there is only
a lone 2C motif)
34

## Slide 35
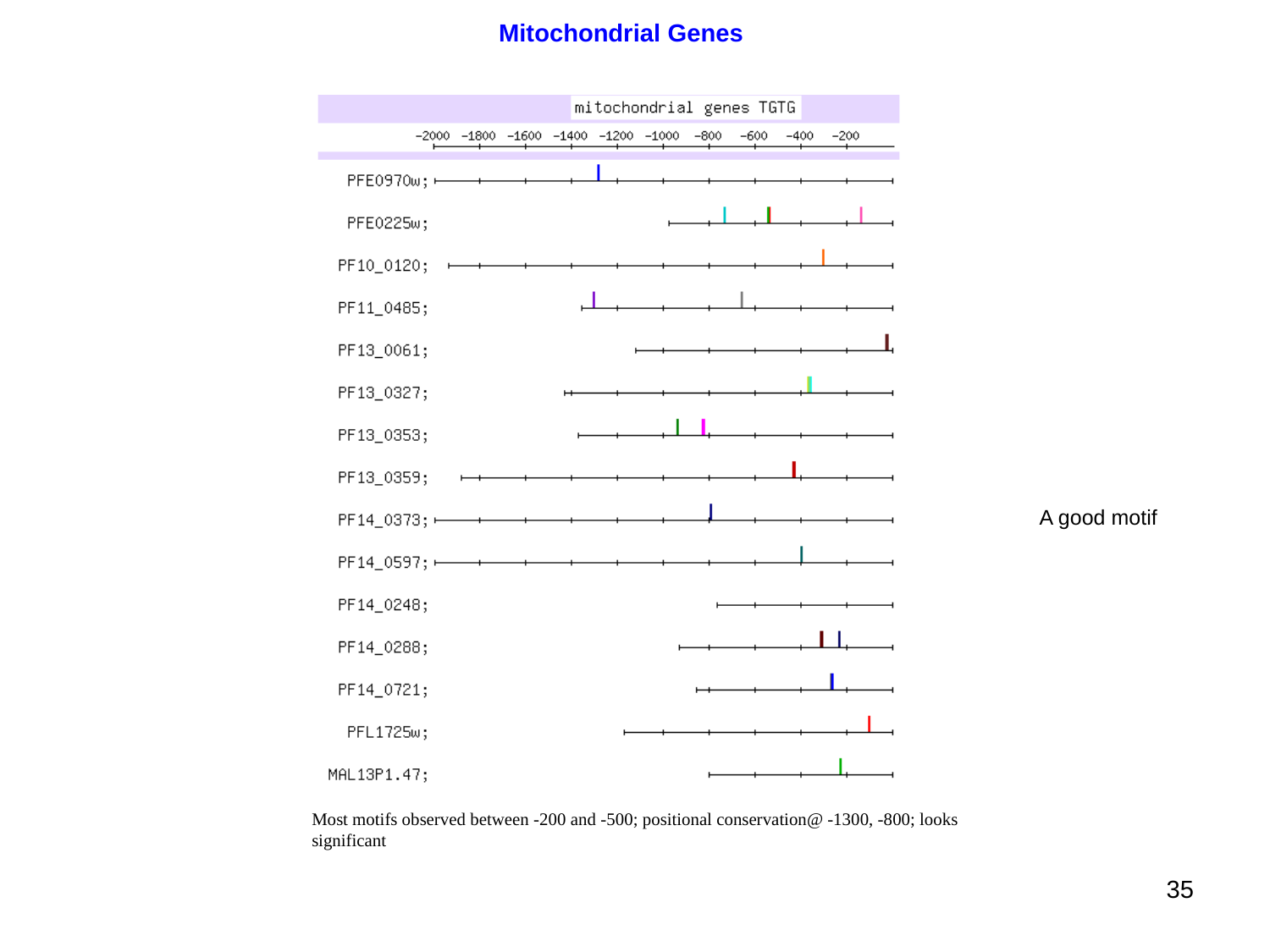

Mitochondrial Genes
A good motif
Most motifs observed between -200 and -500; positional conservation@ -1300, -800; looks
significant
35

## Slide 36
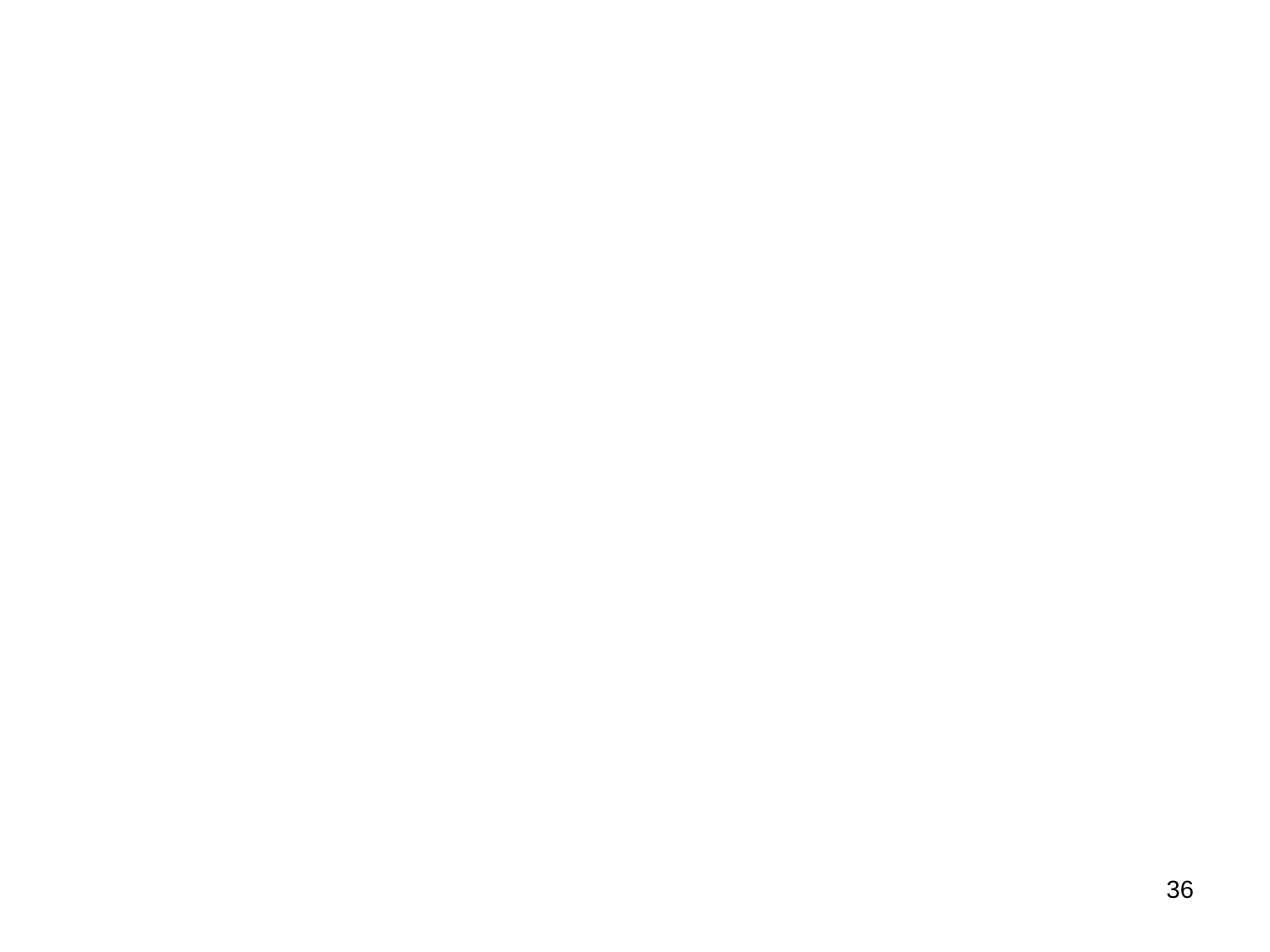

36

## Slide 37
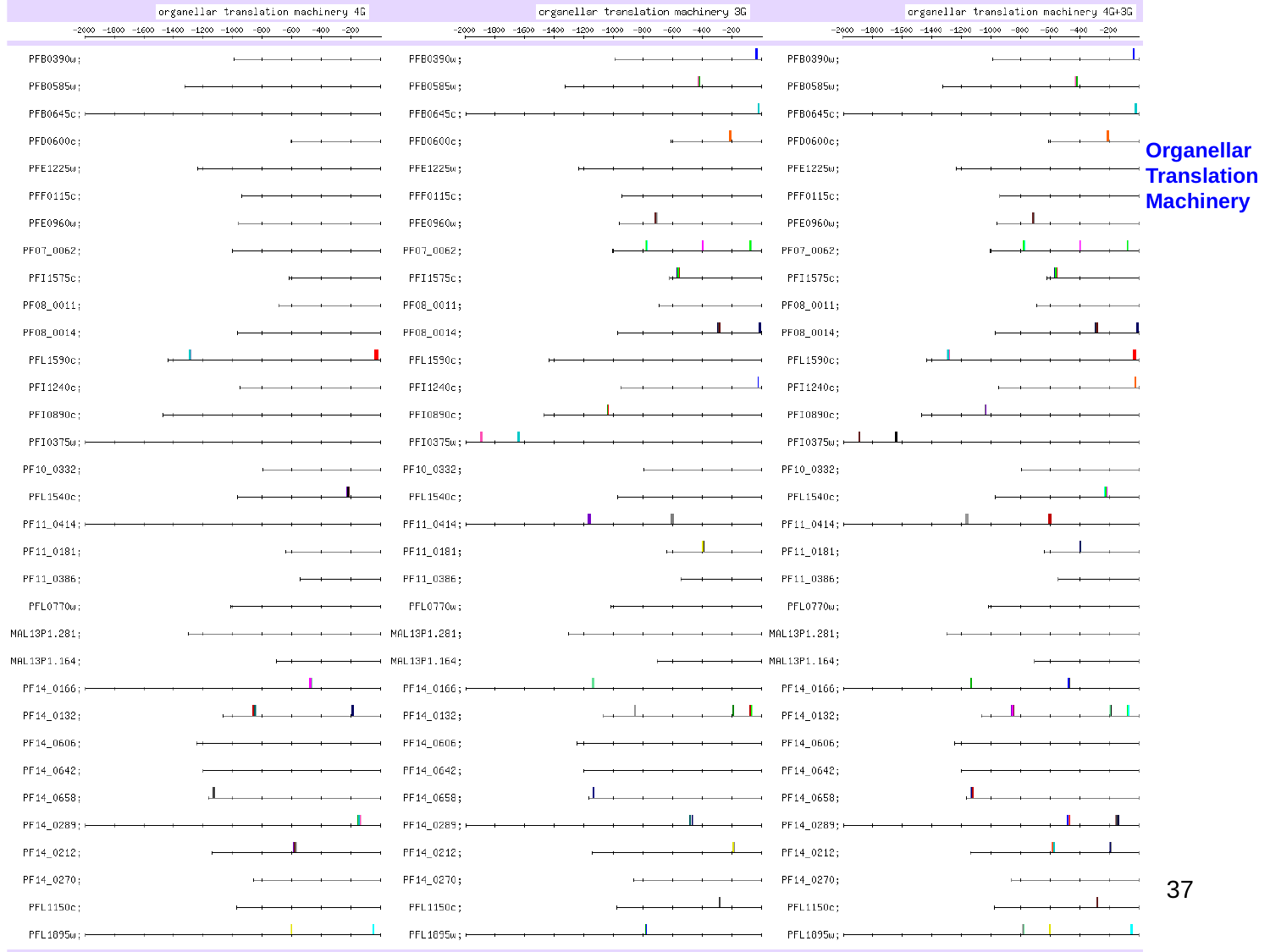

Organellar
Translation
Machinery
37

## Slide 38
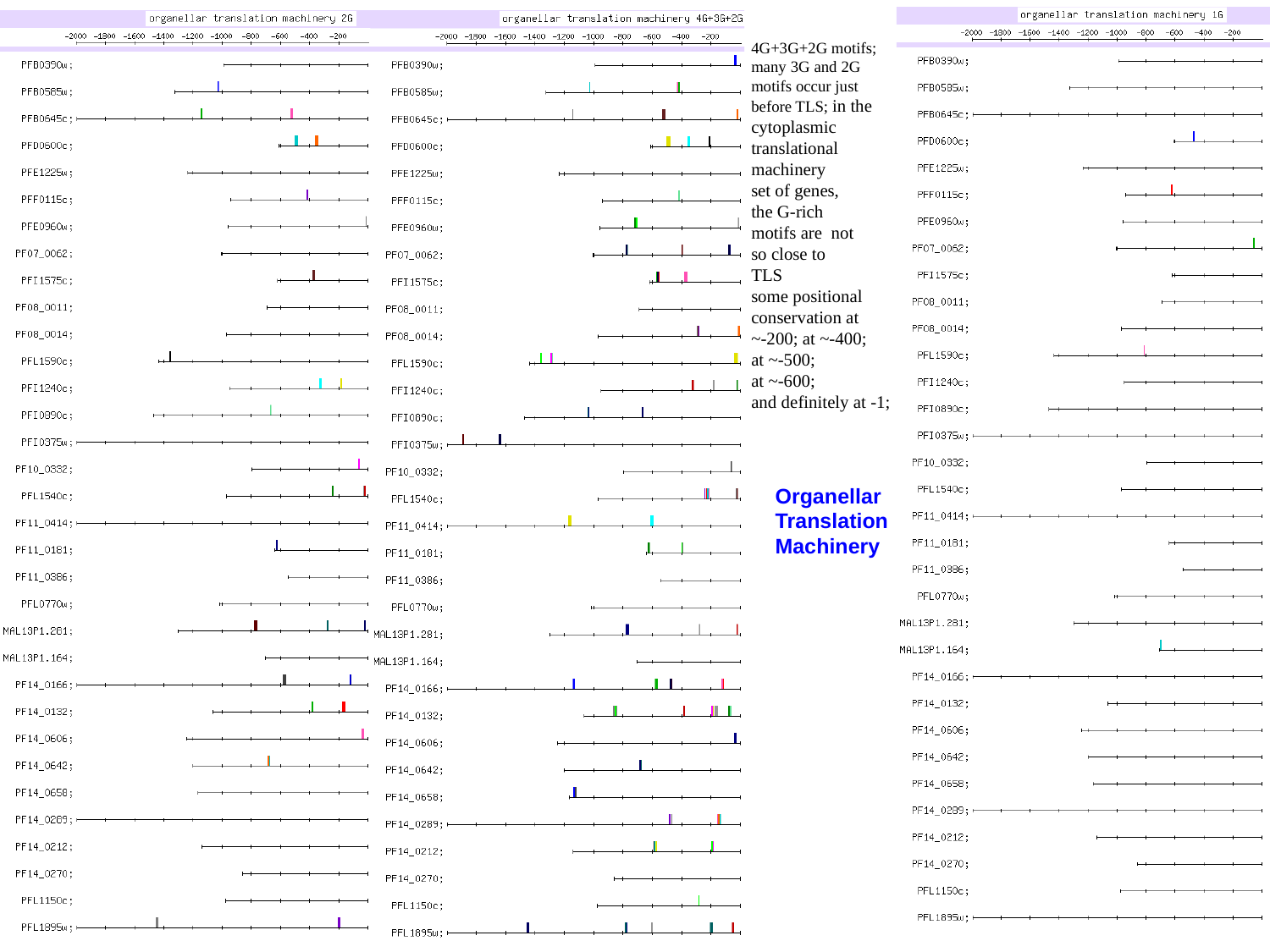

4G+3G+2G motifs;
many 3G and 2G
motifs occur just
before TLS; in the
cytoplasmic
translational
machinery
set of genes,
the G-rich
motifs are not
so close to
TLS
some positional
conservation at
~-200; at ~-400;
at ~-500;
at ~-600;
and definitely at -1;
Organellar
Translation
Machinery
38

## Slide 39
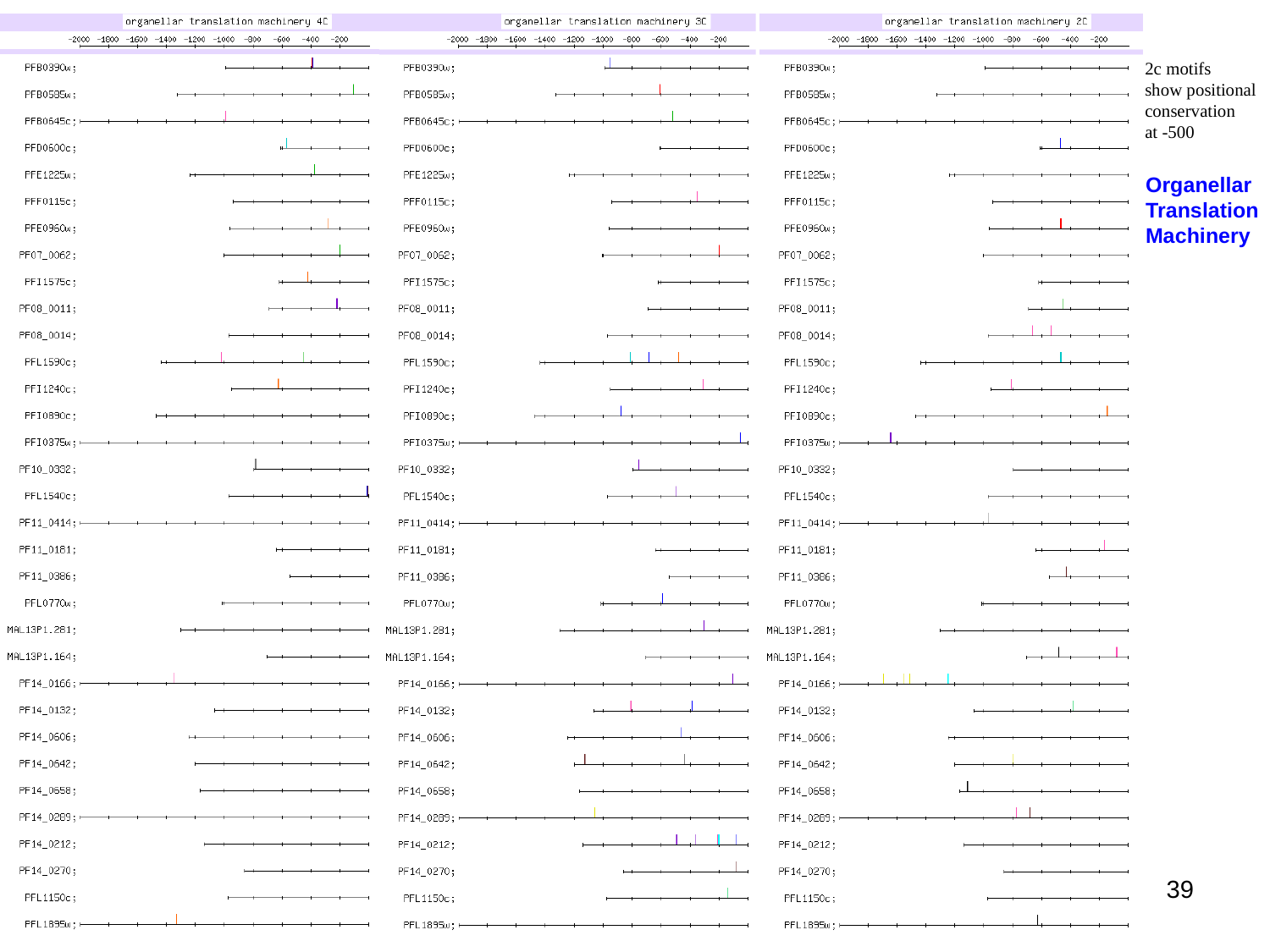

2c motifs
show positional
conservation
at -500
Organellar
Translation
Machinery
39

## Slide 40
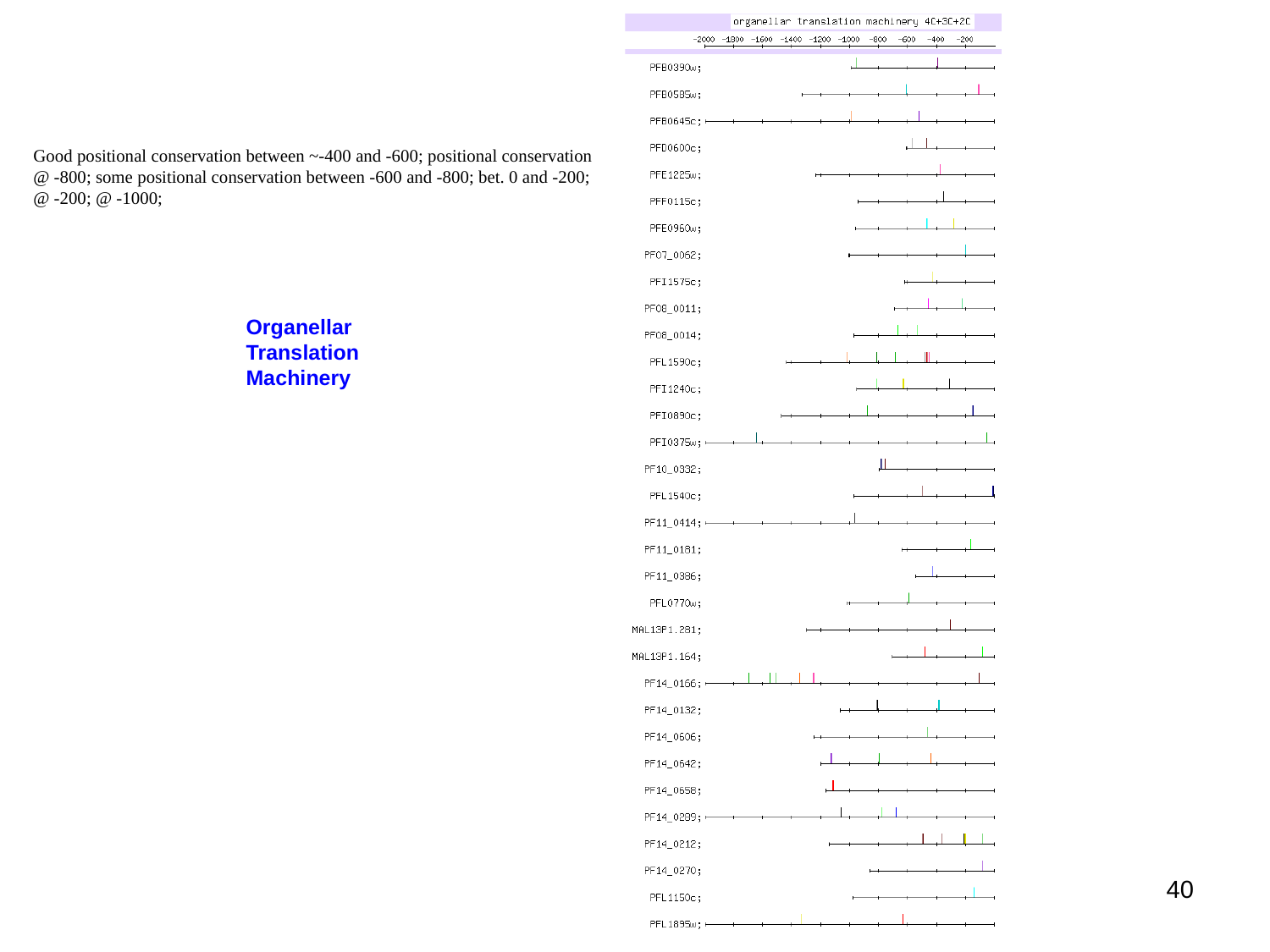

Good positional conservation between ~-400 and -600; positional conservation
@ -800; some positional conservation between -600 and -800; bet. 0 and -200;
@ -200; @ -1000;
Organellar
Translation
Machinery
40

## Slide 41
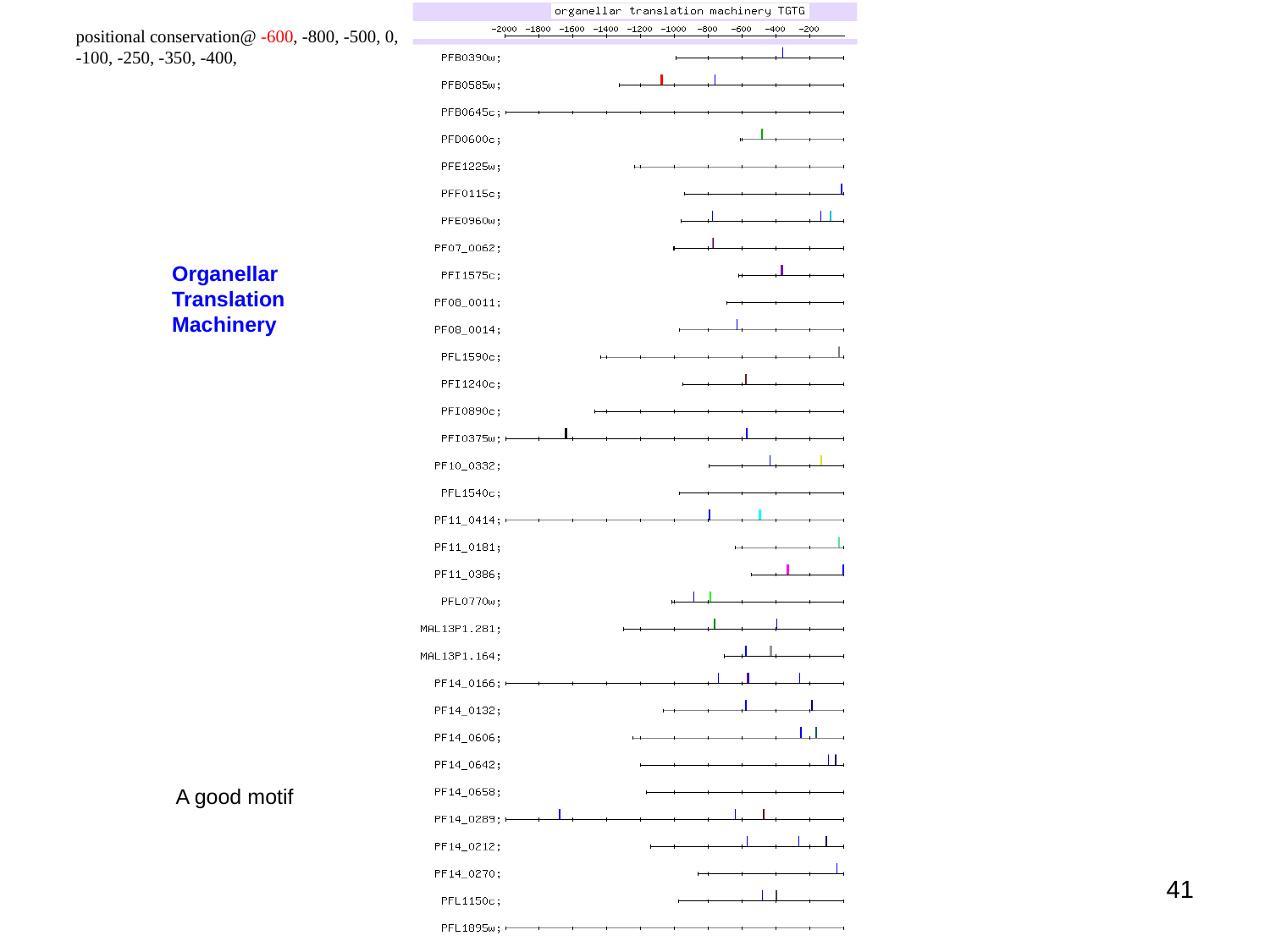

positional conservation@ -600, -800, -500, 0,
-100, -250, -350, -400,
Organellar
Translation
Machinery
A good motif
41

## Slide 42
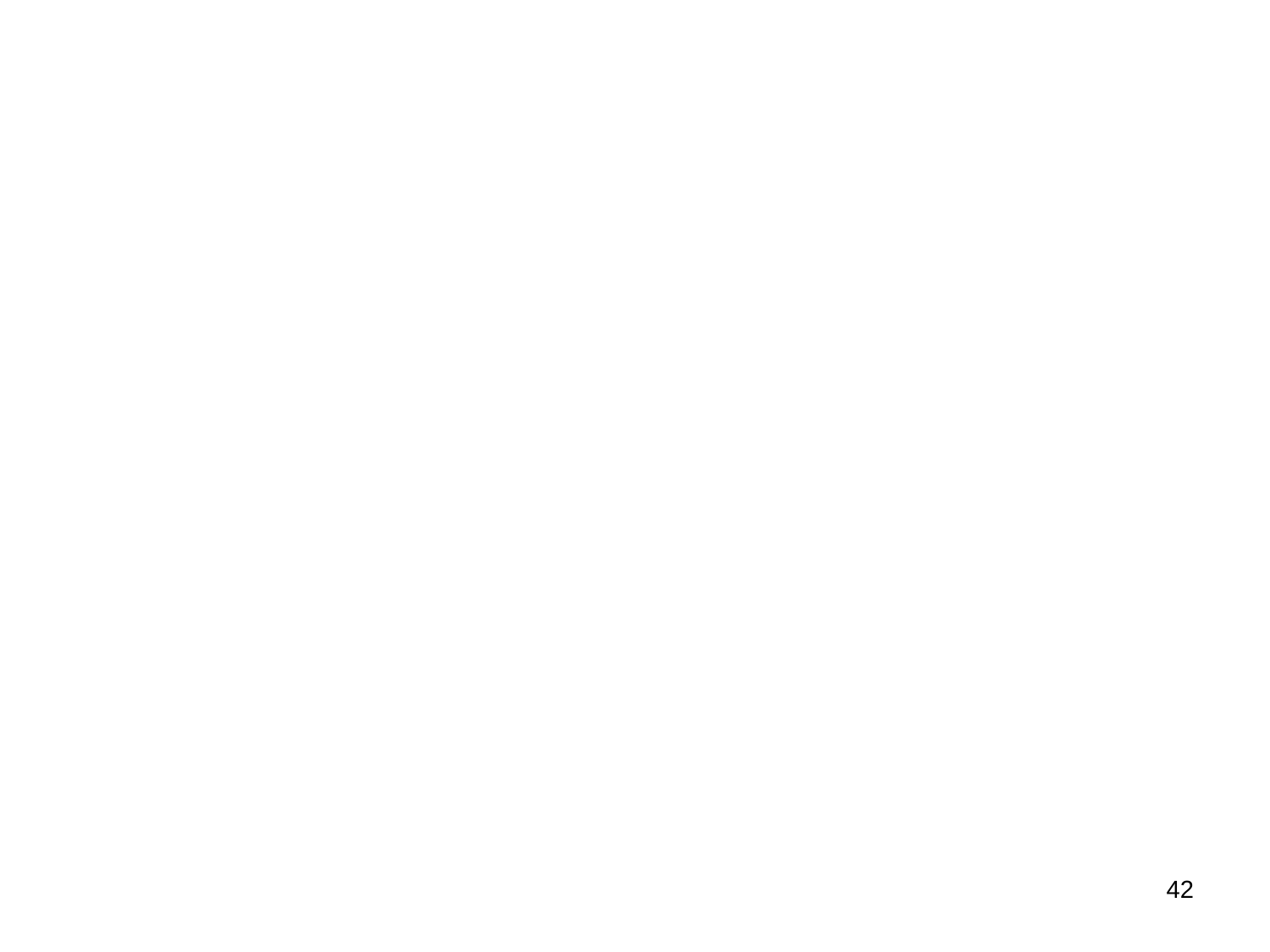

42

## Slide 43
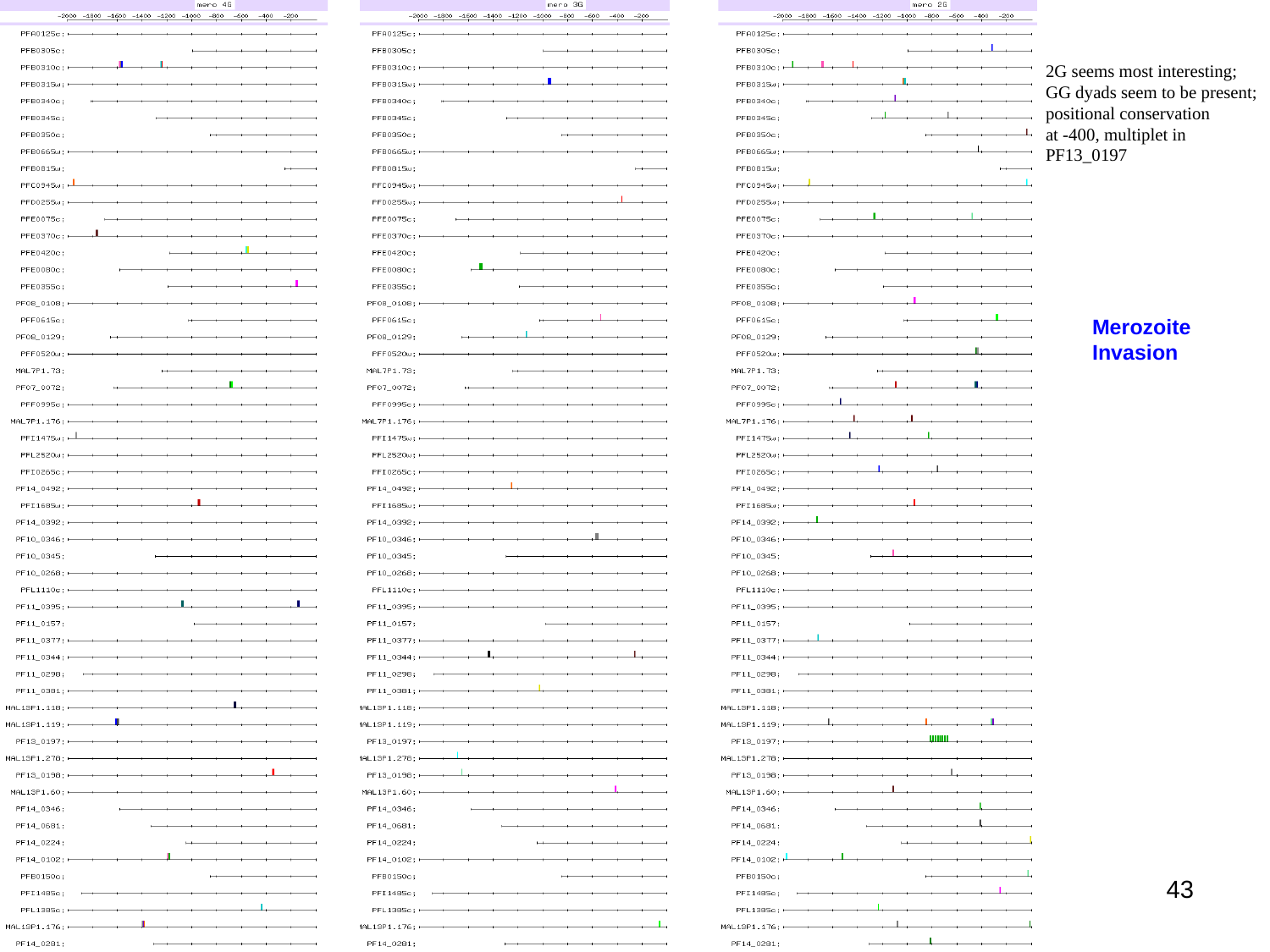

2G seems most interesting;
GG dyads seem to be present;
positional conservation
at -400, multiplet in
PF13_0197
Merozoite
Invasion
43

## Slide 44
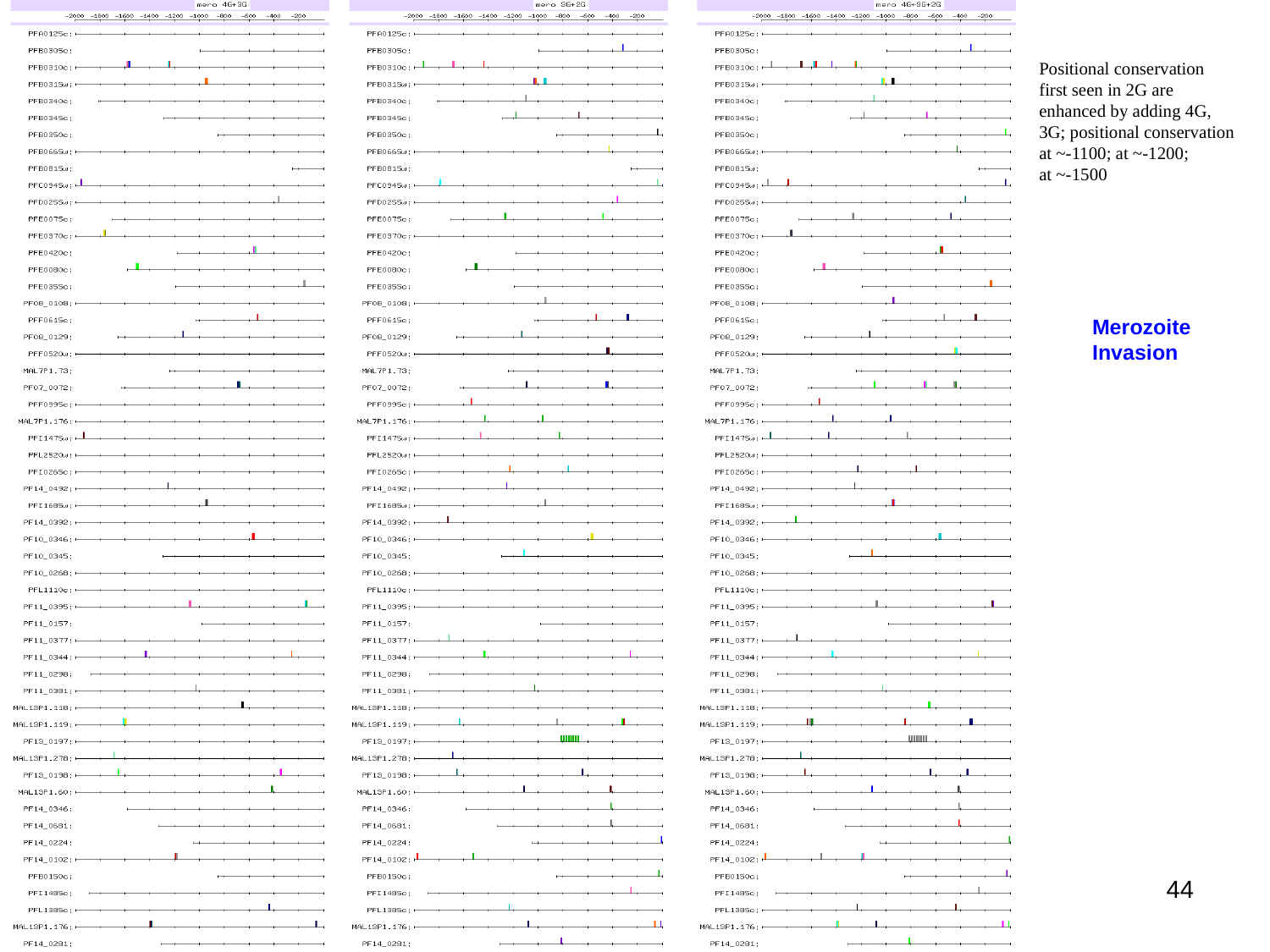

Positional conservation
first seen in 2G are
enhanced by adding 4G,
3G; positional conservation
at ~-1100; at ~-1200;
at ~-1500
Merozoite
Invasion
44

## Slide 45
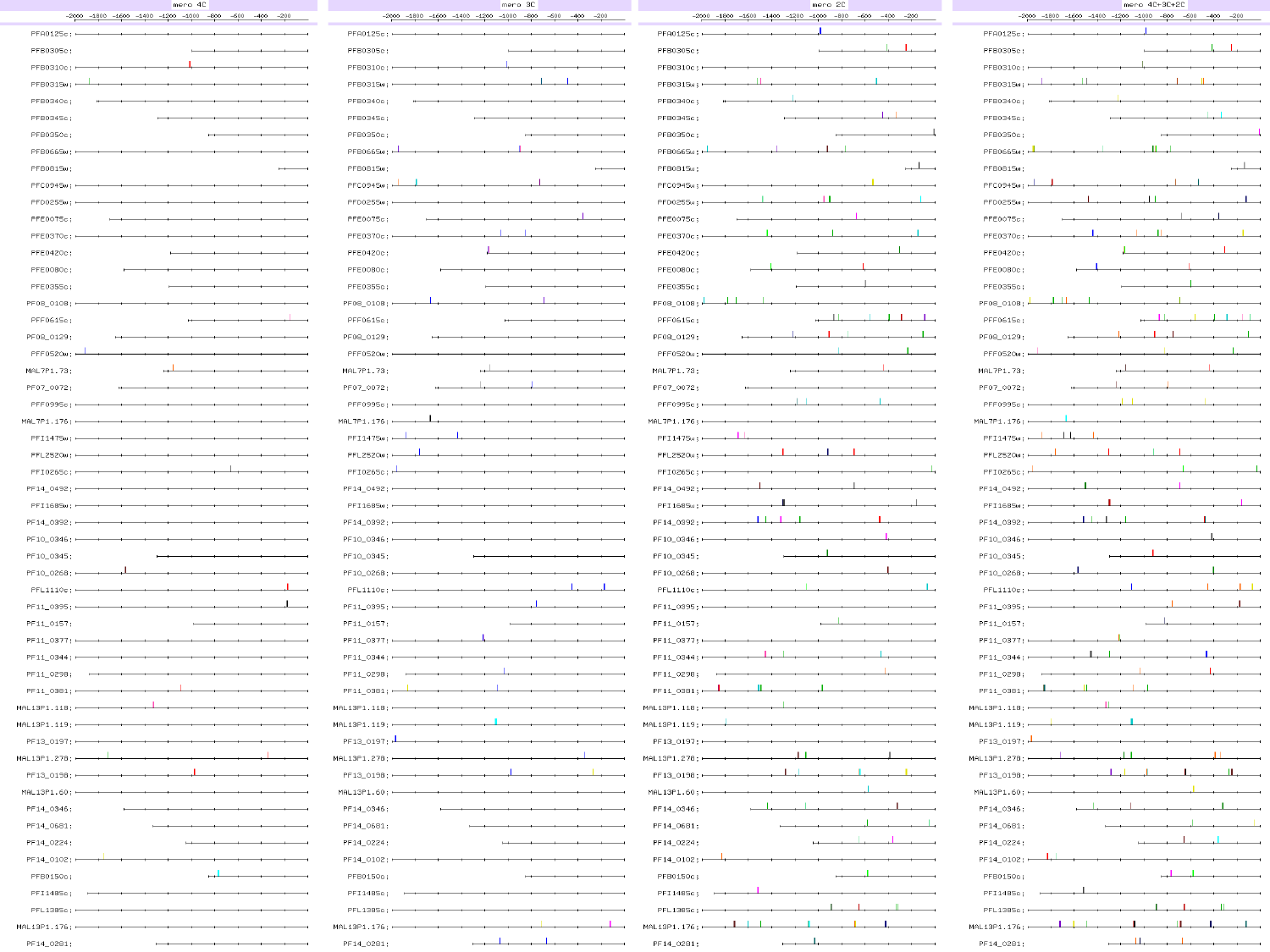

45

## Slide 46
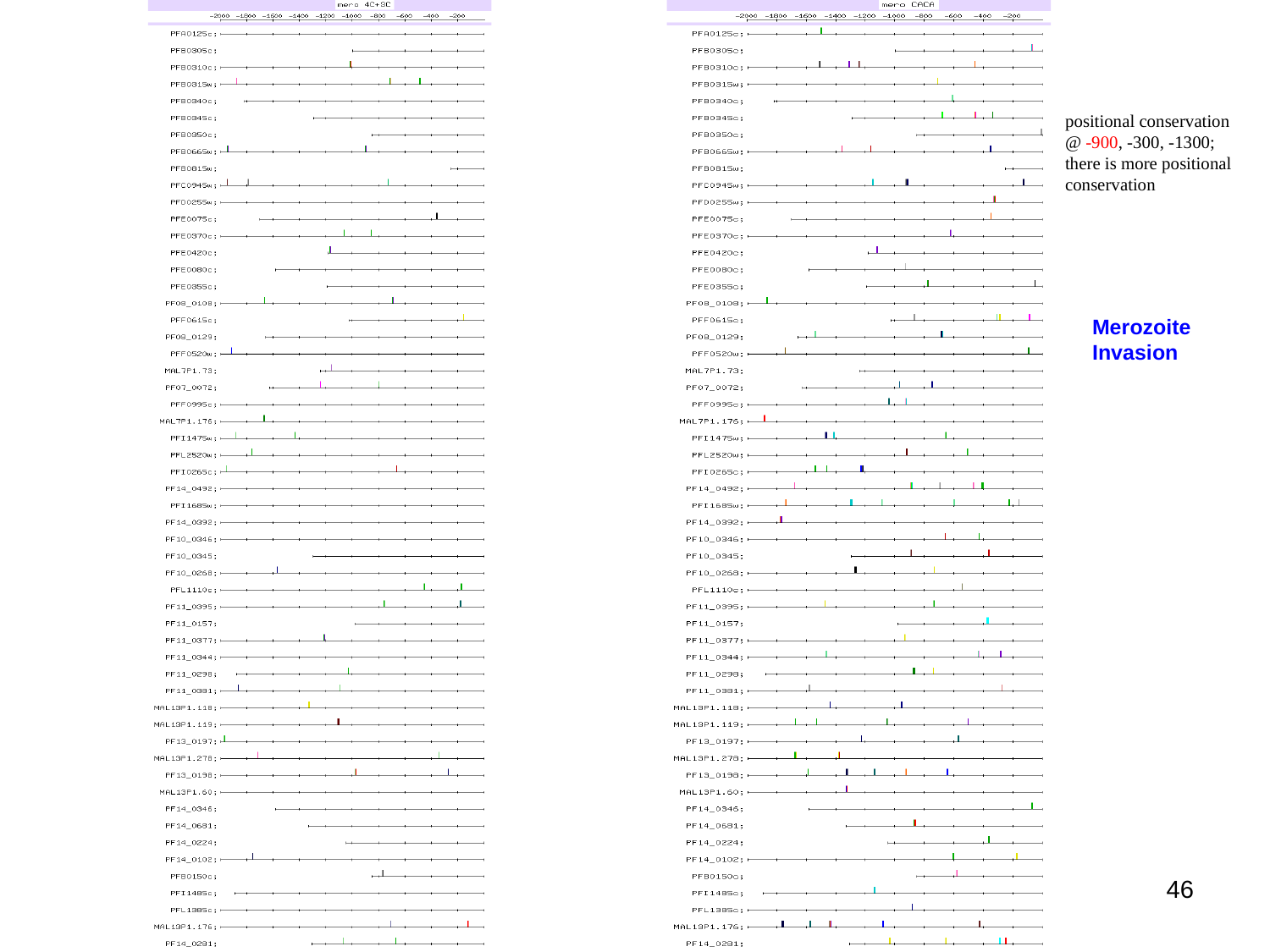

positional conservation
@ -900, -300, -1300;
there is more positional
conservation
Merozoite
Invasion
46

## Slide 47
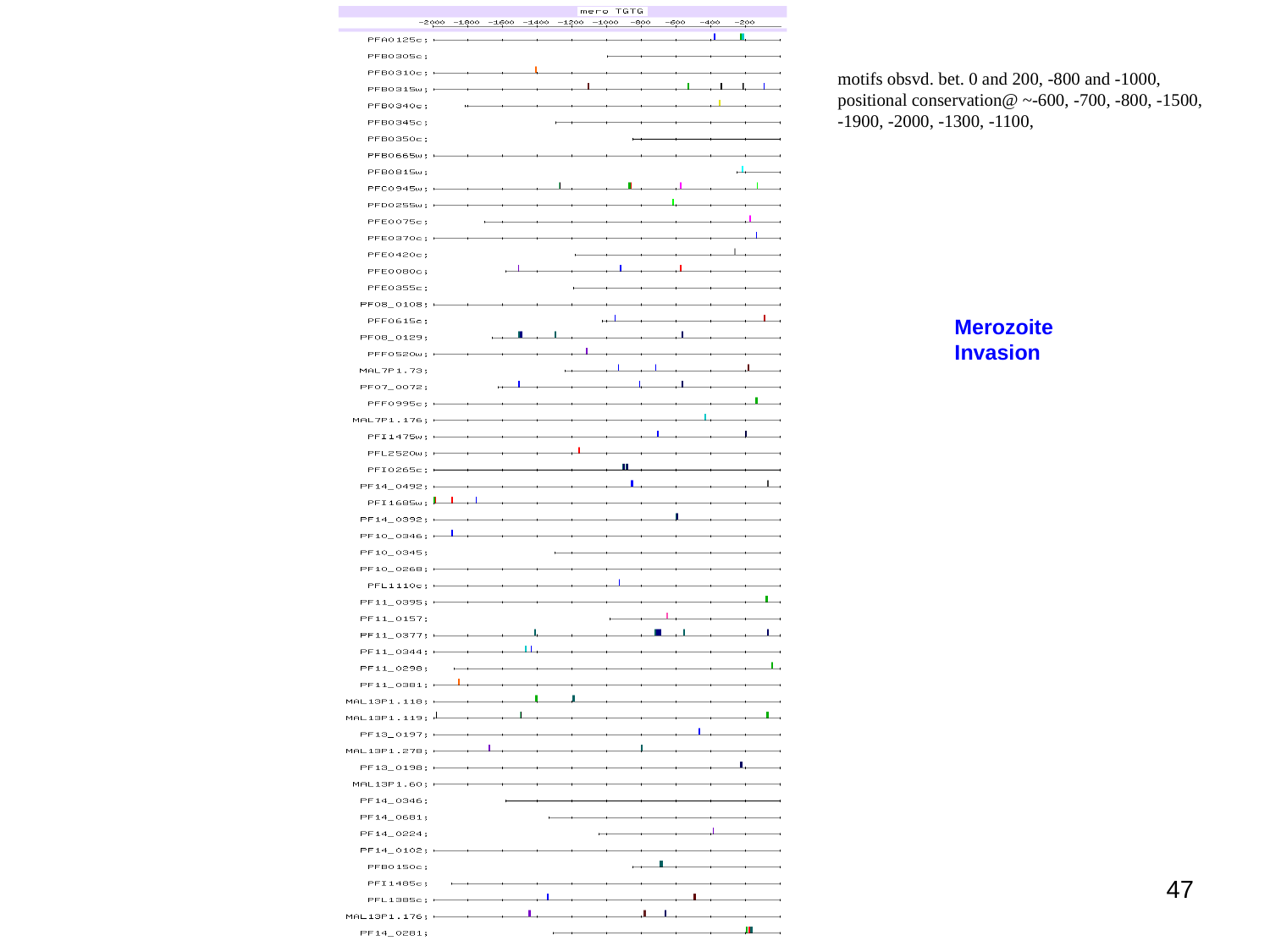

motifs obsvd. bet. 0 and 200, -800 and -1000,
positional conservation@ ~-600, -700, -800, -1500,
-1900, -2000, -1300, -1100,
Merozoite
Invasion
47

## Slide 48
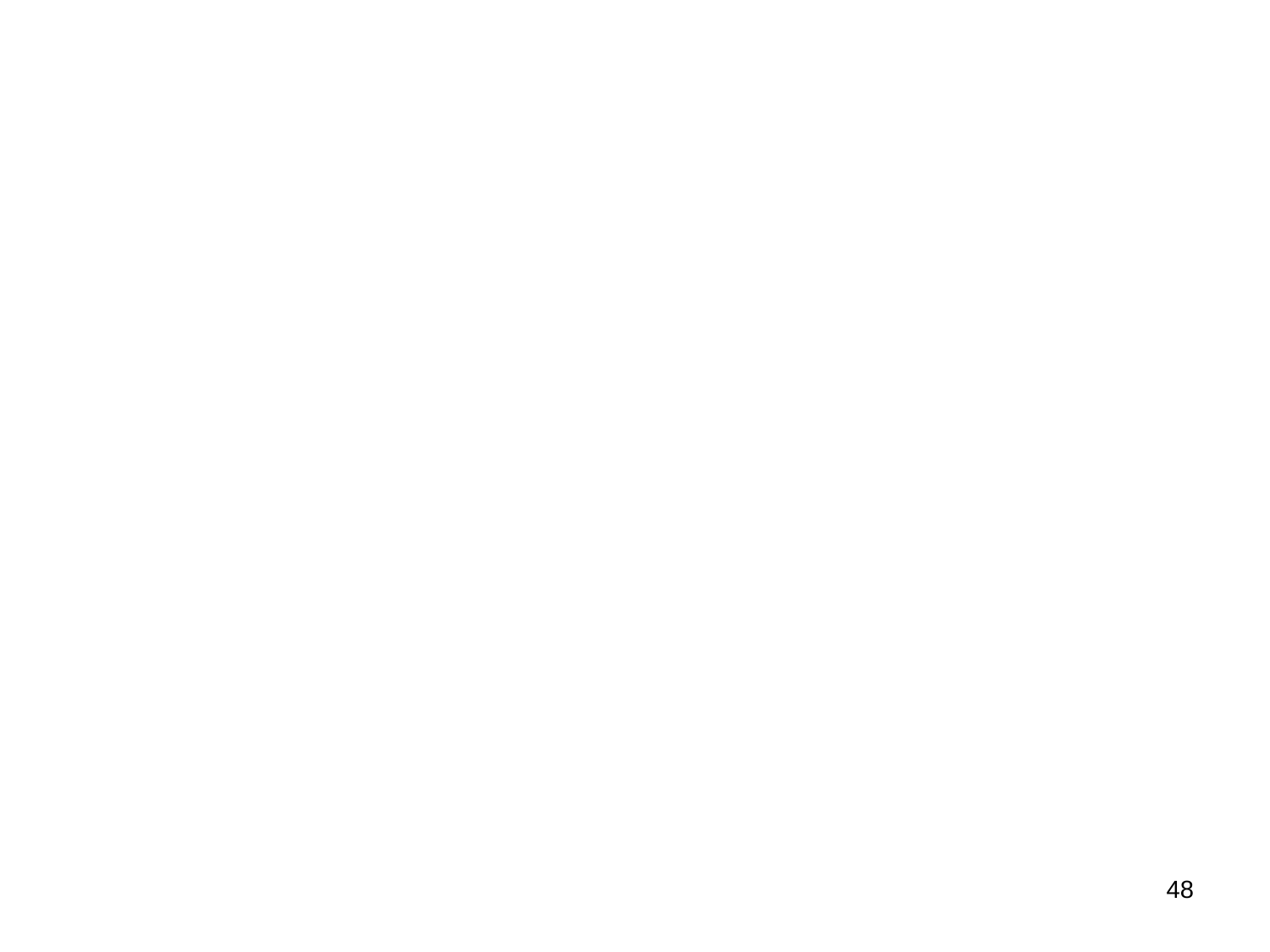

48

## Slide 49
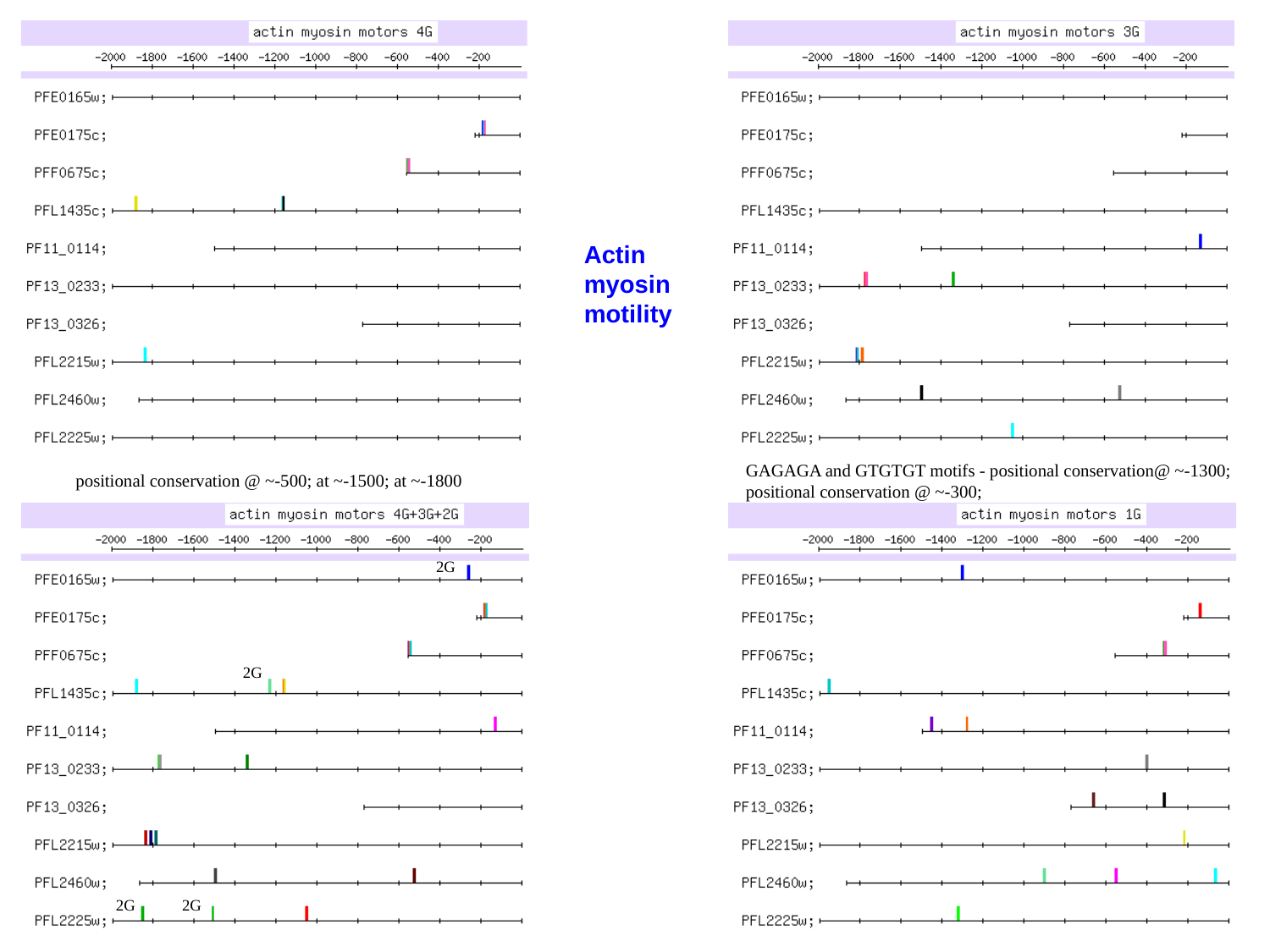

Actin
myosin
motility
GAGAGA and GTGTGT motifs - positional conservation@ ~-1300;
positional conservation @ ~-300;
positional conservation @ ~-500; at ~-1500; at ~-1800
2G
2G
49
2G
2G

## Slide 50
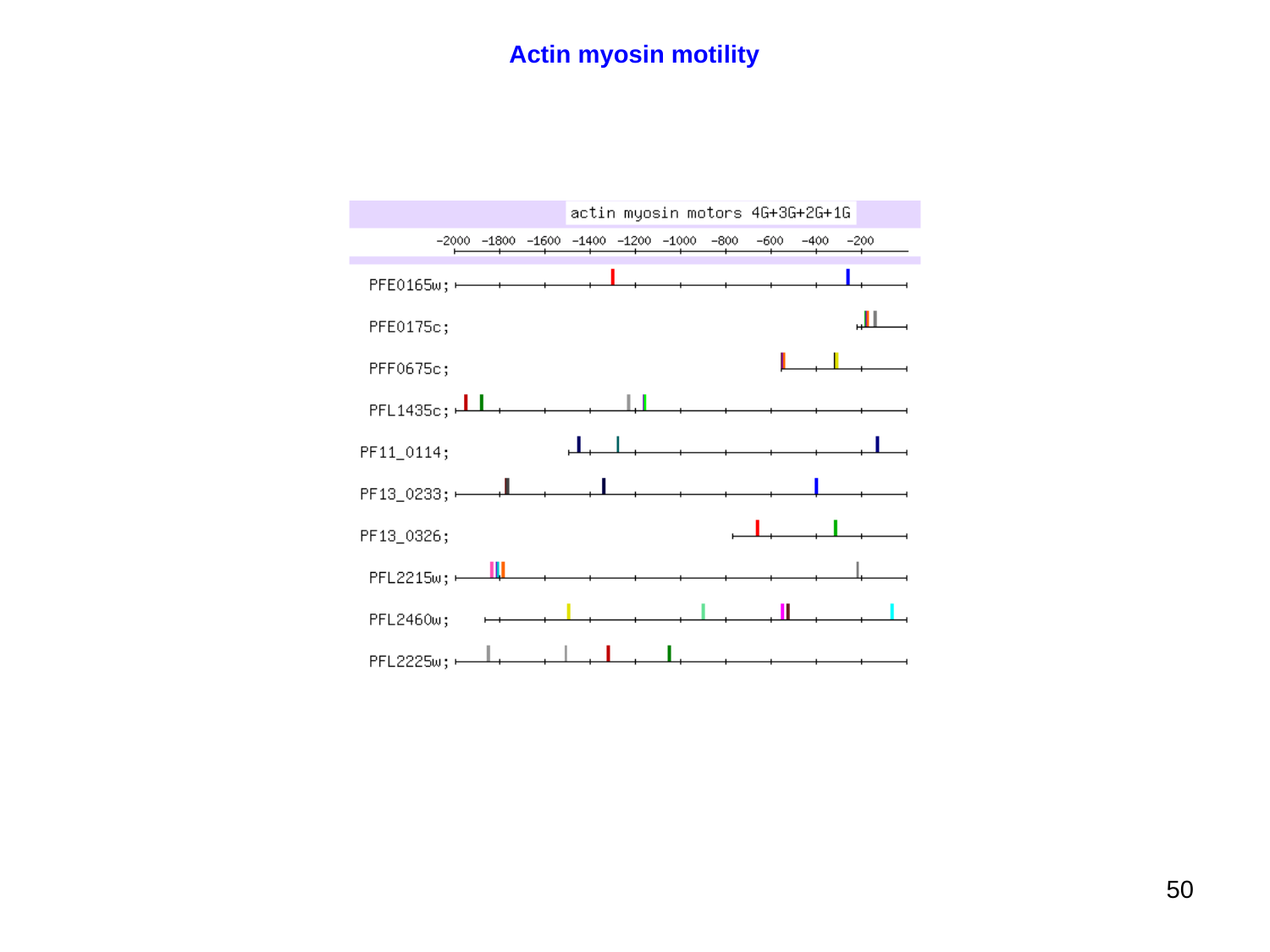

Actin myosin motility
50

## Slide 51
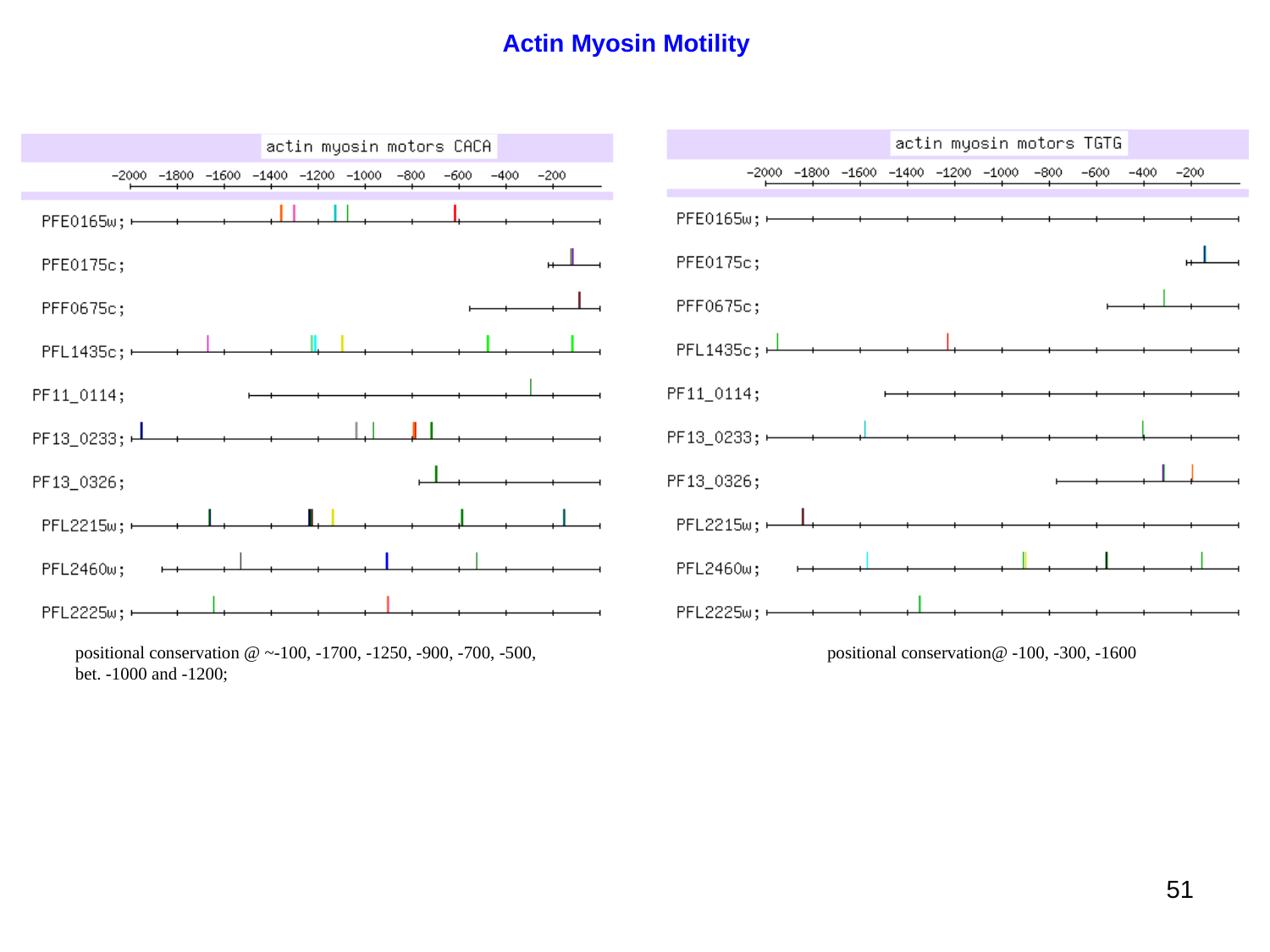

Actin Myosin Motility
positional conservation @ ~-100, -1700, -1250, -900, -700, -500,
bet. -1000 and -1200;
positional conservation@ -100, -300, -1600
51

## Slide 52
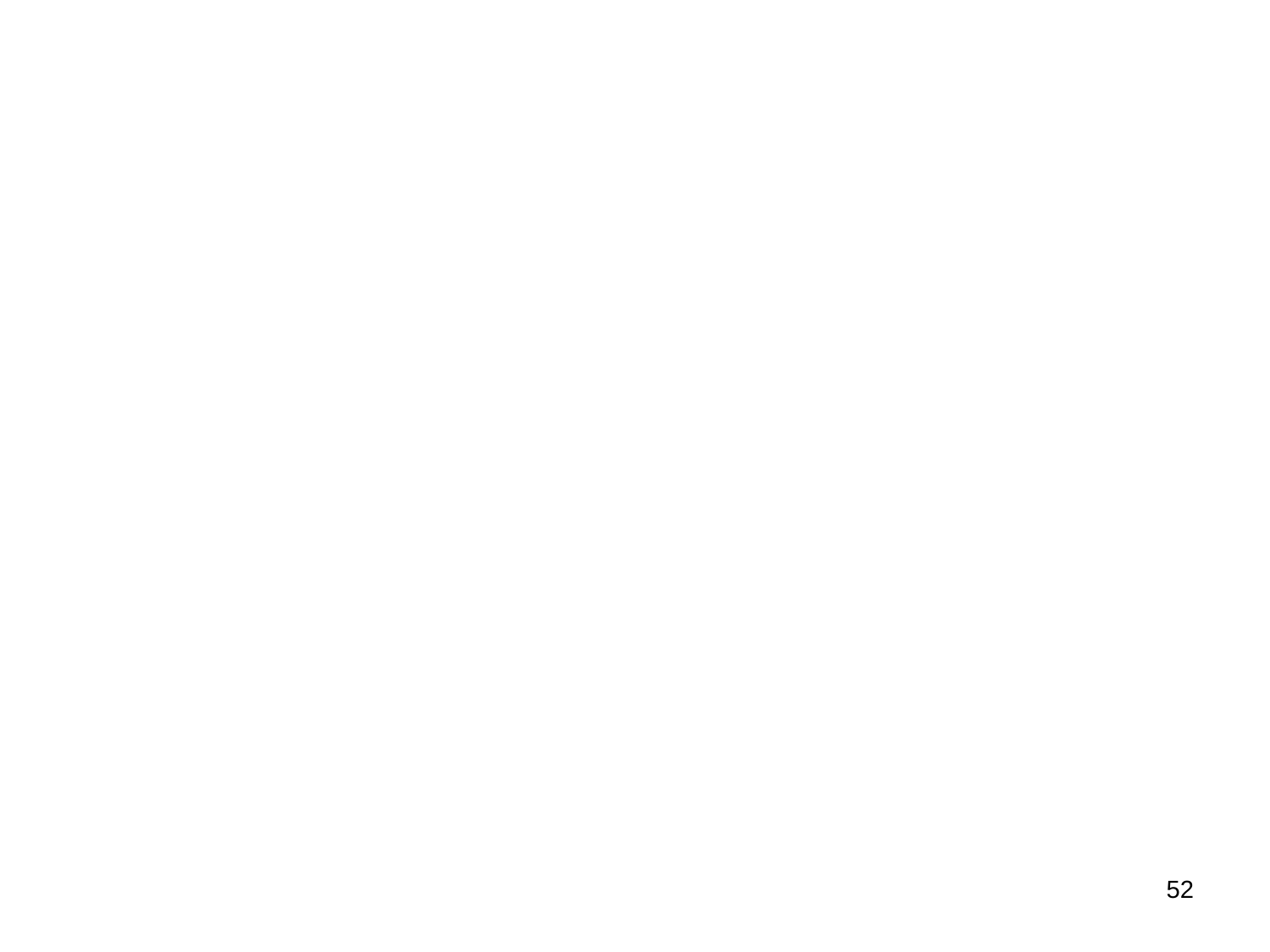

52
