# Supplementary material for: Identification of putative regulatory motifs in the upstream regions of co-expressed functional groups of genes in Plasmodium falciparum
Source: BMC Genomics. 2009 Jan 13;10:18. doi: 10.1186/1471-2164-10-18 (PMC2662883; doi:10.1186/1471-2164-10-18)
Supplement: Additional file 8 — Statistically validated positionally conserved motifs. Positionally conserved upstream motifs which have been statistically validated by Monte Carlo simulations are listed. [file 1471-2164-10-18-S8.doc]

Additional file 8. Statistically validated positionally conserved motifs are listed.

In Additional file 6, sets of motifs occurring in each set of upstream sequences are listed. Positionally conserved motifs, in Additional file 6, which have been statistically validated, are listed below.

In the table below, the set of gene upstream sequences and the specific motif being examined are given as a heading line. This heading line is similar to the corresponding heading line in Additional file 6. The heading line is followed by one or more sets of statistically validated positionally conserved motifs (the sequence and upstream position of each motif, and the upstream sequence in which the motif occurs is given). Each set of motifs is preceded by a line with 8 numbers; column headings for the numbers are given below the heading line. Column headings are explained below.

Upstream window: the upstream window in which the motifs occur; all the listed motifs and their positions occur in this window.

Nmot: the number of motifs observed in the window (the motifs are listed)

Nsimm: the number of simulations (out of 20,000) in which Nmot or more motifs are observed in the window (Methods).

Pmot: the probability that Nmot or more motifs occur in the window by chance.

Nsimm is used to estimate Pmot; for example, the probability, Pmot, of observing the first set of 4 motifs (transcription machinery set) in the window -1301 to -1400, by chance, is 53/20,000 or <0.005. If Nsimm is <1,000, the probability is <0.05 that Nmot or more motifs occur in the window by chance.

Nseq: the number of sequences with one or more motifs, observed in the window (the IDs of the sequences are listed)

Nsims: the number of simulations (out of 20,000) in which Nseq or more sequences with motifs are observed in the window.

Pseq: the probability that Nseq or more sequences with motifs occur in the window by chance.

Nsims is used to estimate Pseq; for example, the probability, Pseq, of observing the first set of 4 sequences with motifs (transcription machinery set), in the window -1301 to -1400, by chance is 23/20,000 or <0.005.

Each set of motifs listed below, is a set of statistically validated positionally conserved motifs, and has been highlighted in Additional file 6. For statistical validation, overlapping windows of width 100 nt have been considered, with the starting point of consecutive windows differing by 50 nt (Methods). If, in 2 consecutive windows, common motifs were found, motifs from both windows have been pooled together and considered as a single set of positionally conserved motifs; both sets have also been highlighted together in Additional file 6. For example, in the ribonucleotide synthesis set, common motifs occur in the consecutive windows, -751 to -850 and -801 to -900; motifs from both windows have been highlighted together, in Additional file 6, as a single set of positionally conserved motifs.

FIRST SET

Transcription machinery - G-rich - 4G+3G

Upstream window Nmot Nsimm Pmot Nseq Nsims Pseq

-1301 -1400 4 53 <0.005 4 23 <0.005

GGTGGGAAAAA -1382 PFF1390w

GGGTGAAAATAAAA -1374 PF11_0445

AGAGGGGAAAA -1366 PFE0465c

GCGGGTCTACAAGAAAAATGAA -1322 PFC0155c

Ribonucleotide synthesis - G-rich - 4G+3G+2G

Upstream window Nmot Nsimm Pmot Nseq Nsims Pseq

-751 -850 7 113 <0.01 7 36 <0.005

ATAAGGGCATATTTAAAA -835 PF10_0121

GTAGGAAAATATAA -827 PF10_0225

TGGGGGGA -824 PFI1420w

ATAAGGGCACAATAGAAA -815 PF10_0123

AAATGGGGATTTTTAAAA -805 PF13_0044

TACGCGCA -761 PF14_0697

CAGGTGCC -759 PF10_0086

-801 -900 6 338 <0.05 6 208 <0.05

CGGTGGCA -877 PFE0660c

ATAAGGGCATATTTAAAA -835 PF10_0121

GTAGGAAAATATAA -827 PF10_0225

TGGGGGGA -824 PFI1420w

ATAAGGGCACAATAGAAA -815 PF10_0123

AAATGGGGATTTTTAAAA -805 PF13_0044

-1301 -1400 4 629 <0.05 4 365 <0.05

ACAAGGGGAAAAAGGAAT -1397 MAL13P1.221

CCAAGGAGATAT -1370 PF13_0287

CGAAGGAA -1354 PF10_0121

CGAACACA -1316 PF10_0289

-1701 -1800 4 124 <0.01 4 50 <0.005

CAAGTGCC -1760 PF10_0123

TAAAGGCG -1748 PF13_0287

ATAAGGGGATATCAAAAA -1736 PF13_0044

TAAAGGGA -1733 PF14_0100

DNA replication – CACA

Upstream window Nmot Nsimm Pmot Nseq Nsims Pseq

-251 -350 8 328 <0.05 7 639 <0.05

CACCAAAAACACGAAAAAAA -350 PF14_0254

ATACACCT -333 PF14_0177

ACACACAT -332 PF07_0023

ACACACAT -328 PFF1225c

ACACAGCT -298 PF14_0602

CCACACAT -294 PF14_0254

ACATACAT -280 PFL1120c

TACACACC -264 PFB0895c

Proteasome – CACA

Upstream window Nmot Nsimm Pmot Nseq Nsims Pseq

-801 -900 10 76 <0.005 9 111 <0.01

GCACAC -884 PFI0630w

TACATACATAC -881 PFA0400c

GGCACATATAA -855 PF10_0081

CGCACAAGAAT -854 PF14_0716

TCACAC -845 PF10_0298

TCCACACAAAA -824 PF10_0174

TACATATATAC -823 PF14_0632

TACATACATAC -823 PFC0745c

ACACAC -816 PF14_0676

TACATATATAC -815 PFC0745c

-851 -950 9 206 <0.05 8 309 <0.05

AGCATACATAC -947 PF13_0063

TCTTACACCCC -938 PF13_0282

TACATACATAC -911 MAL13P1.190

TCACAC -905 PF14_0632

TACATACATAT -903 MAL13P1.190

GCACAC -884 PFI0630w

TACATACATAC -881 PFA0400c

GGCACATATAA -855 PF10_0081

CGCACAAGAAT -854 PF14_0716

-1601 -1700 8 60 <0.005 8 13 <0.001

ACACAC -1697 PF14_0716

TCACAC -1682 PF14_0632

ACACAC -1657 PF14_0676

TCACAC -1655 MAL13P1.270

TCACAC -1649 PFF0420c

TGCACATAAAT -1626 PF14_0025

TACATACATAT -1619 PFB0260w

GACATATATAC -1606 PF13_0033

Mitochondrial genes - C-rich - 4C+3C+2C

Upstream window Nmot Nsimm Pmot Nseq Nsims Pseq

-451 -550 6 80 <0.005 6 33 <0.005

TTTCCC -544 PFE0970w

GTGCCC -527 PF13_0061

TAGCCC -515 PF13_0353

GTTCCC -515 PF13_0359

CACCCC -497 PF13_0327

TTTCCC -466 PF14_0597

-501 -600 5 389 <0.05 5 233 <0.05

ACGCAT -565 PFE0225w

TTTCCC -544 PFE0970w

GTGCCC -527 PF13_0061

TAGCCC -515 PF13_0353

GTTCCC -515 PF13_0359

Organellar translation machinery - G-rich - 4G+3G+2G

Upstream window Nmot Nsimm Pmot Nseq Nsims Pseq

-1 -100 13 42 <0.005 13 4 <0.001

GAAAAGAGGGATGTATA -84 PF07_0062

TTGATAGGTGGG -83 PF14_0132

GGAAGGACAAA -67 PF10_0332

ATGTATGGGGATATG -57 PFL1895w

CACTATGGGTAGCTG -45 PFB0390w

CTTAATGAGGAG -42 PF14_0606

TTTACATATATTGGGGTGTACA -42 PFL1590c

CAAGAGGA -34 PFL1540c

ACATATGGGTACCTT -30 PFB0645c

GAGAGGAAATA -27 MAL13P1.281

AAAAGGGAAAG -27 PFI1240c

CGGTTTGTGGAG -19 PFE0960w

TGCTATGGGCAAGTT -17 PF08_0014

Organellar translation machinery – TGTG

Upstream window Nmot Nsimm Pmot Nseq Nsims Pseq

-551 -650 8 808 <0.05 8 543 <0.05

TGTGAA -643 PF14_0289

TGTGAA -634 PF08_0014

TATGTGTATGT -584 PFI1240c

TGTGAA -581 MAL13P1.164

TGTGAA -581 PF14_0132

TGTGAA -576 PFI0375w

TGTGGAGTTGT -574 PF14_0166

TGTGAA -572 PF14_0212

-701 -800 7 838 <0.05 7 569 <0.05

TGTGAA -796 PF11_0414

TGTGTATATTT -795 PFL0770w

TGTGAA -778 PFE0960w

GGTGTGAAAGG -777 PF07_0062

AGTGGATGTGA -770 MAL13P1.281

TGTGAA -765 PFB0585w

TGTGAA -743 PF14_0166

-------------------------------------------------------------------

-------------------------------------------------------------------

-------------------------------------------------------------------

SECOND SET

Cytoplasmic translation machinery - 4G

Upstream window Nmot Nsimm Pmot Nseq Nsims Pseq

-751 -850 13 9 <0.001 9 457 <0.05

TGGGGTTC -848 PF14_0240

TGTGGGGGGT -814 PF08_0076

TGTAATTAAAGGGGTT -801 PFL2055w

AAGAAATGCGGGGTG -793 PF14_0627

ACATAGGGGG -791 PFE0845c

TAGGGGAAAAA -790 MAL13P1.209

AAAAGTGGGGGA -790 PF10_0149

ATAGGGGGGAGG -789 PFE0845c

GGGGAA -788 MAL13P1.209

GTGGGGGAATGT -786 PF10_0149

GCGGGG -786 PF14_0627

TAGGGGTTATT -771 PF10_0038

AGTGAAAAAAGGGGAC -757 PF11_0312

-1101 -1200 10 151 <0.01 9 336 <0.05

AAGGGGAAAAG -1192 PF08_0039

AGGGGTTT -1181 PF11_0051

ATAAAAGGGG -1161 MAL13P1.209

AAGGGGATATG -1146 PF10_0043

GGGAAAACAAGGGGAA -1137 PFL0210c

GGGGTAT -1135 MAL13P1.92

GGGGCCT -1135 PF10_0043

ATGTATAAGGGG -1132 PF07_0088

AAAAAAGGGG -1120 PFF0885w

TAGGGGGAGGGA -1111 PF07_0080

Cytoplasmic translation machinery - 4C

Upstream window Nmot Nsimm Pmot Nseq Nsims Pseq

-851 -950 11 88 <0.005 10 196 <0.01

CCCCATTTTTG -948 PFC0295c

TTTTCCCC -942 PF07_0043

GCCCCT -942 PFE1005w

CCCCTT -939 PF11_0272

TTTTCCCC -938 PFE0185c

CCCCCCCTTTA -925 PF11_0312

CCCCCTTTATCACCACATA -916 PFC1020c

AACCCC -912 PFE0185c

CCCCACAGTGA -909 PFC0400w

CCCCCT -890 PFF0885w

AACCCC -875 PF14_0428

-901 -1000 10 236 <0.05 9 573 <0.05

CCCCCT -986 PF07_0079

CCCCATTTTTG -948 PFC0295c

TTTTCCCC -942 PF07_0043

GCCCCT -942 PFE1005w

CCCCTT -939 PF11_0272

TTTTCCCC -938 PFE0185c

CCCCCCCTTTA -925 PF11_0312

CCCCCTTTATCACCACATA -916 PFC1020c

AACCCC -912 PFE0185c

CCCCACAGTGA -909 PFC0400w

Cytoplasmic translation machinery – TGTG

Upstream window Nmot Nsimm Pmot Nseq Nsims Pseq

-1551 -1650 6 311 <0.05 6 308 <0.05

ATATGTGCTC -1640 PFC0535w

TGTGTTCCTTT -1633 MAL7P1.81

TGAGGTGTCCA -1604 PF07_0043

TTGTGTGGCC -1599 PF14_0240

TGTGTTCCTTG -1593 PFF0885w

GGAGTGCATAT -1570 PFF1500c

-1701 -1800 6 205 <0.05 6 203 <0.05

TGTGTTCCTTT -1794 PFC0775w

ATATGTGCAC -1790 PF11_0245

ATATGTGCAT -1761 PFE0885w

TATGTGTCCCT -1746 PFB0830w

TAAGTGCACAT -1713 PF13_0171

TCATGTGGAC -1710 PF13_0129

DNA replication machinery – TGTG

Upstream window Nmot Nsimm Pmot Nseq Nsims Pseq

-201 -300 7 1169 <0.06 7 917 <0.05

TTTATGTGTGTA -292 PF13_0251

TTTATGTGTGTA -274 PFE0155w

TGTGTG -259 PF11_0117

CATTAATGTGTA -259 PFA0545c

TATATGTGTGTG -235 PF13_0291

TCTTTTTGTGTA -229 MAL7P1.21

TGTATGTGTGTA -216 PF07_0023

DNA replication machinery - G-rich - 4G+3G+2G+1G

Upstream window Nmot Nsimm Pmot Nseq Nsims Pseq

-401 -500 9 237 <0.05 9 141 <0.01

TTTTTTTTGGTGTGGGG -496 PFB0840w

TATATCTTTCCTTGGGG -491 PFL1285c

AGGAAAGAAAA -473 MAL13P1.22

AAGAAAAGAAA -458 PFE0155w

GAGAAAAAGAA -456 PFE1345c

AAGAAAAGGAA -454 PFI0235w

AGAAAAGGCAG -451 PFA0545c

AAAGGAGATAAA -445 PFL0150w

AAGGGAATCAA -428 PF14_0602

-451 -550 9 261 <0.05 9 150 <0.01

AAAGGGAGAGAGAGA -515 PFL2005w

AGAAAAAGGAA -512 MAL7P1.21

TTTTTTTTGGTGTGGGG -496 PFB0840w

TATATCTTTCCTTGGGG -491 PFL1285c

AGGAAAGAAAA -473 MAL13P1.22

AAGAAAAGAAA -458 PFE0155w

GAGAAAAAGAA -456 PFE1345c

AAGAAAAGGAA -454 PFI0235w

AGAAAAGGCAG -451 PFA0545c

-1551 -1650 6 847 <0.05 6 605 <0.05

AGGAGAAGAAA -1607 PF14_0254

GGAGAAATATTAA -1603 MAL13P1.22

AAGAAGACGAG -1577 PFB0840w

ATTTTGAGAAAGGGA -1576 PF10_0165

TATATAATCCTGTGTGG -1575 PFD0590c

GAAAAAAGAAG -1566 PFF1470c

Proteasome - G-rich - 4G+3G

Upstream window Nmot Nsimm Pmot Nseq Nsims Pseq

-901 -1000 5 1087 <0.055 5 901 <0.05

GAGGGTTG -989 PFD0665c

GGGCAT -970 PFB0260w

CAGGGGGC -969 PFE0915c

ATTATGTGGGAAAAA -921 PFC0520w

ATGGGATG -913 MAL8P1.128

-951 -1050 5 1082 <0.055 5 893 <0.05

AGTAAGAGGGAATAA -1039 PF13_0063

GGGCAT -1007 PF14_0716

GAGGGTTG -989 PFD0665c

GGGCAT -970 PFB0260w

CAGGGGGC -969 PFE0915c

Proteasome – TGTG

Mitochondrial genes - G-rich - 4G+3G+2G+1G

Upstream window Nmot Nsimm Pmot Nseq Nsims Pseq

-201 -300 6 69 <0.005 6 38 <0.005

GAAAAAGGAA -300 PF10_0120

GTGAATGGCG -286 PF13_0061

CAAAACGGGA -282 PF14_0373

ATGCGCA -229 MAL13P1.47

CTCAAAGGGG -226 PFE0225w

GTAATAAGCG -204 PF14_0721

Mitochondrial genes - TGTG

Organellar translation machinery - C-rich - 4C+3C+2C

Upstream window Nmot Nsimm Pmot Nseq Nsims Pseq

-401 -500 12 259 <0.05 10 792 <0.05

ACCCAA -496 PF14_0212

TGCTCC -482 MAL13P1.164

GCTCCC -482 PFL1590c

GCCTCC -473 PFD0600c

TCCATTTTGC -472 PFE0960w

TCCATTTTGG -471 PFL1590c

TCCCAT -465 PF14_0606

GGCTCC -457 PF08_0011

TCCCCT -453 PFL1590c

GGCCCT -445 PF14_0642

TGCCAT -434 PF11_0386

TCCCCC -428 PFI1575c

-451 -550 12 279 <0.05 10 821 <0.05

ACACCT -538 PF08_0014

GGTCCC -522 PFB0645c

ACCCAA -505 PFL1540c

ACCCAA -496 PF14_0212

TGCTCC -482 MAL13P1.164

GCTCCC -482 PFL1590c

GCCTCC -473 PFD0600c

TCCATTTTGC -472 PFE0960w

TCCATTTTGG -471 PFL1590c

TCCCAT -465 PF14_0606

GGCTCC -457 PF08_0011

TCCCCT -453 PFL1590c

Merozoite invasion – TGTG

Upstream window Nmot Nsimm Pmot Nseq Nsims Pseq

-51 -150 10 527 <0.05 10 312 <0.05

ATATATGTGTA -141 PFF0995c

GTGTGT -137 PFE0370c

GTGCGC -132 PFC0945w

GTGTGT -98 PFB0315w

GTGTGTGTACA -98 PFF0615c

ATGTGTGTACC -86 PF11_0395

ATATATGTGTA -81 MAL13P1.119

GTATGTATATA -79 PF11_0377

GTATGTATGTG -77 PF14_0492

ATATATGTGTA -53 PF11_0298

-1401 -1500 8 479 <0.05 7 900 <0.05

ATATGTGCGTA -1499 MAL13P1.119

GTATGTATGTA -1493 PF08_0129

ACGTGCATGCC -1467 PF11_0344

GTGTGC -1446 MAL13P1.176

GTGTGT -1439 PF11_0344

ATATGTATGTA -1418 PF11_0377

ACATGTGTGTA -1414 PFB0310c

ATATATGTGTA -1411 MAL13P1.118

Merozoite invasion - G-rich - 4G+3G+2G

Merozoite invasion - 4C+3C

Upstream window Nmot Nsimm Pmot Nseq Nsims Pseq

-651 -750 7 390 <0.05 6 909 <0.05

TTTTCCCT -731 PFC0945w

TTTCCC -717 PFB0315w

GTTCCC -714 MAL13P1.176

CCCACACA -714 PFB0315w

TTTCCCAT -697 PF08_0108

TTTTCCCT -675 PF14_0281

TGCCCCAT -668 PFI0265c

-701 -800 7 320 <0.05 6 880 <0.05

TTTCCC -797 PF07_0072

TTTACCCC -773 PFB0150c

TTTCCC -761 PF11_0395

TTTTCCCT -731 PFC0945w

TTTCCC -717 PFB0315w

GTTCCC -714 MAL13P1.176

CCCACACA -714 PFB0315w

-1851 -1950 6 247 <0.05 6 195 <0.01

TTTCCCAT -1947 PFB0665w

GGTCCC -1947 PFC0945w

TTCCCC -1919 PFF0520w

TTTCCC -1884 PFI1475w

TACCCC -1880 PFB0315w

GTTCCC -1866 PF11_0381

-1901 -2000 5 725 <0.05 5 632 <0.05

TTTCCCAT -1971 PF13_0197

TTTCCC -1958 PFI0265c

TTTCCCAT -1947 PFB0665w

GGTCCC -1947 PFC0945w

TTCCCC -1919 PFF0520w

Actin myosin motility - CACA
