# Supplementary figures and images for: Identification of putative regulatory motifs in the upstream regions of co-expressed functional groups of genes in Plasmodium falciparum
Source: BMC Genomics. 2009 Jan 13;10:18. doi: 10.1186/1471-2164-10-18 (PMC2662883; doi:10.1186/1471-2164-10-18)

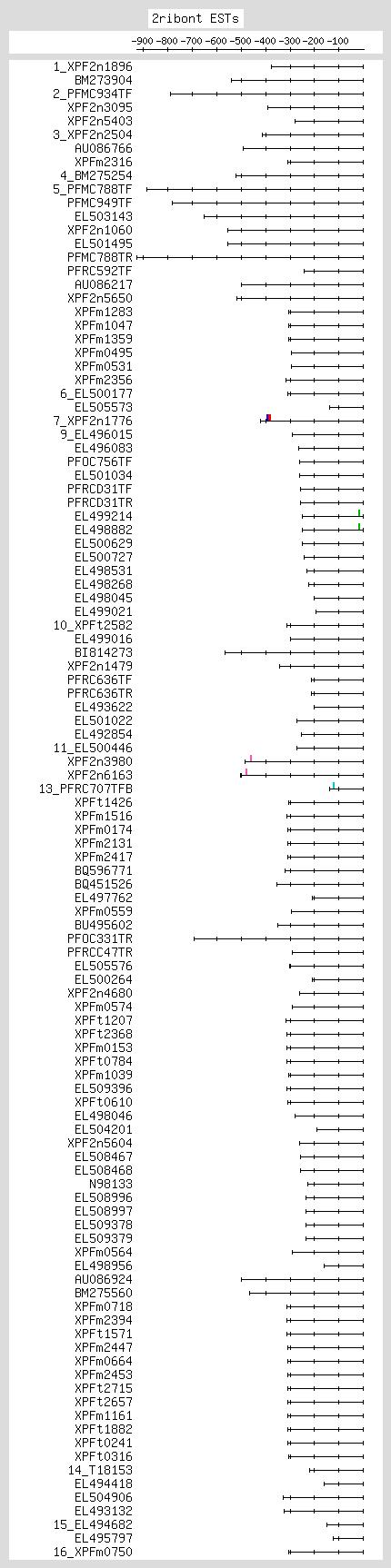

Supplement: Additional file 9 — Feature maps showing the occurrence of putative Pf regulatory motifs in ESTs and orthologous sequences. The maps show the distribution of putative Pf regulatory motifs (a) in ESTs retrieved for the gene upstream sequences and (b) in the upstream sequences of orthologous genes from other Plasmodium species. [file 1471-2164-10-18-S9.zip › Iengar_addnl_file9_bmcg/map_2ribont_est.jpg]

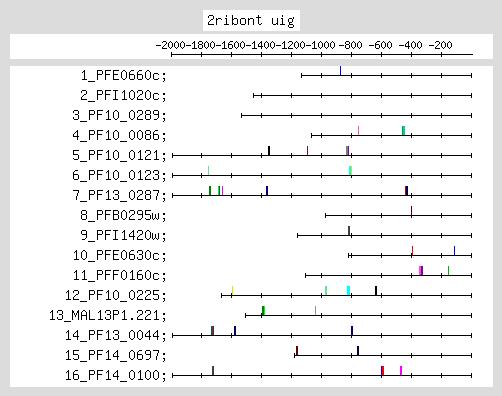

Supplement: Additional file 9 — Feature maps showing the occurrence of putative Pf regulatory motifs in ESTs and orthologous sequences. The maps show the distribution of putative Pf regulatory motifs (a) in ESTs retrieved for the gene upstream sequences and (b) in the upstream sequences of orthologous genes from other Plasmodium species. [file 1471-2164-10-18-S9.zip › Iengar_addnl_file9_bmcg/map_2ribont_uig.jpg]

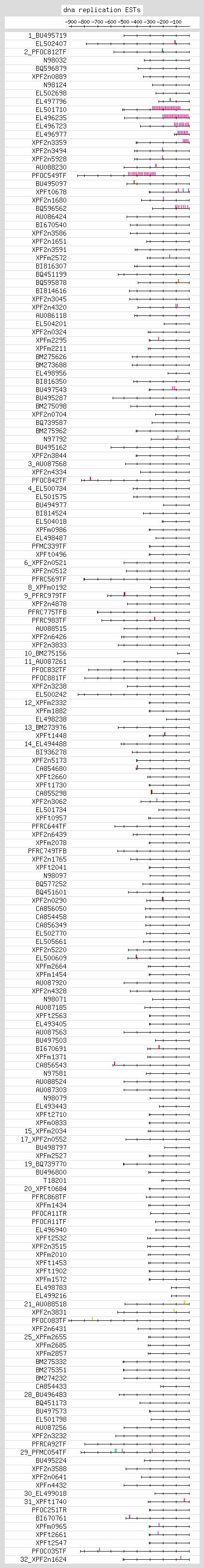

Supplement: Additional file 9 — Feature maps showing the occurrence of putative Pf regulatory motifs in ESTs and orthologous sequences. The maps show the distribution of putative Pf regulatory motifs (a) in ESTs retrieved for the gene upstream sequences and (b) in the upstream sequences of orthologous genes from other Plasmodium species. [file 1471-2164-10-18-S9.zip › Iengar_addnl_file9_bmcg/map_dnarep_est.jpg]

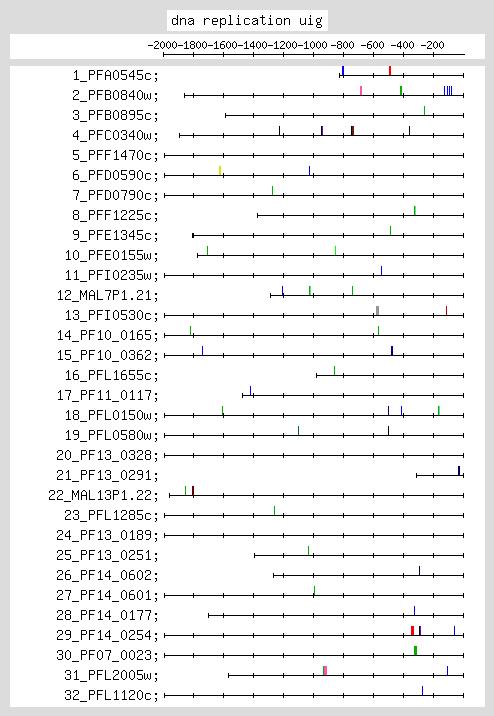

Supplement: Additional file 9 — Feature maps showing the occurrence of putative Pf regulatory motifs in ESTs and orthologous sequences. The maps show the distribution of putative Pf regulatory motifs (a) in ESTs retrieved for the gene upstream sequences and (b) in the upstream sequences of orthologous genes from other Plasmodium species. [file 1471-2164-10-18-S9.zip › Iengar_addnl_file9_bmcg/map_dnarep_uig.jpg]

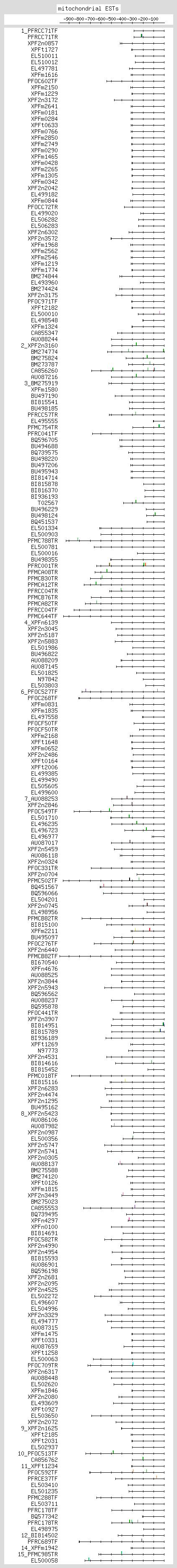

Supplement: Additional file 9 — Feature maps showing the occurrence of putative Pf regulatory motifs in ESTs and orthologous sequences. The maps show the distribution of putative Pf regulatory motifs (a) in ESTs retrieved for the gene upstream sequences and (b) in the upstream sequences of orthologous genes from other Plasmodium species. [file 1471-2164-10-18-S9.zip › Iengar_addnl_file9_bmcg/map_mito_est.jpg]

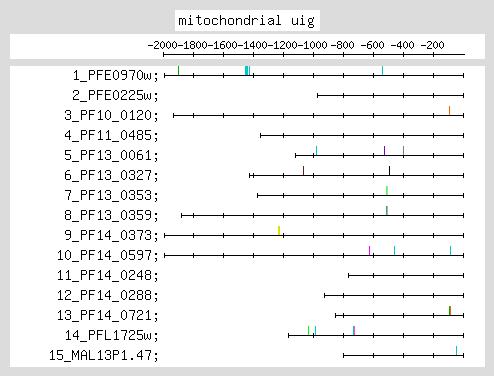

Supplement: Additional file 9 — Feature maps showing the occurrence of putative Pf regulatory motifs in ESTs and orthologous sequences. The maps show the distribution of putative Pf regulatory motifs (a) in ESTs retrieved for the gene upstream sequences and (b) in the upstream sequences of orthologous genes from other Plasmodium species. [file 1471-2164-10-18-S9.zip › Iengar_addnl_file9_bmcg/map_mito_uig.jpg]

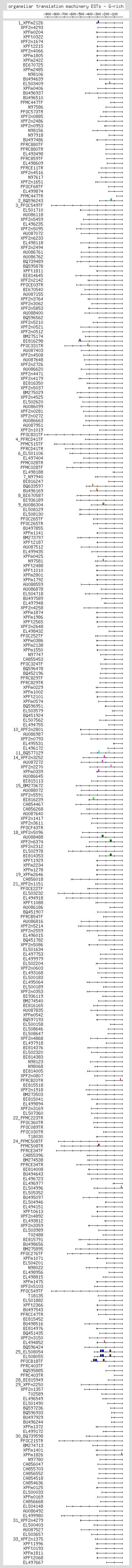

Supplement: Additional file 9 — Feature maps showing the occurrence of putative Pf regulatory motifs in ESTs and orthologous sequences. The maps show the distribution of putative Pf regulatory motifs (a) in ESTs retrieved for the gene upstream sequences and (b) in the upstream sequences of orthologous genes from other Plasmodium species. [file 1471-2164-10-18-S9.zip › Iengar_addnl_file9_bmcg/map_orgtrans_est_g_rich.jpg]

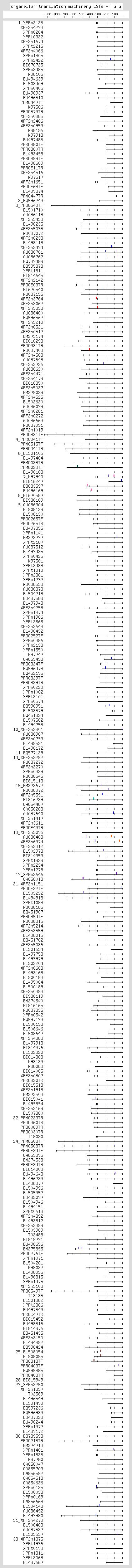

Supplement: Additional file 9 — Feature maps showing the occurrence of putative Pf regulatory motifs in ESTs and orthologous sequences. The maps show the distribution of putative Pf regulatory motifs (a) in ESTs retrieved for the gene upstream sequences and (b) in the upstream sequences of orthologous genes from other Plasmodium species. [file 1471-2164-10-18-S9.zip › Iengar_addnl_file9_bmcg/map_orgtrans_est_tgtg.jpg]

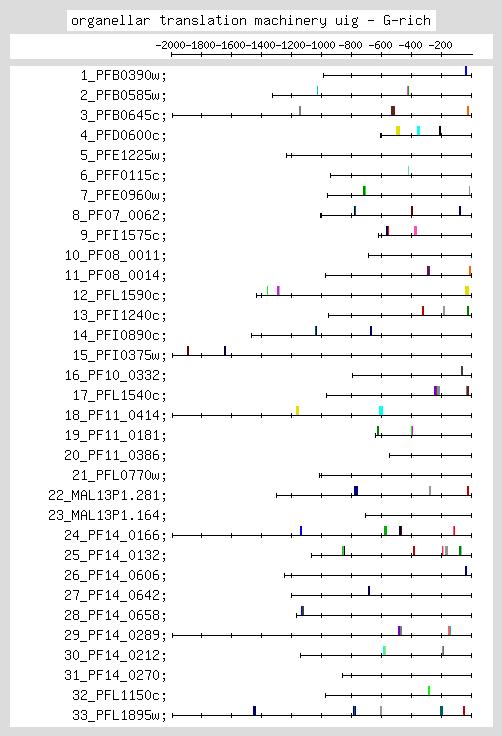

Supplement: Additional file 9 — Feature maps showing the occurrence of putative Pf regulatory motifs in ESTs and orthologous sequences. The maps show the distribution of putative Pf regulatory motifs (a) in ESTs retrieved for the gene upstream sequences and (b) in the upstream sequences of orthologous genes from other Plasmodium species. [file 1471-2164-10-18-S9.zip › Iengar_addnl_file9_bmcg/map_orgtrans_uig_g_rich.jpg]

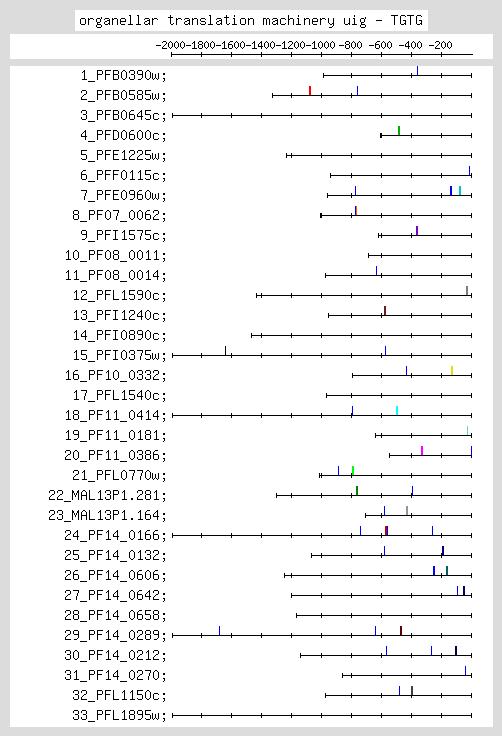

Supplement: Additional file 9 — Feature maps showing the occurrence of putative Pf regulatory motifs in ESTs and orthologous sequences. The maps show the distribution of putative Pf regulatory motifs (a) in ESTs retrieved for the gene upstream sequences and (b) in the upstream sequences of orthologous genes from other Plasmodium species. [file 1471-2164-10-18-S9.zip › Iengar_addnl_file9_bmcg/map_orgtrans_uig_tgtg.jpg]

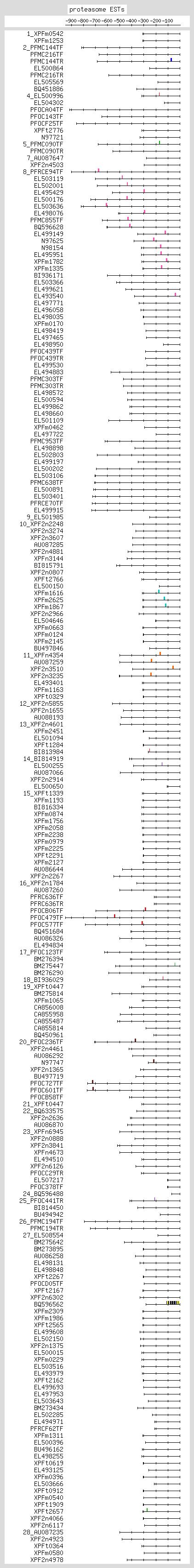

Supplement: Additional file 9 — Feature maps showing the occurrence of putative Pf regulatory motifs in ESTs and orthologous sequences. The maps show the distribution of putative Pf regulatory motifs (a) in ESTs retrieved for the gene upstream sequences and (b) in the upstream sequences of orthologous genes from other Plasmodium species. [file 1471-2164-10-18-S9.zip › Iengar_addnl_file9_bmcg/map_protea_est.jpg]

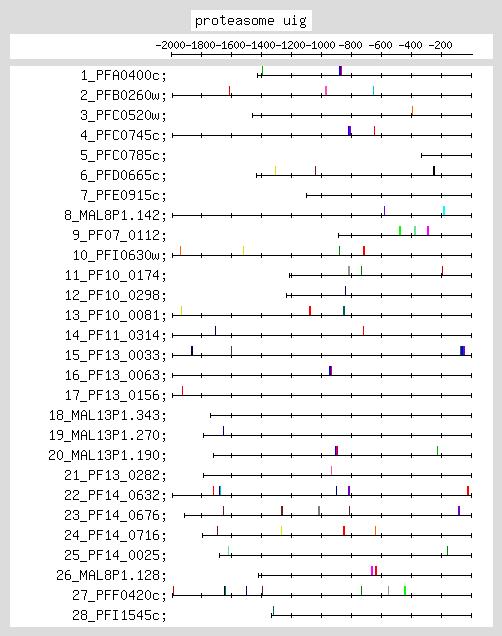

Supplement: Additional file 9 — Feature maps showing the occurrence of putative Pf regulatory motifs in ESTs and orthologous sequences. The maps show the distribution of putative Pf regulatory motifs (a) in ESTs retrieved for the gene upstream sequences and (b) in the upstream sequences of orthologous genes from other Plasmodium species. [file 1471-2164-10-18-S9.zip › Iengar_addnl_file9_bmcg/map_protea_uig.jpg]

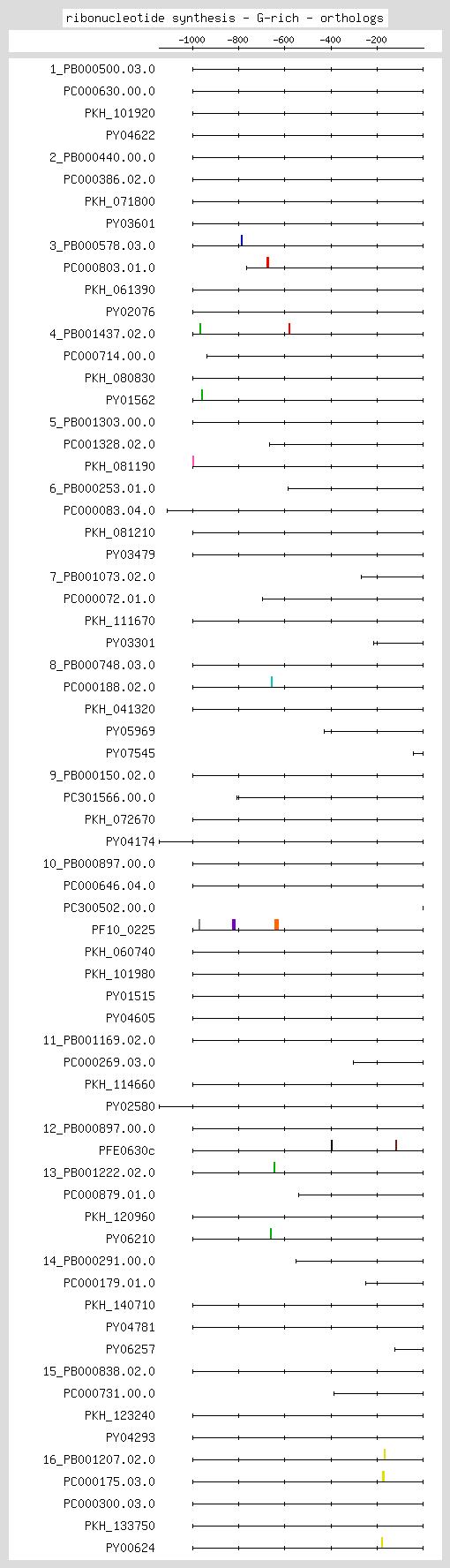

Supplement: Additional file 9 — Feature maps showing the occurrence of putative Pf regulatory motifs in ESTs and orthologous sequences. The maps show the distribution of putative Pf regulatory motifs (a) in ESTs retrieved for the gene upstream sequences and (b) in the upstream sequences of orthologous genes from other Plasmodium species. [file 1471-2164-10-18-S9.zip › Iengar_addnl_file9_bmcg/orthologs_map_2ribont_g_rich.jpg]

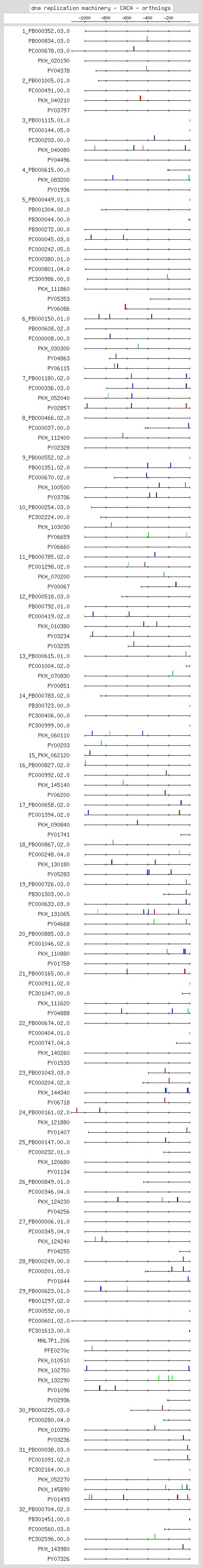

Supplement: Additional file 9 — Feature maps showing the occurrence of putative Pf regulatory motifs in ESTs and orthologous sequences. The maps show the distribution of putative Pf regulatory motifs (a) in ESTs retrieved for the gene upstream sequences and (b) in the upstream sequences of orthologous genes from other Plasmodium species. [file 1471-2164-10-18-S9.zip › Iengar_addnl_file9_bmcg/orthologs_map_dnarep_caca.jpg]

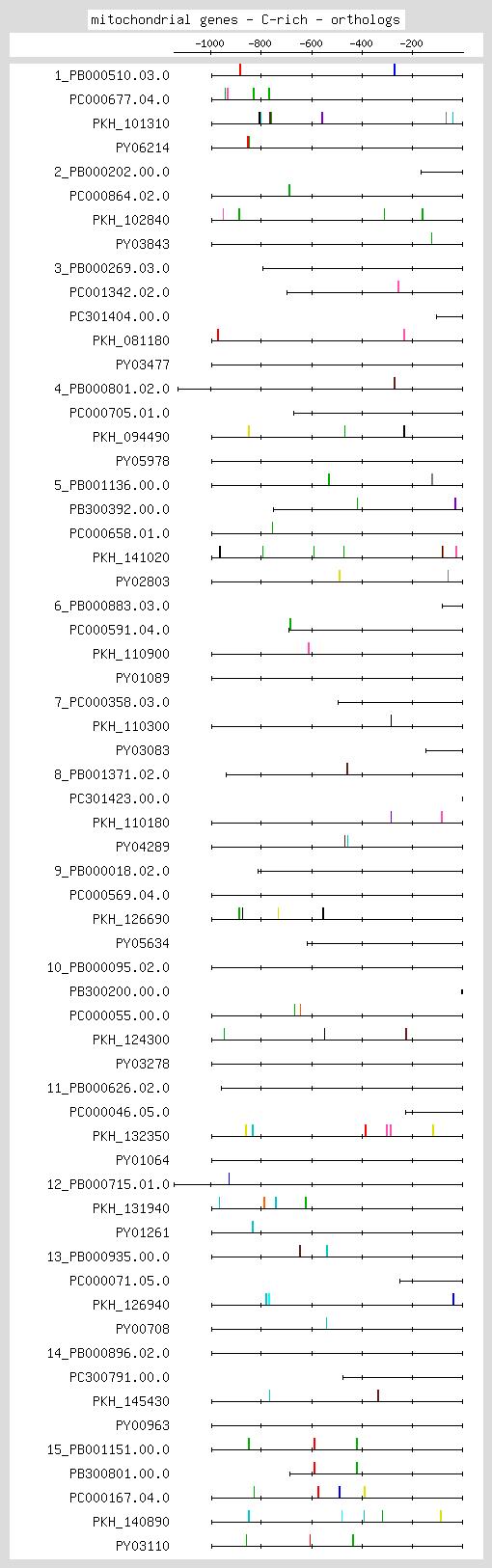

Supplement: Additional file 9 — Feature maps showing the occurrence of putative Pf regulatory motifs in ESTs and orthologous sequences. The maps show the distribution of putative Pf regulatory motifs (a) in ESTs retrieved for the gene upstream sequences and (b) in the upstream sequences of orthologous genes from other Plasmodium species. [file 1471-2164-10-18-S9.zip › Iengar_addnl_file9_bmcg/orthologs_map_mito_c_rich.jpg]

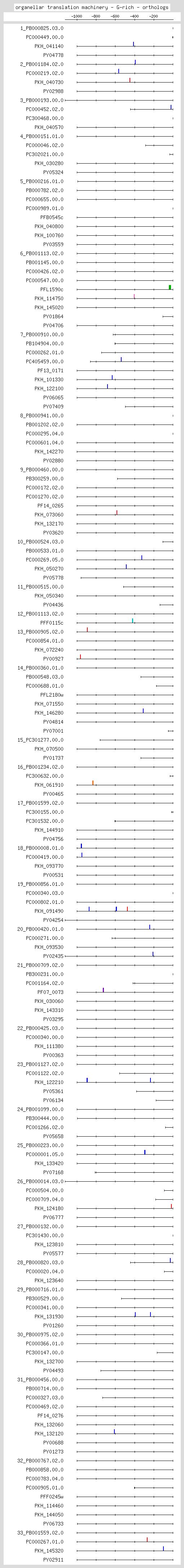

Supplement: Additional file 9 — Feature maps showing the occurrence of putative Pf regulatory motifs in ESTs and orthologous sequences. The maps show the distribution of putative Pf regulatory motifs (a) in ESTs retrieved for the gene upstream sequences and (b) in the upstream sequences of orthologous genes from other Plasmodium species. [file 1471-2164-10-18-S9.zip › Iengar_addnl_file9_bmcg/orthologs_map_orgtrans_g_rich.jpg]

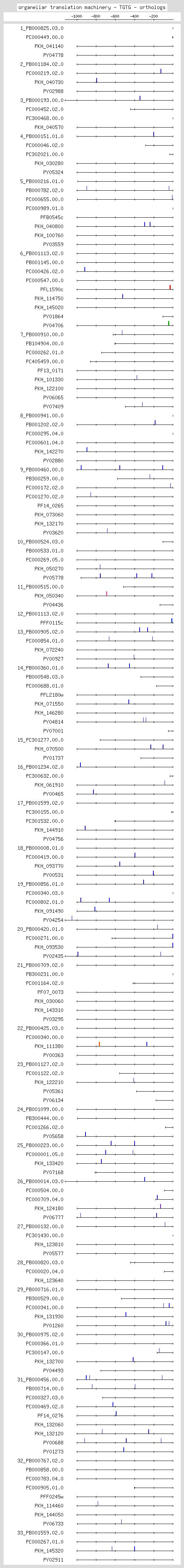

Supplement: Additional file 9 — Feature maps showing the occurrence of putative Pf regulatory motifs in ESTs and orthologous sequences. The maps show the distribution of putative Pf regulatory motifs (a) in ESTs retrieved for the gene upstream sequences and (b) in the upstream sequences of orthologous genes from other Plasmodium species. [file 1471-2164-10-18-S9.zip › Iengar_addnl_file9_bmcg/orthologs_map_orgtrans_tgtg.jpg]

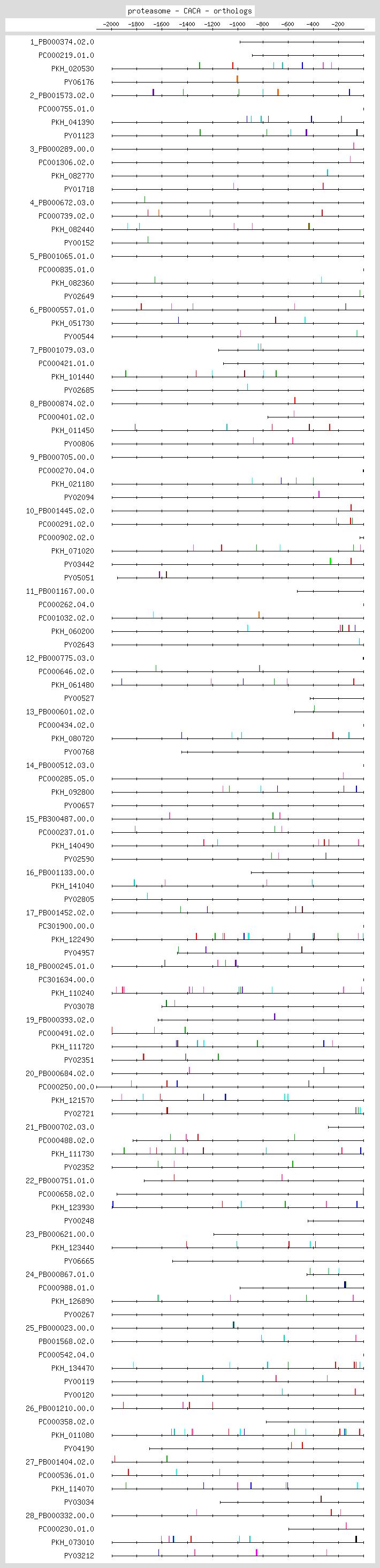

Supplement: Additional file 9 — Feature maps showing the occurrence of putative Pf regulatory motifs in ESTs and orthologous sequences. The maps show the distribution of putative Pf regulatory motifs (a) in ESTs retrieved for the gene upstream sequences and (b) in the upstream sequences of orthologous genes from other Plasmodium species. [file 1471-2164-10-18-S9.zip › Iengar_addnl_file9_bmcg/orthologs_map_protea_caca.jpg]
