# Supplementary material for: Identification of putative regulatory motifs in the upstream regions of co-expressed functional groups of genes in Plasmodium falciparum
Source: BMC Genomics. 2009 Jan 13;10:18. doi: 10.1186/1471-2164-10-18 (PMC2662883; doi:10.1186/1471-2164-10-18)
Supplement: Additional file 10 — Positional conservation information for the strong motif groups identified for the 13 functional groups of genes. The table summarizes the positional conservation information for motifs in all functional groups and helps the reader navigate through the Additional files. [file 1471-2164-10-18-S10.doc]

Additional file 10. Positional conservation information for the strong motifs identified in the 13 sets of upstream regions.

| Functional group | Strong motif group identified | †Motif type: location of positional conservation (number of occurrences) | ‡Relevant figure (F) or table (T) |
| --- | --- | --- | --- |
| Transcription machinery | G-rich | 4G+3G: ~ -1400 (4) | T-2, F-1, AF-6, F-5 |
| Cytoplasmic translation machinery | G-rich | 4G: ~ -800 (13), -1100 (10) | AF-1:9, AF-6, AF-7:5-7 |
|  | C-rich | 4C: ~ -900 (12); | AF-1:13, AF-6, AF-7:9-12 |
|  | TGTG | TGTG: ~ -1600 (6), -1750 (6) | AF-1:17, AF-6, AF-7:13 |
| Glycolytic pathway | G-rich |  | AF-1:30, AF-7:15 |
| *Ribonucleotide synthesis* | *G-rich* | *4G+3G+2G+1G: ~ -800 (8), -1300 (4), -1700 (4), -400 (6; p<0.22),* | *AF-1:36, AF-6, F-6* |
| Deoxynucleotide synthesis | No strong motif |  | AF-2:2 |
| *DNA replication machinery* | *CACA* | *~ -300 (8), -500 (5; p<0.10), -1000 (5; p<0.15)* | *AF-2:12, F-3, AF-6, F-7* |
|  | TGTG | ~ -250 (7) | AF-2:7-8, AF-7:23, AF-6 |
|  | G-rich | 2G+1G: ~-450 (11), -1600 (6) | AF-2:9-11, AF-7:21, AF-6 |
| TCA cycle | G-rich |  | AF-2:19, AF-7:25 |
| *Proteasome* | *CACA* | *between -800 and -1000 (15), -1600 and -1700 (8)* | *AF-2:29, AF-6, F-8* |
|  | G-rich | 4G+3G: ~ -1000 (7) | AF-2:25, AF-6, AF-7:27 |
|  | TGTG, TGTATG |  | AF-2:27, AF-6, AF-7:30 |
| *Mitochondrial genes* | *C-rich* | *4C+3C+2C: ~ -500 (7), -100 (4; p<0.08)* | *AF-2:37, F-2, AF-6, F-9* |
|  | *G-rich* | *4G+3G+2G+1G: between ~ -200 and -300 (7)* | *AF-2:38, AF-6, AF-7:33* |
|  | *TGTG* | *motifs occurred between ~ -200 and -450* | *AF-2:39, AF-6, AF-7:35* |
| *Organellar translation machinery* | *G-rich* | *4G+3G+2G; close to TLS, at ~ -30(13)* | *AF-2:45-46, AF-6, F-10, AF-7:37-38* |
|  | *TGTG* | *~ -600 (8), -800 (7), -50 (9; p<0.08), -400 (6; p<0.29),* | *AF-2:48, F-4, AF-6, F-11* |
|  | *C-rich* | *~-500 (15)* | AF-2:43, AF-7:39-40 |
| Merozoite invasion genes | TGCACA |  | AF-3:2-3, AF-6, AF-7:46 |
|  | TGTG | ~-100 (10), -1400 (8) | AF-3:5, AF-6, AF-7:47 |
|  | G-rich | 4G+3G+2G | AF-3:9, AF-6, AF-7:43-44 |
|  | C-rich | 4C+3C: ~-700 (10), -1900 (8) | AF-3:7, AF-6, AF-7:45-46 |
| Actin myosin motors | TGCACA |  | AF-3:16-17, AF-6, AF-7:51 |
|  | G-rich |  | AF-3:20-21, AF-6, AF-7:49-50 |
|  | TGTG |  | AF-3:18-19, AF-6, AF-7:51 |
| Early ring transcript genes | No strong motif |  | AF-4:2,4-6 |

†Column 3 specifies the types of sorted motifs examined (e.g., 4G+3G), the approximate locations of positional conservation, and the number of positionally conserved motifs observed; p-values have been given for sets of motifs which are of some interest and show positional conservation, but are statistically weakly significant (p>0.05).

‡Column 4 indicates the relevant Figures (F), Tables (T) and Additional files (AF) that the reader may turn to while following the 2^nd^ sub-section of Results (‘Positional Conservation of Motif Occurrences in the Upstream Regions’). Thus, AF-7:5-7 refers to slides 5 to 7 in Additional file 7.

The most interesting motifs are italicized.
