# Supplementary material for: Identification of putative regulatory motifs in the upstream regions of co-expressed functional groups of genes in Plasmodium falciparum
Source: BMC Genomics. 2009 Jan 13;10:18. doi: 10.1186/1471-2164-10-18 (PMC2662883; doi:10.1186/1471-2164-10-18)
Supplement: Additional file 9 — Feature maps showing the occurrence of putative Pf regulatory motifs in ESTs and orthologous sequences. The maps show the distribution of putative Pf regulatory motifs (a) in ESTs retrieved for the gene upstream sequences and (b) in the upstream sequences of orthologous genes from other Plasmodium species. [file 1471-2164-10-18-S9.zip › Iengar_addnl_file9_bmcg/readme_orthologs.doc]

*Searching for Pf regulatory motifs in the upstream sequences of ‘in genera’ orthologs*

The occurrence of putative regulatory motifs identified for a set of genes in *Pf*, in the upstream sequences of orthologous genes from other *Plasmodium* species, would afford considerable validity to the motifs. Therefore, an attempt was made to validate some of the positionally conserved motifs observed in the upstream sequences of the ribonucleotide synthesis, proteasome, DNA replication machinery, mitochondrial and organellar translation machinery sets of genes. Each of these sets of genes was considered, and for each gene in the set, the IDs of orthologous genes from other *Plasmodium* species (‘in genera’ orthologs, and also paralogs) were retrieved from PlasmoDB. The sequence retrieval tool of PlasmoDB was then used to retrieve the upstream sequence of each orthologous gene. 1000 nt upstream of the TLS were retrieved for the orthologous genes of all the above sets, except the proteasome set; for the proteasome set, 2000 nt upstream of the TLS were extracted because positionally conserved motifs in *Pf* were observed at ~-900 and -1600. The set of putative regulatory motifs (including the positionally conserved ones) observed in the upstream sequences of each set of genes is listed in Additional file 6. These motifs were searched for in the orthologous upstream sequences. The DNA pattern and feature map programs were used to search for and display motifs in the upstream sequences of orthologous genes.

It must be pointed out that the present exercise is one in pattern identification. For example, G-rich motifs identified for the *Pf* ribonucleotide synthesis genes and listed in Additional file 6, were searched for in the orthologous upstream sequences. Variants of the G-rich motif that are absent from Additional file 6, but are present in the orthologous upstream sequences, will not be picked up in the latter. For a complete list of over-represented G-rich motifs occurring in the orthologous upstream sequences, motif-searching procedures (AlignACE, MEME, Weeder) will have to be used. As the aim of the present exercise was only to check if (for example) the G-rich motifs identified for *Pf* ribonucleotide genes, occurred in the orthologous upstream sequences, only pattern identification was carried out. Matching motifs in the orthologous upstream sequences would thus be identified.

*Feature maps:*

2 feature maps are presented for each motif studied in a set of gene upstream sequences; viewing the 2 maps side by side would facilitate an understanding of the results. The filenames of the relevant pairs of feature maps are given below for each motif in each set of genes. One map displays the *Pf* gene upstream sequences, with the putative regulatory motifs being marked as vertical lines. The ID for each upstream sequence is prefixed by a serial number. The second map displays orthologous upstream sequences retrieved for the upstream sequences. Orthologous upstream sequences, retrieved for a single *Pf* upstream sequence, constitute a group. The first orthologous sequence in each group has been prefixed by the serial number of the upstream sequence it was retrieved for. Thus, for example, the group of orthologous sequences prefixed by 1 constitute sequences retrieved for the *Pf* upstream sequence prefixed 1, the group of orthologous sequences prefixed by 2 constitute sequences retrieved for the *Pf* upstream sequence prefixed 2, etc..

*Names of the relevant feature maps:*

*(i) Ribonucleotide synthesis genes – G-rich motif*

*Pf* upstream sequences with regulatory motifs marked = map_2ribont_uig.jpg

Orthologous upstream sequences with *Pf* regulatory motifs marked = orthologs_map_2ribont_g_rich.jpg

*(ii) DNA replication machinery genes – CACA motif*

*Pf* upstream sequences with regulatory motifs marked = map_dnarep_uig.jpg

Orthologous upstream sequences with *Pf* regulatory motifs marked = orthologs_map_dnarep_caca.jpg

*(iii) Proteasome – CACA motif*

*Pf* upstream sequences with regulatory motifs marked = map_protea_uig.jpg

Orthologous upstream sequences with *Pf* regulatory motifs marked = orthologs_map_protea_caca.jpg

*(iv) Mitochondrial genes – C-rich motif*

*Pf* upstream sequences with regulatory motifs marked = map_mito_uig.jpg

Orthologous upstream sequences with *Pf* regulatory motifs marked = orthologs_map_mito_c_rich.jpg

*(v) Organellar translation machinery – G-rich*

*Pf* upstream sequences with regulatory motifs marked = map_orgtrans_uig_g_rich.jpg

Orthologous upstream sequences with *Pf* regulatory motifs marked = orthologs_map_orgtrans_g_rich.jpg

*(vi) Organellar translation machinery – TGTG motif*

*Pf* upstream sequences with regulatory motifs marked = map_orgtrans_uig_tgtg.jpg

Orthologous upstream sequences with *Pf* regulatory motifs marked = orthologs_map_orgtrans_tgtg.jpg

Observations made from the feature maps for the different sets of genes are described in the Results section.

Upstream CACA motifs for the DNA replication machinery and proteasome sets of genes, C-rich motifs for the mitochondrial genes and TGTG motifs for the organellar translation machinery genes were all observed in the orthologous upstream sequences. Oddly, G-rich motifs for the ribonucleotide synthesis and organellar translation machinery sets of genes were not observed in the orthologous upstream sequences from other *Plasmodium* species. Since G-rich motifs for *two* sets of genes were not observed, it is possible that the occurrence of these motifs, in these sets of genes, is unique to *Pf*.
