# Supplementary material for: Identification of putative regulatory motifs in the upstream regions of co-expressed functional groups of genes in Plasmodium falciparum
Source: BMC Genomics. 2009 Jan 13;10:18. doi: 10.1186/1471-2164-10-18 (PMC2662883; doi:10.1186/1471-2164-10-18)
Supplement: Additional file 9 — Feature maps showing the occurrence of putative Pf regulatory motifs in ESTs and orthologous sequences. The maps show the distribution of putative Pf regulatory motifs (a) in ESTs retrieved for the gene upstream sequences and (b) in the upstream sequences of orthologous genes from other Plasmodium species. [file 1471-2164-10-18-S9.zip › Iengar_addnl_file9_bmcg/readme_retrieving_est_seqs.doc]

*Retrieving EST sequences*

Expressed sequence tags or ESTs are small pieces of DNA sequence that are generated by sequencing the ends of an expressed gene. As ESTs contain extensions to the 5′ end of the target mRNA, one can try to search for regulatory motifs in these additional sequence stretches. An attempt was, therefore, made to see if some of the positionally conserved putative regulatory motifs identified in the present study were to be found in *Pf* ESTs. The systematic presence, in the ESTs, of regulatory motifs identified for a set of co-expressed genes would suggest that the motif was playing a regulatory role at the post-transcriptional (mRNA) rather than at the transcriptional (DNA) level. *Pf* ESTs were retrieved from PlasmoDB for the upstream sequences of the ribonucleotide synthesis, proteasome, DNA replication machinery, mitochondrial and organellar translation machinery co-expression clusters; these clusters were of interest because positionally conserved upstream motifs were observed for them. ESTs for each upstream sequence in each cluster were retrieved from PlasmoDB by pasting the upstream sequence, selecting the blastn option (e-value, 0.05; low complexity filter on) and selecting *Pf* as organism.

Upstream motifs for the above sets of co-expressed genes are listed in Additional file 5; motifs listed for each set of genes were searched for in the EST sequences retrieved for the set of genes. The DNA pattern and feature map programs were used for searching and displaying EST motifs. In Additional file 6, motifs occurring in each upstream sequence of each set of co-expressed genes are listed. An attempt was made to check if the motif(s) occurring in each upstream sequence of a set of genes in Additional file 6 were also occurring in the ESTs obtained for the upstream sequence. Checking for the presence of the upstream motif in the ESTs ensured that relevant 5’ ESTs were being examined. Further, for the EST to be considered valid, the start codon was required to be present in the EST, and the regulatory motif was required to occur at the appropriate upstream position with respect to the start codon. Only if an EST motif is observed at the appropriate position in the region upstream of the start codon can it be regarded as a putative regulatory motif occurring in the expressed gene sequence (mRNA).

*Legend for feature maps*

2 feature maps are presented for each motif studied in a set of gene upstream sequences; viewing the 2 maps side by side would facilitate an understanding of the results. The filenames of the relevant pairs of feature maps are given below for each set of genes. One map displays the gene upstream sequences, with the putative regulatory motifs being marked as vertical lines. The ID for each upstream sequence is prefixed by a serial number. The second map displays ESTs retrieved for the upstream sequences. ESTs retrieved for a single upstream sequence constitute a group. The first EST in each group has been prefixed by the serial number of the upstream sequence it was retrieved for. Thus, for example, the group of ESTs prefixed by 1 constitute ESTs retrieved for the upstream sequence prefixed 1, the group of ESTs prefixed by 2 constitute ESTs retrieved for the upstream sequence prefixed 2, etc.. If no ESTs were retrieved for an upstream sequence, then the prefix of the upstream sequence was not used for the ESTs. The relevant set of motifs listed in Additional file 5 was used to identify regulatory motifs in all the EST sequences for the set of upstream sequences. The regulatory motifs in the ESTs were then scrutinized in greater detail; the occurrence of the motifs listed in Additional file 6, in the ESTs, was checked for. Only ESTs in which the regulatory motif occurred at the appropriate upstream location with respect to the start codon (given in Additional file 6) were considered while discussing the results. Observations made from the feature maps for the different sets of genes are described below.

Names of the relevant feature maps:

Upstream sequence and regulatory motif map = map_2ribont_uig.jpg

EST sequence and regulatory motif map = map_2ribont_est.jpg

*Ribonucleotide synthesis:* 110 ESTs were retrieved for 14 out of the 16 upstream sequences of this set of genes; for 2 upstream sequences, no ESTs were found.

No G-rich motif was identified in the ESTs of 10 upstream sequences.

For upstream sequence 7, 1 EST was picked and the EST had a motif. The EST motif matches the upstream motif at -443; but the EST motif occurs at ~ -390; thus the position of the EST motif is too close to the end of the EST sequence and the EST sequence does not have the gene start codon.

For upstream sequence 9, 14 ESTs were picked and only 2 of these had a motif; the EST motifs did not match the upstream motif.

For upstream sequence 11, 3 ESTs were picked and 2 out of 3 had a motif; both EST motifs matched the upstream motif at -158, a motif which is not positionally conserved and which occurs close to the TLS; both ESTs, however, had the start codon.

For upstream sequence 13, 50 ESTs were picked and only 1 had the motif; the EST motif matches the uptream motif at -1045 (but the EST is only ~150 nt in length and does not include the start codon).

Thus, in the ESTs of 1 upstream sequence, an upstream motif which occurs close to the TLS (at -158), and which is not positionally conserved, is observed; in the ESTs of 2 upstream sequences, upstream motifs were observed, but the ESTs did not include the start codon, and in the EST of 1 upstream sequence, the motif observed was not an upstream one.

Thus, in the majority of ESTs, the G-rich motif was *not* observed. G-rich motifs occurring at the appropriate upstream position with respect to the start codon (~-400, -800, etc., where positional conservation is ocurs), were *not* observed in any of the ESTs. This would suggest that the motif is not observed in the expressed gene (transcribed and spliced mRNA) and that the motif plays a regulatory role at the transcriptional (DNA) level and not at the post-transcriptional (RNA) level.

---------------------------------------------------------------------------------------------------------------

Names of the relevant feature maps:

Upstream sequence and regulatory motif map = map_dnarep_uig.jpg

EST sequence and regulatory motif map = map_dnarep_est.jpg

*DNA Replication machinery:* 189 ESTs were retrieved for the 32 upstream sequences of this set of genes. For 9 upstream sequences, no ESTs were retrieved. In the ESTs of 13 upstream sequences, no motif was found. In the ESTs for the remaining 10 upstream sequences, motifs were observed.

Upstream sequence 1 – 1 EST out of 2 had a motif; the EST motif is not the same as the upstream motif.

Upstream sequence 2 – 21 ESTs out of 50 had motifs. The upstream motif at ~ -100 (ACATACAT) occurs 4 to 5 times in upstream sequence 2 (PFB0840w); the majority of EST motifs matched this motif. Conspicuously, the motif occurred 18 to 20 times in some of the ESTs. Closer comparison of upstream sequence 2 and the ESTs with the motif showed that while the upstream motif is found in the EST sequences, the rest of the EST sequence does not match with the upstream sequence. Thus, upstream sequence 2 and the ESTs with the motif are not related. The upstream motif at -688 matched EST motifs in PF0C812TF and EL497796; both EST sequences, however, do not include the start codon.

**Upstream sequence 3 – 1 EST out of 3 had a motif; the EST motif matches the upstream motif at -264; the start codon is present in the EST.

**Upstream sequence 9 – 2 ESTs out of 7 had motifs, the EST motifs match the upstream motif at -489; the start codon is present in the EST;

*Upstream sequence 13 – 1 EST out of 2 had a motif, the EST motif matches the first upstream motif at -118; the start codon is present in the EST.

Upstream sequence 14 – 7 ESTs out of 50 had motifs; all EST motifs match the first upstream motif at -572; while the upstream motif is present in the ESTs, the rest of the ESTs are unrelated to the upstream sequence.

*Upstream sequence 21 – 3 ESTs out of 3 had a motif; all EST motifs match the first upstream motif at -34; the start codon is present in all ESTs.

**Upstream sequence 29 – 1 EST out of 5 had 4 motifs; the EST motifs match the second and third upstream motifs (at -294, -350); the start codon is present in the EST.

*Upstream sequence 31 – 5 ESTs out of 7 had motifs; 4 EST motifs match the first upstream motif at ~-111; the start codon is present in some of these ESTs. The motif in the EST, BI670761, does not match the upstream motifs.

Upstream sequence 32 – 1 EST out of 1 had a motif; the EST motif matches the upstream motif at -280 but the rest of the EST sequence is unrelated to the upstream sequence.

Thus, in the ESTs for 6 upstream sequences, motifs matching the upstream CACA motifs and occurring at the appropriate position with respect to the start codon, were identified. In the ESTs of 3 of these upstream sequences, the identified EST motifs match positionally conserved upstream motifs (occurring at -264, ~ -300 and -489). Thus, some of the positionally conserved upstream motifs were observed in the ESTs. Owing to this latter observation, one cannot rule out the possibility that the CACA motif plays a role at the RNA or post-transcriptional level. One must, however, bear in mind that no upstream motifs were identified in the ESTs of 13 other upstream sequences.

---------------------------------------------------------------------------------------------------------------

Names of the relevant feature maps: map_protea_uig.jpg, map_protea_est.jpg

*Proteasome:* 229 ESTs were retrieved for the 28 proteasome upstream sequences. For 2 upstream sequences, no ESTs were retrieved. In the ESTs for 12 upstream sequences, no motifs were observed. In the ESTs for the remaining 14 upstream sequences, motifs were observed.

Upstream sequence 2 – motif occurs in 1 out of 7 ESTs. The EST motif matches the upstream motif at -1619. The EST, however, does not have the start codon.

Upstream sequence 4 – motif occurs in 1 out of 7 ESTs. EST motif does not match any of the upstream motifs.

Upstream sequence 5 – motif occurs in 1 out of 2 ESTs; uig has no motifs, so EST motif does not match uig motif

*Upstream sequence 8 (MAL8P1.142) – motif occurs in 16 out of 50 ESTs. The EST motifs match the upstream motif at -190. The start codon is present in the ESTs.

Upstream sequence 10 – motif occurs in 3 out of 19 ESTs. EST motif does not match any of the upstream motifs.

*Upstream sequence 11 – motif occurs in 4 out of 7 ESTs. EST motifs match upstream motif at -199. The start codon is present in some of the ESTs.

Upstream sequence 13 – motif occurs in 1 out of 5 ESTs; EST motif does not match any of 3 upstream motifs.

Upstream sequence 14 – motif occurs in 1 out of 4 ESTs; EST motif does not match any of 2 upstream motifs.

Upstream sequence 16 – motif occurs in 3 out of 10 ESTs; EST motif does not match upstream motif.

Upstream sequence 17 – motif occurs in 1 out of 4 ESTs. EST motif does not match upstream motif.

Upstream sequence 18 – motif occurs in 1 out of 1 EST; however, no motifs occur in the upstream sequence 18. Thus, there are no motifs in the upstream sequence to which the EST motifs can be related.

*Upstream sequence 20 – motif occurs in 4 out of 9 ESTs. EST motifs match upstream motif at -232. The start codon is also present in the ESTs.

Upstream sequence 25 – motif occurs in 1 out of 3 ESTs. EST motif does not match any upstream motif.

Upstream sequence 27 (PFF0420c) – in 1 EST sequence (BQ596562) out of 50 retrieved for this upstream sequence, 12-15 motifs occur. The EST motifs do not match any of the 7 upstream motifs. The EST and upstream sequences are different.

In the ESTs for 3 proteasome upstream sequences, motifs matching upstream CACA motifs (occurring at -190, -199 and -232) were observed. In none of the ESTs, however, were motifs matching positionally conserved upstream CACA motifs (occurring at ~-900) observed. This would suggest that the positionally conserved upstream motifs were not occurring in the expressed gene (mRNA) as they were not playing a regulatory role at the post-transcriptional level, but were playing a regulatory role at the DNA level, as transcription regulatory motifs.

---------------------------------------------------------------------------------------------------------------

Names of the relevant feature maps: map_mito_uig.jpg, map_mito_est.jpg

*Mitochondrial genes:* 244 EST sequences were retrieved for the 15 mitochondrial upstream sequences. For 2 upstream sequences, no ESTs were retrieved. In the ESTs of 3 upstream sequences, no motifs were observed. In the ESTs for the remaining 10 upstream sequences, motifs were observed.

Upstream sequence 1 – motifs occur in 2 out of 50 ESTs; 1 EST motif matches the upstream motif at -544, but the EST does not include the start codon (upstream motif is at -544 and EST motif is at -210). The upstream motifs at -1453 and -544 are the same. Although the upstream motif is present in the EST, the rest of the EST sequence differs from the upstream sequence.

Upstream sequence 2 – motifs occur in 6 out of 6 ESTs; however, no motifs occur in the upstream sequence 2. Thus there are no motifs in the upstream sequence to which the EST motifs can be related.

Upstream sequence 3 – motifs occur in 11 out of 38 ESTs. 1 EST motif (occurring at ~ -50 in PFMC754TR) matches the only upstream motif at -98. Although the upstream motif is present in the EST, the rest of the EST sequence is different from the upstream sequence.

**Upstream sequence 6 – motifs occur in 2 ESTs out of 17; EST motif at ~-750 in PF0C527TF alone matches the upstream motif at -497. The start codon is present in the EST.

Upstream sequence 7 – motifs occur in 15 out of 50 ESTs. The upstream motif at -515 is observed in 4 ESTs; these ESTs, however, do not have the start codon. The remaining EST motifs are not related to the upstream motif.

Upstream sequence 8 – motifs occur in 7 out of 50 ESTs. The upstream motif at -515 is observed once in EL500356. The EST, however, does not have the start codon.

*Upstream sequence 10 – motifs occur in 2 out of 2 ESTs. The upstream motif at -90 is observed in the 2 ESTs, and in both ESTs, the start codon is present. The upstream motif at -466 is not present in the ESTs.

Upstream sequence 11 – motifs occur in 3 out of 12 ESTs; however, no motifs occur in the upstream sequence 11. Thus there are no motifs in the upstream sequence to which the EST motifs can be related.

Upstream sequence 12 – motif occurs in 1 out of 2 ESTs; however, no motifs occur in the upstream sequence 12. Thus there are no motifs in the upstream sequence to which the EST motifs can be related.

*Upstream sequence 15 – motifs occur in 2 out of 2 ESTs. The upstream motif at -52 is present in both ESTs. The start codon is also present in both ESTs.

In the ESTs for 3 mitochondrial upstream sequences, motifs matching upstream C-rich motifs and occurring at the appropriate position with respect to start codon, were observed. In a single EST of one of these three upstream sequences, the identified EST motif matched a positionally conserved upstream motif (occurring at -497). Thus, a positionally conserved upstream C-rich motif was observed in a single EST. This, however, would not be sufficient evidence to suggest that the C-rich motif plays a regulatory role at the post-transcriptional (RNA) level.

---------------------------------------------------------------------------------------------------------------

Names of the relevant feature maps: map_orgtrans_uig_g_rich.jpg, map_orgtrans_est_g_rich.jpg

*Organellar translation machinery (G-rich motif):* 339 ESTs were retrieved for 33 organellar translation machinery upstream sequences. For 10 upstream sequences, no ESTs were retrieved. In the ESTs for 10 sequences, no motifs were found. In the ESTs for the remaining 13 sequences, motifs were observed.

Upstream sequence 1 – motifs occur in 2 out of 37 ESTs. The EST motifs do not match the upstream motif at -45.

Upstream sequence 2 – motifs occur in 1 out 1 ESTs. An EST motif matches the upstream motif at -433, but no start codon is present in the EST.

Upstream sequence 3 – motifs occur in 2 out of 50 ESTs. The EST motifs do not match any of the 3 upstream motifs.

Upstream sequences 4, 6, 8, 10, 19, 22, 28, 29, 31, 33 – no motifs occur in the ESTs retrieved for these sequences.

**Upstream sequence 7 – 2 motifs occur in 4 ESTs. The EST motifs match the upstream motif at -19. The start codon is present in the ESTs.

Upstream sequence 9 – motifs occur in 2 out of 48 ESTs. One EST motif matches the upstream motif at -571, but the EST does not have the start codon.

**Upstream sequence 11 – motifs occur in 1 out of 1 ESTs. One EST motif matches the upstream motif at -17; the EST has the start codon.

Upstream sequence 14 – motifs occur in 4 out of 6 ESTs. All 4 EST motifs match upstream motif at -675, but the ESTs do no have start codons.

Upstream sequence 15 – motifs occur in 1 out of 10 ESTs. The EST motif matches the upstream motif at -1649, but the EST lacks a start codon.

Upstream sequence 18 – motifs occur in 3 out of 8 ESTs. The EST motifs match upstream motif at -614, but the ESTs lack start codons.

Upstream sequence 21 – motif occurs in 1 out of 50 ESTs; however, no motifs occur in the upstream sequence 21. Thus there are no motifs in the upstream sequence to which the EST motifs can be related.

Upstream sequence 24 – motifs occur in 3 out of 45 ESTs. The EST motif on PFMC508TR matches the upstream motif at -1142, but this EST lacks the start codon.

**Upstream sequence 25 – motifs occur in 3 out of 6 ESTs; 3EST motifs match the upstream motif at -83, 3 EST motifs match the upstream motif at -176 and 3 EST motifs match the upstream motif at -200. In all 3 ESTs, the start codon is present.

Upstream sequence 30 – motif occurs in 1 out of 18 ESTs. The EST motif matches no upstream motif.

In the upstream sequences of this set of genes, G-rich motifs (quite uncharacteristically) frequently occur close to the TLS. Considering that (i) a sizeable number of the motifs occur close to the TLS, and (ii) that motifs occurring close to the TLS, rather than those occurring further upstream, are more likely to be transcribed into the ESTs, it is quite surprising to find a distinct paucity of EST motifs matching upstream ones, in the ESTs for this set of upstream sequences. In the ESTs for 2 upstream sequences, motifs matching upstream G-rich motifs (occurring at -19 and -17) were observed. For the upstream sequence 25 (PF14_0132), 3 interesting ESTs were observed; each EST had 3 motifs matching upstream G-rich motifs (occurring at -83, -176 and -200), and each EST also had the start codon. Thus, altogether, in the ESTs of only 3 upstream sequences out of 33, were motifs matching upstream motifs observed. More evidence would, therefore, be needed to suggest that the motifs play a regulatory role at the post-transcriptional level.

---------------------------------------------------------------------------------------------------------------

Names of the relevant feature maps: map_orgtrans_uig_tgtg.jpg, map_orgtrans_est_tgtg.jpg

*Organellar translation machinery (TGTG motif):* 339 ESTs were retrieved for 33 organellar translation machinery upstream sequences. For 10 upstream sequences, no ESTs were retrieved. In the ESTs for 9 sequences, no motifs were found. In the ESTs for the remaining 14 sequences, motifs were observed.

Upstream sequence 1 – motif occurs in 1 out of 38 ESTs. The EST motif matches the upstream motif at -363, but the rest of the EST and upstream sequences are diferent. Upstream sequences 2, 4, 8, 11, 14, 22, 28, 29, 33 – no motifs occur in the ESTs retrieved for these sequences.

Upstream sequence 3 – motifs occur in 8 out of 50 ESTs; however, no motifs occur in the upstream sequence 3. Thus there are no motifs in the upstream sequence to which the EST motifs can be related.

**Upstream sequence 6 – motifs occur in 5 out of 9 ESTs. The motif in EST PFMC028TF matches the upstream motif at -18; the EST has a start codon.

**Upstream sequence 7 – motifs occur in 4 out 4 ESTs. 3 EST motifs match the upstream motif at -84 and 2 EST motifs match the upstream motif at -140. ESTs have start codons.

Upstream sequence 9 – motifs occur in 4 out of 48 ESTs. The EST motifs do not match the upstream motif.

Upstream sequence 10 – motif occurs in 1 out of 5 ESTs; however, no motifs occur in the upstream sequence 10. Thus there are no motifs in the upstream sequence to which the EST motifs can be related.

Upstream sequence 15 – motifs occur in 5 out of 10 ESTs. 4 EST motifs match the upstream motif at -576, but the rest of the EST and upstream sequences are different. 1 EST motif matches the upstream motif at -1646, but the EST lacks the start codon.

Upstream sequence 18 – motifs occur in 3 out of 8 ESTs. 2 EST motifs match the upstream motif at -501, but the ESTs lack start codon; 2 EST motifs match the upstream motif at -796, but the ESTs lack the start codon; 1 EST motif matches upstream motif at -796, but the EST motif occurs *after* the start codon.

**Upstream sequence 19 – motifs occur in 2 out of 2 ESTs. The EST motifs match the upstream motif at -32. The ESTs have start codons.

Upstream sequence 21 – motifs occur in 4 out of 50 ESTs. The EST motifs match the upstream motif at -890, but the rest of the EST and upstream sequences are different.

Upstream sequence 24 – motifs occur in 4 out of 45 ESTs. The EST motifs match upstream motifs at -263 and -743 (both upstream motifs are TGTGAA), but the rest of the EST and upstream sequences are different.

**Upstream sequence 25 – motifs occur in 4 out of 6 ESTs. EST motifs match upstream motif at -195. The ESTs have start codons.

Upstream sequence 30 – motifs occur in 4 out of 18 ESTs. The EST motifs match the upstream motifs at -269, -572 (both the upstream motifs are the same, TGTGAA), but the rest of the EST and upstream sequences are different.

**Upstream sequence 31 – motif occurs in 1 out 4 ESTs. The EST motif matches the upstream motif at -42. The EST has the start codon.

In the ESTs for 5 out of 33 organellar translation machinery upstream sequences, motifs matching upstream TGTG motifs (occurring at -18, -84, -140, -32, -195, -42) were observed. Most of these EST motifs match upstream motifs occurring close to the TLS. More evidence would be needed to suggest that the upstream TGTG motifs observed for this set of genes play a regulatory role at the post-transcriptional level.

---------------------------------------------------------------------------------------------------------------
